# Modular Synthesis of Monoanionic PN Ligands Leads to Unexpected Structural Diversity in Lanthanum Chemistry

**DOI:** 10.1021/acs.inorgchem.4c02897

**Published:** 2024-10-15

**Authors:** Benjamin Wittwer, Florian Hett, Michael Seidl, Stephan Hohloch

**Affiliations:** Faculty of Chemistry and Pharmacy, Institute of General, Inorganic and Theoretical Chemistry, Leopold-Franzens-University Innsbruck, Innrain 80-82, 6020 Innsbruck, Austria

## Abstract

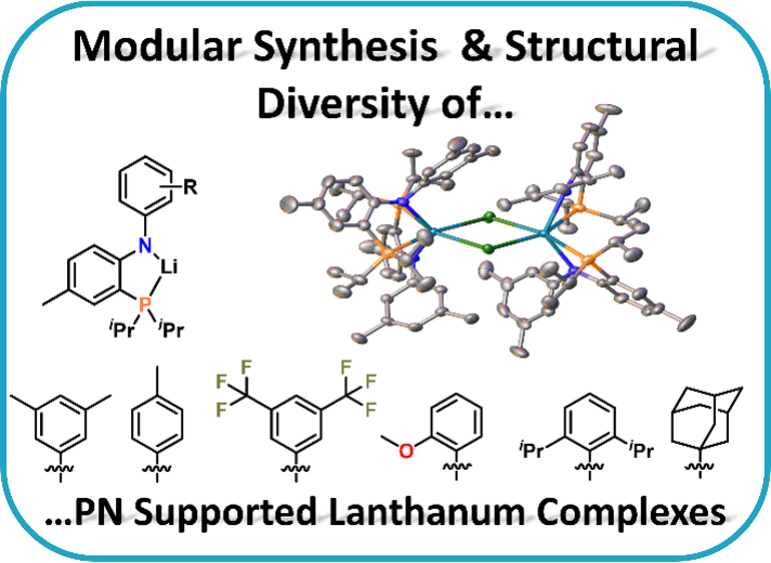

We report a new synthetic entry to a series of *N*-substituted anilidophosphine ligands (short HPN^R^, R = pTol (**HPN**^**Tol**^), 3,5-dimethylphenyl (**HPN**^**3,5Me**^), 3,5-bis(trifluoromethyl)phenyl (**HPN**^**3,5CF3**^), 2-methoxyphenyl (**HPN**^**OMe**^), diisopropylphenyl (**HPN**^**Dipp**^), and adamantyl (**HPN**^**Ad**^)), allowing a detailed tuning of their steric (and electronic) properties. **HPN**^**R**^ could be converted into their lithium salts **LiPN**^**R**^, which are effective precursors for salt metathesis reactions. The new ligands are used for the synthesis of an array of lanthanide complexes using LaCl_3_(THF)_1.2_ as a precursor. Depending on the steric bulk of the anilidophosphine ligand, either chloride-bridged dimers of the general formula [(PN^R^)_2_La(μ-Cl_2_)La(PN^R^)_2_] (R = pTol (**3f**), 3,5-dimethylphenyl (**3d**) and adamantyl (**3a**)) or mononuclear complexes of the general formula (PN^R^)_2_LaCl (R = diisopropylphenyl (**3e**)) are observed, if the complexation reaction is carried out in toluene. Contrary, if salt metathesis reactions are carried out in dimethoxyethane (DME) as a coordinating solvent, *–ate* complexes of the general formula [(PN^R^)_2_La(μ-Cl_2_)Li(DME)] (R = Adamantyl (**4a**) and R = 2-methoxyphenyl (**4d**)) or [Li(DME)_3_][(PN^R^)_2_LaCl_2_] (R = 3,5-bis(trifluoromethyl)phenyl (**4b**), R = pTol (**4f**) and R = 3,5-dimethylphenyl (**4d**)) are observed. All ligands and complexes have been thoroughly characterized by 1D and 2D NMR spectroscopy, IR, and X-ray crystallography. Finally, the steric demand of the new anilidophosphine ligands is evaluated using SambVca simulations.

## Abstract 2

The synthesis of six new bidentate, monoanionic PN^R^ ligands with different steric and electronic properties is reported. Their complexation behavior is further evaluated in lanthanide chemistry, revealing, depending on the steric bulk of the new PN^R^ ligands and solvents, an unexpected large variety of neutral and anionic lanthanum complexes.

## Introduction

**Figure 1:**
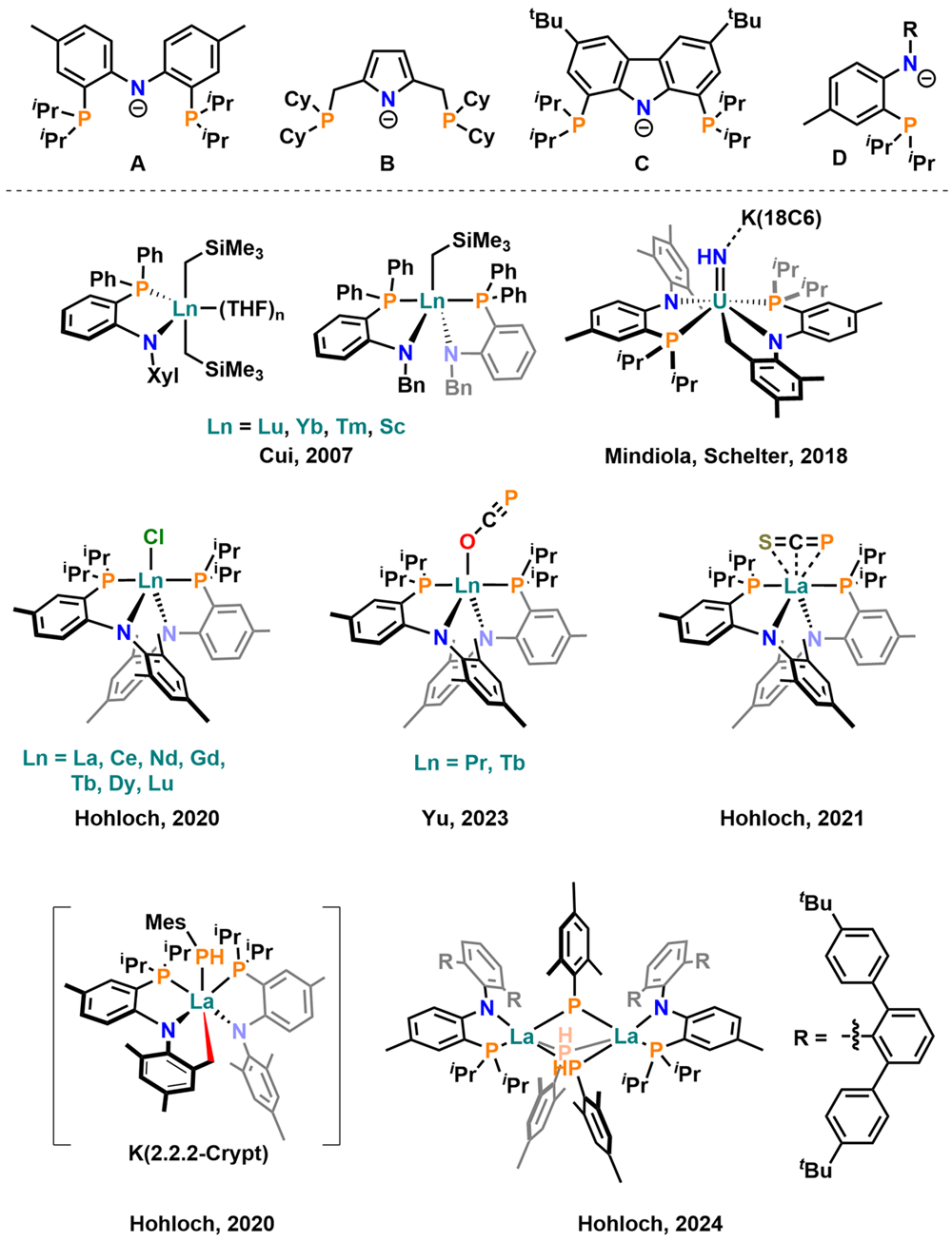
Selected monoanionic PN(P) ligands typically used in rare earth metal chemistry so far (top) and selected examples of f-element complexes, supported by bidentate monoanionic PN ligands.

Unarguably, amido- and anilidophosphine ligands of the PN(P) type (Figure 1, top) have led to multiple breakthroughs and paradigm changes in chemistry in the past decades.^1−11^ For example, in the realm of early transition metal chemistry, these ligands led to the development and stabilization of highly reactive metal–ligand multiple bonds,^12−20^ while in the late transition metal regime, they were capable of stabilizing terminal iridium, platinum, and palladium nitrido/nitrene complexes^21−23^ as well as rare 1,2-bridging coordination of [P_2_]^0/–1/–2^ on platinum,^24^ or recently allowed the isolation of a Pd(I) CO complex.^25^ However, their chemical potential is not only limited to the d-block elements but also in main group chemistry PN(P) ligands have led to multiple interesting discoveries.^26−30^ Recently, they have also been introduced to f-element chemistry,^31,32^ where they were found to stabilize a highly reactive lutetium phosphinidene,^33^ a wide array of cerium complexes,^34^ various polyhydride complexes,^35−37^ and polymerization catalysts.^38−41^ Additionally, bidentate PN ligands have been used to explore the reactivity of polarized lanthanum–phosphorus bonds.^42−44^

**Scheme 1:**
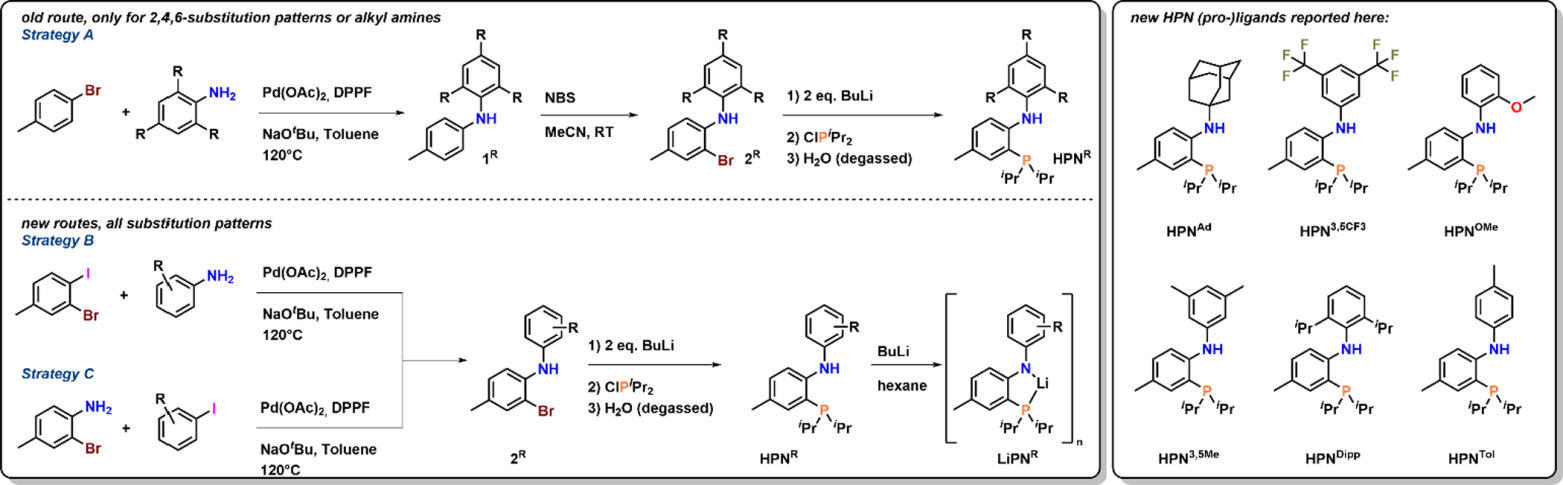
Classical Route (A) to 2,4,6-Trisubstituted Anilidophosphine Ligands (top), New Approaches (B, C) To Enhance Substrate Scope and Modularity of the Anilidophosphine Ligands

Previous routes to such bidentate monoanionic anilidophosphine ligands (**D**, Figure 1) relied on the use of 2,4,6-substituted R-groups (*strategy A*, Scheme 1).^45^ This pattern is mandatory, as in the second step of the classical route (*Strategy A*, Scheme 1) an *ortho*-bromination step is taken, which is usually favored in the *ortho-* and *para*-position of the ground-lying anilines. Thus, the traditional route relies on this “2,4,6-blocking” pattern as otherwise problems with regioselectivity might diminish the yield and/or lead to tedious purification strategies. This strongly limits the modularity of the synthesis, *e.g.*, tuning the steric and electronic properties of the anilidophosphine ligand. Indeed, so far only 2,4,6-trimethyl and 2,4,6-triisopropylphenyl substituents (**HPN**^**Mes**^ and **HPN**^**Tripp**^) have been successfully implemented.^45^ Although **HPN**^**Mes**^ lead to a plethora of interesting findings, such as the isolation of highly basic titanium and zirconium nitrido complexes,^46,47^ the isolation of a titanium arsenido^48^ or methylidene,^49^ the stabilization of a uranium(IV) parent imido complex,^50^ the stabilization of lanthanide [OCP]^−^ complexes,^51^ and the unique η^3^-coordination of the phosphaethynthiolate anion [SCP]^−^,^52^ to name a few, it holds several drawbacks. For example, a typical and undesired side reaction with large electropositive metals is the C–H activation of a mesityl methyl group, leading to cyclometalated complexes. This, among others, resulted in the isolation of a parent hafnium imido complex,^53^ the formation of a parent uranium(IV) imido complex^50^ (both via the formation of transient nitrides), and the isolation of a C–H activated lanthanum phosphanido complex (formed via a transient phosphinidene).^44^ Thus, new strategies in the synthesis of such anilidophosphines are urgently needed, which allow a more modular control of the electronic and steric properties, giving access to alkyl as well as *ortho-*, *meta-*, or *para-*substitution patterns. We recently tried to overcome this limitation by designing a new strategy (s*trategy C*, Scheme 1) which allowed us to install bulky substituents, such as a terphenyl group on the anilidophosphine ligand framework (**HPN**^**Terph**^). This offered severe steric protection and led to the isolation of the first bridged lanthanum phosphinidene complex reported so far.^54^ Here, we further expand the synthetic access to modularly substituted anilidophosphines and present the synthesis of overall six new anilidophosphine ligands. In addition to the synthesis of these new ligands, their coordination behavior toward lanthanum is investigated and different complexation strategies are implemented, leading to an unexpected array of new bis-PN lanthanum complexes.

## Results and Discussion

### Synthesis of HPN^R^ and LiPN^R^

Since bromination in the second step of the classical synthetic route is problematic, the new routes must rely on installing the bromide atom prior to the installation of the *N*-substituent. Circumventing this problem, we found that iodides tend to react first in cross-coupling reactions compared to bromides. Thus, we envisioned two different strategies (Scheme 1). The first strategy (*strategy B*) relies on the use of commercially available 4-iodo-3-bromo-toluene, which is processed in a Buchwald–Hartwig cross-coupling to yield the brominated aniline **2**^**R**^. Alternatively (*strategy C*), *para*-toluidine can be directly brominated in the *ortho*-position using *N*-bromosuccinimide (NBS) and subsequently coupled with aryl iodides to give the desired brominated aniline **2**^**R**^,^54^ which can then be further functionalized via classical halide-lithium exchange and subsequent addition of chlorodiisopropylphosphine to give the desired anilidophosphine ligands **HPN**^**R**^. Both *strategy B* and *strategy C* are successful and can individually be applied, depending on the availability of the corresponding iodo/aniline precursors. Following *strategy B,* we have successfully synthesized **HPN**^**R**^ (R = pTol (**HPN**^**Tol**^), diisopropylphenyl (**HPN**^**Dipp**^), 3,5-bis(trifluoromethyl)phenyl (**HPN**^**3,5CF3**^), and adamantyl (**HPN**^**Ad**^). Notably, **HPN**^**3,5CF3**^ and **HPN**^**Ad**^ can also be accessed using *strategy A*. *Strategy C* was successfully applied to isolate **HPN**^**R**^ where R = 3,5-dimethylphenyl (**HPN**^**3,5Me**^) and 2-methoxyphenyl (**HPN**^**OMe**^). Successful formation of the desired anilinophosphines is given by ^31^P{^1^H} NMR spectroscopy showing the characteristic singlets at −16.6 (**HPN**^**Ad**^, Figure S47), −14.5 (**HPN**^**3,5CF3**^, Figure S54), −14.6 (**HPN**^**OMe**^, Figure S63), −15.5 (**HPN**^**3,5Me**^, Figure S70), −17.8 (**HPN**^**Dipp**^, Figure S77), and −15.9 ppm (**HPN**^**Tol**^, Figure S84). In addition, the ^19^F NMR spectrum of **HPN**^**3,5CF3**^ shows the expected singlet at −62.9 ppm (Figure S56) and the ^1^H NMR spectra of the remaining **HPN**^**R**^ molecules show the expected pattern, *e.g.*, the methoxy resonance at 3.33 ppm for **HPN**^**OMe**^ (Figure S61). Unambiguous proof for the formation of **HPN**^**Dipp**^ was furthermore given by X-ray diffraction analysis on single crystals grown from a concentrated hexane solution (see the SI, Figure S277 (left) and Tables S1 and S3 for more information). Lithium salts of the anilinophosphines **HPN**^**R**^ for transmetalation chemistry can be obtained via their deprotonation in hexane using butyl lithium. This results in the quick precipitation of the desired **LiPN**^**R**^ salts as bright yellow powder. Successful deprotonation is indicated by ^31^P{^1^H} NMR spectroscopy showing the characteristic singlets at −1.7 (**LiPN**^**Ad**^, Figure S91), −7.5 (**LiPN**^**3,5CF3**^, Figure S100), −4.7 (**LiPN**^**OMe**^, Figure S112), −5.1 (**LiPN**^**3,5Me**^, Figure S122), −10.9 (**LiPN**^**Dipp**^, Figure S131), and −7.4 ppm (**LiPN**^**Tol**^, Figure S141). Furthermore, the resonances at 3.2 (**LiPN**^**Ad**^, Figure S94), −0.8 (**LiPN**^**3,5CF3**^, Figure S105), 3.2 (**LiPN**^**OMe**^, Figure S115), 3.7/0.5 (**LiPN**^**3,5Me**^, Figure S125), −0.9 (**LiPN**^**Dipp**^, Figure S134), and 3.0/–1.7 ppm (**LiPN**^**Tol**^, Figure S144) in the ^7^Li NMR of the complexes confirm the presence of lithium in these molecules. Notably, the ^1^H NMR spectra of **LiPN**^**OMe**^ and **LiPN**^**Tol**^ showed unexpectedly broad resonance, indicating some dynamic behavior (Figures S110 and S139). This was overcome by VT ^1^H NMR spectroscopy, giving access to the clean and defined NMR patterns expected (Figures S109 and 138). For clarity, these NMR parameters have also been summarized in Table 1, *vide supra*.

**Figure 2:**
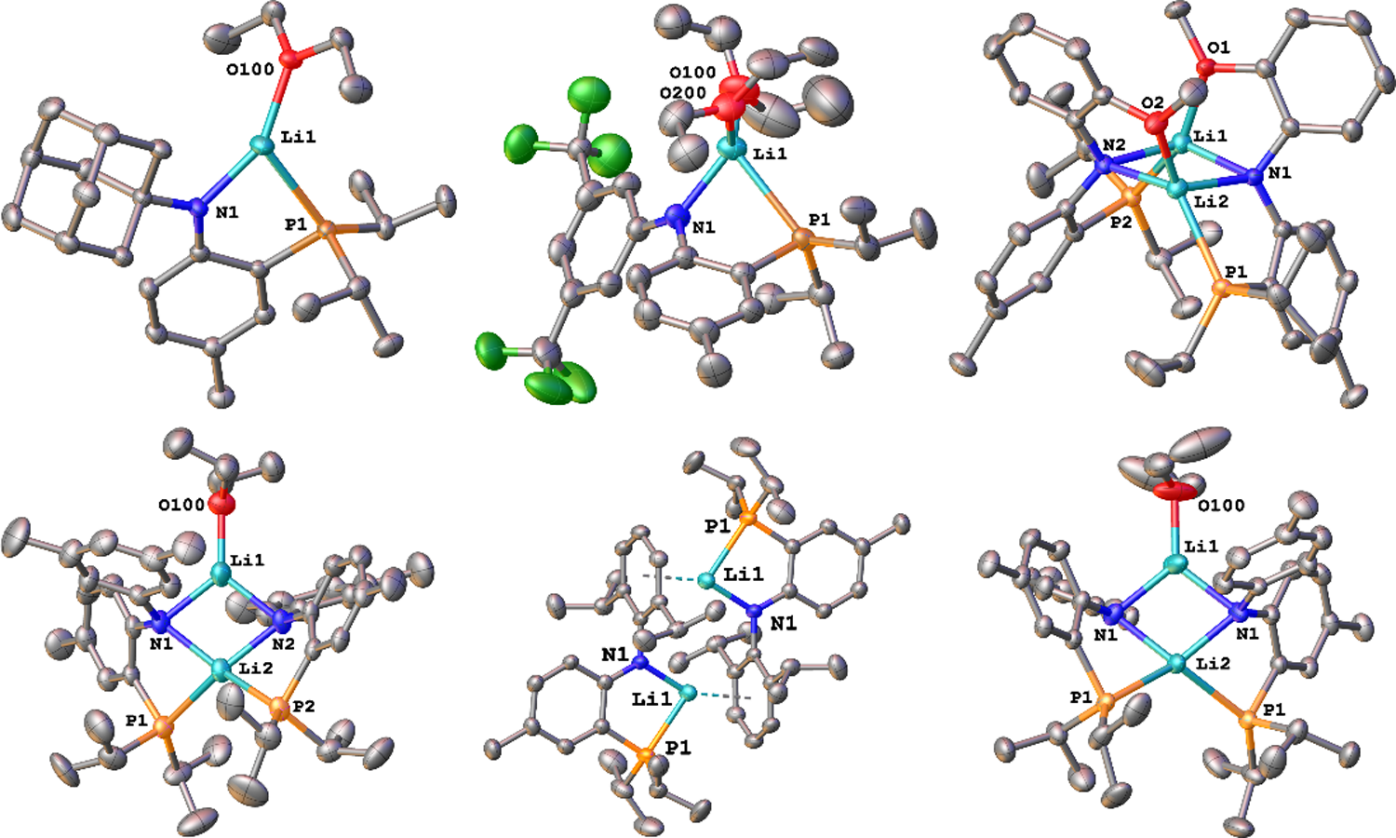
Molecular structures of **LiPN**^**Ad**^, **LiPN**^**3,5CF3**^, and **LiPN**^**OMe**^ (top row, f.l.t.r) and **LiPN**^**3,5Me**^, **LiPN**^**Dipp**^, and **LiPN**^**Tol**^ (bottom row, f.l.t.r). Hydrogen atoms and solvent lattice molecules have been omitted for clarity. Ellipsoids are shown at a probability level of 50%.

**Table 1:** Overview of Characteristic NMR Signals of **HPN**^**R**^ and **LiPN**^**R**^ (in ppm)

	^31^P{^1^H}	^19^F	^7^Li
**HPN**^**Ad**^	–16.6		
**LiPN**^**Ad**^	–1.7		3.2
**HPN**^**3,5CF3**^	–14.5	–62.9	
**LiPN**^**3,5CF3**^	–7.5	–63.2	–0.8
**HPN**^**OMe**^	–14.6		
**LiPN**^**OMe**^	–4.7		3.2
**HPN**^**3,5Me**^	–15.5		
**LiPN**^**3,5Me**^	–5.1		3.7/0.5^a^
**HPN**^**Dipp**^	–17.8		
**LiPN**^**Dipp**^	–10.9		–0.9
**HPN**^**Tol**^	–15.9		
**LiPN**^**Tol**^	–7.4		3.0/–1.7^a^

a The presence of two signals is in line with the solid-state structures of these molecules (*vide infra*), displaying one Li atom being only coordinated by P,N and one which is bound to an additional Et_2_O ligand.

**Figure 3:**
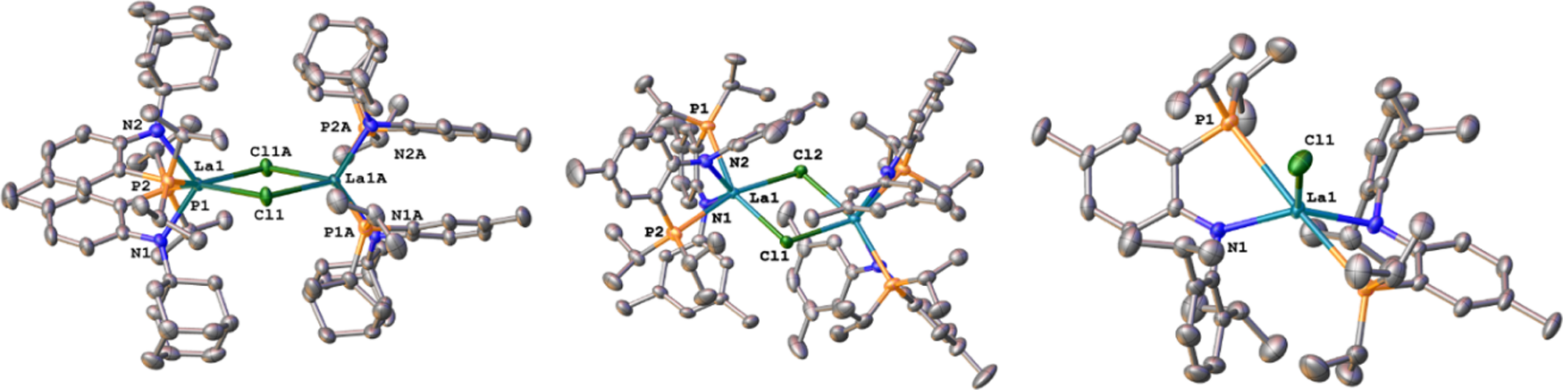
Molecular structures of the bis-PN complexes **3a**, **3d**, and **3e** (f.l.t.r.). Hydrogen atoms and lattice solvent molecules have been omitted for clarity. Ellipsoids are shown at a probability level of 50%.

Unambiguous proof for the successful formation of the desired **LiPN**^**R**^ salts was given by X-ray diffraction studies on single crystals obtained by a concentrated solution of diethyl ether for **LiPN**^**Ad**^, **LiPN**^**3,5CF3**^, **LiPN**^**OMe**^, **LiPN**^**3,5Me**^, and **LiPN**^**Tol**^ (Figure 2). Single crystals for **LiPN**^**Dipp**^ were grown from a concentrated solution of hexane. Depending on the steric bulk of the nitrogen substituent various structural motifs have been observed. **LiPN**^**Ad**^ and **LiPN**^**3,5CF3**^ have both been found to crystallize as monomers with one and two molecules of ether completing the coordination sphere of the lithium center. For larger R-groups such as in **LiPN**^**Dipp**^, we found the formation of a dimeric species, which (similar to previously reported **LiPN**^**Mes**^) dimerizes via Li-Arene interactions.^55^ For medium-sized R-group or groups with coordinating functionalities also dimeric species in which the lithium ions are bridged between the nitrogen donors. Depending on the presence or absence of an additional internal donor, such as in **LiPN**^**OMe**^, the coordination sphere of the lithium ions is either completed by an external diethyl ether molecule or by the internal donor function. Notably, if **LiPN**^**OMe**^ is crystallized from pure diethyl ether, the internal donor functions were shown to be partially displaced by external diethyl ether donors, resulting in the dimeric structure **LiPN**^**OMe**^**·Et**_**2**_**O** (see Figure S277) and Tables S1 and S3 for more information) The Li–P distances are found to range from 2.466(5) Å to 2.509(3) Å with **LiPN**^**Dipp**^ displaying the shortest and **LiPN**^**OMe**^**· Et**_**2**_**O** displaying the longest Li–P interaction. The latter is potentially caused by the stronger Li–O interaction formed with oxophilic metal centers (*vide infra*). Similar trends are observed for the Li–N distances, ranging from 1.959(5) Å in **LiPN**^**Dipp**^ to 2.134(3) Å in **LiPN**^**OMe**^**· Et**_**2**_**O**. Further information on the structural parameters can be found in SI, Tables S1, and S3.

### Complexation Reactions in Toluene

**Scheme 2:**
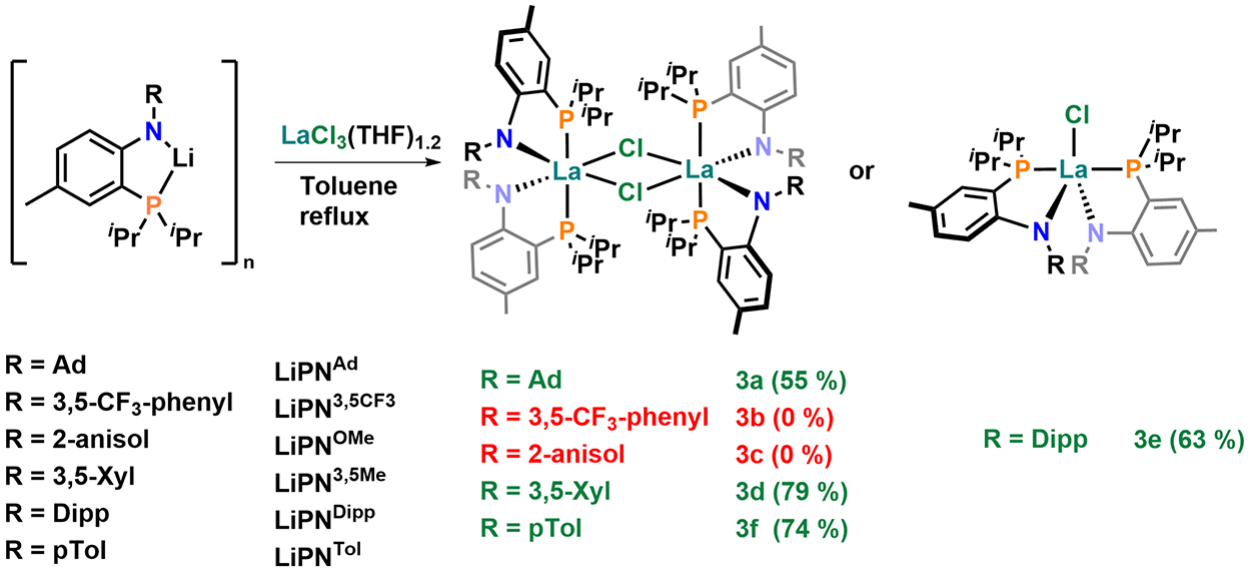
(Attempted) Synthesis of Neutral Bis-PN Lanthanum Complexes via Salt Metathesis in Refluxing Toluene Numbers in brackets refer to isolated yields. Red colours indicate that these combinations did not yield the desired bis-PN complexes.

With the new **LiPN**^**R**^ precursors in hand, we were interested in their coordination behavior toward lanthanum, which revealed unexpected obstacles. While the salt metathesis reaction between two equivalents of **LiPN**^**Ad**^, **LiPN**^**3,5Me**^, **LiPN**^**Dipp**^, and **LiPN**^**Tol**^ with one equivalent of LaCl_3_(THF)_1.2_ in boiling toluene (as previously reported)^55^ gave access to clean single products **3a** and **3d**–**f** as yellow solids in good yields of 55–79% after heating and workup, for **LiPN**^**OMe**^ (proposed **3c**) and **LiPN**^**3,5CF3**^ (proposed **3b**) only messy reactions could be observed and none of the anticipated complexes could be isolated from the reaction mixture (Scheme 2). In the case of the methoxy-substituted ligand **[PN**^**OMe**^**]**^**–**^, we found several indicators that the methoxy group is demethylated and X-ray diffraction analysis on very weakly diffracting crystals, indicated migration of the methyl group onto the phosphorus atom, resulting in the formation of compound **X** (Figure S277). Deprotection/conversion of anisoles to phenols is a common reaction in the presence of strong Lewis acids and lanthanides.^56,57^ In the case of the fluorinated ligand **LiPN**^**3,5CF3**^, the reaction mixture turns dark blue upon mixing and sluggish brown upon heating. The blue color might indicate some radical process, but as stated before, no useful reaction products could be isolated from any reaction between **LiPN**^**3,5CF3**^ and LaCl_3_(THF)_1.2_ in toluene so far. Despite these two drawbacks, successful formation of the desired PN complexes **3a**, **3d**, and **3e** was indicated by ^31^P{^1^H} NMR spectroscopy showing singlet resonances at 16.0 ppm for **3a**, 7.5 ppm for **3d**, 8.9 ppm for **3e**, and 6.5 ppm for **3f** (compare Table 2). Unambiguous proof for the successful formation of bis-PN complexes was delivered by X-ray diffraction analysis performed on single crystals grown from a concentrated hexane solution for **3a**, **3e**, and a concentrated solution of diethyl ether for **3d** (Figure 3). The complexes crystallize in the triclinic, orthorhombic, and monoclinic space groups *P*–1, *Pbcn,* and *P*2_1_/*c* for **3a**, **3d**, and **3e**, respectively, showing severe structural differences. While only the Dipp-substituted complex **3e** was found to be monomeric in the crystalline state, with a pentafold coordinated lanthanum ion in a distorted trigonal bipyramidal environment (τ_5_ = 0.70; compare 0.17 for **(PN**^**Terph**^**)**_**2**_**LaCl**(54) and 0.87 for **(PN**^**Mes**^**)**_**2**_**LaCl**(55)), the complexes **3a** and **3d** were found to form chloride bridged dimers, indicating that even the steric bulk of the **PN**^**Ad**^ ligand (*vide infra*) is not sufficient to suppress dimerization in the solid state (and arguably also not in solution). However, comparing the structures of **3a** and **3d**, it becomes evident that the lanthanum ion in **3a** is strongly shifted out of the PN ligand plane by 1.834(1)/1.896(1) Å (compared to 0.659(1)/0.379(1) Å in **3d**). Additionally, the ligand metal plane (defined by P–La–N) is tilted by 54.52(1)/57.93(1)° *vs*. the ligand plane defined by the PCCN atoms (16.567(1)/9.424(1)° in **3d**) which indicates severe steric pressure in this system. The La1–N1/La1–N2 distances were found to be 2.3908(19)/2.371(2) Å in **3a**, 2.414(3)/2.429(3) Å in **3d**, and 2.399(2) Å in **3e**, while the La1–P2/La1-P2 distances are 3.0696(6)/3.0657(6) Å in **3a**, 3.1898/3.2085(11) Å in **3d**, and 3.2132(3) Å in **3e**. Given the diverse values of the La–N distances, no direct correlation toward the donor strength of the anilido donor (*N*-substitution pattern) can be drawn and the distance is most likely governed by structural/steric rather than electronic factors. In addition, we found that if for example **3d** is crystallized from coordinating solvents such as DME; the dimeric nature is broken and a 7-fold coordinated lanthanum ion is observed (Figure S277). This results in a severe elongation of the metal–ligand bonds to 2.460(3)/2.468(3) Å for La1–N1/La1–N2 and 3.1914(11)/3.2064(11) Å for La1–P1/La1–P2.

**Table 2:** Overview of Characteristic NMR Signals of the Neutral Complexes **3** and the Anionic-ate Complexes **4** (in ppm)

neutral complexes **3**		-*ate* complexes **4**		
	^31^P{^1^H}		^31^P{^1^H}	^7^Li
**3a**	16.0	**4a**	8.9	0.6
**3b**		**4b**	10.2	–0.1
**3c**		**4c**	5.8	0.9
**3d**	7.5	**4d**	7.8	0.0
**3e**	8.9	**4e**		
**3f**	6.5	**3f**	8.4	0.1

**Scheme 3:**
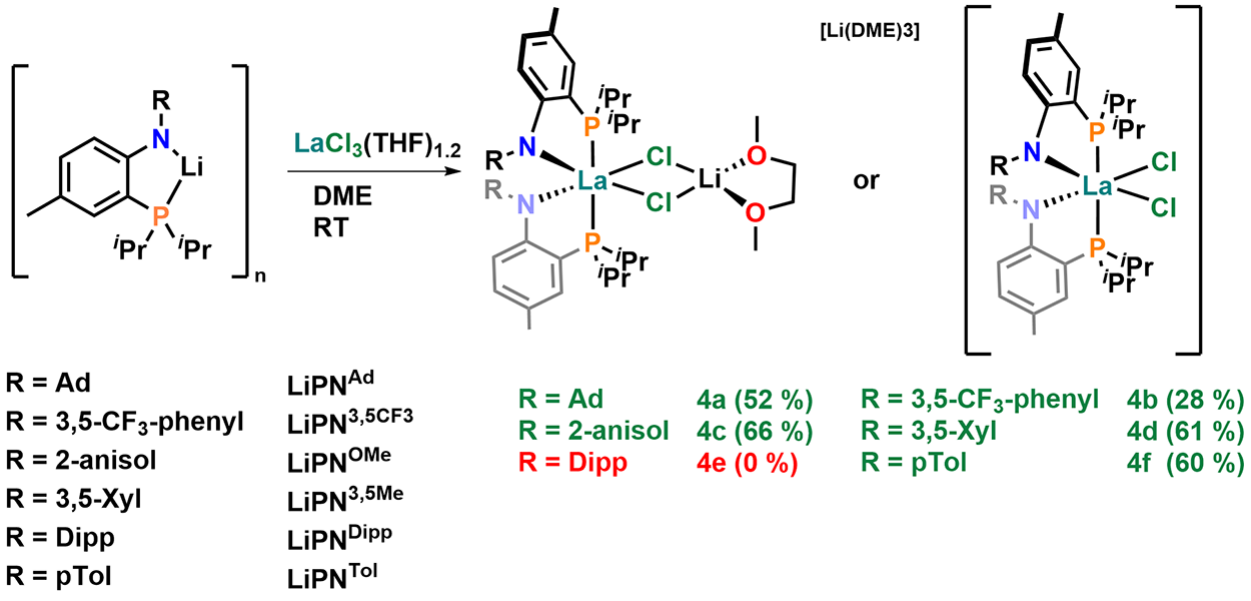
(Attempted) Synthesis of Anionic Bis-PN Lanthanate Complexes via Salt Metathesis in DME at Room Temperature Numbers in brackets refer to isolated yields. Red colours indicate that these combinations did not yield the desired bis-PN complexes.

### Complexation Reactions in DME

**Figure 4:**
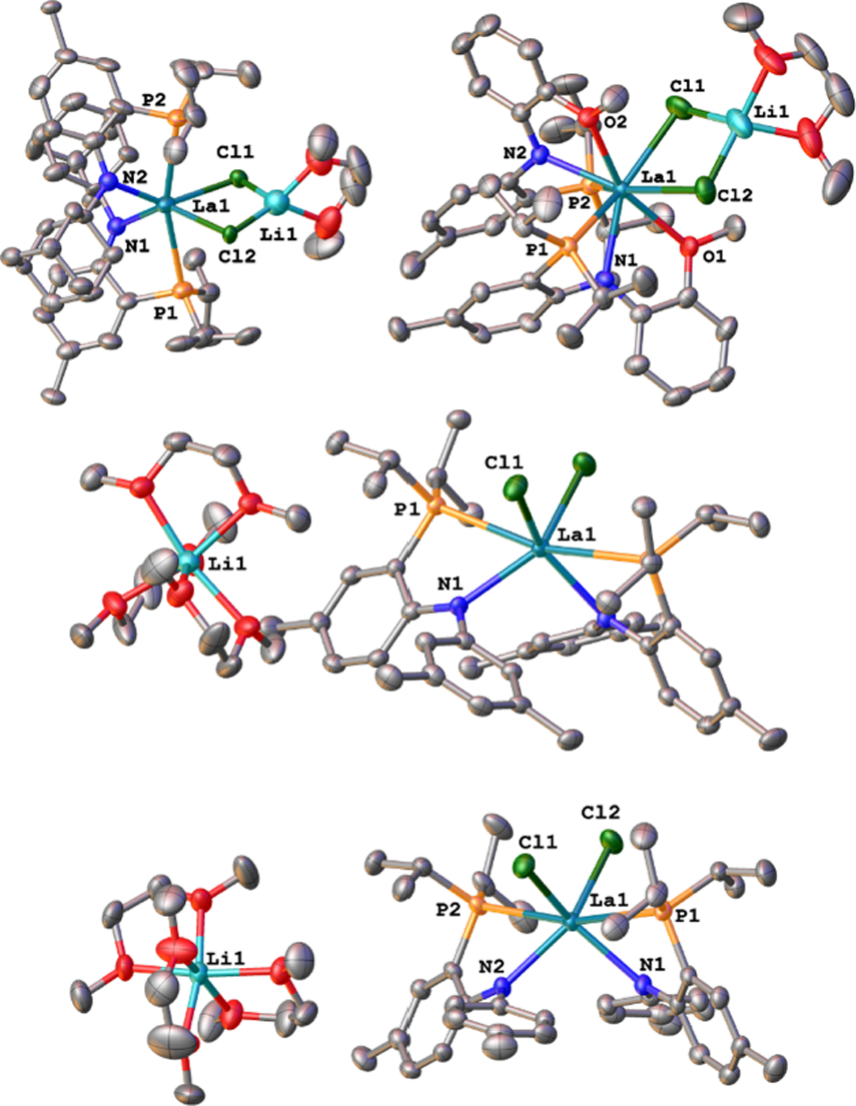
Molecular structures of the *–ate* complexes **4a** (top, left), **4c** (top, right), **4d** (middle), and **4f** (bottom). Hydrogen atoms and lattice solvent molecules have been omitted for clarity. Ellipsoids are shown at a probability level of 50%.

Given the fact that in boiling toluene, **LiPN**^**OMe**^ and **LiPN**^**3,5CF3**^ could not be transmetalated with LaCl_3_(THF), we further sought other/milder strategies to install these PN ligands on lanthanum. Gratifyingly, room temperature reactions between two equivalents of **LiPN**^**R**^ and one equivalent of LaCl_3_(THF)_1.2_ in DME yielded the *–ate* complexes **4a**–**d** and **4f** in moderate to good yields between 28 and 66% (Scheme 3). Notably, the transmetalation with **LiPN**^**Dipp**^ fails under these conditions indicating that its bulkyness prevents the formation of a putative *–ate* complex **4e**. Instead, we find the formation of at least three different products of which, one can be identified as complex **3e** by ^31^P{^1^H} NMR spectroscopy. Formation of the *–ate* complexes is indicated by several spectroscopic features: First of all, the ^31^P{^1^H} NMR resonances of all synthesized complexes are different to the ones observed, if transmetalation is performed in toluene (compare Table 2) and second for all complexes a defined resonance in the ^7^Li NMR is observed at 0.6 ppm for **4a**, −0.1 ppm for **4b**, 0.9 ppm for **4c**, 0.0 ppm for **4d**, and 0.1 ppm for **4f** respectively. Despite being relatively small, the difference between signals centered around 1 and 0 ppm already indicates the presence of two structural motifs in solution (*vide infra*). Indeed, X-ray diffraction analysis on crystals grown from a concentrated solution of hexane for **4a**, **4c**, **4d**, and **4f** shows, that the complexes exist either as contact ion pairs, with the lithium ion being centered between the chloride ligands, or as solvent-separated ion pairs with distinct [Li(DME)_3_]^+^ units and the corresponding lanthanate-centered counterions [(PN^R^)_2_LaCl_2_]^−^ (Figure 4). The structures that display no contact between the anionic *–ate* complex and the lithium ions display ^7^Li NMR shifts of 0.0 ppm for **4d** and 0.1 ppm for **4f**, while the ion contact pairs show ^7^Li NMR values at 0.6 ppm for **4a** and 0.9 ppm for **4c**. This indicates that, despite no suitable crystals could be grown for **4b** (^7^Li NMR shift at −0.1 ppm), it adopts a structure similar to **4d** and **4f** with full ion pair separation. The lithium incorporation or excorporation also results in a major structural difference regarding the halide ions. While for the contact ion pair complexes, the La–Cl distances were found at 2.854(5)/2.934(4) Å in **4a** and 2.8560(9)/2.9192(9) Å in **4c**, these distances are much shorter at 2.7476(9) Å in **4d** and 2.7265(10)/2.7449(10) Å in **4f**. Similarly the Cl1–La1–Cl2 angles increase from 80.10(12) and 76.15(3) ° in **4a** and **4c** to 96.94(4) and 98.76(4) ° in **4d** and **4f**. A similar extension of the La1–Cl1/2 distances is also seen in the dimeric complexes **3a** and **3d**, alongside a more acute Cl1–La1–Cl2 angle (see Tables S1–S4 for more information). The La–N distances are 2.476(15)/2.463(14) Å in **4a**, 2.468(2)/2.452(2) Å in **4c**, 2.473(2) Å in **4d**, and 2.482(3)/2.476(3) Å in **4f**, while the La–P distances are 3.149(5)/3.127(5) Å, 3.1890(8)/3.1947(9) Å, 3.1300(8) Å, and 3.1218(10)/3.1660(10) Å in **4a**,**c**,**d**,**f**, respectively. Notably, the “tridenate” methoxy-substituted ligand in **4c** shows a strong interaction between the lanthanum center and the oxygen atom of the methoxy group (La1–O1 and La1–O2 display distances of 2.658(2) Å and 2.632(2) Å), which results in a slight elongation of the La–N and the La–P bonds. Overall, the coordination motif in this complex can be better described as ON or ON(P) coordination instead of pure PN coordination .

### Steric Properties of the Bis-PN Complexes

**Figure 5:**
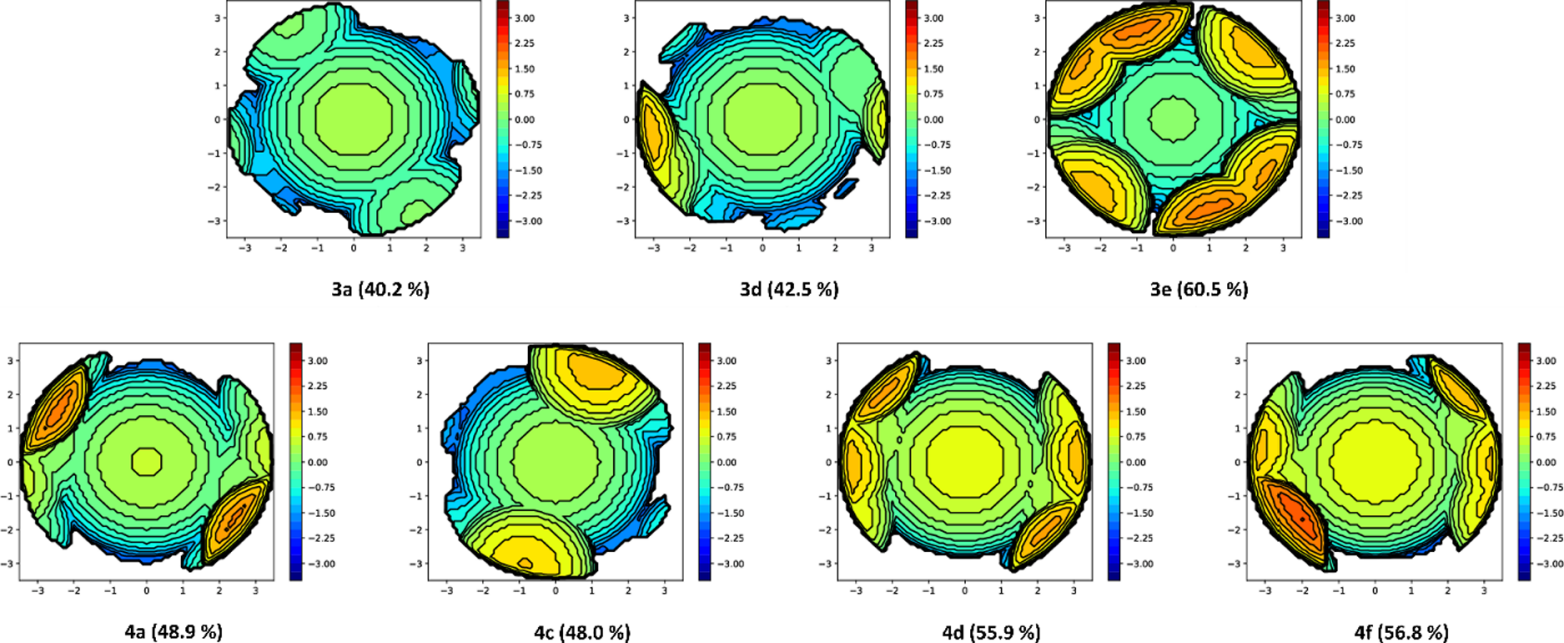
%V_buried_ determined by using the SambVca software suite. For bridged systems, as well as the −*ate* complexes, dummy atoms have been placed between the two chloride atoms, defining the *Z*-axis as a straight line through the chloride atom (in **3e**) or the dummy atoms in all other structures displayed.

To seek potential reasons for the different coordination and structural motifs within the series of complexes **3** and **4**, we further examined the steric bulk of the coligands in their lithium salts (see SI, Tables S5–S7), their neutral complexes, and their *–ate* complexes (Figure 5 and Tables S8–S11).^58,59^ This showed that no clear correlation between the steric bulk of the nitrogen substituent and the structural motifs observed can be drawn. For example, in the *–ate* complexes **4**, the p-tolyl-substituted complex **4f** seems to have a higher steric bulk, compared to 3,5-xylyl substituted complex **4d**. However, upon close examination of the solid-state structure, it becomes obvious that in both cases, the P^*i*^Pr_2_ substituents are orientated “upwards”, while the *N*-substituent point away from the reference point of the SambVca calculations, diminishing the effect of the sterically different *N*-substituents. Despite these orientation effects, some qualitative statements can be drawn from the series. For example, the anisole-substituted ligand **PN**^**OMe**^ seems to offer the least shielding effect followed by the *p*-tolyl and the adamantyl-substituted systems **PN**^**Tol**^ and **PN**^**Ad**^. Contrary, the Dipp-substituted ligand **PN**^**Dipp**^ offers the highest steric protection with a buried volume of 60.5% around the metal center in **3e**. Thus, **PN**^**Dipp**^ was found to offer a steric protection lying in between the ubiquitous **PN**^**Mes**^ ligand (%*V*_buried_ = 47.5%) and the recently reported **PN**^**Terph**^ system (%*V*_buried_ = 69.9%).^54^ Although the **PN**^**Dipp**^ ligand does not completely lack vulnerable protons on the *N*-substituent, the methine protons of the Dipp group are much less reactive compared to the mesityl-methyl protons. The combination of the high steric bulk in combination with the absence of easily activatable C–H groups renders the **PN**^**Dipp**^ ligand as a potential candidate to stabilize highly reactive lanthanide complexes, not accessible using **PN^Mes^**

## Conclusions

We have extended the methodologies to synthesize bidentate, monoanionic anilidophosphine ligands, circumventing several problems in the original synthetic protocol (*e.g.*, regioselective synthesis and substitution scope/patterns) and studied their application in the coordination chemistry of lanthanum. Thereby, we found that complexation is either successful in boiling toluene, giving access to neutral mono- and dinuclear complexes, while complexation in coordinating solvents such as DME results in the formation of different *–ate* complexes being either solvent-separated or contact ion pairs. The latter can be distinguished by ^7^Li NMR spectroscopy making it a useful tool if no X-ray crystals can be obtained for such complexes. Analysis of the steric bulk revealed, that the newly synthesized Dipp-substituted ligand **PN**^**Dipp**^ offers a well-defined steric profile around the lanthanum center with a %*V*_buried_ of 60.5% lying in between the ubiquitous **PN**^**Mes**^ and the recently reported **PN**^**Terph**^ system. This renders the ligand a potential candidate for the synthesis of highly reactive lanthanum complexes. First efforts in this direction will be reported shortly.

## Experimental Section

### General Considerations

Unless otherwise stated, all operations were performed in a glovebox under an atmosphere of purified argon or using high-vacuum standard Schlenk techniques under an argon atmosphere. All glassware (including glass-fiber filters) was stored in an oven at 140 °C for at least 12 h prior to use. Anhydrous ^*n*^hexane, ^*n*^pentane, toluene, and diethyl ether were purchased from Aldrich in Sure-Seal reservoirs (18L) and were dried using an SPS and stored over 3 Å molecular sieves at least 3 days prior to use. THF and DME were distilled, under argon, from purple sodium benzophenone ketyl and stored with 3 Å molecular sieves. Celite and 3 Å molecular sieves were activated/dried under vacuum overnight at 250 °C. *N*-(*p*-tolyl)-adamantan-1-amine (**1**^**Ad**^),^60^*N*-(*p*-tolyl)-3,5-bis(trifluoromethyl)aniline (**1**^**3,5CF3**^),^60^ 2-bromo-*N*-(3,5-dimethylphenyl)-4-methylaniline (**2**^**3,5Me**^),^61^ and 2-bromo-4-methyl-*N*-(*p*-tolyl)aniline (**2**^**Tol**^)^62^ were synthesized through a slightly modified pathway to the literature. All other chemicals were used as received unless otherwise stated. CDCl_3_ was used as received and C_6_D_6_ was stored and dried over freshly activated 3 Å molecular sieves.^1^H, ^7^Li, ^13^C, ^19^F, and ^31^P NMR spectra were recorded on 400 MHz Bruker Avance 4 Neo spectrometer using J-Young tubes. ^1^H and ^13^C NMR chemical shifts are reported with reference to residual solvent resonances of C_6_D_6_ at 7.16 and 128.06 ppm and for CDCl_3_ at 7.26 and 77.16 ppm, respectively. ^31^P NMR chemical shifts are reported with respect to external H_3_PO_4_ (aqueous solution, delta 0.0 ppm). Elemental analysis was performed using an Elementar vario microcube instrument. Mass spectrometry experiments were performed on a Thermo Finnigan Q Exactive Orbitrap spectrometer. IR spectra were measured neat at room temperature on an IR Bruker Alpha ATR spectrometer. All UV/vis/NIR spectra were collected on an Avantes spectrometer equipped with a deuterium and halogen light source and a CMOS detector. **Caution!***Large quantities of n-BuLi are potentially pyrophoric and should be handled using proper needle and syringe techniques. Also, chlorodiisopropylphosphine has a very pungent smell and should only be used in well-ventilated fume hoods.*

### Synthetic Procedures

#### General Procedure for the Preparation of **1**^**Ad**^ and **1**^**3,5CF3**^

In a 100-mL Schlenk flask, Pd(dba)_2_ (2 mol %), SPhos (4 mol %), and sodium *tert*-butoxide (1.2 equiv) were combined and suspended in toluene (50 mL). A substituted bromobenzene (1.0 equiv) and an excess of aniline (1.1 equiv) were added to the reaction mixture and heated to 120 °C for 24 h. On the next day, the mixture was allowed to cool down to room temperature and filtered through Celite. All volatile components were removed under reduced pressure and redissolved in diethyl ether (100 mL). The ethereal phase was washed twice with water (50 mL) and once with brine (50 mL). The organic phase was dried with anhydrous sodium sulfate, filtered, and the solvent was removed under reduced pressure. The residue was redissolved in hexane and filtered through a silica pad (4 cm), and all volatile components were removed under reduced pressure to observe an analytically clean product.

##### Preparation of **1^Ad^**

From Pd(dba)_2_ (0.02 equiv, 0.60 mmol, 346 mg), SPhos (0.04 equiv, 1.20 mmol, 494 mg), sodium *tert*-butoxide (1.2 equiv, 36.06 mmol, 3.47 g), 1-bromo-4-methylbenzene (1.0 equiv, 30.06 mmol, 5.14 g), and 1-adamantylamine (1.1 equiv, 33.06 mmol, 5.00 g). 7.14 g (98.4%) of an orange solid. ^1^H NMR (400 MHz, C_6_D_6_, in ppm) δ = 6.99 (d, *J* = 8.0 Hz, 2H, C*H*_Ar_), 6.73 (d, *J* = 8.4 Hz, 2H, C*H*_Ar_), 2.87 (br. s, 1H, N*H*), 2.20 (s, 3H, C*H*_*3*_), 1.92 (s, 3H, C*H*_Ad_), 1.74 (d, *J* = 2.8 Hz, 6H, C*H*_2,Ad_), 1.51 (q, *J* = 12.1 Hz, 6H, C*H*_2,Ad_); ^13^C{^1^H} NMR (101 MHz, C_6_D_6_, in ppm) δ = 143.8 (*C*_Ar_), 129.2 (*C*H_Ar_), 128.5 (*C*_Ar_), 120.6 (*C*H_Ar_), 51.8 (*C*_Ad_), 43.6 (*C*H_2,Ad_), 36.4 (*C*H_2,Ad_), 29.8 (*C*H_Ad_), 20.4 (*C*H_3_).

##### Preparation of **1^3,5CF3^**

Pd(dba)_2_ (0.02 equiv, 0.34 mmol, 196 mg), SPhos (0.04 equiv, 0.68 mmol, 280 mg), sodium *tert*-butoxide (1.2 equiv, 20.48 mmol, 1.97 g), 1-bromo-3,5-bis(trifluoromethyl)benzene (1.0 equiv, 17.06 mmol, 5.00 g), and *p*-toluidine (1.1 equiv, 18.77 mmol, 2.01 g). Yield: 4.55 g (83.5%) of a white crystalline solid. ^1^H NMR (400 MHz, C_6_D_6_, in ppm) δ = 7.28 (s, 1H, C*H*_Ar_), 6.90 (s, 2H, C*H*_Ar_), 6.86 (d, *J* = 8.1 Hz, 2H, C*H*_Ar_), 6.69 (d, *J* = 8.3 Hz, 2H, C*H*_Ar_), 4.75 (s, 1H, N*H*), 2.08 (s, 3H, C*H*_*3*_); ^13^C{^1^H} NMR (101 MHz, C_6_D_6_, in ppm) δ = 146.4 (*C*_Ar_), 137.7 (*C*_Ar_), 133.9 (*C*_Ar_), 132.8 (q, *J* = 32.8 Hz, *C*F_3_), 130.5 (C*H*_Ar_), 121.6 (*C*H_Ar_), 114.5 (*C*H_Ar_), 112.1 (p, *C*H_Ar_), 20.8 (*C*H_3_); ^19^F NMR (377 MHz, C_6_D_6_, in ppm) δ = −62.95 (s, 6F); ^19^F{^1^H} NMR (377 MHz, C_6_D_6_, in ppm) δ = −62.95 (s, 6F).

#### General Procedure for the Preparation of **2**^**Ad**^ and **2**^**3,5CF3**^

Compound **1**^**R**^ (1.0 equiv) was dissolved in acetonitrile (300 mL) and cooled to 0 °C. Solid NBS (1.05 equiv) was added in small portions over a timeperiod of 2 h. The reaction mixture was stirred overnight and was allowed to warm up to room temperature. On the next day, all volatile components were removed under reduced pressure and redissolved in diethyl ether (100 mL). The organic phase was washed twice with water (50 mL) and once with brine (50 mL). The ethereal phase was dried over anhydrous sodium sulfate, and the solvent was removed under reduced pressure. The crude product was redissolved in hexane and filtered through a silica pad (4 cm), and all volatile components were removed under reduced pressure to observe an analytically clean product.

##### Preparation of **2^Ad^**

From **1**^**Ad**^ (1.0 equiv, 24.03 mmol, 5.80 g) and NBS (1.05 equiv, 25.23 mmol, 4.49 g). 6.06 g (78.7%) of a white solid. ^1^H NMR (400 MHz, C_6_D_6_, in ppm) δ = 7.27 (d, *J* = 1.5 Hz, 1H, C*H*_Ar_), 6.99 (d, *J* = 8.3 Hz, 1H, C*H*_Ar_), 6.85–6.79 (m, 1H, C*H*_Ar_), 4.15 (s, 1H, N*H*), 1.98 (s, 3H, C*H*_*3*_), 1.88 (s, 3H, C*H*_Ad_), 1.83 (d, *J* = 2.8 Hz, 6H, C*H*_2,Ad_), 1.55–1.43 (m, 6H, C*H*_2,Ad_); ^13^C{^1^H} NMR (101 MHz, C_6_D_6_, in ppm) δ = 141.9 (*C*_Ar_), 133.5 (*C*H_Ar_), 128.7 (*C*H_Ar_), 118.1 (*C*H_Ar_), 114.1 (*C*_Ar_), 52.8 (*C*_Ad_), 43.5 (*C*H_2,Ad_), 36.6 (*C*H_2,Ad_), 30.1 (*C*H_Ad_), 20.0 (*C*H_3_). Hi–Res Mass (ESI^+^) calcd for [C_17_H_23_BrN]^+^: 320.1008; found: 320.1008.

##### Preparation of **2^3,5CF3^**

From **1**^**3,5CF3**^ (1.0 equiv, 9.40 mmol, 3.00 g) and NBS (1.05 equiv, 9.87 mmol, 1.76 g). 3.60 g (96.2%) of a white solid. ^1^H NMR (400 MHz, C_6_D_6_, in ppm) δ = 7.31 (s, 1H, C*H*_Ar_), 7.16 (s, 1H, C*H*_Ar_), 6.87 (s, 2H, C*H*_Ar_), 6.75 (d, *J* = 8.1 Hz, 1H, C*H*_Ar_), 6.53 (dd, *J* = 8.2, 1.7 Hz, 1H, C*H*_Ar_), 5.35 (s, 1H, N*H*), 1.86 (s, 3H, C*H*_*3*_); ^13^C{^1^H} NMR (101 MHz, C_6_D_6_, in ppm) δ = 145.1 (*C*_Ar_), 136.3 (*C*_Ar_), 135.1 (*C*_Ar_), 134.2 (*C*H_Ar_), 132.9 (q, *J* = 33.0 Hz, *C*F_3_), 129.3 (*C*H_Ar_), 123.9 (d, *J* = 272.9 Hz, *C*(CF_3_)), 120.8 (*C*H_Ar_), 116.5 (*C*_Ar_), 116.1 (*C*H_Ar_), 113.5 (hept, *J* = 3.9 Hz, *C*H_Ar_), 20.2 (*C*H_3_); ^19^F NMR (377 MHz, C_6_D_6_, in ppm) δ = −62.86 (s, 6F); ^19^F{^1^H} NMR (377 MHz, C_6_D_6_, in ppm) δ = −62.86 (s, 6F). Hi–Res Mass (ESI^+^) calcd for [C_15_H_11_BrF_6_N]^+^: 397.9974; found: 397.9978.

#### General Procedure for the Preparation of **2**^**OMe**^**, 2**^**3,5Me**^, **2**^**Dipp**^, and **2**^**Tol**^

In a 100-mL Schlenk flask, palladium(II) acetate (2–5 mol %), dppf (4–10 mol %), and sodium *tert*-butoxide (1.2 equiv) were suspended in toluene (50 mL). Afterward, a substituted benzene iodide (1.0 equiv) and an excess of aniline (1.1–2.0 equiv) were added to the reaction mixture and stirred at 120 °C for 2–5 days. The suspension was filtered through a pad of Celite and the solvent was removed. The residue was redissolved in diethyl ether (100 mL) and washed twice with water (50 mL) and once with brine (50 mL). All volatile components were removed under reduced pressure and redissolved in hexane. The organic solution dried over anhydrous sodium sulfate and filtered through a medium porosity frit. Finally, the crude product was filtered through a silica pad (4 cm) and all volatile components of the filtrate were removed under reduced pressure to observe the desired compounds.

##### Preparation of **2^OMe^**

From palladium(II) acetate (0.02 equiv, 0.43 mmol, 96 mg), dppf (0.04 equiv, 0.86 mmol, 474 mg), sodium *tert*-butoxide (1.2 equiv, 25.64 mmol, 2.46 g), 2-iodoanisol (1.0 equiv, 21.36 mmol, 5.00 g), and 2-bromo-4-methylaniline (1.1 equiv, 23.5 mmol, 4.37 g). 3.44 g (55.1%) of a yellow oil. ^1^H NMR (400 MHz, C_6_D_6_, in ppm) δ = 7.27 (m, 1H, C*H*_Ar_), 7.26–7.23 (m, 2H, C*H*_Ar_), 6.81 (dd, *J* = 5.9, 3.6 Hz, 2H, C*H*_Ar_), 6.70 (dd, *J* = 8.4, 1.9 Hz, 1H, C*H*_Ar_), 6.68 (s, 1H, N*H*), 6.58–6.53 (m, 1H, C*H*_Ar_), 3.24 (s, 3H, OC*H*_*3*_), 1.92 (s, 3H, C*H*_*3*_); ^13^C{^1^H} NMR (101 MHz, C_6_D_6_, in ppm) δ = 149.6 (*C*_Ar_), 138.9 (*C*_Ar_), 133.8 (*C*H_Ar_), 132.6 (*C*_Ar_), 131.4 (*C*_Ar_), 129.0 (*C*H_Ar_), 121.2 (*C*H_Ar_), 117.6 (*C*_Ar_), 116.5 (*C*H_Ar_), 114.3 (*C*_Ar_), 111.1 (*C*H_Ar_), 55.2 (O*C*H_3_), 20.2 (*C*H_3_). Hi–Res Mass (ESI^+^) calcd for [C_14_H_15_BrNO]^+^: 292.0332; found: 292.0330.

##### Preparation of **2^3,5Me^**

From palladium(II) acetate (0.02 equiv, 0.43 mmol, 97 mg), dppf (0.04 equiv, 0.86 mmol, 478 mg), sodium *tert*-butoxide (1.2 equiv, 25.86 mmol, 2.49 g), 1-iodo-3,5-dimethylbenzene (1.0 equiv, 21.55 mmol, 5.00 g), and 2-bromo-4-methylaniline (1.3 equiv, 28.01 mmol, 5.21 g). 5.78 g (92.4%) of a yellow oil. ^1^H NMR (400 MHz, CDCl_3_, in ppm) δ = 7.38 (s, 1H, C*H*_Ar_), 7.22 (d, *J* = 8.3 Hz, 1H, C*H*_Ar_), 7.01 (dd, *J* = 8.3, 2.0 Hz, 1H, C*H*_Ar_), 6.76 (s, 2H, C*H*_Ar_), 6.67 (s, 1H, C*H*_Ar_), 5.74 (br. s, 1H, N*H*), 2.31 (s, 6H, C*H*_*3*_), 2.30 (s, 3H, C*H*_*3*_); ^13^C{^1^H} NMR (101 MHz, CDCl_3_, in ppm) δ = 142.4 (*C*_Ar_), 139.2 (*C*_Ar_), 138.9 (*C*_Ar_), 133.3 (*C*H_Ar_), 131.1 (*C*H_Ar_), 128.8 (*C*H_Ar_), 123.9 (*C*H_Ar_), 117.2 (*C*H_Ar_), 117.0 (*C*H_Ar_), 112.9 (*C*_Ar_), 21.5 (*C*H_3Ar_), 20.4 (*C*H_3Ar_). Hi–Res Mass (ESI^+^) calcd for [C_15_H_17_BrN]^+^: 290.0539; found: 290.0538.

##### Preparation of **2^Dipp^**

From palladium(II) acetate (0.05 equiv, 0.84 mmol, 189 mg), dppf (0.10 equiv, 1.68 mmol, 933 mg), sodium *tert*-butoxide (1.2 equiv, 20.21 mmol, 1.94 g), 2,6-diisopropylaniline (2.0 equiv, 33.68 mmol, 5.97 g), and 2-bromo-1-iodo-4-methylbenzene (1.0 equiv, 16.84 mmol, 5.00 g). 4.21 g (72.2%) of a yellow oil. *Caution:* If the silica pad was too small, a second silica pad is necessary to remove the excess of aniline. ^1^H NMR (400 MHz, CDCl_3_, in ppm) δ = 7.36–7.27 (m, 2H, C*H*_Ar_), 7.23–7.18 (m, 2H, C*H*_Ar_), 6.79 (dd, *J* = 8.3, 1.4 Hz, 1H, C*H*_Ar_), 6.04 (d, *J* = 8.3 Hz, 1H, C*H*_Ar_), 5.51 (br. s, 1H, N*H*), 3.08 (hept, *J* = 6.9 Hz, 2H, C*H*(CH_3_)_2_), 2.20 (s, 3H, C*H*_*3*_), 1.19 (d, *J* = 6.9 Hz, 6H, CH(C*H*_*3*_)_2_), 1.12 (d, *J* = 6.9 Hz, 6H, CH(C*H*_*3*_)_2_); ^13^C{^1^H} NMR (101 MHz, CDCl_3_, in ppm) δ = 147.8 (*C*_Ar_), 142.8 (*C*_Ar_), 135.2 (*C*_Ar_), 132.8 (*C*H_Ar_), 129.0 (*C*H_Ar_), 127.9 (*C*H_Ar_), 127.7 (*C*H_Ar_), 124.0 (*C*H_Ar_), 112.7 (*C*H_Ar_), 108.8 (*C*_Ar_), 28.5 (*C*H(CH_3_)_2_), 24.8 (CH(*C*H_3_)_2_), 23.2 (CH(*C*H_3_)_2_), 20.2 (*C*H_3_). Hi–Res Mass (ESI^+^) calcd for [C_19_H_25_BrN]^+^: 346.1165; found: 346.1163.

##### Preparation of **2^Tol^**

From palladium(II) acetate (0.02 equiv, 0.47 mmol, 106 mg), dppf (0.04 equiv, 0.94 mmol, 523 mg), sodium *tert*-butoxide (1.2 equiv, 28.29 mmol, 2.72 g), *p*-toluidine (1.1 equiv, 25.93 mmol, 2.78 g), and 2-bromo-1-iodo-4-methylbenzene (1.0 equiv, 23.57 mmol, 7.00 g). 6.18 g (94.9%) of a colorless oil. ^1^H NMR (400 MHz, CDCl_3_, in ppm) δ = 7.37 (d, *J* = 2.0 Hz, 1H, C*H*_Ar_), 7.14 (d, *J* = 8.2 Hz, 2H, C*H*_Ar_), 7.11 (d, *J* = 8.3 Hz, 1H, C*H*_Ar_), 7.04 (d, *J* = 8.4 Hz, 2H, C*H*_Ar_), 6.97 (dd, *J* = 8.5, 2.0 Hz, 1H, C*H*_Ar_), 5.76 (s, 1H, N*H*), 2.34 (s, 3H, C*H*_*3*_), 2.28 (s, 3H, C*H*_*3*_). ^13^C{^1^H} NMR (101 MHz, CDCl_3_, in ppm) δ = 139.6 (*C*_Ar_), 139.5 (*C*_Ar_), 133.2 (*C*H_Ar_), 132.1 (*C*_Ar_), 130.5 (*C*_Ar_), 130.0 (*C*H_Ar_), 128.8 (*C*H_Ar_), 120.4 (*C*H_Ar_), 116.0 (*C*H_Ar_), 112.1 (*C*_Ar_), 20.8 (*C*H_3_), 20.3 (*C*H_3_). Hi–Res Mass (ESI^+^) calcd for [C_14_H_15_BrN]^+^: 276.0382; found: 276.0381.

#### General Procedure for the Preparation of **HPN**^**R**^

Compound **2**^**R**^ (1.0 equiv) was dissolved in diethyl ether (50 mL) and cooled to −78 °C, and a 2.5 M ^*n*^BuLi solution in hexane (2.05 equiv) was added carefully via syringe, and a color change to yellow was observed. After complete addition, the reaction mixture was allowed to warm up to room temperature and stirred for 2–24 h depending on the substrate. The reaction mixture was again cooled to –78 °C and chlorodiisopropylphosphine (1.1 equiv) was added slowly to the mixture. The resulting suspension was stirred for 48 h at room temperature. Degassed water (5 mL) was added to the reaction and stirred for further 30 min. The organic phase was washed twice with water (50 mL) and once with brine (50 mL). Afterward, the organic phase was dried over anhydrous sodium sulfate, filtered, and all volatile components were removed under reduced pressure. Finally, the residue was redissolved in hexane and filtered through a silica plug (4 cm), and all volatile components were removed under reduced pressure to obtain **HPN**^**R**^.

##### Preparation of **HPN^Ad^**

Compound **2**^**Ad**^ (1.0 equiv, 12.49 mmol, 4.00 g), a 2.5 M solution of ^*n*^BuLi (2.05 equiv, 25.60 mmol, 10.24 mL) in hexane and chlorodiisopropylphosphine (1.1 equiv, 13.74 mmol, 2.10 g). Silica plug was washed with hexane and the product was elueted with 2% ethyl acetate. 2.13 g (47.7%) of a white solid. ^1^H NMR (400 MHz, C_6_D_6_, in ppm) δ = 7.12 (t, *J* = 2.6 Hz, 1H, C*H*_Ar_), 7.07 (dd, *J* = 8.3, 5.1 Hz, 1H, C*H*_Ar_), 7.02 (dd, *J* = 8.4, 2.0 Hz, 1H, C*H*_Ar_), 5.64 (d, *J* = 12.3 Hz, 1H, N*H*), 2.24 (s, 3H, C*H*_*3*_), 2.02 (m, 6H, C*H*_2,Ad_), 1.98 (m, 2H, C*H*(CH_3_)_2_), 1.94 (d, *J* = 4.1 Hz, 3H, C*H*_Ad_), 1.53 (m, 6H, C*H*_2,Ad_), 1.14 (dd, *J* = 15.5, 6.9 Hz, 6H, CH(C*H*_*3*_)_2_), 0.96 (dd, *J* = 12.0, 6.9 Hz, 6H, CH(C*H*_*3*_)_2_); ^13^C{^1^H} NMR (101 MHz, C_6_D_6_, in ppm) δ = 151.2 (*C*_Ar_), 133.7 (*C*H_Ar_), 130.6 (*C*H_Ar_), 125.6 (*C*_Ar_), 120.3 (*C*_Ar_), 116.6 (*C*_Ar_), 52.5 (*C*_Ad_), 43.9 (*C*H_2,Ad_), 36.9 (*C*H_2,Ad_), 30.3 (*C*H_Ad_), 24.4 (*C*H(CH_3_)_2_), 20.8 (CH(*C*H_3_)_2_), 20.6 (*C*H_3_), 19.4 (CH(*C*H_3_)_2_); ^31^P{^1^H} NMR (162 MHz, C_6_D_6_, in ppm) δ = −16.56 (s, 1P); ^31^P NMR (162 MHz, C_6_D_6_, in ppm) δ = −16.59 (s, 1P). Hi–Res Mass (ESI^+^) calcd for [C_23_H_37_NP]^+^: 358.2658; found: 358.2648. Elemental analysis (%) calcd for C_23_H_36_NP: C, 77.27; H, 10.15; N, 3.92; found: C, 77.11; H, 10.30; N, 3.77. UV/vis/NIR: λ_max_ = 327 (ε = 14570 L mol^–1^ cm^–1^). IR (cm^–1^): 3328, 2908, 2850, 1605, 1576, 1511, 1478, 1450, 1396, 1380, 1356, 1343, 1323, 1309, 1286, 1264, 1245, 1227, 1186, 1153, 1125, 1096, 1055, 1033, 992, 927, 880, 821, 806, 731, 678, 661, 610, 543, 494, 471, 455, 425.

##### Preparation of **HPN^3,5CF3^**

Compound **2**^**3,5CF3**^ (1.0 equiv, 8.18 mmol, 3.26 g), a 2.5 M solution of ^*n*^BuLi (2.05 equiv, 16.76 mmol, 6.7 mL) in hexane and chlorodiisopropylphosphine (1.1 equiv, 8.99 mmol, 1.37 g). 3.26 g (91.5%) of an off-white solid. Silica plug was performed with 1% ethyl acetate. ^1^H NMR (400 MHz, C_6_D_6_, in ppm) δ = 7.31 (s, 1H, C*H*_Ar_), 7.21 (s, 2H, C*H*_Ar_), 7.10 (s, 1H, C*H*_Ar_), 7.04 (dd, *J* = 8.1, 4.6 Hz, 1H, C*H*_Ar_), 6.99 (d, *J* = 9.6 Hz, 1H, N*H*), 6.78 (dd, *J* = 8.2, 1.8 Hz, 1H, C*H*_Ar_), 2.12 (s, 3H, C*H*_*3*_), 1.83 (m, 2H, C*H*(CH_3_)_2_), 0.91 (dd, *J* = 15.6, 7.0 Hz, 6H, CH(C*H*_*3*_)_2_), 0.83 (dd, *J* = 11.9, 6.9 Hz, 6H, CH(C*H*_*3*_)_2_); ^13^C{^1^H} NMR (101 MHz, C_6_D_6_, in ppm) δ = 146.2 (*C*_Ar_), 144.1 (*C*_Ar_), 143.9 (*C*_Ar_), 134.0 (*C*H_Ar_), 133.0 (q, *J* = 32.8 Hz, *C*F_3_), 132.4 (*C*_Ar_), 131.1 (*C*H_Ar_), 125.5 (*C*_Ar_), 124.1 (d, *J* = 272.8 Hz, *C*(CF_3_)), 119.7 (*C*H_Ar_), 115.2 (*C*H_Ar_), 112.8 (hept, *J* = 3.9 Hz, *C*H_Ar_), 23.0 (*C*H(CH_3_)_2_), 22.9 (*C*H(CH_3_)_2_), 20.9 (*C*H_3_), 20.2 (CH(*C*H_3_)_2_), 20.0 (CH(*C*H_3_)_2_), 18.8 (CH(*C*H_3_)_2_), 18.7 (CH(*C*H_3_)_2_); ^31^P{^1^H} NMR (162 MHz, C_6_D_6_, in ppm) δ = −14.54 (s, 1P); ^31^P NMR (162 MHz, C_6_D_6_, in ppm) δ = −14.57 (s, 1P); ^19^F NMR (377 MHz, C_6_D_6_, in ppm) δ = −62.88 (s, 6F); ^19^F{^1^H} NMR (377 MHz, C_6_D_6_, in ppm) δ = −62.88 (s, 6F). Hi–Res Mass (ESI^+^) calcd for [C_21_H_25_F_6_NP]^+^: 436.1624; found: 436.1618. Elemental analysis (%) calcd for C_21_H_24_F_6_NP · 0.66 H_2_O: C, 56.38; H, 5.71; N, 3.13; found: C, 56.17; H, 5.41; N, 3.03. UV/vis/NIR: λ_max_ = 326 (ε = 17440 L mol^–1^ cm^–1^), 348 (ε = 16900 L mol^–1^ cm^–1^). IR (cm^–1^): 3228, 3112, 2969, 2936, 2873, 1621, 1595, 1541, 1472, 1388, 1325, 1307, 1272, 1219, 1168, 1127, 1094, 1021, 996, 962, 931, 884, 855, 823, 806, 727, 696, 682, 643, 623, 600, 557, 533, 518, 486, 459, 441.

##### Preparation of **HPN^OMe^**

Compound **2**^**OMe**^ (1.0 equiv, 11.77 mmol, 3.44 g), a 2.5 M solution of ^*n*^BuLi (2.05 equiv, 24.14 mmol, 9.7 mL) in hexane and chlorodiisopropylphosphine (1.1 equiv, 12.95 mmol, 1.98 g). 3.42 g (88.2%) of a yellow oil. ^1^H NMR (400 MHz, C_6_D_6_, in ppm) δ = 7.80 (d, *J* = 9.8 Hz, 1H, N*H*), 7.52–7.41 (m, 2H, C*H*_Ar_), 7.19–7.16 (m, 1H, C*H*_Ar_), 6.93 (dd, *J* = 8.3, 2.1 Hz, 1H, C*H*_Ar_), 6.86 (td, *J* = 7.6, 1.4 Hz, 1H, C*H*_Ar_), 6.79 (td, *J* = 7.7, 1.6 Hz, 1H, C*H*_Ar_), 6.60 (dd, *J* = 7.9, 1.4 Hz, 1H, C*H*_Ar_), 3.33 (s, 3H, OC*H*_*3*_), 2.19 (s, 3H, C*H*_*3*_), 1.96 (pd, *J* = 7.0, 1.8 Hz, 2H, C*H*(CH_3_)_2_), 1.07 (dd, *J* = 15.4, 7.0 Hz, 6H, CH(C*H*_*3*_)_2_), 0.92 (dd, *J* = 11.6, 6.9 Hz, 6H, CH(C*H*_*3*_)_2_); ^13^C{^1^H} NMR (101 MHz, C_6_D_6_, in ppm) δ = 149.4 (*C*_Ar_), 146.6 (*C*_Ar_), 134.0 (*C*H_Ar_), 133.8 (*C*_Ar_), 130.7 (*C*H_Ar_), 129.3 (*C*_Ar_), 123.2 (*C*_Ar_), 121.2 (*C*H_Ar_), 119.9 (*C*H_Ar_), 118.0 (*C*H_Ar_), 114.4 (*C*H_Ar_), 111.1 (*C*H_Ar_), 55.3 (O*C*H_3_), 23.2 (*C*H(CH_3_)_2_), 20.9 (*C*H_3_), 20.4 (CH(*C*H_3_)_2_), 19.0 (CH(*C*H_3_)_2_); ^31^P{^1^H} NMR (162 MHz, C_6_D_6_, in ppm) δ = −14.59 (s, 1P); ^31^P NMR (162 MHz, C_6_D_6_, in ppm) δ = −14.58 (s, 1P). Hi–Res Mass (ESI^+^) calcd for [C_20_H_29_NOP]^+^: 330.1981; found: 330.1970. Elemental analysis (%) calcd for C_20_H_28_NOP: C, 72.92; H, 8.57; N, 4.25; found: C, 73.00; H, 8.52; N, 4.15. UV/vis/NIR: λ_max_ = 326 (ε = 9900 L mol^–1^ cm^–1^). IR (cm^–1^): 3353, 2952, 2924, 2867, 1595, 1568, 1515, 1488, 1462, 1392, 1362, 1339, 1294, 1266, 1239, 1176, 1147, 1115, 1049, 1029, 880, 817, 778, 737, 688, 661, 610, 580, 551, 523, 490, 453.

##### Preparation of **HPN^3,5Me^**

Compound **2**^**3,5Me**^ (1.0 equiv, 17.23 mmol, 5.00 g), a 2.5 M solution of ^*n*^BuLi (2.05 equiv, 35.32 mmol, 14.1 mL) in hexane and chlorodiisopropylphosphine (1.1 equiv, 18.95 mmol, 2.89 g). 4.20 g (74.4%) of a white solid. ^1^H NMR (400 MHz, C_6_D_6_, in ppm) δ = 7.44 (dd, *J* = 8.3, 4.8 Hz, 1H, C*H*_Ar_), 7.28 (d, *J* = 11.4 Hz, 1H, C*H*_Ar_), 7.15 (s, 1H, N*H*), 6.93 (dd, *J* = 8.3, 2.1 Hz, 1H, C*H*_Ar_), 6.80 (s, 2H, C*H*_Ar_), 6.51 (s, 1H, C*H*_Ar_), 2.19 (s, 3H, C*H*_*3*_), 2.11 (s, 6H, C*H*_*3*_), 1.96 (pd, *J* = 6.9, 2.3 Hz, 2H, C*H*(CH_3_)_2_), 1.08 (dd, *J* = 15.6, 7.0 Hz, 6H, CH(C*H*_*3*_)_2_), 0.95 (dd, *J* = 11.9, 6.9 Hz, 6H, CH(C*H*_*3*_)_2_); ^13^C{^1^H} NMR (101 MHz, C_6_D_6_, in ppm) δ = 147.5 (*C*_Ar_), 147.3 (*C*_Ar_), 144.1 (*C*_Ar_), 139.0 (*C*_Ar_), 133.6 (d, *J* = 2.7 Hz, *C*H_Ar_), 130.9 (*C*H_Ar_), 123.2 (*C*H_Ar_), 121.7 (*C*_Ar_), 117.8 (*C*H_Ar_), 116.2 (*C*H_Ar_), 23.3 (*C*H(CH_3_)_2_), 23.2 (*C*H(CH_3_)_2_), 21.5 (*C*H_3_), 20.9 (*C*H_3_), 20.3 (CH(*C*H_3_)_2_), 19.0 (CH(*C*H_3_)_2_); ^31^P{^1^H} NMR (162 MHz, C_6_D_6_, in ppm) δ = −15.48 (s, 1P); ^31^P NMR (162 MHz, C_6_D_6_, in ppm) δ = −15.49 (s, 1P). Hi–Res Mass (ESI^+^) calcd for [C_21_H_31_NP]^+^: 328.2189; found: 328.2180. Elemental analysis (%) calcd for C_21_H_30_NP: C, 77.03; H, 9.23; N, 4.28; found: C, 77.27; H, 9.23; N, 4.09. UV/vis/NIR: λ_max_ = 327 (ε = 13980 L mol^–1^ cm^–1^), 348 (ε = 13880 L mol^–1^ cm^–1^). IR (cm^–1^): 3353, 2961, 2920, 2863, 1595, 1509, 1472, 1388, 1360, 1329, 1307, 1290, 1268, 1256, 1241, 1168, 1143, 1100, 1076, 1066, 1031, 1021, 880, 841, 819, 733, 712, 700, 688, 672, 661, 647, 612, 565, 547, 492, 459.

##### Preparation of **HPN^Dipp^**

Compound **2**^**Dipp**^ (1.0 equiv, 7.22 mmol, 2.50 g), a 2.5 M solution of ^*n*^BuLi (2.05 equiv, 14.80 mmol, 5.9 mL) in hexane and chlorodiisopropylphosphine (1.1 equiv, 7.94 mmol, 1.21 g). 1.67 g (60.3%) of a yellow oil. ^1^H NMR (400 MHz, C_6_D_6_, in ppm) δ = 7.23 (m, 2H, C*H*_Ar_), 7.20 (s, 1H, N*H*), 7.17 (s, 1H, C*H*_Ar_), 7.15–7.12 (m, 1H, C*H*_Ar_), 6.80 (dd, *J* = 8.3, 1.9 Hz, 1H, C*H*_Ar_), 6.29 (dd, *J* = 8.3, 5.3 Hz, 1H, C*H*_Ar_), 3.40 (hept, *J* = 6.9 Hz, 2H, C*H*(CH_3_)_2_), 2.15 (s, 3H, C*H*_*3*_), 2.05 (heptd, *J* = 6.9, 2.9 Hz, 2H, PC*H*(CH_3_)_2_), 1.22–1.15 (m, 18H, 3· CH(C*H*_*3*_)_2_), 1.04 (dd, *J* = 12.1, 6.9 Hz, 6H, CH(C*H*_*3*_)_2_); ^13^C{^1^H} NMR (101 MHz, C_6_D_6_, in ppm) δ = 152.3 (*C*_Ar_), 147.9 (*C*_Ar_), 141.5 (*C*_Ar_), 137.3 (*C*_Ar_), 133.5 (*C*H_Ar_), 131.4 (*C*H_Ar_), 127.5 (*C*H_Ar_), 125.9 (*C*_Ar_), 124.2 (*C*H_Ar_), 116.4 (*C*_Ar_), 111.7 (*C*H_Ar_), 29.0 (*C*H(CH_3_)_2_), 25.0 (C*H*(CH_3_)_2_), 23.8 (PC*H*(CH_3_)_2_), 23.1 (PC*H*(CH_3_)_2_), 20.1 (CH(*C*H_3_)_2_), 19.2 (PCH(*C*H_3_)_2_); ^31^P{^1^H} NMR (162 MHz, C_6_D_6_, in ppm) δ = −17.79 (s, 1P); ^31^P NMR (162 MHz, C_6_D_6_, in ppm) δ = −17.83 (s, 1P). Hi–Res Mass (ESI^+^) calcd for [C_25_H_39_NP]^+^: 384.2815; found: 384.2818. Elemental analysis (%) calcd for C_25_H_38_NP: C, 78.29; H, 9.99; N, 3.65; found: C, 78.11; H, 10.04; N, 3.54. UV/vis/NIR: λ_max_ = 327 (ε = 12580 L mol^–1^ cm^–1^). IR (cm^–1^): 3285, 2961, 2924, 2865, 1605, 1588, 1492, 1460, 1380, 1362, 1347, 1327, 1290, 1266, 1249, 1178, 1141, 1104, 1058, 1043, 1031, 1025, 935, 878, 815, 790, 741, 731, 700, 686, 665, 635, 614, 565, 529, 486, 453.

##### Preparation of **HPN^Tol^**

Compound **2**^**Tol**^ (1.0 equiv, 14.48 mmol, 4.00 g), a 2.5 M solution of ^*n*^BuLi (2.05 equiv, 29.69 mmol, 11.9 mL) in hexane and chlorodiisopropylphosphine (1.1 equiv, 15.93 mmol, 2.43 g). 2.86 g (63.0%) of a yellow oil. ^1^H NMR (400 MHz, C_6_D_6_, in ppm) δ = 7.33 (dd, *J* = 8.4, 4.9 Hz, 1H, C*H*_Ar_), 7.28 (d, *J* = 11.5 Hz, 1H, N*H*), 7.14 (m, 2H, C*H*_Ar_), 7.03 (d, *J* = 8.4 Hz, 2H, C*H*_Ar_), 6.93 (d, *J* = 5.6 Hz, 2H, C*H*_Ar_), 2.19 (s, 3H, C*H*_*3*_), 2.12 (s, 3H, C*H*_*3*_), 1.95 (m, 2H, C*H*(CH_3_)_2_), 1.06 (dd, *J* = 15.5, 7.0 Hz, 6H, CH(C*H*_*3*_)_2_), 0.94 (dd, *J* = 11.9, 6.9 Hz, 6H, CH(C*H*_*3*_)_2_); ^13^C{^1^H} NMR (101 MHz, C_6_D_6_, in ppm) δ = 147.8 (*C*_Ar_), 141.7 (*C*_Ar_), 141.5 (*C*_Ar_), 133.6 (*C*H_Ar_), 133.5 (*C*H_Ar_), 130.5 (*C*H_Ar_), 130.2 (*C*_Ar_), 128.6 (*C*H_Ar_), 120.5 (*C*_Ar_), 119.1 (*C*H_Ar_), 116.8 (*C*H_Ar_), 23.3 (*C*H(CH_3_)_2_), 20.9 (*C*H(CH_3_)_2_), 20.7 (*C*H_3_), 20.4 (*C*H_3_), 19.0 (CH(*C*H_3_)_2_); ^31^P{^1^H} NMR (162 MHz, C_6_D_6_, in ppm) δ = −15.86 (s, 1P); ^31^P NMR (162 MHz, C_6_D_6_, in ppm) δ = −15.83 (s, 1P). Hi–Res Mass (ESI^+^) calcd for [C_20_H_29_NP]^+^: 314.2032; found: 314.2023. Elemental analysis (%) calcd for C_20_H_28_NP · 0.33 H_2_O: C, 75.20; H, 9.05; N, 4.39; found: C, 75.68; H, 8.89; N, 4.22. UV/vis/NIR: λ_max_ = 326 (ε = 15790 L mol^–1^ cm^–1^), 349 (ε = 15910 L mol^–1^ cm^–1^). IR (cm^–1^): 3338, 2961, 2950, 2922, 2897, 2863, 1617, 1601, 1515, 1460, 1411, 1384, 1360, 1309, 1266, 1245, 1221, 1180, 1143, 1123, 1113, 1074, 1031, 1021, 880, 831, 819, 806, 729, 696, 663, 649, 610, 596, 559, 551, 523, 500, 471, 433.

#### General Procedure for the Preparation of **LiPN**^**R**^

In a 100-mL Schlenk flask, **HPN**^**R**^ (1.0 equiv) was dissolved in hexane and cooled to −78 °C. Afterward, ^*n*^BuLi (1.0 equiv) was added dropwise via a syringe. The reaction mixture was allowed to warm up to room temperature and stirred overnight. On the next day, the resulting suspension was filtered in a glovebox and washed with a small amount of −40 °C cold hexane (3 mL). The solid was dried in vacuo to obtain clean **LiPN**^**R**^.

##### Preparation of **LiPN^Ad^**

Compound **HPN**^**Ad**^ (1.0 equiv, 5.96 mmol, 2.13 g) and a 2.5 M solution of ^*n*^BuLi (1.0 equiv, 5.96 mmol, 2.38 mL) in hexane. 1.63 g (75.3%) of a yellow solid. ^1^H NMR (400 MHz, C_6_D_6_, in ppm) δ = 6.98 (s, 2H, C*H*_Ar_), 6.77 (s, 1H, C*H*_Ar_), 2.28 (s, 3H, C*H*_*3*_), 2.09 (br. s, 9H, C*H* & C*H*_2,Ad_), 2.04–1.90 (m, 2H, C*H*(CH_3_)_2_), 1.68 (br. s, 6H, C*H*_2,Ad_), 1.07 (dd, *J* = 12.9, 6.9 Hz, 6H, CH(C*H*_*3*_)_2_), 0.97–0.84 (m, 6H, CH(C*H*_*3*_)_2_); ^13^C{^1^H} NMR (101 MHz, C_6_D_6_, in ppm) δ = 161.7 (*C*_Ar_), 141.5 (*C*_Ar_), 132.6 (*C*H_Ar_), 130.4 (*C*H_Ar_), 128.8 (*C*_Ar_), 123.1 (*C*H_Ar_), 54.3 (*C*_Ad_), 46.5 (*C*H_2,Ad_), 37.4 (*C*H_2,Ad_), 31.0 (*C*H_Ad_), 23.1 (*C*H(CH_3_)_2_), 20.8 (*C*H_3_), 20.3 (CH(*C*H_3_)_2_), 19.6 (CH(*C*H_3_)_2_); ^31^P{^1^H} NMR (162 MHz, C_6_D_6_, in ppm) δ = −1.73 (s, 1P); ^31^P NMR (162 MHz, C_6_D_6_, in ppm) δ = −1.78 (s, 1P); ^7^Li{^1^H} NMR (156 MHz, C_6_D_6_, in ppm) δ = 3.16 (s, 1Li); ^7^Li NMR (156 MHz, C_6_D_6_, in ppm) δ = 3.16 (s, 1Li). Elemental analysis (%) calcd for C_23_H_35_LiNP: C, 76.01; H, 9.71; N, 3.85; found: C, 75.48; H, 9.86; N, 3.85. UV/vis/NIR: λ_max_ = 317 (ε = 23130 L mol^–1^ cm^–1^), 380 (ε = 12430 L mol^–1^ cm^–1^). IR (cm^–1^): 2959, 2903, 2883, 2848, 2824, 1597, 1458, 1386, 1362, 1352, 1305, 1278, 1231, 1211, 1180, 1141, 1098, 1082, 1053, 1027, 935, 872, 825, 815, 788, 776, 706, 667, 645, 612, 549, 535, 506, 478, 449, 410.

##### Preparation of **LiPN^3,5CF3^**

Compound **HPN**^**3,5CF3**^ (1.0 equiv, 8.18 mmol, 3.56 g) and a 2.5 M solution of ^*n*^BuLi (1.0 equiv, 8.18 mmol, 3.27 mL) in hexane. 2.61 g (72.3%) of a yellow solid. ^1^H NMR (400 MHz, C_6_D_6_, in ppm) δ = 7.13 (s, 1H, C*H*_Ar_), 7.08 (m, 2H, C*H*_Ar_), 6.99 (s, 1H, C*H*_Ar_), 6.75 (br. s, 2H, C*H*_Ar_), 2.28 (s, 3H, C*H*_*3*_), 1.83 (br. s, 2H, C*H*(CH_3_)_2_), 0.89 (br. s, 6H, CH(C*H*_*3*_)_2_), 0.80 (br. s, 6H, CH(C*H*_*3*_)_2_); ^13^C{^1^H} NMR (101 MHz, C_6_D_6_, in ppm) δ = 159.7 (*C*_Ar_), 134.4 (*C*H_Ar_), 133.3 (*C*_Ar_), 131.9 (*C*H_Ar_), 131.7 (*C*_Ar_), 126.8 (*C*_Ar_), 124.8 (*C*H_Ar_), 123.2 (*C*_Ar_), 113.7 (*C*H_Ar_), 105.8 (*C*H_Ar_), 21.6 (*C*H(CH_3_)_2_), 21.0 (*C*H_3_), 19.8 (CH(*C*H_3_)_2_), 18.6 (CH(*C*H_3_)_2_); ^31^P{^1^H} NMR (162 MHz, C_6_D_6_, in ppm) δ = −7.46 (s, 1P); ^31^P NMR (162 MHz, C_6_D_6_, in ppm) δ = −7.41 (s, 1P); ^19^F NMR (377 MHz, C_6_D_6_, in ppm) δ = −63.21 (s, 6F); ^19^F{^1^H} NMR (377 MHz, C_6_D_6_, in ppm) δ = −63.21 (s, 6F); ^7^Li{^1^H} NMR (156 MHz, C_6_D_6_, in ppm) δ = 0.84 (s, 1Li); ^7^Li NMR (156 MHz, C_6_D_6_, in ppm) δ = 0.83 (s, 1Li). Elemental analysis (%) calcd for C_21_H_23_F_6_LiNP: C, 57.15; H, 5.25; N, 3.17; found: C, 56.39; H, 5.68; N, 2.91. UV/vis/NIR: λ_max_ = 327 (ε = 25700 L mol^–1^ cm^–1^), 351 (ε = 25870 L mol^–1^ cm^–1^). IR (cm^–1^): 2955, 2926, 2871, 1593, 1470, 1374, 1278, 1207, 1172, 1164, 1123, 1068, 1025, 982, 947, 882, 864, 847, 829, 798, 721, 704, 692, 680, 653, 604, 541, 508, 494, 471, 459.

##### Preparation of **LiPN^OMe^**

Compound **HPN**^**OMe**^ (1.0 equiv, 11.59 mmol, 3.82 g) and a 2.5 M solution of ^*n*^BuLi (1.0 equiv, 11.59 mmol, 4.64 mL) in hexane. 2.67 g (68.7%) of a yellow solid. ^1^H NMR (400 MHz, C_6_D_6_, in ppm) δ = 7.37–7.26 (m, 1H, C*H*_Ar_), 7.12 (m, 1H, C*H*_Ar_), 7.03–6.86 (m, 3H, C*H*_Ar_), 6.63–6.33 (m, 2H, C*H*_Ar_), 3.01 (m, 3H, OC*H*_*3*_), 2.24 (m, 3H, C*H*_*3*_), 1.88 (m, 2H, C*H*(CH_3_)_2_), 1.33–0.36 (m, 12H, CH(C*H*_*3*_)_2_); ^13^C{^1^H} NMR (101 MHz, C_6_D_6_, in ppm) δ = 165.8 (*C*_Ar_), 165.6 (*C*_Ar_), 152.3 (*C*_Ar_), 151.8 (*C*H_Ar_), 133.3 (*C*H_Ar_), 132.3 (*C*H_Ar_), 127.1 (*C*H_Ar_), 126.6 (*C*_Ar_), 124.6 (*C*_Ar_), 123.2 (*C*H_Ar_), 119.1 (*C*H_Ar_), 115.2 (*C*_Ar_), 113.3 (*C*_Ar_), 109.5 (*C*H_Ar_), 54.7 (O*C*H_3_), 24.5 (*C*H(CH_3_)_2_), 20.5 (*C*H_3_), 19.9 (CH(*C*H_3_)_2_), 17.6 (CH(*C*H_3_)_2_); ^31^P{^1^H} NMR (162 MHz, C_6_D_6_, in ppm) δ = −3.78–-5.97 (m, 1P); ^31^P NMR (162 MHz, C_6_D_6_, in ppm) δ = −4.74 (br. s, 1P); ^7^Li{^1^H} NMR (156 MHz, C_6_D_6_, in ppm) δ = 3.21 (m, 1Li); ^7^Li NMR (156 MHz, C_6_D_6_, in ppm) δ = 3.21 (m, 1Li). Elemental analysis (%) calcd for C_20_H_27_LiNOP: C, 71.63; H, 8.12; N, 4.18; found: C, 71.97; H, 8.52; N, 3.85. UV/vis/NIR: λ_max_ = 326 (ε = 259820 L mol^–1^ cm^–1^), 358 (ε = 273260 L mol^–1^ cm^–1^), 641 (ε = 6200 L mol^–1^ cm^–1^). IR (cm^–1^): 2952, 2922, 2867, 2840, 1588, 1564, 1486, 1462, 1452, 1384, 1327, 1284, 1264, 1217, 1192, 1174, 1156, 1135, 1115, 1049, 1029, 892, 880, 853, 843, 825, 794, 776, 727, 688, 665, 598, 549, 498, 467, 422.

##### Preparation of **LiPN^3,5Me^**

Compound **HPN**^**3,5Me**^ (1.0 equiv, 12.81 mmol, 4.20 g) and a 2.5 M solution of ^*n*^BuLi (1.0 equiv, 12.81 mmol, 5.13 mL) in hexane. 2.41 g (56.6%) of a yellow solid. ^1^H NMR (400 MHz, C_6_D_6_, in ppm) δ = 7.54 (dd, *J* = 8.1, 4.3 Hz, 1H, C*H*_Ar_), 7.25 (s, 1H, C*H*_Ar_), 7.15–7.11 (m, 1H, C*H*_Ar_), 6.55 (s, 2H, C*H*_Ar_), 6.11 (s, 1H, C*H*_Ar_), 2.35 (s, 3H, C*H*_*3*_), 1.98 (s, 6H, C*H*_*3*_), 1.34 (m, 2H, C*H*(CH_3_)_2_), 1.18 (br. s, 6H, CH(C*H*_*3*_)_2_), 1.11–1.04 (m, 6H, CH(C*H*_*3*_)_2_); ^13^C{^1^H} NMR (101 MHz, C_6_D_6_, in ppm) δ = 159.8 (*C*_Ar_), 158.1 (*C*_Ar_), 141.5 (*C*_Ar_), 139.3 (*C*_Ar_), 133.5 (*C*H_Ar_), 130.9 (*C*H_Ar_), 128.3 (*C*H_Ar_), 127.8 (*C*_Ar_), 124.6 (*C*H_Ar_), 115.9 (*C*H_Ar_), 112.5 (*C*H_Ar_), 21.8 (*C*H_3_), 21.1 (*C*H_3_), 20.3 (CH(*C*H_3_)_2_); ^31^P{^1^H} NMR (162 MHz, C_6_D_6_, in ppm) δ = −5.14 (s, 1P); ^31^P NMR (162 MHz, C_6_D_6_, in ppm) δ = −5.16 (s, 1P); ^7^Li{^1^H} NMR (156 MHz, C_6_D_6_, in ppm) δ = 3.67 (br. s, 1Li), 0.53 (br. s, 1Li); ^7^Li NMR (156 MHz, C_6_D_6_, in ppm) δ = 3.72 (br. s, 1Li), 0.48 (br. s, 1Li). Elemental analysis (%) calcd for C_21_H_29_LiNP: C, 75.66; H, 8.77; N, 4.20; found: C, 75.18; H, 8.92; N, 4.03. UV/vis/NIR: λ_max_ = 326 (ε = 21760 L mol^–1^ cm^–1^), 360 (ε = 23230 L mol^–1^ cm^–1^). IR (cm^–1^): 2955, 2924, 2867, 1578, 1464, 1384, 1329, 1298, 1270, 1225, 1168, 1139, 1033, 982, 960, 878, 849, 831, 815, 806, 704, 690, 657, 614, 592, 553, 502, 476, 453.

##### Preparation of **LiPN^Dipp^**

Compound **HPN**^**Dipp**^ (1.0 equiv, 1.49 mmol, 0.57 g) and a 2.5 M solution of ^*n*^BuLi (1.0 equiv, 1.49 mmol, 0.6 mL) in hexane. 0.49 g (85.2%) of a yellow solid. ^1^H NMR (400 MHz, C_6_D_6_, in ppm) δ = 7.36 (d, *J* = 7.6 Hz, 2H, C*H*_Ar_), 7.26–7.18 (m, 1H, C*H*_Ar_), 6.83 (dd, *J* = 5.8, 2.2 Hz, 1H, C*H*_Ar_), 6.78 (dd, *J* = 8.5, 2.3 Hz, 1H, C*H*_Ar_), 5.93 (dd, *J* = 8.4, 6.3 Hz, 1H, C*H*_Ar_), 3.32 (hept, *J* = 6.9 Hz, 2H, C*H*(CH_3_)_2_), 2.21 (s, 3H, C*H*_*3*_), 1.96–1.75 (m, *J* = 6.9 Hz, 2H, C*H*(CH_3_)_2_), 1.29 (d, *J* = 7.0 Hz, 6H, CH(C*H*_*3*_)_2_), 1.25 (m, 6H, CH(C*H*_*3*_)_2_), 1.00 (m, 12H, CH(C*H*_*3*_)_2_); ^13^C{^1^H} NMR (101 MHz, C_6_D_6_, in ppm) δ = 165.1 (*C*_Ar_), 164.9 (*C*_Ar_), 152.1 (*C*_Ar_), 144.8 (*C*_Ar_), 133.2 (*C*H_Ar_), 132.6 (*C*H_Ar_), 123.8 (*C*H_Ar_), 122.1 (*C*H_Ar_), 116.8 (*C*H_Ar_), 113.3 (*C*H_Ar_), 109.6 (*C*_Ar_), 28.4 (*C*H(CH_3_)_2_), 24.6 (*C*H(CH_3_)_2_), 23.6 (P*C*H(CH_3_)_2_), 20.5 (*C*H_3_), 19.3 (CH(*C*H_3_)_2_); ^31^P{^1^H} NMR (162 MHz, C_6_D_6_, in ppm) δ = −10.89 (s, 1P); ^31^P NMR (162 MHz, C_6_D_6_, in ppm) δ = −10.92 (s, 1P); ^7^Li{^1^H} NMR (156 MHz, C_6_D_6_, in ppm) δ = −0.88 (s, 1Li); ^7^Li NMR (156 MHz, C_6_D_6_, in ppm) δ = −0.88 (s, 1Li). Elemental analysis (%) calcd for C_25_H_37_LiNP · C_4_H_10_O: C, 75.13; H, 10.22; N, 3.02; found: C, 74.83; H, 9.89; N, 3.41. UV/vis/NIR: λ_max_ = 327 (ε = 23300 L mol^–1^ cm^–1^), 421 (ε = 19640 L mol^–1^ cm^–1^). IR (cm^–1^): 2961, 2865, 1601, 1497, 1464, 1417, 1382, 1362, 1317, 1286, 1268, 1227, 1149, 1102, 1058, 1033, 880, 812, 788, 686, 616, 461.

##### Preparation of **LiPN^Tol^**

Compound **HPN**^**Tol**^ (1.0 equiv, 6.38 mmol, 2.00 g) and a 2.5 M solution of ^*n*^BuLi (1.0 equiv, 6.38 mmol, 2.6 mL) in hexane. 1.44 g (70.8%) of a yellow solid. ^1^H NMR (400 MHz, C_6_D_6_, in ppm) δ = 7.36 (br. s, 2H, C*H*_Ar_), 7.13 (br. s, 1H, C*H*_Ar_), 6.75 (d, *J* = 6.6 Hz, 2H, C*H*_Ar_), 6.63 (br. s, 2H, C*H*_Ar_), 2.46 (s, 3H, C*H*_*3*_), 1.98 (br. s, 5H, C*H*_*3*_ & C*H*(CH_3_)_2_), 1.11–0.92 (m, 12H, CH(C*H*_*3*_)_2_); ^13^C{^1^H} NMR (101 MHz, C_6_D_6_, in ppm) δ = 156.8 (*C*_Ar_), 141.5 (*C*_Ar_), 133.3 (*C*H_Ar_), 131.4 (*C*H_Ar_), 130.9 (*C*H_Ar_), 128.8 (*C*_Ar_), 126.8 (*C*_Ar_), 114.9 (*C*H_Ar_), 21.3 (*C*H_3_), 20.5 (*C*H_3_), 20.0 (*C*H(CH_3_)_2_), 19.7 (CH(*C*H_3_)_2_); ^31^P{^1^H} NMR (162 MHz, C_6_D_6_, in ppm) δ = −7.40 (s, 1P); ^31^P NMR (162 MHz, C_6_D_6_, in ppm) δ = −7.57 (s, 1P); ^7^Li{^1^H} NMR (156 MHz, C_6_D_6_, in ppm) δ = 3.02 (br. s, 1Li), −1.64 (br. s, 1Li); ^7^Li NMR (156 MHz, C_6_D_6_, in ppm) δ = 2.98 (br. s, 1Li), −1.71 (br. s, 1Li). Elemental analysis (%) calcd for C_20_H_27_LiNP: C, 75.22; H, 8.52; N, 4.39; found: C, 75.29; H, 8.89; N, 4.09. UV/vis/NIR: λ_max_ = 326 (ε = 23280 L mol^–1^ cm^–1^), 360 (ε = 24780 L mol^–1^ cm^–1^). IR (cm^–1^): 2957, 2920, 2867, 1605, 1597, 1499, 1464, 1415, 1386, 1315, 1301, 1288, 1268, 1245, 1194, 1182, 1158, 1137, 1119, 1035, 1025, 1004, 880, 857, 835, 810, 774, 721, 706, 661, 614, 553, 525, 504, 496, 480, 457.

#### General Procedure for the Preparation of La(PN^R^)_2_Cl/(PN^R^)_2_La(μ-Cl_2_)La(PN^R^)_2_ (**3a**–**f**) Complexes

**LiPN**^**R**^ (2.0 equiv) and LaCl_3_(thf)_1.2_ (1.0 equiv) were combined and toluene (40 mL) was added. Afterward, the suspension was stirred at 120 °C overnight. The reaction mixture was allowed to cool down to room temperature and all volatile components were removed in vacuo. The resulting residue was suspended in hexane and the suspension was filtered through a medium porosity frit. The solid was washed with −40 °C cold pentane (2 × 2 mL). The obtained solid was dried *in vacuo* to yield the corresponding complexes.

##### Preparation of **3a**

From **LiPN**^**Ad**^ (2.0 equiv, 550 μmol, 200 mg) and LaCl_3_(thf)_1.2_ (1.0 equiv, 275 μmol, 90.8 mg). 134 mg (55.3%) of a yellow solid. ^1^H NMR (400 MHz, C_6_D_6_, in ppm) δ = 6.86 (s, 4H, C*H*_Ar_), 6.77 (s, 4H, C*H*_Ar_), 6.52 (s, 4H, C*H*_Ar_), 2.40 (s, 8H, C*H*(CH_3_)_2_), 2.29 (s, 12H, C*H*_*3*_), 2.18 (s, 24H, C*H*_2,Ad_), 1.81 (s, 12H, C*H*_Ad_), 1.74 (s, 24H, C*H*_2,Ad_), 1.62 (s, 24H, CH(C*H*_*3*_)_2_), 1.49–0.98 (m, 24H, CH(C*H*_*3*_)_2_); ^13^C{^1^H} NMR (101 MHz, C_6_D_6_, in ppm) δ = 153.1 (*C*_Ar_), 148.6 (*C*_Ar_), 141.5 (*C*_Ar_), 46.4 (*C*H_2,Ad_), 37.3 (*C*H_2,Ad_), 31.0 (*C*H_Ad_), 21.0 (CH(*C*H_3_)_2_); ^31^P{^1^H} NMR (162 MHz, C_6_D_6_, in ppm) δ = 16.02 (s, 4P); ^31^P NMR (162 MHz, C_6_D_6_, in ppm) δ = 16.20 (s, 4P). Elemental analysis (%) calcd for C_92_H_140_Cl_2_La_2_N_4_P_4_: C, 62.26; H, 7.95; N, 3.16; found: C, 58.98; H, 7.81; N, 2.73; (Despite numerous attempts, due to the high reactivity of complex **3a** toward moisture and air, no better elemental analysis could be obtained.) UV/vis/NIR: λ_max_ = 328 (ε = 125170 L mol^–1^ cm^–1^), 358 (ε = 134580 L mol^–1^ cm^–1^). IR (cm^–1^): 2901, 2848, 1597, 1456, 1388, 1366, 1352, 1303, 1276, 1227, 1149, 1098, 1082, 1055, 982, 939, 872, 829, 808, 788, 778, 729, 708, 688, 657, 641, 531, 469, 431.

##### Preparation of **3d**

From **LiPN**^**3,5Me**^ (2.0 equiv, 600 μmol, 200 mg) and LaCl_3_(thf)_1.2_ (1.0 equiv, 300 μmol, 99.0 mg). 196 mg (79.0%) of a yellow solid. ^1^H NMR (400 MHz, C_6_D_6_, in ppm) δ = 7.12 (d, *J* = 7.4 Hz, 4H, C*H*_Ar_), 6.97 (d, *J* = 8.3 Hz, 8H, C*H*_Ar_), 6.90 (s, 8H, C*H*_Ar_), 6.50 (s, 4H, C*H*_Ar_), 2.19 (s, 24H, C*H*_*3*_), 2.18 (s, 12H, C*H*_*3*_), 1.31–1.22 (m, 24H, CH(C*H*_*3*_)_2_), 1.22–1.16 (m, 8H, C*H*(C*H*_*3*_)_2_), 0.95 (m, 24H, CH(C*H*_*3*_)_2_); ^13^C{^1^H} NMR (101 MHz, C_6_D_6_, in ppm) δ = 161.4 (*C*_Ar_), 141.3 (*C*_Ar_), 132.8 (*C*_Ar_), 132.3 (*C*H_Ar_), 128.8 (*C*H_Ar_), 125.9 (*C*_Ar_), 123.3 (*C*H_Ar_), 119.0 (*C*H_Ar_), 118.7 (*C*H_Ar_), 21.8 (*C*H_3_), 19.7 (*C*H(CH_3_)_2_), 19.1 (CH(*C*H_3_)_2_), 18.1 (CH(*C*H_3_)_2_); ^31^P{^1^H} NMR (162 MHz, C_6_D_6_, in ppm) δ = 7.54 (s, 2P); ^31^P NMR (162 MHz, C_6_D_6_, in ppm) δ = 7.51 (s, 2P). Elemental analysis (%) calcd for C_84_H_116_Cl_2_La_2_N_4_P_4_: C, 60.98; H, 7.07; N, 3.39; found: C, 60.46; H, 7.28; N, 3.29. UV/vis/NIR: λ_max_ = 326 (ε = 68120 L mol^–1^ cm^–1^), 359 (ε = 72230 L mol^–1^ cm^–1^), 391 (ε = 53790 L mol^–1^ cm^–1^). IR (cm^–1^): 2957, 2920, 2869, 1580, 1544, 1511, 1468, 1386, 1321, 1303, 1276, 1237, 1213, 1156, 1031, 988, 955, 884, 837, 821, 804, 710, 692, 680, 657, 614, 580, 555, 533, 498, 471, 433.

##### Preparation of **3e**

From **LiPN**^**Dipp**^ (2.0 equiv, 513 μmol, 200 mg) and LaCl_3_(thf)_1.2_ (1.0 equiv, 257 μmol, 84.7 mg). 153 mg (63.4%) of a yellow solid. ^1^H NMR (400 MHz, C_6_D_6_, in ppm) δ = 7.28 (d, *J* = 7.5 Hz, 4H, C*H*_Ar_), 7.22 (d, *J* = 7.5 Hz, 2H, C*H*_Ar_), 6.87–6.81 (m, 2H, C*H*_Ar_), 6.65 (dd, *J* = 8.5, 2.0 Hz, 2H, C*H*_Ar_), 5.58 (dd, *J* = 8.4, 5.1 Hz, 2H, C*H*_Ar_), 3.55 (br. s, 4H, C*H*(CH_3_)_2_), 2.06 (s, 6H, C*H*_*3*_), 2.00 (br. s, 4H, PC*H*(CH_3_)_2_) 1.42 (br. s, 24H, CH(C*H*_*3*_)_2_), 1.10 (br. s, 24H, CH(C*H*_*3*_)_2_); ^13^C{^1^H} NMR (101 MHz, C_6_D_6_, in ppm) δ = 162.9 (*C*_Ar_), 147.9 (*C*_Ar_), 137.7 (*C*_Ar_), 133.1 (*C*H_Ar_), 132.4 (*C*H_Ar_), 127.5 (*C*H_Ar_), 126.7 (*C*H_Ar_), 123.6 (*C*_Ar_), 116.9 (*C*H_Ar_), 28.6 (*C*H(CH_3_)_2_), 26.6 (CH(*C*H_3_)_2_), 26.1 (*C*H(CH_3_)_2_), 22.7 (CH(*C*H_3_)_2_), 20.5 (*C*H_3_), 20.0 (CH(*C*H_3_)_2_; ^31^P{^1^H} NMR (162 MHz, C_6_D_6_, in ppm) δ = 8.93 (s, 2P); ^31^P NMR (162 MHz, C_6_D_6_, in ppm) δ = 8.90 (s, 2P). Elemental analysis (%) calcd for C_50_H_74_ClLaN_2_P_2_: C, 63.93; H, 7.94; N, 2.98; found: C, 64.01; H, 8.04; N, 2.89. UV/vis/NIR: λ_max_ = 319 (ε = 46520 L mol^–1^ cm^–1^), 360 (ε = 46180 L mol^–1^ cm^–1^). IR (cm^–1^): 2959, 2924, 2867, 1603, 1497, 1468, 1423, 1384, 1362, 1315, 1278, 1252, 1213, 1174, 1147, 1104, 1053, 933, 884, 851, 839, 815, 790, 733, 694, 647, 612, 539, 500, 471, 429.

##### Preparation of **3f**

From **LiPN**^**Tol**^ (2.0 equiv, 626 μmol, 200 mg) and LaCl_3_(thf)_1.2_ (1.0 equiv, 313 μmol, 103 mg). 186 mg (74.1%) of a yellow solid. ^1^H NMR (400 MHz, C_6_D_6_, in ppm) δ = 7.12 (d, *J* = 9.1 Hz, 8H, C*H*_Ar_), 7.06 (d, *J* = 8.1 Hz, 8H, C*H*_Ar_), 6.94 (d, *J* = 7.1 Hz, 8H, C*H*_Ar_), 6.88 (dd, *J* = 9.0, 4.4 Hz, 4H, C*H*_Ar_), 2.17 (s, 12H, C*H*_*3*_), 2.09 (s, 12H, C*H*_*3*_), 2.05 (d, *J* = 6.5 Hz, 8H, C*H*(CH_3_)_2_), 1.21–1.09 (m, 24H, CH(C*H*_*3*_)_2_), 1.09–0.99 (m, 24H, CH(C*H*_*3*_)_2_); ^13^C{^1^H} NMR (101 MHz, C_6_D_6_, in ppm) δ = 162.0 (*C*_Ar_), 161.8 (*C*_Ar_), 145.3 (*C*_Ar_), 133.3 (*C*H_Ar_), 132.6 (*C*H_Ar_), 131.1 (*C*_Ar_), 125.7 (*C*_Ar_), 125.2 (*C*H_Ar_), 123.9 (*C*H_Ar_), 117.2 (*C*H_Ar_), 24.3 (*C*H(CH_3_)_2_), 19.7 (CH(*C*H_3_)_2_), 18.3 (CH(*C*H_3_)_2_); ^31^P{^1^H} NMR (162 MHz, C_6_D_6_, in ppm) δ = 6.47 (s, 2P); ^31^P NMR (162 MHz, C_6_D_6_, in ppm) δ = 6.47 (s, 2P). Elemental analysis (%) calcd for C_80_H_108_Cl_2_La_2_N_4_P_4_ · C_6_H_14_: C, 61.32; H, 7.30; N, 3.33; found: C, 61.58; H, 7.13; N, 3.26. UV/vis/NIR: λ_max_ = 326 (ε = 52220 L mol^–1^ cm^–1^), 361 (ε = 56270 L mol^–1^ cm^–1^), 398 (ε = 55180 L mol^–1^ cm^–1^). IR (cm^–1^): 2955, 2920, 2867, 1599, 1494, 1466, 1409, 1384, 1284, 1213, 1188, 1174, 1143, 1107, 1031, 880, 843, 810, 727, 706, 694, 655, 614, 506, 490, 465.

#### General Procedure for the Preparation of [Li(DME)_3_][(PN^R^)_2_LaCl_2_] and [(PN^R^)_2_La(μ-Cl_2_)Li(DME)] (**4a**–**f**)

**LiPN**^**R**^ (2.0 equiv) and LaCl_3_(thf)_1.2_ (1.0 equiv) were combined, suspended in 1,2-dimethoxyethane (15 mL), and stirred at room temperature overnight. On the next day, the reaction mixture was centrifuged and washed twice with small amounts of −40 °C cold pentane to remove possible impurities. The obtained solid was dried in vacuo to obtain the corresponding −*ate* complexes.

##### Preparation of **4a**

From **LiPN**^**Ad**^ (2.0 equiv, 275 μmol, 100 mg) and LaCl_3_(thf)_1.2_ (1.0 equiv, 138 μmol, 45.4 mg). 73.5 mg (52.4%) of an off-white solid. ^1^H NMR (400 MHz, C_6_D_6_, in ppm) δ = 7.05 (s, 4H, C*H*_Ar_), 6.94 (s, 2H, C*H*_Ar_), 3.14 (s, 6H, DME-C*H*_*3*_), 2.87 (s, 4H, DME-C*H*_*2*_), 2.45 (s, 12H, C*H*_2,Ad_), 2.33 (s, 6H, C*H*_*3*_), 2.33 (br. m, 4H, C*H*(CH_3_)_2_), 2.09 (s, 6H, C*H*_Ad_), 1.60–1.43 (m, 24H, C*H*_2,Ad_, CH(C*H*_*3*_)_2_), 1.41–1.21 (m, 12H, CH(C*H*_*3*_)_2_). ^13^C{^1^H} NMR (101 MHz, C_6_D_6_, in ppm) δ = 160.9 (*C*_Ar_), 141.5 (*C*_Ar_), 133.0 (*C*H_Ar_), 131.4 (*C*H_Ar_), 121.0 (*C*_Ar_), 119.0 (*C*H_Ar_), 70.5 (DME-*C*H_2_), 59.3 (DME-*C*H_3_), 52.5 (C_Ad_), 43.3 (*C*H_2,Ad_), 37.0 (*C*H_2,Ad_), 30.6 (*C*H(CH_3_)_2_), 30.2 (*C*H_Ad_), 20.8 (*C*H_3_), 19.3 (CH(*C*H_3_)_2_); ^31^P{^1^H} NMR (162 MHz, C_6_D_6_, in ppm) δ = 8.86 (s, 2P); ^31^P NMR (162 MHz, C_6_D_6_, in ppm) δ = 8.63 (s, 2P). ^7^Li{^1^H} NMR (156 MHz, C_6_D_6_, in ppm) δ = 0.60 (s, 1Li). ^7^Li NMR (156 MHz, C_6_D_6_, in ppm) δ = 0.60 (s, 1Li). Elemental analysis (%) calcd for C_50_H_80_Cl_2_LaLiN_2_O_2_P_2_: C, 58.88; H, 7.91; N, 2.75; found: C, 58.92; H, 8.10; N, 2.71. UV/vis/NIR: λ_max_ = 327 (ε = 55620 L mol^–1^ cm^–1^), 404 (ε = 23700 L mol^–1^ cm^–1^). IR (cm^–1^): 2906, 2867, 2848, 1599, 1509, 1456, 1384, 1356, 1305, 1288, 1278, 1256, 1235, 1213, 1153, 1125, 1080, 1023, 980, 935, 872, 827, 804, 778, 708, 688, 661, 645, 610, 531, 474, 439, 420.

##### Preparation of **4b**

From **LiPN**^**3,5CF3**^ (2.0 equiv, 227 μmol, 100 mg) and LaCl_3_(thf)_1.2_ (1.0 equiv, 113 μmol, 37.4 mg). 37 mg (28.1%) of an off-white solid. ^1^H NMR (400 MHz, C_6_D_6_, in ppm) δ = 7.82 (s, 4H, C*H*_Ar_), 7.42 (s, 2H, C*H*_Ar_), 6.85 (m, 4H, C*H*_Ar_), 6.79 (dd, *J* = 8.2, 4.4 Hz, 2H, C*H*_Ar_), 2.75 (s, 6H, DME-C*H*_*3*_), 2.53 (s, 4H, DME-C*H*_*2*_), 2.14–2.03 (m, 4H, C*H*(CH_3_)_2_), 2.07 (s, 6H, C*H*_*3*_), 1.29 (dd, *J* = 15.9, 6.9 Hz, 12H, CH(C*H*_*3*_)_2_), 0.82 (dd, *J* = 13.2, 7.0 Hz, 12H, CH(C*H*_*3*_)_2_); ^13^C{^1^H} NMR (101 MHz, C_6_D_6_, in ppm) δ = 159.9 (*C*_Ar_), 154.1 (*C*_Ar_), 134.6 (*C*_Ar_), 133.3 (*C*_Ar_), 133.2 (*C*H_Ar_), 129.6 (*C*_Ar_), 124.6 (d, *J* = 273.0 Hz, *C*_Ar_), 122.0 (*C*_Ar_), 120.8 (*C*H_Ar_), 118.9 (*C*H_Ar_), 69.7 (DME-*C*H_2_), 58.9 (DME-*C*H_3_), 24.9 (*C*H(CH_3_)_2_), 20.7 (*C*H_3_), 19.6 (CH(*C*H_3_)_2_), 18.7 (CH(*C*H_3_)_2_); ^31^P{^1^H} NMR (162 MHz, C_6_D_6_, in ppm) δ = 10.22 (s, 2P); ^31^P NMR (162 MHz, C_6_D_6_, in ppm) δ = 10.33 (s, 2P); ^19^F{^1^H} NMR (377 MHz, C_6_D_6_, in ppm) δ = −62.68 (s, 6F); ^19^F NMR (377 MHz, C_6_D_6_, in ppm) δ = −62.68 (s, 6F); ^7^Li{1H} NMR (156 MHz, C_6_D_6_, in ppm) δ = −0.12 (s, 1Li); ^7^Li NMR (156 MHz, C_6_D_6_, in ppm) δ = −0.12 (s, 1Li). Elemental analysis (%) calcd for C_54_H_76_Cl_2_F_12_LaLiN_2_O_6_P_4_: C, 47.84; H, 5.65; N, 2.07; found: C, 43.90; H, 5.02; N, 1.95. or C, 53.08; H, 5.40; N, 2.77. (Despite numerous attempts, due to the high fluorine content of complex **4b,** no better elemental analysis could be obtained.) UV/vis/NIR: λ_max_ = 326 (ε = 86550 L mol^–1^ cm^–1^), 360 (ε = 93050 L mol^–1^ cm^–1^). IR (cm^–1^): 2955, 2928, 2871, 1590, 1466, 1368, 1272, 1164, 1119, 1080, 1029, 984, 949, 868, 849, 827, 723, 702, 680, 657, 606, 498, 476, 449.

##### Preparation of **4c**

From **LiPN**^**OMe**^ (2.0 equiv, 298 μmol, 100 mg) and LaCl_3_(thf)_1.2_ (1.0 equiv, 149 μmol, 49.2 mg). 95 mg (66.1%) of an off-white solid. ^1^H NMR (400 MHz, C_6_D_6_, in ppm) δ = 6.85 (m, 8H, C*H*_Ar_), 6.72–6.42 (m, 6H, C*H*_Ar_), 4.35 (s, 6H, OC*H*_*3*_), 3.09 (s, 4H, DME-C*H*_*2*_), 3.08 (s, 6H, DME-C*H*_*3*_), 2.25 (s, 6H; C*H*_*3*_), 2.08 (s, 4H, C*H*(CH_3_)_2_), 1.17–1.05 (br. s, 12H, CH(C*H*_*3*_)_2_), 1.05–0.95 (br. s, 12H, CH(C*H*_3_)_2_; ^13^C{^1^H} NMR (101 MHz, C_6_D_6_, in ppm) δ = 154.9 (*C*_Ar_), 151.3 (*C*_Ar_), 147.7 (*C*_Ar_), 132.0 (*C*H_Ar_), 131.0 (*C*H_Ar_), 123.5 (*C*H_Ar_), 113.2 (*C*H_Ar_), 110.0 (*C*H_Ar_), 71.2 (DME-*C*H_2_), 58.4 (DME-*C*H_3_), 25.9 (*C*H(CH_3_)_2_), 21.3 (*C*H_3_), 19.5 (CH(*C*H_3_)_2_), 19.1 (CH(*C*H_3_)_2_); ^31^P{^1^H} NMR (162 MHz, C_6_D_6_, in ppm) δ = 5.78 (s, 2P); ^31^P NMR (162 MHz, C_6_D_6_, in ppm) δ = 5.84 (s, 2P); ^7^Li{^1^H} NMR (156 MHz, C_6_D_6_, in ppm) δ = 0.88 (s, 1Li); ^7^Li NMR (156 MHz, C_6_D_6_, in ppm) δ = 0.88 (s, 1Li). Elemental analysis (%) calcd for C_44_H_64_Cl_2_LaLiN_2_O_4_P_2_: C, 54.84; H, 6.69; N, 2.91; found: C, 52.87; H, 7.08; N, 2.46. (despite numerous attempts, due to the high reactivity of complex **4c** toward moisture and air, no better elemental analysis could be obtained.) UV/vis/NIR: λ_max_ = 326 (ε = 48280 L mol^–1^ cm^–1^), 357 (ε = 51470 L mol^–1^ cm^–1^). IR (cm^–1^): 2930, 2869, 2838, 1586, 1513, 1486, 1466, 1445, 1388, 1364, 1333, 1288, 1274, 1241, 1213, 1196, 1170, 1156, 1141, 1115, 1076, 1045, 1009, 974, 888, 857, 839, 825, 796, 725, 670, 659, 588, 498, 451.

##### Preparation of **4d**

From **LiPN**^**3,5Me**^ (2.0 equiv, 600 μmol, 200 mg) and LaCl_3_(thf)1.2 (1.0 equiv, 300 μmol, 99.0 mg). 208 mg (60.8%) of an off-white solid. ^1^H NMR (400 MHz, C_6_D_6_, in ppm) δ = 7.19 (s, 4H, C*H*_Ar_), 7.15 (s, 2H, C*H*_Ar_), 6.99 (d, *J* = 8.4 Hz, 2H, C*H*_Ar_), 6.95 (d, *J* = 4.9 Hz, 2H, C*H*_Ar_), 6.50 (s, 2H, C*H*_Ar_) 2.91 (s, 6H, DME-C*H*_*3*_), 2.73 (s, 4H, DME-C*H*_*2*_), 2.38 (s, 12H, C*H*_*3*_), 2.17 (s, 6H, C*H*_*3*_), 1.44 (m, 12H, CH(C*H*_*3*_)_2_), 1.31–1.17 (m, 4H, C*H*(CH_3_)_2_), 1.44 (br. s, 12H, CH(C*H*_*3*_)_2_); ^13^C{^1^H} NMR (101 MHz, C_6_D_6_, in ppm) δ = 161.7 (*C*_Ar_), 141.1 (*C*_Ar_), 132.8 (*C*H_Ar_), 132.6 (*C*H_Ar_), 126.1 (*C*_Ar_), 121.4 (*C*H_Ar_), 121.0 (*C*_Ar_), 119.7 (*C*H_Ar_), 117.8 (*C*_Ar_), 70.3 (DME-*C*H_3_), 59.0 (DME-*C*H_2_), 21.9 (*C*H_3_), 20.8 (*C*H_3_), 20.1 (*C*H(CH_3_)_2_), 19.2 (CH(*C*H_3_)_2_); ^31^P NMR (162 MHz, C_6_D_6_, in ppm) δ = 7.82 (s, 2P); ^31^P{^1^H} NMR (162 MHz, C_6_D_6_, in ppm) δ = 7.80 (s, 2P); ^7^Li{^1^H} NMR (156 MHz, C_6_D_6_, in ppm) δ = 0.03 (s, 1Li); ^7^Li NMR (156 MHz, C_6_D_6_, in ppm) δ = 0.03 (s, 1Li). Elemental analysis (%) calcd for C_54_H_88_Cl_2_LaLiN_2_O_6_P_2_: C, 56.89; H, 7.78; N, 2.46; found: C, 56.53; H, 7.12; N, 2.74. UV/vis/NIR: λ_max_ = 326 (ε = 42000 L mol^–1^ cm^–1^), 399 (ε = 8960 L mol^–1^ cm^–1^). IR (cm^–1^): 2952, 2865, 1597, 1582, 1511, 1470, 1388, 1331, 1305, 1276, 1243, 1156, 1104, 1062, 882, 855, 819, 690, 674, 476.

##### Preparation of **4f**

From **LiPN**^**Tol**^ (2.0 equiv, 626 μmol, 200 mg) and LaCl_3_(thf)_1.2_ (1.0 equiv, 313 μmol, 103 mg). 202 mg (58.0%) of an off-white solid. ^1^H NMR (400 MHz, C_6_D_6_, in ppm) δ = 7.47 (d, *J* = 7.5 Hz, 4H, C*H*_Ar_), 7.25 (d, *J* = 8.0 Hz, 4H, C*H*_Ar_), 7.11 (dd, *J* = 8.3, 4.6 Hz, 2H, C*H*_Ar_), 6.98 (d, *J* = 8.4 Hz, 2H, C*H*_Ar_), 6.93 (dd, *J* = 4.7, 1.6 Hz, 2H, C*H*_Ar_), 3.00 (s, 18H, DME-C*H*_*3*_), 2.98 (s, 12H, DME-C*H*_*2*_), 2.18 (s, 6H, C*H*_*3*_), 2.17 (s, 6H, C*H*_*3*_), 1.42 (dd, *J* = 15.3, 6.9 Hz, 12H, CH(C*H*_*3*_)_2_), 1.33–1.14 (m, 4H, C*H*(CH_3_)_2_), 1.01 (dd, *J* = 12.8, 6.9 Hz, 12H, CH(C*H*_*3*_)_2_); ^13^C{^1^H} NMR (101 MHz, C_6_D_6_, in ppm) δ = 162.0 (*C*_Ar_), 161.8 (*C*_Ar_), 148.8 (*C*_Ar_), 132.9 (*C*H_Ar_), 132.7 (*C*H_Ar_), 132.3 (*C*H_Ar_), 126.0 (*C*_Ar_), 120.9 (*C*H_Ar_), 119.1 (*C*H_Ar_), 119.1 (*C*H_Ar_), 71.1 (DME-*C*H_3_), 58.8 (DME-*C*H_2_), 25.3 (br. *C*H_3_), 22.4 (*C*H(CH_3_)_2_) 20.8 (*C*H_3_), 20.1 (CH(*C*H_3_)_2_), 19.2 (CH(*C*H_3_)_2_); ^31^P{^1^H} NMR (162 MHz, C_6_D_6_, in ppm) δ = 8.36 (s, 2P); ^31^P NMR (162 MHz, C_6_D_6_, in ppm) δ = 8.36 (s, 2P); ^7^Li{^1^H} NMR (156 MHz, C_6_D_6_, in ppm) δ = 0.06 (s, 1Li); ^7^Li NMR (156 MHz, C_6_D_6_, in ppm) δ = 0.05 (s, 1Li). Elemental analysis (%) calcd for C_52_H_84_Cl_2_LaLiN_2_O_6_P_2_: C, 56.17; H, 7.61; N, 2.52; found: C, 57.94; H, 7.11; N, 2.84. (Despite numerous attempts, due to the high reactivity of complex **4f** toward moisture and air, no better elemental analysis could be obtained.) UV/vis/NIR: λ_max_ = 326 (ε = 50690 L mol^–1^ cm^–1^), 358 (ε = 52870 L mol^–1^ cm^–1^), 406 (ε = 38520 L mol^–1^ cm^–1^). IR (cm^–1^): 2952, 2922, 2869, 1599, 1494, 1470, 1411, 1390, 1298, 1270, 1245, 1194, 1174, 1145, 1123, 1109, 1084, 1031, 896, 857, 845, 812, 796, 725, 706, 661, 612, 508, 492, 474, 445.

### X-ray Crystallography

Single crystals for X-ray diffraction experiments were measured at the analytical facility of the University of Innsbruck. All crystals were kept at 153(2) or 173(2) K throughout data collection. Data were collected using the APEXIV software package. Data refinement and reduction were performed with Bruker Saint (V8.34A). All structures were solved with SHELXT^63^ and refined using the OLEX 2 software package as a graphical interface, and structure refinement with ShelXL using full matrix least square minimization on F^2^.^64−66^ Strongly disordered solvent molecules were removed using the SQUEEZE operation.^67^ All nonhydrogen atoms were refined anisotropically, and hydrogen atoms were included at the geometrically calculated positions and refined using a riding model. All structures have been submitted to the CCDC and can be obtained under the numbers presented in the SI Tables S1, S2, and S3.

## Supporting Information Available

The Supporting Information is available free of charge via the Internet at http://pubs.acs.org and contains ^1^H, ^13^C, ^31^P, ^7^Li, ^19^F, and 2D NMR spectra of all new compounds, mass data for all air-stable compounds, UV–vis-NIR and IR spectra for all new compounds, crystallographic details, and more details concerning the %buried volume calculations^58,59^ (PDF)
