# Supplementary material for: Modular Synthesis of Monoanionic PN Ligands Leads to Unexpected Structural Diversity in Lanthanum Chemistry
Source: Inorg Chem. 2024 Oct 15;63(43):20448–61. doi: 10.1021/acs.inorgchem.4c02897 (PMC11523257; doi:10.1021/acs.inorgchem.4c02897)
Supplement: Supplementary file 1 — ic4c02897_si_001.pdf [file ic4c02897_si_001.pdf]

# - Supporting Information -

Modular Synthesis of Monoanionic PN Ligands Leads to Unexpected Structural Diversity in Lanthanum Chemistry

Benjamin Wittwer,[a] Florian Hett,[a] Michael Seidl,[a] Stephan Hohloch[a]*

Table of contents

NMR spectra 2

Mass spectra 224

UV-Vis-NIR spectra 236

IR spectra 247

Crystallographic details 254

Buried Volume 260

Literature 261

[a] University of Innsbruck, Institute of General, Inorganic and Theoretical Chemistry, Innrain 80 – 82, 6020 Innsbruck, Austria

NMR spectra

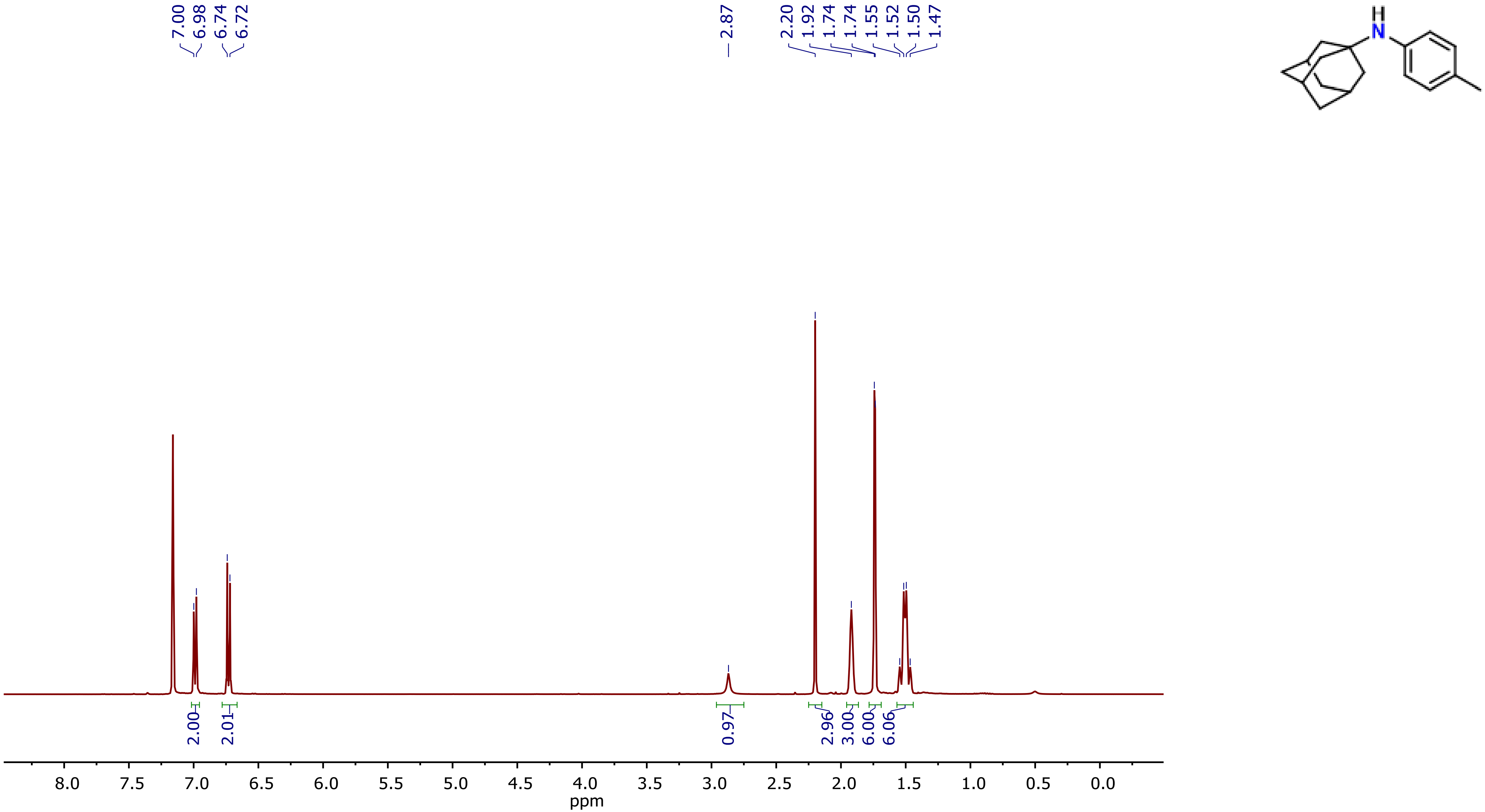

*Figure S 1: ^{1}H NMR spectrum of **1a** in C_6D_6 at 298 K.*

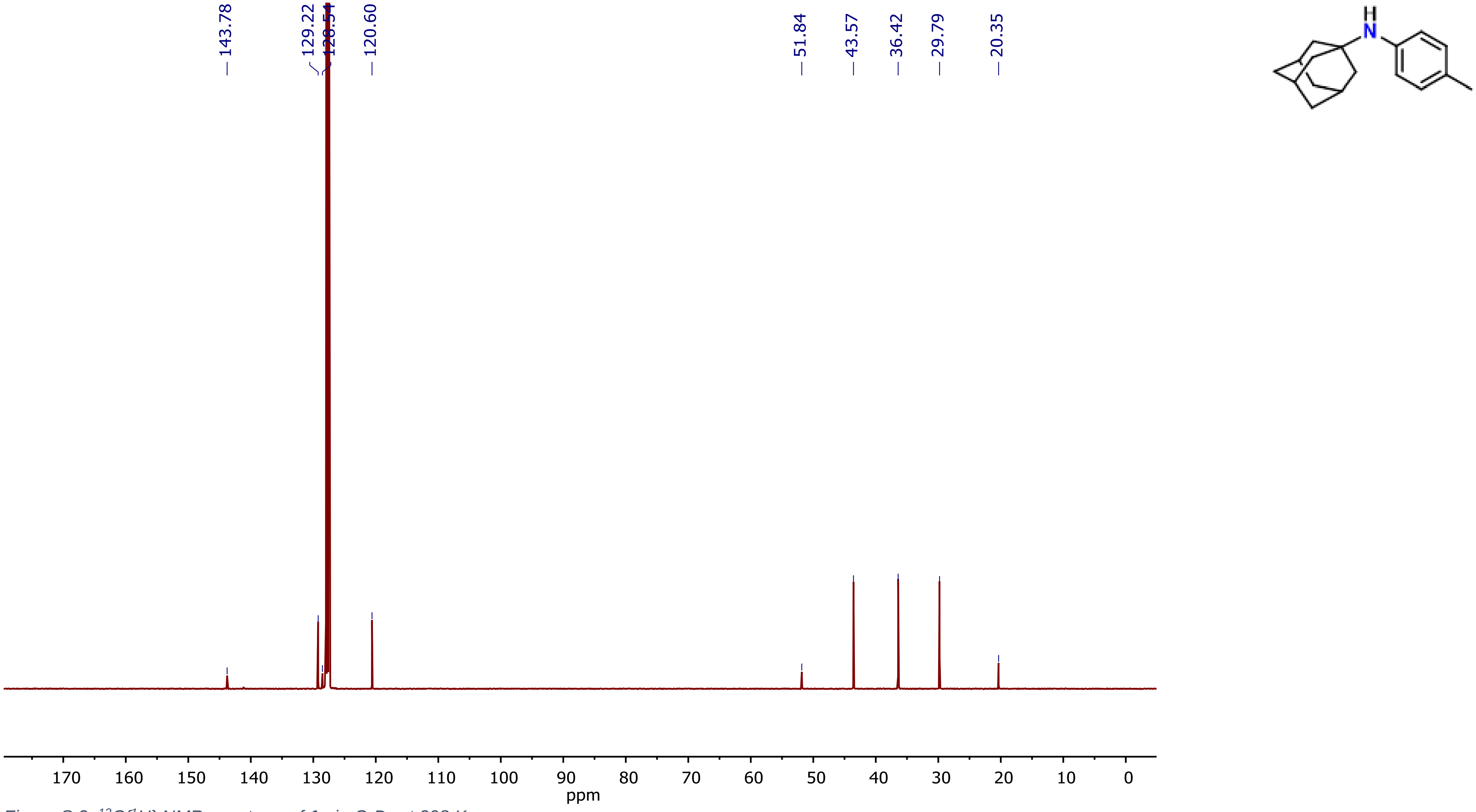

*Figure S 2: $^{13}C\{^{1}H\}$ NMR spectrum of **1a** in C_6D_6 at 298 K.*

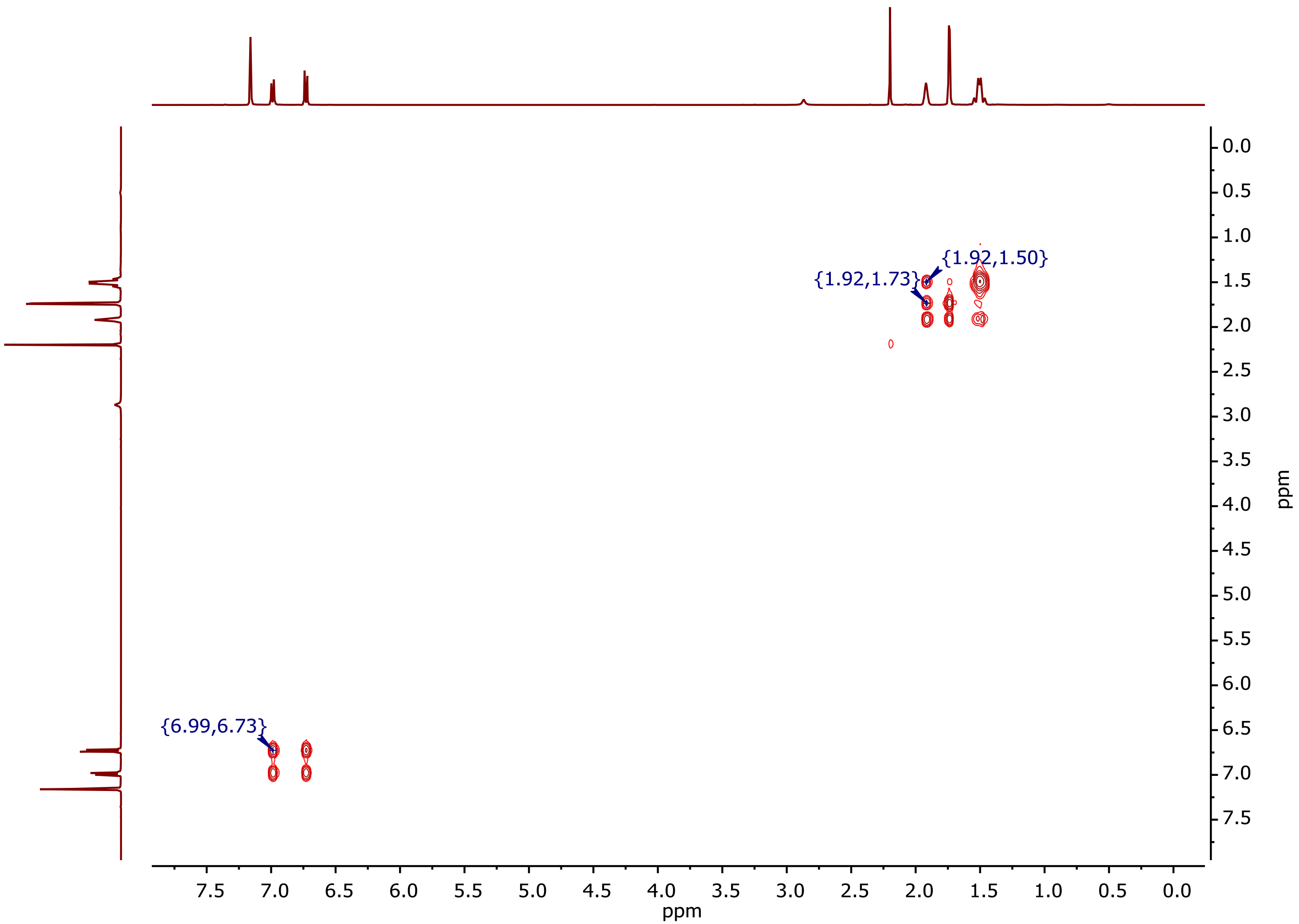

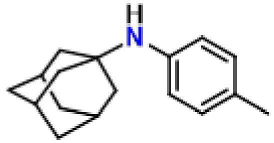

*Figure S 3: ^{1}H-^{1}H COSY NMR spectrum of **1a** in C_6D_6 at 298 K.*

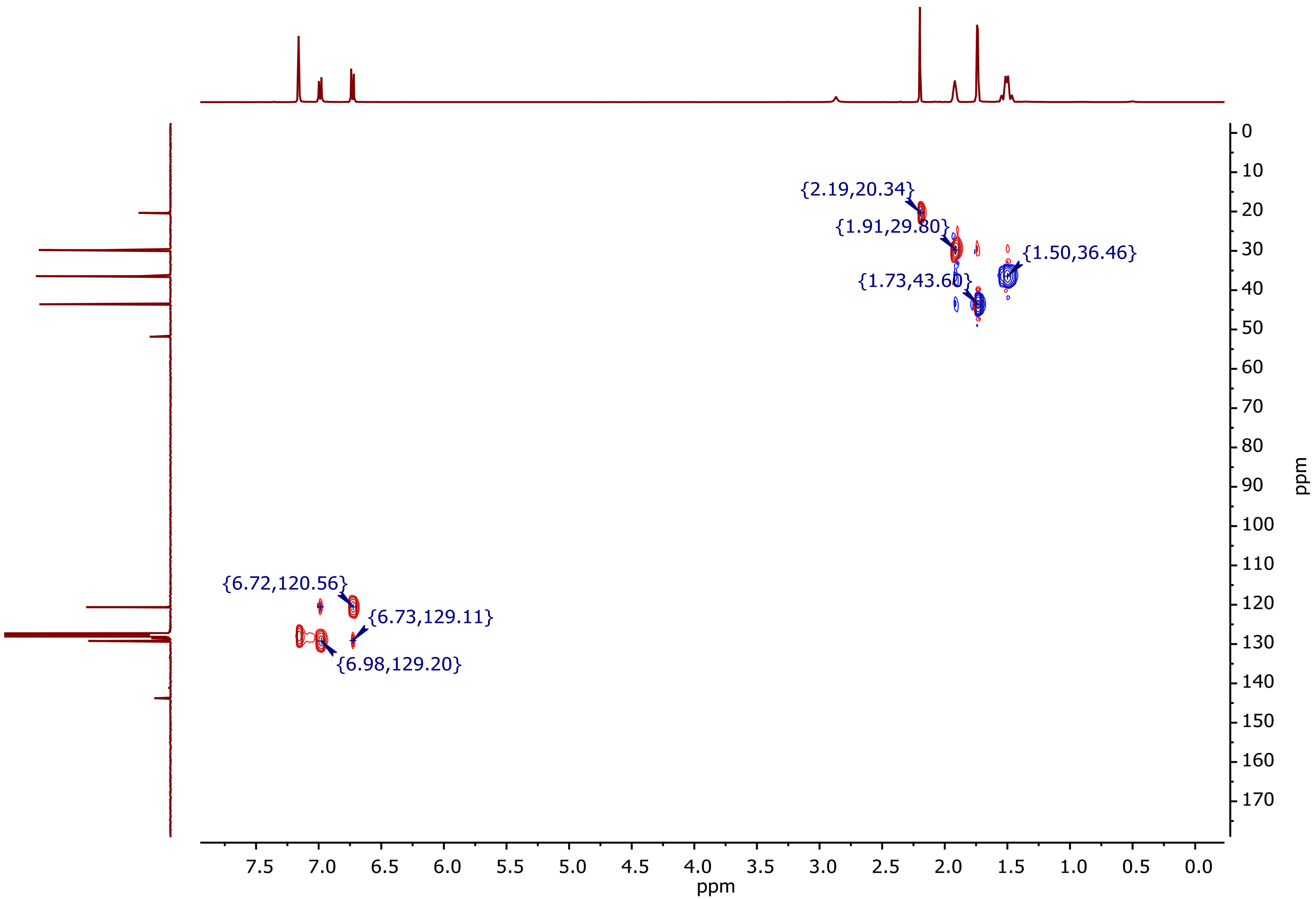

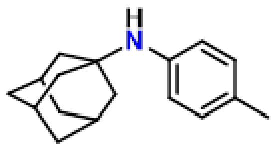

Figure S 4: ^{1}H-^{13}C HSQC NMR spectrum of **1a** in C_6D_6 at 298 K.

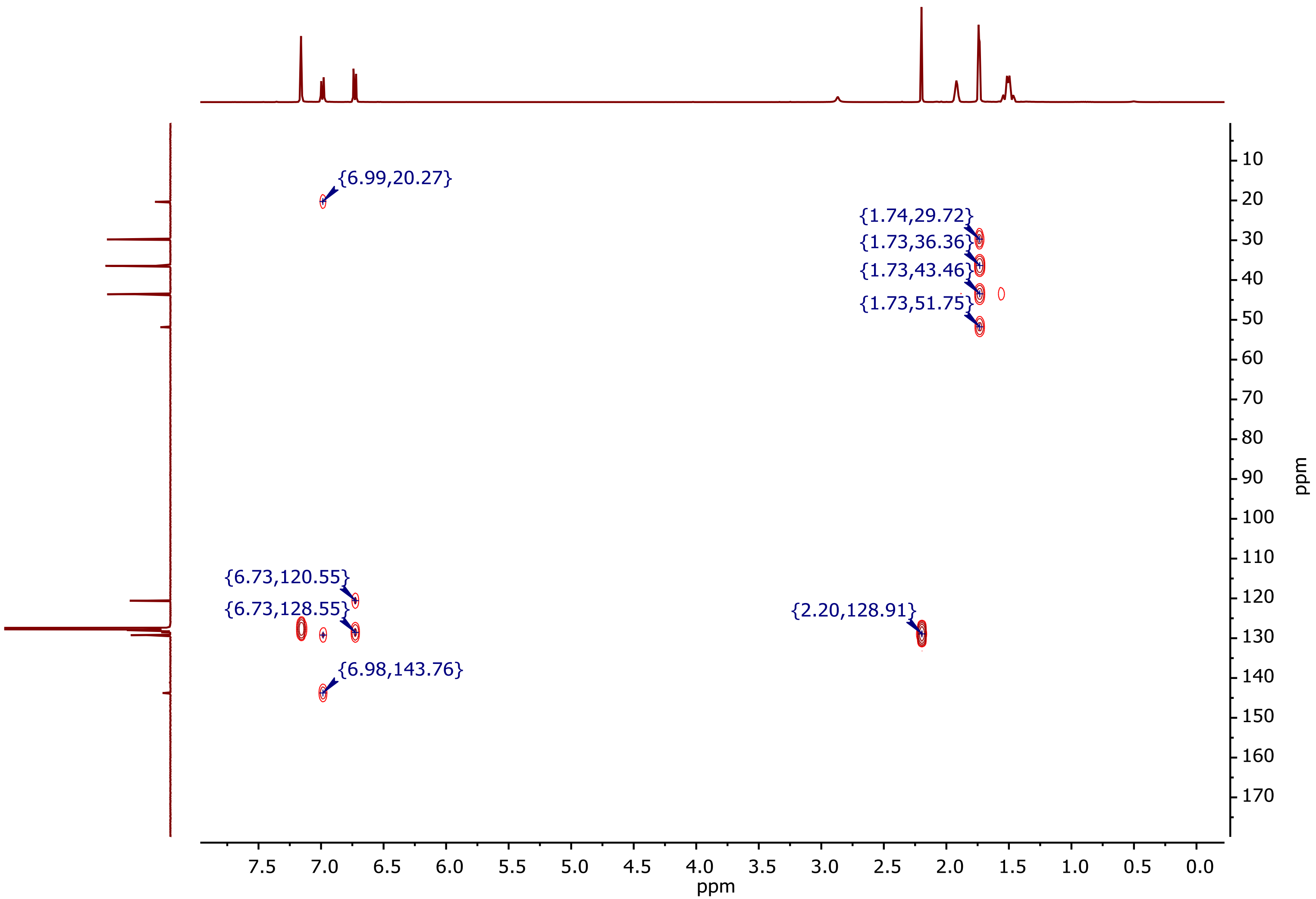

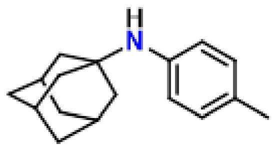

*Figure S 5: ^{1}H-^{13}C HMBC NMR spectrum of **1a** in C_6D_6 at 298 K.*

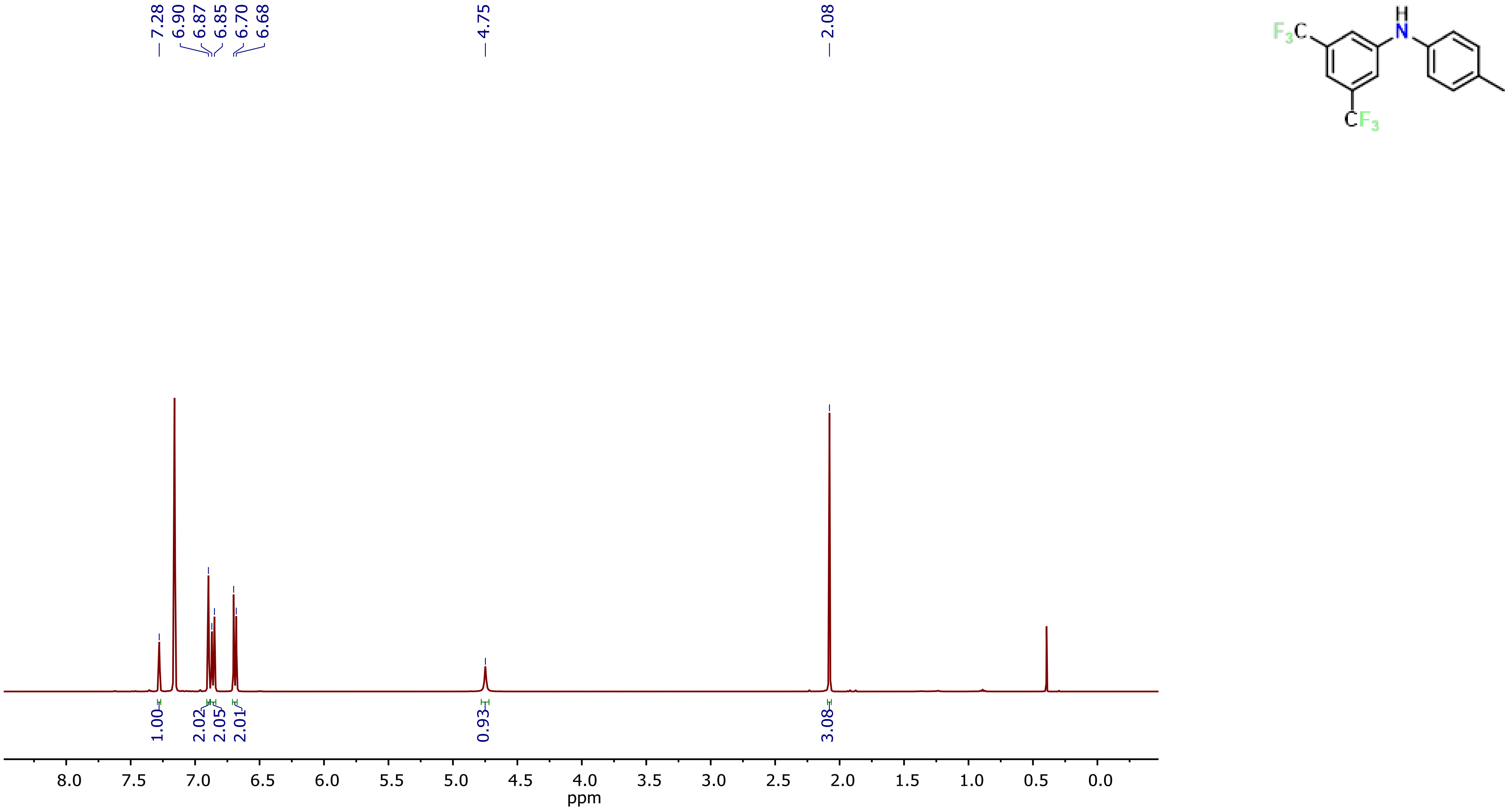

Figure S 6: ^{1}H NMR spectrum of ***1b*** *in C_6D_6 at 298 K.*

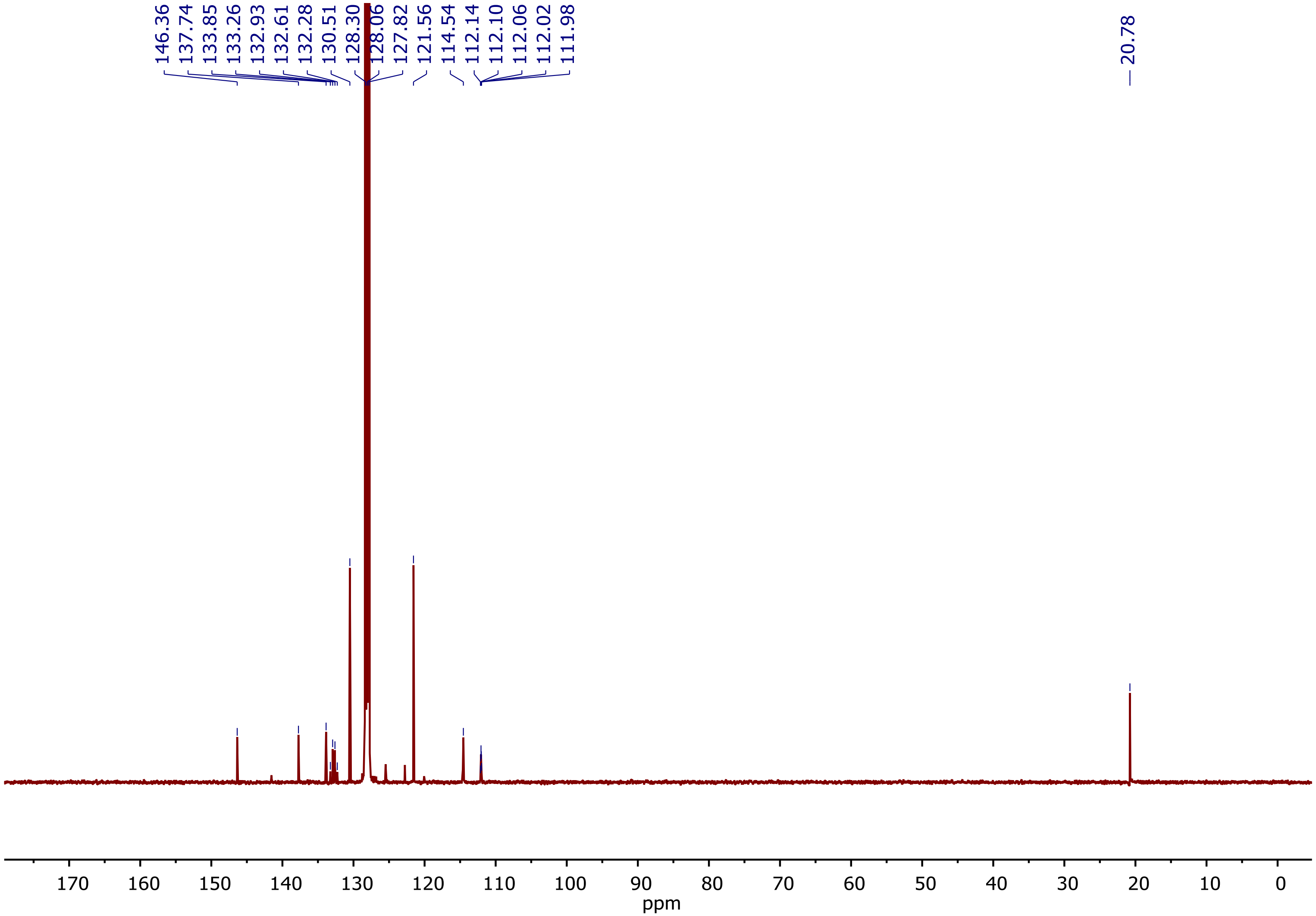

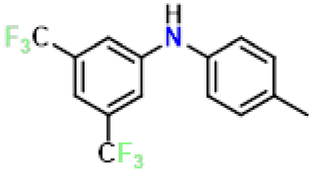

*Figure S 7: $^{13}C\{^1H\}$ NMR spectrum of **1b** in C_6D_6 at 298 K.*

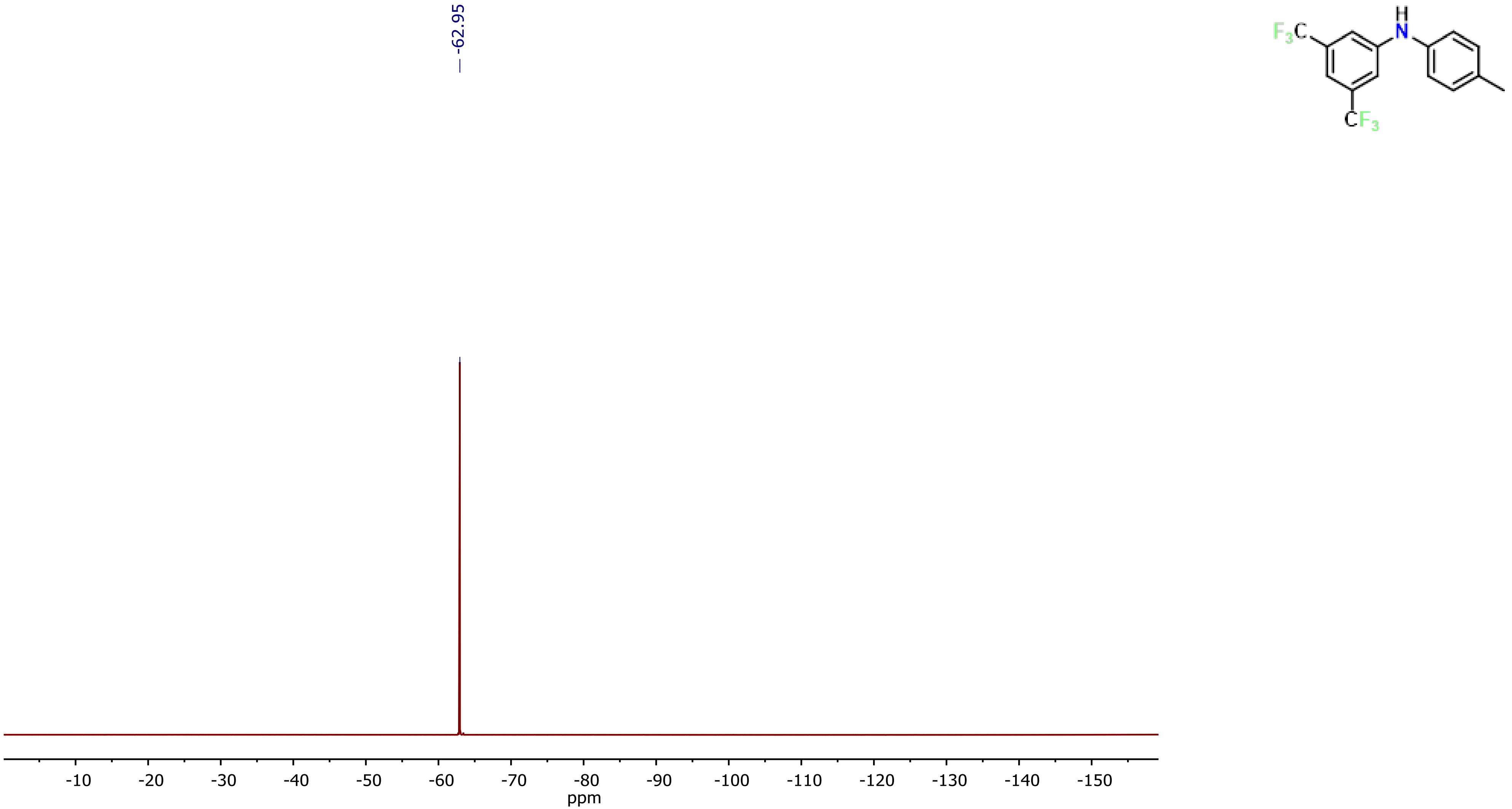

*Figure S 8: ^{19}F NMR spectrum of **1b** in C_6D_6 at 298 K.*

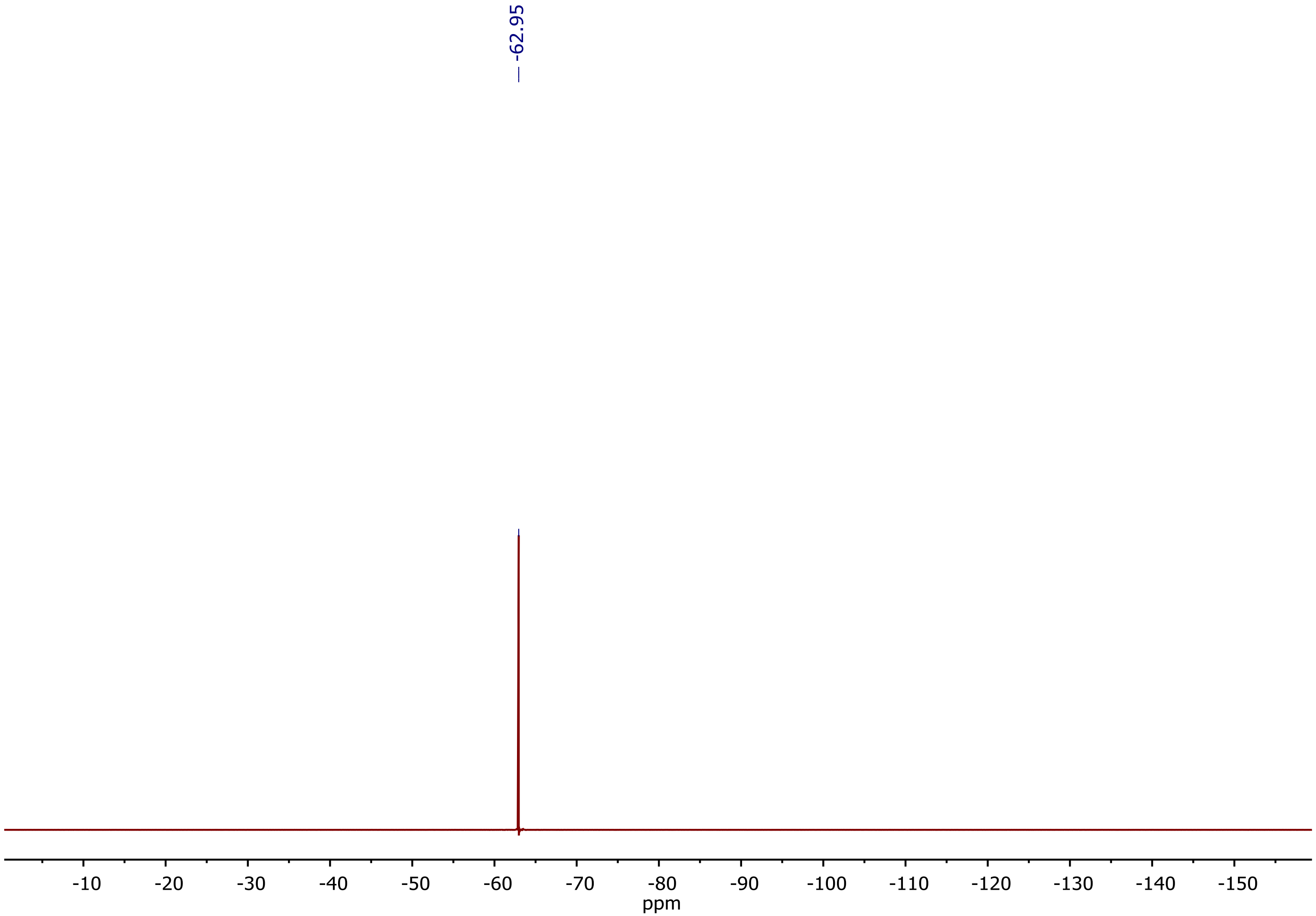

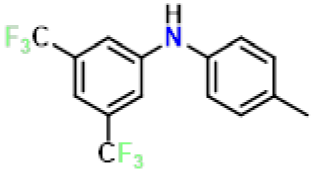

*Figure S 9: $^{19}F\{^1H\}$ NMR spectrum of **1b** in C_6D_6 at 298 K.*

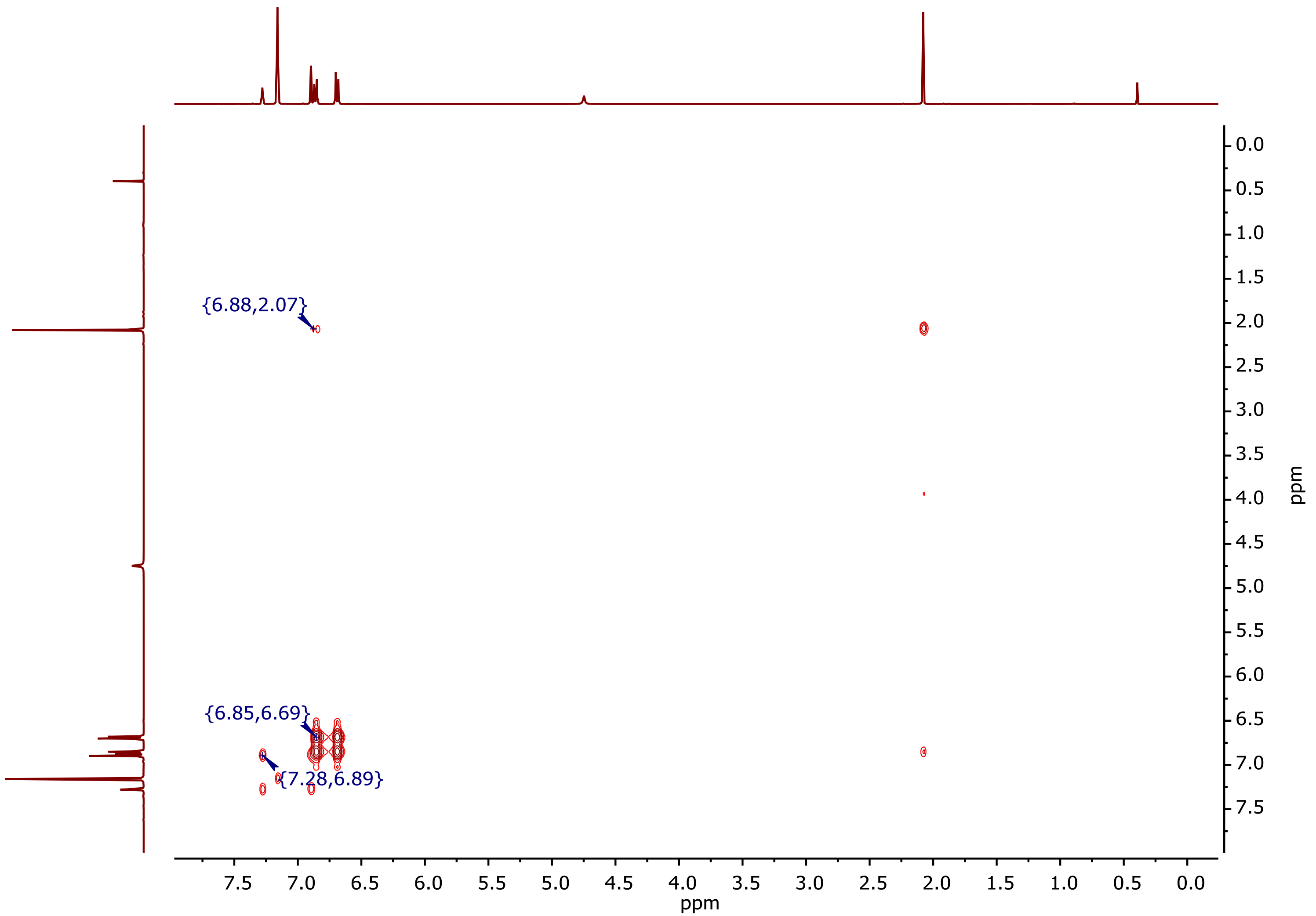

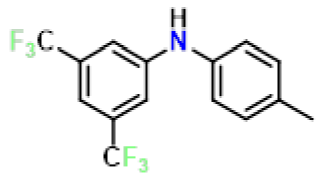

Figure S 10: ^{1}H-^{1}H COSY NMR spectrum of **1b** in C_6D_6 at 298 K.

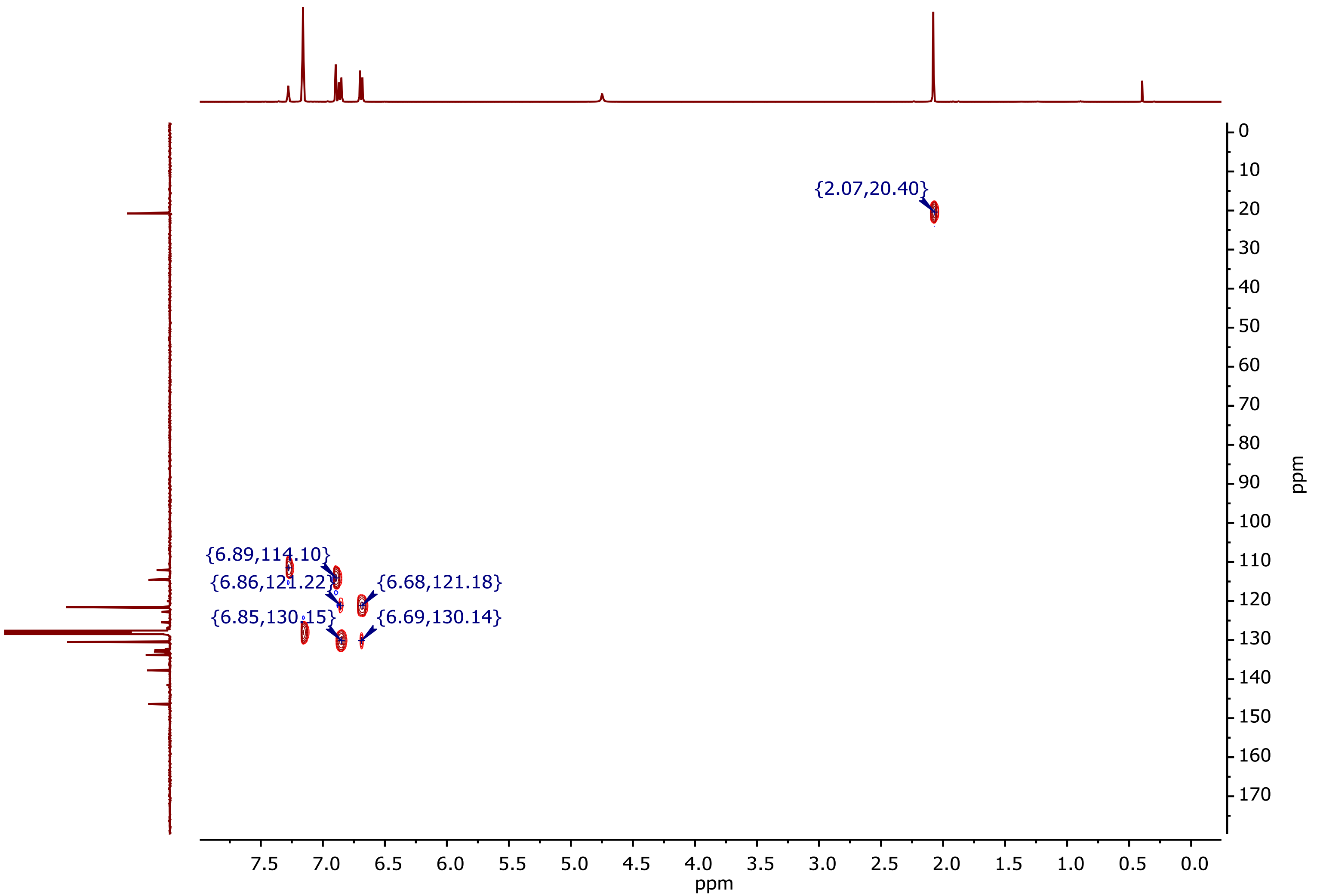

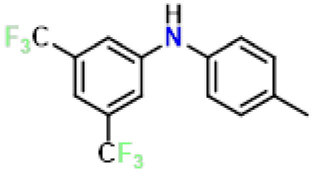

*Figure S 11: ^{1}H-^{13}C HSQC NMR spectrum of **1b** in C_6D_6 at 298 K.*

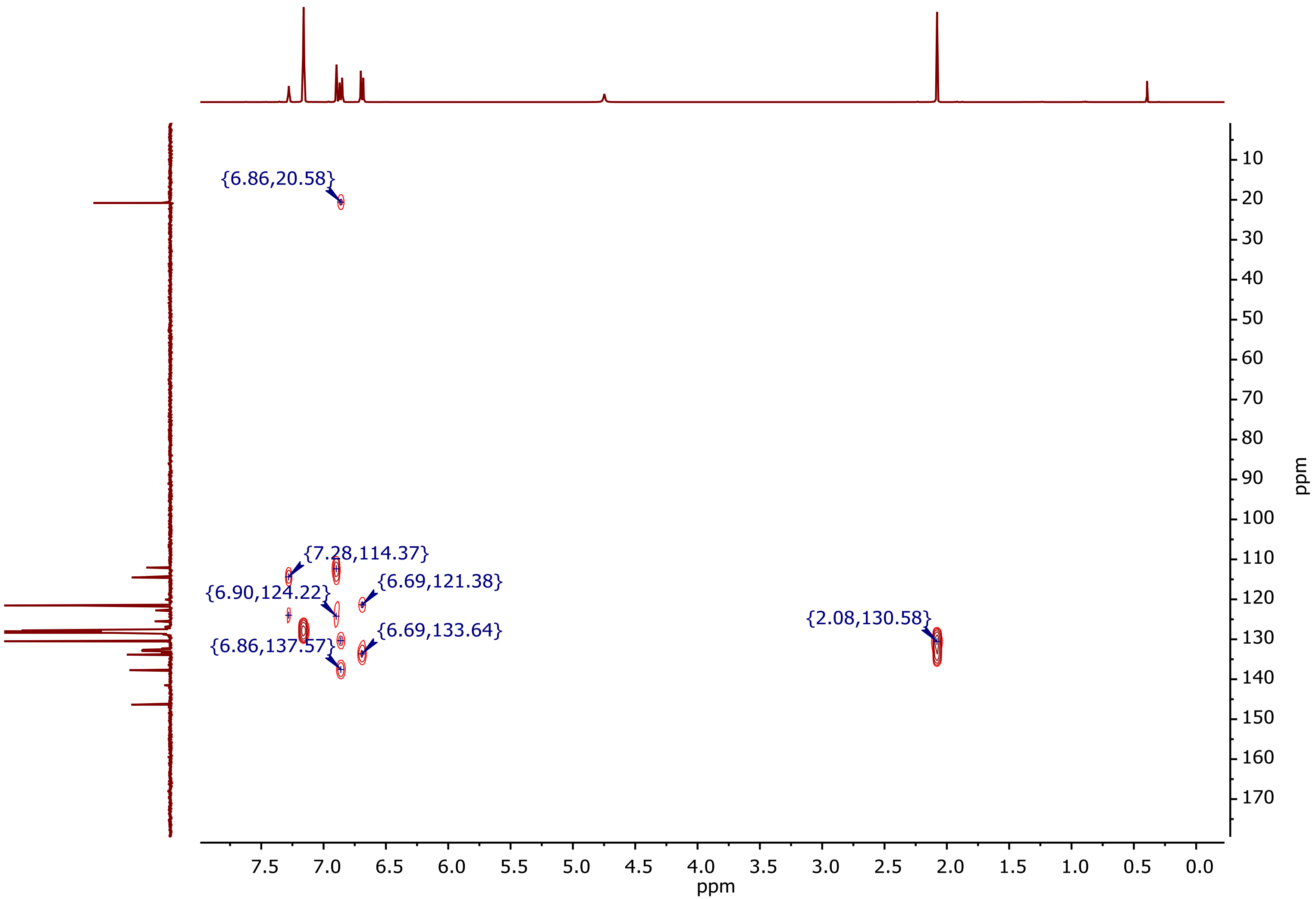

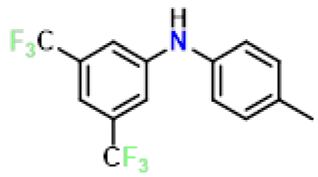

*Figure S 12: ^{1}H-^{13}C HMBC NMR spectrum of **1b** in C_6D_6 at 298 K.*

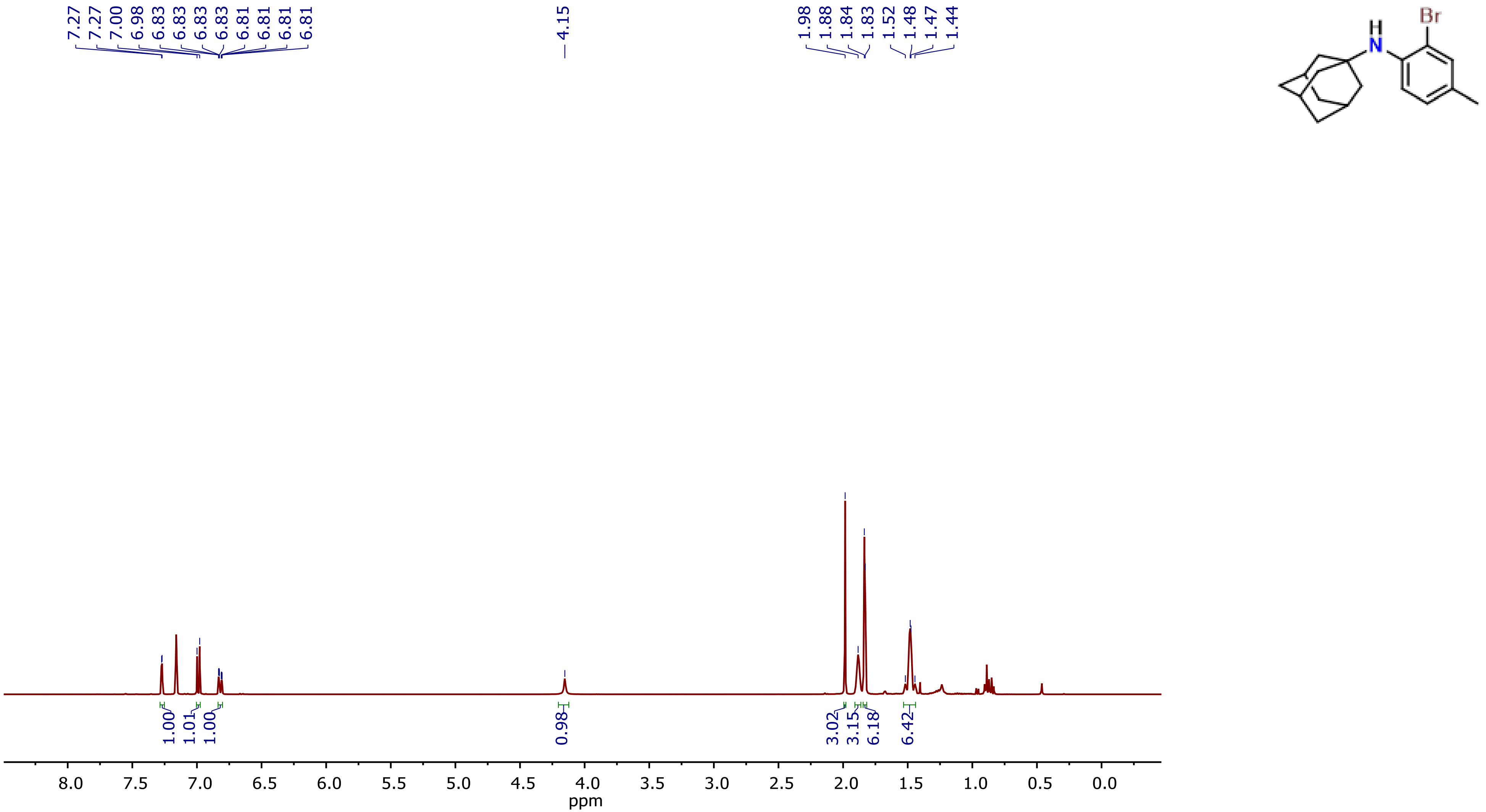

*Figure S 13: ¹H NMR spectrum of **2a** in C_6D_6 at 298 K.*

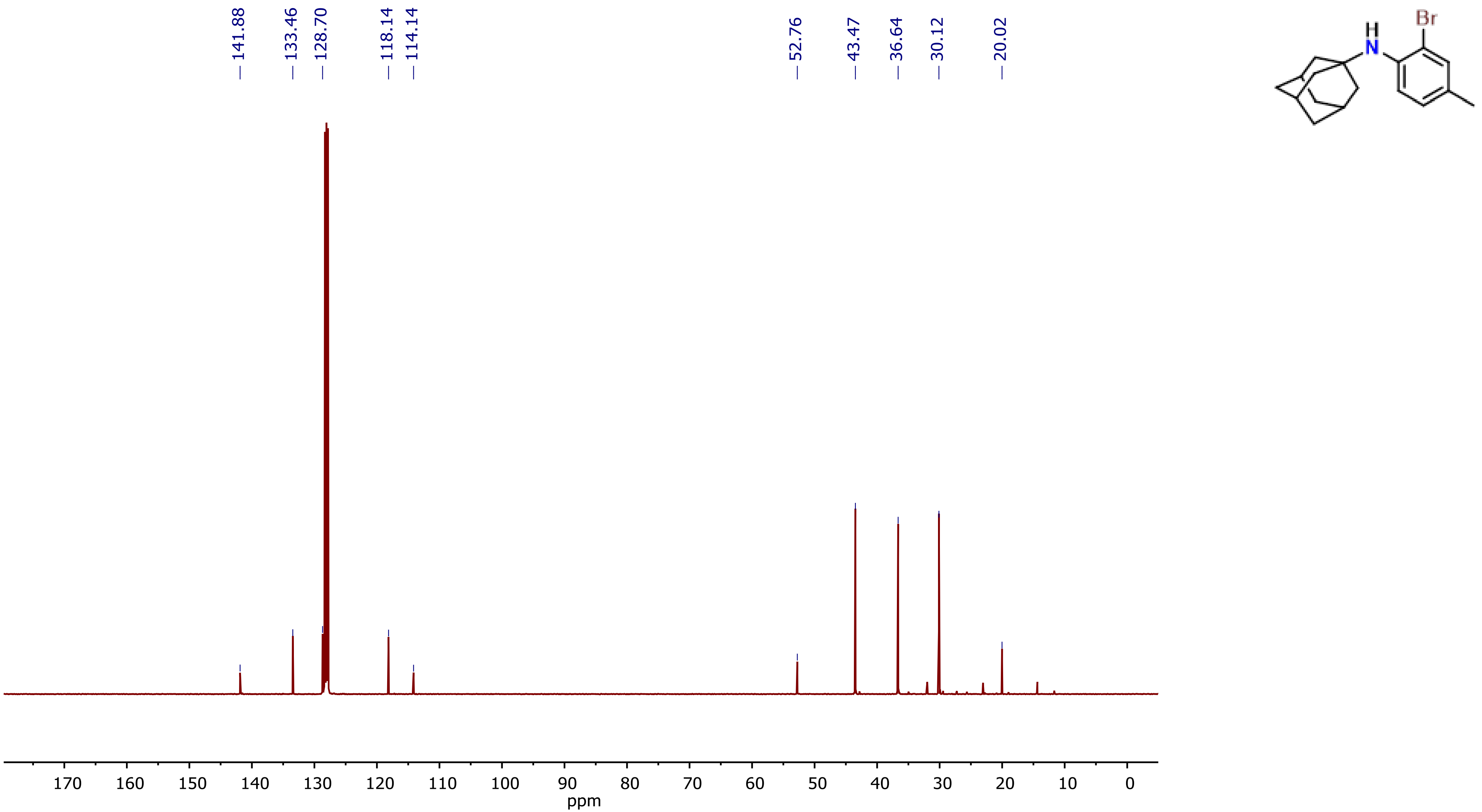

*Figure S 14: $^{13}C\{^1H\}$ NMR spectrum of **2a** in C_6D_6 at 298 K.*

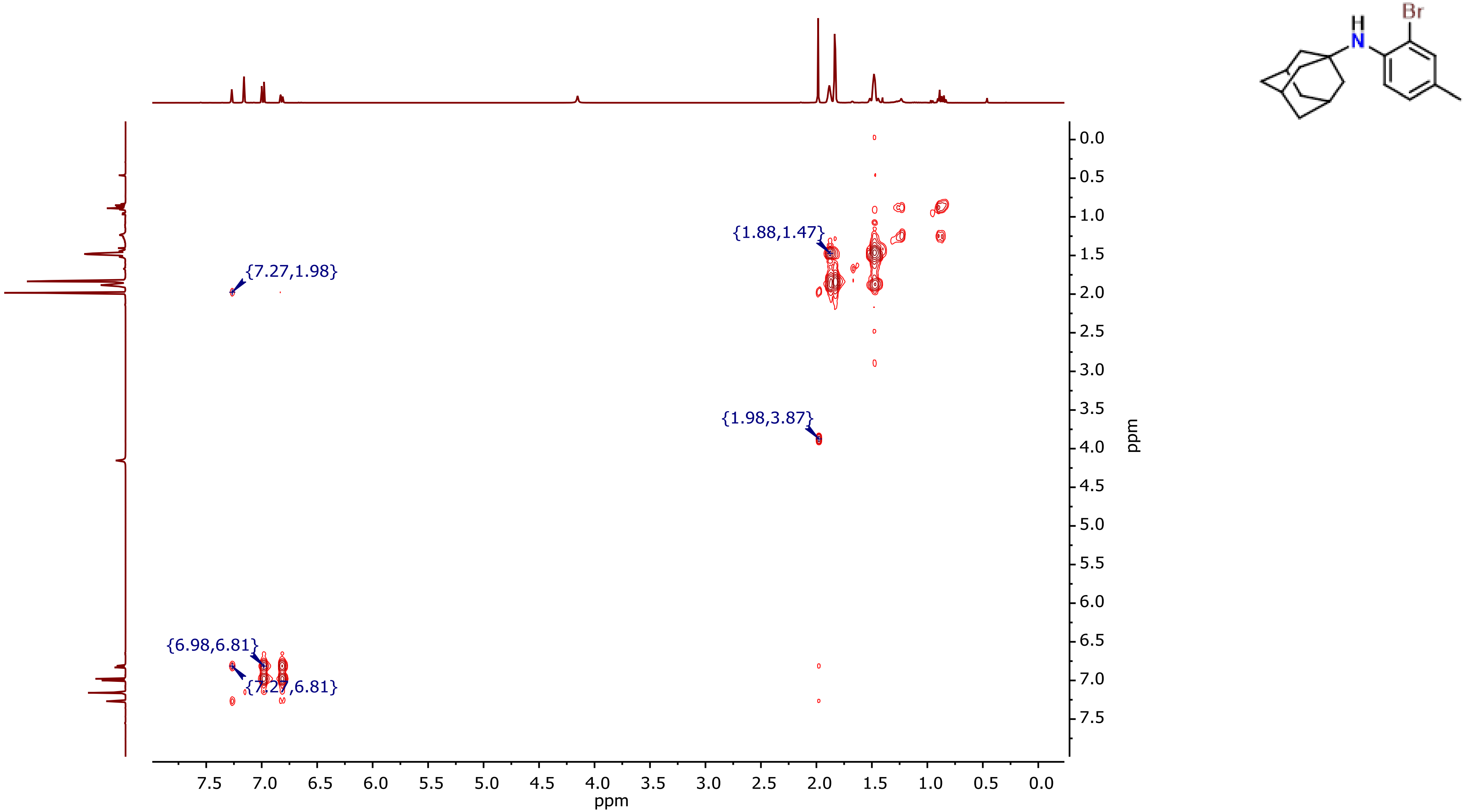

*Figure S 15: ^{1}H-^{1}H COSY NMR spectrum of **2a** in C_6D_6 at 298 K.*

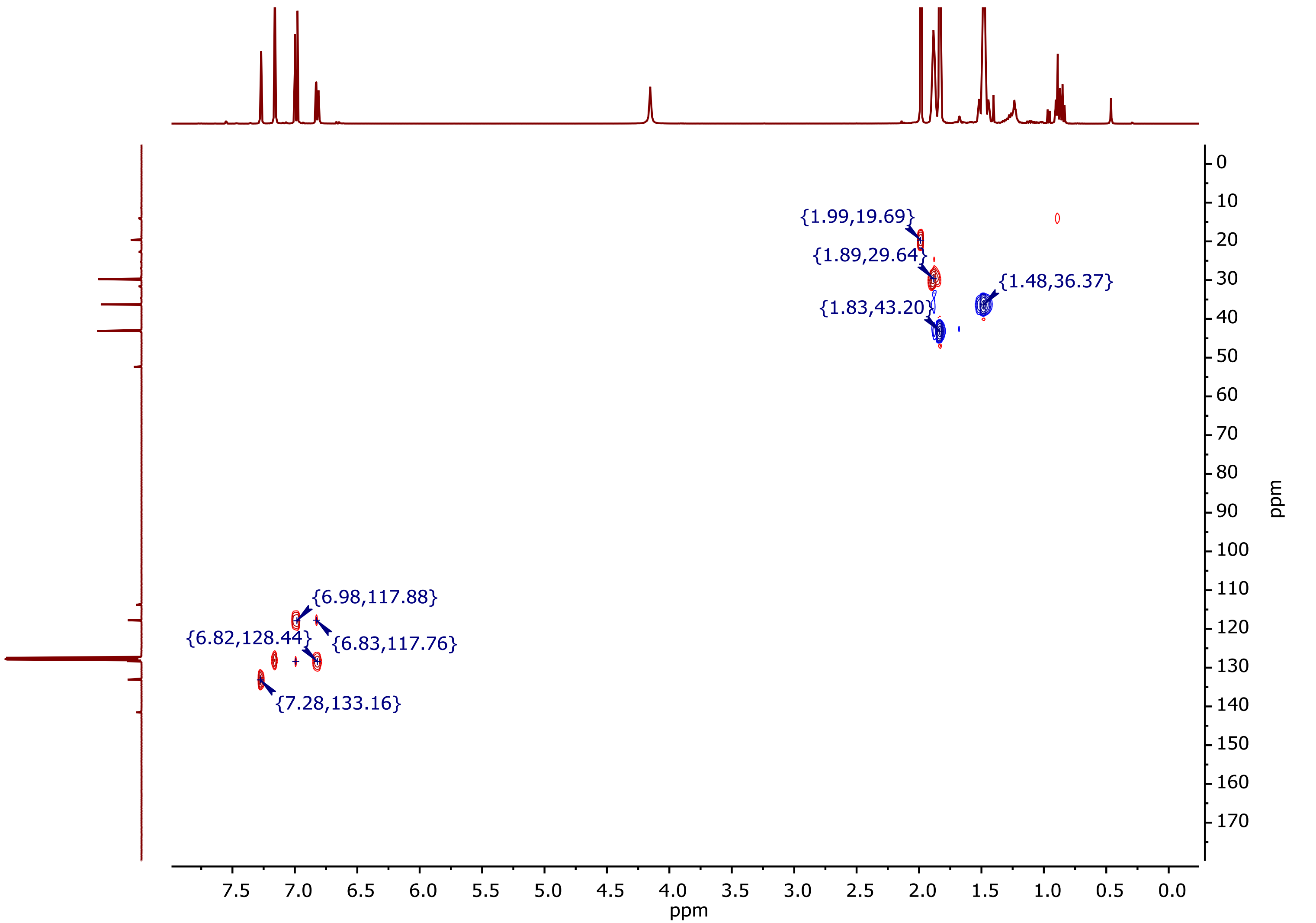

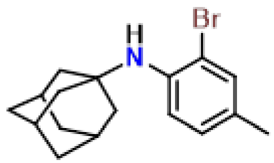

*Figure S 16: ^{1}H-^{13}C HSQC NMR spectrum of **2a** in C_6D_6 at 298 K.*

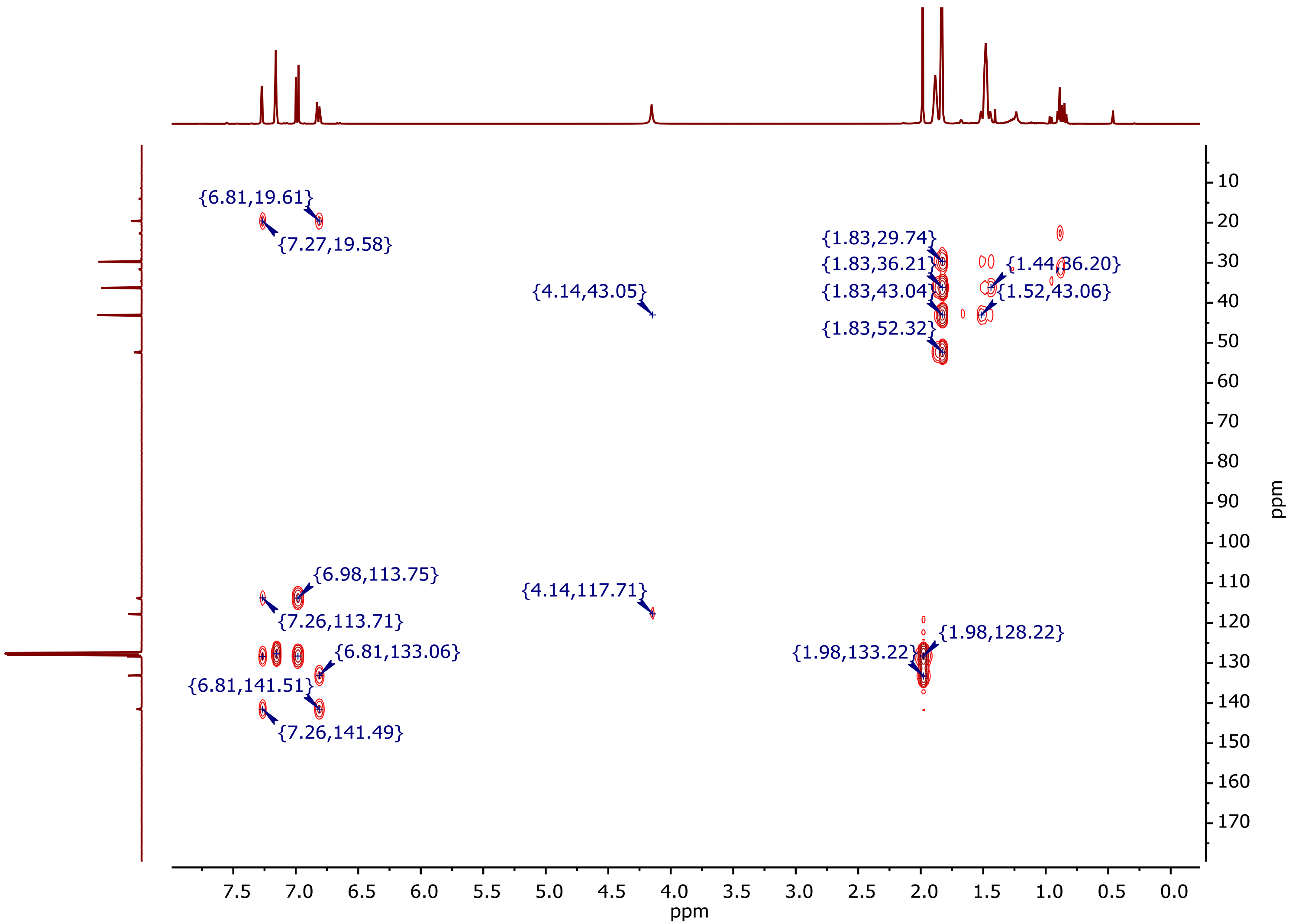

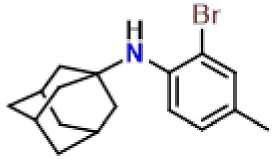

Figure S 17: ^{1}H-^{13}C HMBC NMR spectrum of **2a** in C_6D_6 at 298 K.

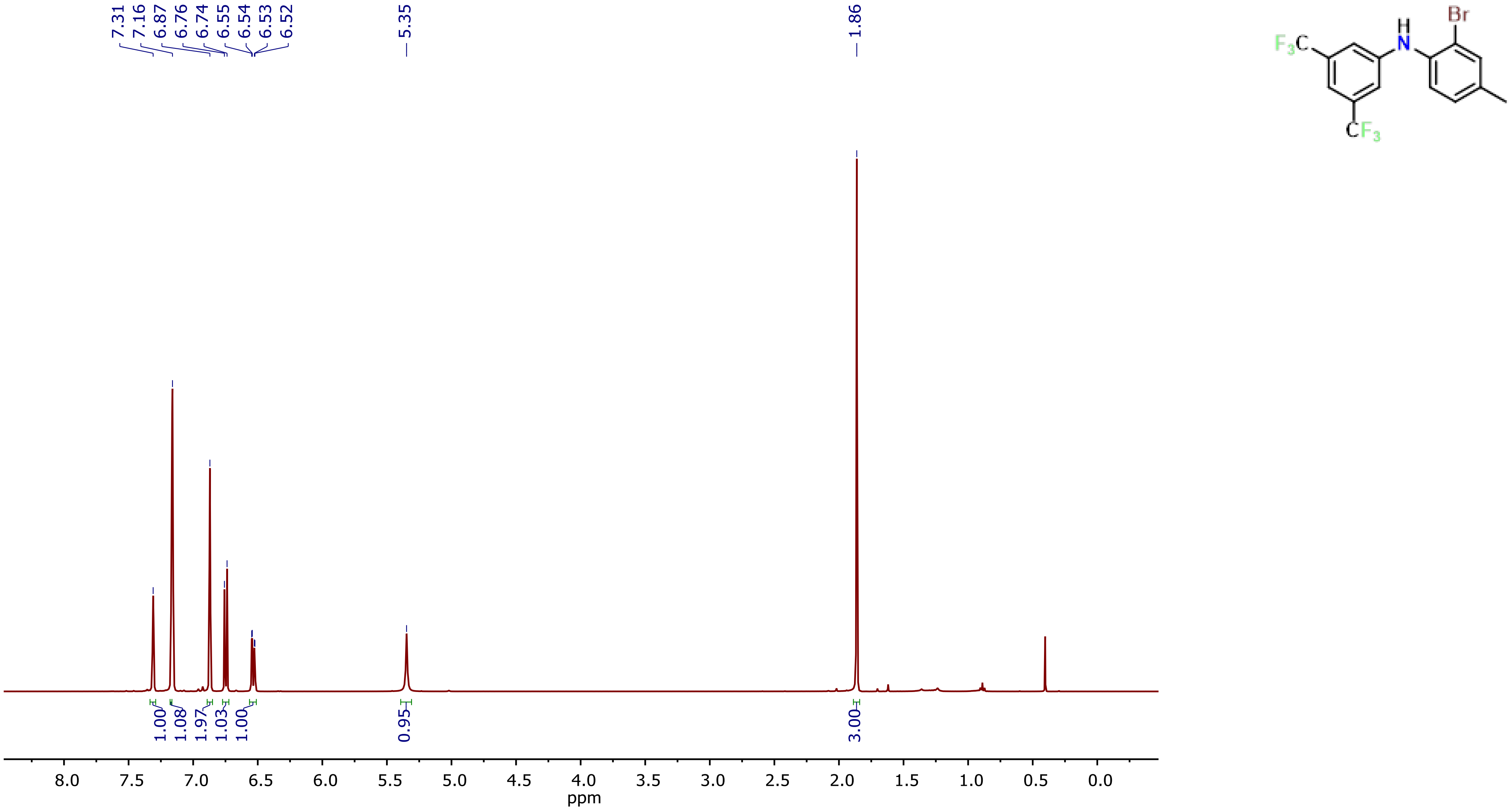

*Figure S 18: ^{1}H NMR spectrum of **2b** in C_6D_6 at 298 K.*

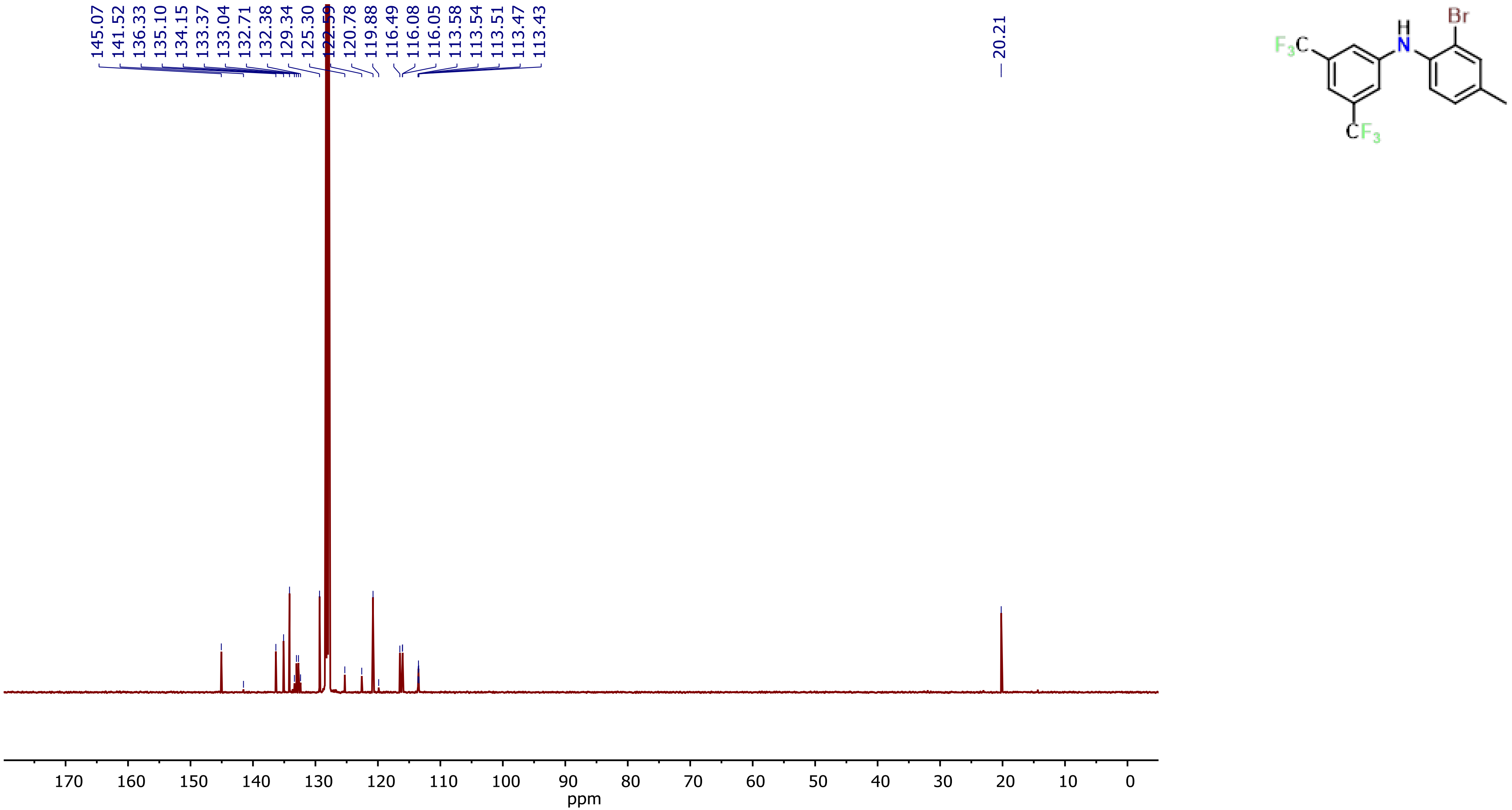

*Figure S 19: $^{13}C\{^{1}H\}$ NMR spectrum of **2b** in C_6D_6 at 298 K.*

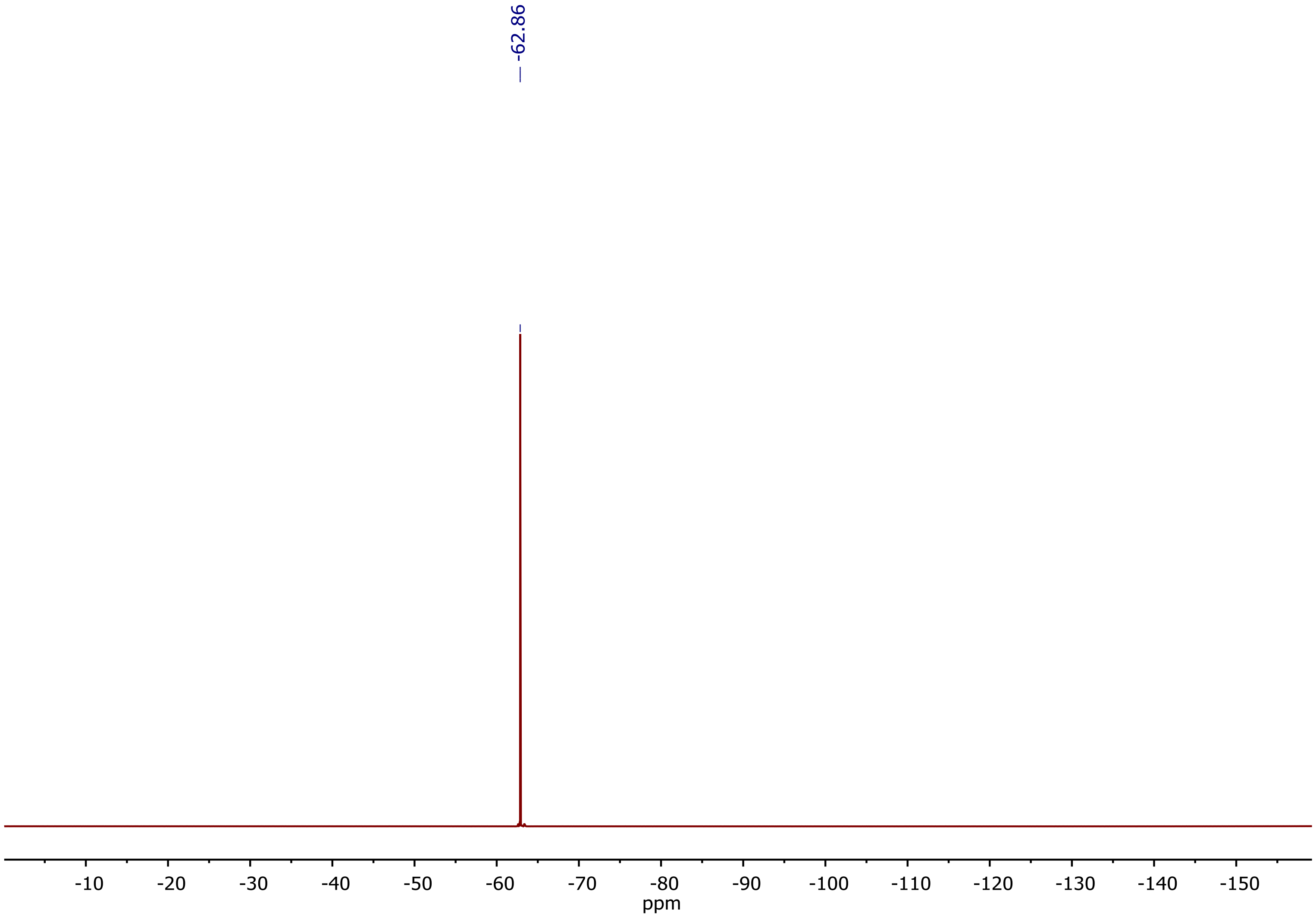

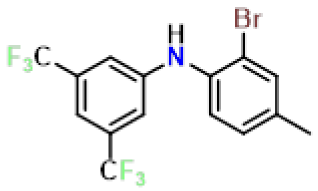

*Figure S 20: ^{19}F NMR spectrum of **2b** in C_6D_6 at 298 K.*

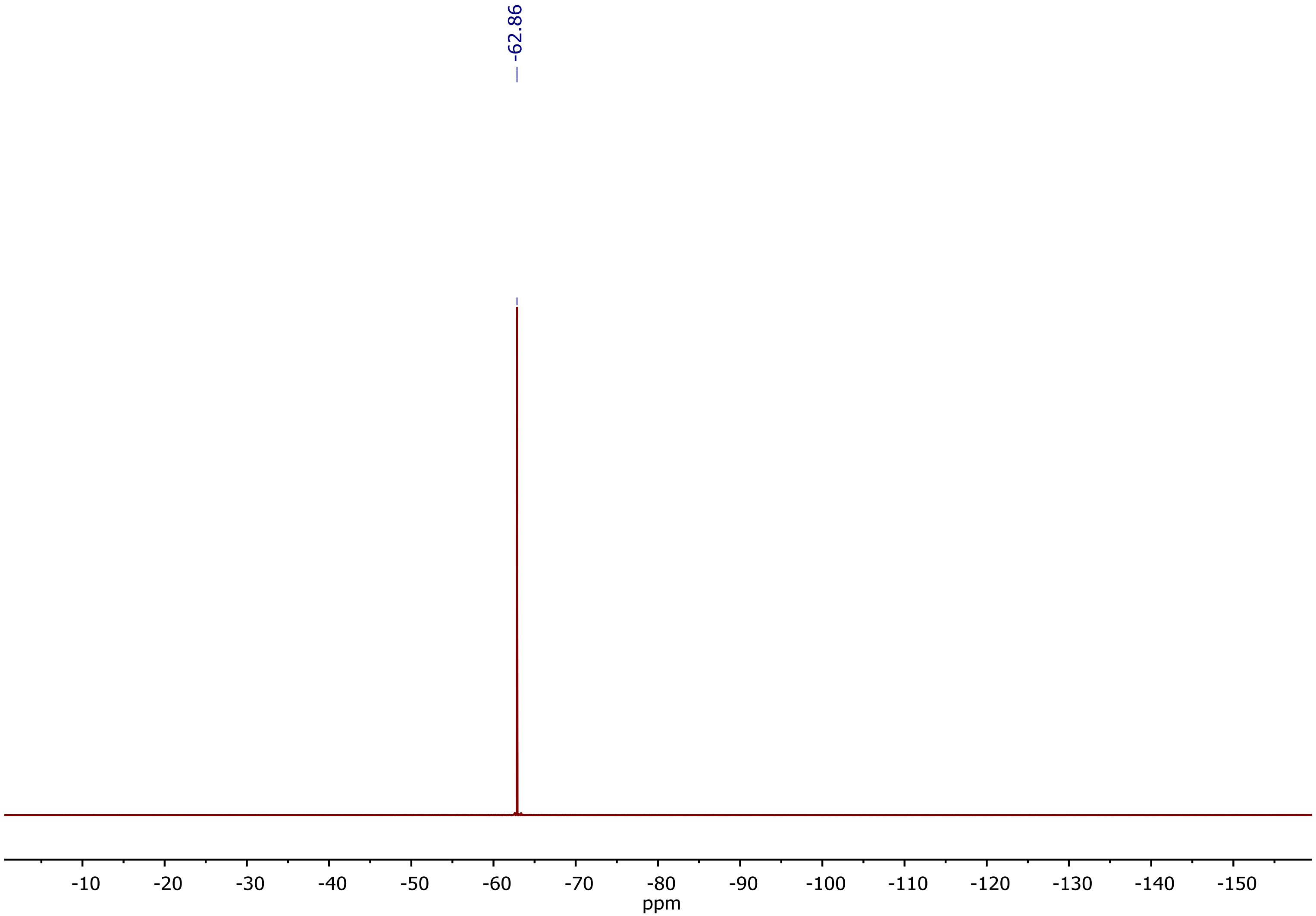

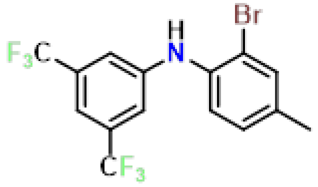

*Figure S 21: $^{19}F\{^{1}H\}$ NMR spectrum of **2b** in C_6D_6 at 298 K.*

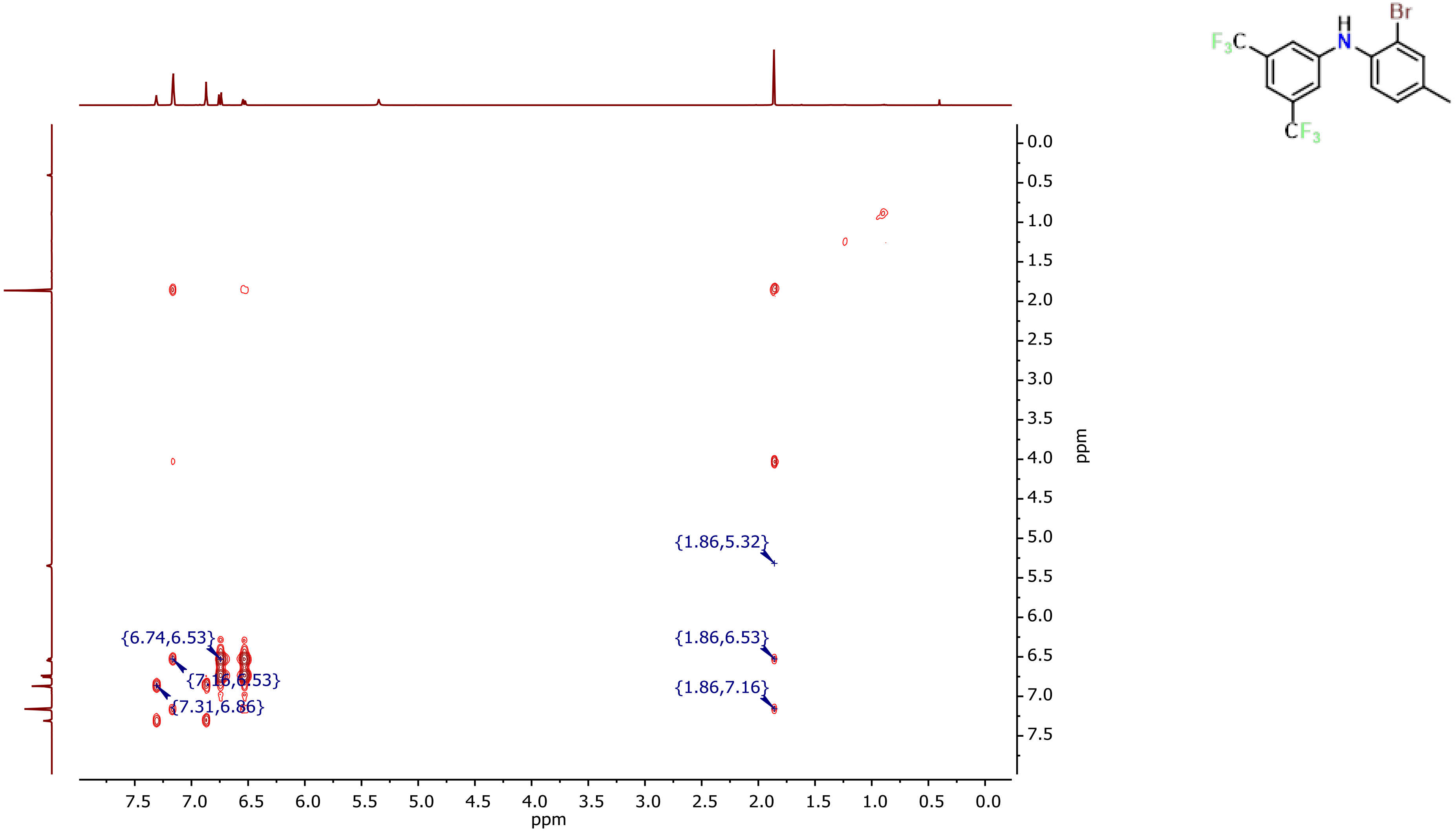

*Figure S 22: ^1H-^1H COSY NMR spectrum of **2b** in C_6D_6 at 298 K.*

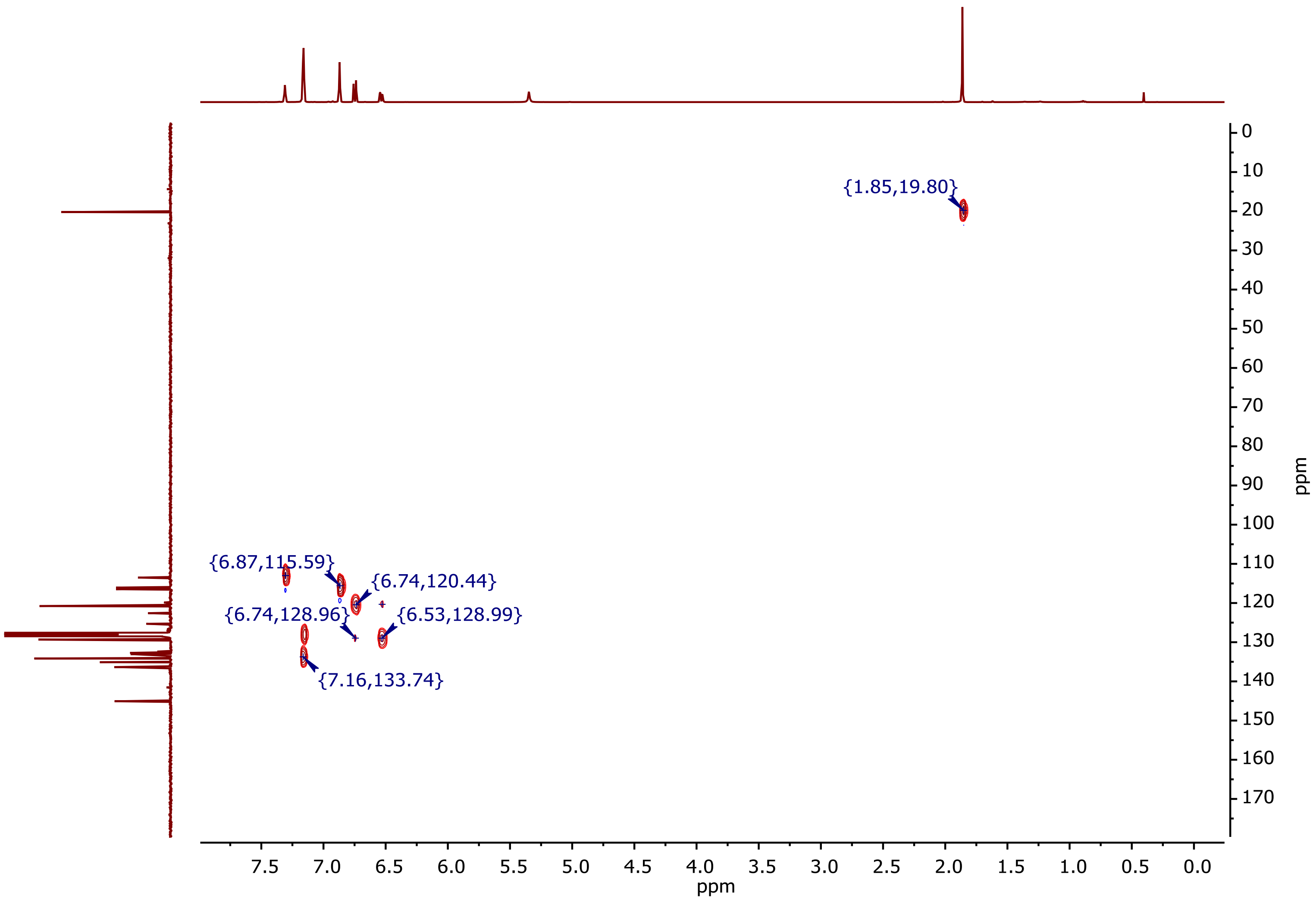

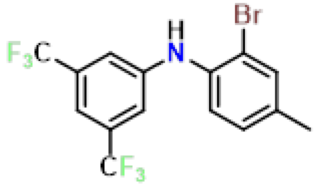

*Figure S 23: ^{1}H-^{13}C HSQC NMR spectrum of **2b** in C_6D_6 at 298 K.*

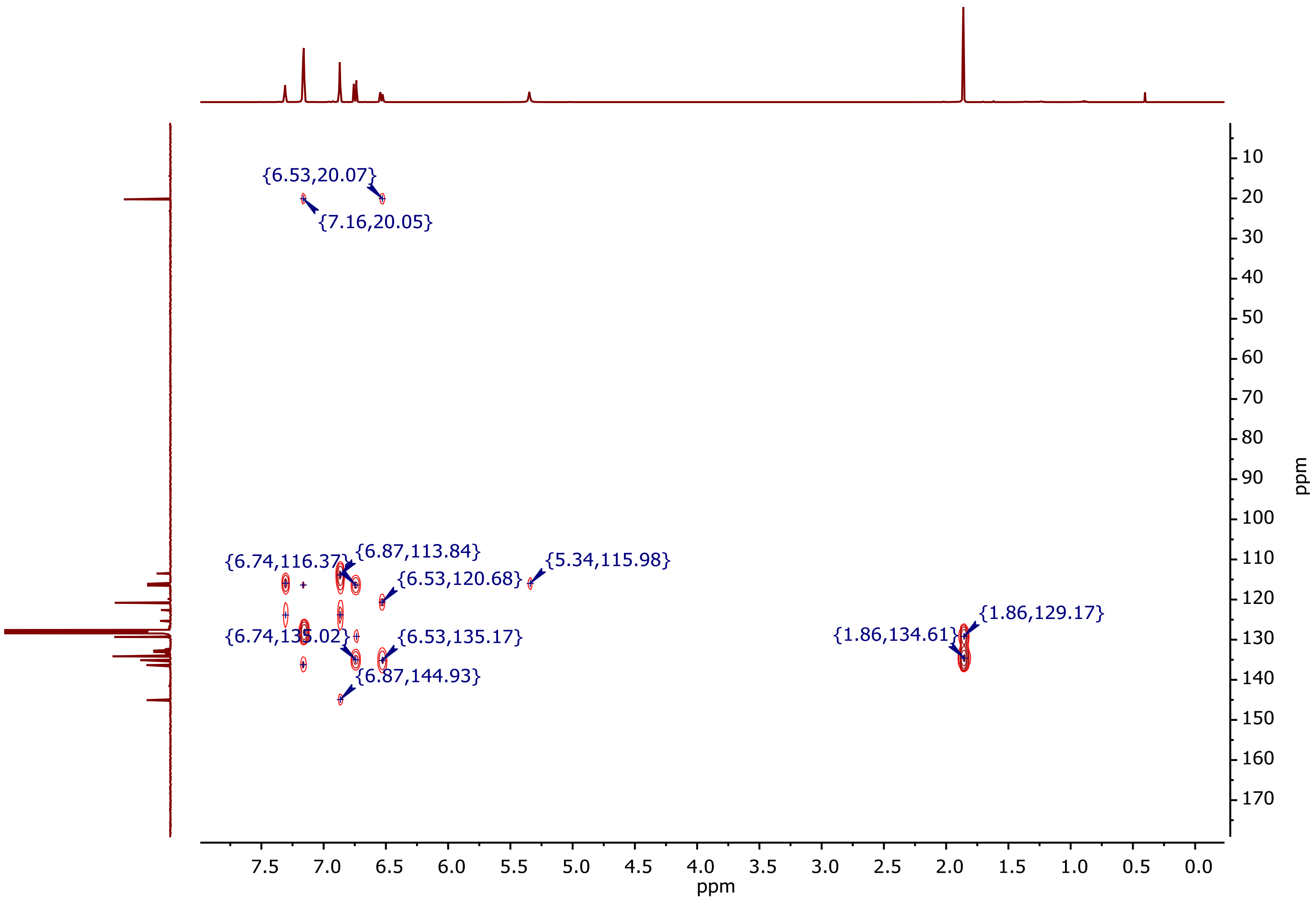

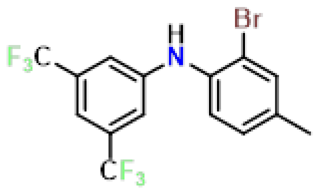

Figure S 24: ^{1}H-^{13}C HMBC NMR spectrum of **2b** in C_6D_6 at 298 K.

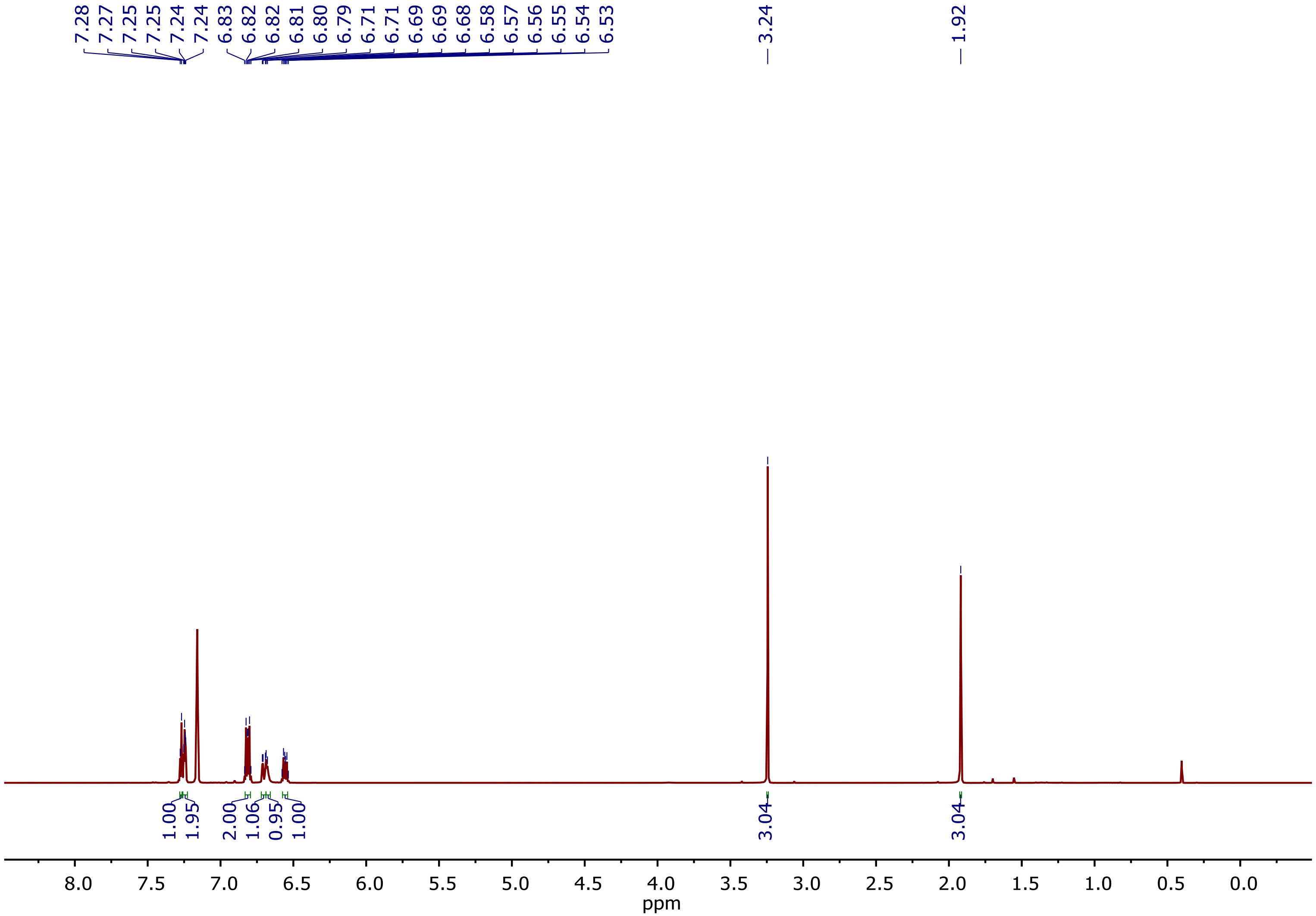

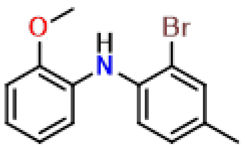

*Figure S 25: ^{1}H NMR spectrum of **2c** in C_6D_6 at 298 K.*

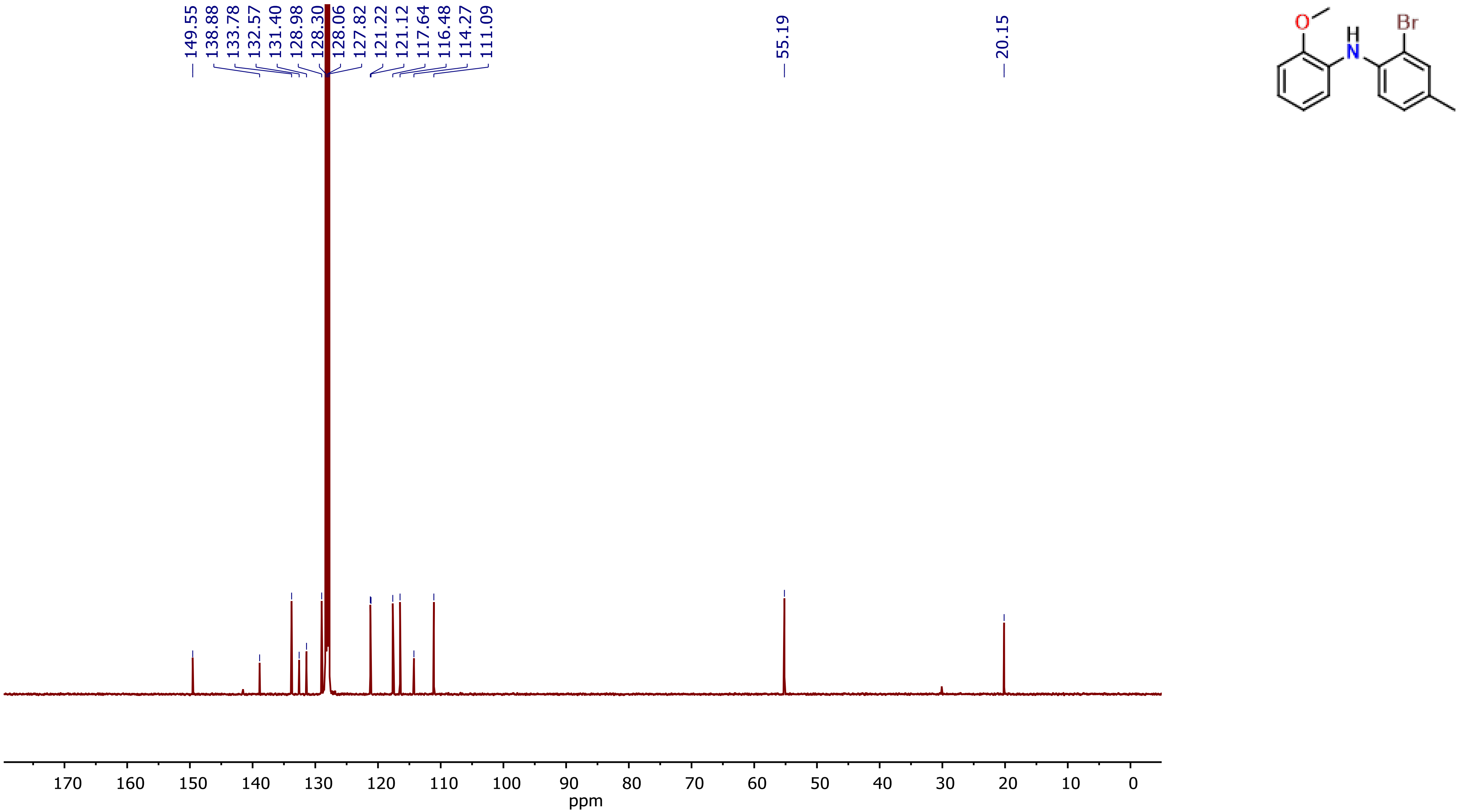

*Figure S 26: $^{13}C\{^{1}H\}$ NMR spectrum of **2c** in C_6D_6 at 298 K.*

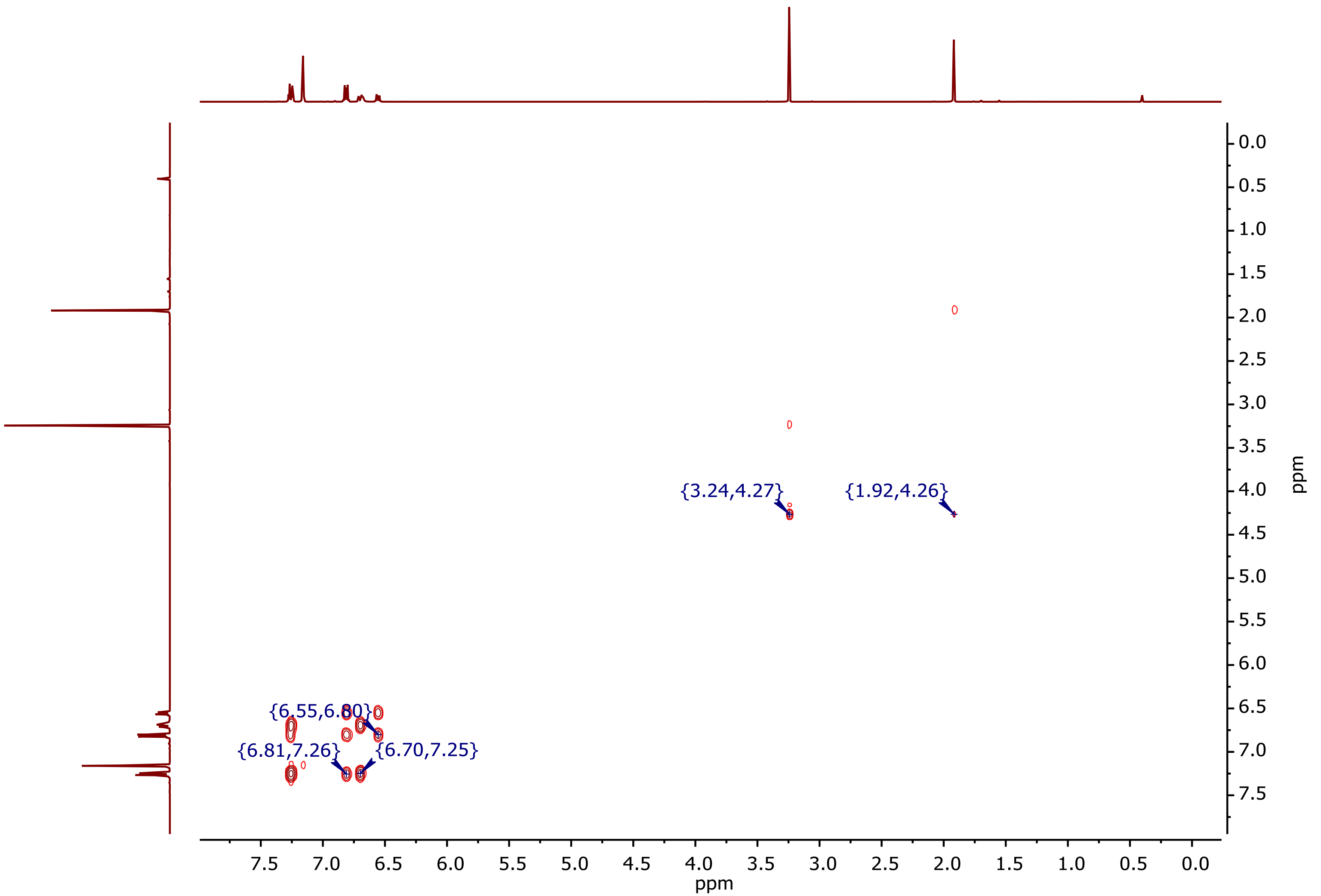

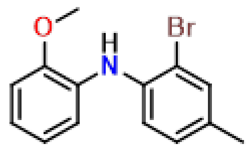

*Figure S 27: ^{1}H-^{1}H COSY NMR spectrum of **2c** in C_6D_6 at 298 K.*

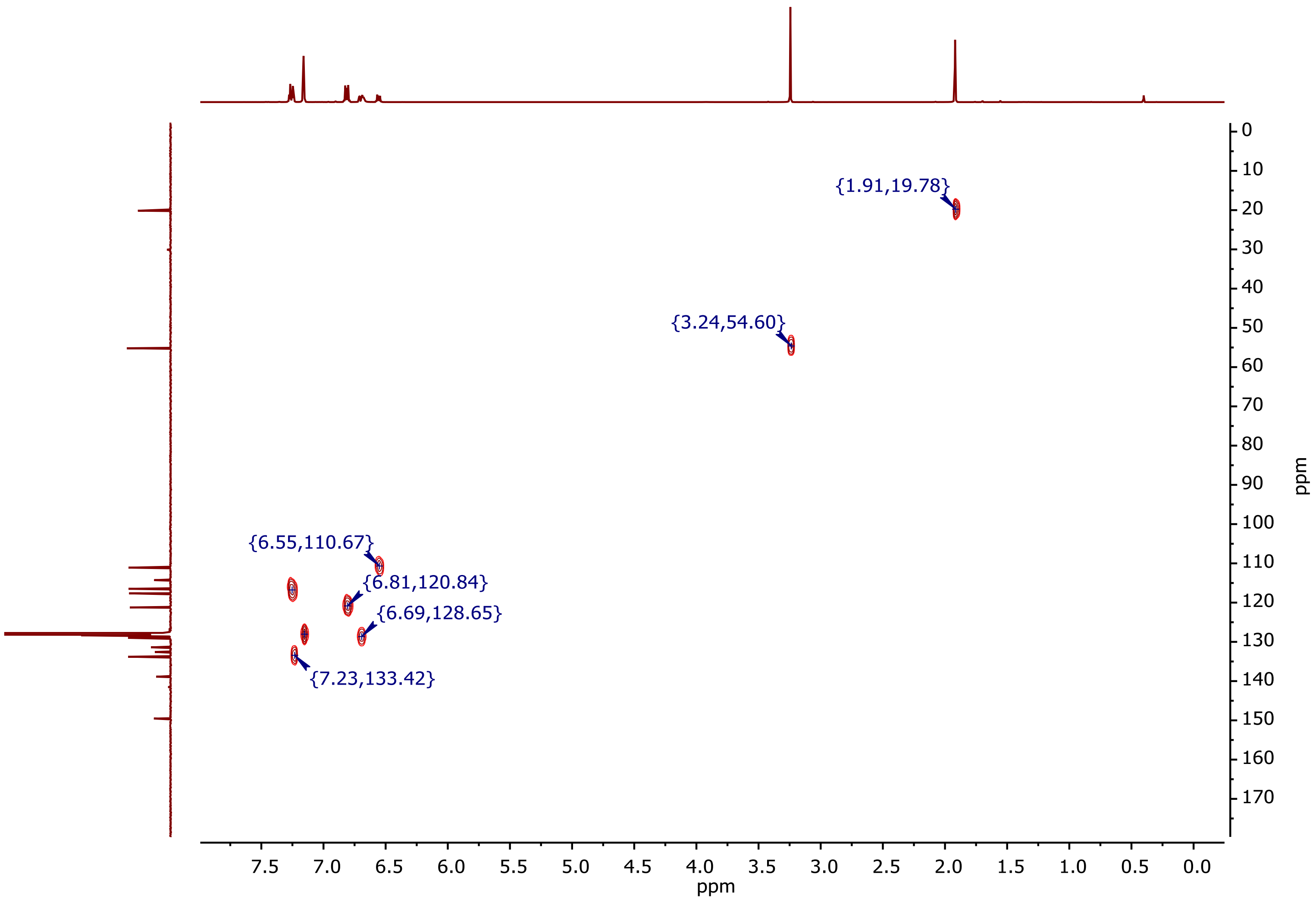

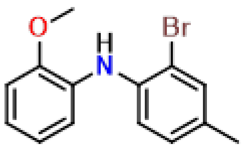

Figure S 28: ^{1}H-^{13}C HSQC NMR spectrum of ***2c*** *in C_6D_6 at 298 K.*

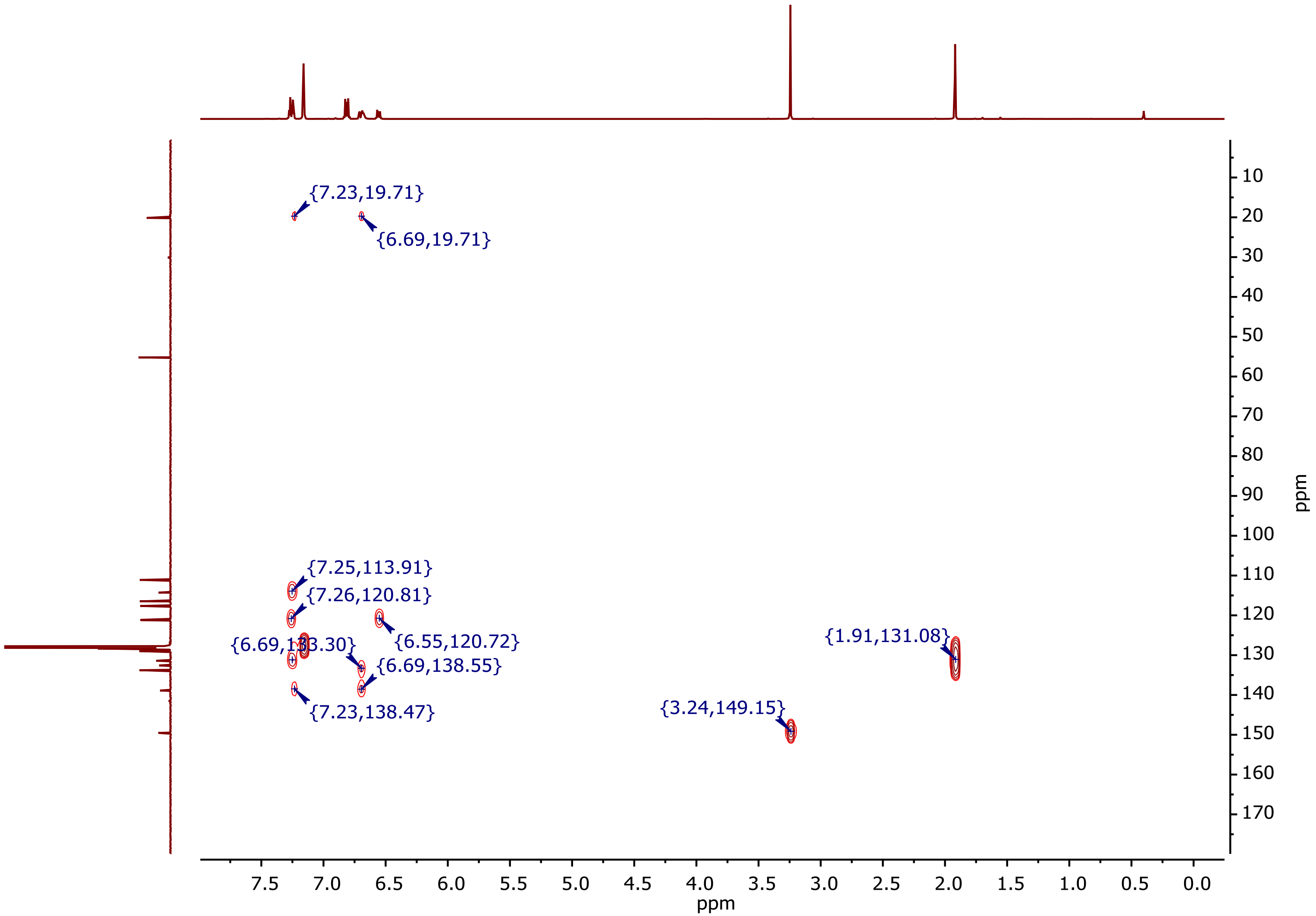

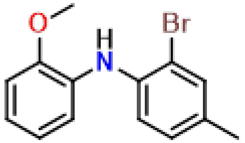

Figure S 29: ^{1}H-^{13}C HMBC NMR spectrum of **2c** in C_6D_6 at 298 K.

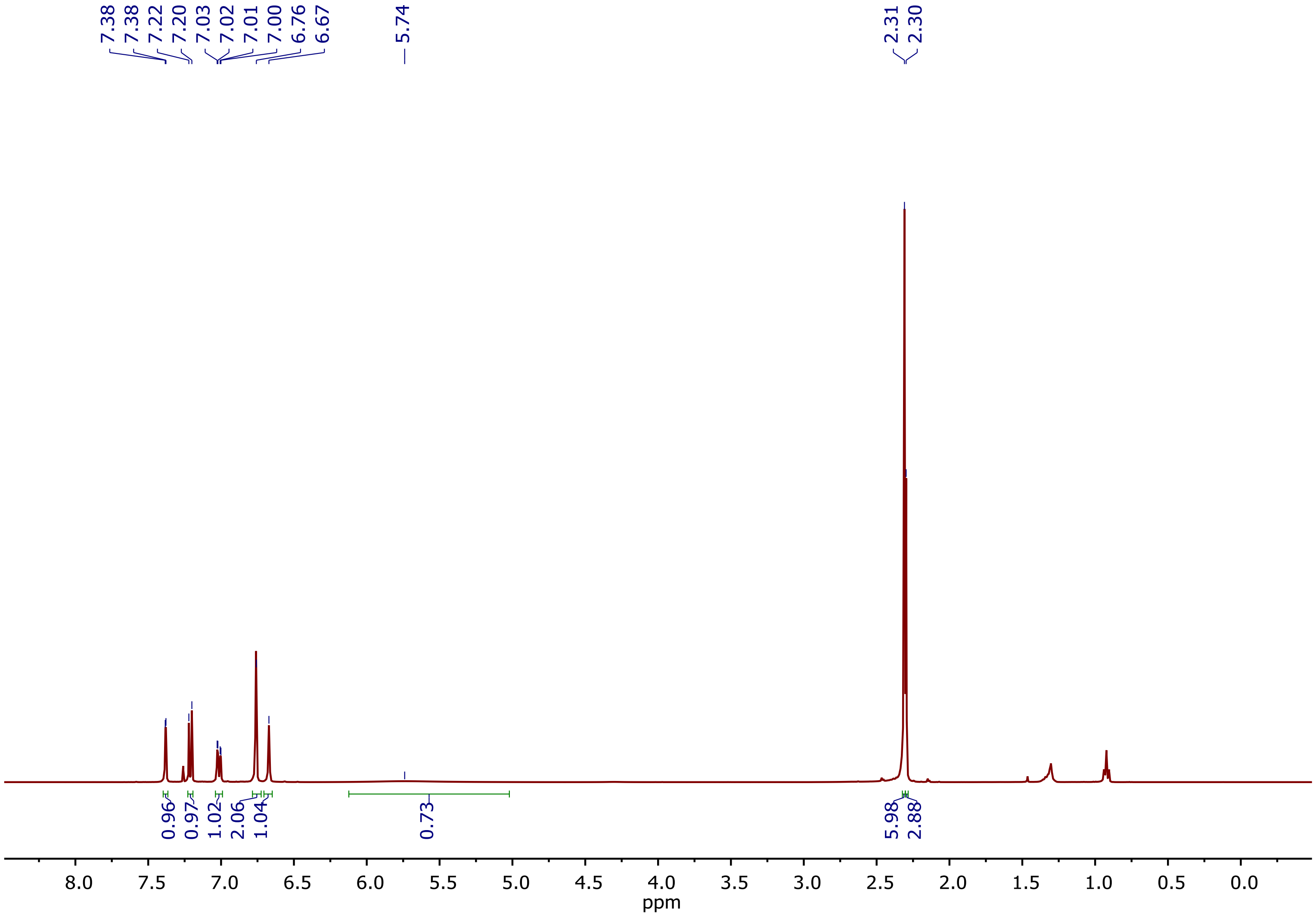

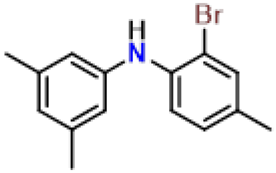

*Figure S 30: ^{1}H NMR spectrum of **2d** in $CDCl_3$ at 298 K.*

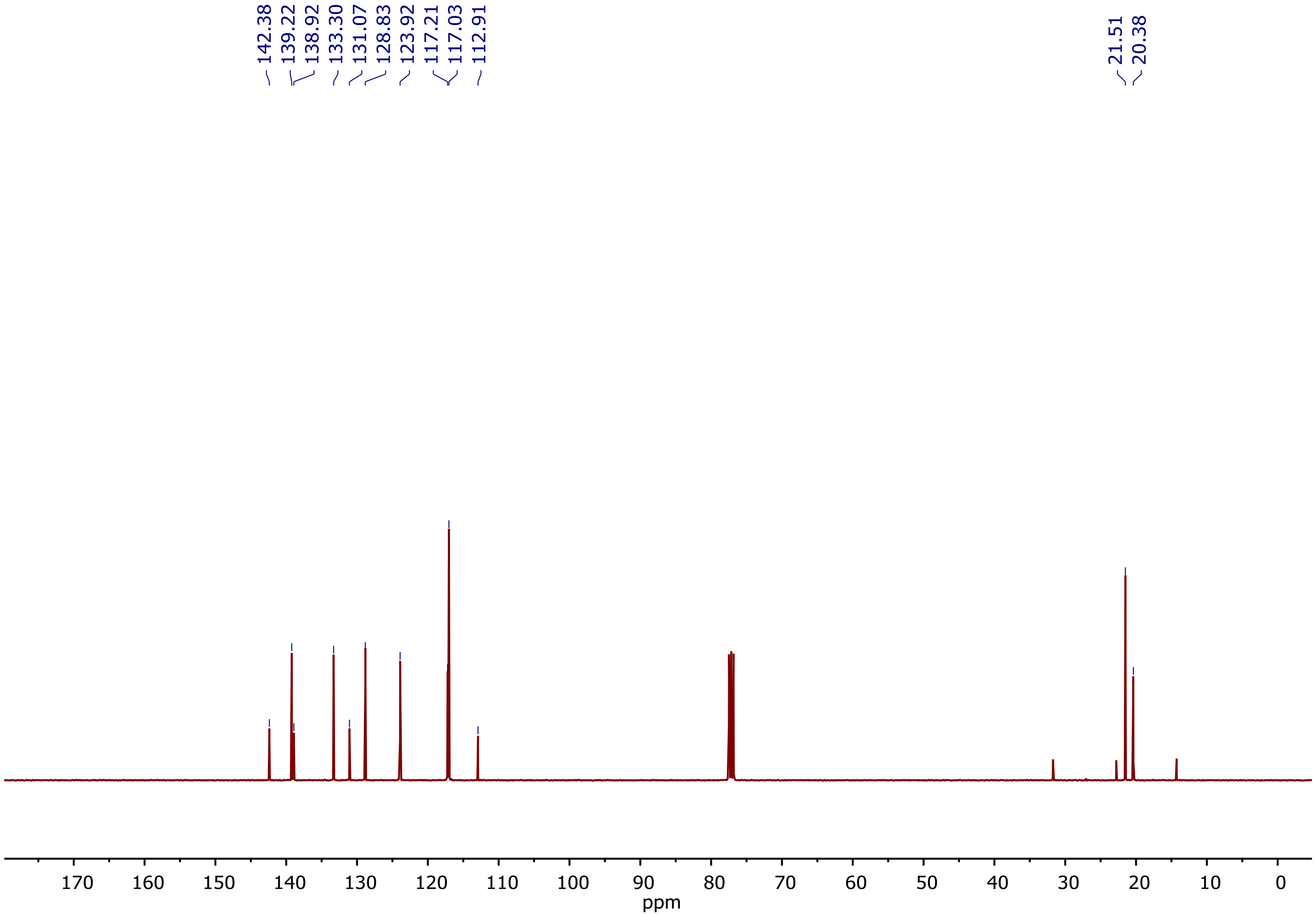

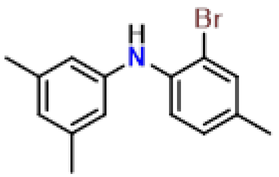

Figure S 31: $^{13}C\{^1H\}$ NMR spectrum of **2d** in $CDCl_3$ at 298 K.

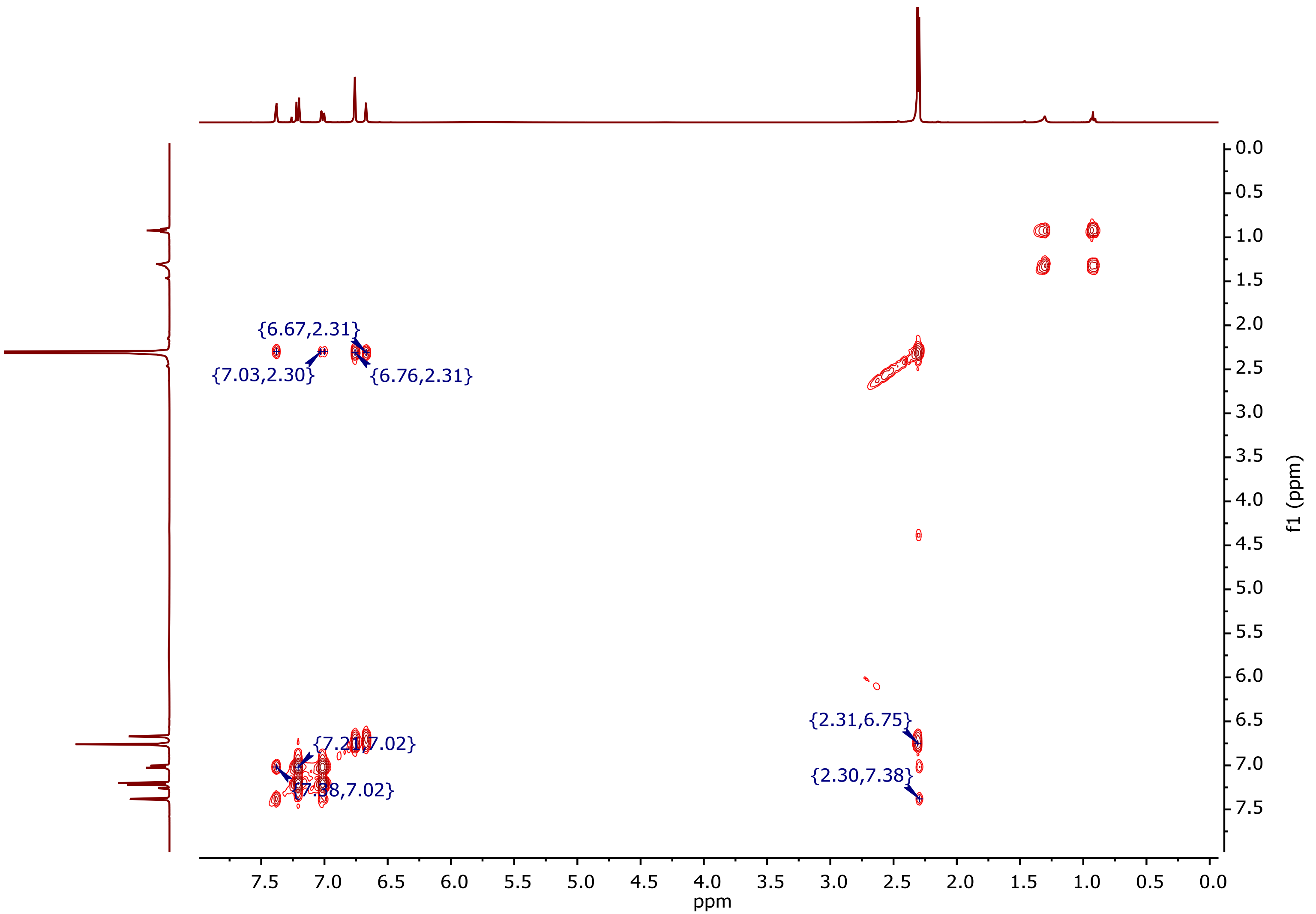

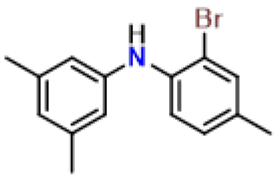

*Figure S 32: ^{1}H-^{1}H COSY NMR spectrum of **2d** in $CDCl_3$ at 298 K.*

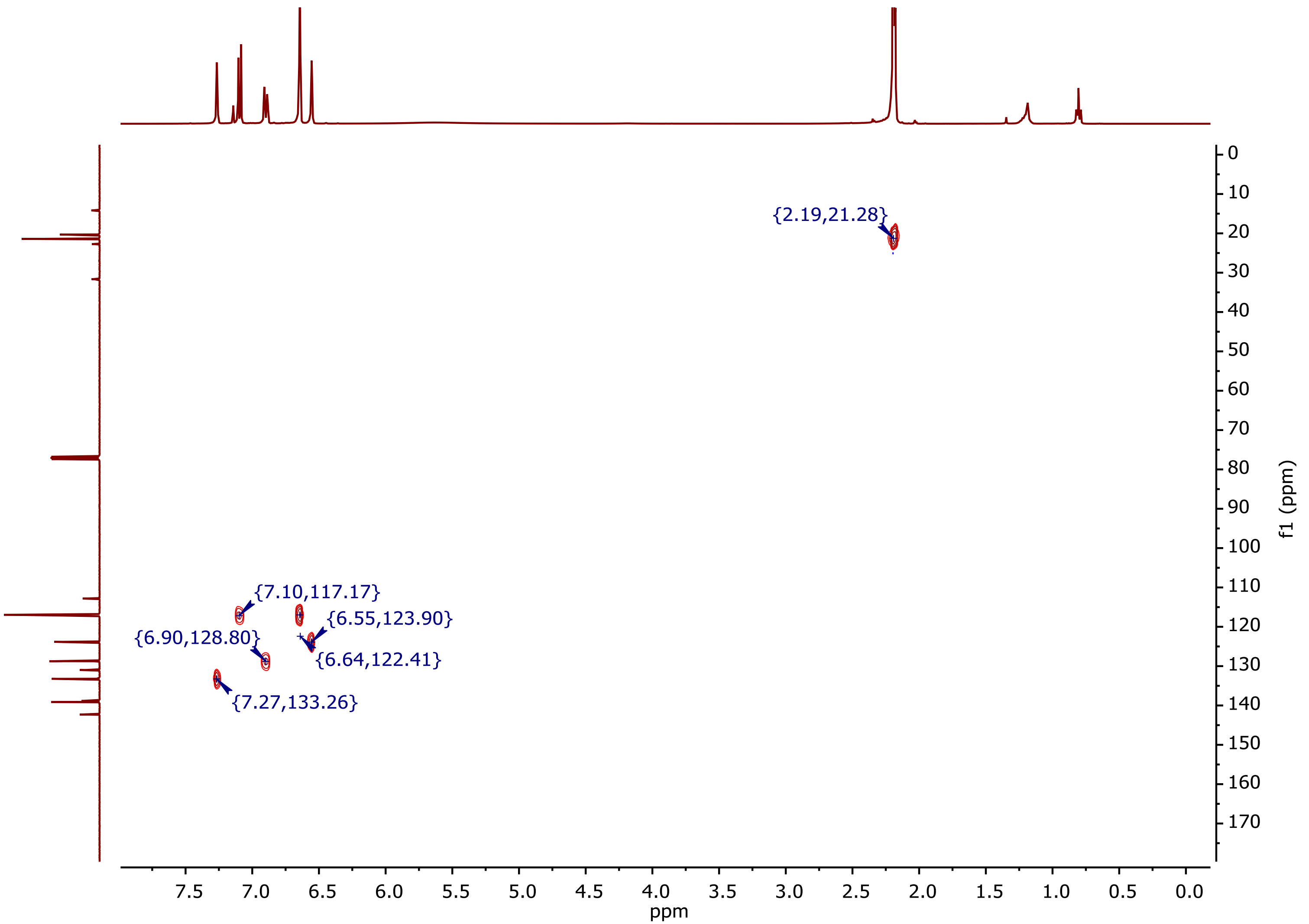

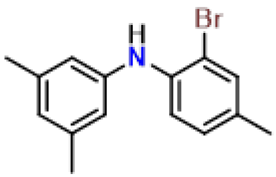

Figure S 33: ^{1}H-^{13}C HSQC NMR spectrum of **2d** in $CDCl_3$ at 298 K.

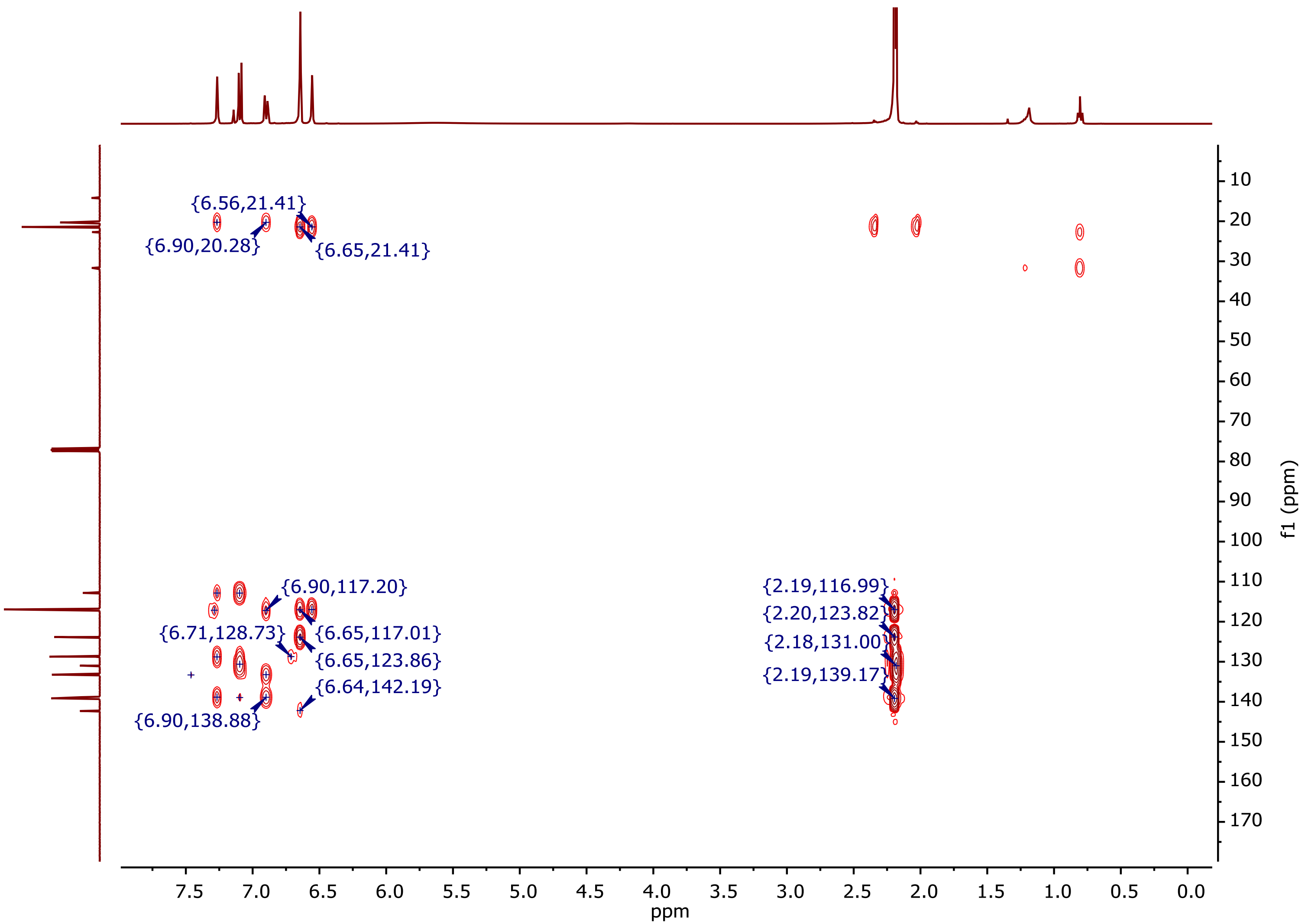

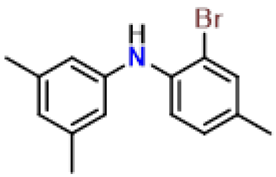

*Figure S 34: ^{1}H-^{13}C HMBC NMR spectrum of **2d** in $CDCl_3$ at 298 K.*

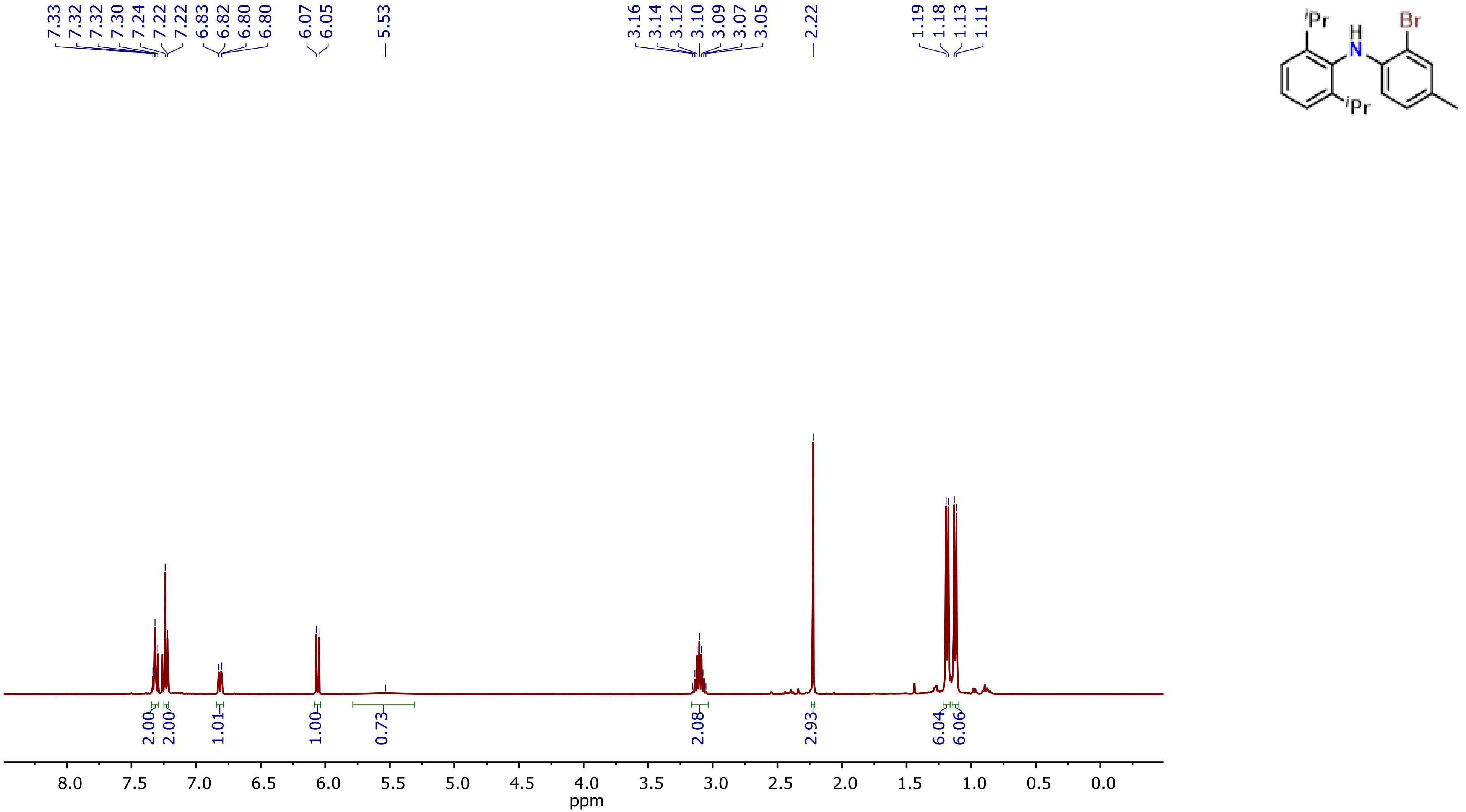

*Figure S 35: ^{1}H NMR spectrum of **2e** in $CDCl_3$ at 298 K.*

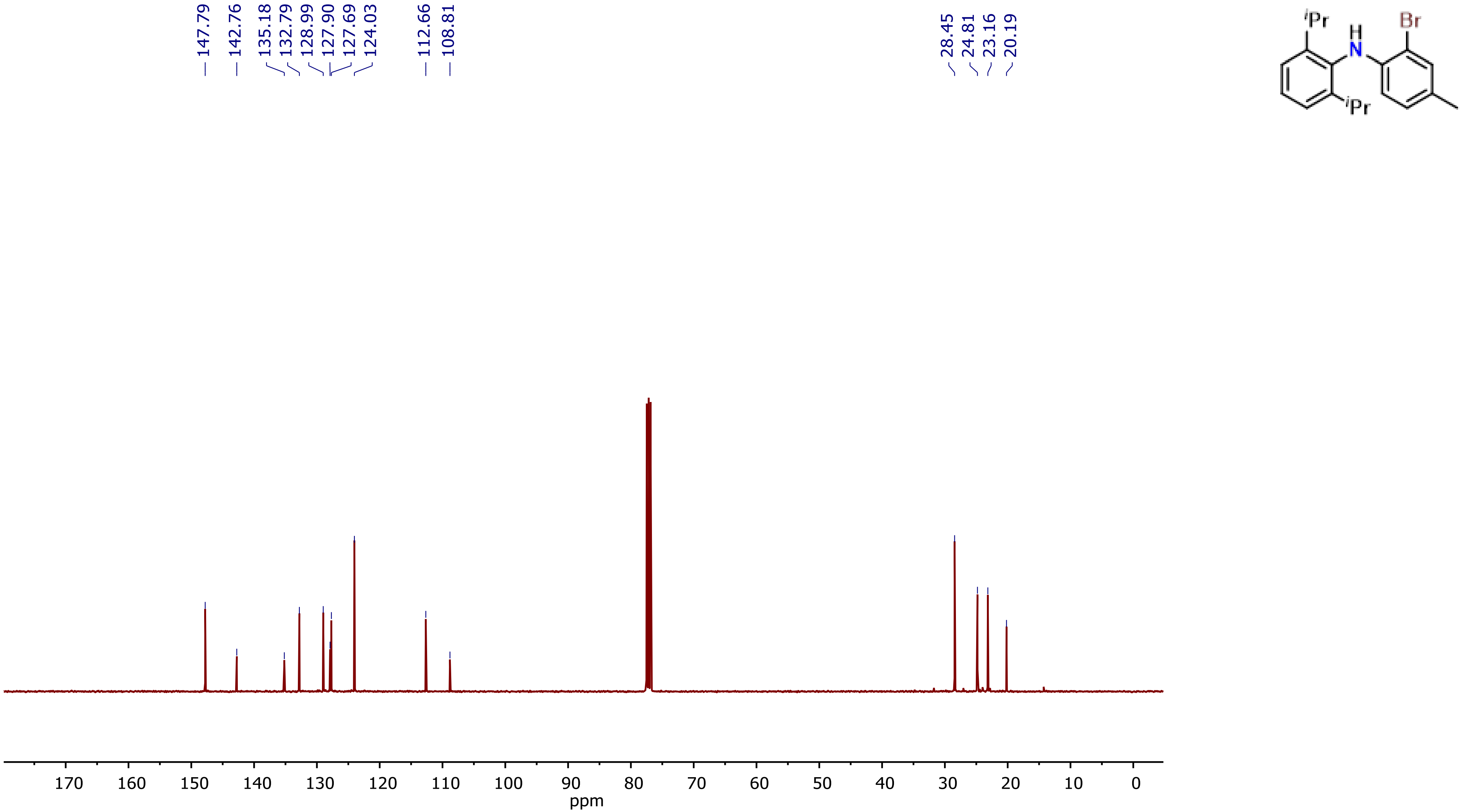

*Figure S 36: $^{13}C\{^{1}H\}$ NMR spectrum of **2e** in $CDCl_3$ at 298 K.*

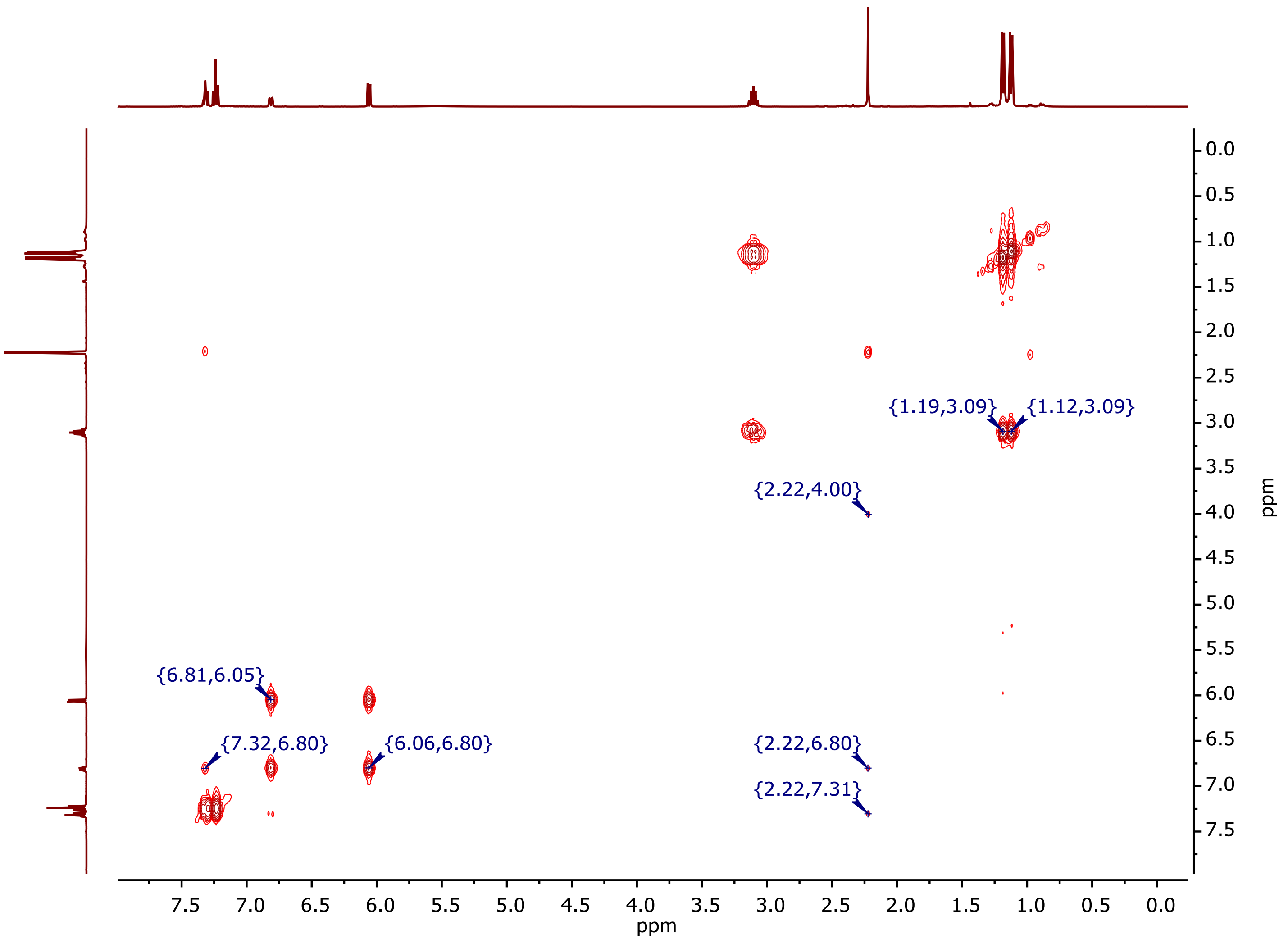

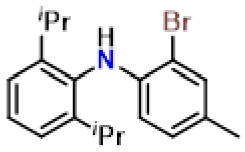

*Figure S 37: ^{1}H-^{1}H COSY NMR spectrum of **2e** in $CDCl_3$ at 298 K.*

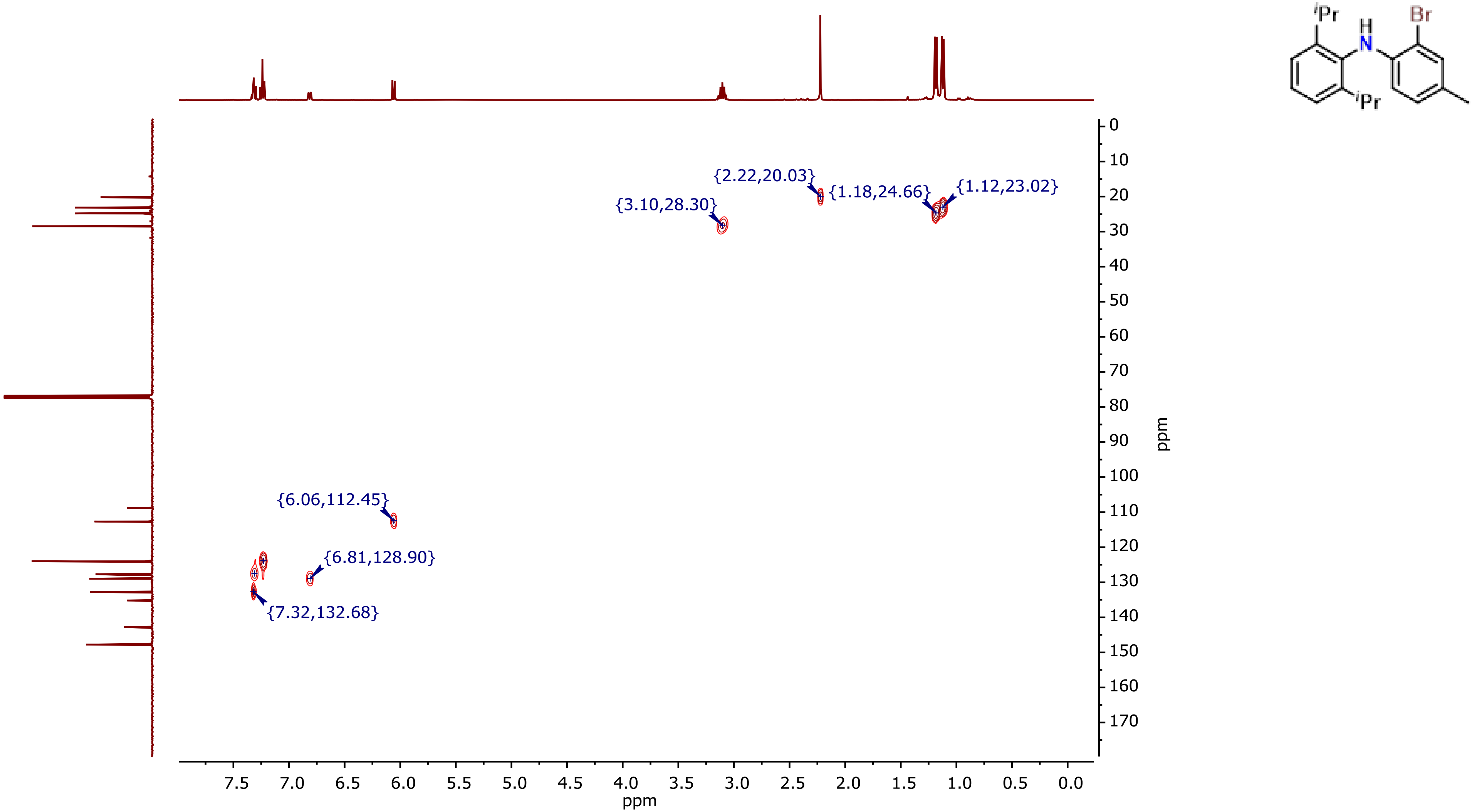

*Figure S 38: ^{1}H-^{13}C HSQC NMR spectrum of **2e** in $CDCl_3$ at 298 K.*

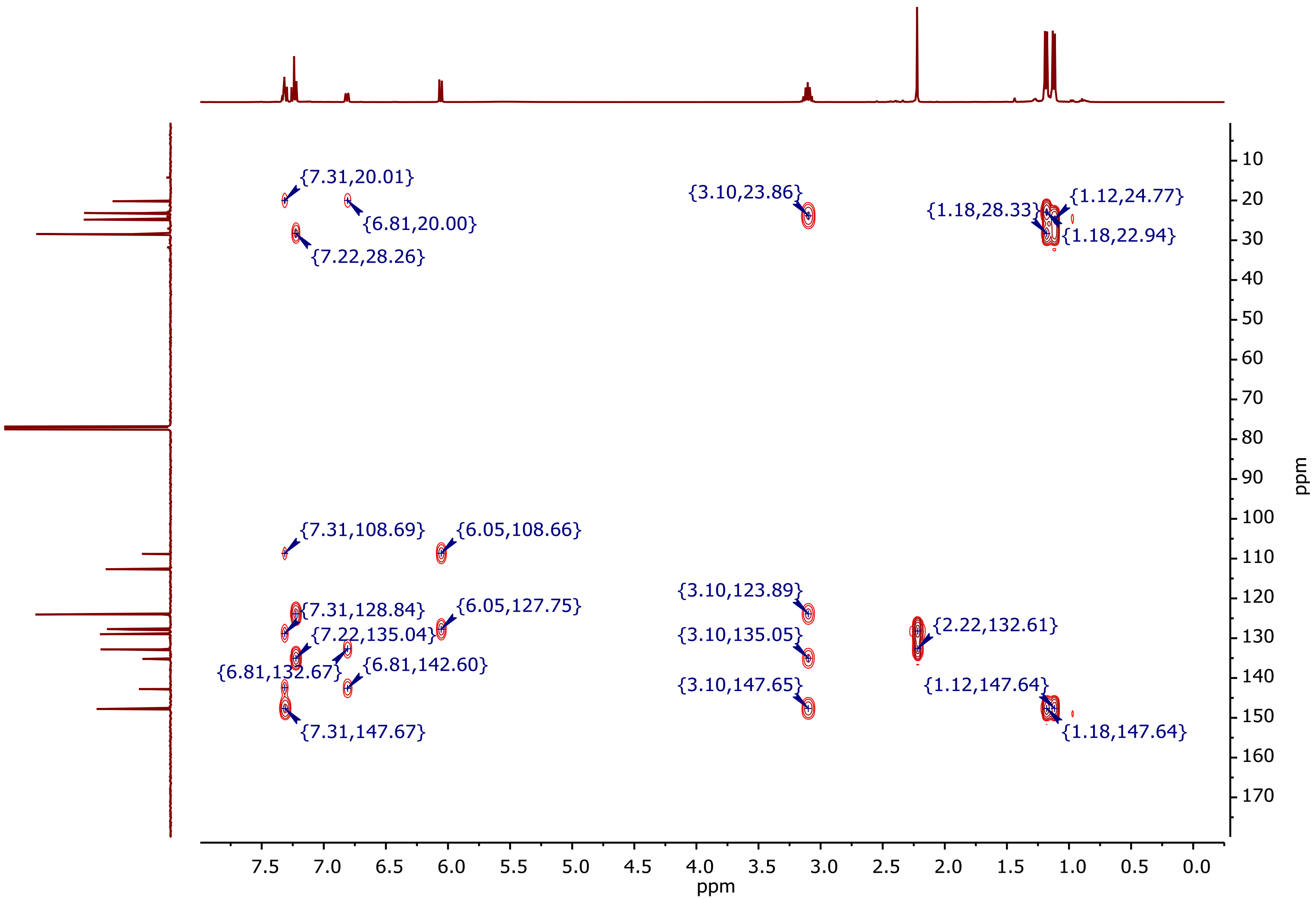

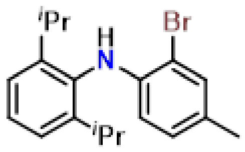

*Figure S 39: ^{1}H-^{13}C HMBC NMR spectrum of **2e** in $CDCl_3$ at 298 K.*

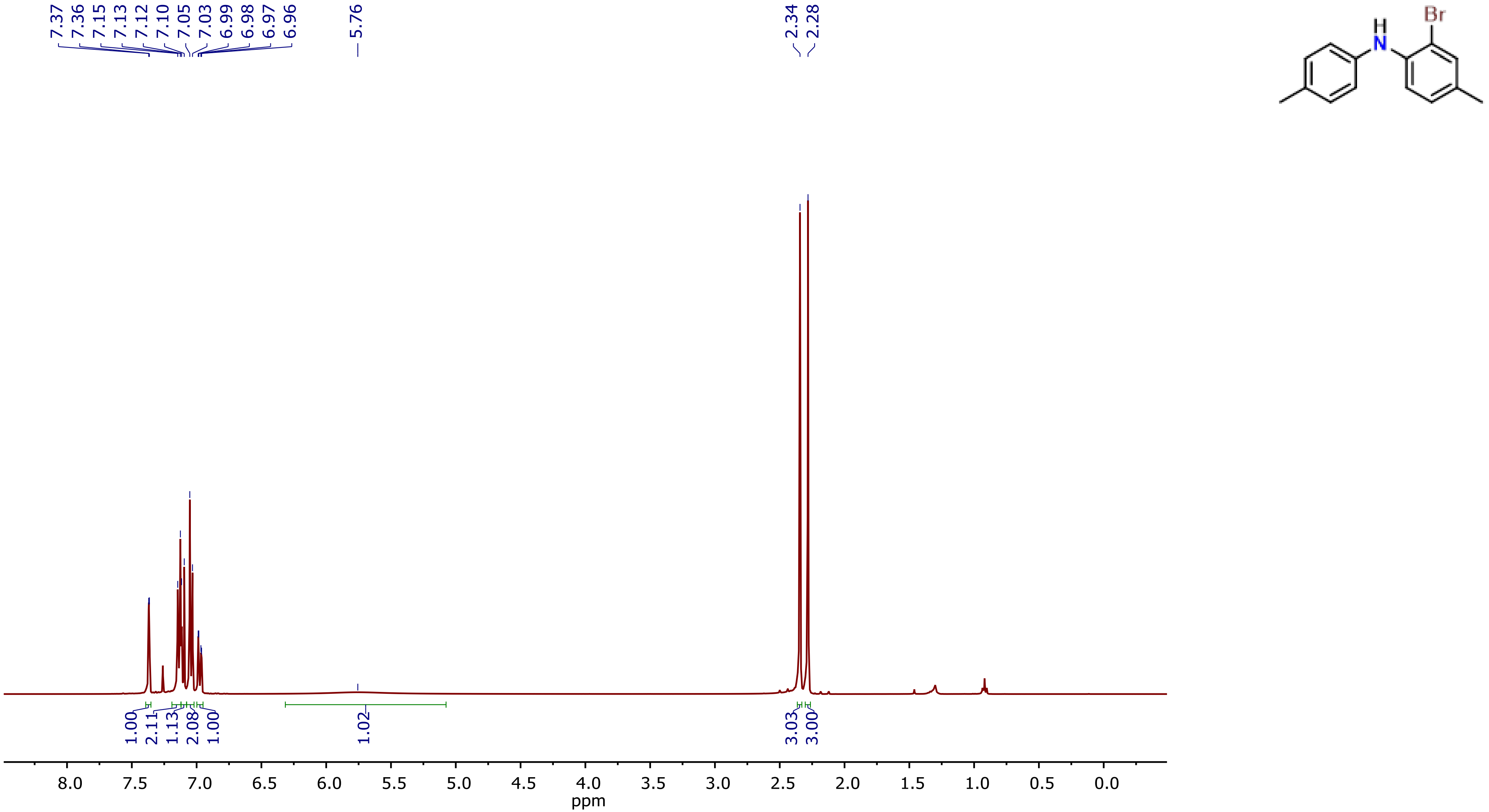

*Figure S 40: ^{1}H NMR spectrum of **2f** in $CDCl_3$ at 298 K.*

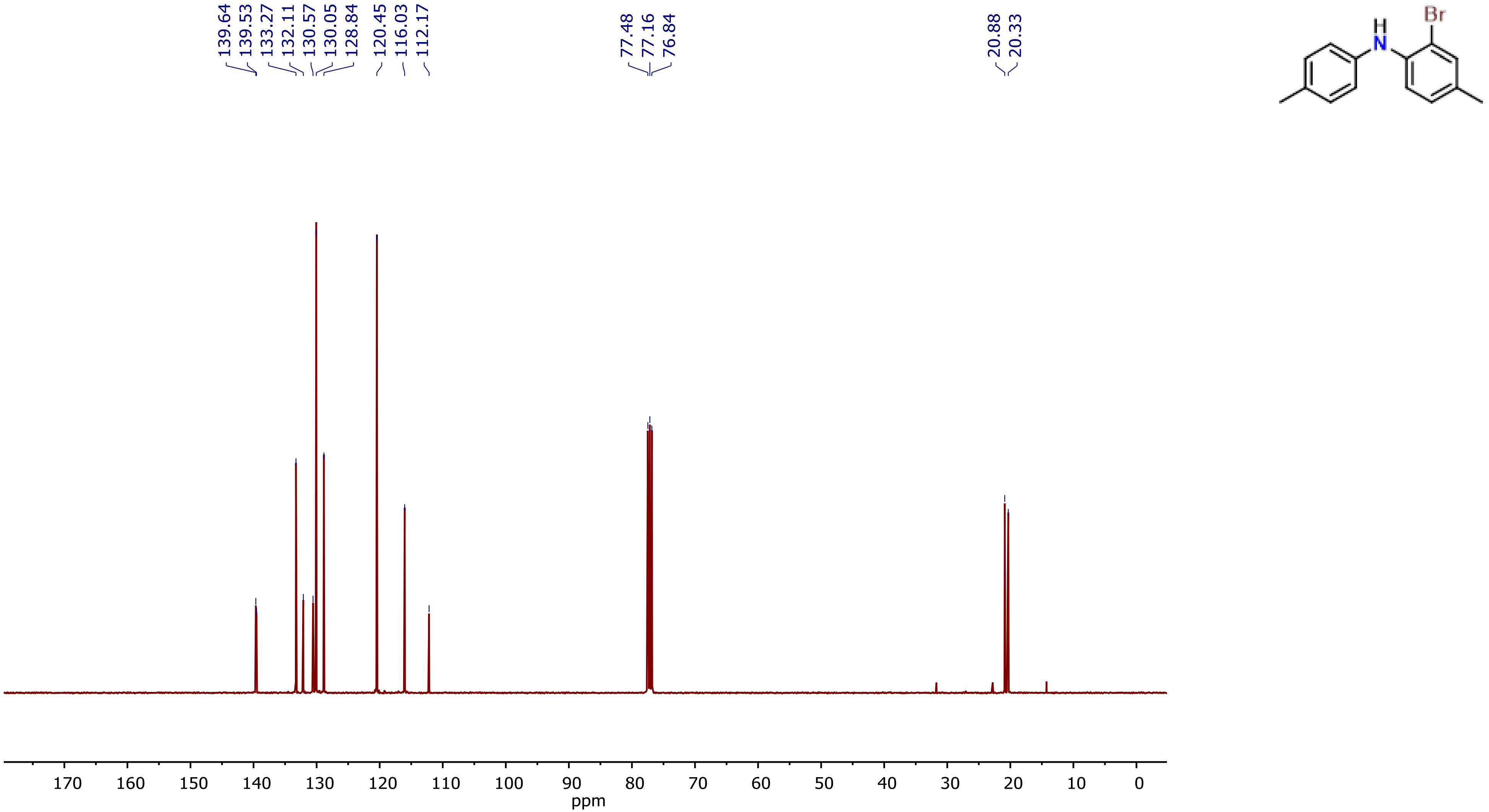

*Figure S 41: $^{13}C\{^{1}H\}$ NMR spectrum of **2f** in $CDCl_3$ at 298 K.*

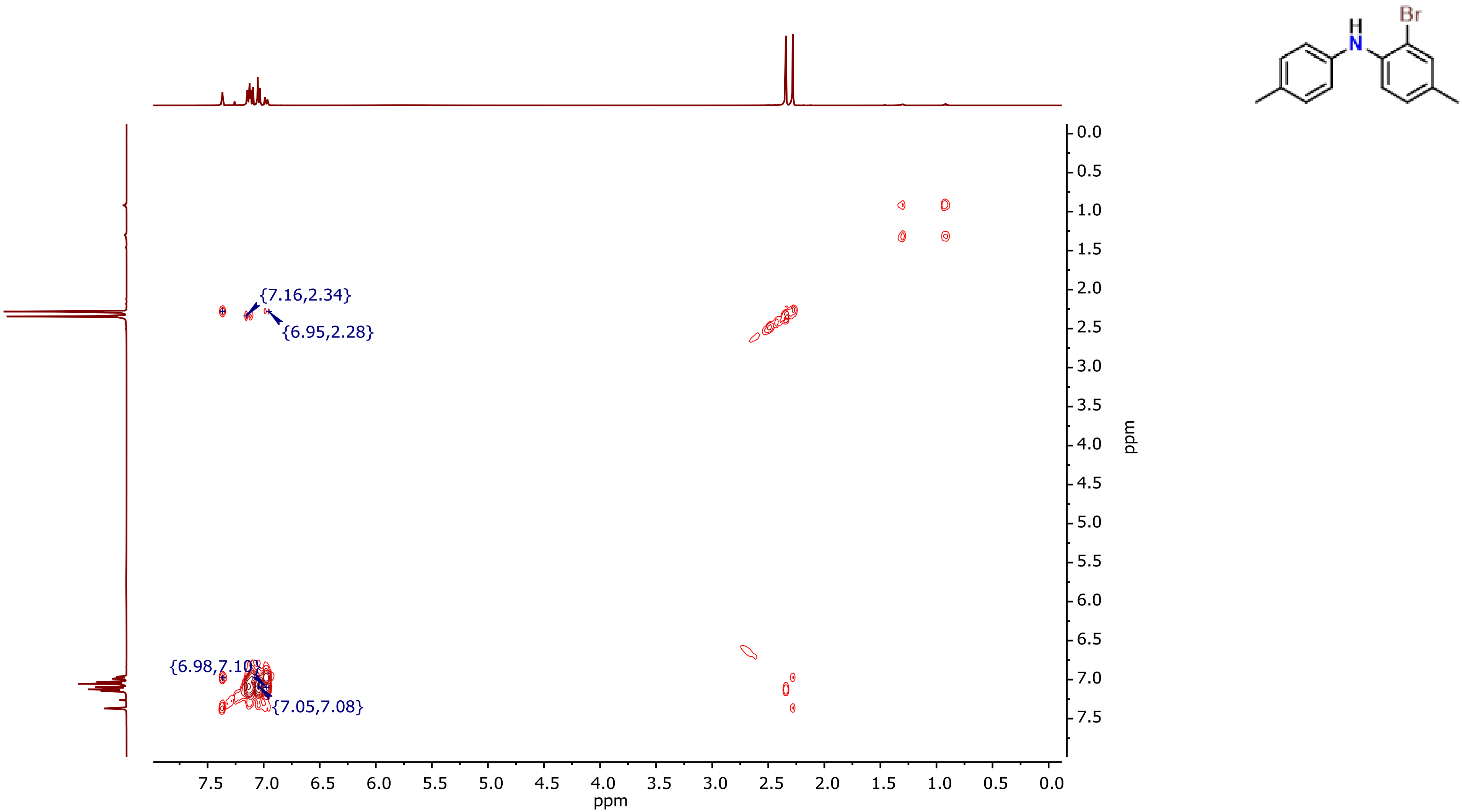

*Figure S 42: ^{1}H-^{1}H COSY NMR spectrum of **2f** in $CDCl_3$ at 298 K.*

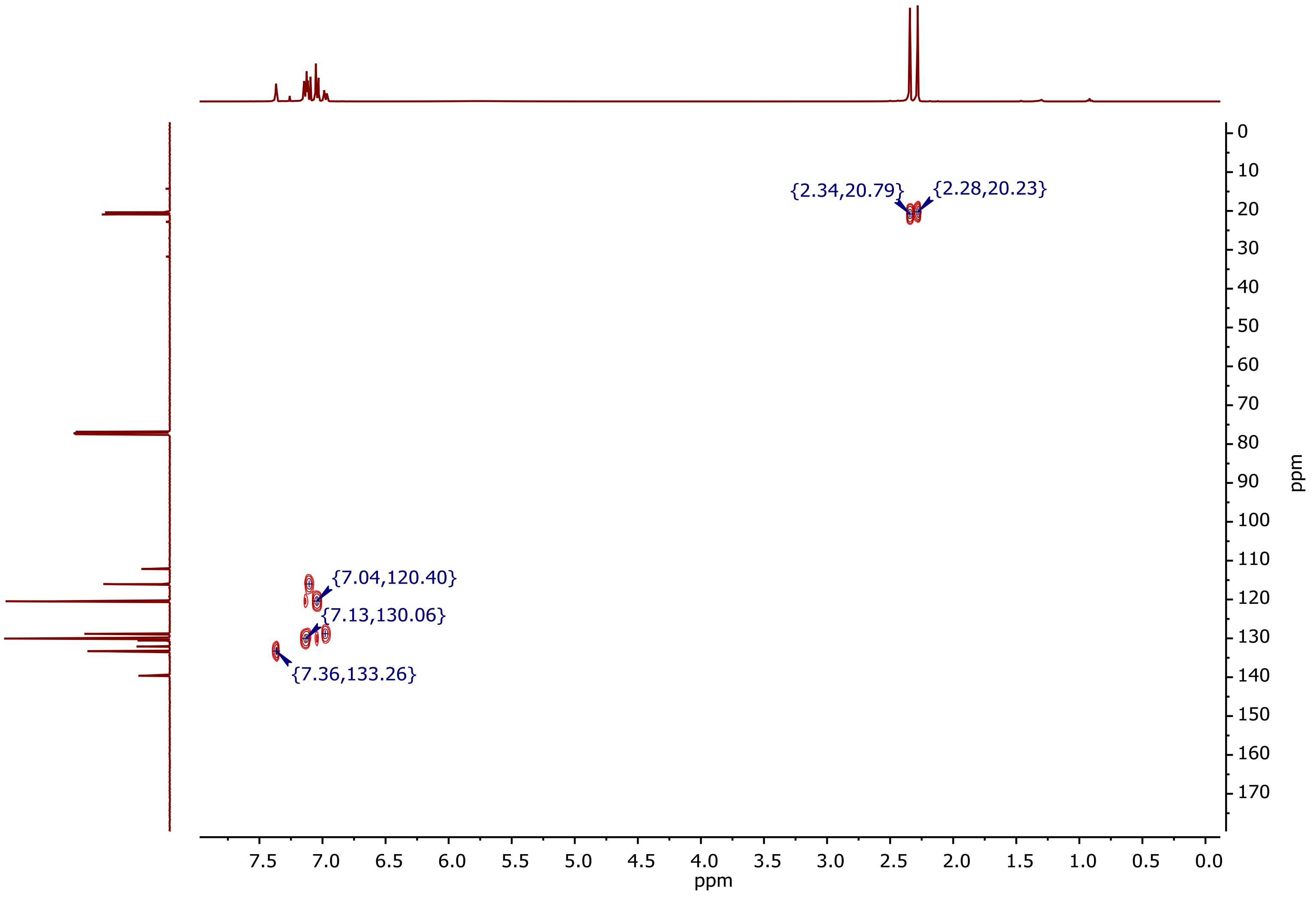

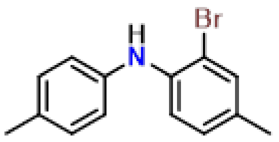

*Figure S 43: ^{1}H-^{13}C HSQC NMR spectrum of **2f** in $CDCl_3$ at 298 K.*

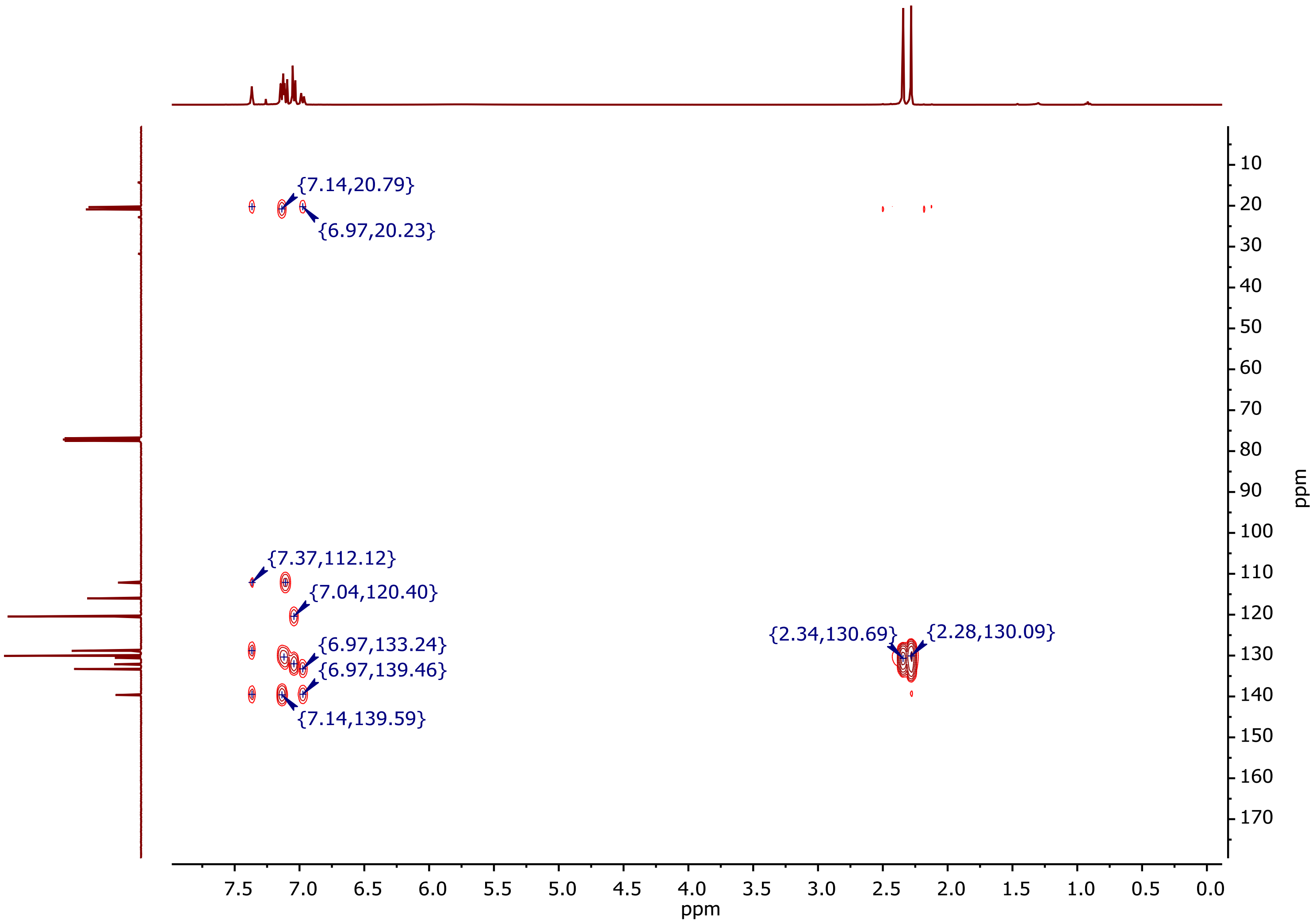

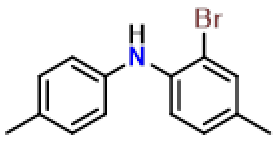

Figure S 44: ^{1}H-^{13}C HMBC NMR spectrum of **2f** in $CDCl_3$ at 298 K.

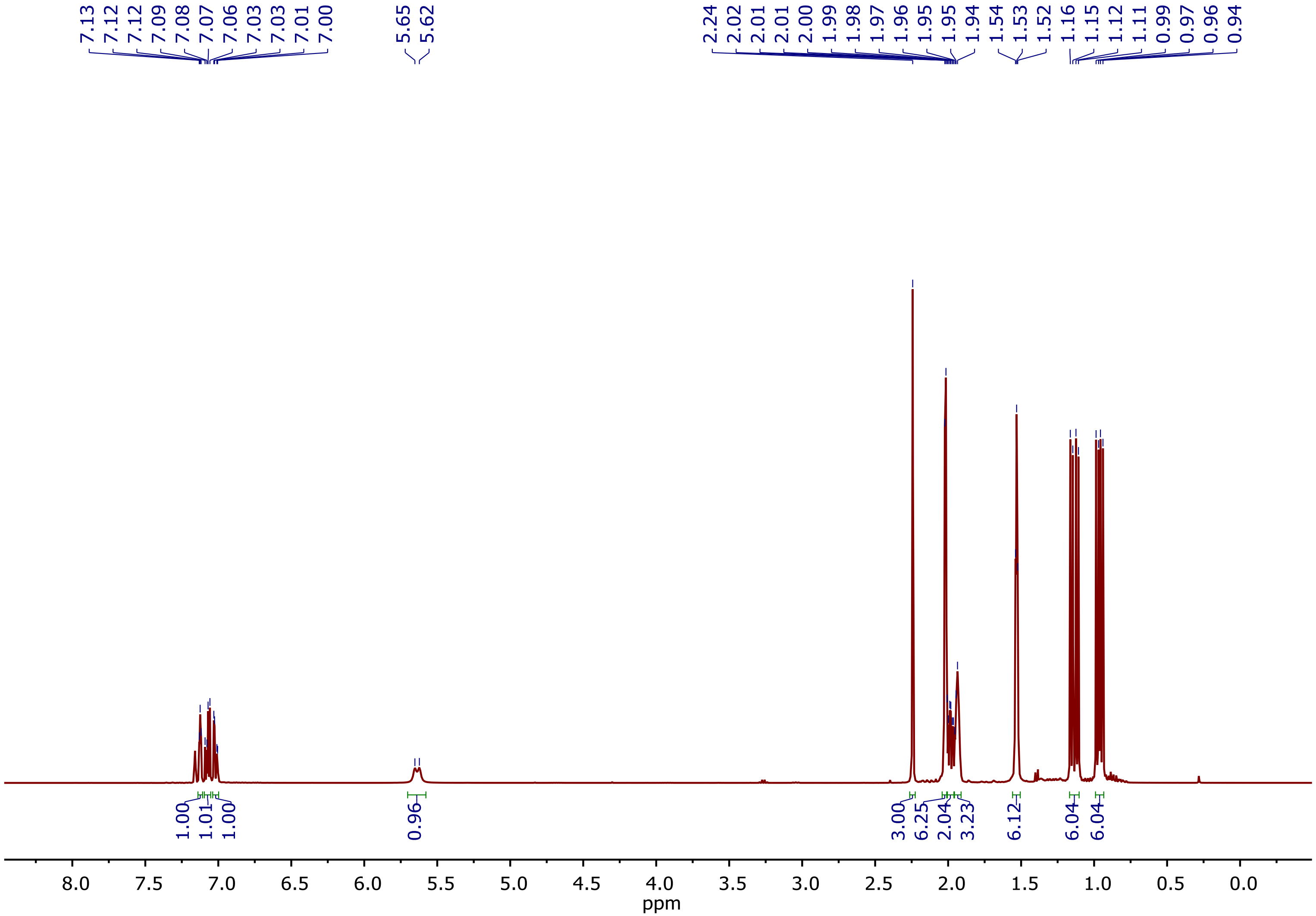

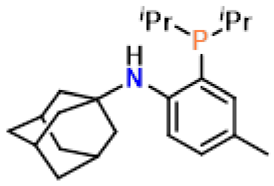

Figure S 45: ^{1}H NMR spectrum of **HPN^{Ad}** in C_6D_6 at 298 K.

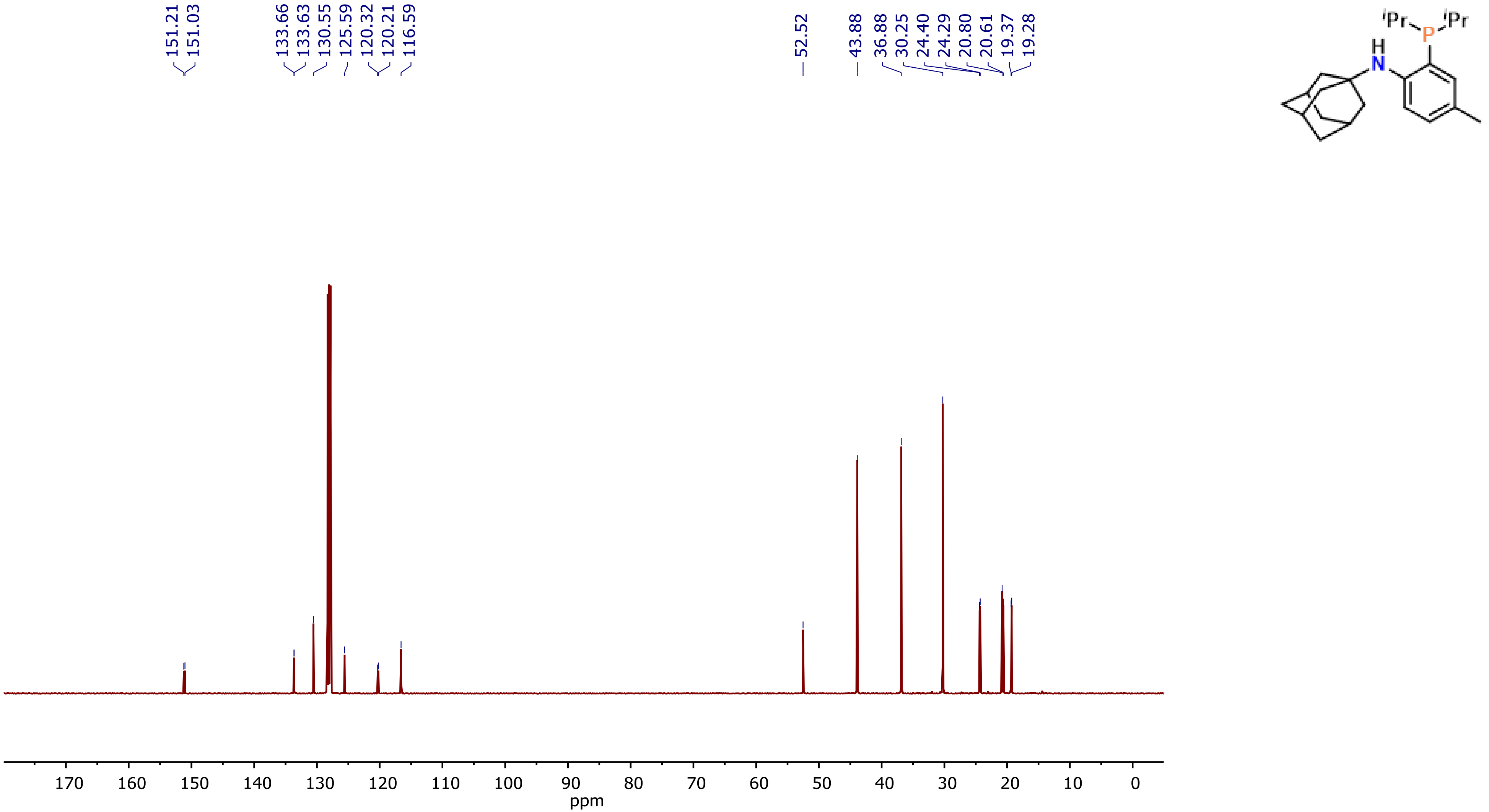

*Figure S 46: $^{13}C\{^{1}H\}$ NMR spectrum of **HPN^{Ad}** in C_6D_6 at 298 K.*

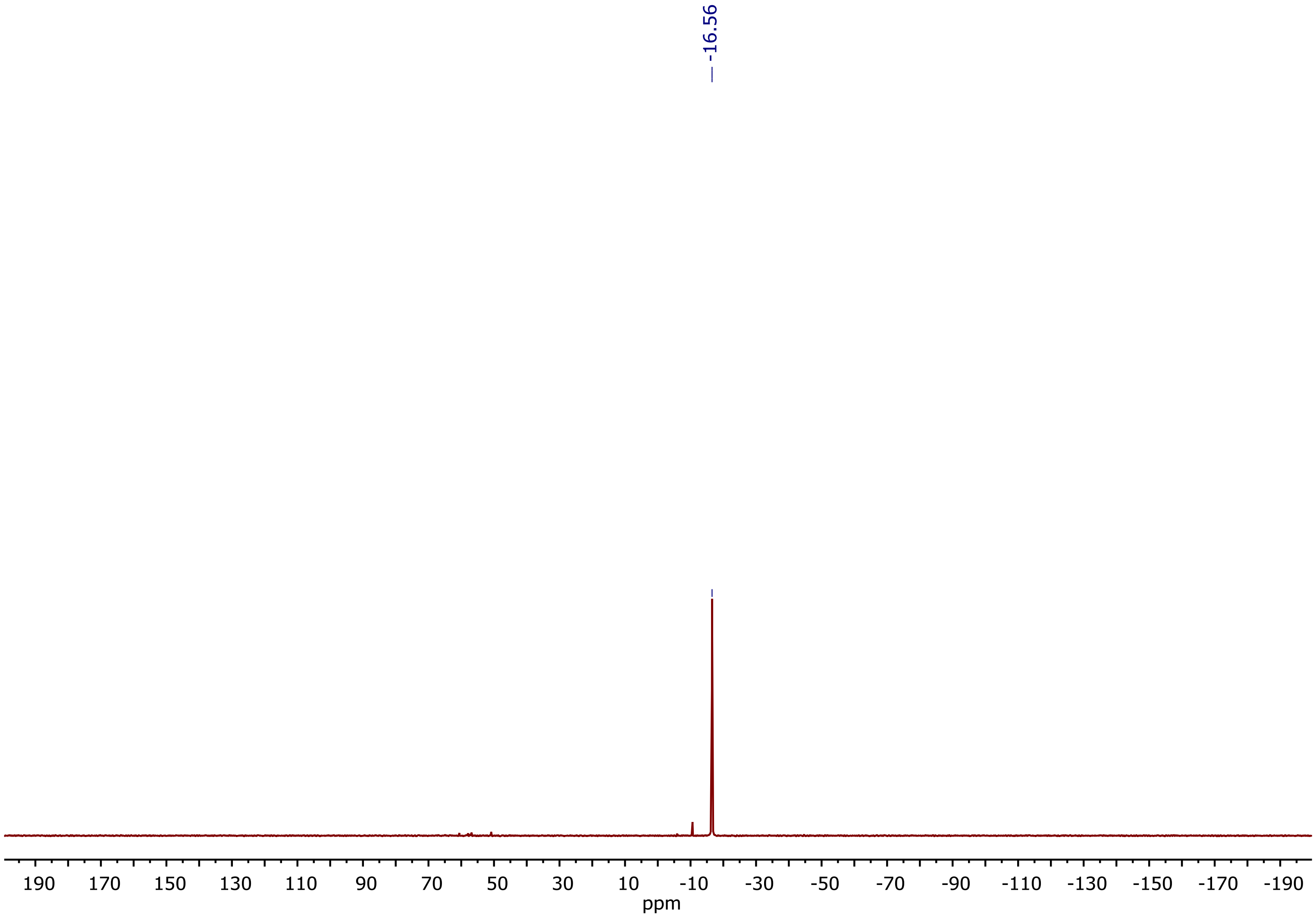

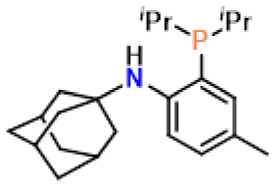

*Figure S 47: $^{31}P\{^{1}H\}$ NMR spectrum of **HPN^{Ad}** in C_6D_6 at 298 K.*

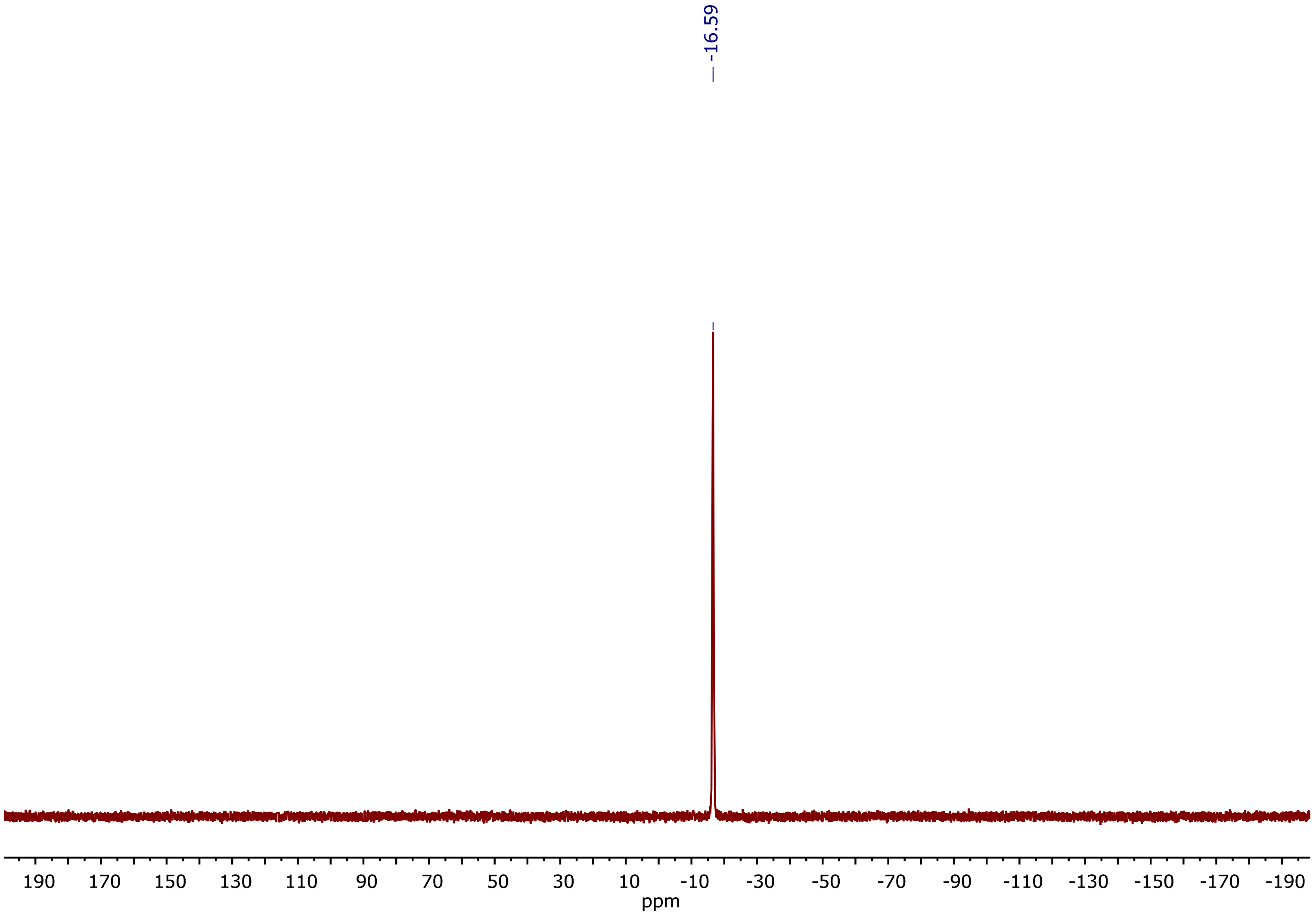

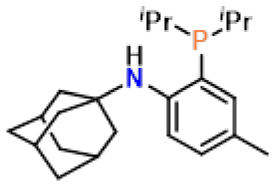

*Figure S 48: ^{31}P NMR spectrum of **HPN^{Ad}** in C_6D_6 at 298 K.*

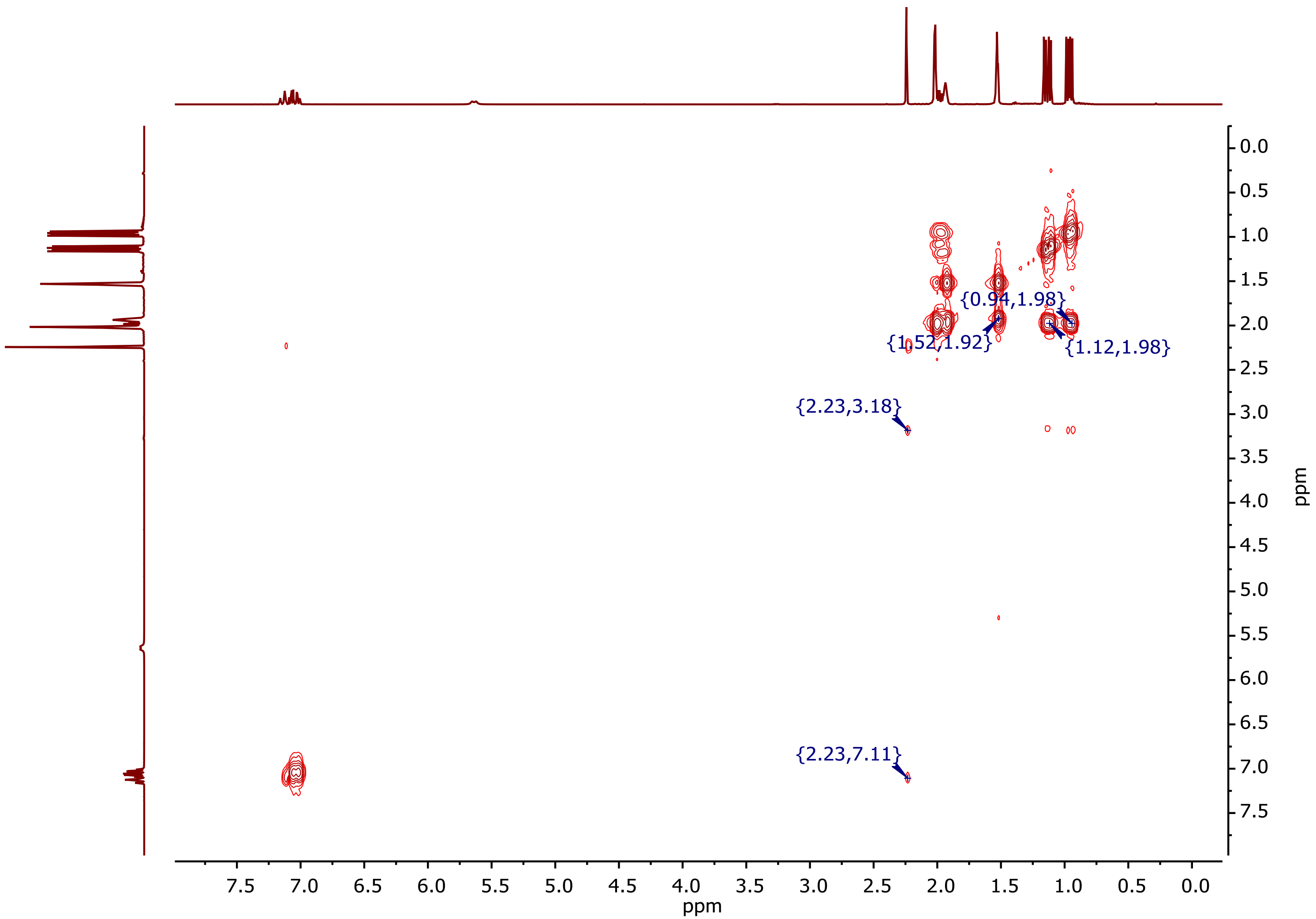

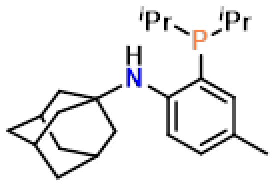

*Figure S 49: ^1H-^1H COSY NMR spectrum of **HPN^{Ad}** in C_6D_6 at 298 K.*

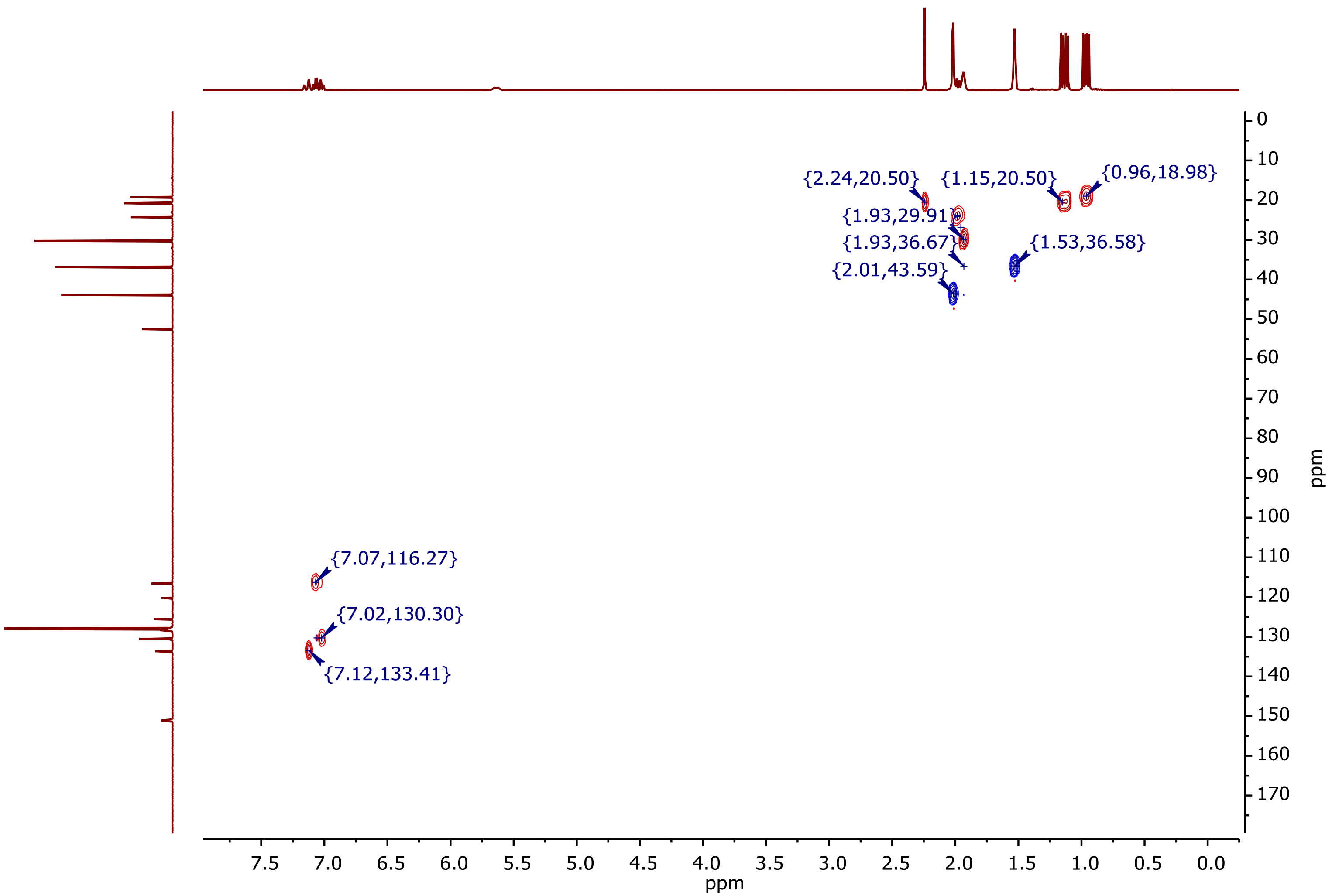

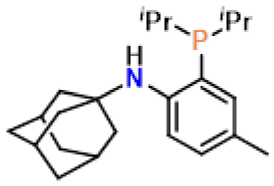

*Figure S 50: ^{1}H-^{13}C HSQC NMR spectrum of **HPN^{Ad}** in C_6D_6 at 298 K.*

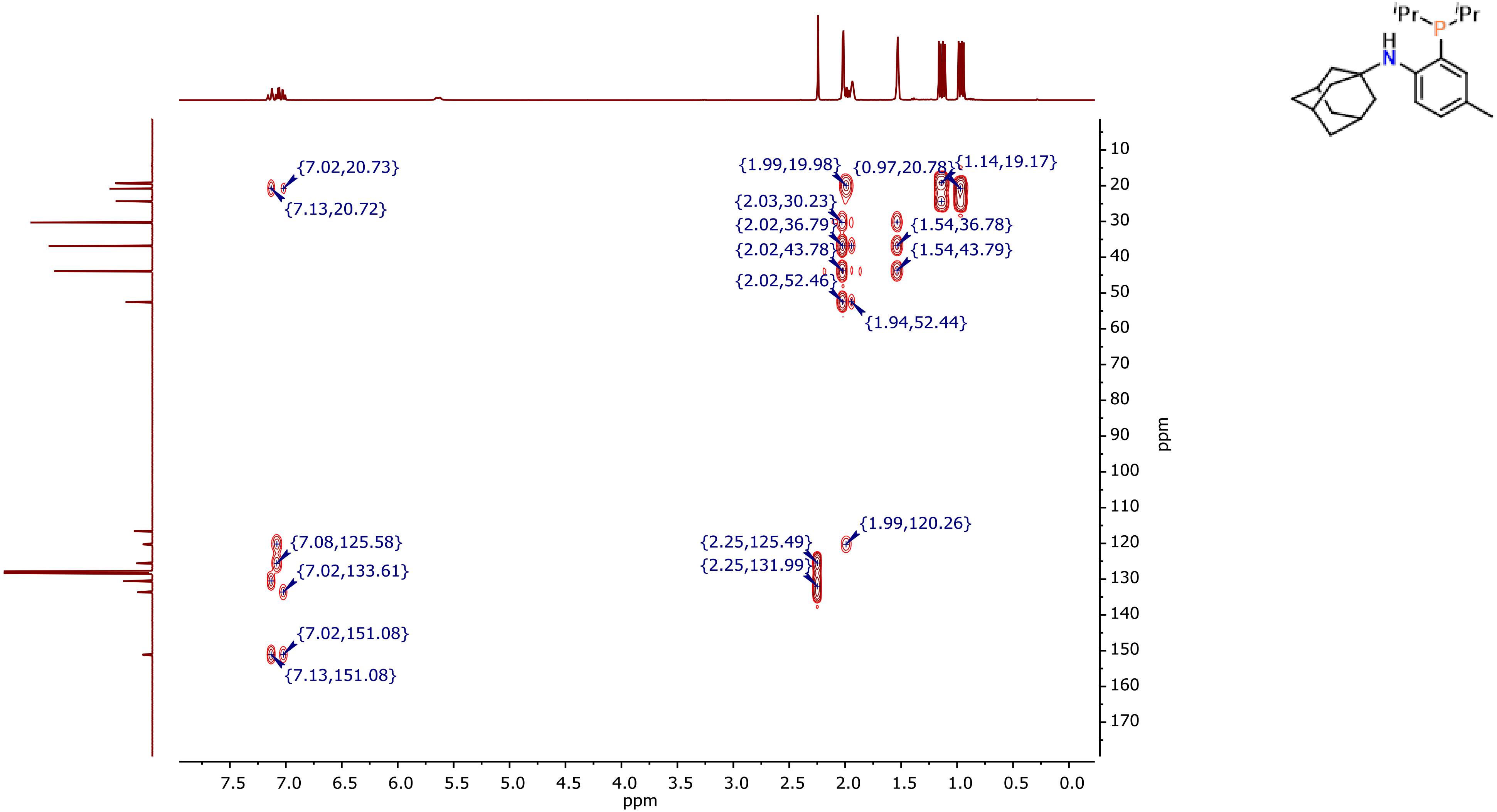

*Figure S 51: ^{1}H-^{13}C HMBC NMR spectrum of **HPN^{Ad}** in C_6D_6 at 298 K.*

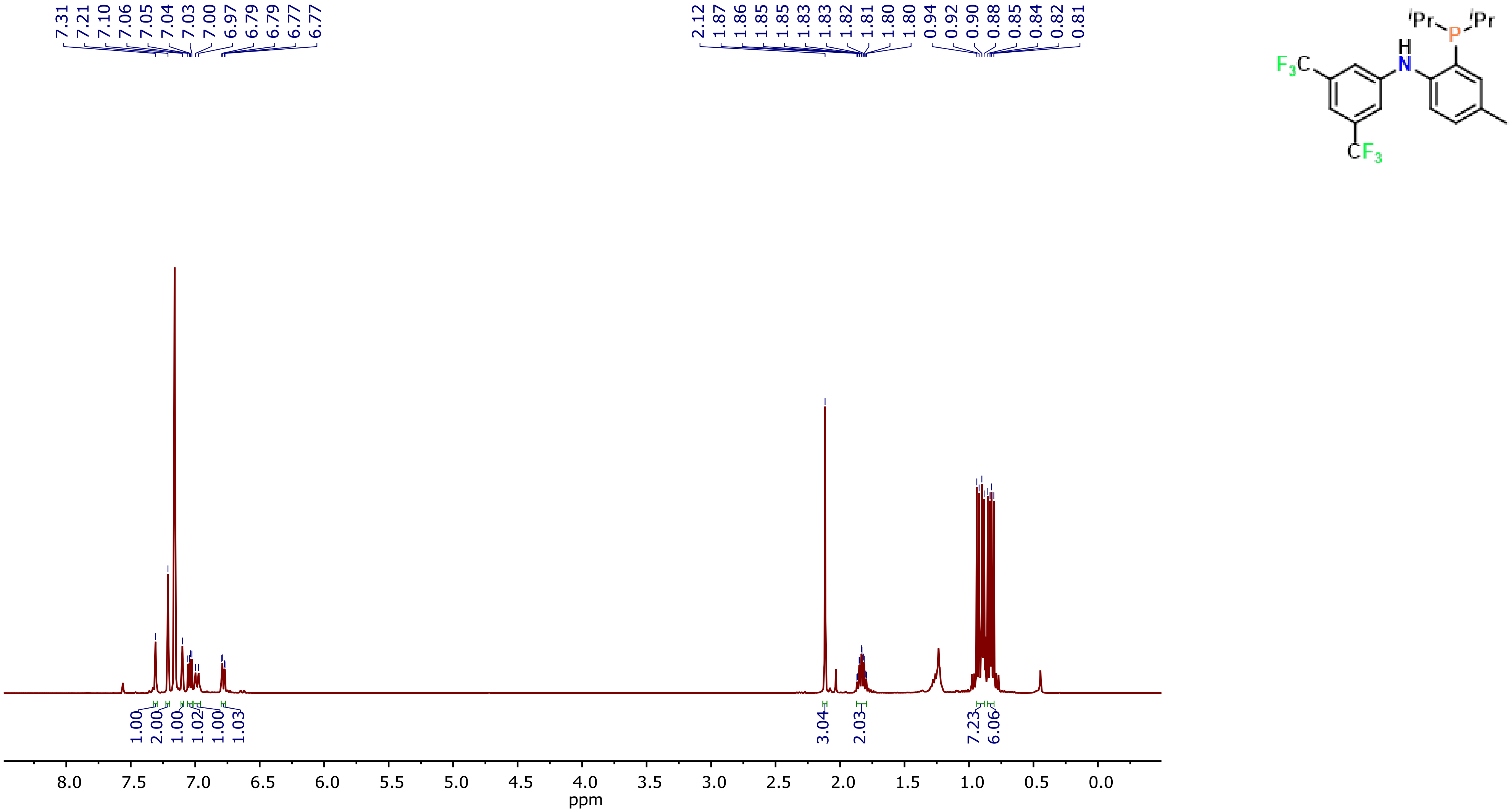

Figure S 52: ^{1}H NMR spectrum of **HPN^{3,5CF3}** in C_6D_6 at 298 K.

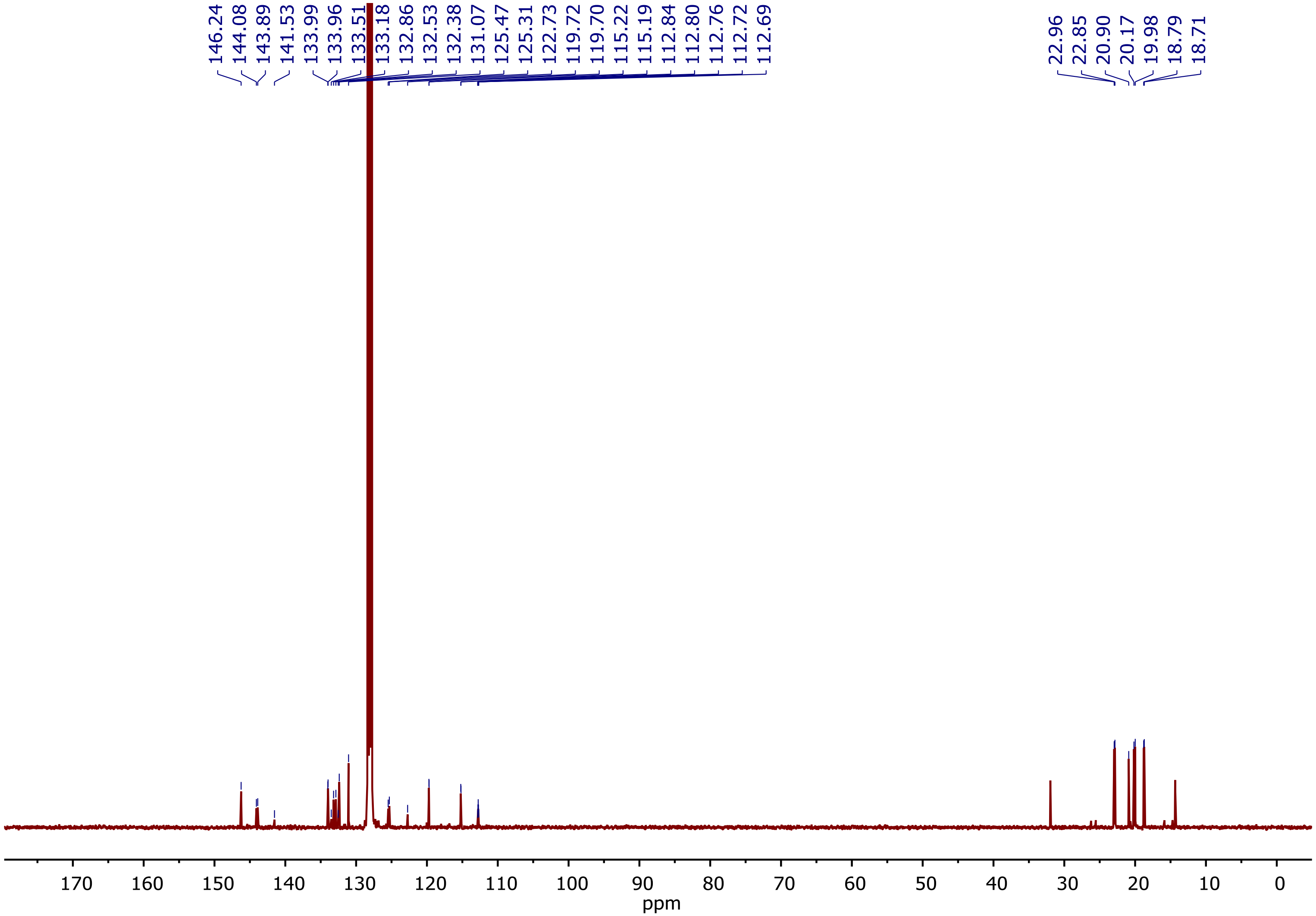

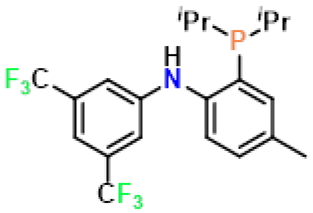

*Figure S 53: $^{13}C\{^{1}H\}$ NMR spectrum of **HPN^{3,5CF3}** in C_6D_6 at 298 K.*

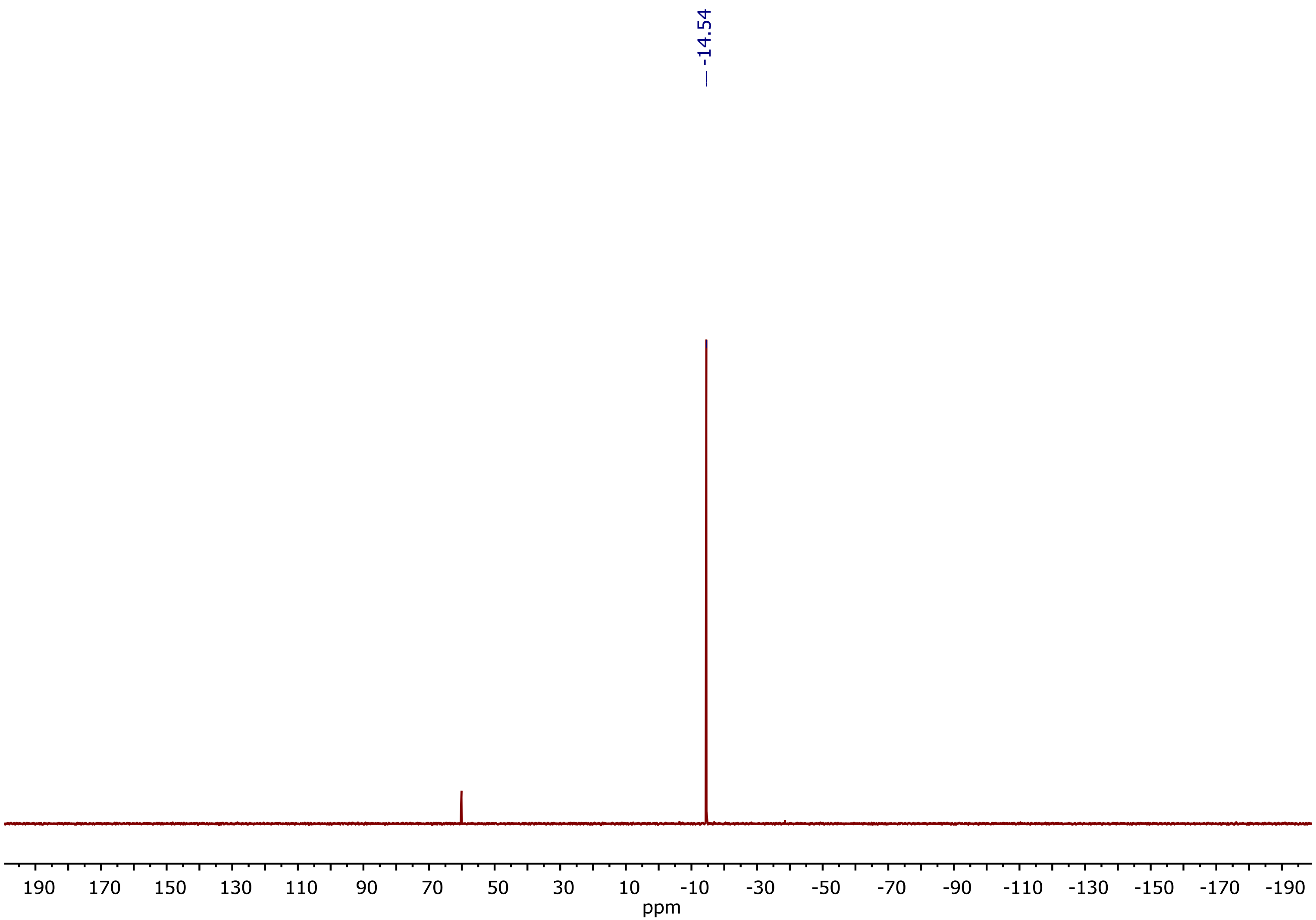

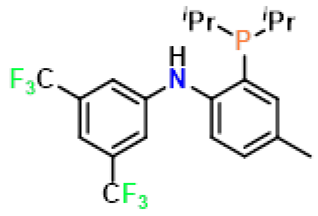

*Figure S 54: $^{31}P\{^1H\}$ NMR spectrum of **HPN^{3,5CF3}** in C_6D_6 at 298 K.*

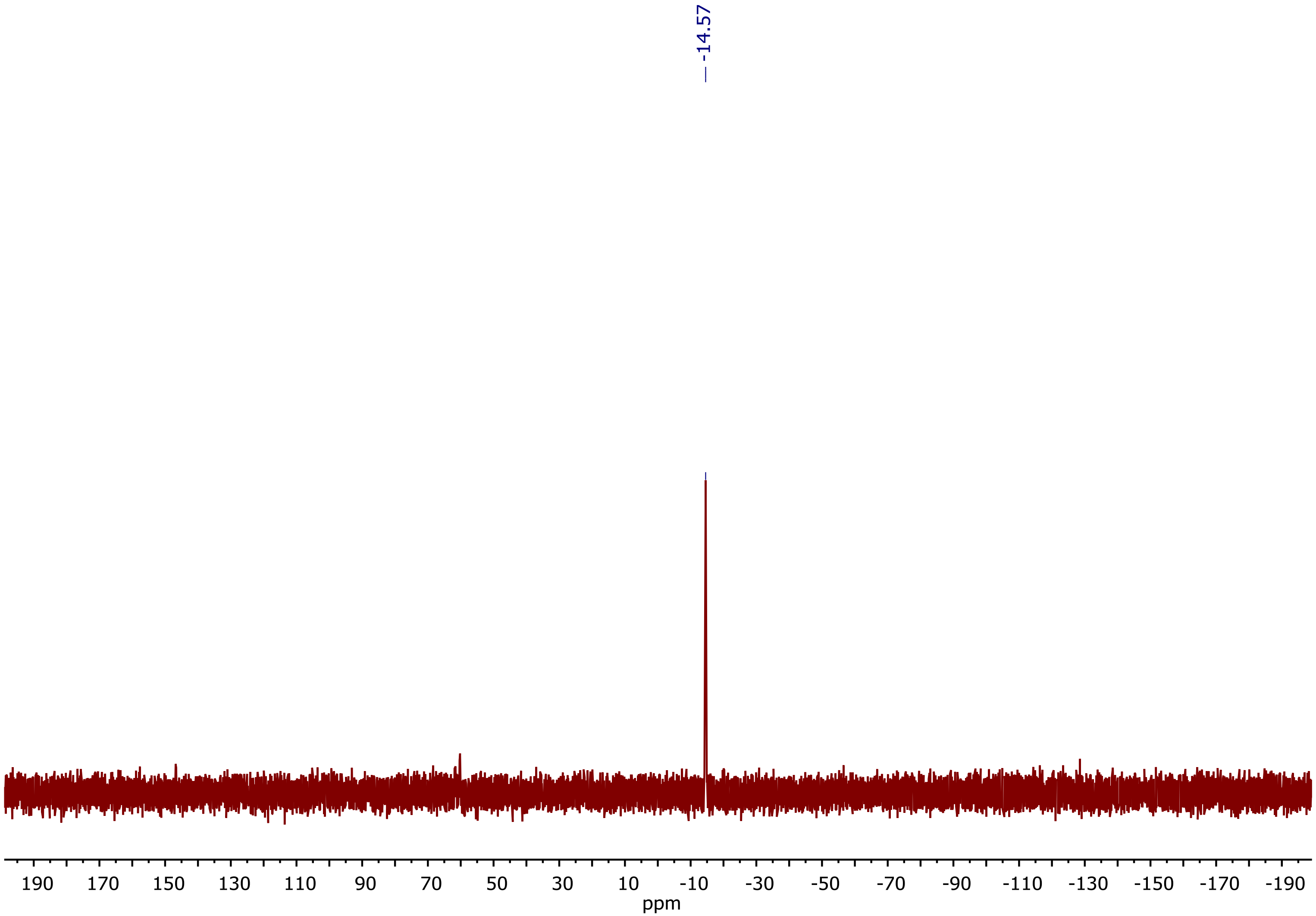

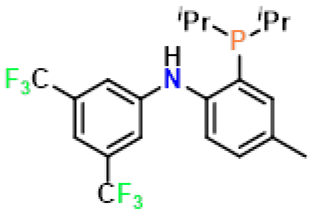

*Figure S 55: ^{31}P NMR spectrum of **HPN^{3,5CF3}** in C_6D_6 at 298 K.*

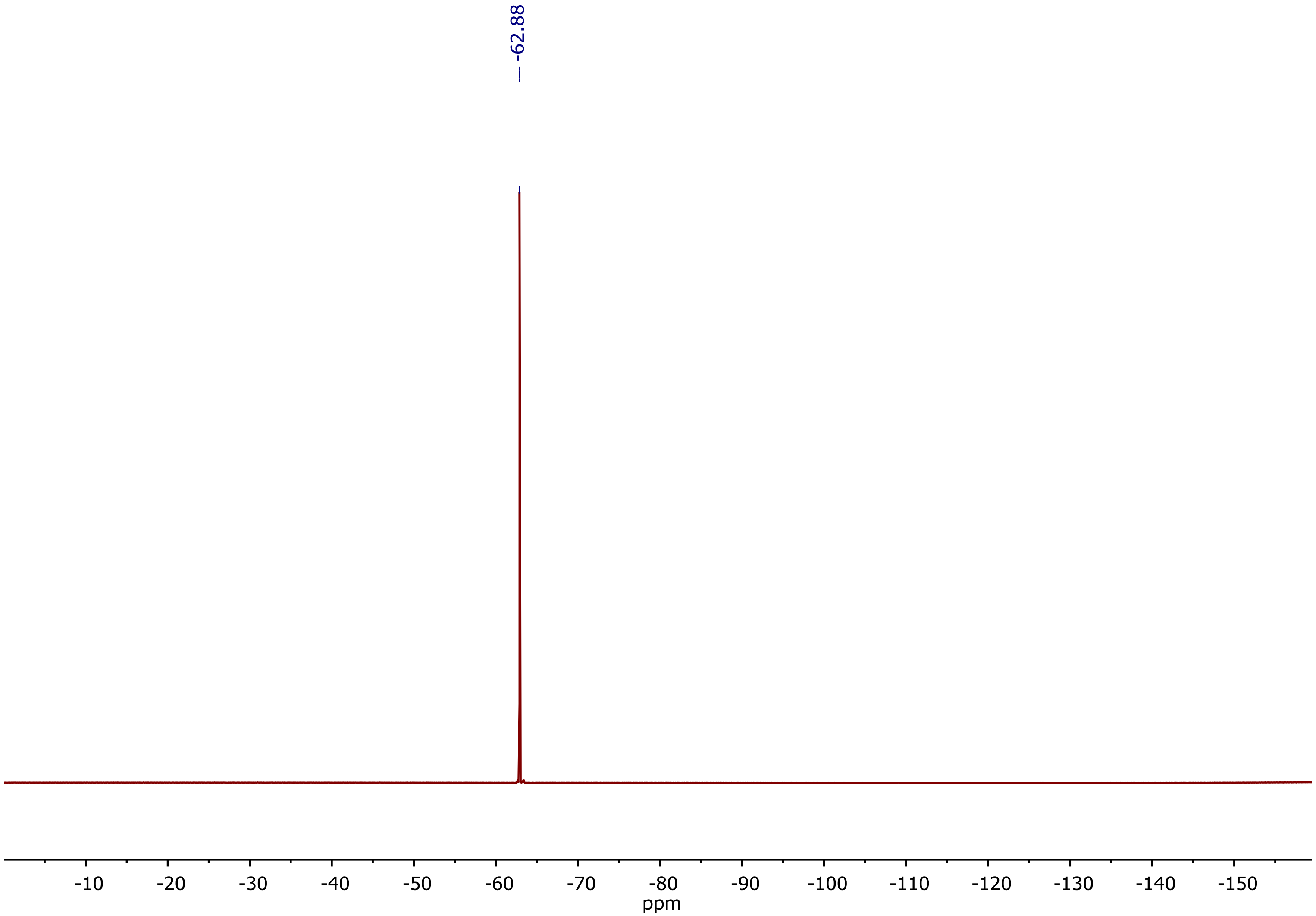

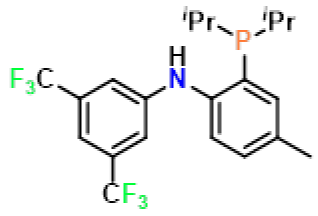

*Figure S 56: ^{19}F NMR spectrum of **HPN^{3,5CF3}** in C_6D_6 at 298 K.*

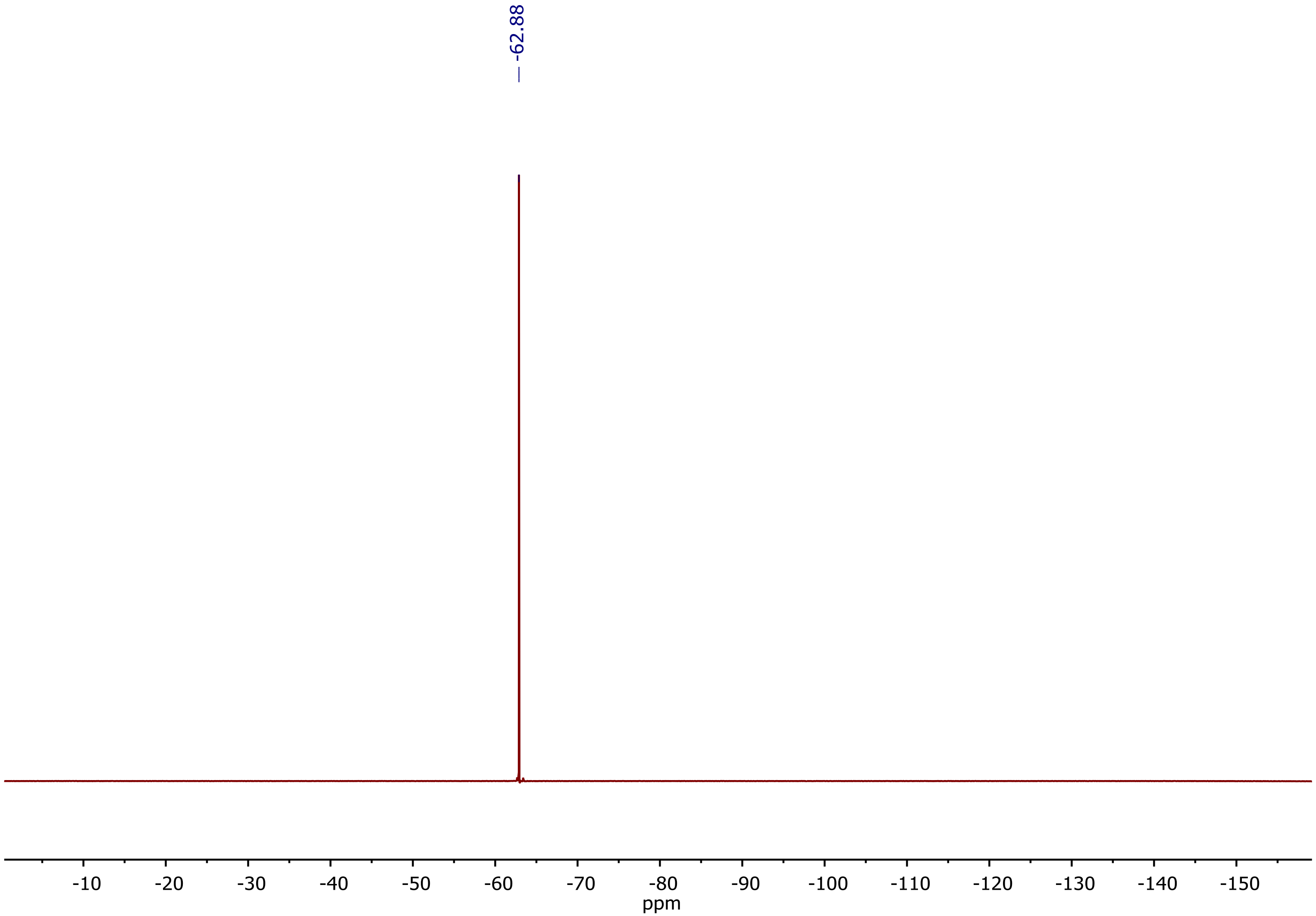

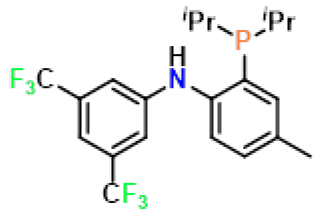

Figure S 57: $^{19}F\{^{1}H\}$ NMR spectrum of **$HPN^{3,5CF3}$** in C_6D_6 at 298 K.

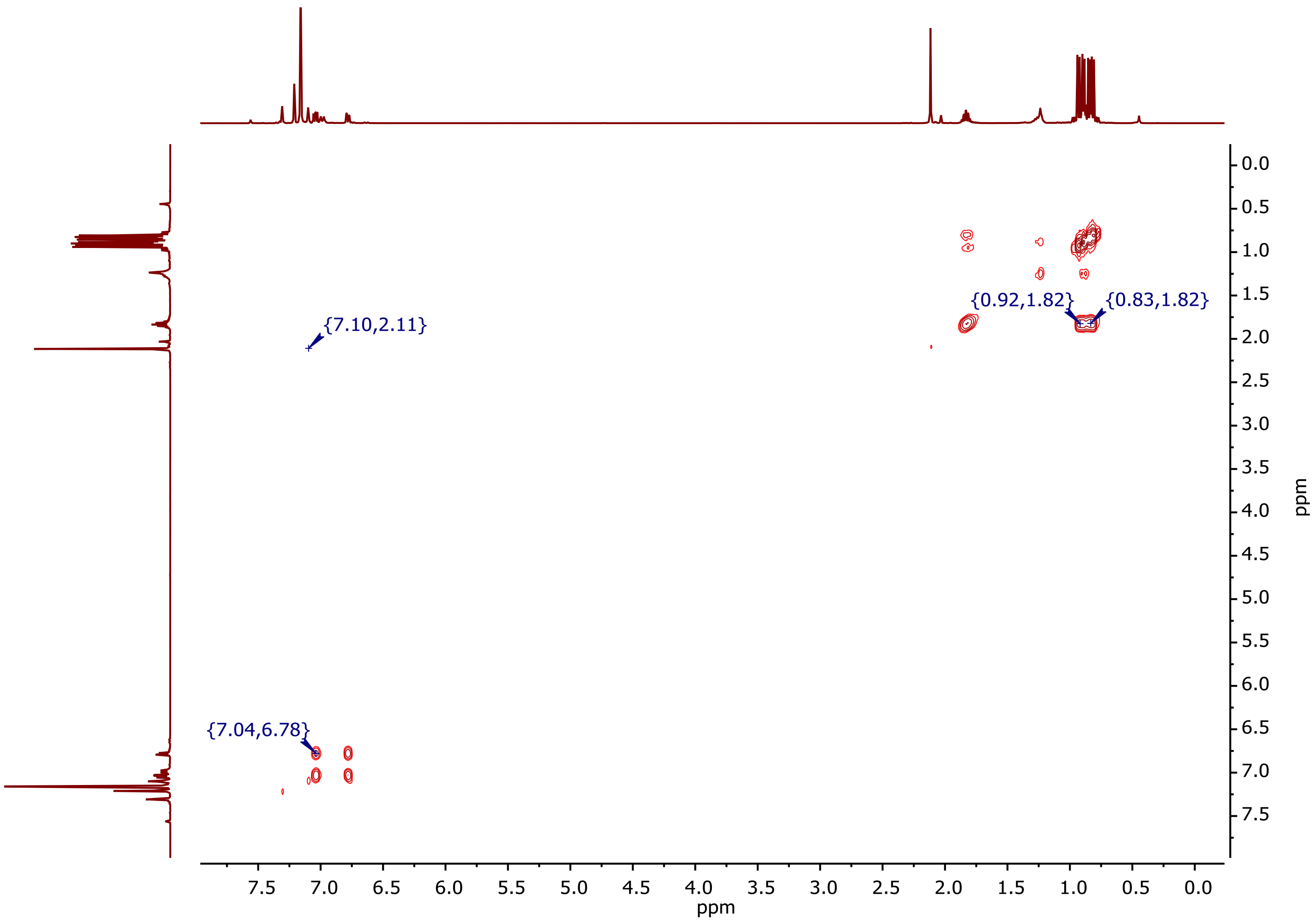

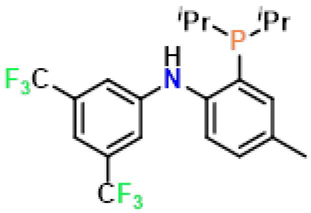

*Figure S 58: ^{1}H-^{1}H COSY NMR spectrum of **HPN^{3,5CF3}** in C_6D_6 at 298 K.*

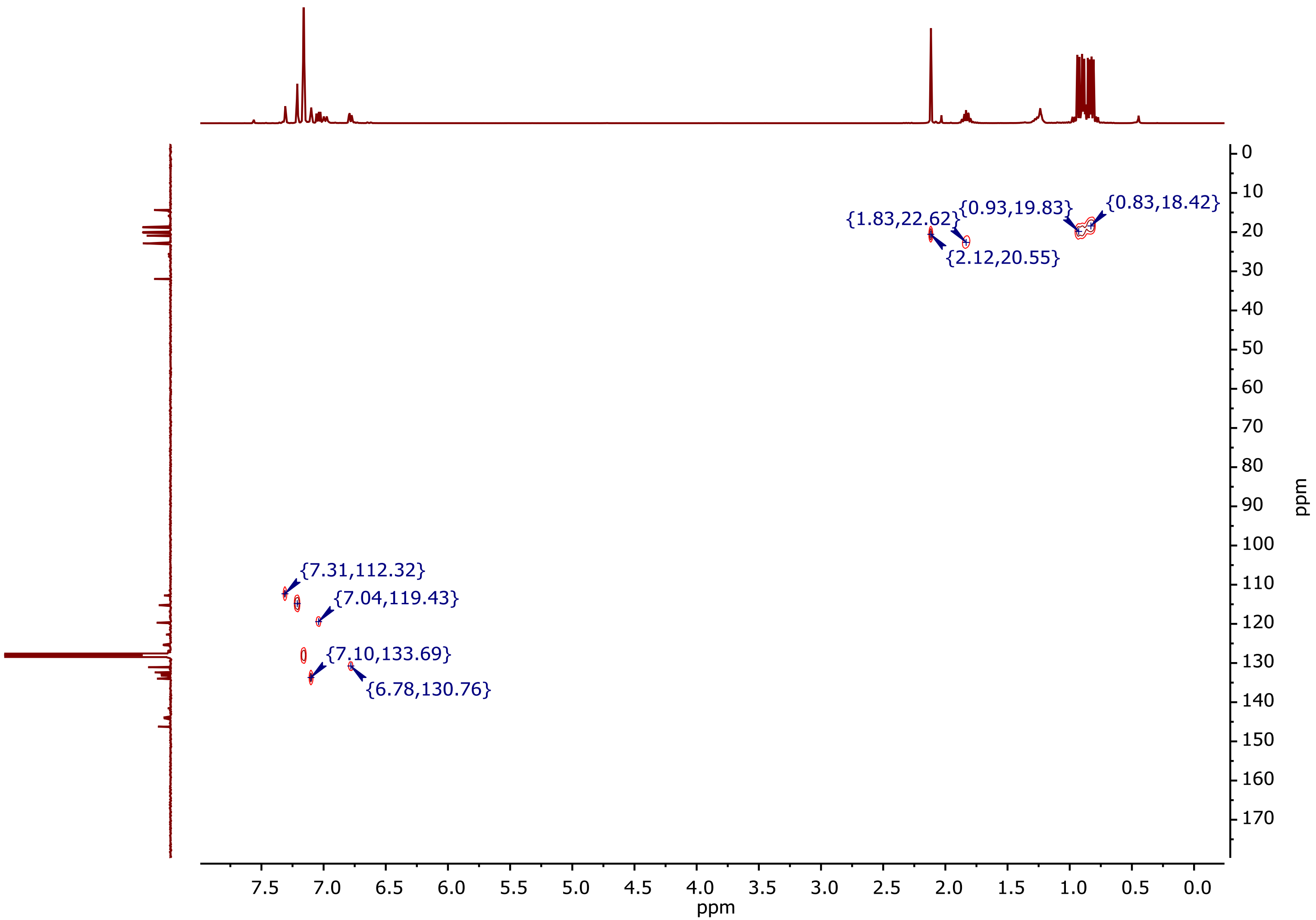

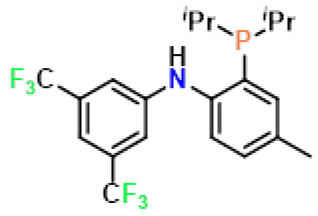

*Figure S 59: ^{1}H-^{13}C HSQC NMR spectrum of **HPN^{3,5CF3}** in C_6D_6 at 298 K.*

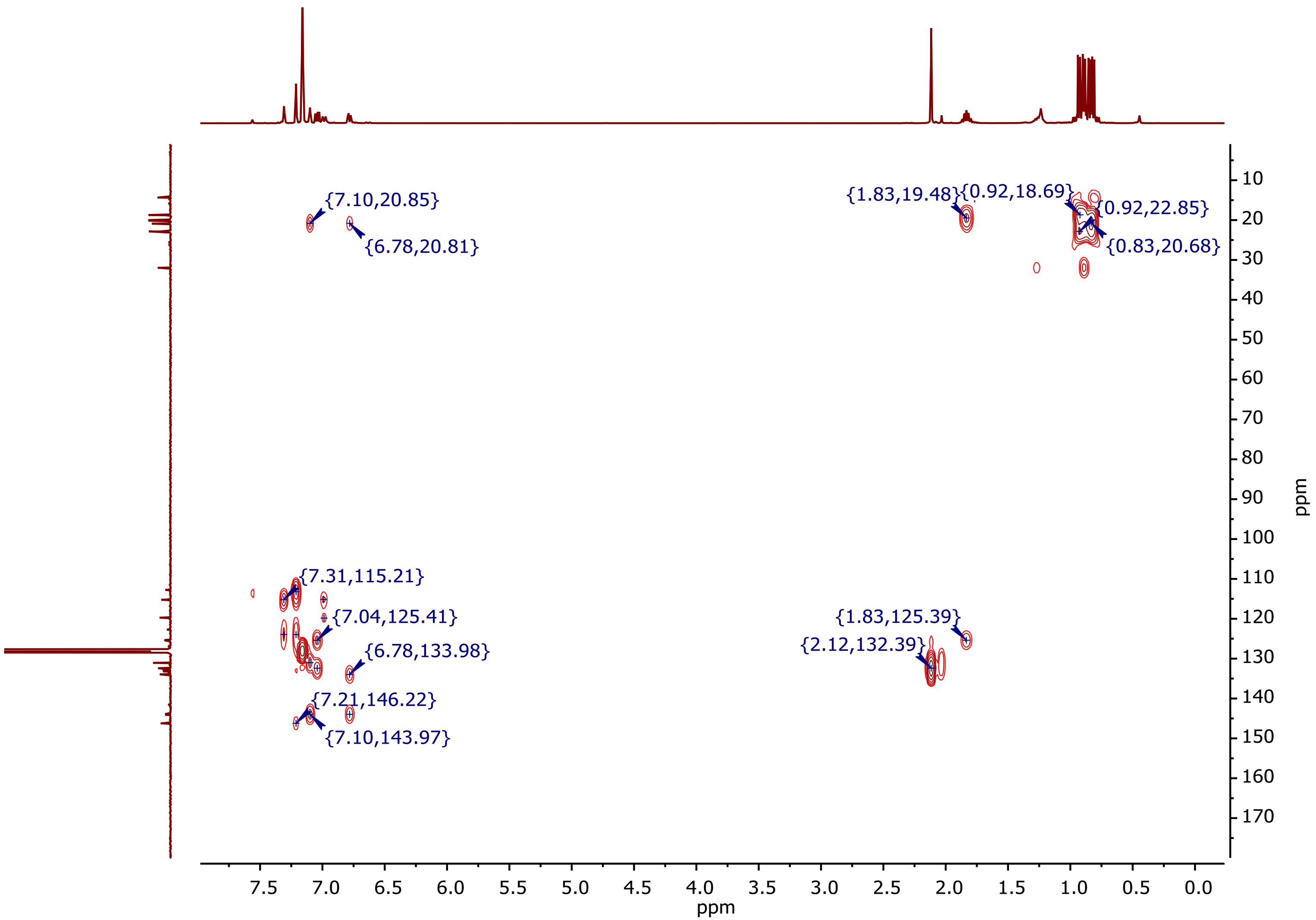

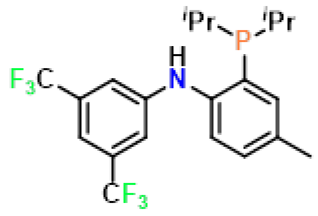

Figure S 60: ^{1}H-^{13}C HMBC NMR spectrum of **HPN^{3,5CF3}** in C_6D_6 at 298 K.

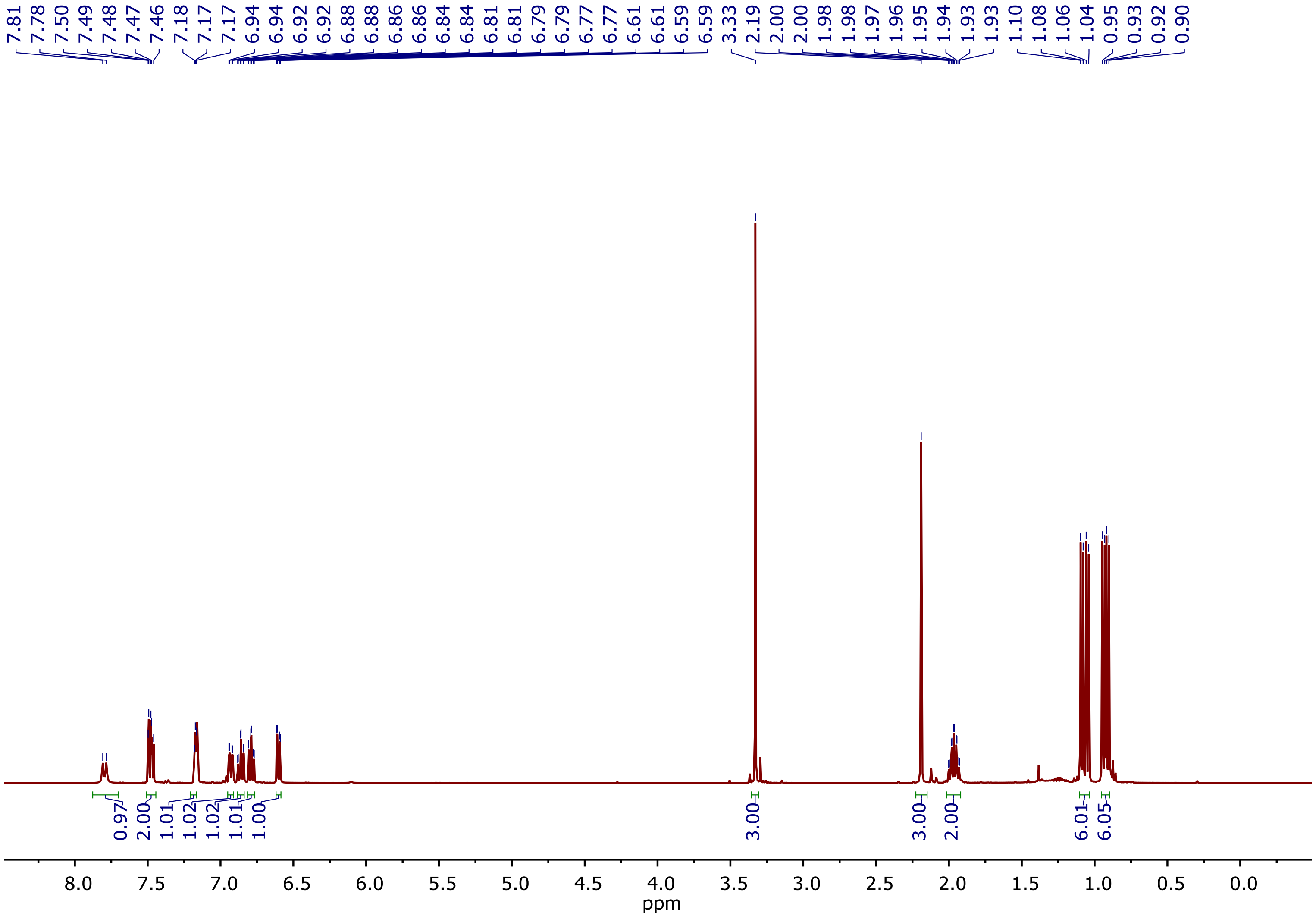

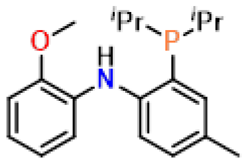

*Figure S 61: ^{1}H NMR spectrum of **HPN^{OMe}** in C_6D_6 at 298 K.*

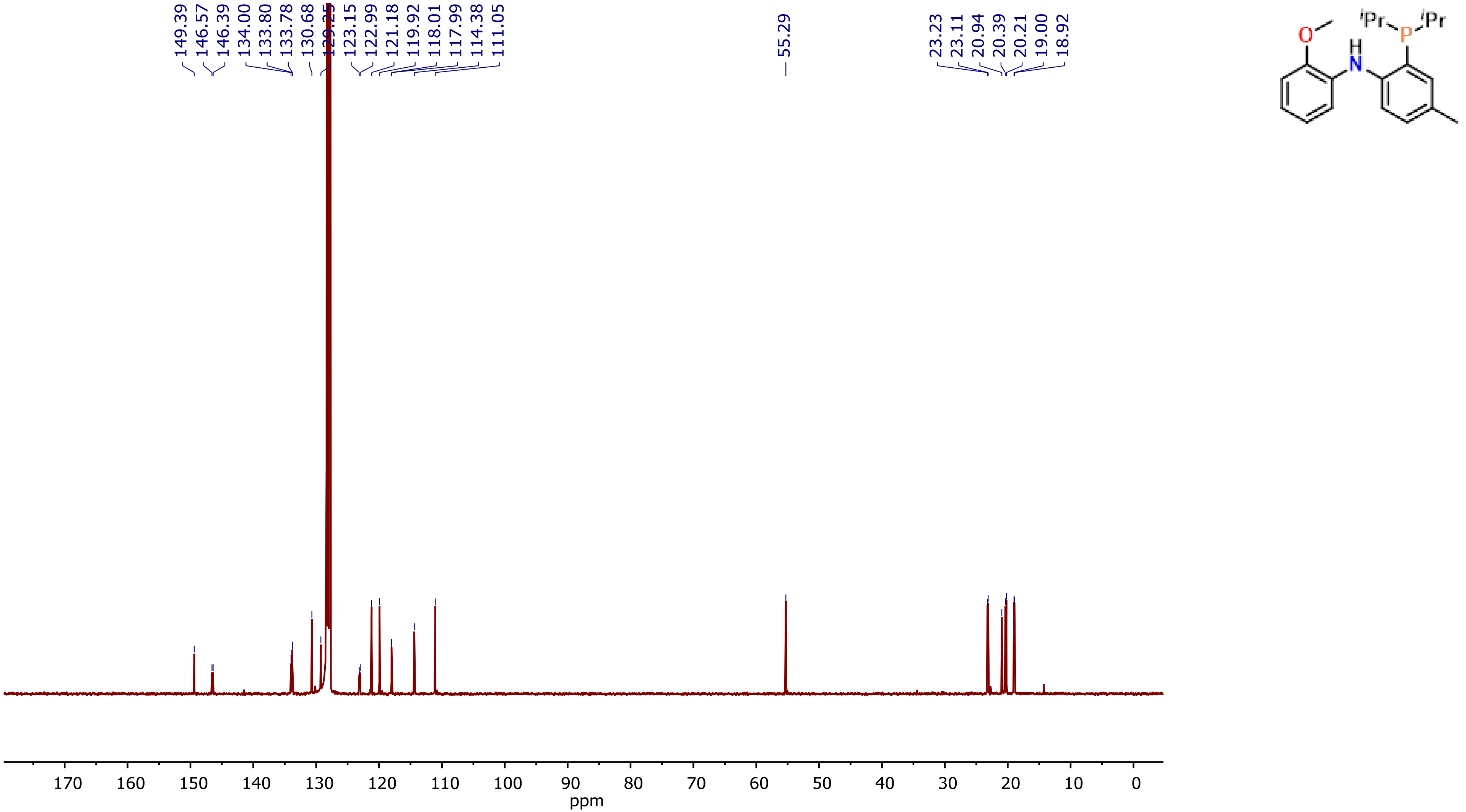

*Figure S 62: $^{13}C\{^{1}H\}$ NMR spectrum of **HPN^{OMe}** in C_6D_6 at 298 K.*

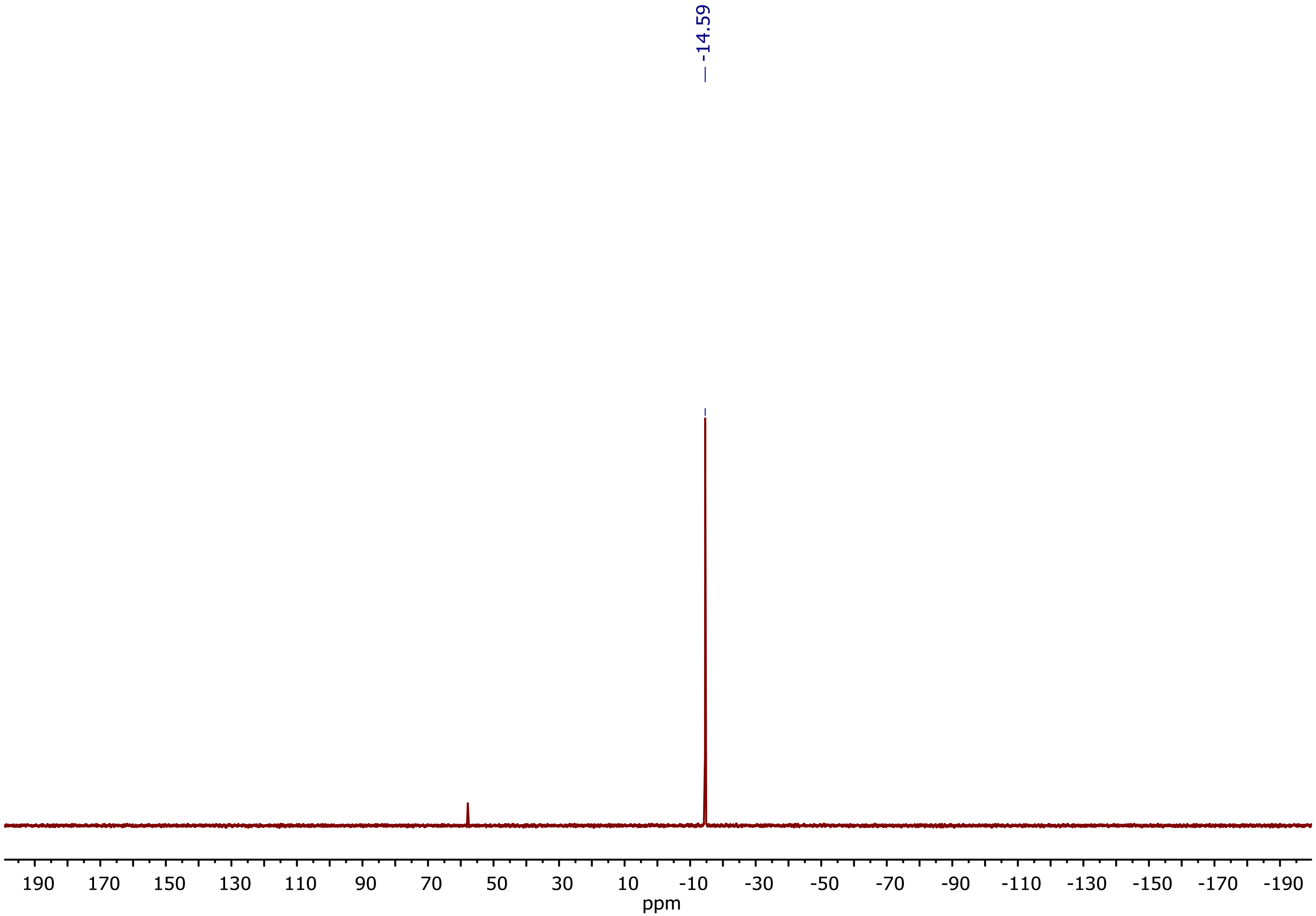

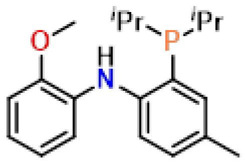

*Figure S 63: $^{31}P\{^1H\}$ NMR spectrum of **HPN^{OMe}** in C_6D_6 at 298 K.*

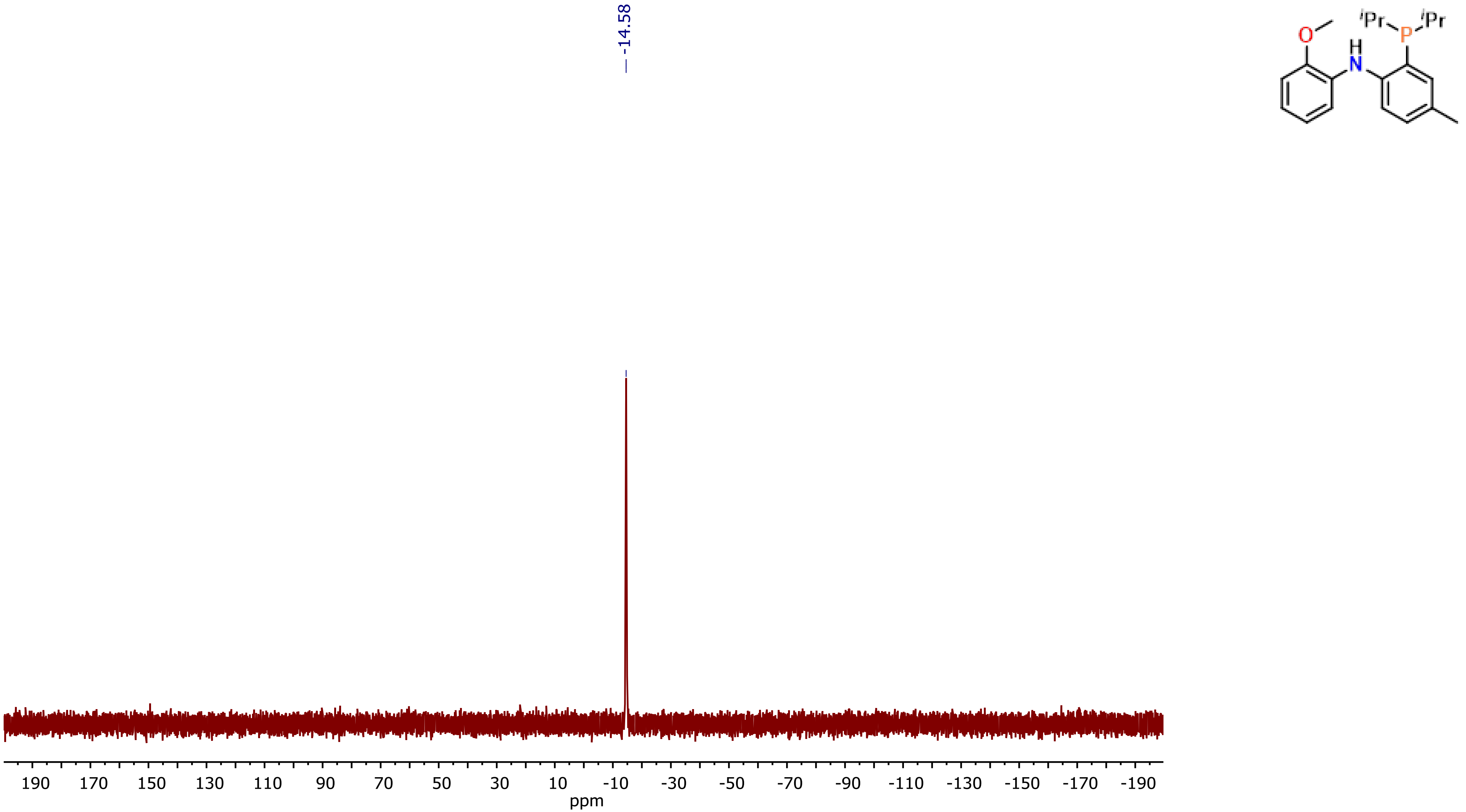

*Figure S 64: ^{31}P NMR spectrum of **HPN^{OMe}** in C_6D_6 at 298 K.*

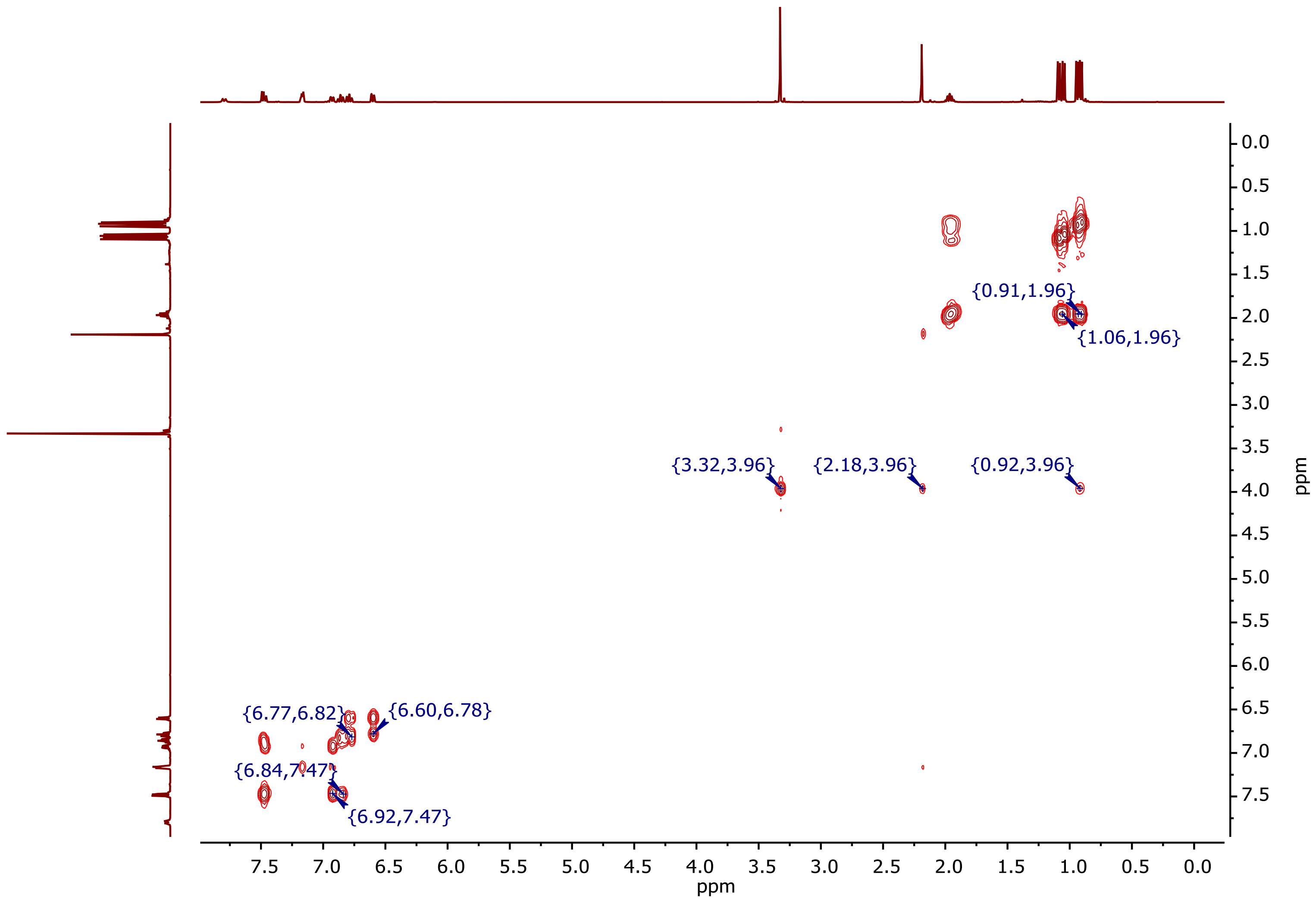

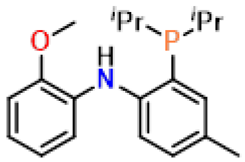

Figure S 65: ^{1}H-^{1}H COSY NMR spectrum of **HPN^{OMe}** in C_6D_6 at 298 K.

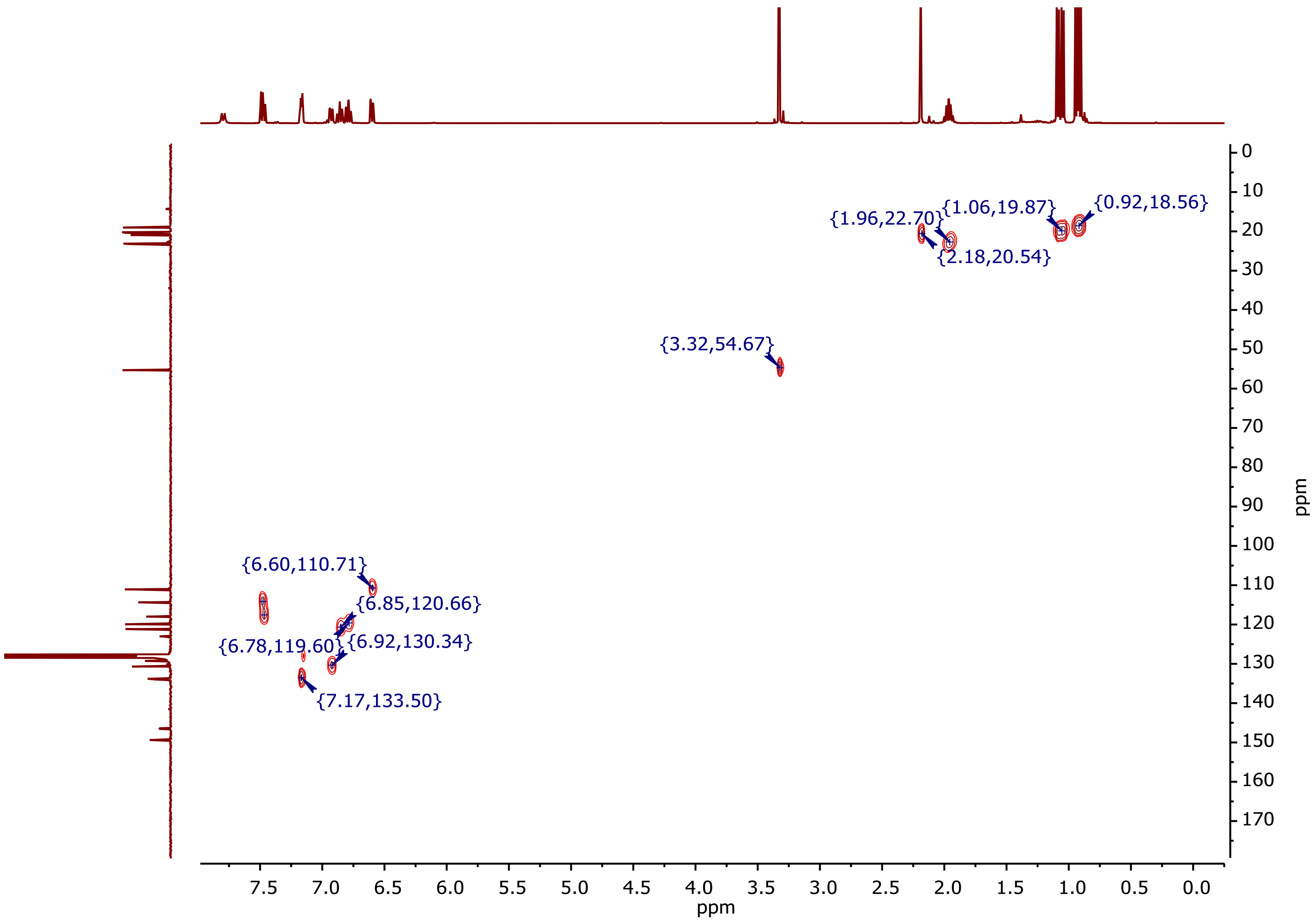

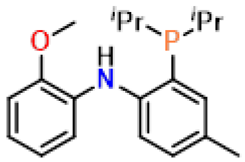

Figure S 66: ^{1}H-^{13}C HSQC NMR spectrum of **HPN^{OMe}** in C_6D_6 at 298 K.

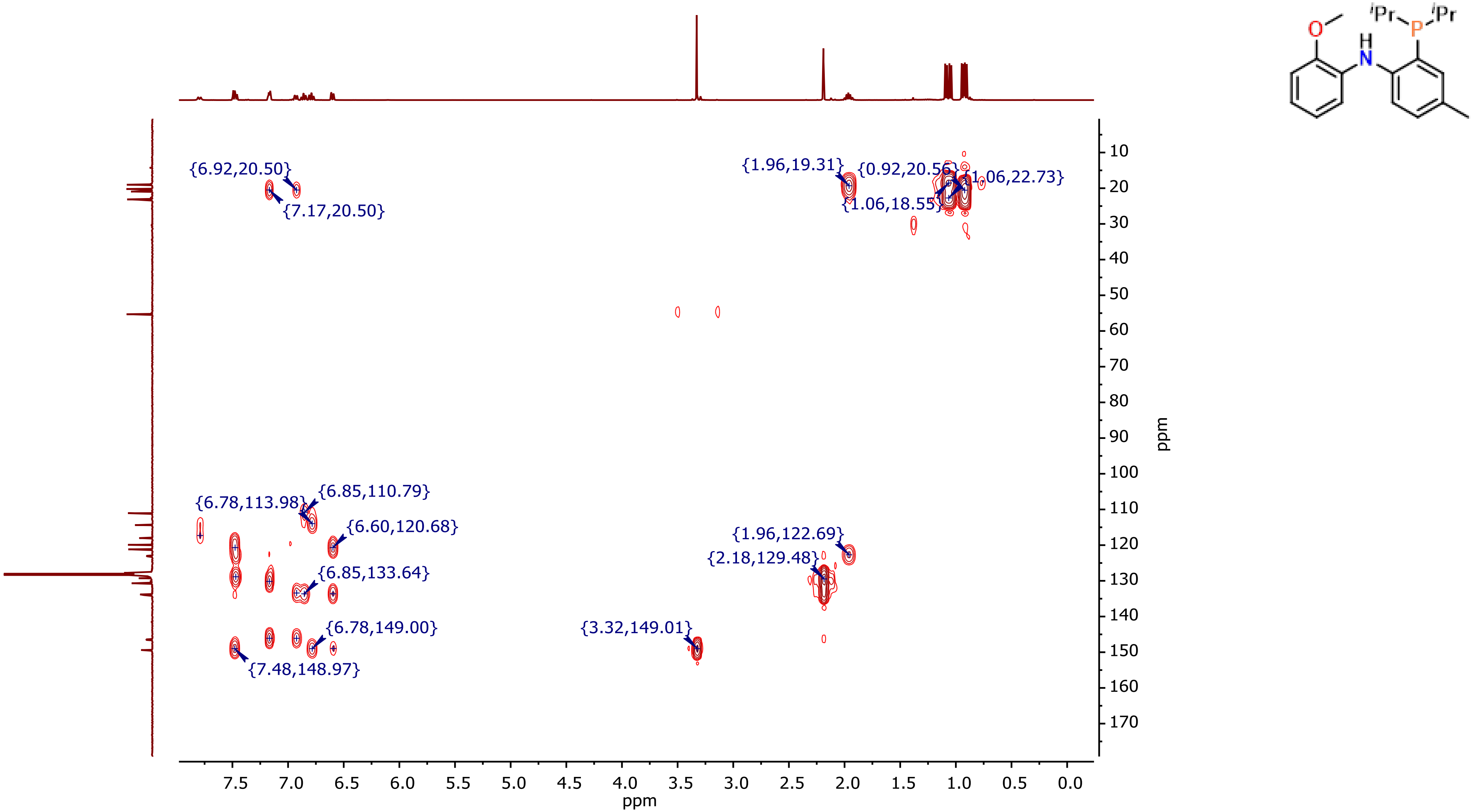

Figure S 67: ^{1}H-^{13}C HMBC NMR spectrum of **HPN^{OMe}** in C_6D_6 at 298 K.

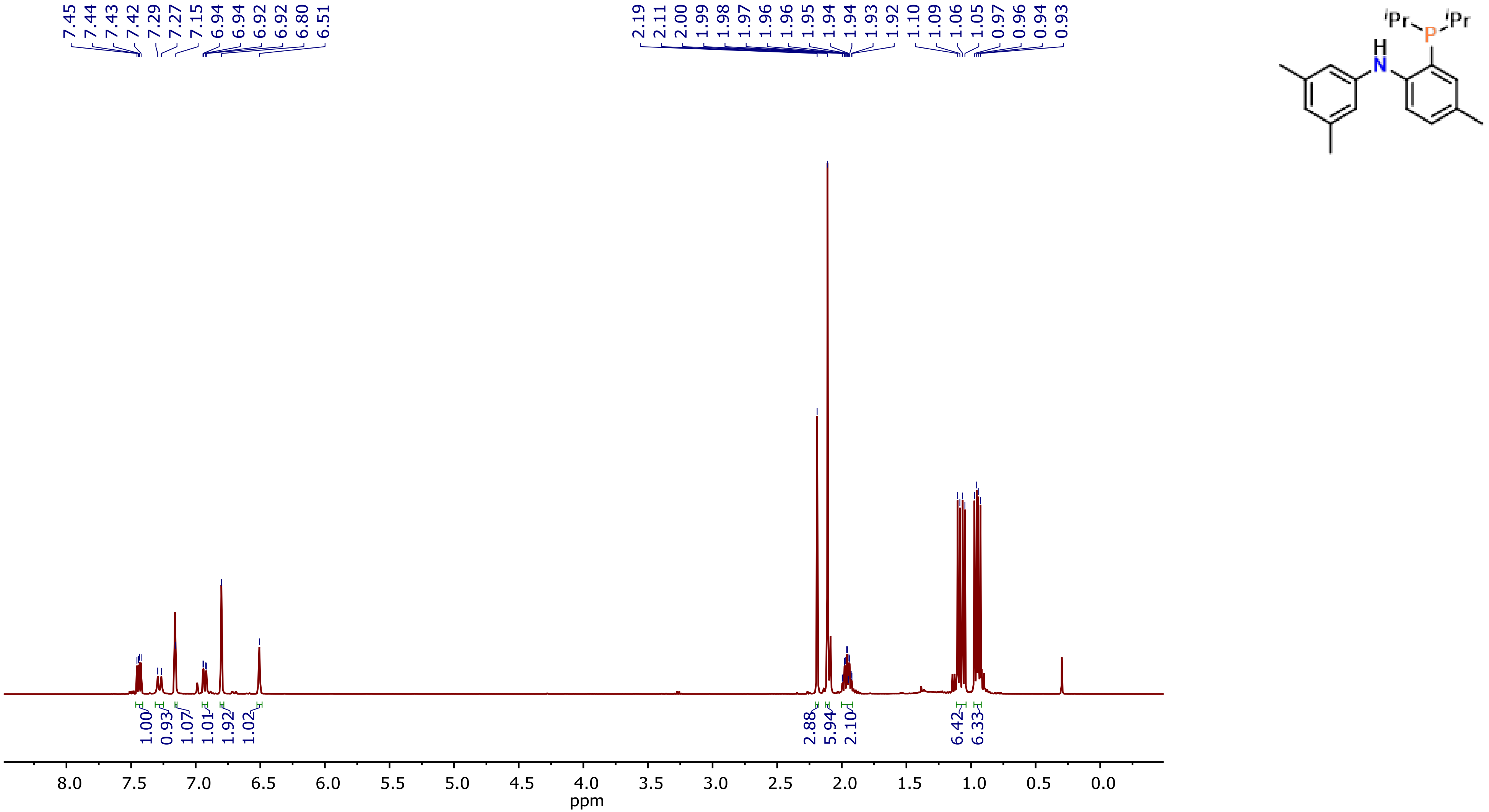

*Figure S 68: ^{1}H NMR spectrum of **HPN^{3,5Me}** in C_6D_6 at 298 K.*

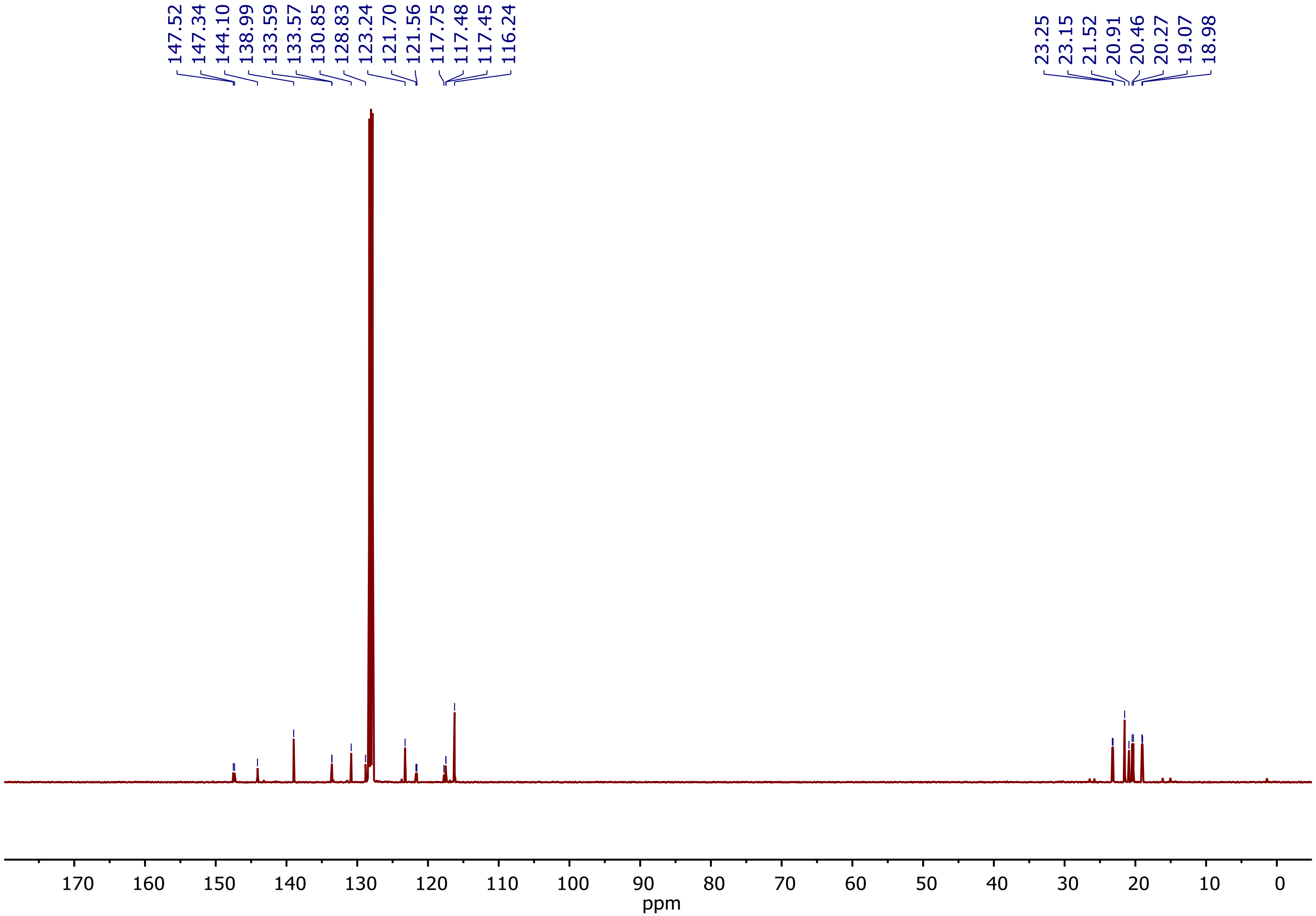

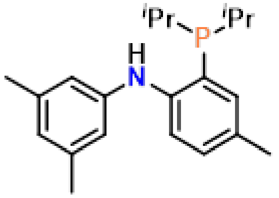

Figure S 69: $^{13}C\{^1H\}$ NMR spectrum of ***$HPN^{3,5Me}$*** *in C_6D_6 at 298 K.*

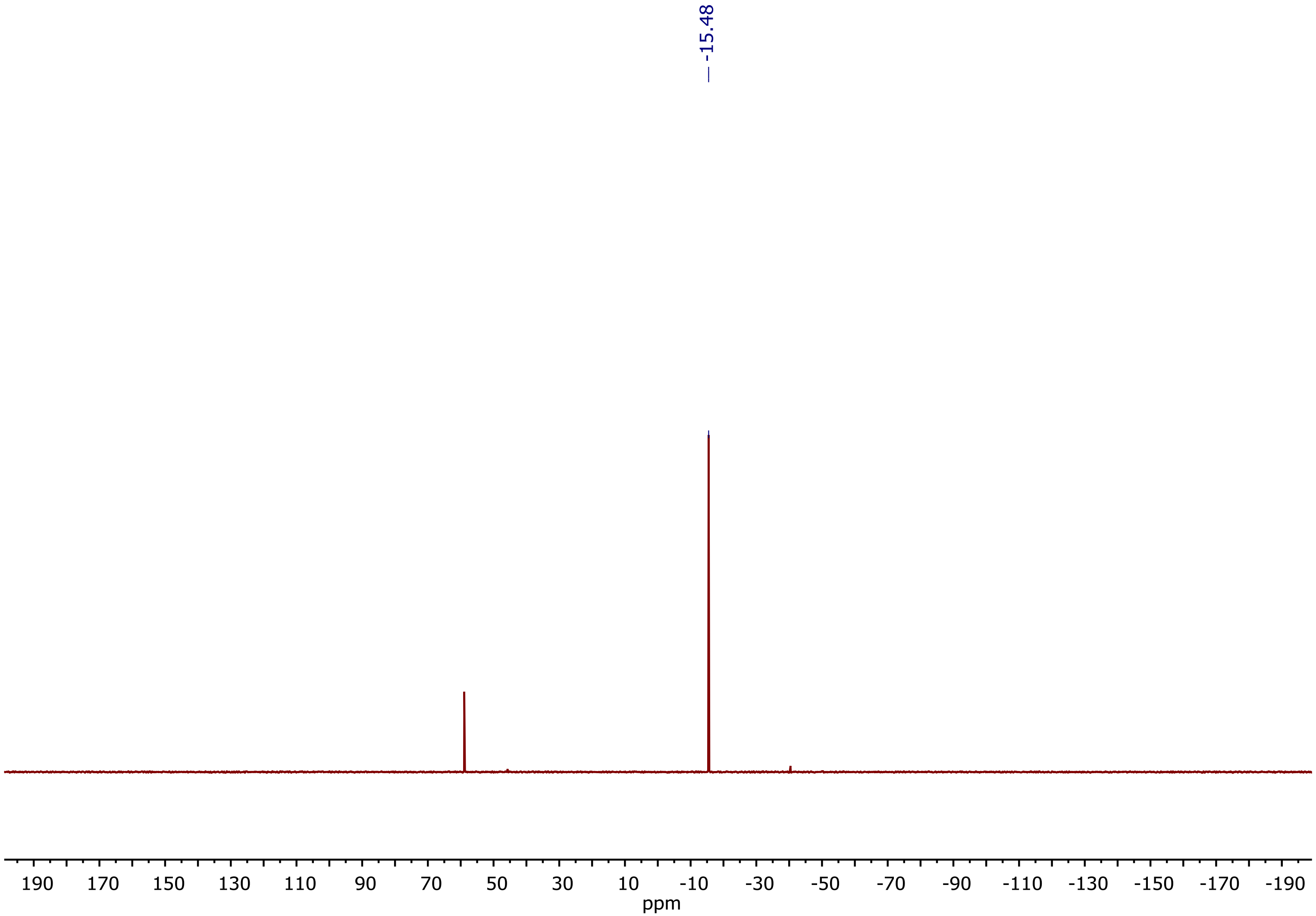

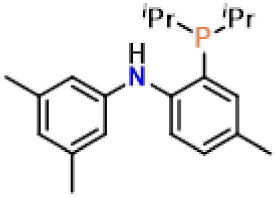

*Figure S 70: $^{31}P\{^{1}H\}$ NMR spectrum of **HPN^{3,5Me}** in C_6D_6 at 298 K. The signal at ca. 58 ppm corresponds to an unknown impurity (most likely arising from slow oxidation of the phosphine ligand in air over time.)*

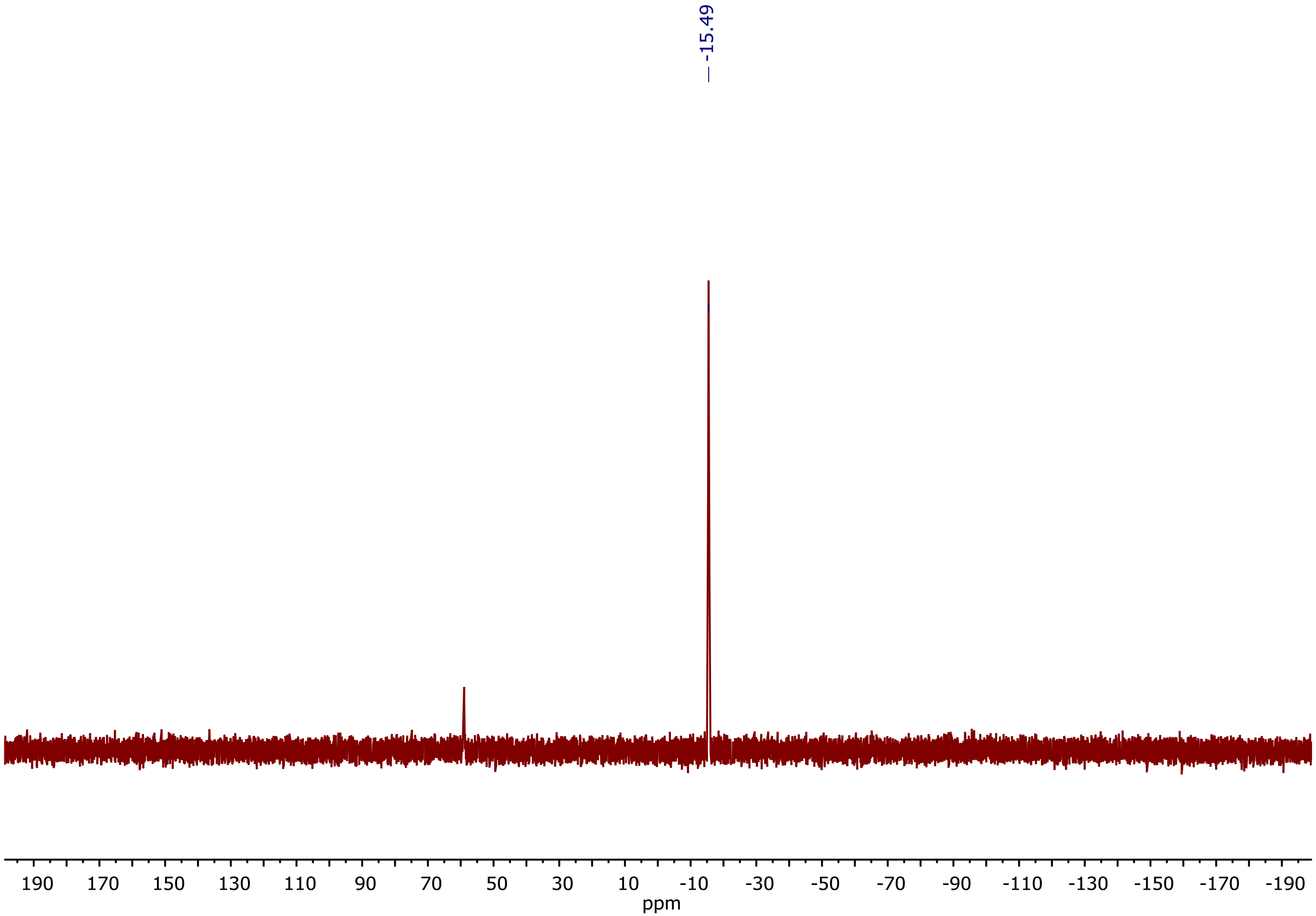

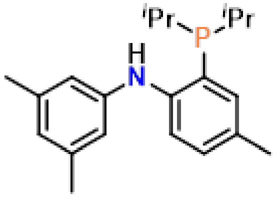

Figure S 71: ^{31}P NMR spectrum of ***HPN***$^{\mathbf{3,5Me}}$ *in C_6D_6 at 298 K. The signal at ca. 58 ppm corresponds to an unknown impurity (most likely arising from slow oxidation of the phosphine ligand in air over time.)*

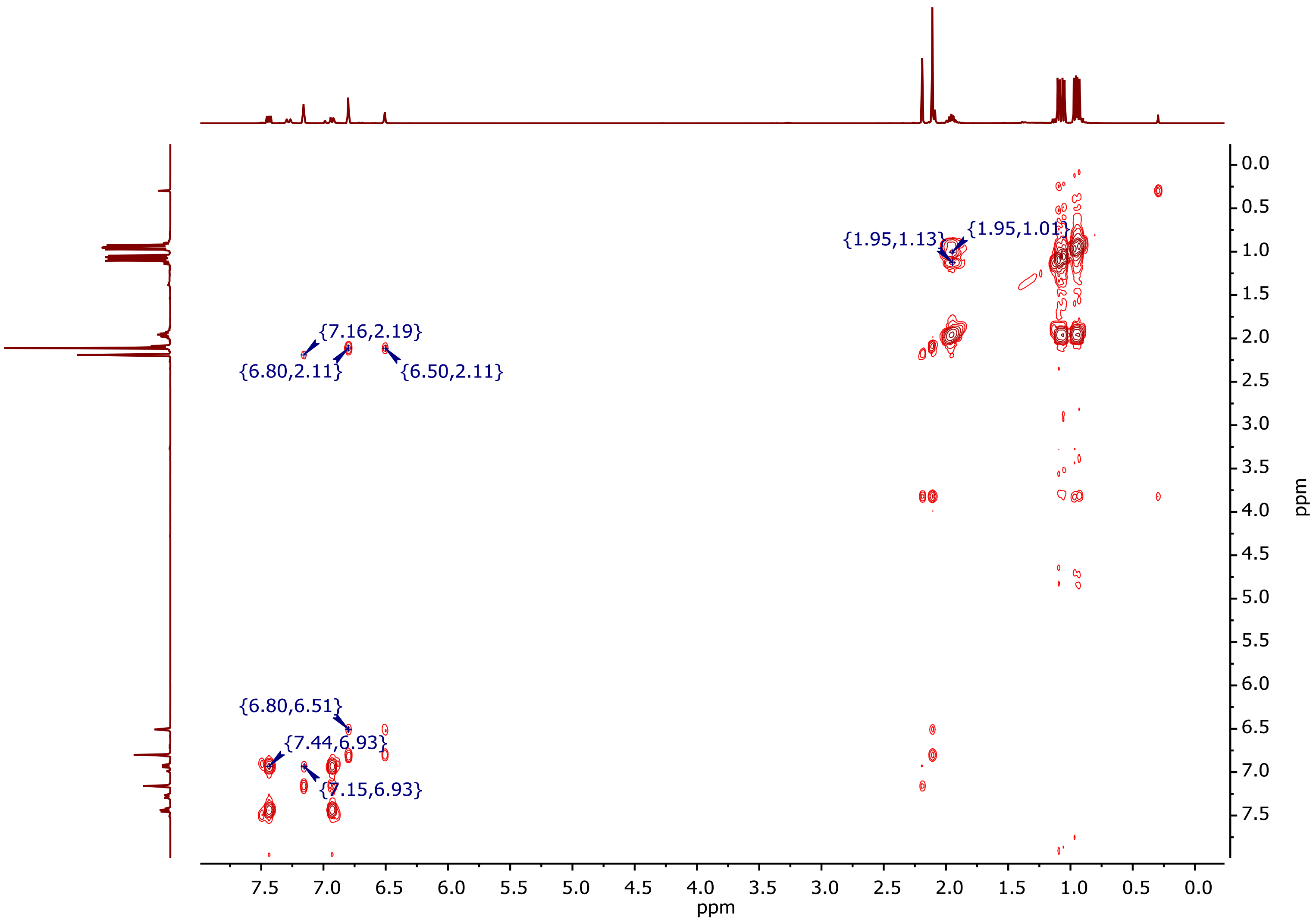

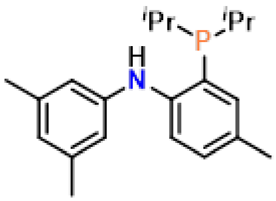

Figure S 72: ^1H-^1H COSY NMR spectrum of **HPN^{3,5Me}** in C_6D_6 at 298 K.

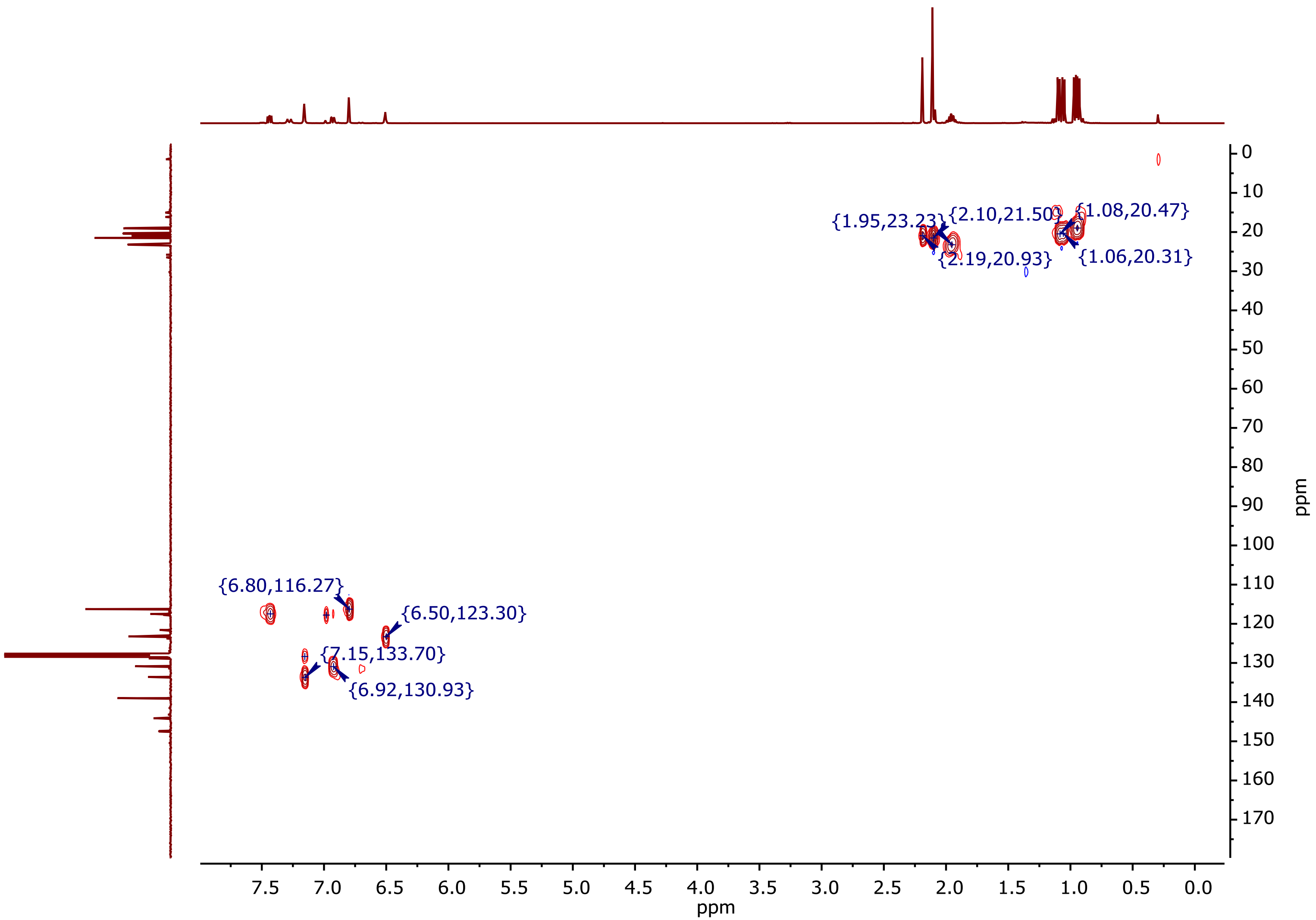

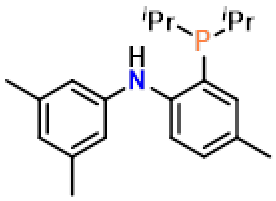

*Figure S 73: ^{1}H-^{13}C HSQC NMR spectrum of **HPN^{3,5Me}** in C_6D_6 at 298 K.*

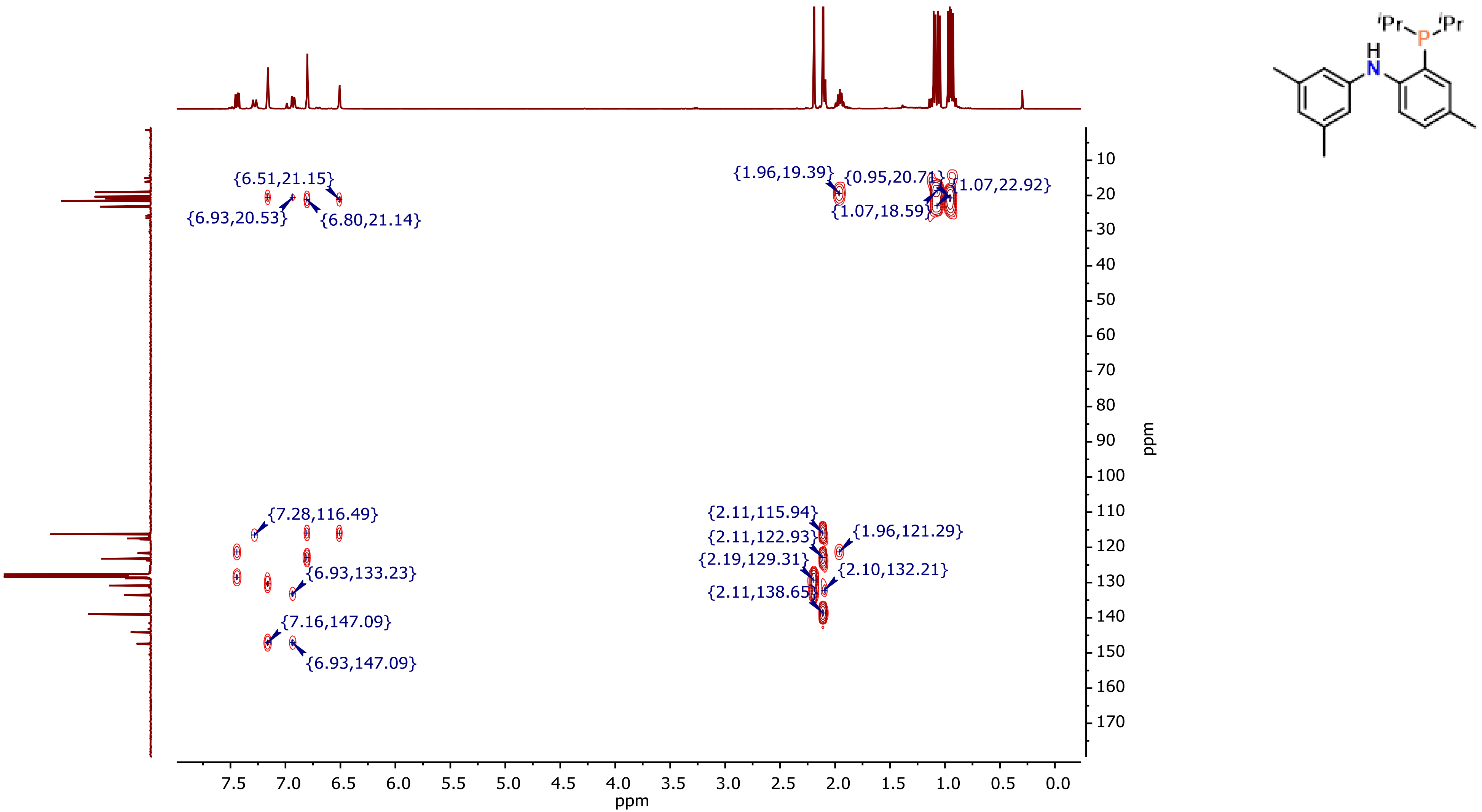

Figure S 74: ^{1}H-^{13}C HMBC NMR spectrum of **HPN^{3,5Me}** in C_6D_6 at 298 K.

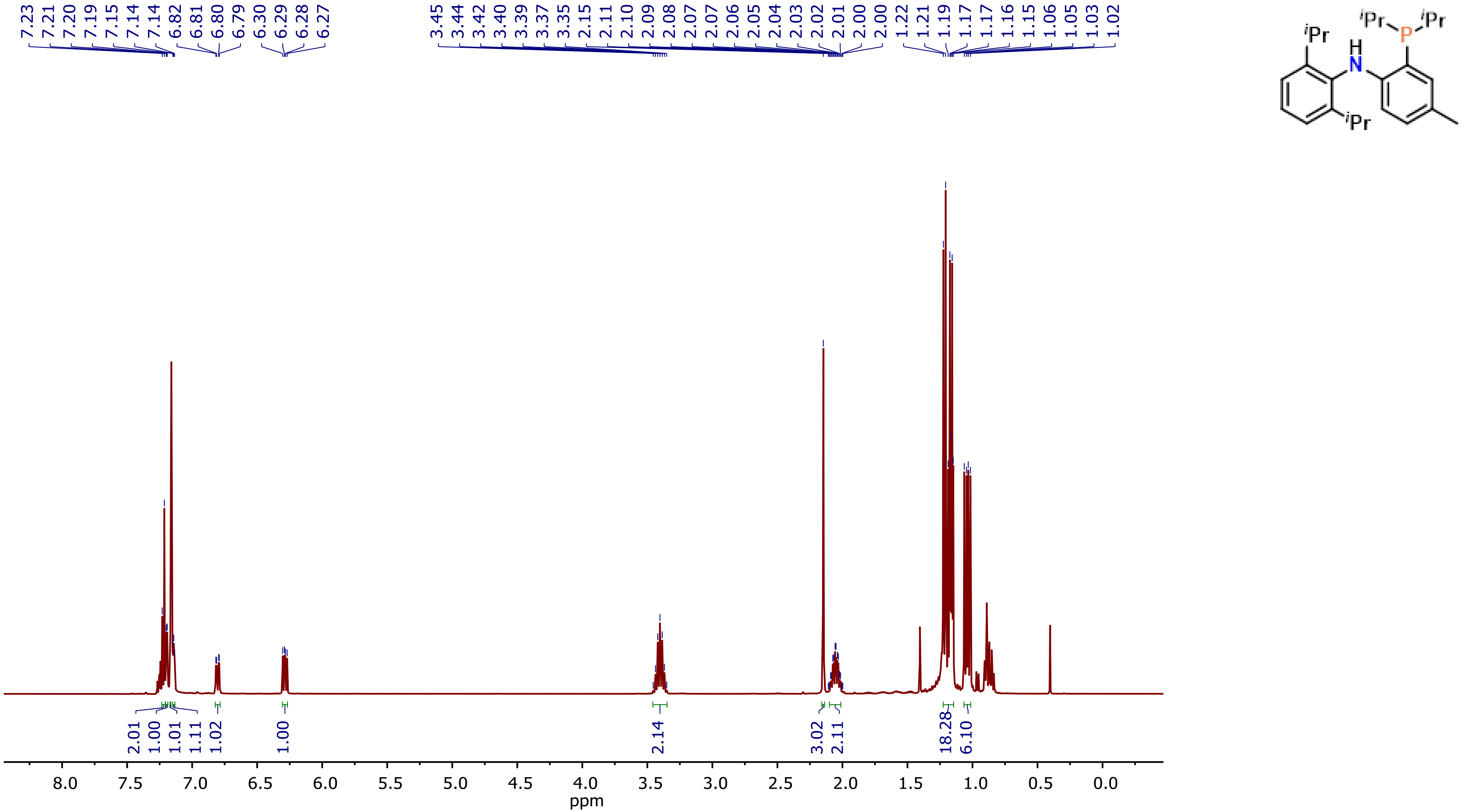

*Figure S 75: ^{1}H NMR spectrum of **HPN^{Dipp}** in C_6D_6 at 298 K.*

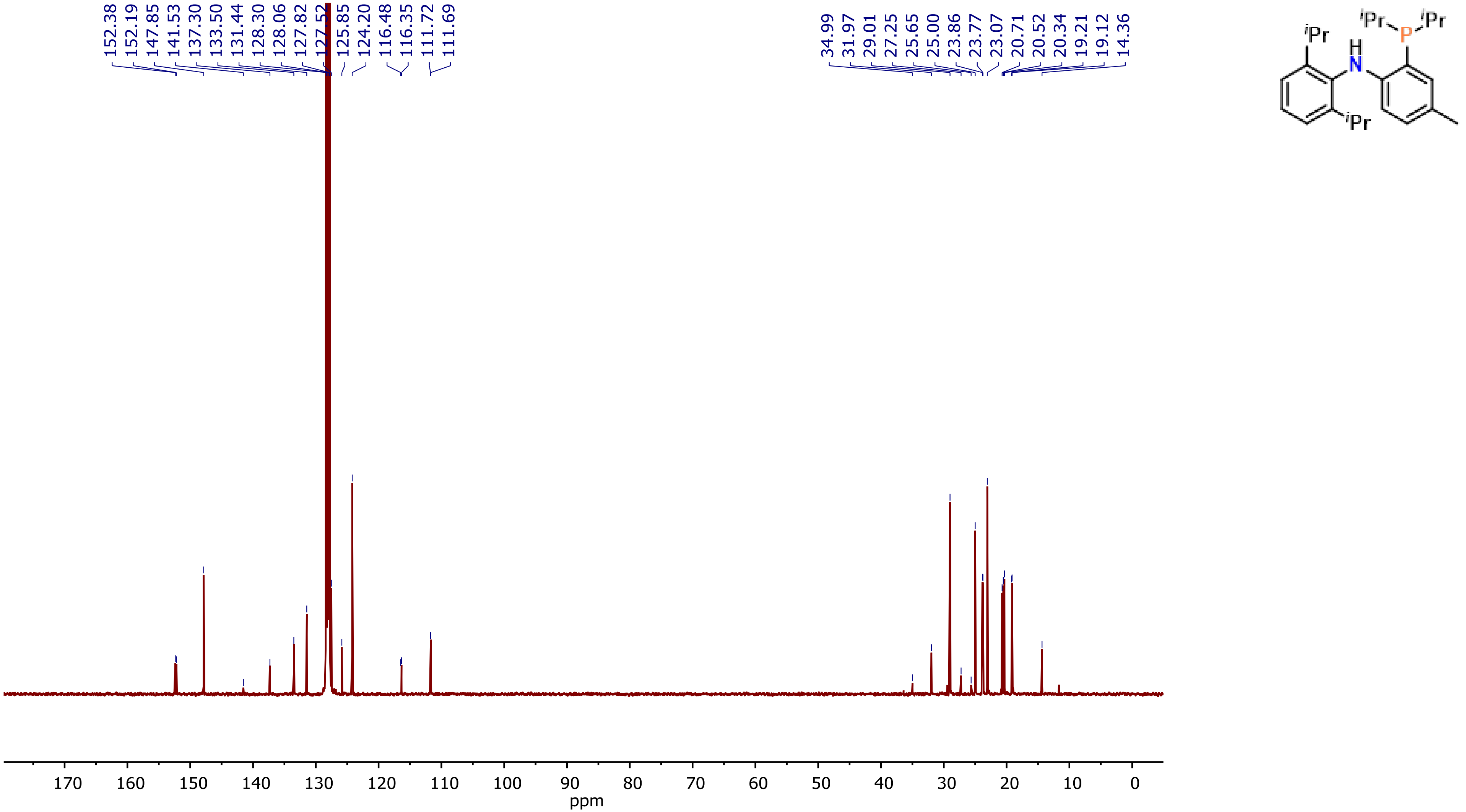

Figure S 76: $^{13}C\{^{1}H\}$ NMR spectrum of **HPN^{Dipp}** in C_6D_6 at 298 K.

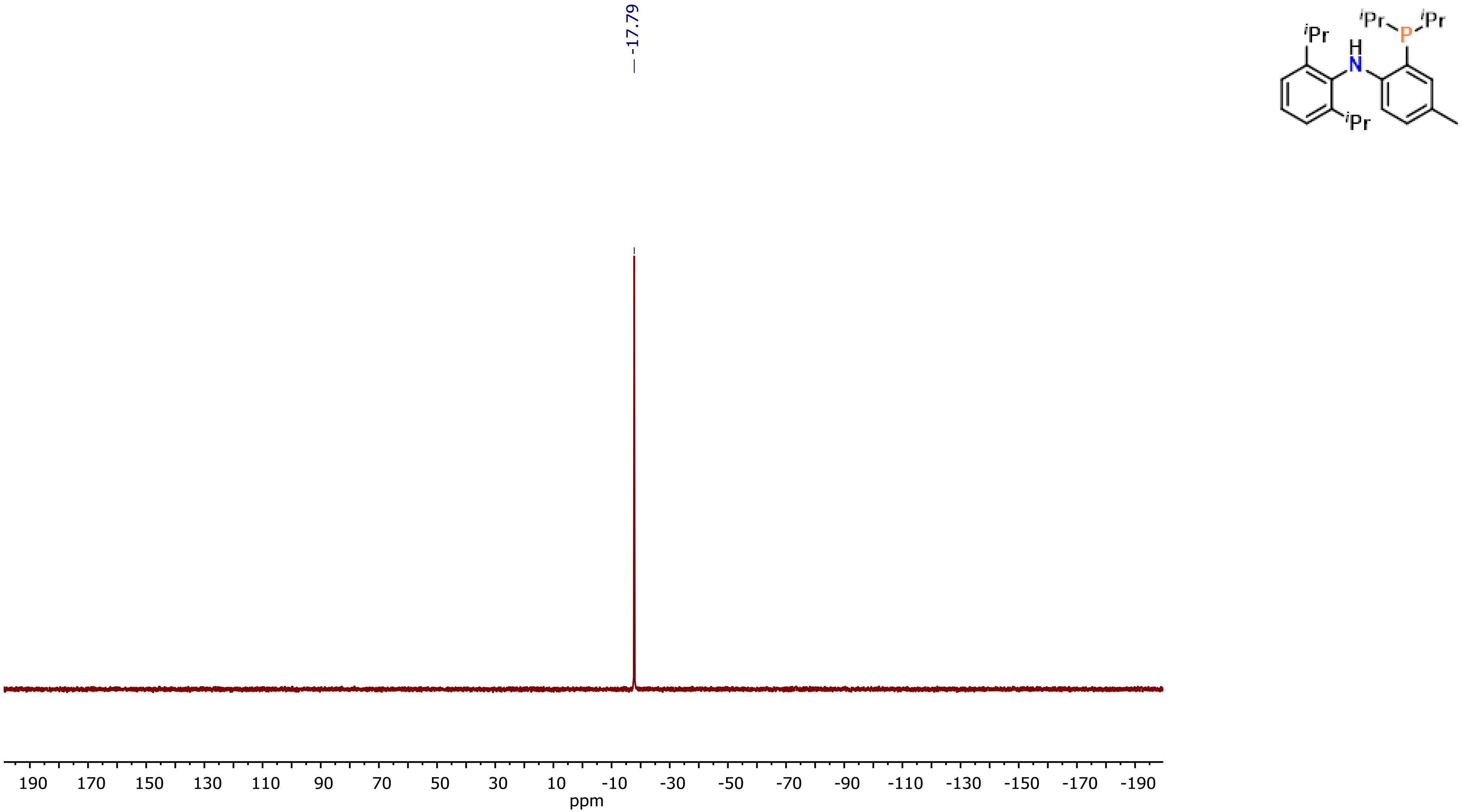

*Figure S 77: $^{31}P\{^{1}H\}$ NMR spectrum of **HPN^{Dipp}** in C_6D_6 at 298 K.*

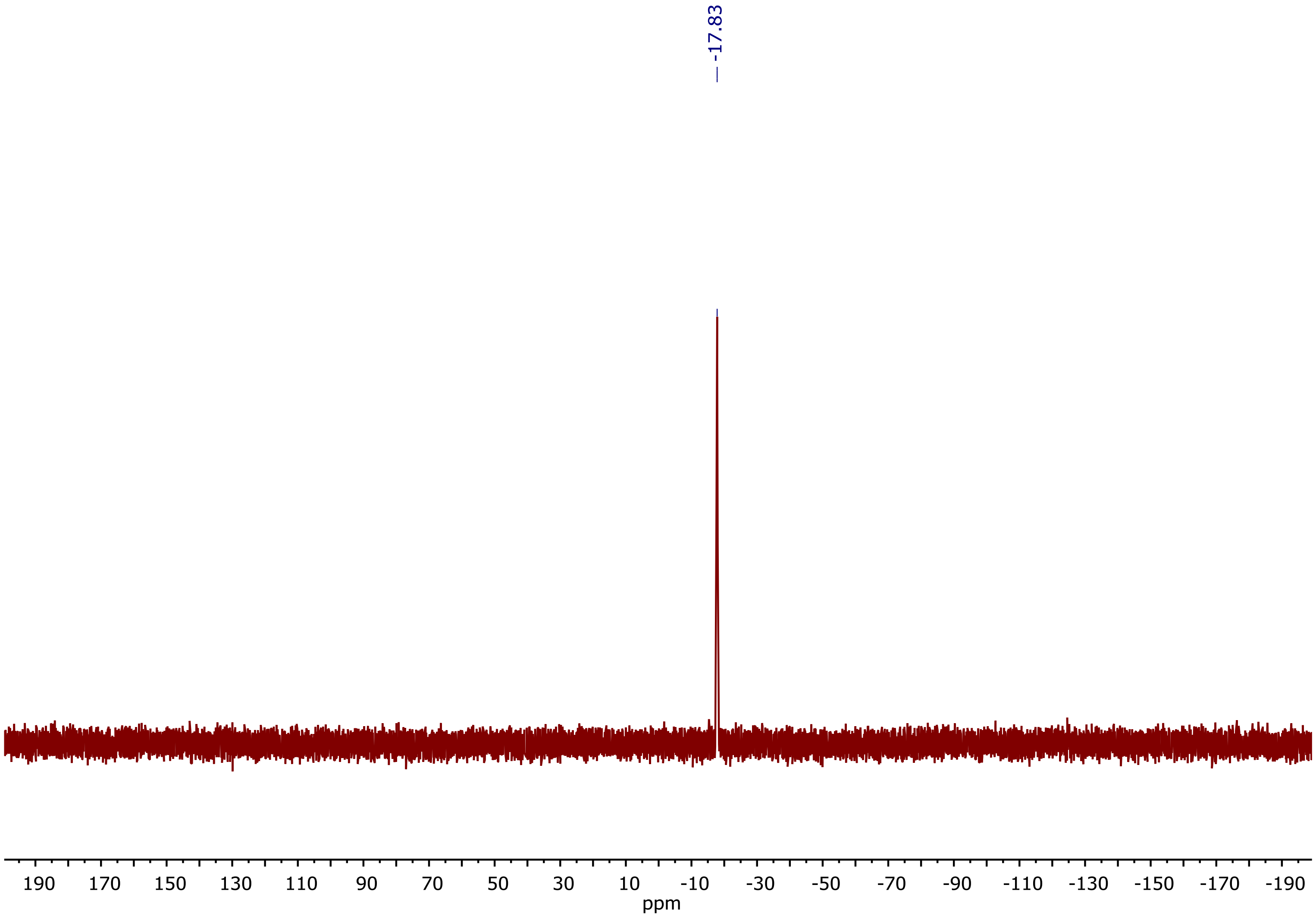

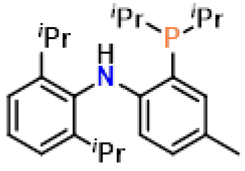

*Figure S 78: ^{31}P NMR spectrum of **HPN^{Dipp}** in C_6D_6 at 298 K.*

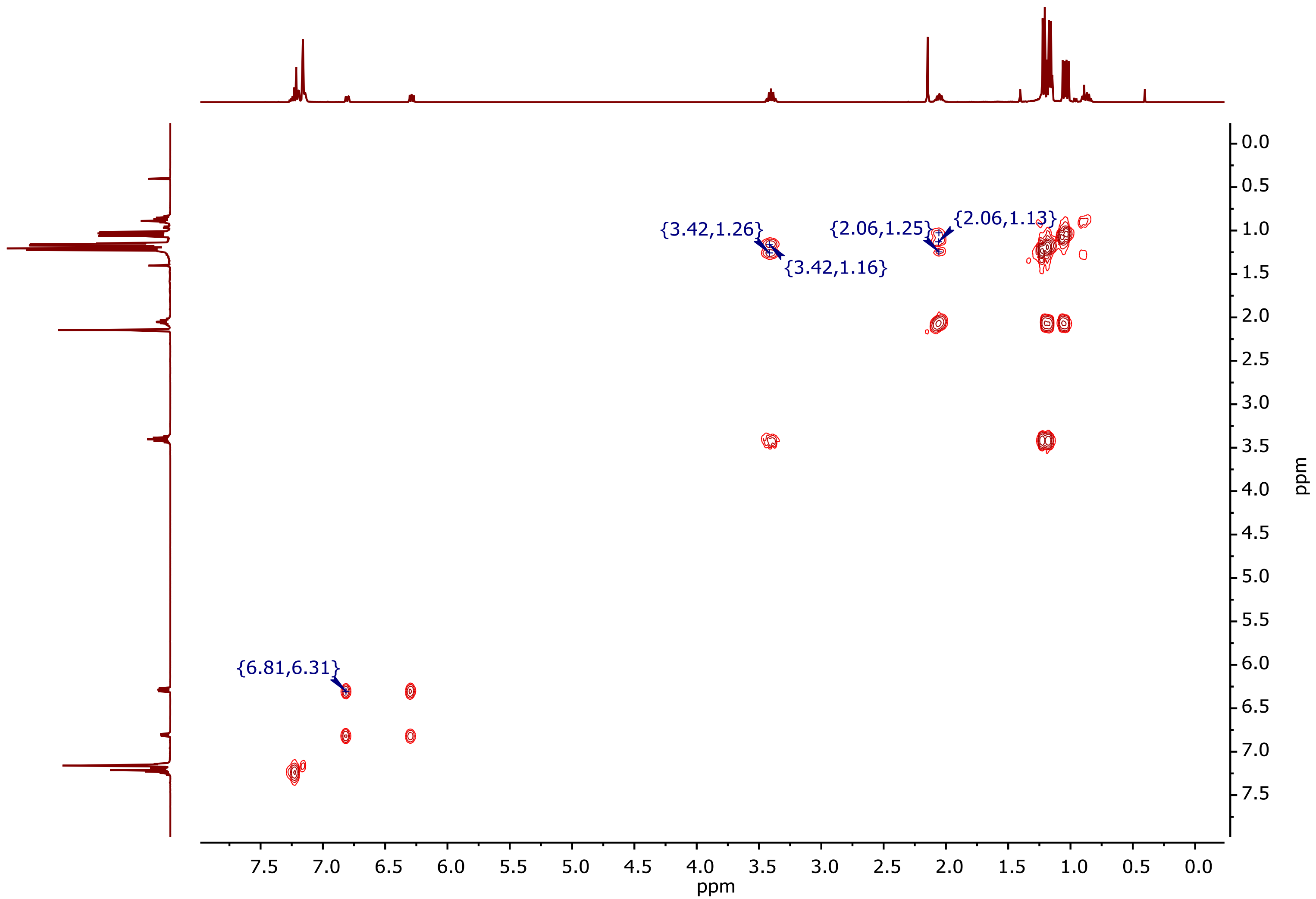

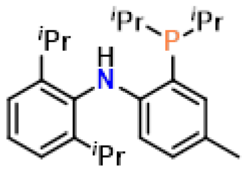

*Figure S 79: ^{1}H-^{1}H COSY NMR spectrum of **HPN^{Dipp}** in C_6D_6 at 298 K.*

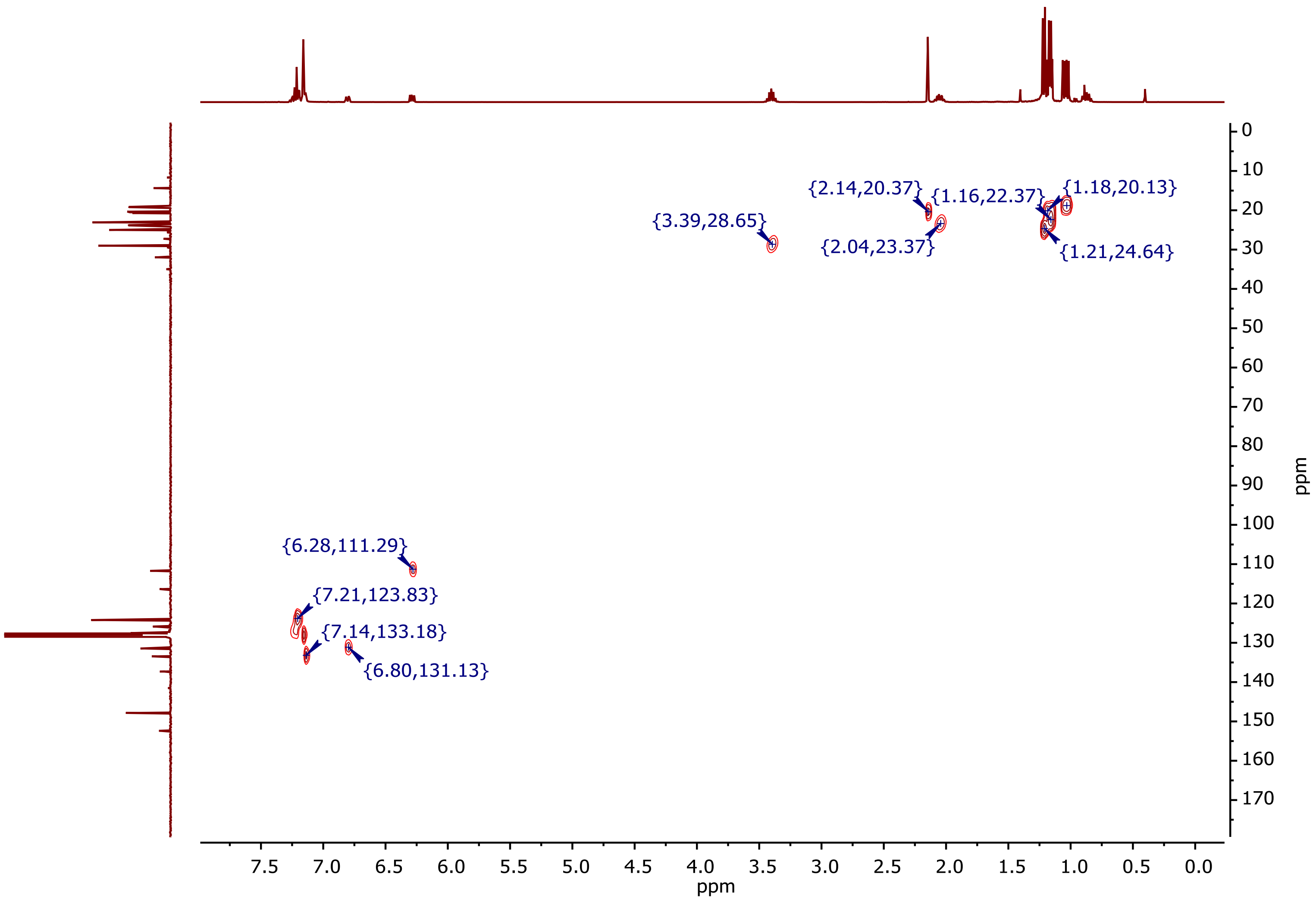

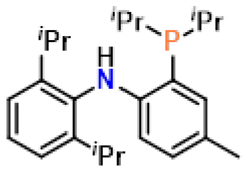

*Figure S 80: ^{1}H-^{13}C HSQC NMR spectrum of **HPN^{Dipp}** in C_6D_6 at 298 K.*

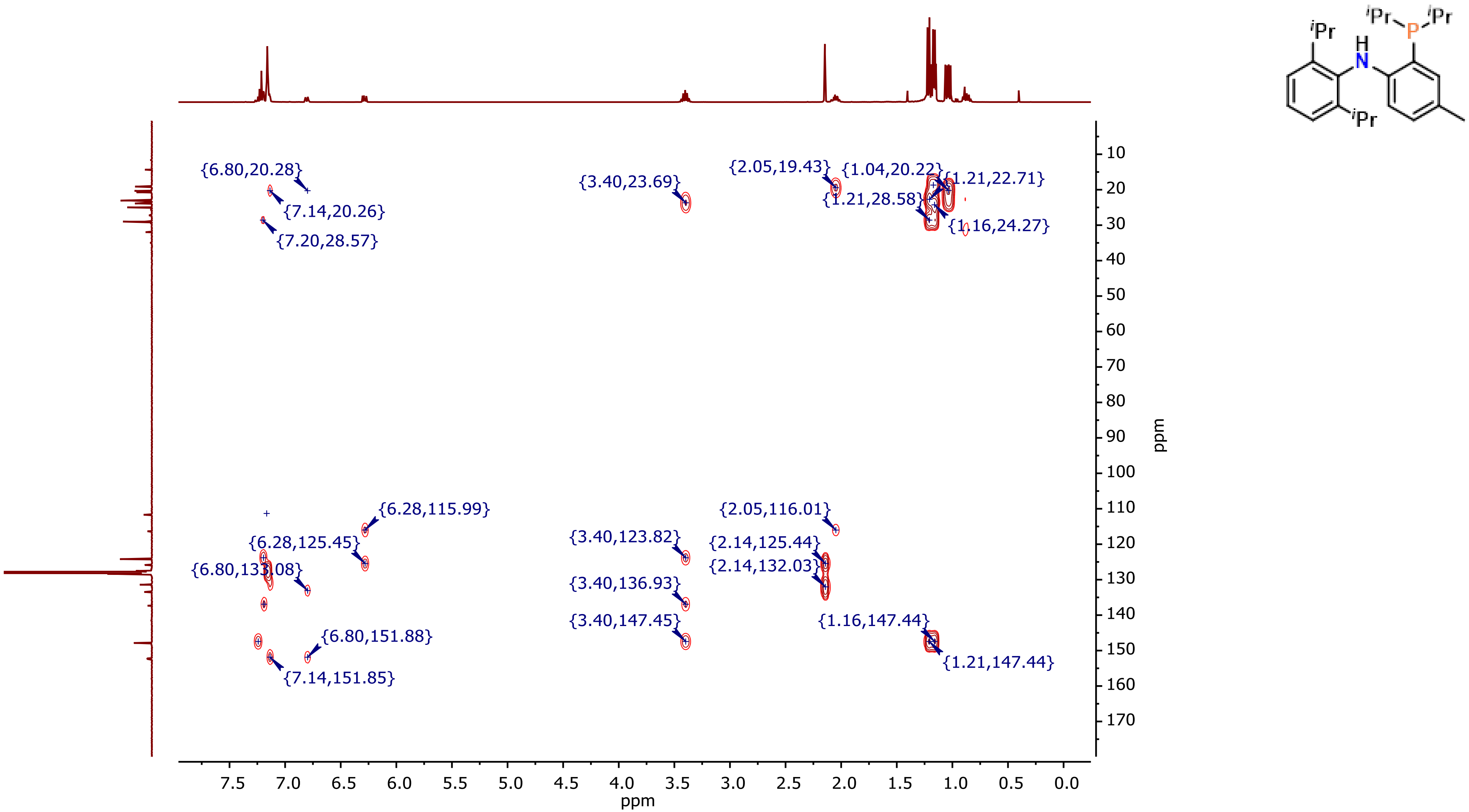

Figure S 81: ^{1}H-^{13}C HMBC NMR spectrum of **HPN^{Dipp}** in C_6D_6 at 298 K.

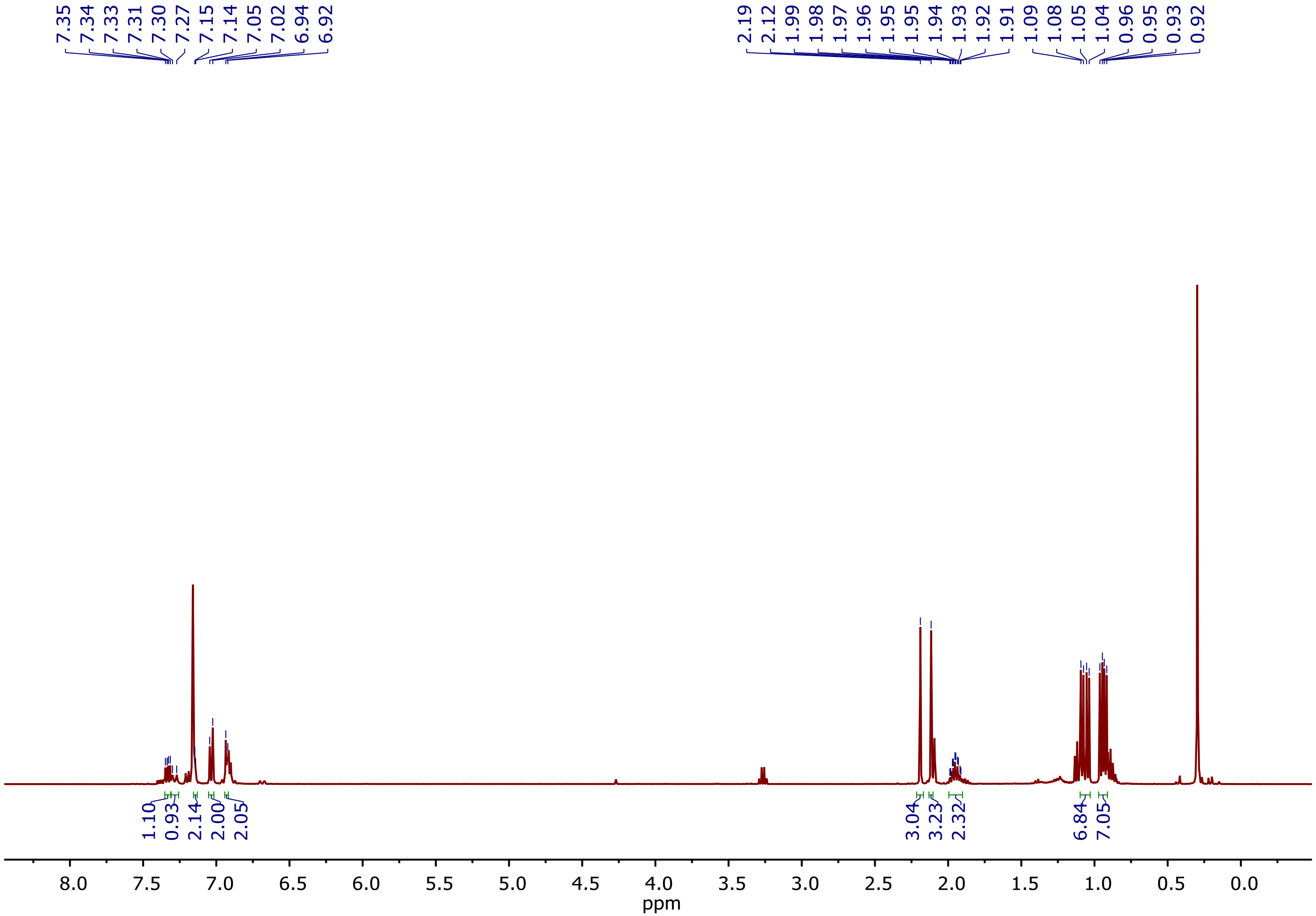

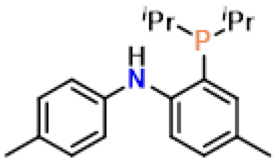

Figure S 82: ^{1}H NMR spectrum of ***HPN^{Tol}*** *in C_6D_6 at 298 K.*

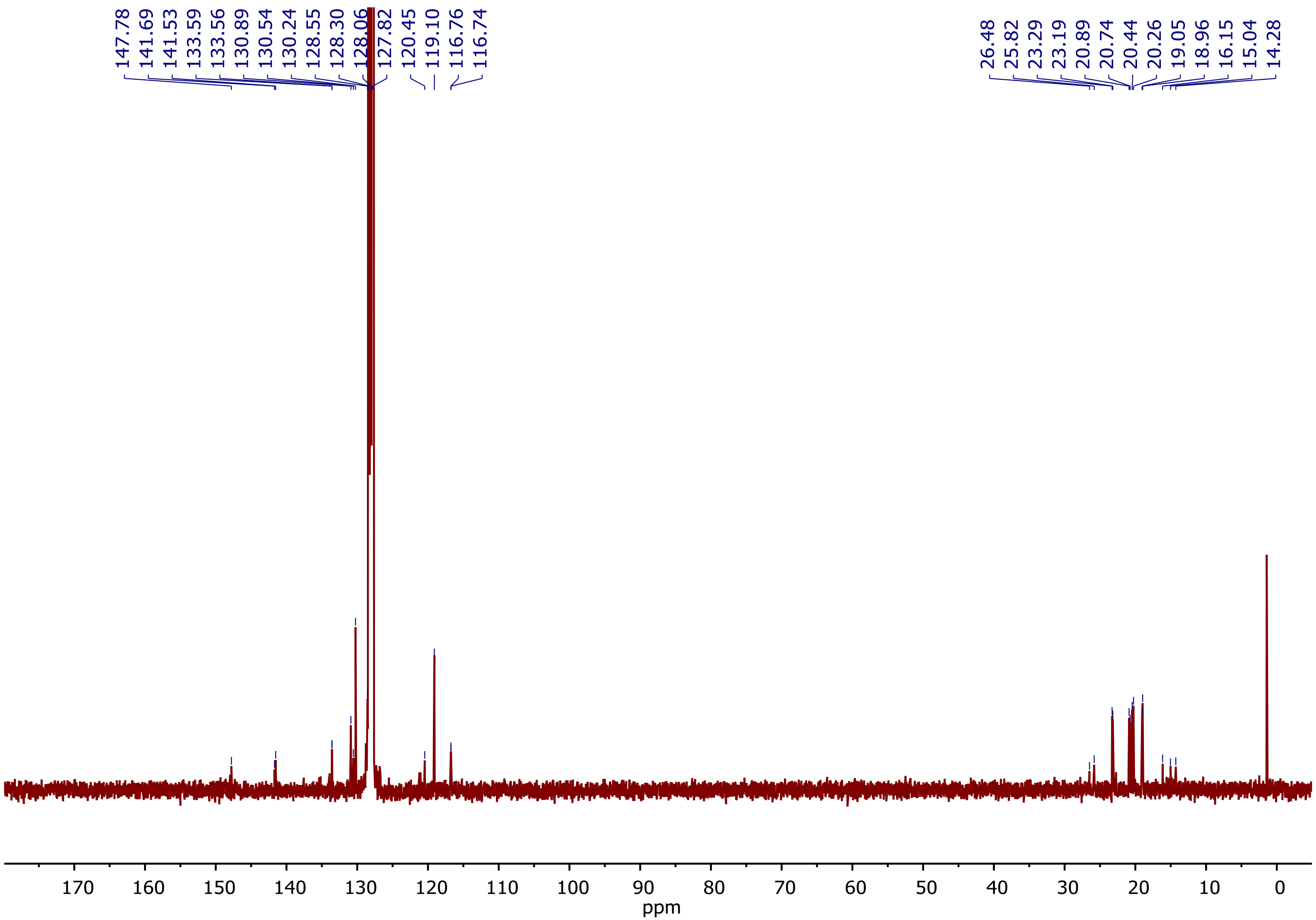

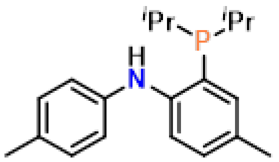

*Figure S 83: $^{13}C\{^{1}H\}$ NMR spectrum of **HPN^{Tol}** in C_6D_6 at 298 K.*

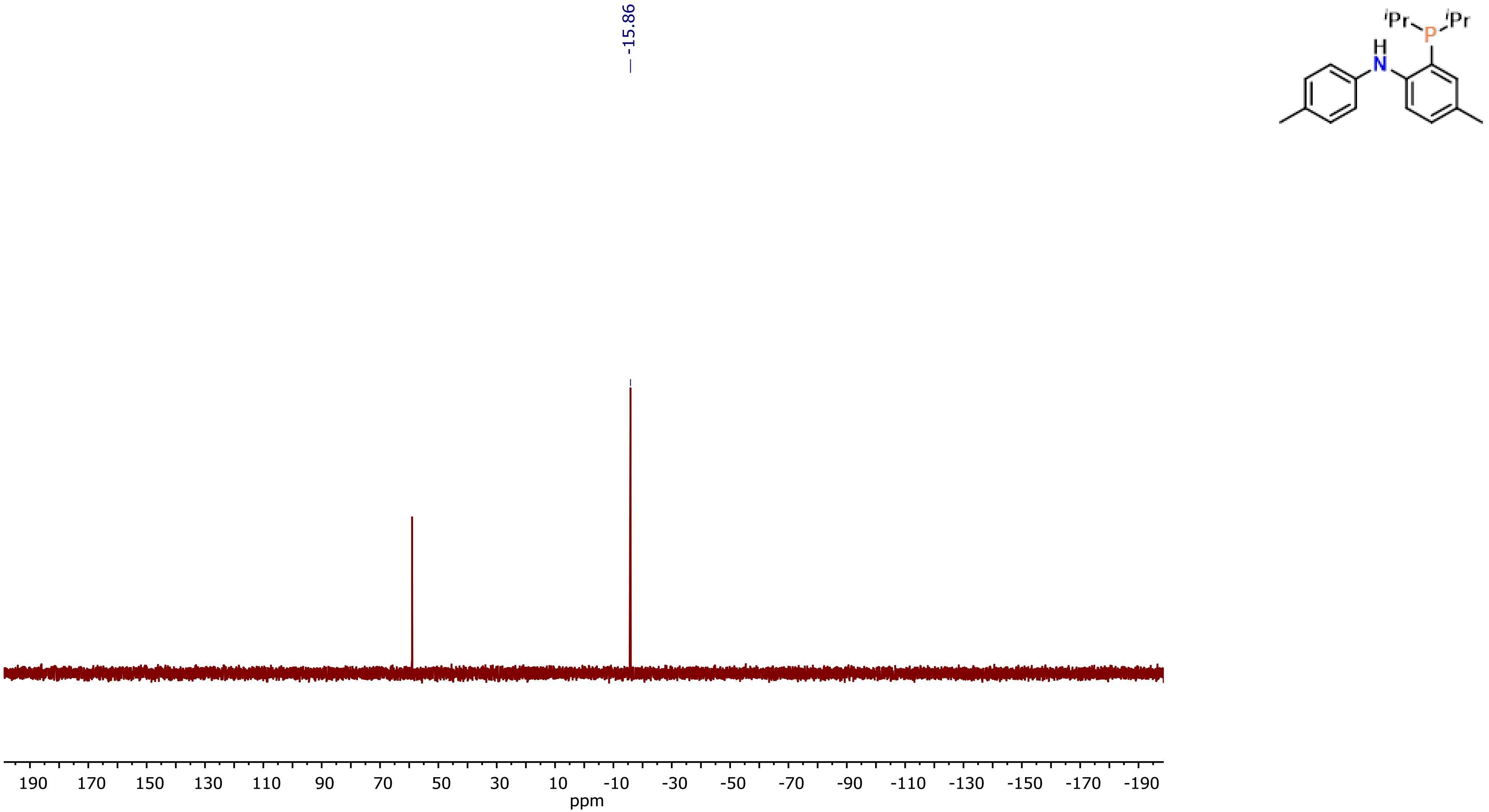

Figure S 84: $^{31}P\{^1H\}$ NMR spectrum of ***HPN^{Tol}*** *in C_6D_6 at 298 K. The signal at ca. 58 ppm corresponds to an unknown impurity (most likely arising from slow oxidation of the phosphine ligand in air over time.)*

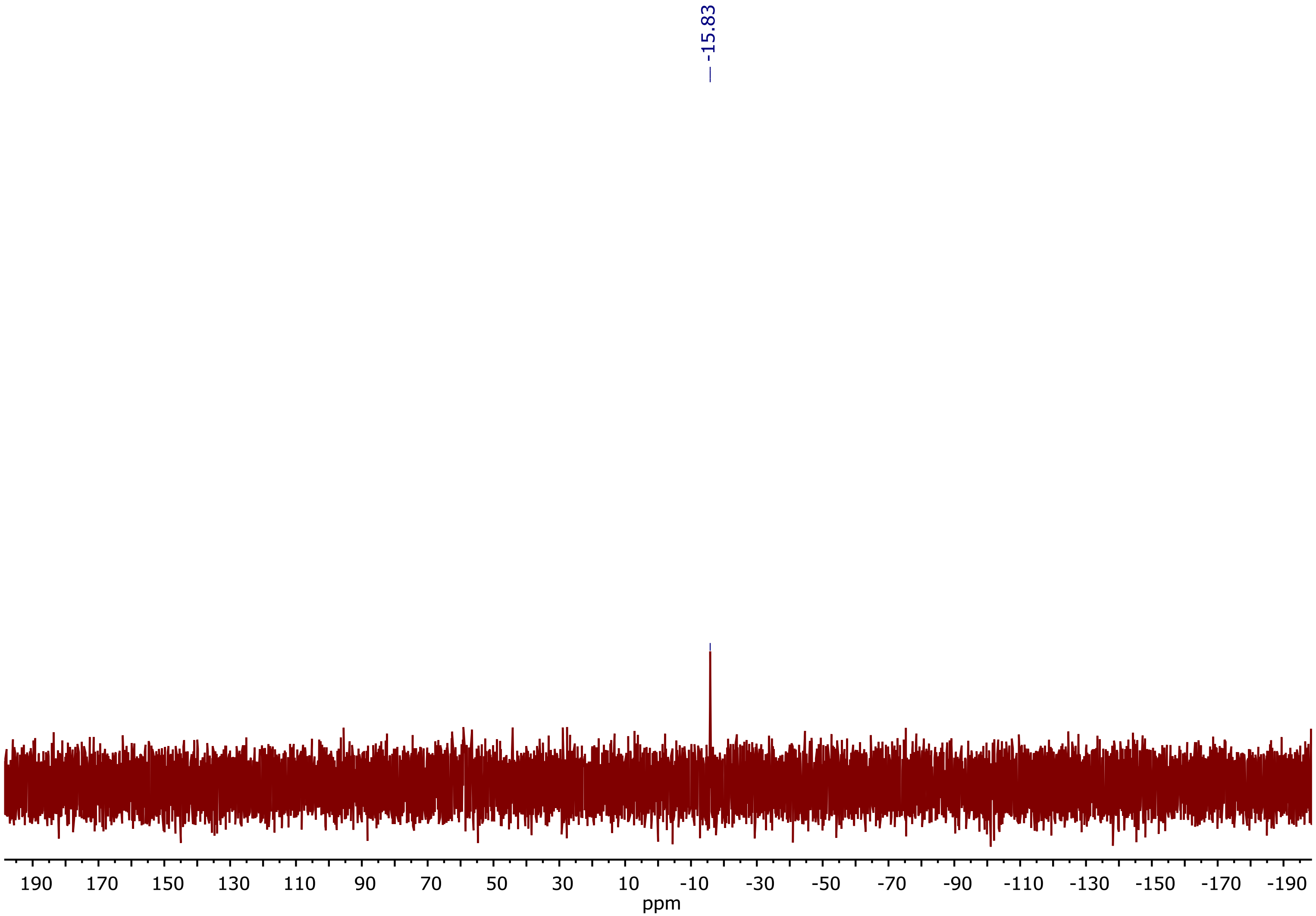

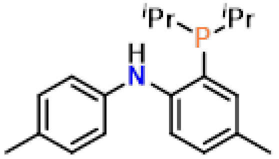

*Figure S 85: ^{31}P NMR spectrum of **HPN^{Tol}** in C_6D_6 at 298 K.*

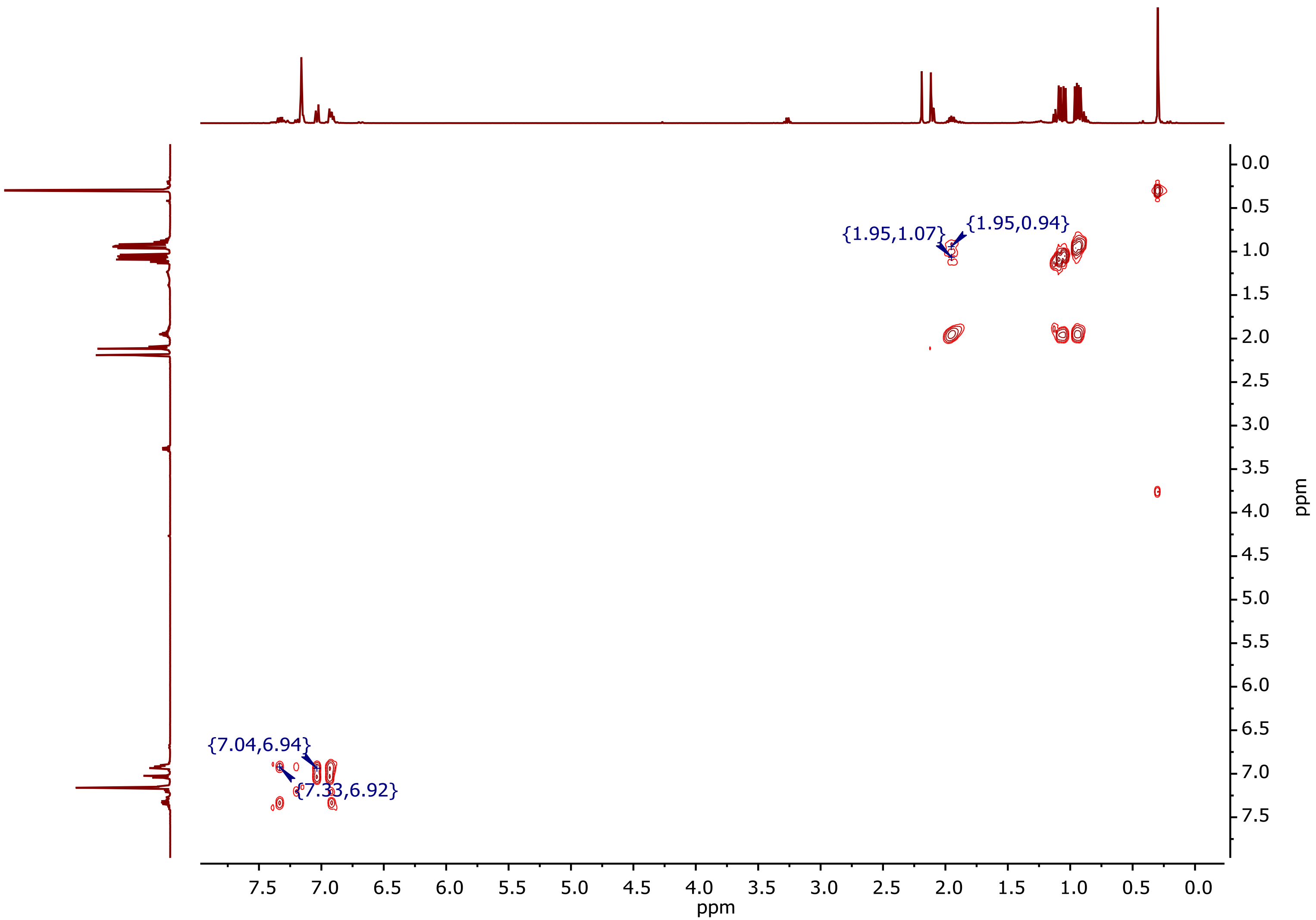

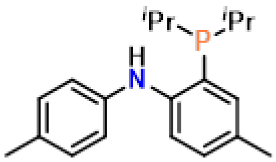

*Figure S 86: ^{1}H-^{1}H COSY NMR spectrum of **HPN^{Tol}** in C_6D_6 at 298 K.*

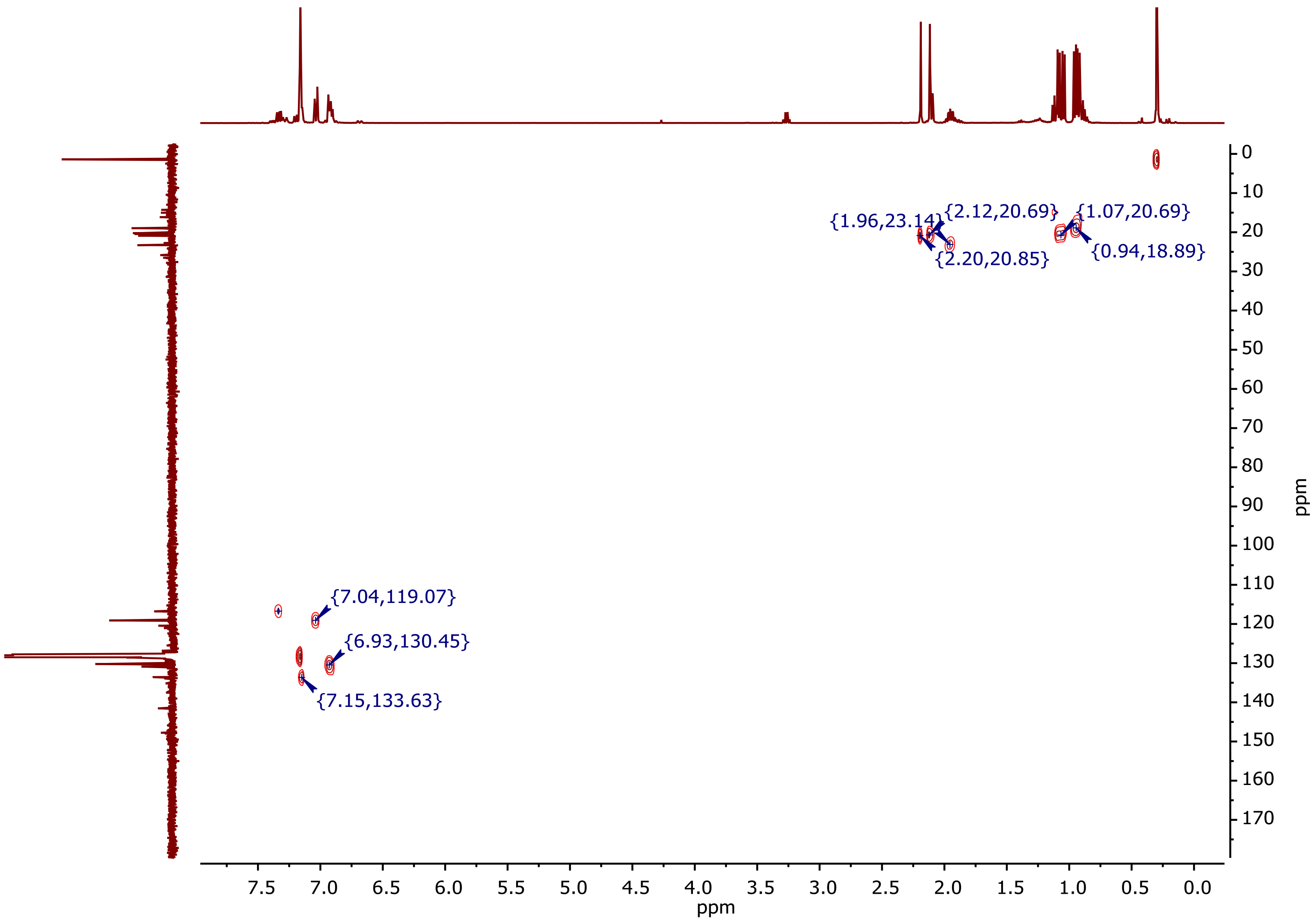

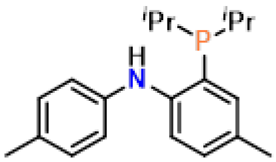

*Figure S 87: ^{1}H-^{13}C HSQC NMR spectrum of **HPN^{Tol}** in C_6D_6 at 298 K.*

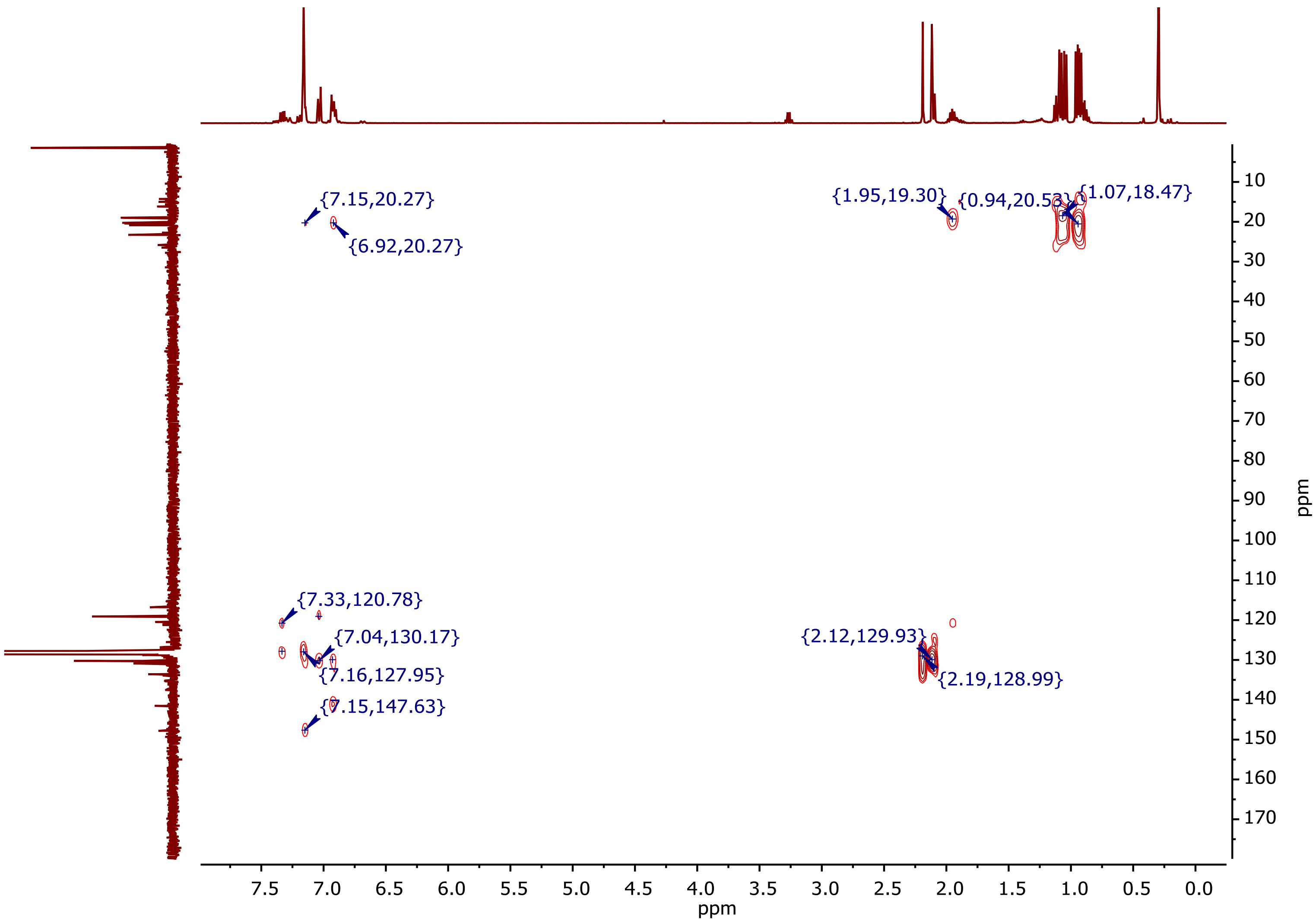

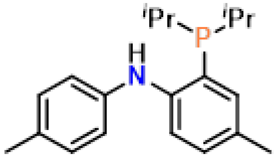

*Figure S 88: ^{1}H-^{13}C HMBC NMR spectrum of **HPN^{Tol}** in C_6D_6 at 298 K.*

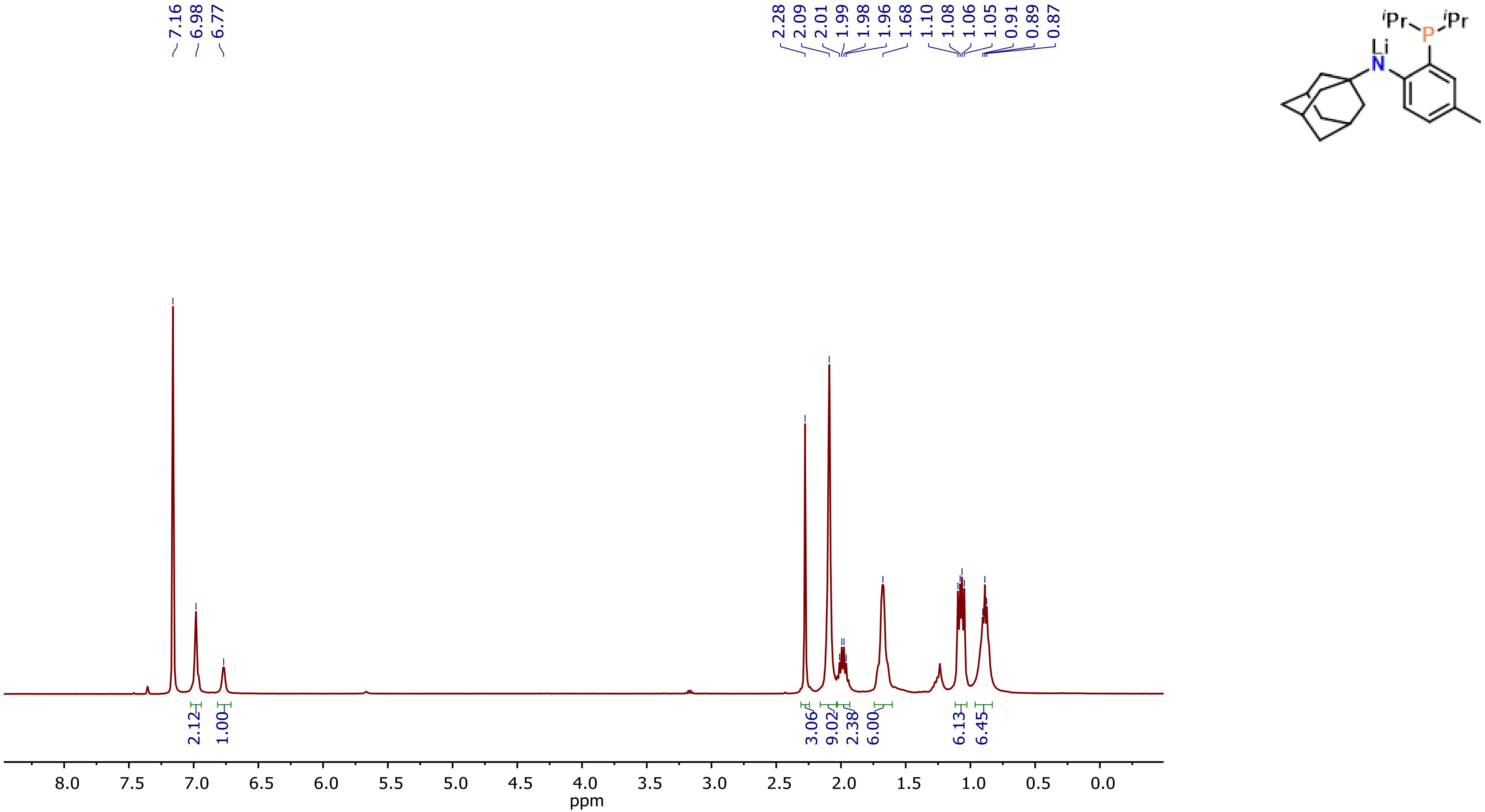

*Figure S 89: ^{1}H NMR spectrum of **LiPN^{Ad}** in C_6D_6 at 298 K.*

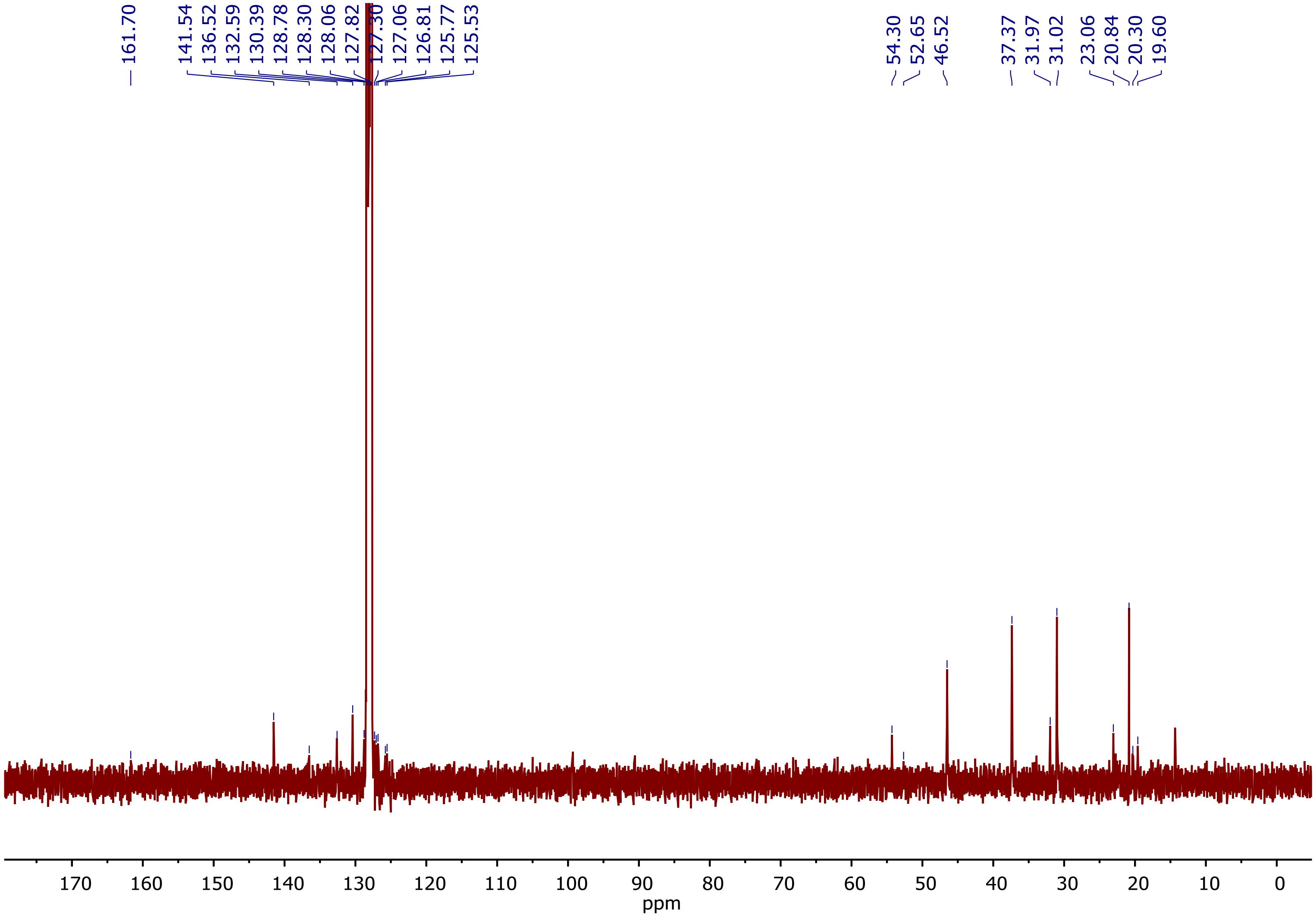

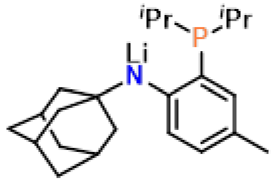

*Figure S 90: $^{13}C\{^1H\}$ NMR spectrum of **LiPN^{Ad}** in C_6D_6 at 298 K.*

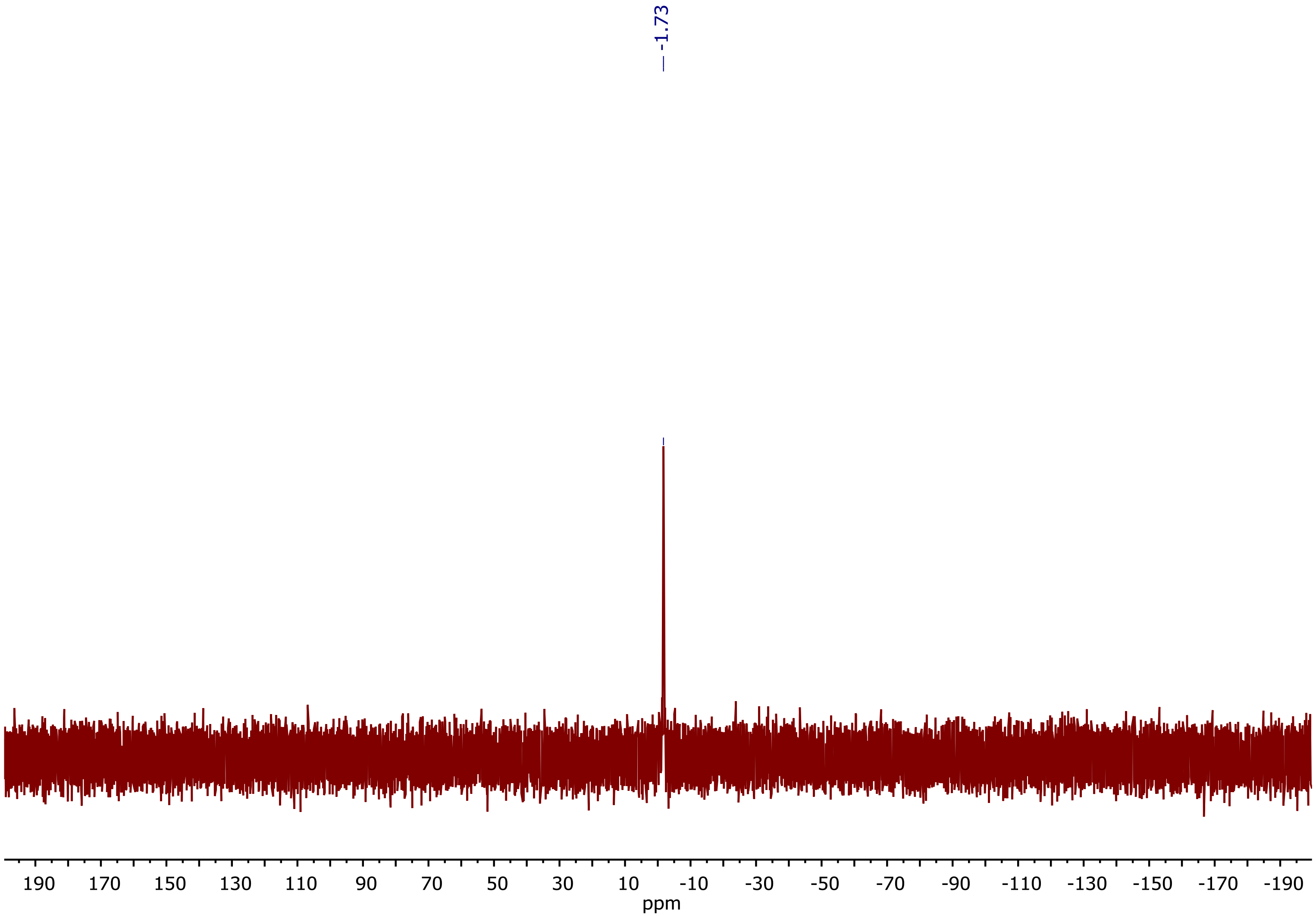

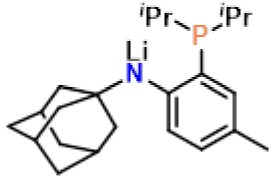

*Figure S 91: $^{31}P\{^1H\}$ NMR spectrum of **LiPN^{Ad}** in C_6D_6 at 298 K.*

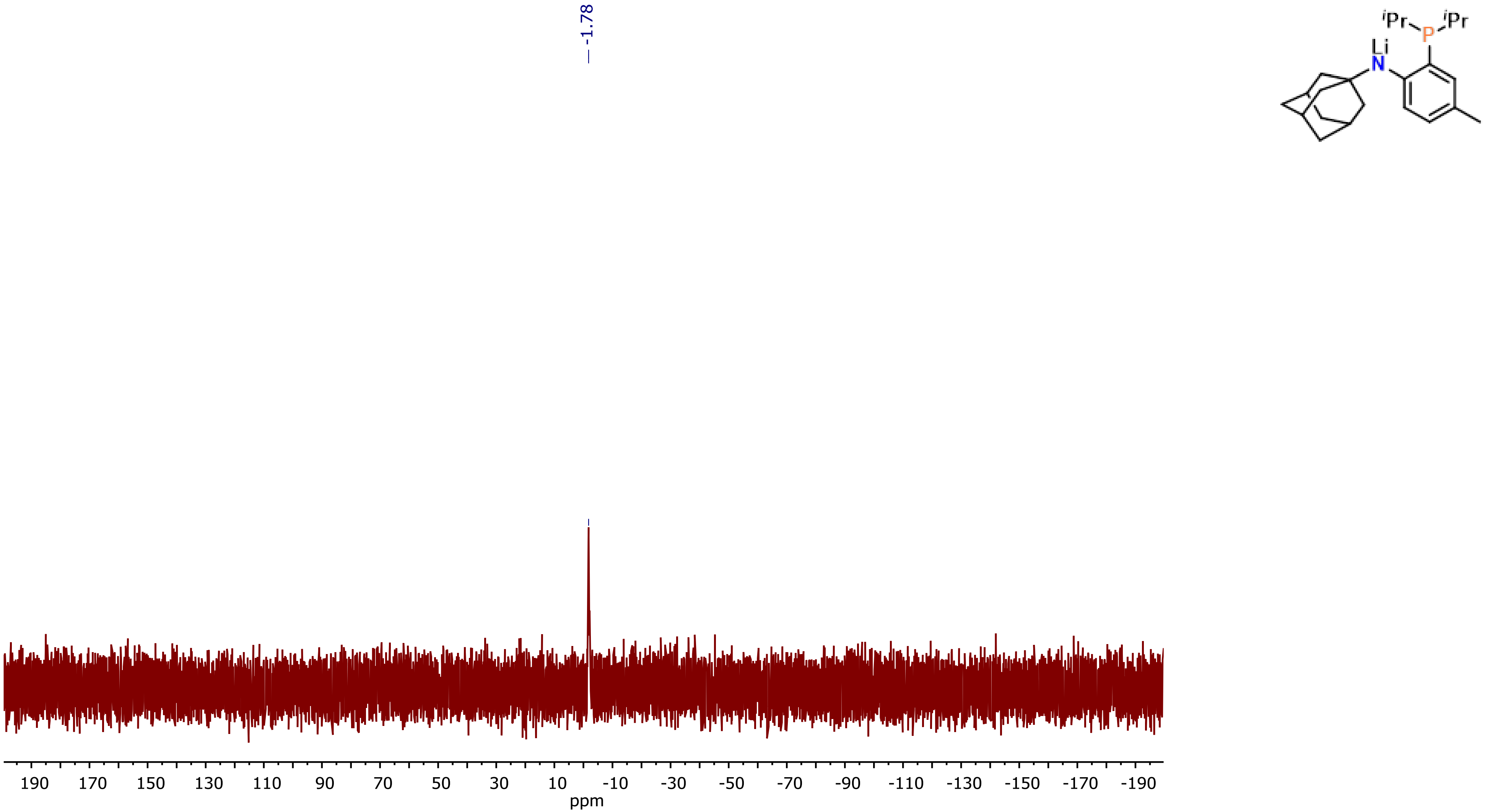

*Figure S 92: ^{31}P NMR spectrum of **LiPN^{Ad}** in C_6D_6 at 298 K.*

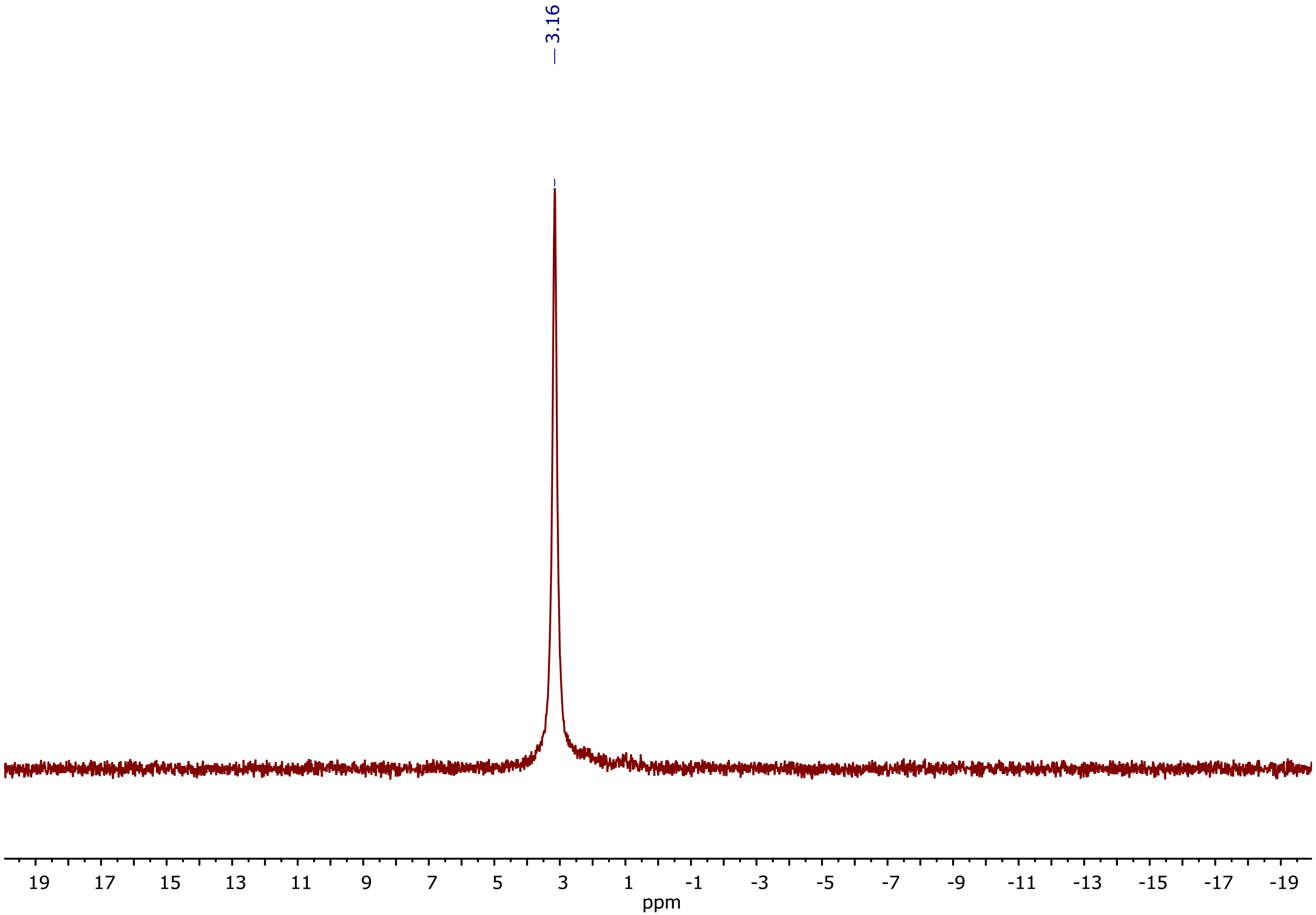

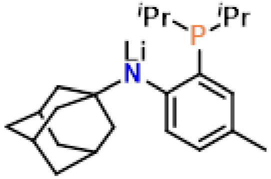

*Figure S 93: ^{7}Li{^{1}H} NMR spectrum of **LiPN^{Ad}** in C_6D_6 at 298 K.*

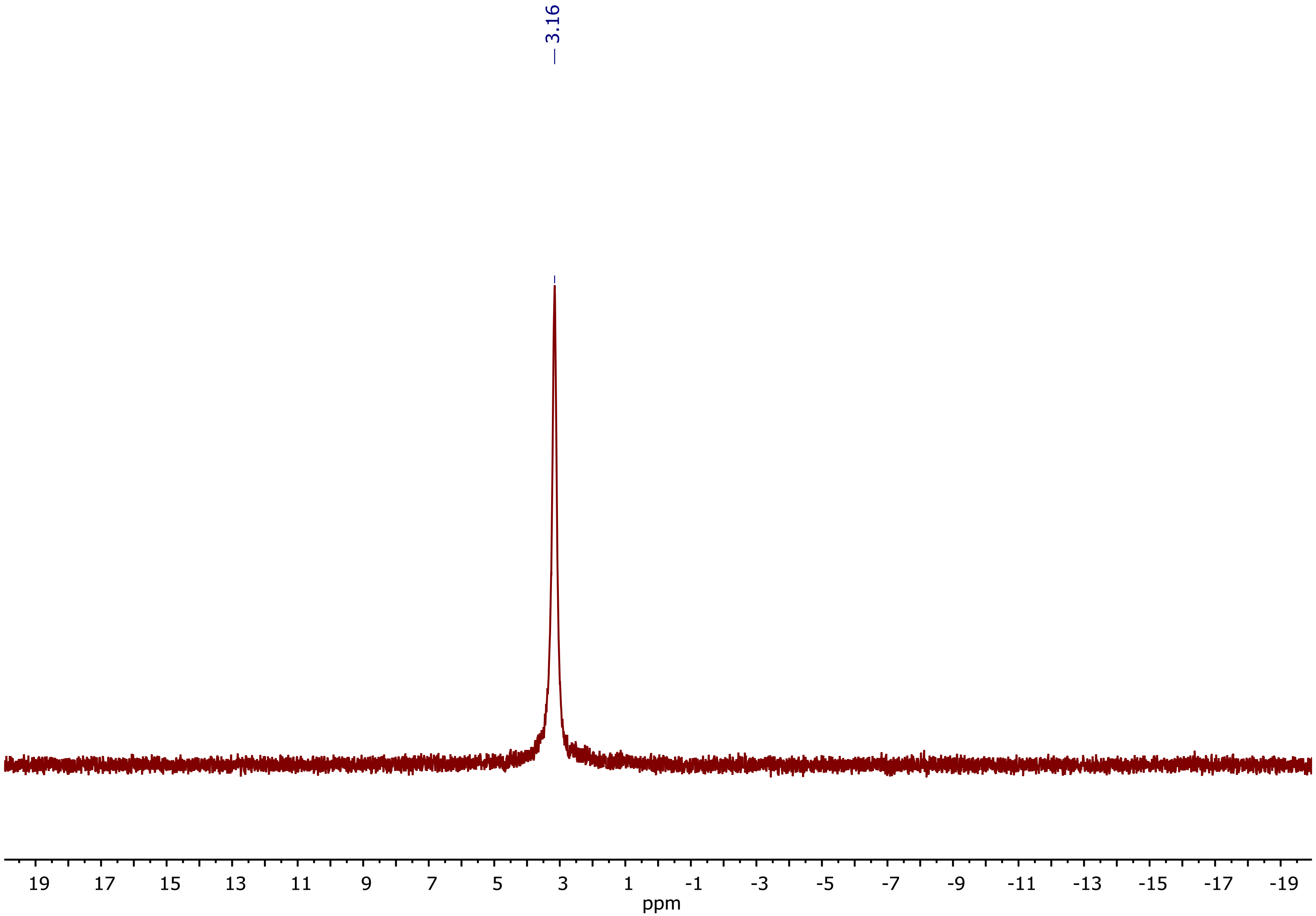

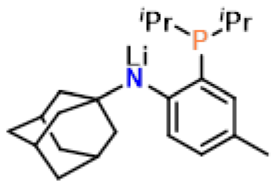

*Figure S 94: ^{7}Li NMR spectrum of **LiPN^{Ad}** in C_6D_6 at 298 K.*

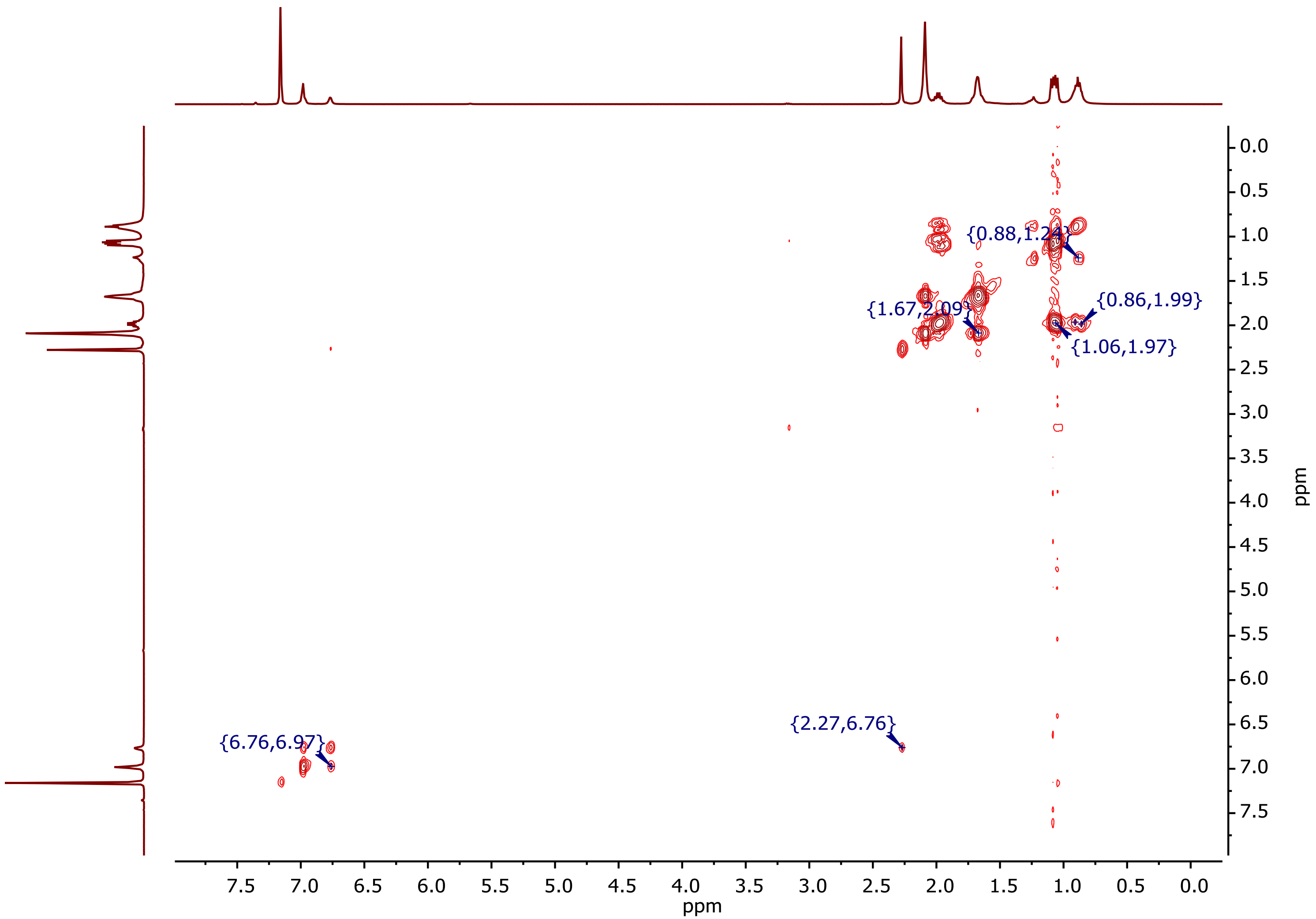

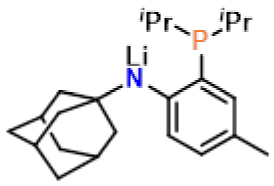

Figure S 95: ^{1}H-^{1}H COSY NMR spectrum of ***LiPN^{Ad}*** in C_6D_6 at 298 K.

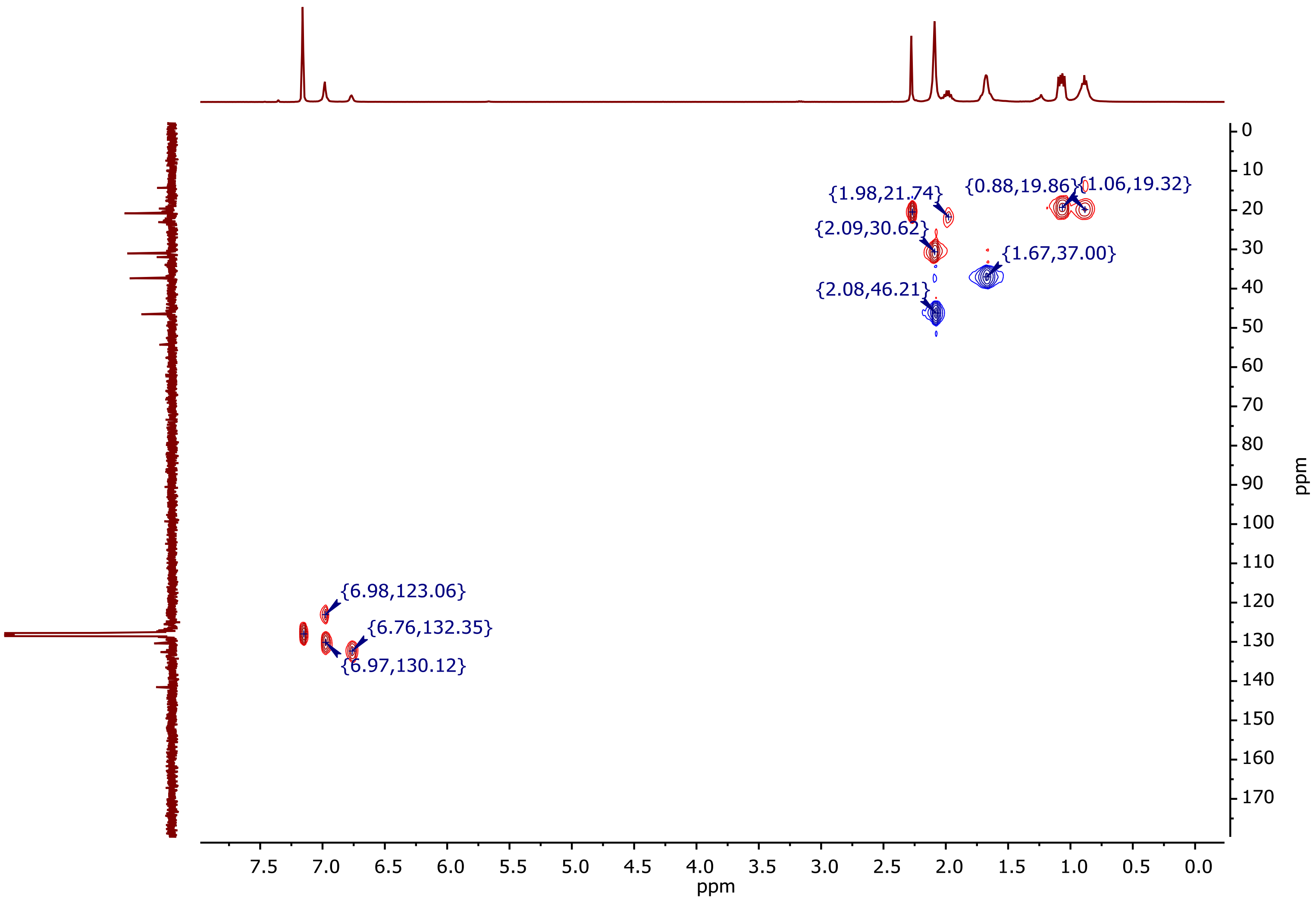

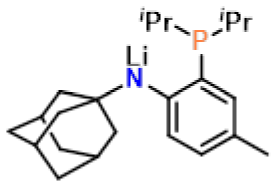

*Figure S 96: ^{1}H-^{13}C HSQC NMR spectrum of **LiPN^{Ad}** in C_6D_6 at 298 K.*

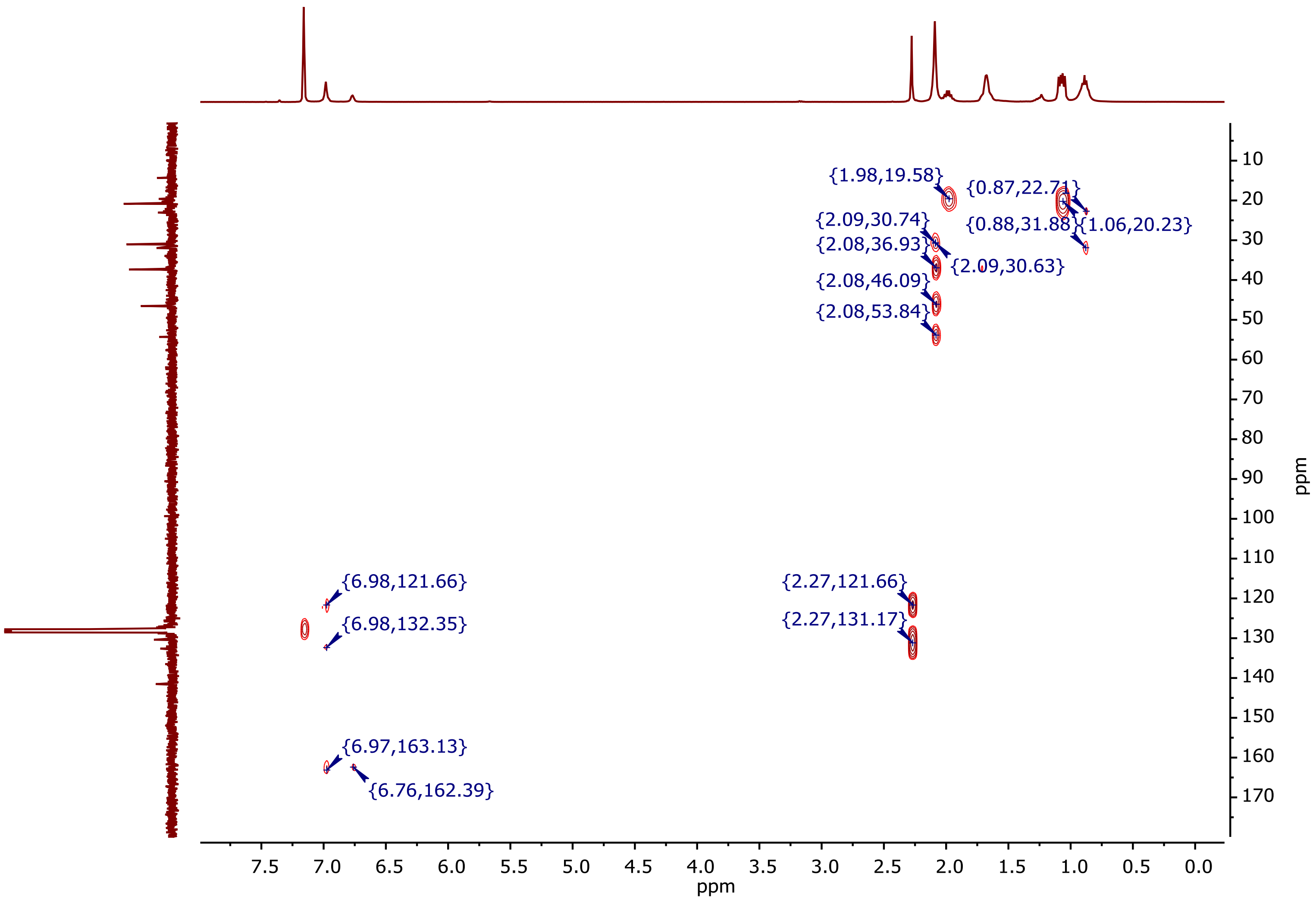

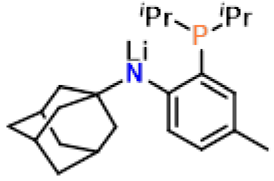

Figure S 97: ^{1}H-^{13}C HMBC NMR spectrum of ***LiPN^Ad*** in C_6D_6 at 298 K.

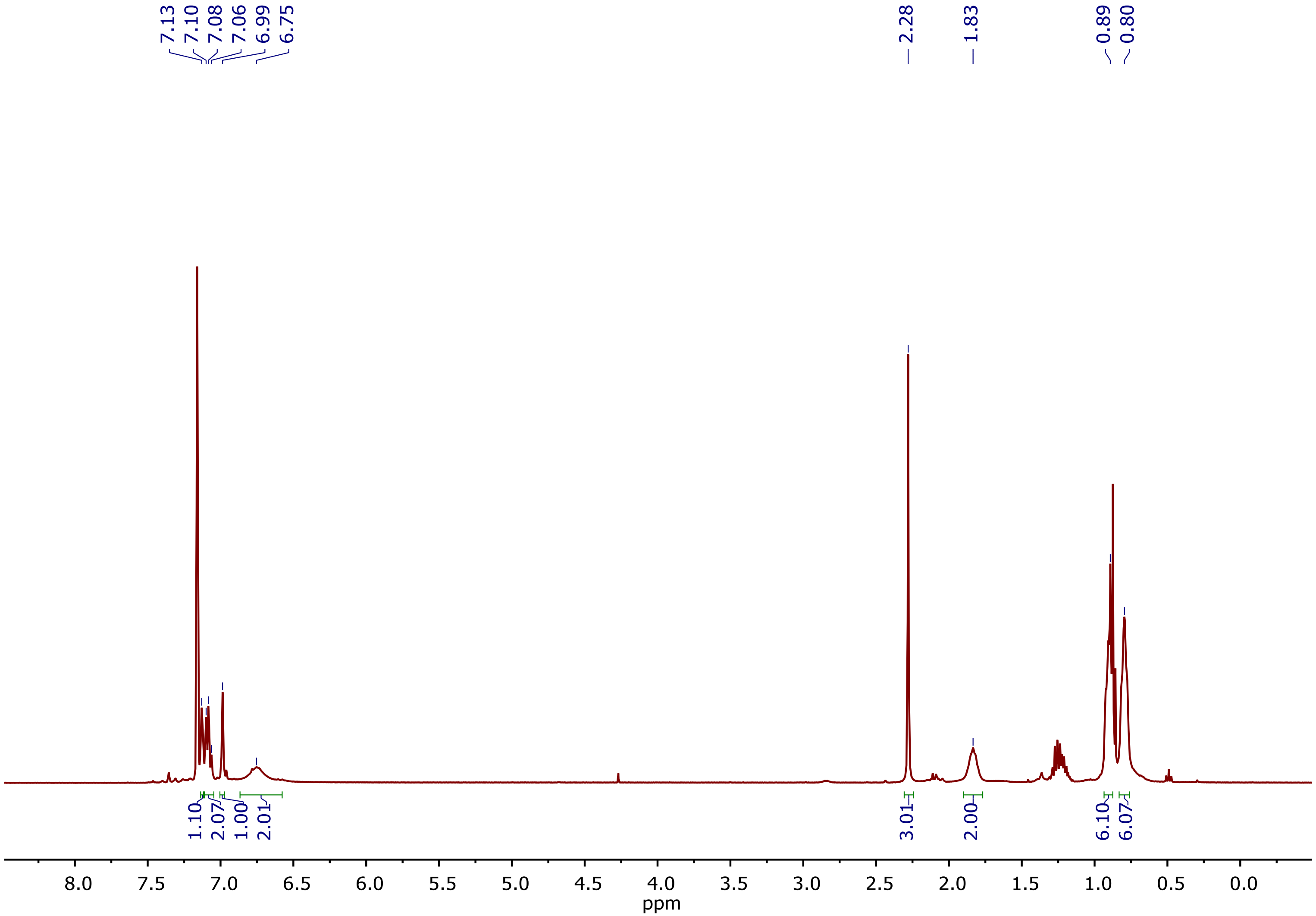

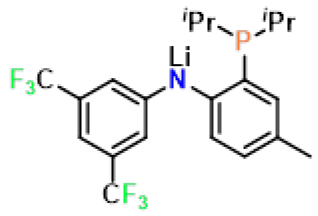

*Figure S 98: ^{1}H NMR spectrum of **LiPN^{3,5CF3}** in C_6D_6 at 298 K.*

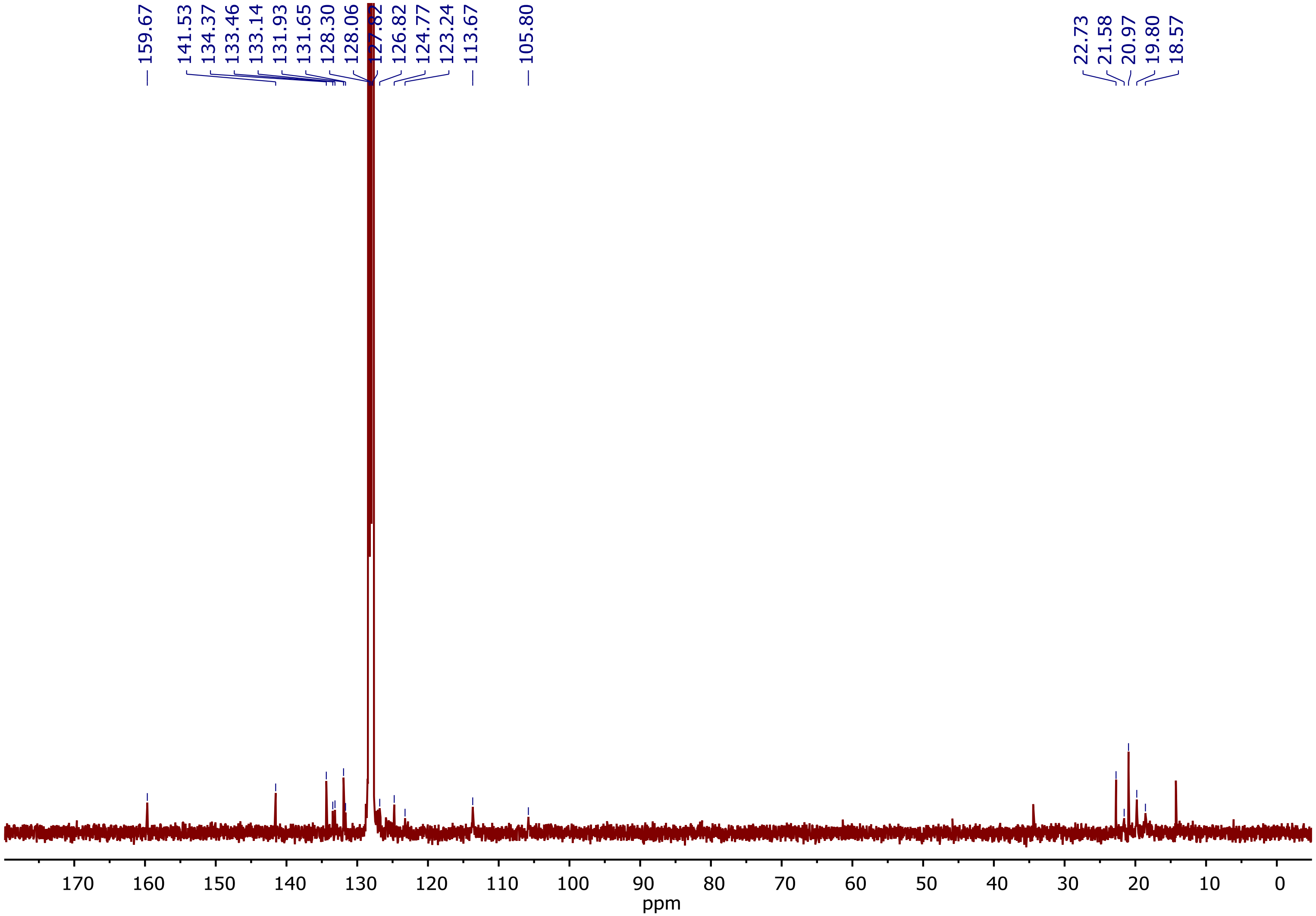

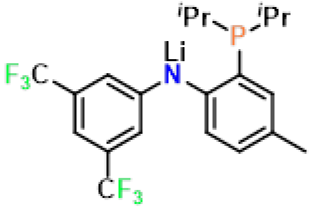

Figure S 99: $^{13}C\{^{1}H\}$ NMR spectrum of **LiPN^{3,5CF3}** in C_6D_6 at 298 K.

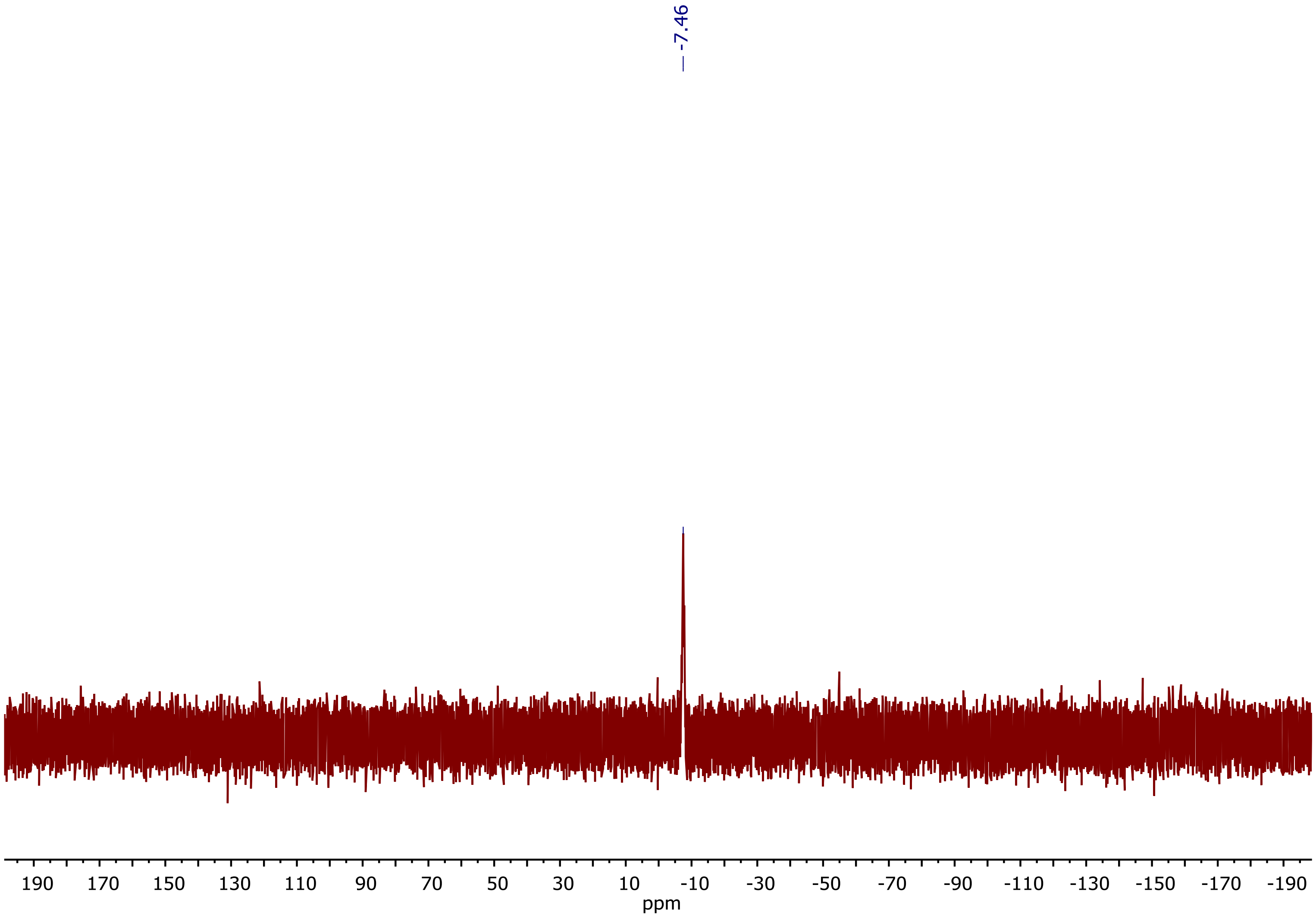

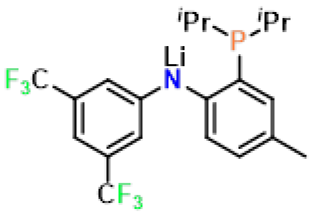

Figure S 100: $^{31}P\{^{1}H\}$ NMR spectrum of ***LiPN***$^{\mathbf{3,5CF3}}$ in C_6D_6 at 298 K.

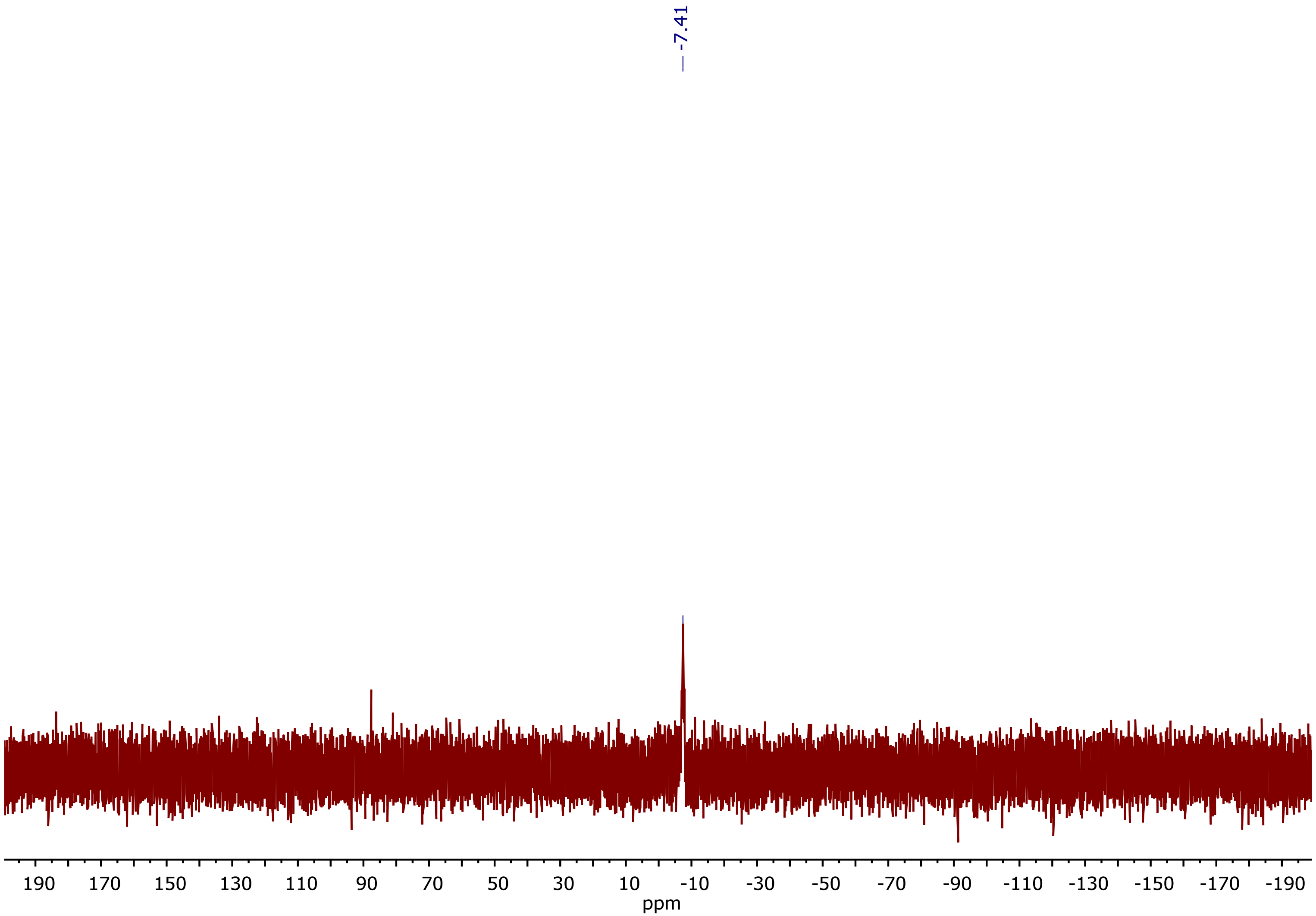

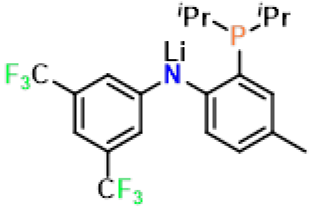

*Figure S 101: ^{31}P NMR spectrum of **LiPN^{3,5CF3}** in C_6D_6 at 298 K.*

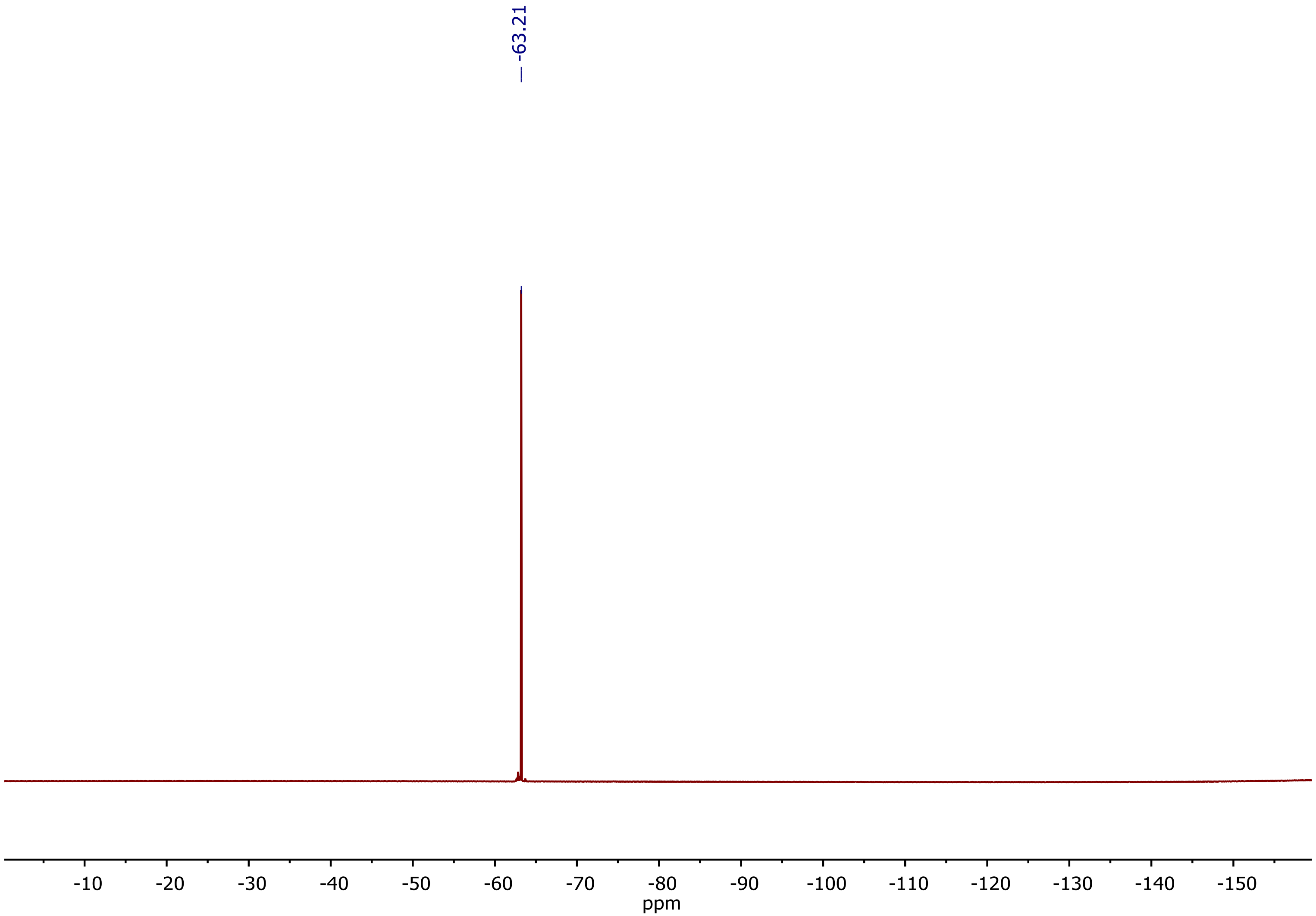

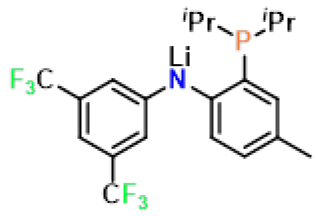

*Figure S 102: ^{19}F NMR spectrum of **LiPN^{3,5CF3}** in C_6D_6 at 298 K.*

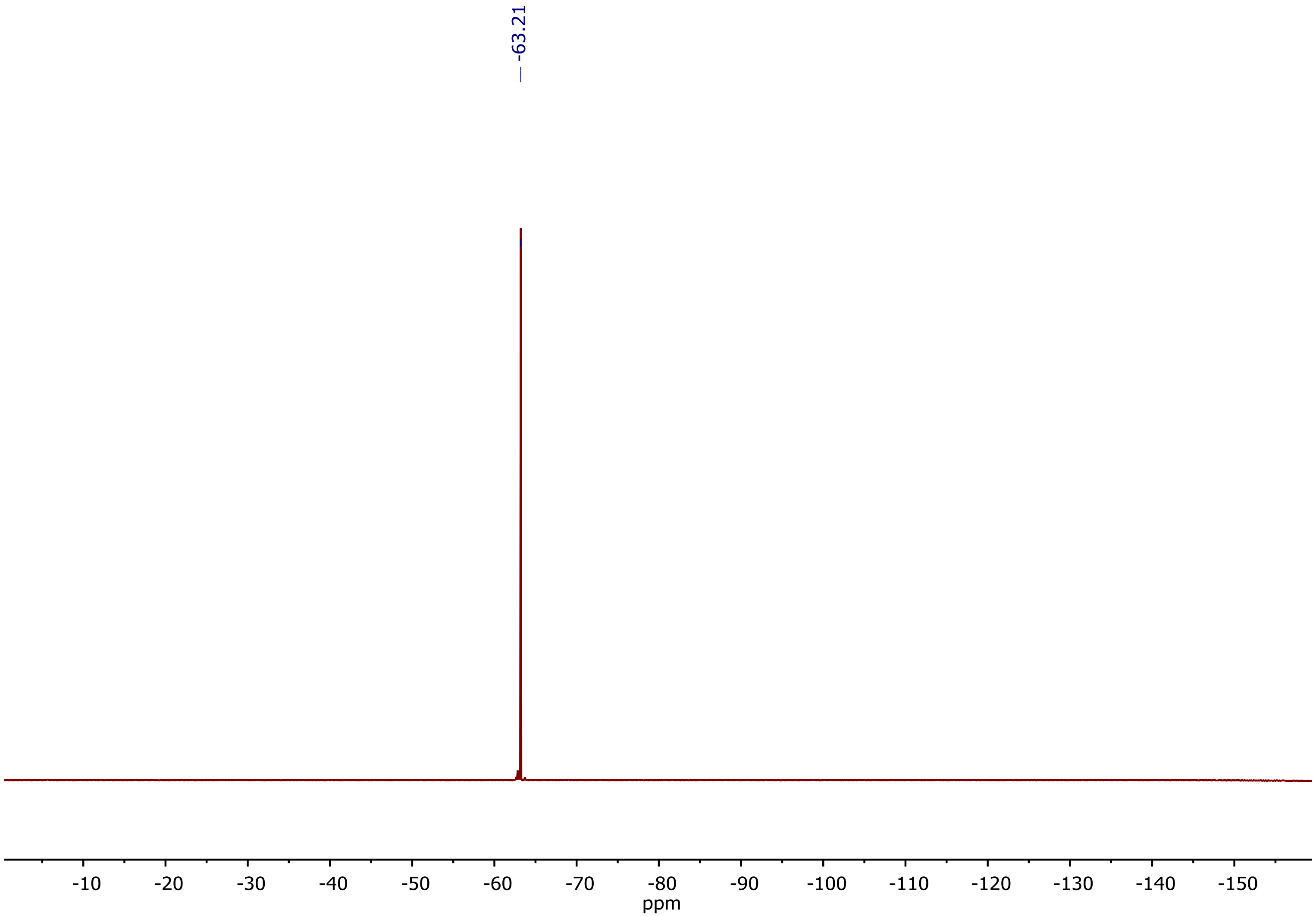

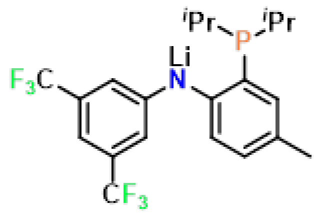

Figure S 103: $^{19}F\{^{1}H\}$ NMR spectrum of ***LiPN^{3,5CF3}*** in C_6D_6 at 298 K.

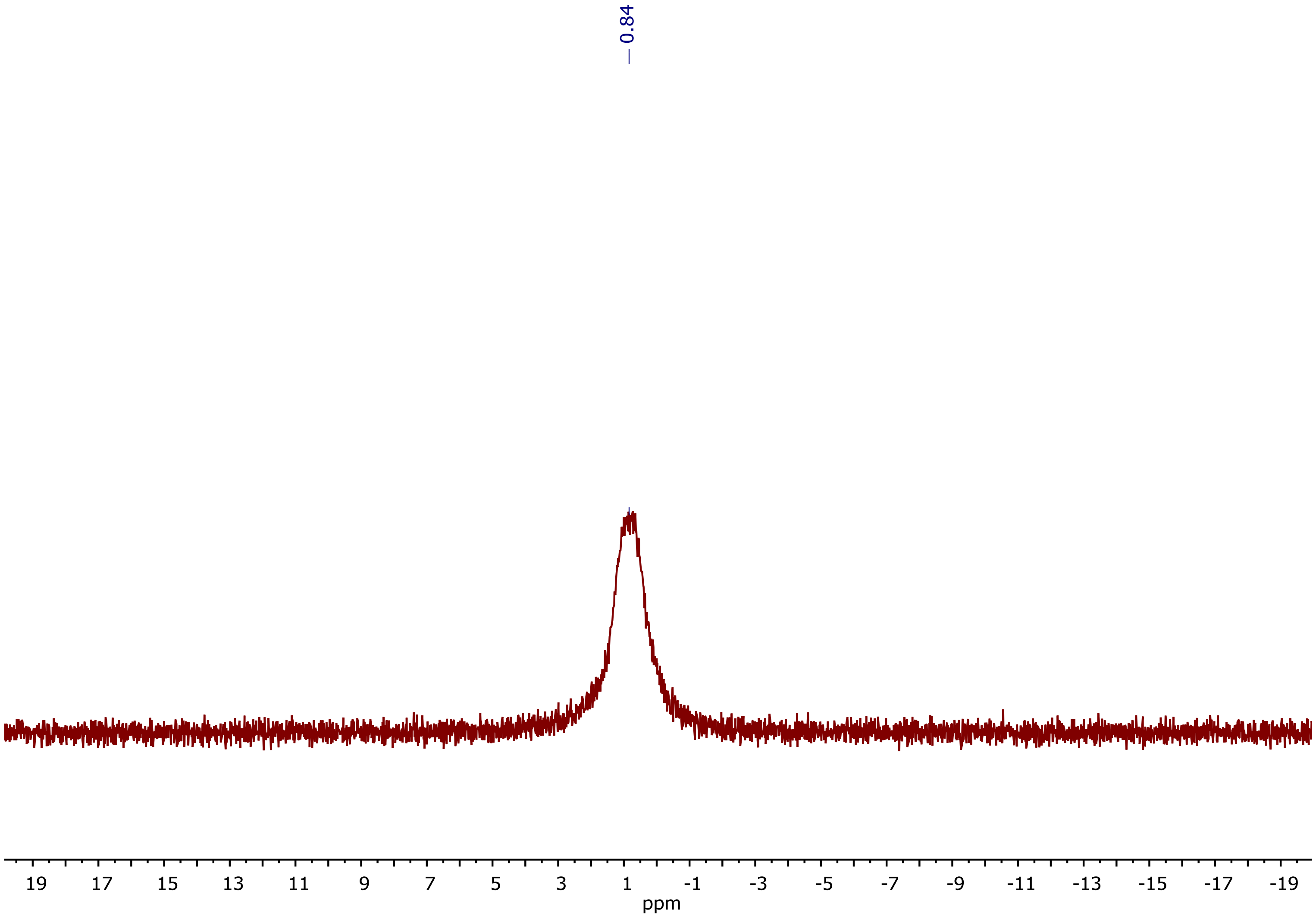

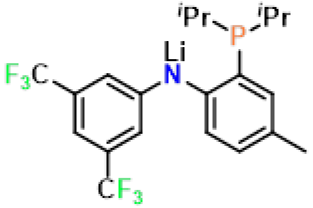

*Figure S 104: ^{7}Li{^{1}H} NMR spectrum of **LiPN^{3,5CF3}** in C_6D_6 at 298 K.*

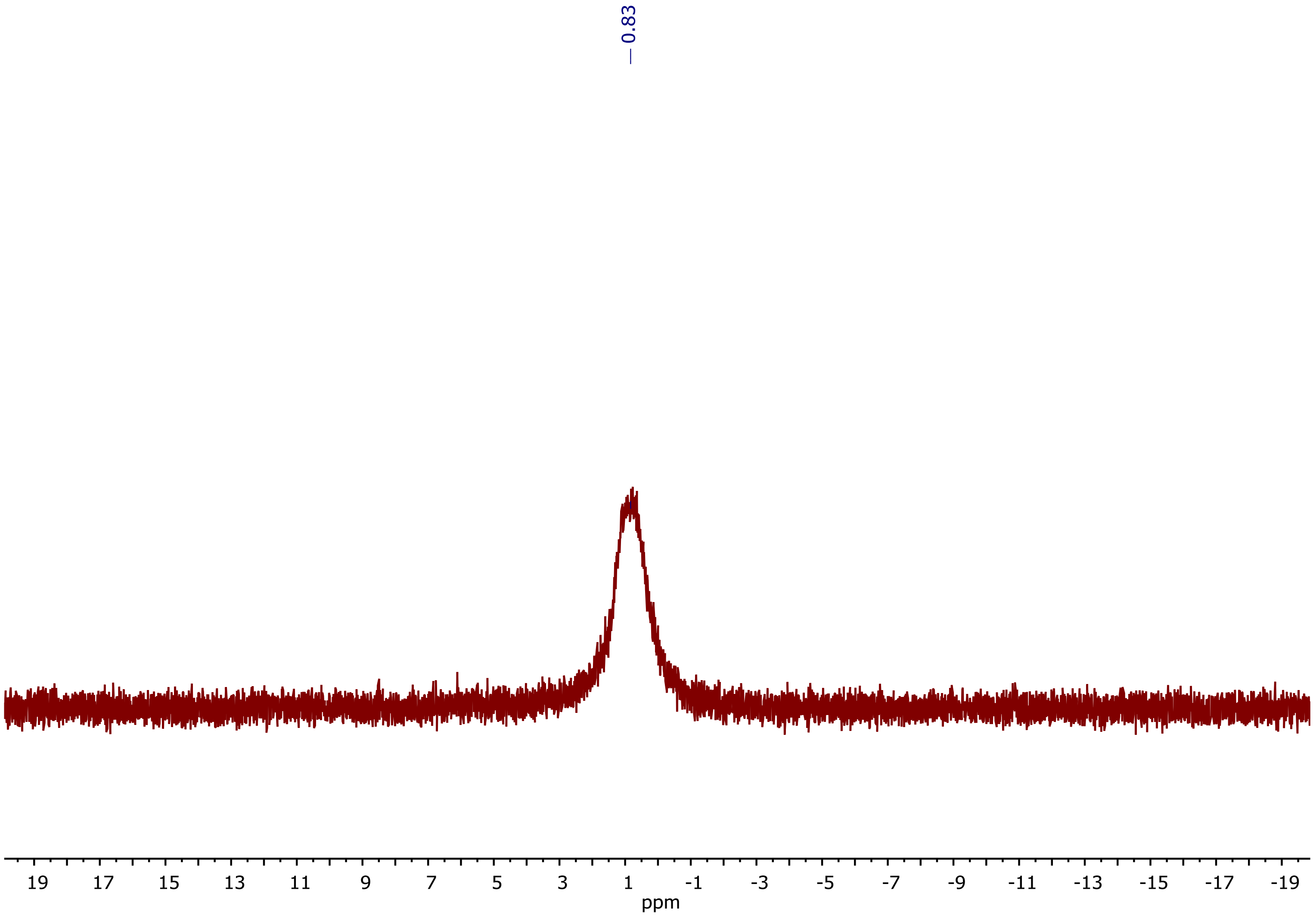

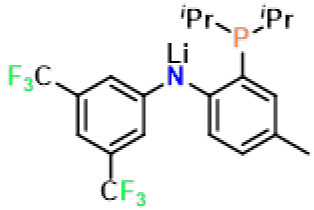

*Figure S 105: ^{7}Li NMR spectrum of **LiPN^{3,5CF3}** in C_6D_6 at 298 K.*

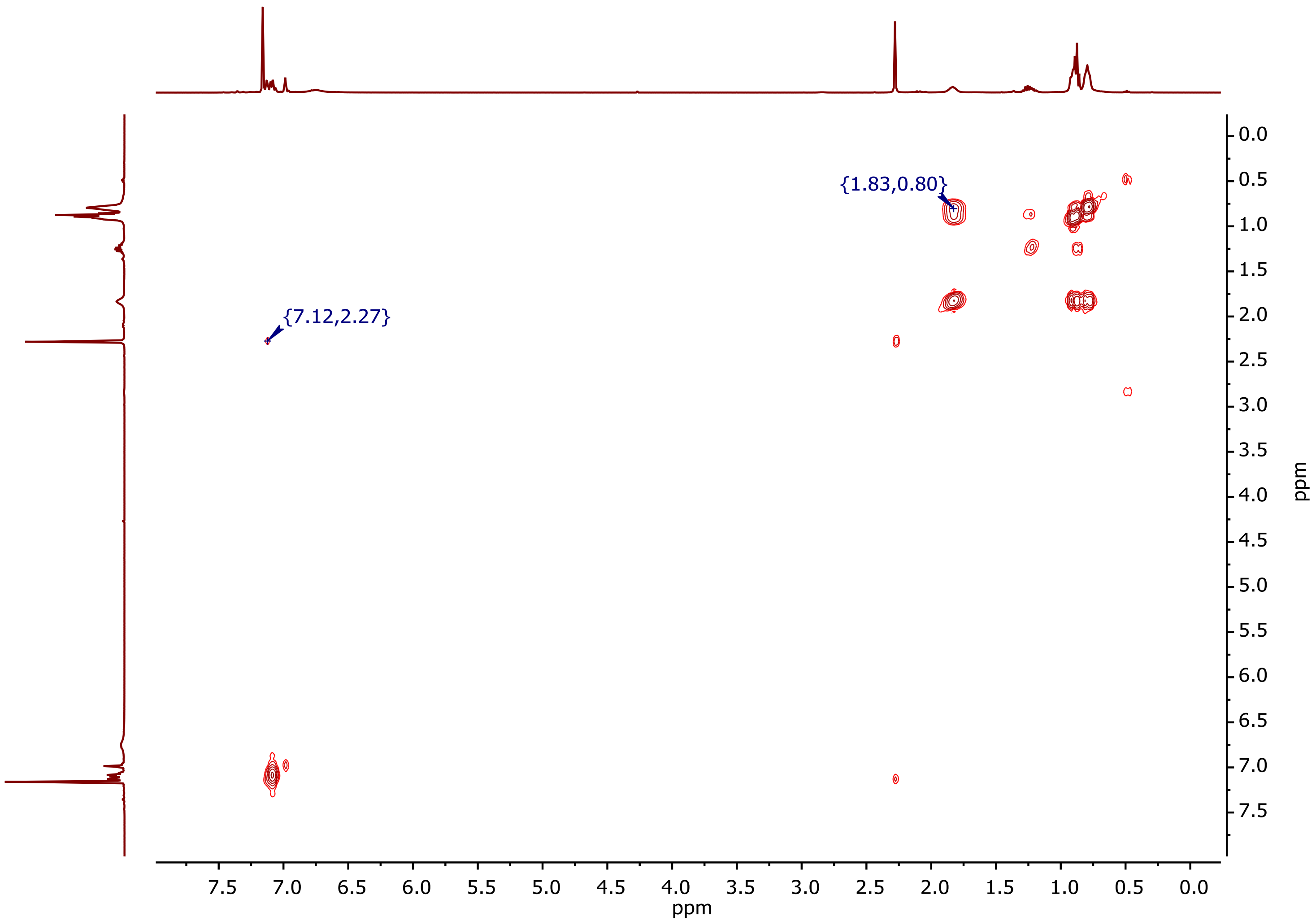

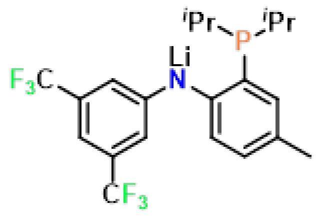

Figure S 106: ^{1}H-^{1}H COSY NMR spectrum of ***LiPN***^{3,5CF3} in C_6D_6 at 298 K.

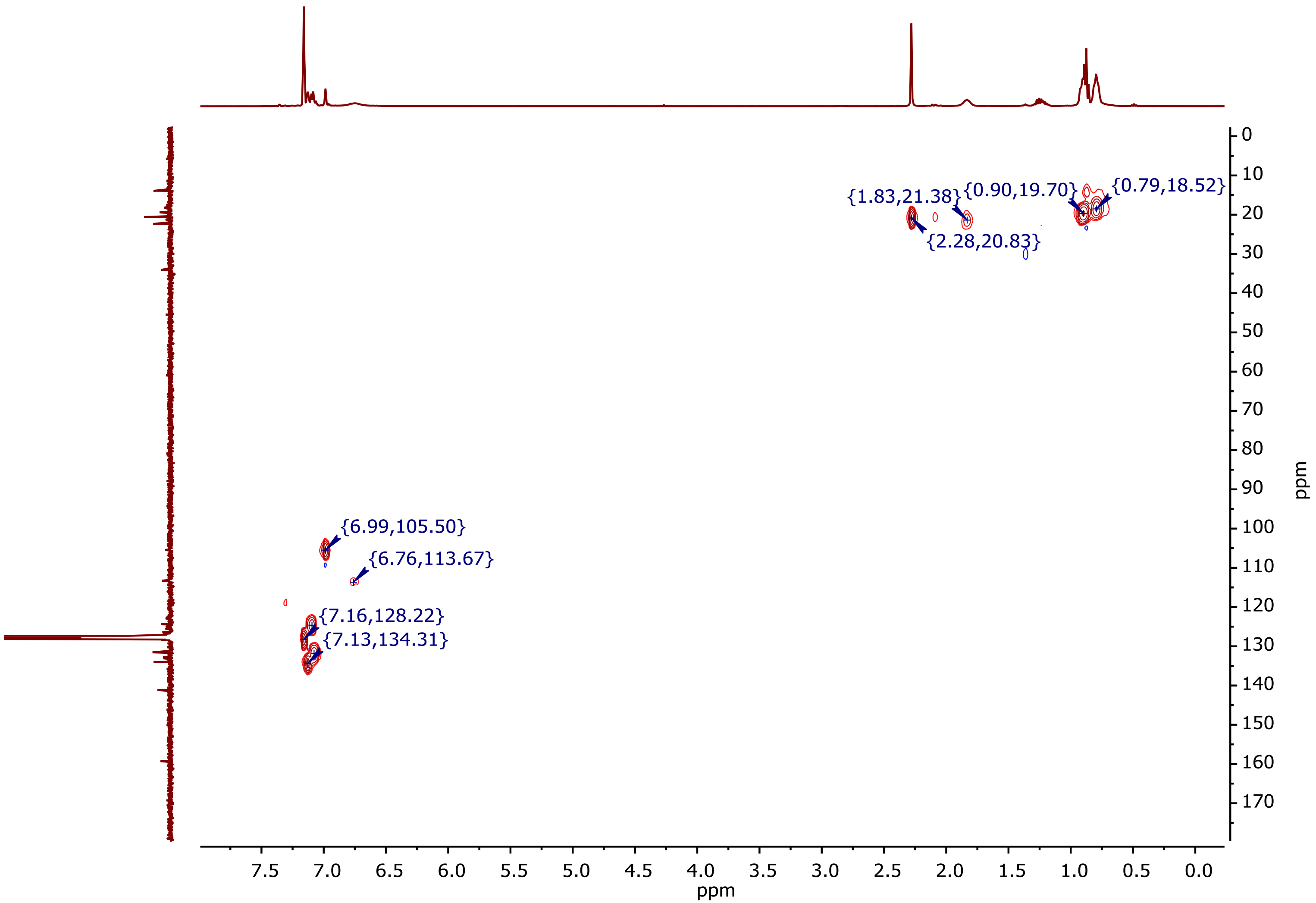

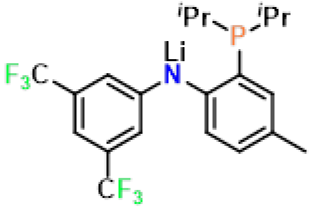

*Figure S 107: ^{1}H-^{13}C HSQC NMR spectrum of **$LiPN^{3,5CF3}$** in C_6D_6 at 298 K.*

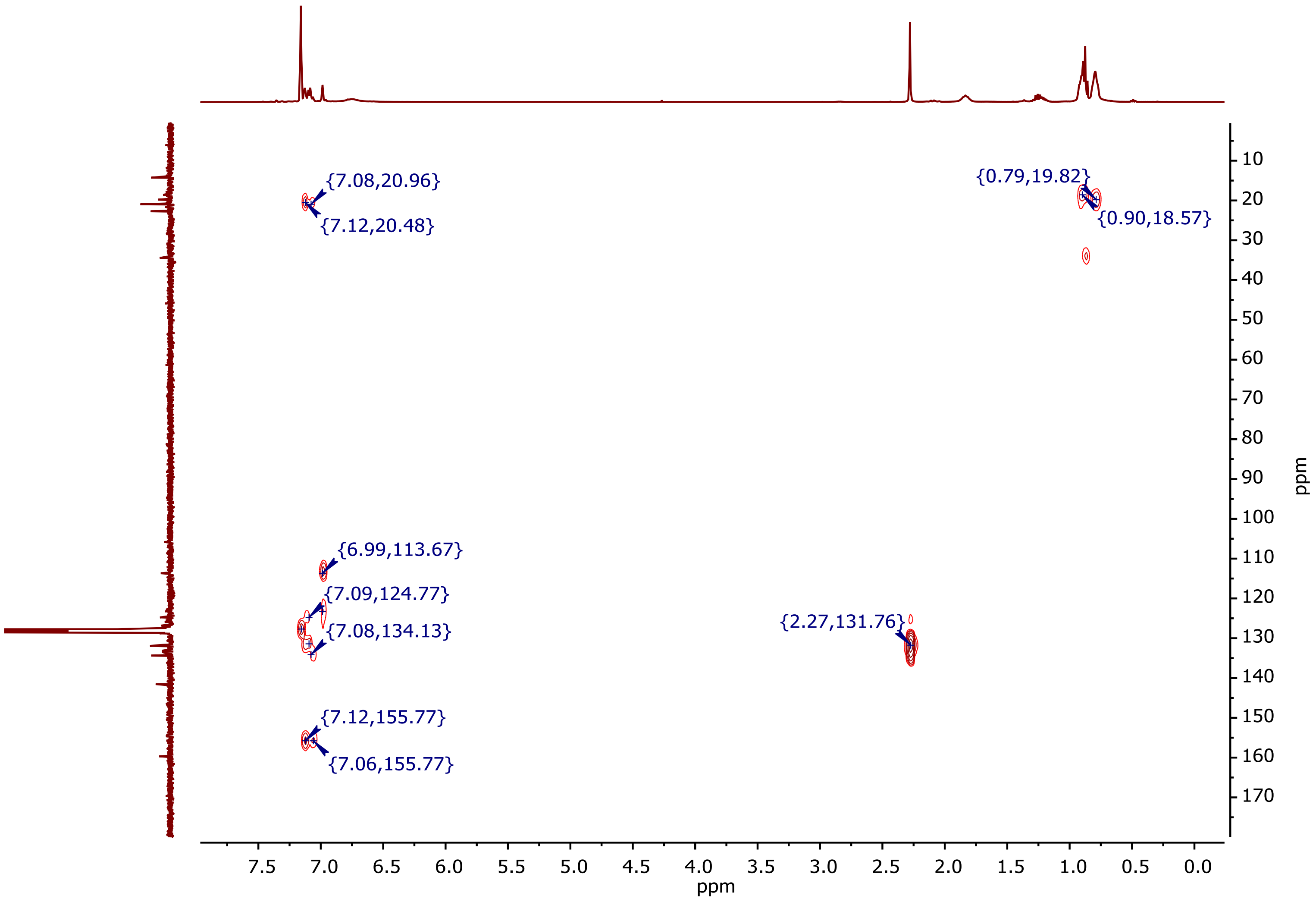

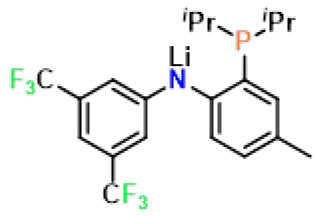

Figure S 108: ^{1}H-^{13}C HMBC NMR spectrum of **LiPN^{3,5CF3}** in C_6D_6 at 298 K.

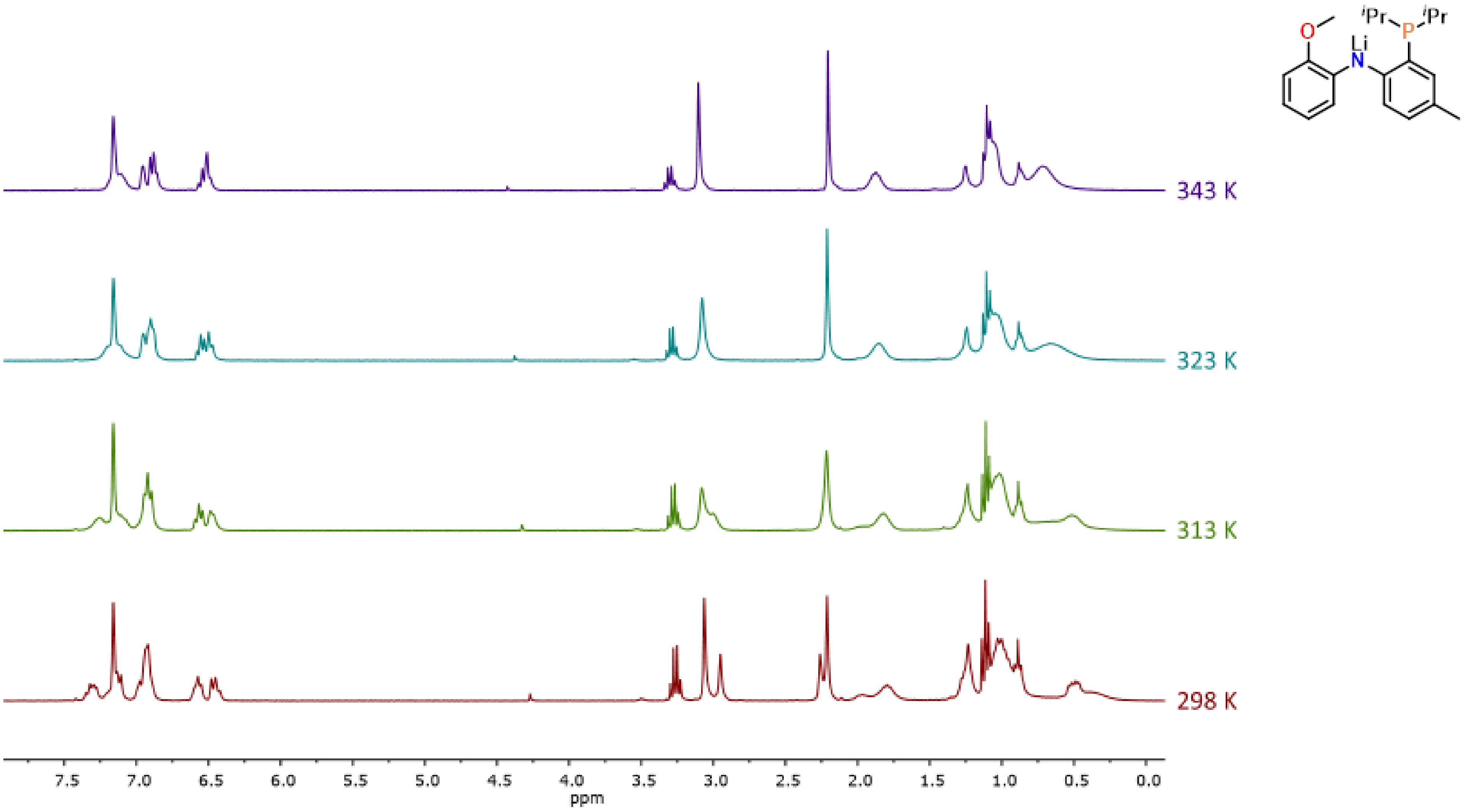

*Figure S 109: VT ¹H NMR spectrum of **LiPN^{OMe}** at 298 K (red), 313 K (green), 323 K (blue) and 343 K (purple).*

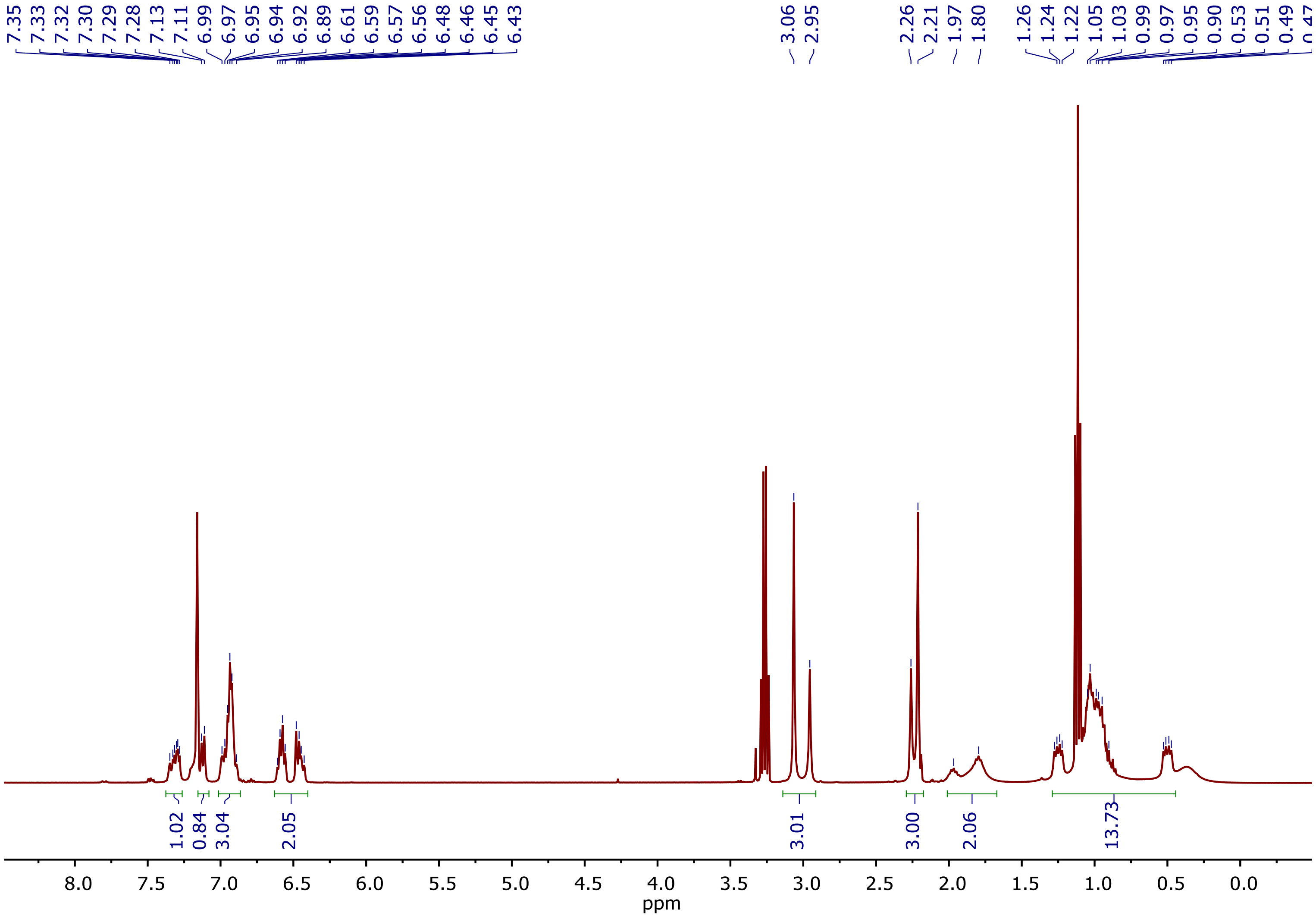

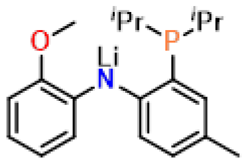

*Figure S 110: ¹H NMR spectrum of **LiPN^{OMe}** in C_6D_6 at 298 K.*

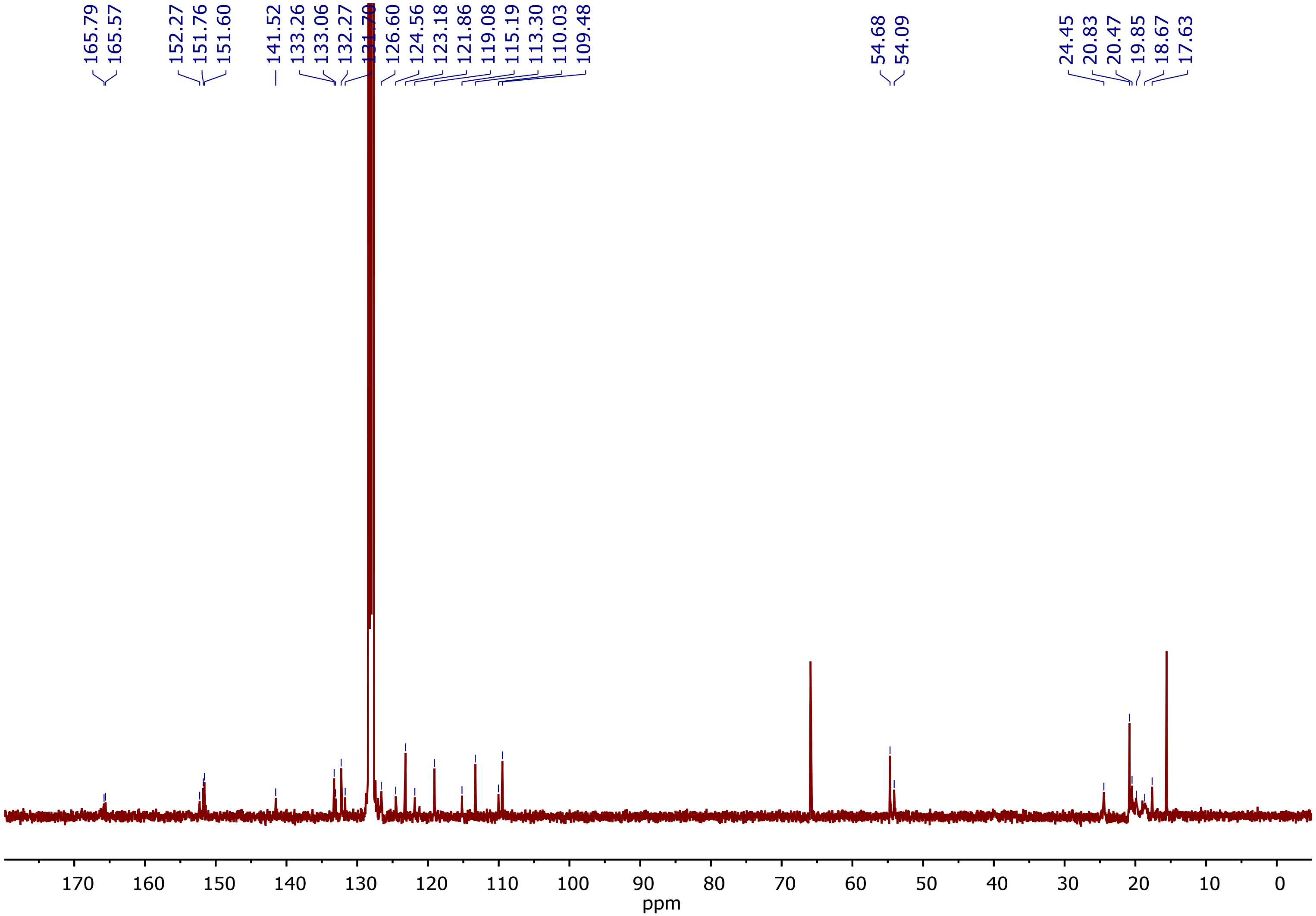

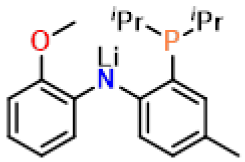

*Figure S 111: $^{13}C\{^{1}H\}$ NMR spectrum of **LiPN^{OMe}** in C_6D_6 at 298 K.*

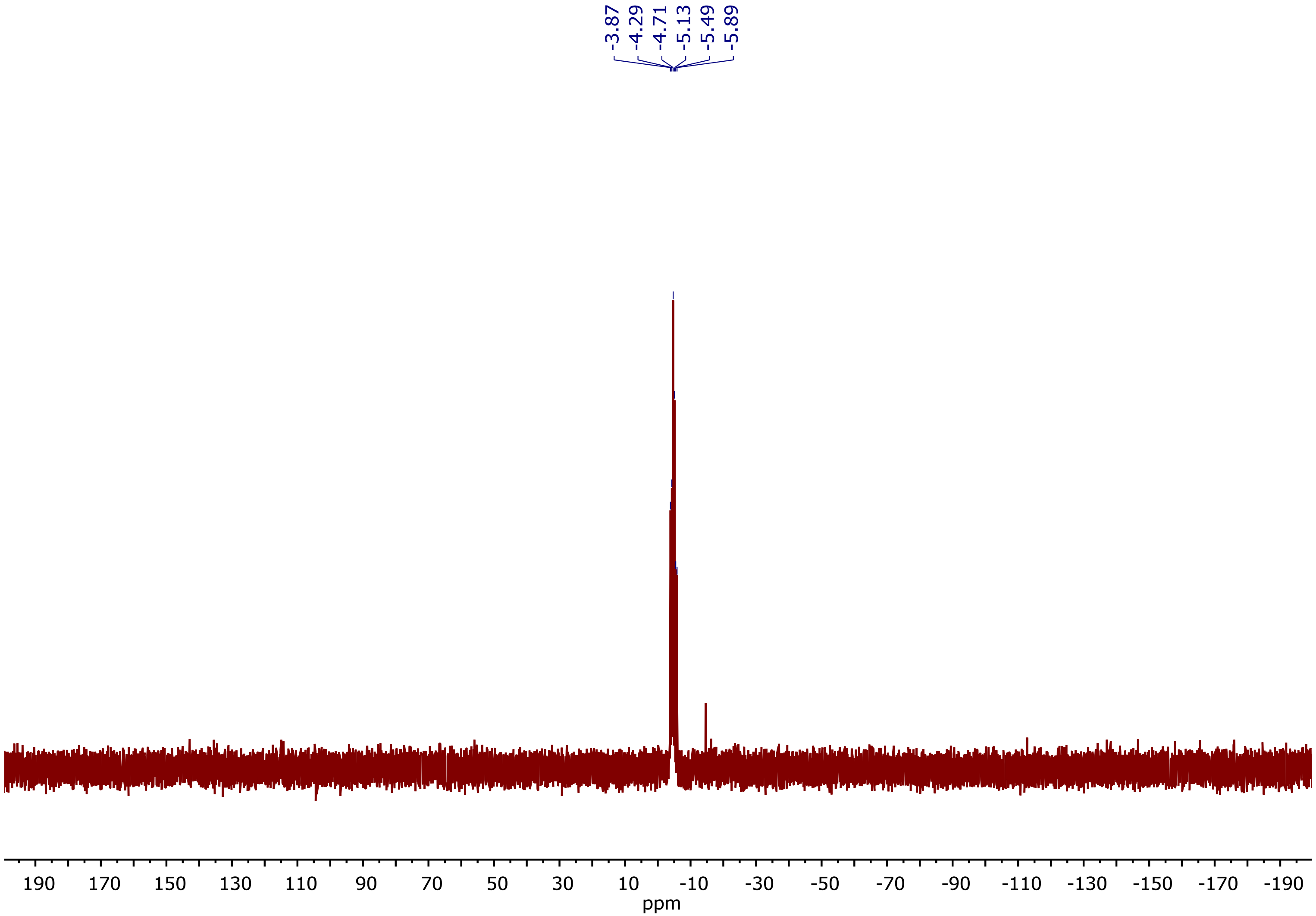

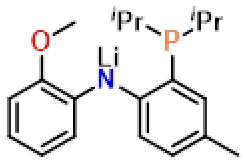

*Figure S 112: $^{31}P\{^{1}H\}$ NMR spectrum of **LiPN^{OMe}** in C_6D_6 at 298 K.*

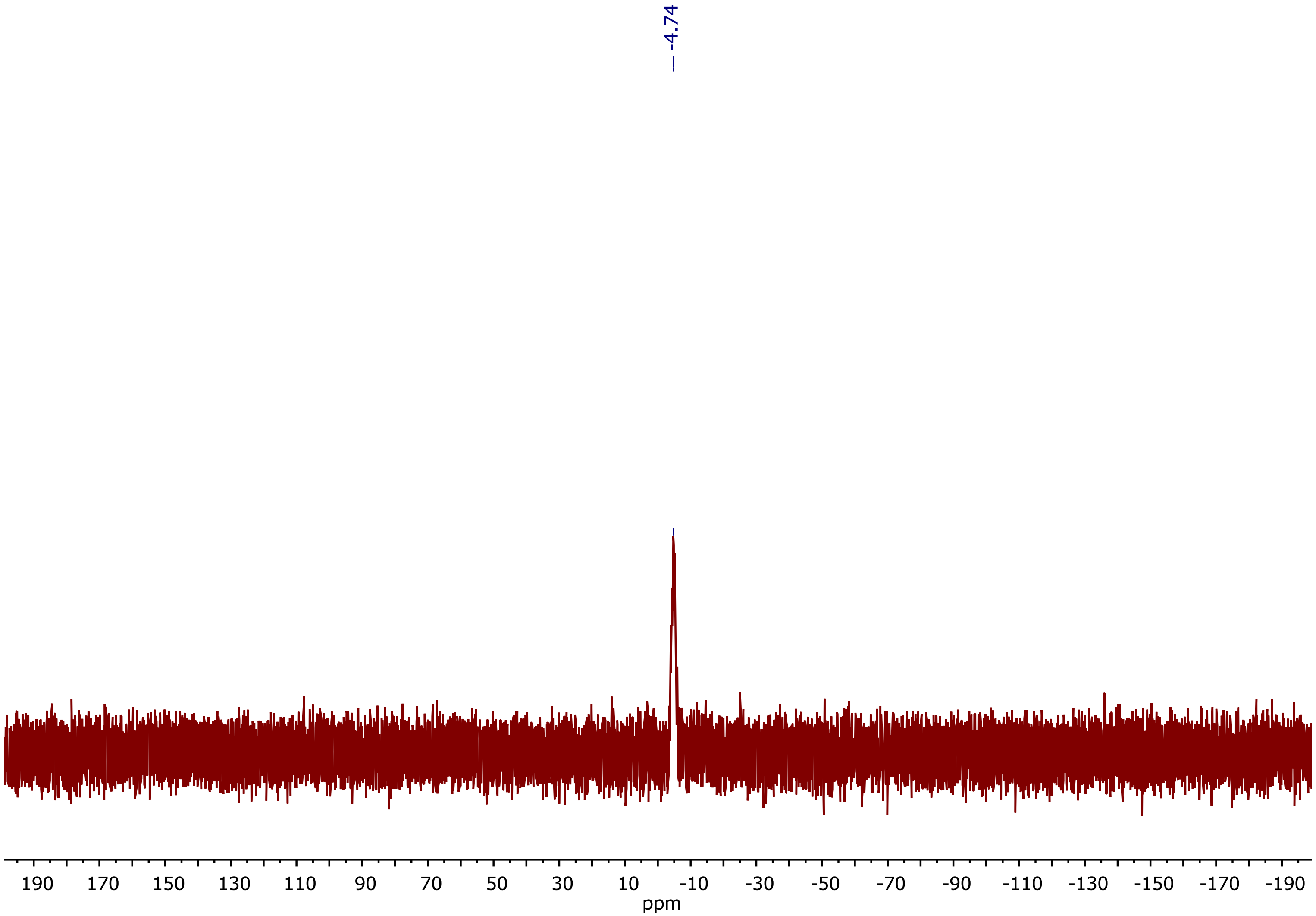

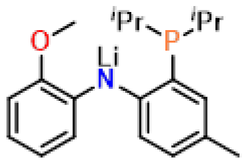

*Figure S 113: ^{31}P NMR spectrum of **LiPN^{OMe}** in C_6D_6 at 298 K.*

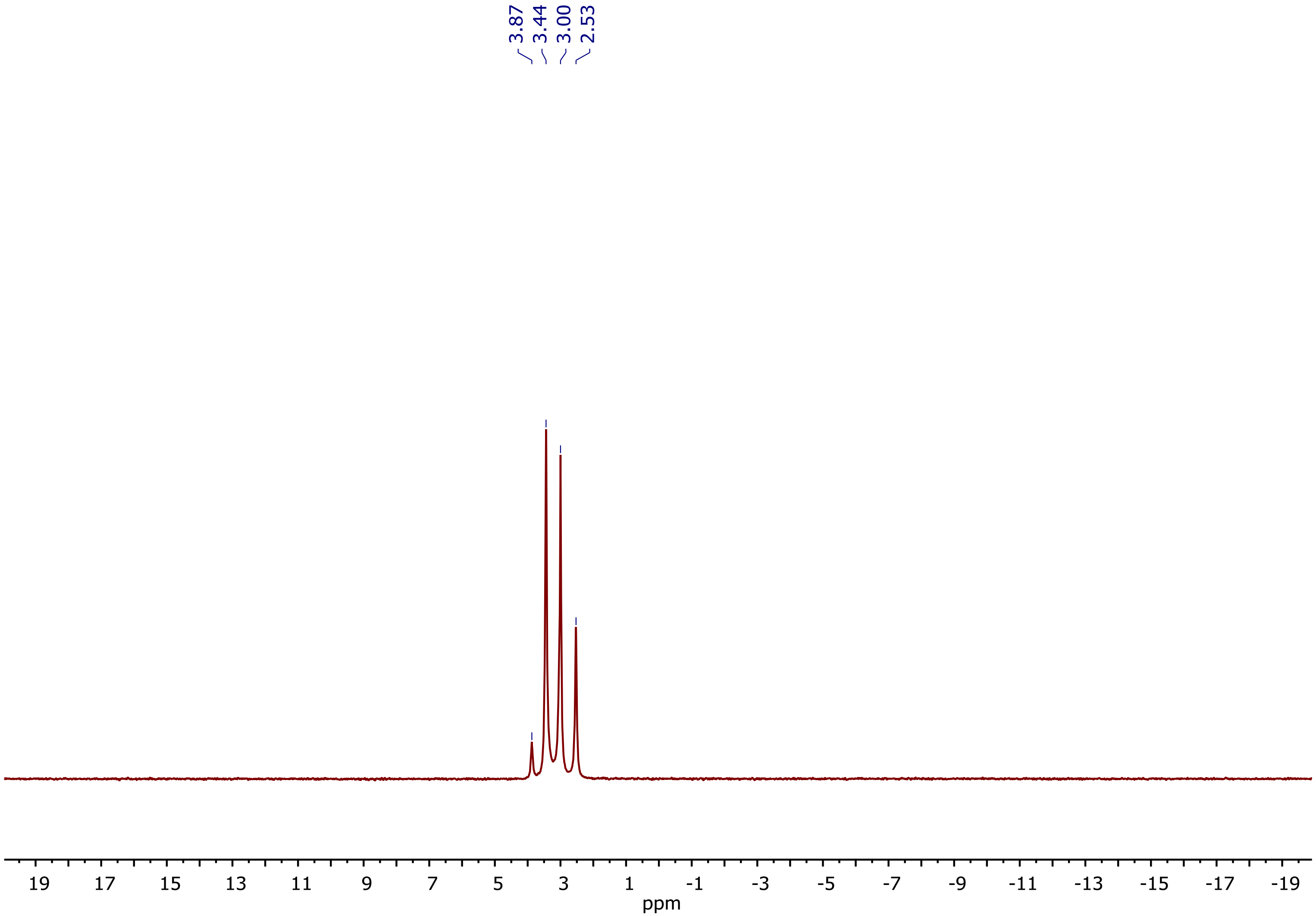

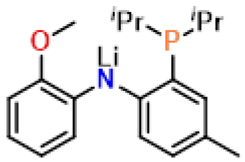

*Figure S 114: ^{7}Li{^{1}H} NMR spectrum of **LiPN^{OMe}** in C_6D_6 at 298 K.*

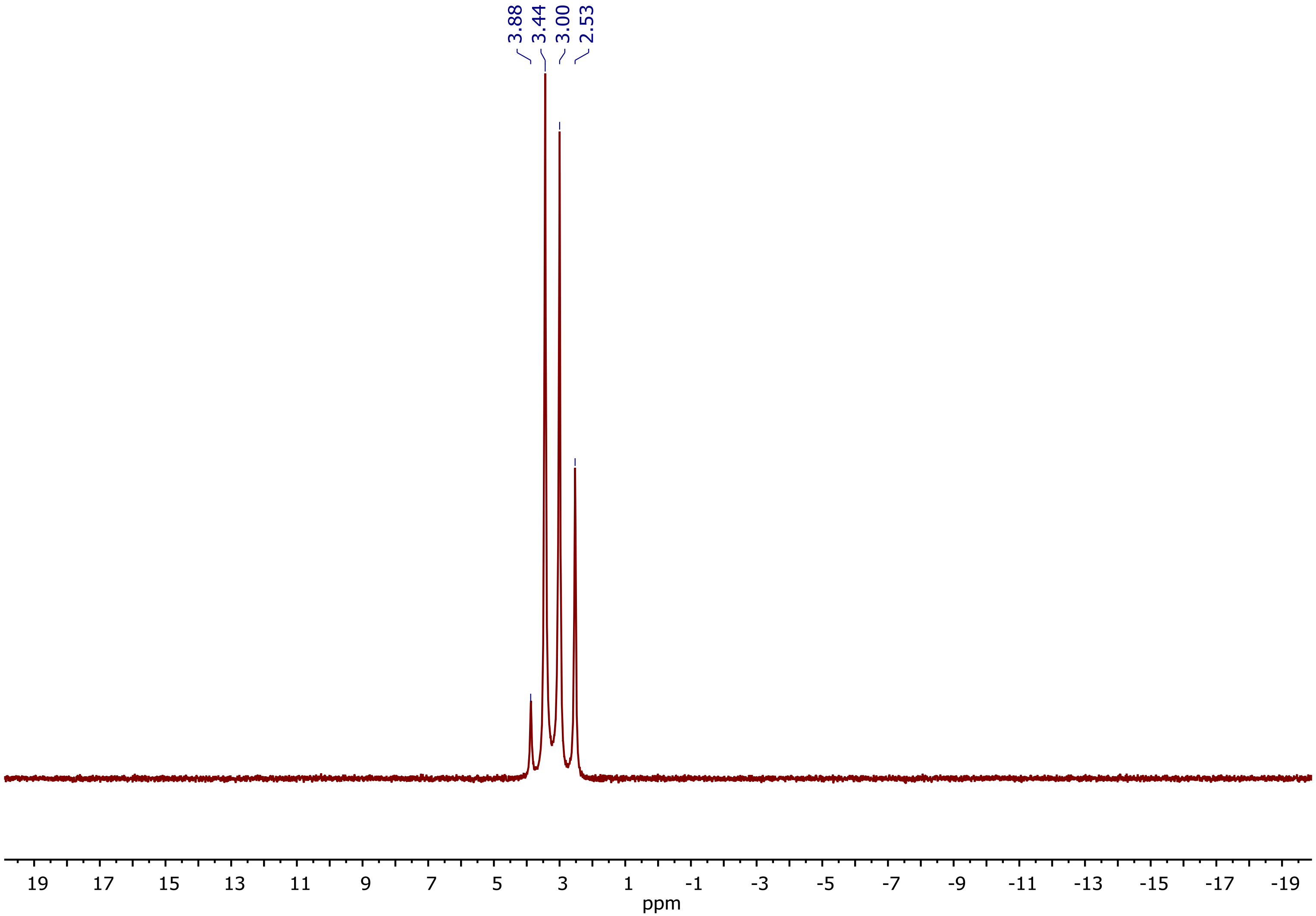

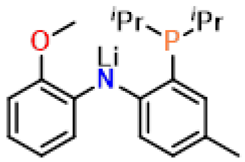

*Figure S 115: $^{7}Li\{^{1}H\}$ NMR spectrum of **LiPN^{OMe}** in C_6D_6 at 298 K.*

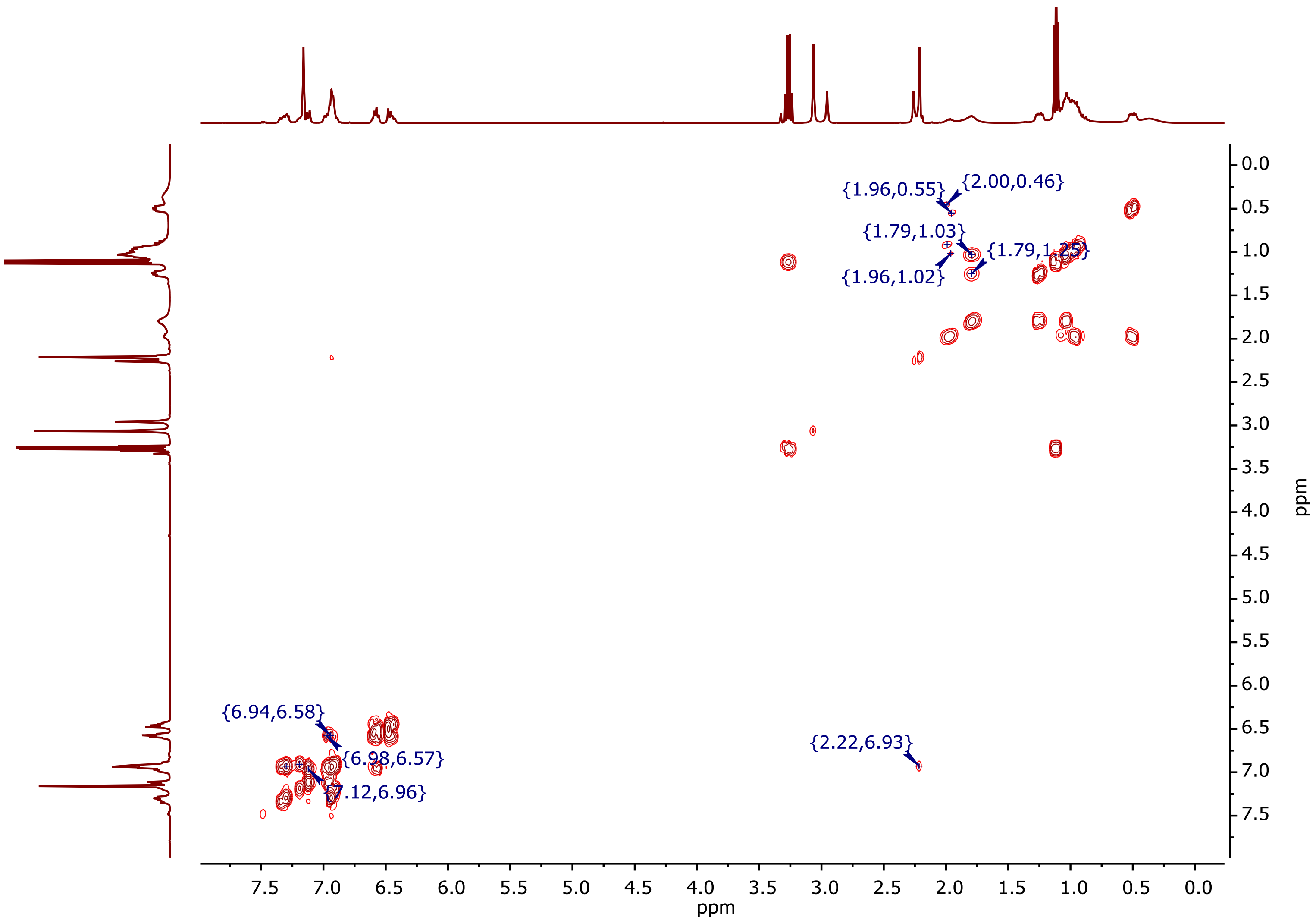

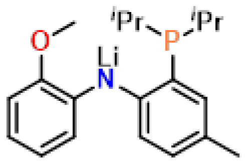

Figure S 116: ^{1}H-^{1}H COSY NMR spectrum of ***LiPN^{OMe}*** in C_6D_6 at 298 K.

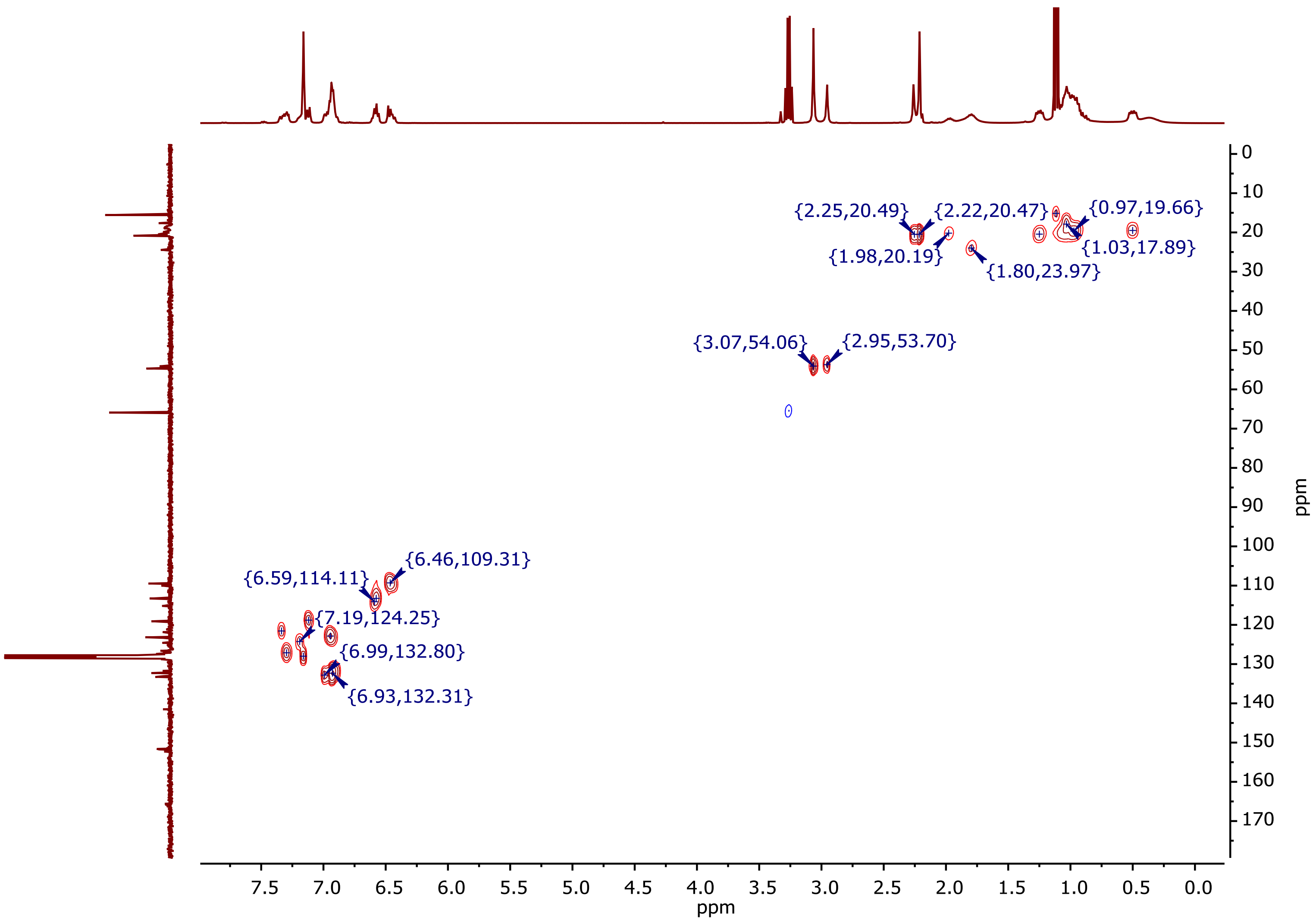

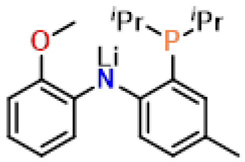

Figure S 117: ^{1}H-^{13}C HSQC NMR spectrum of **LiPN^{OMe}** in C_6D_6 at 298 K.

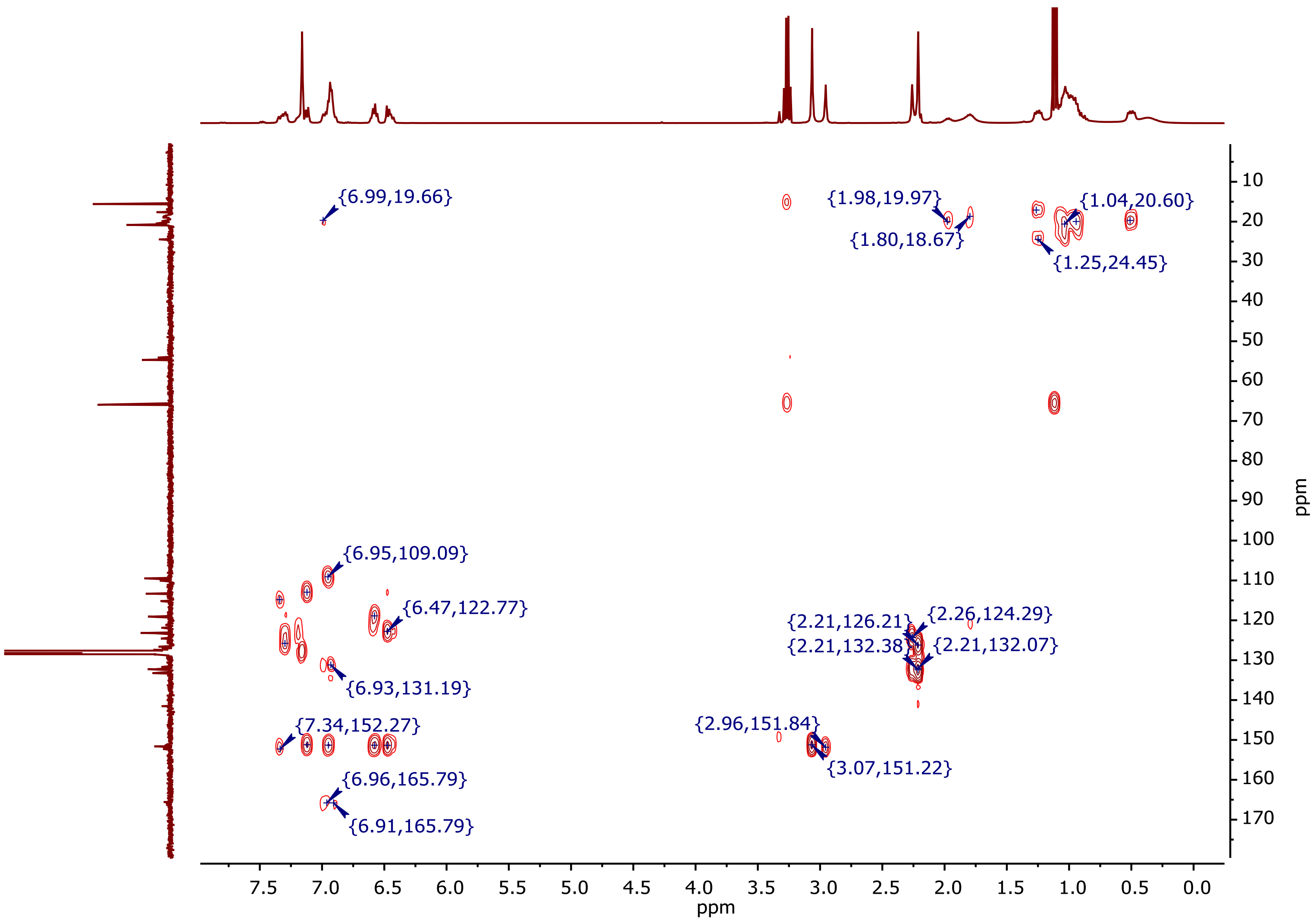

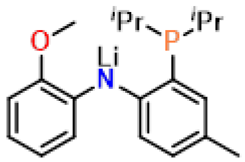

*Figure S 118: ^{1}H-^{13}C HMBC NMR spectrum of **LiPN^{OMe}** in C_6D_6 at 298 K.*

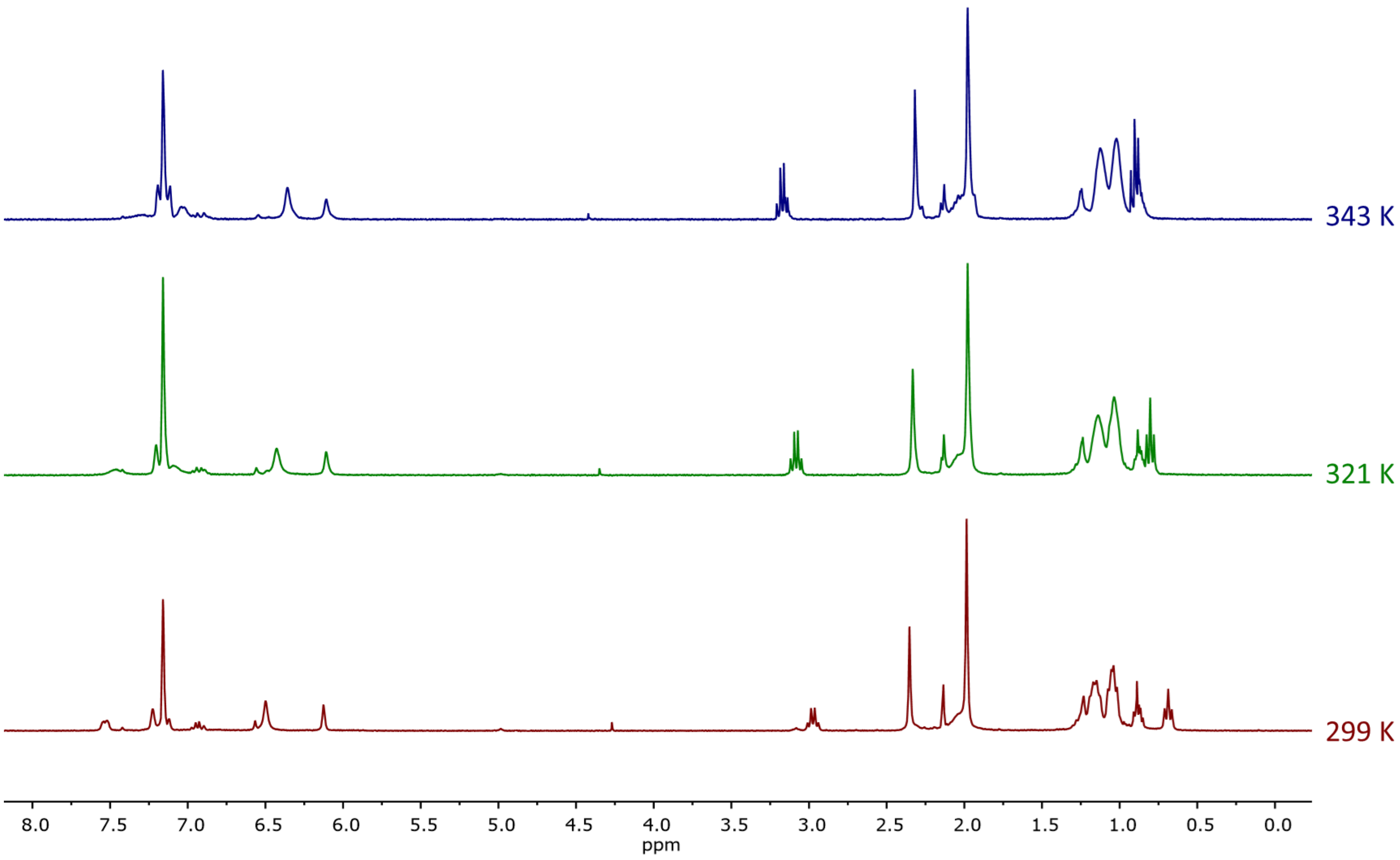

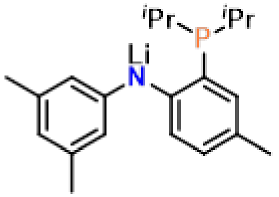

Figure S 119: VT ^{1}H NMR spectrum of ***LiPN^{3,5Me}*** *at 299 K (red), 321 K (green) and 343 K (blue).*

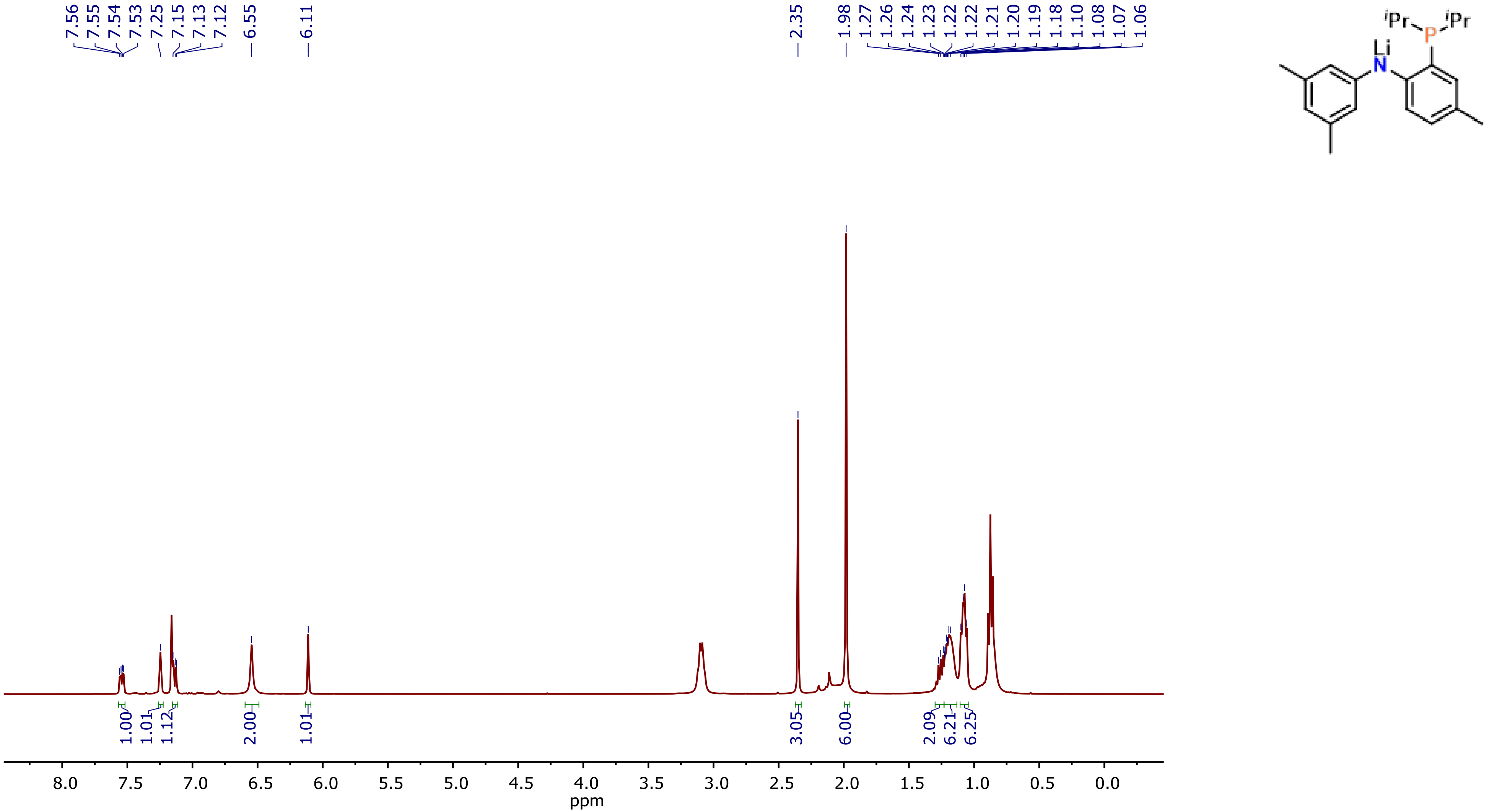

*Figure S 120: ¹H NMR spectrum of **LiPN^{3,5Me}** in C_6D_6 at 298 K.*

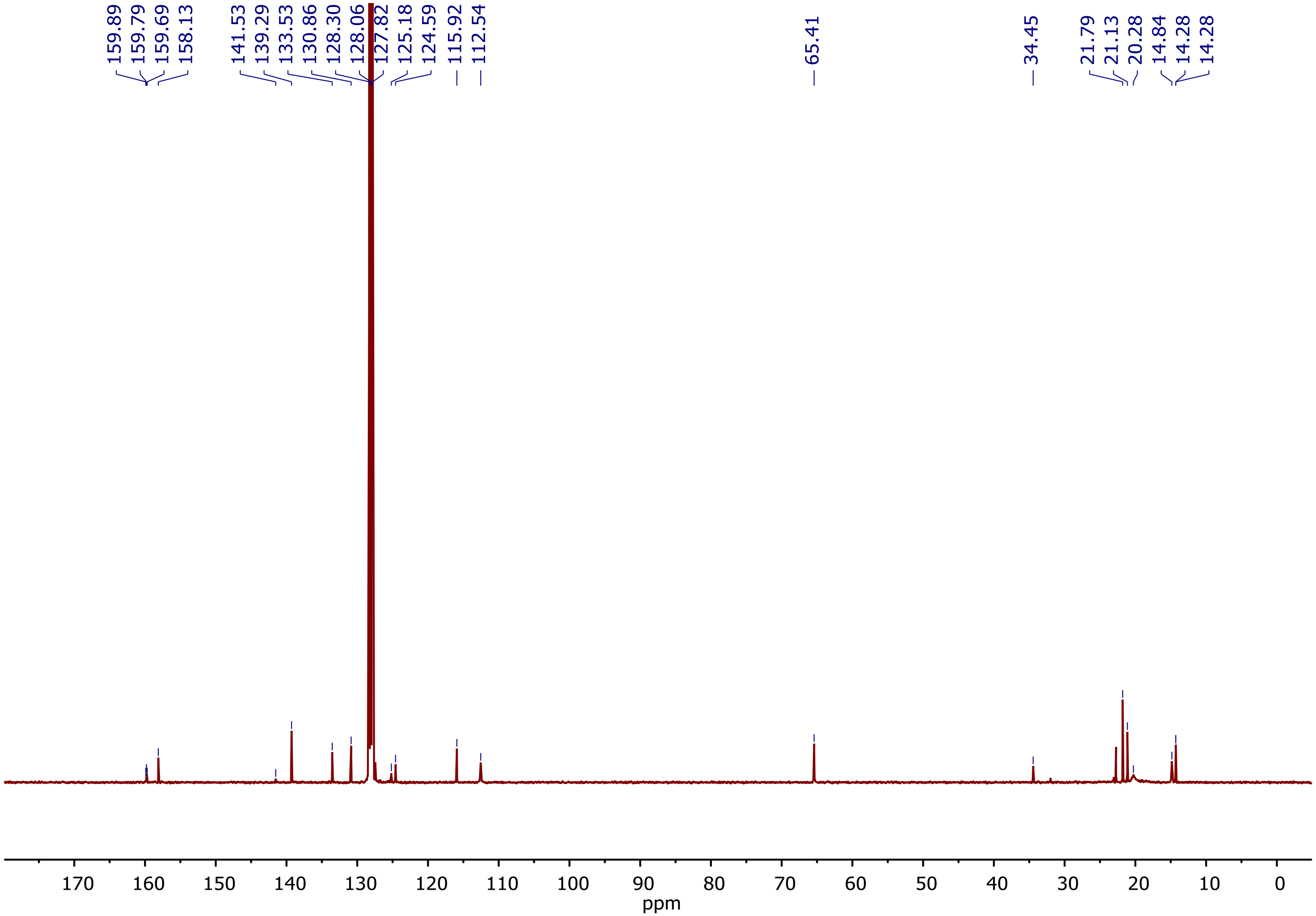

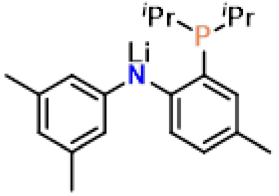

*Figure S 121: $^{13}C\{^1H\}$ NMR spectrum of **LiPN^{3,5Me}** in C_6D_6 at 298 K.*

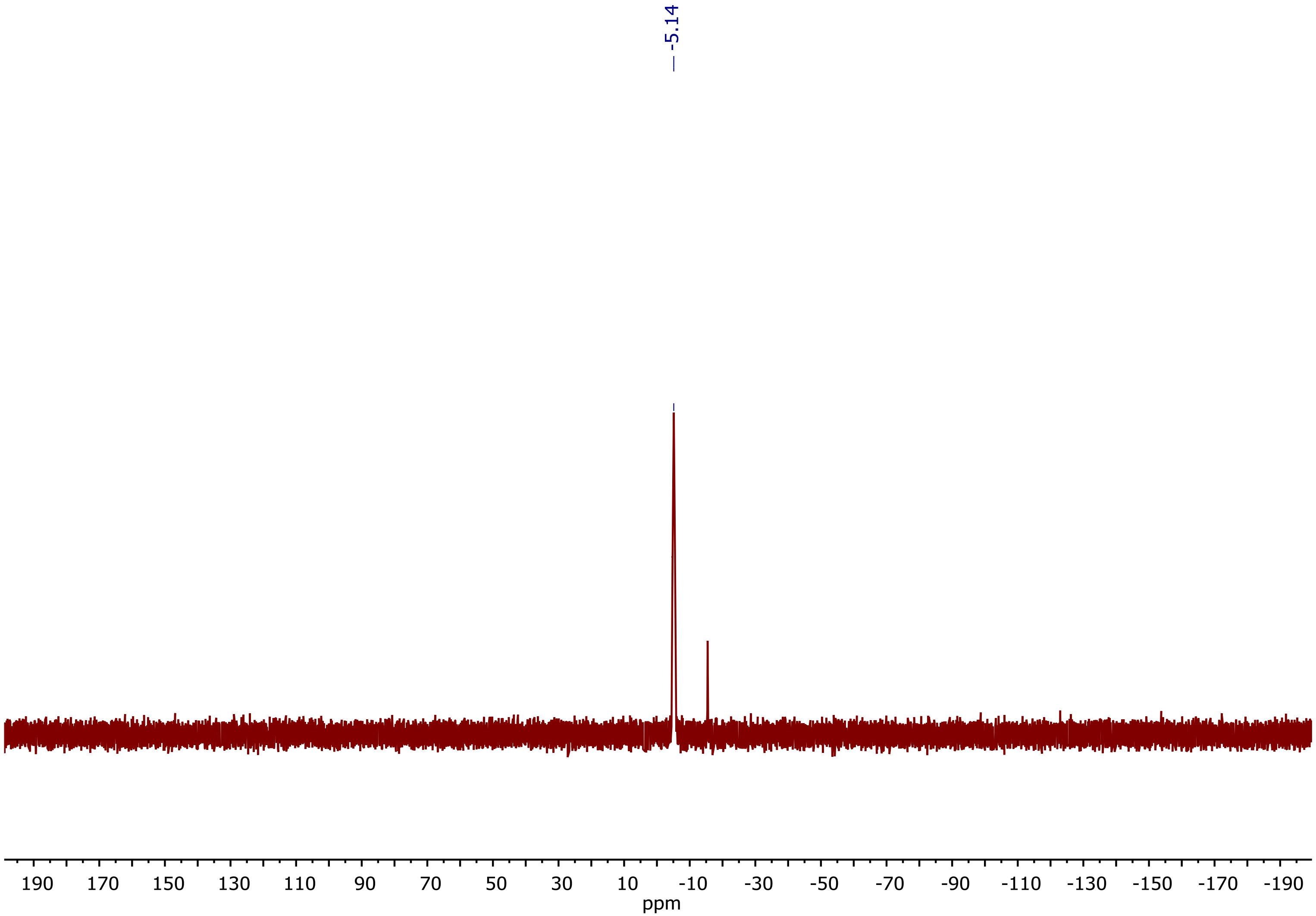

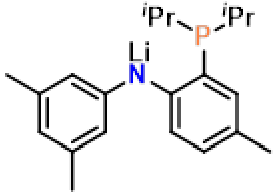

*Figure S 122: $^{31}P\{^{1}H\}$ NMR spectrum of **LiPN^{3,5Me}** in C_6D_6 at 298 K.*

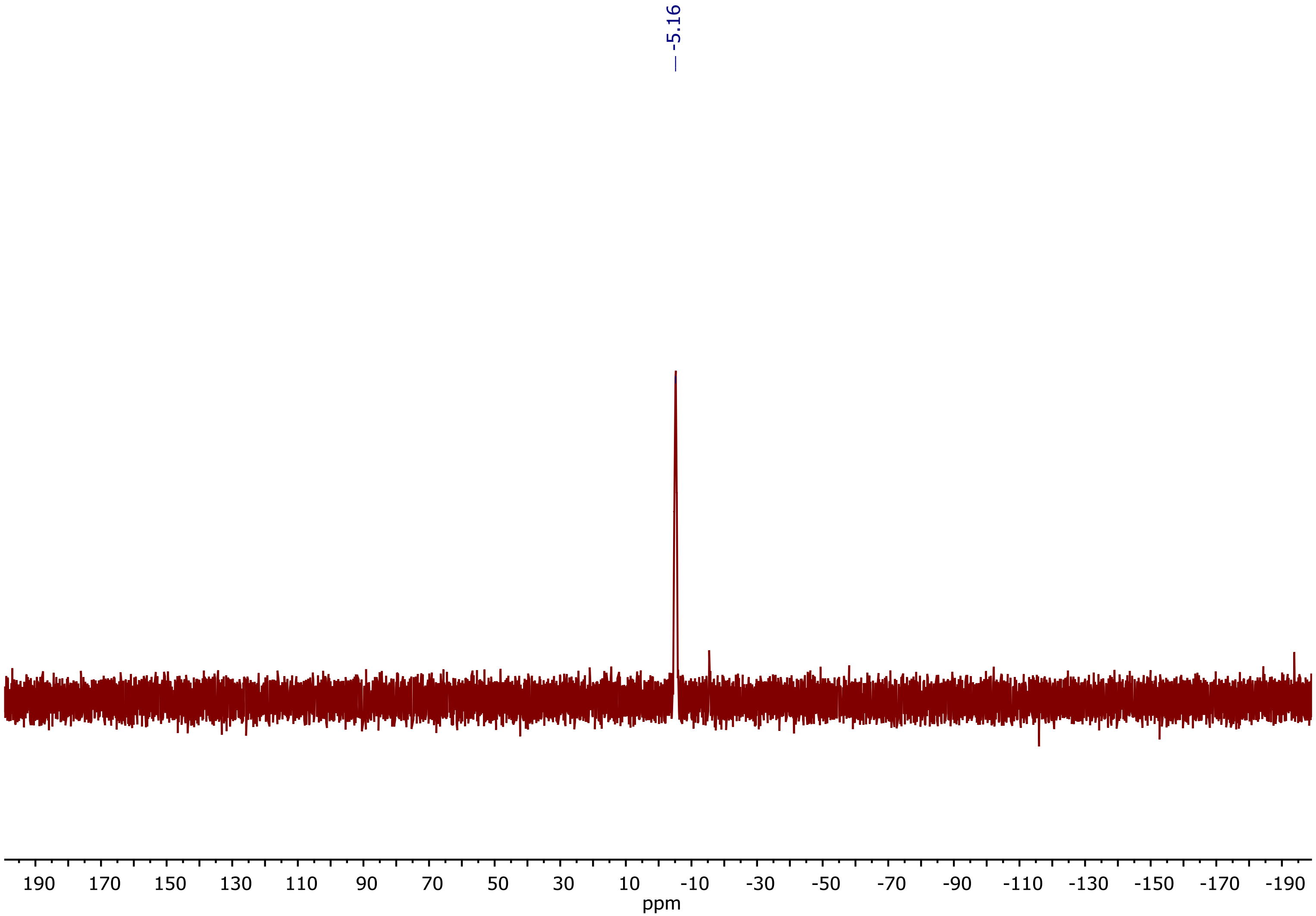

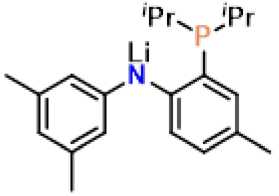

*Figure S 123: ^{31}P NMR spectrum of **LiPN^{3,5Me}** in C_6D_6 at 298 K.*

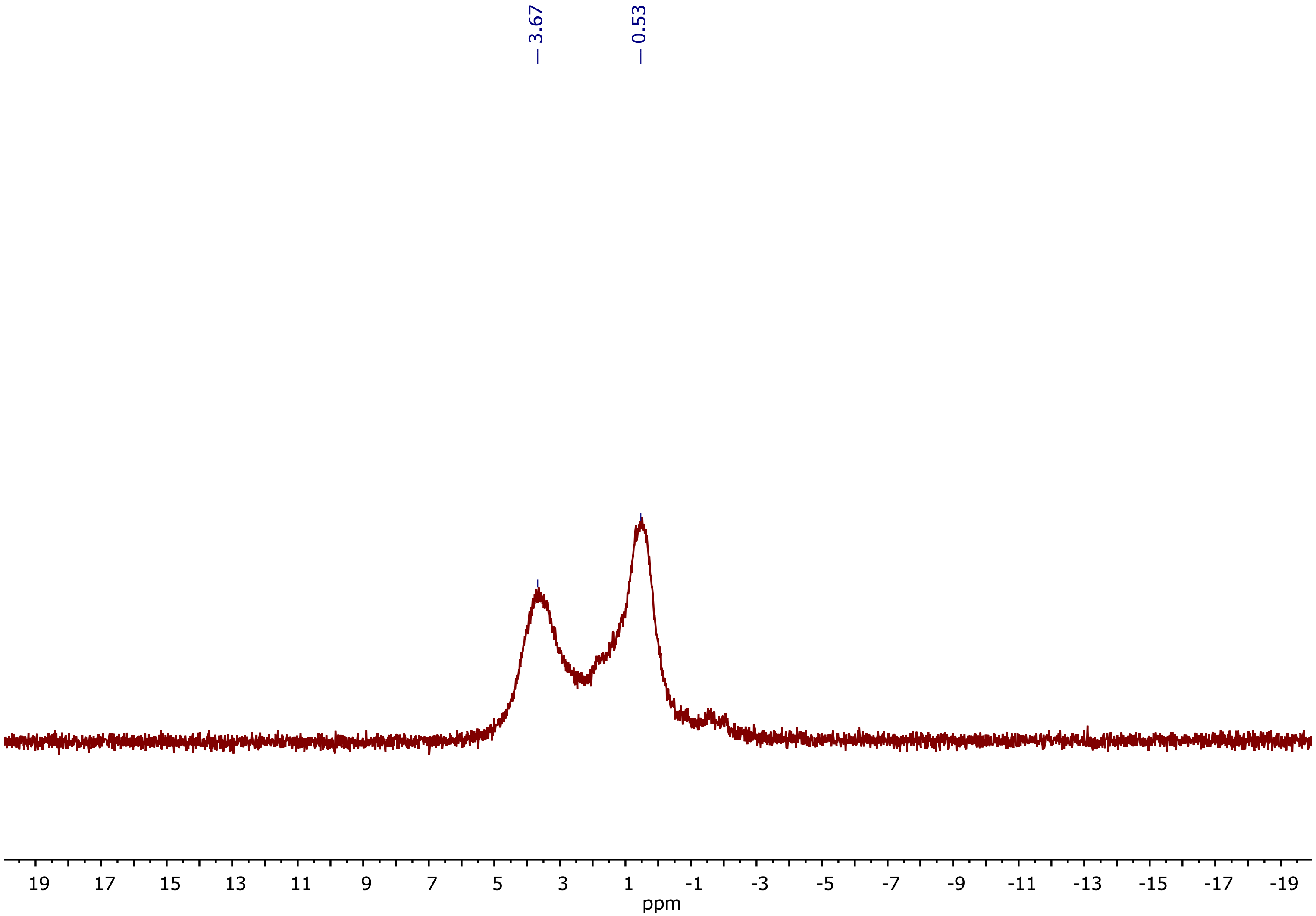

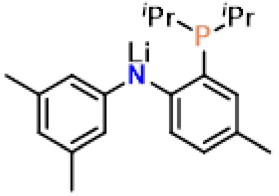

*Figure S 124: ^{7}Li{^{1}H} NMR spectrum of **LiPN^{3,5Me}** in C_6D_6 at 298 K.*

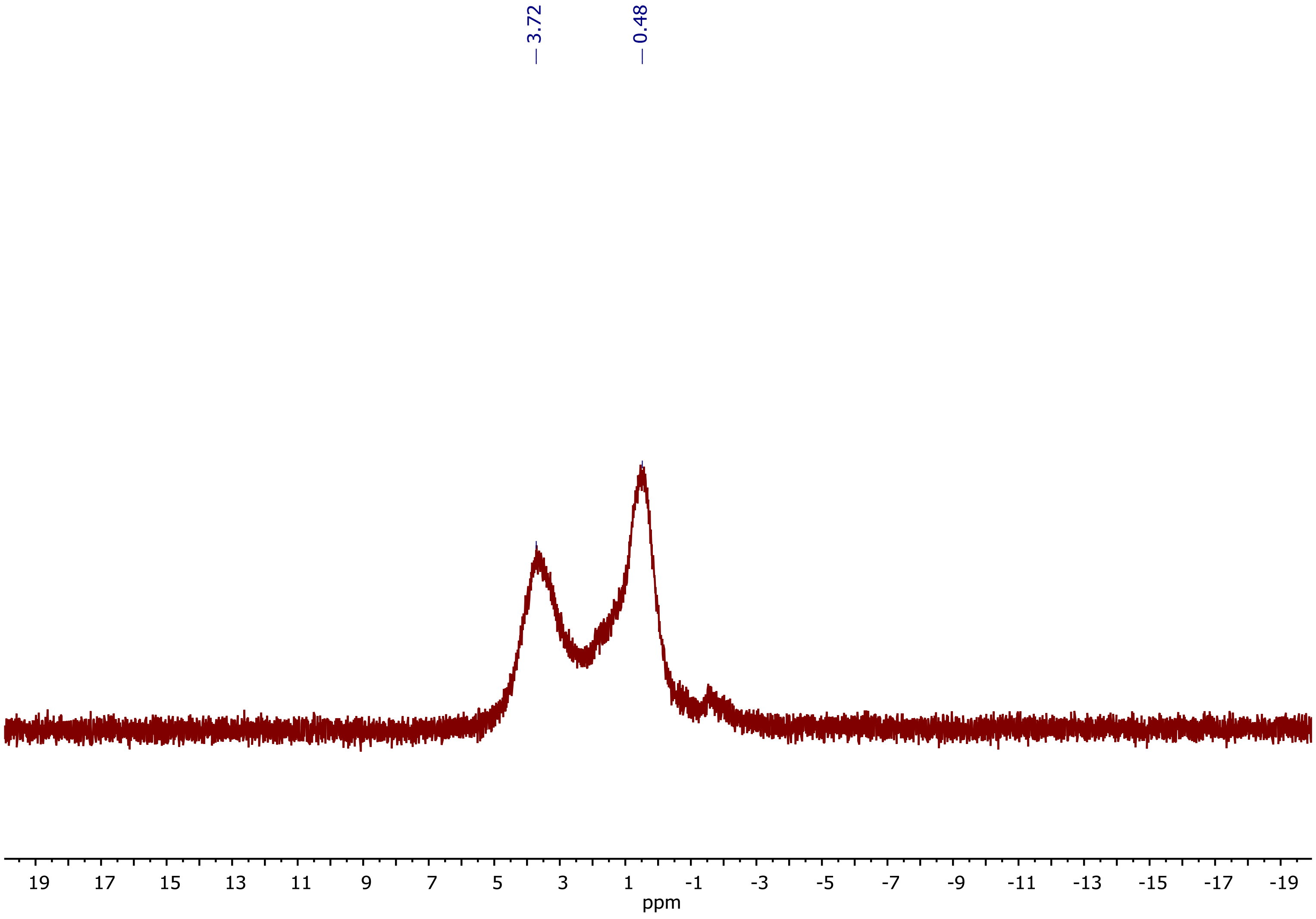

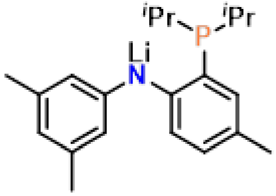

*Figure S 125: ^{7}Li NMR spectrum of **LiPN^{3,5Me}** in C_6D_6 at 298 K.*

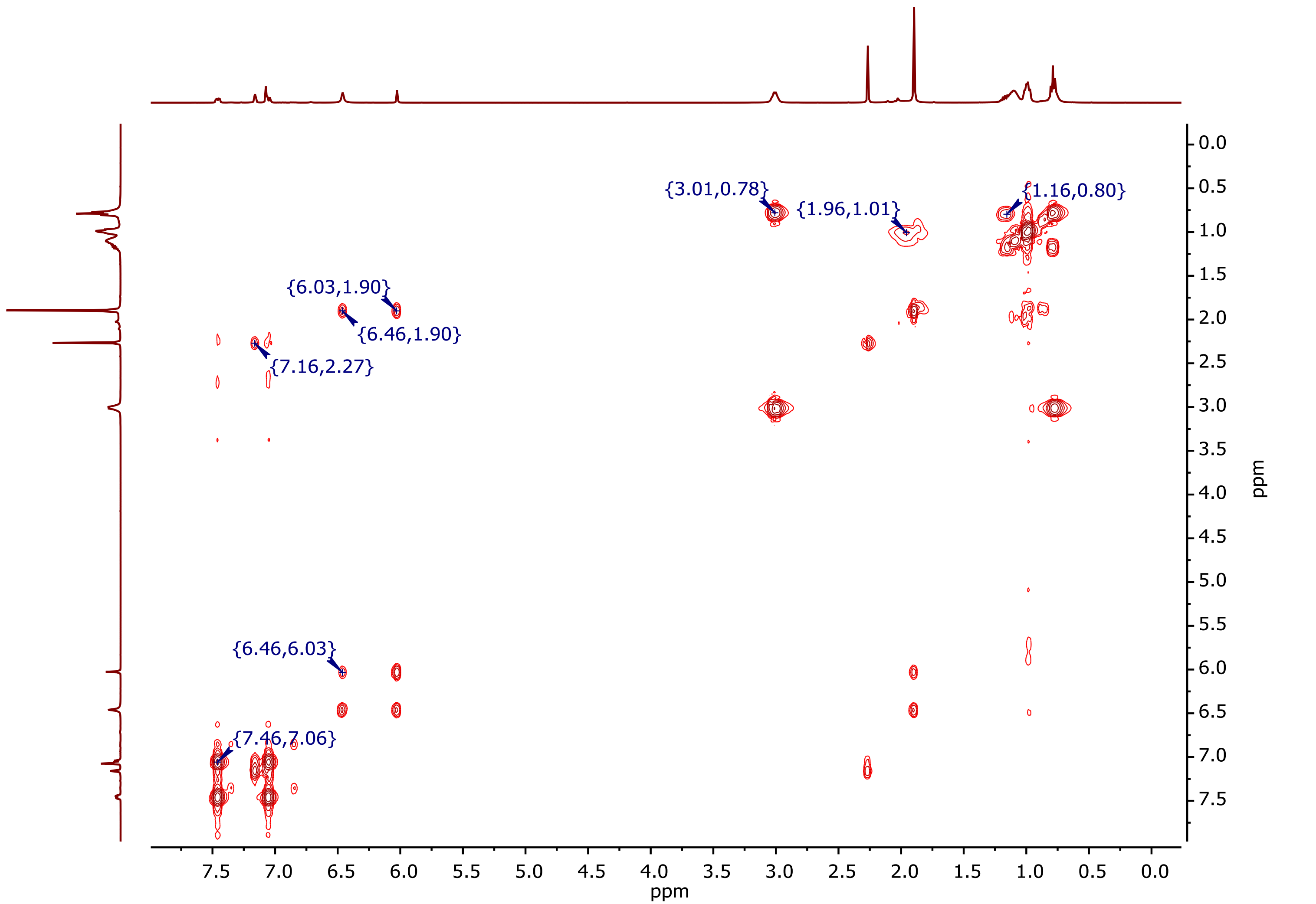

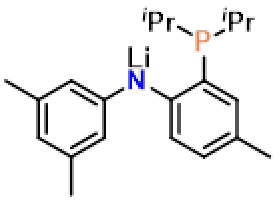

Figure S 126: ^{1}H-^{1}H COSY NMR spectrum of **LiPN**^{3,5Me} in C_6D_6 at 298 K.

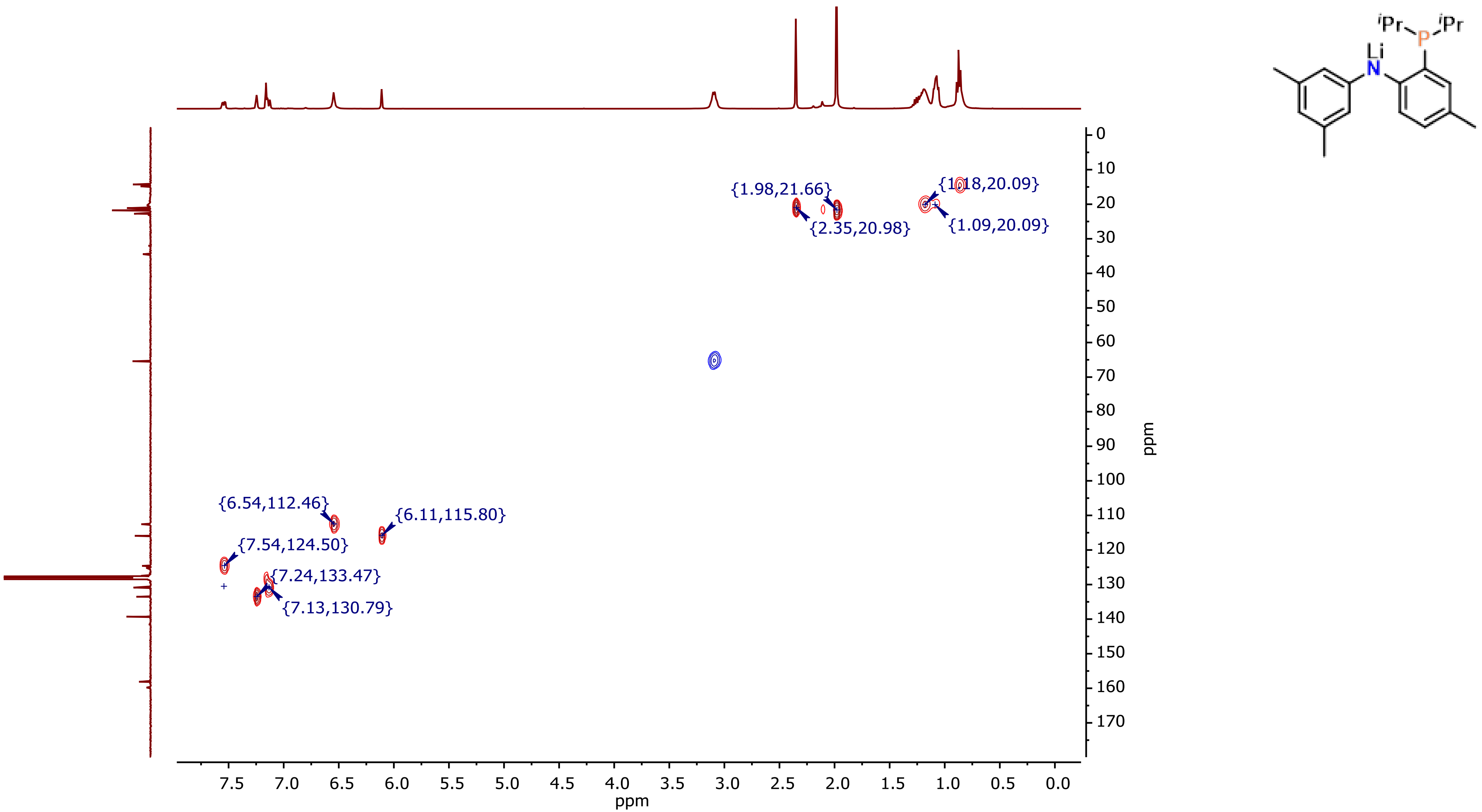

*Figure S 127: ^{1}H-^{13}C HSQC NMR spectrum of **LiPN^{3,5Me}** in C_6D_6 at 298 K.*

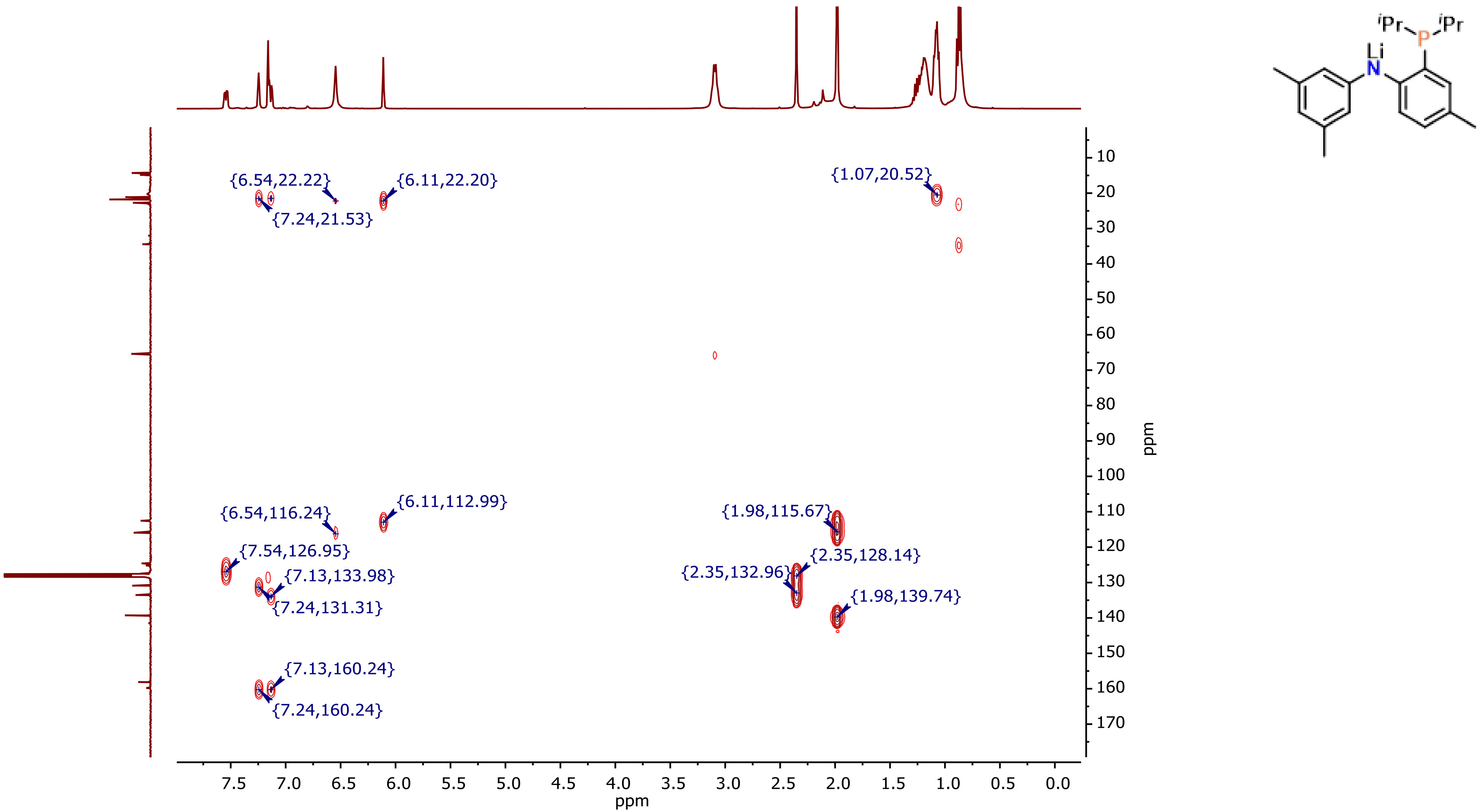

Figure S 128: ^{1}H-^{13}C HMBC NMR spectrum of **LiPN^{3,5Me}** in C_6D_6 at 298 K.

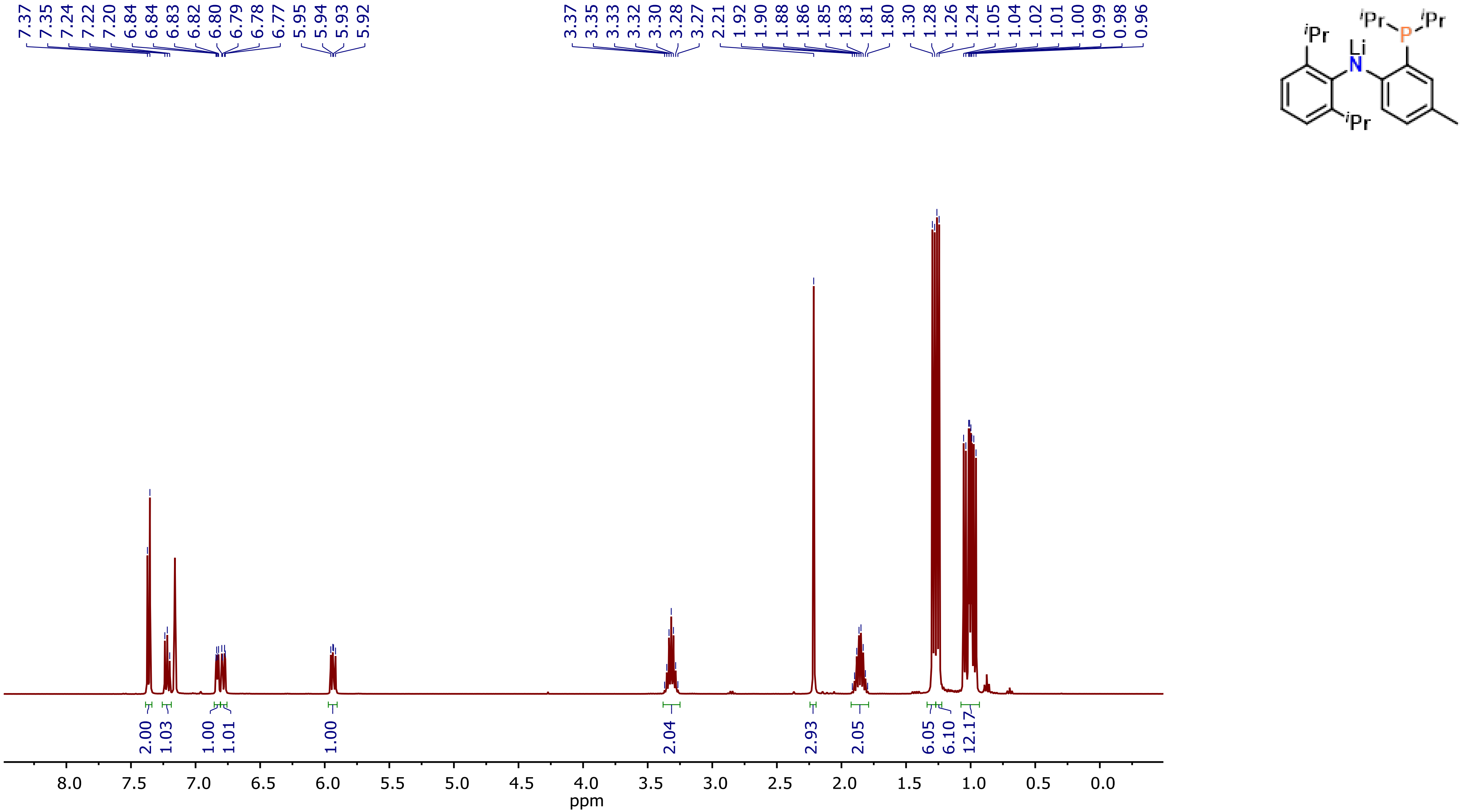

Figure S 129: ^{1}H NMR spectrum of ***LiPN^{Dipp}*** in C_6D_6 at 298 K.

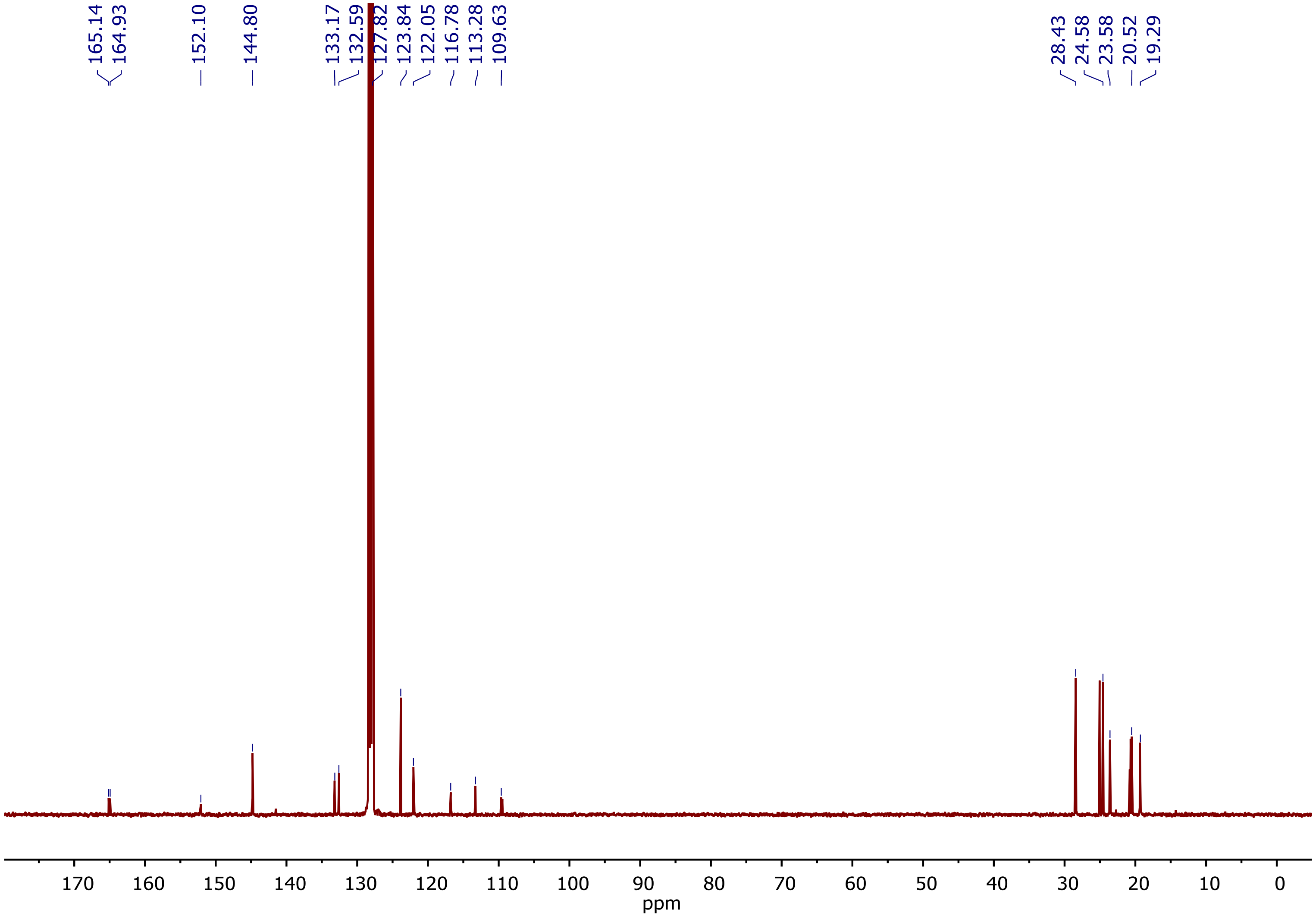

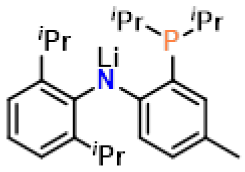

*Figure S 130: $^{13}C\{^{1}H\}$ NMR spectrum of **$LiPN^{Dipp}$** in C_6D_6 at 298 K.*

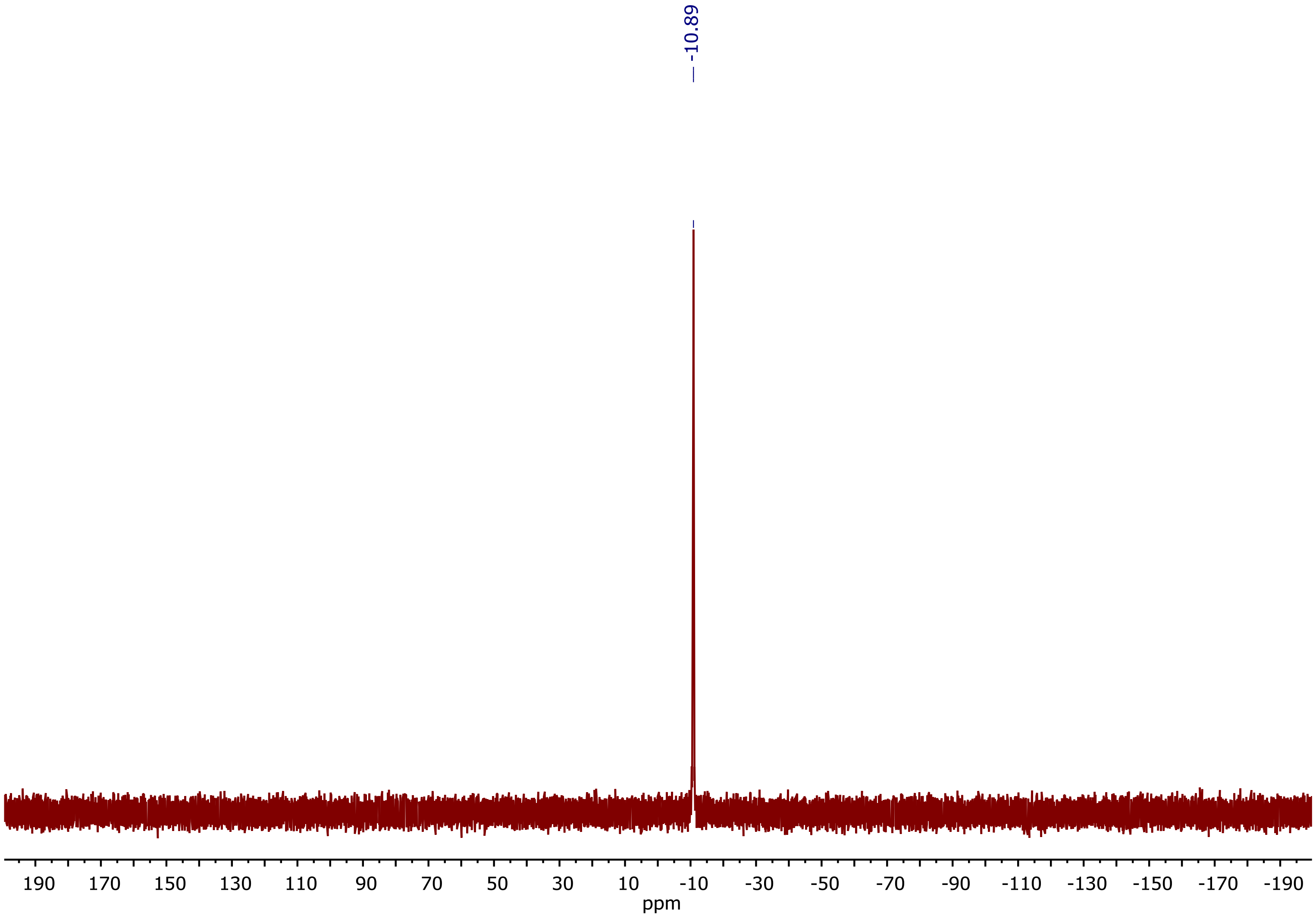

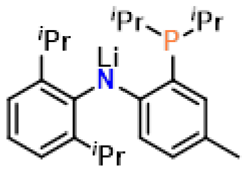

*Figure S 131: $^{31}P\{^{1}H\}$ NMR spectrum of **LiPN^{Dipp}** in C_6D_6 at 298 K.*

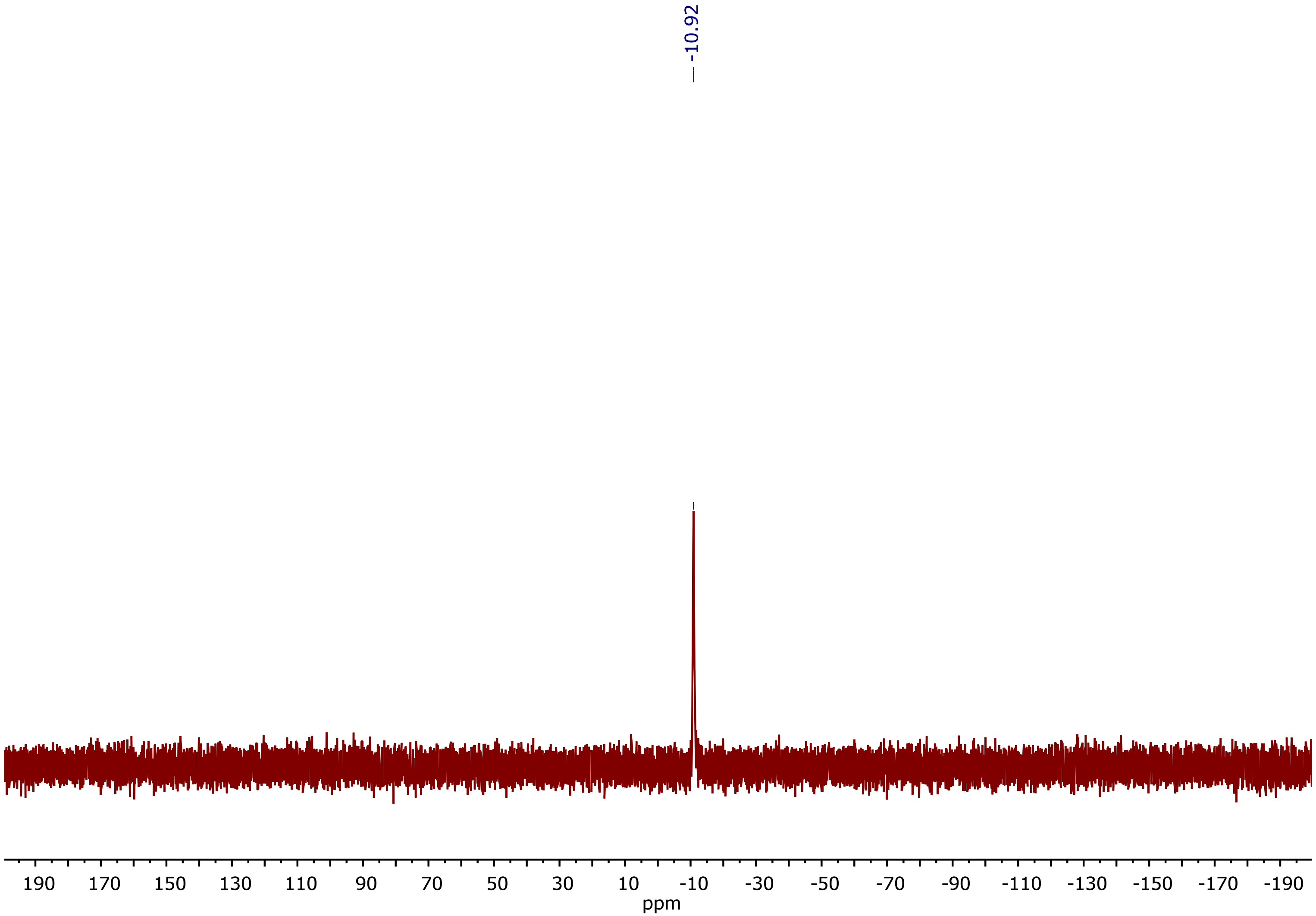

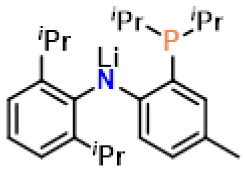

*Figure S 132: ^{31}P NMR spectrum of **LiPN^{Dipp}** in C_6D_6 at 298 K.*

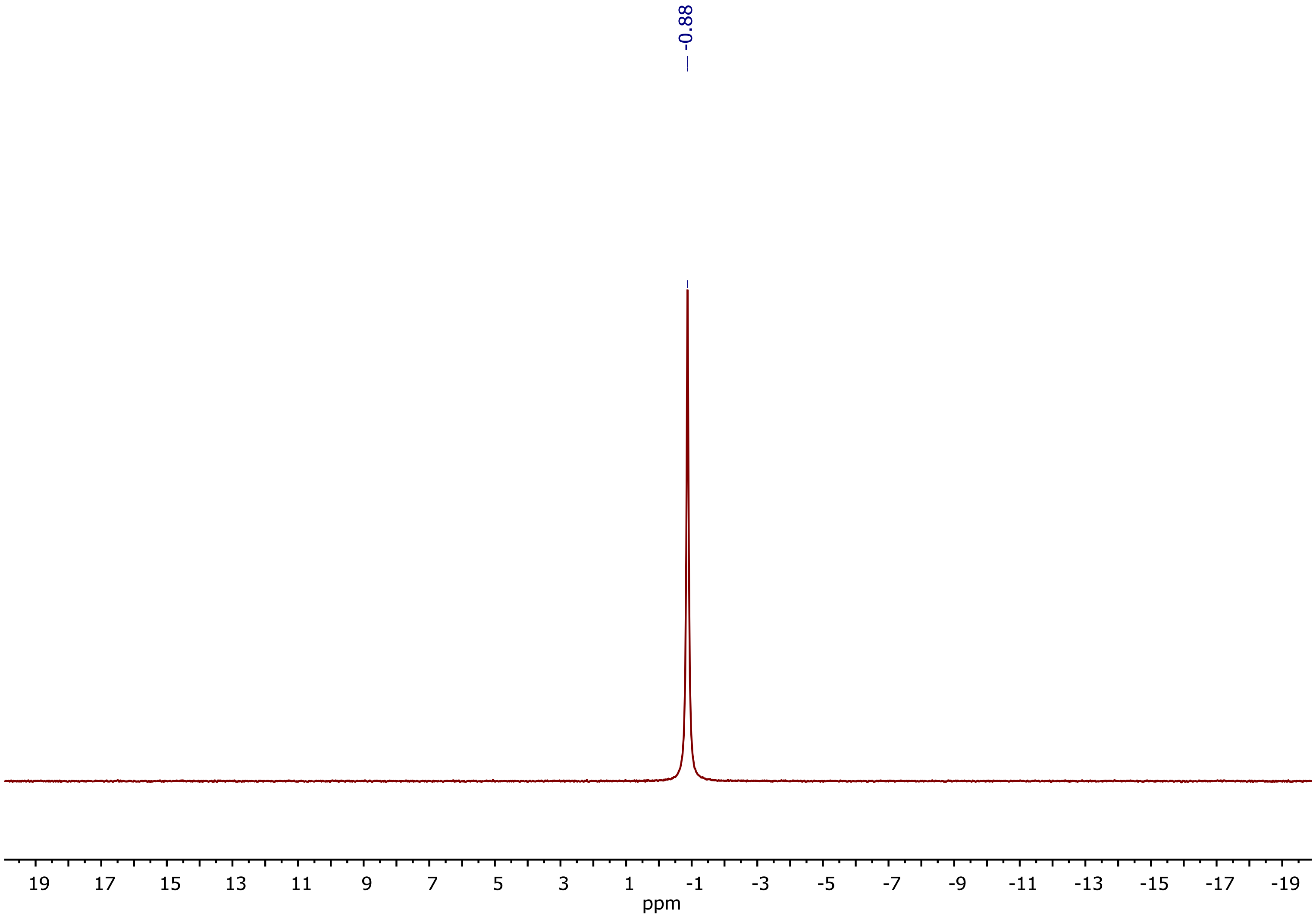

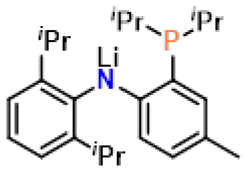

*Figure S 133: $^{7}Li\{^{1}H\}$ NMR spectrum of **LiPN^{Dipp}** in C_6D_6 at 298 K.*

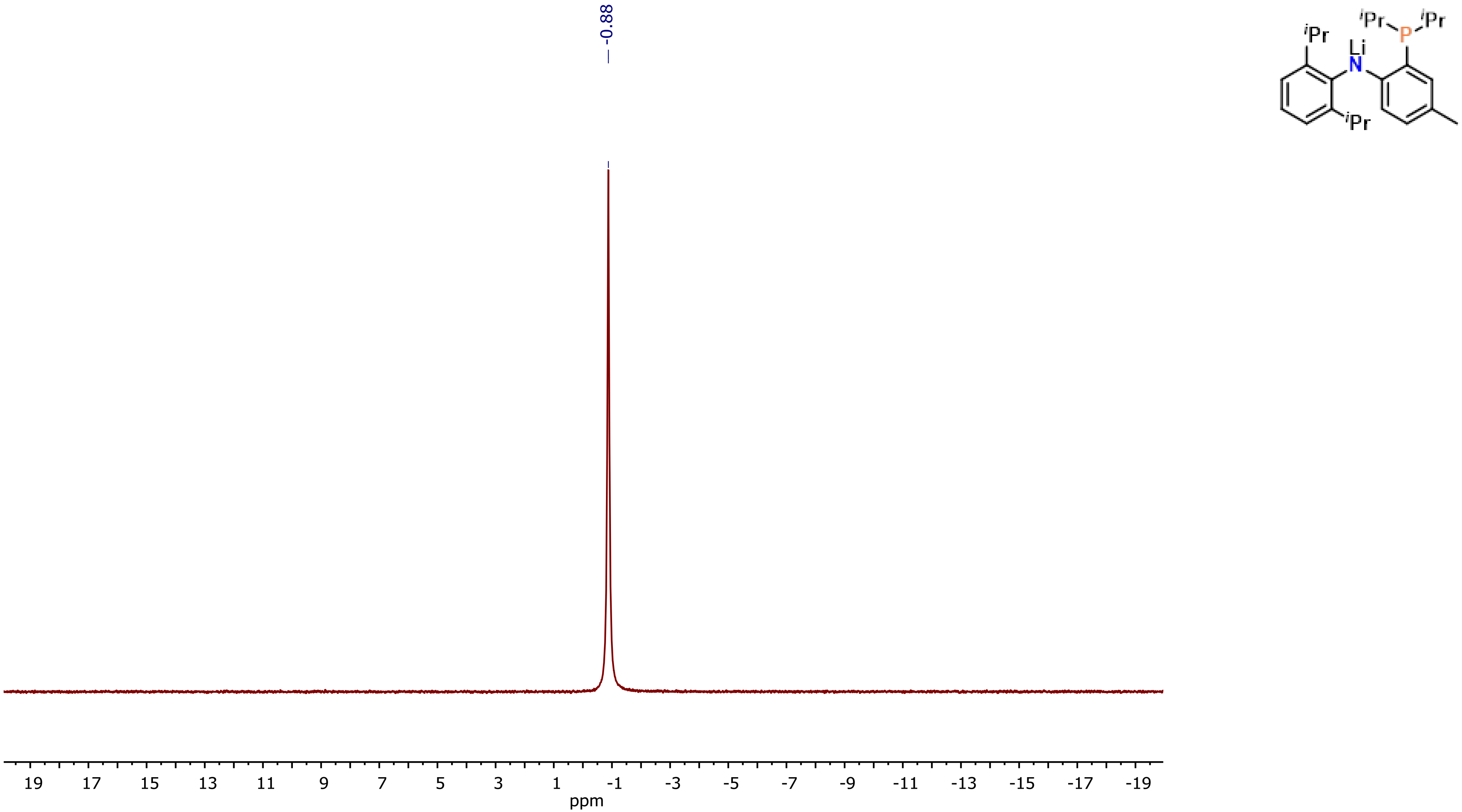

*Figure S 134: ^{7}Li NMR spectrum of **LiPN^{Dipp}** in C_6D_6 at 298 K.*

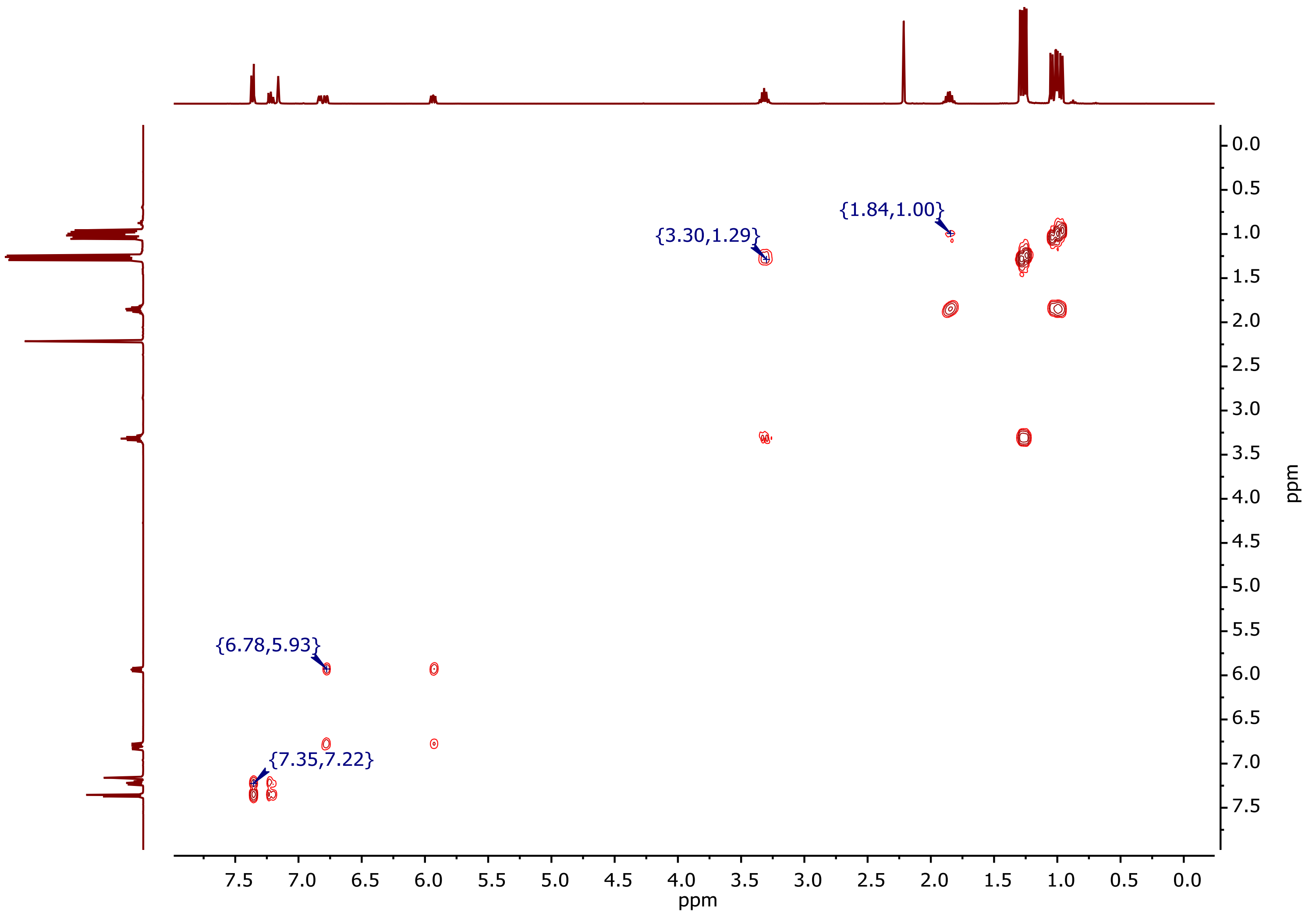

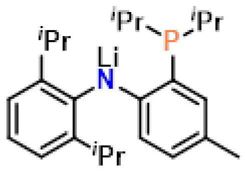

Figure S 135: ^{1}H-^{1}H COSY NMR spectrum of ***LiPN^{Dipp}*** *in C_6D_6 at 298 K.*

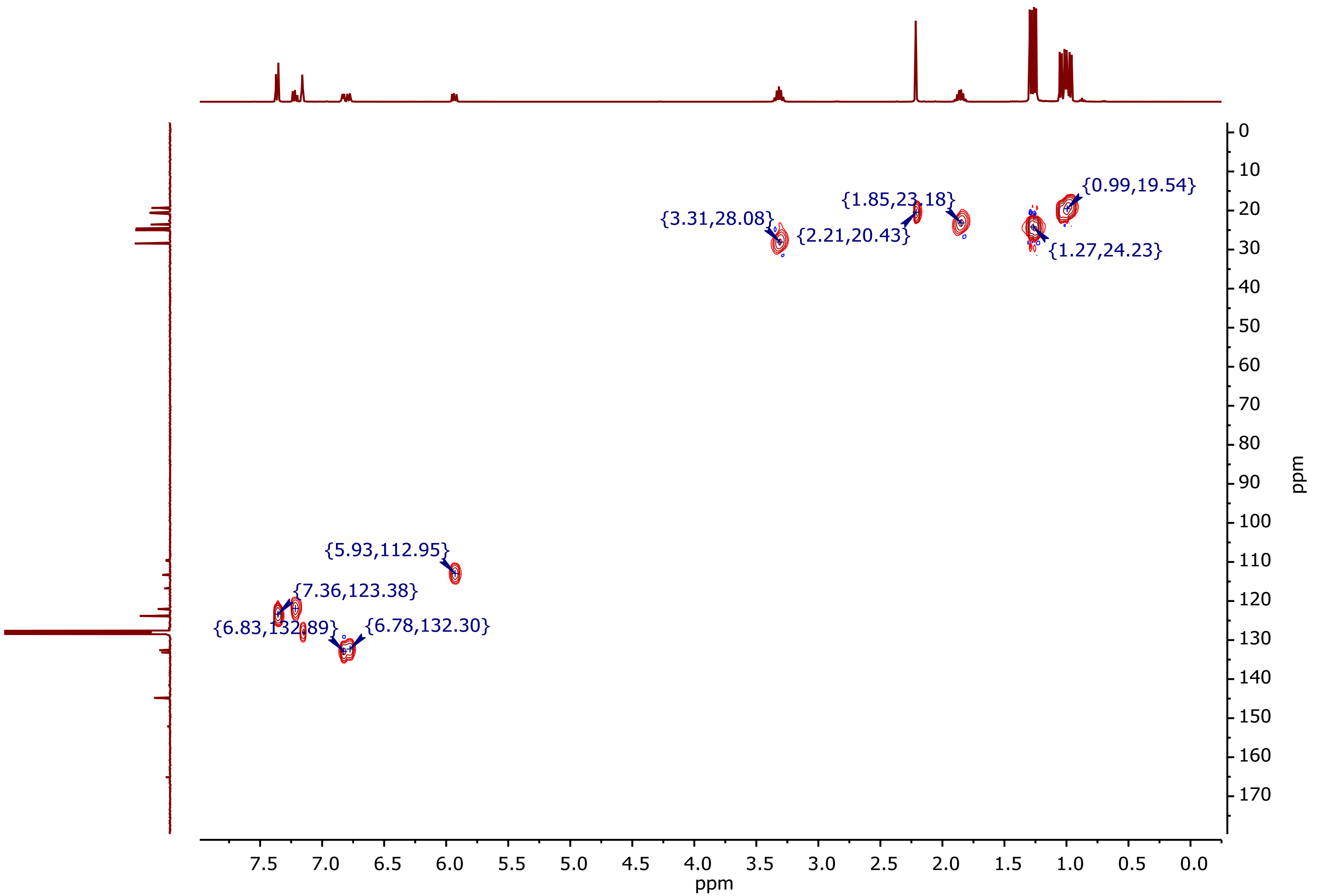

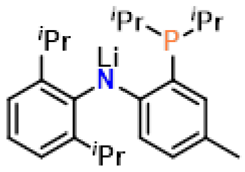

Figure S 136: ^{1}H-^{13}C HSQC NMR spectrum of **LiPN^{Dipp}** in C_6D_6 at 298 K.

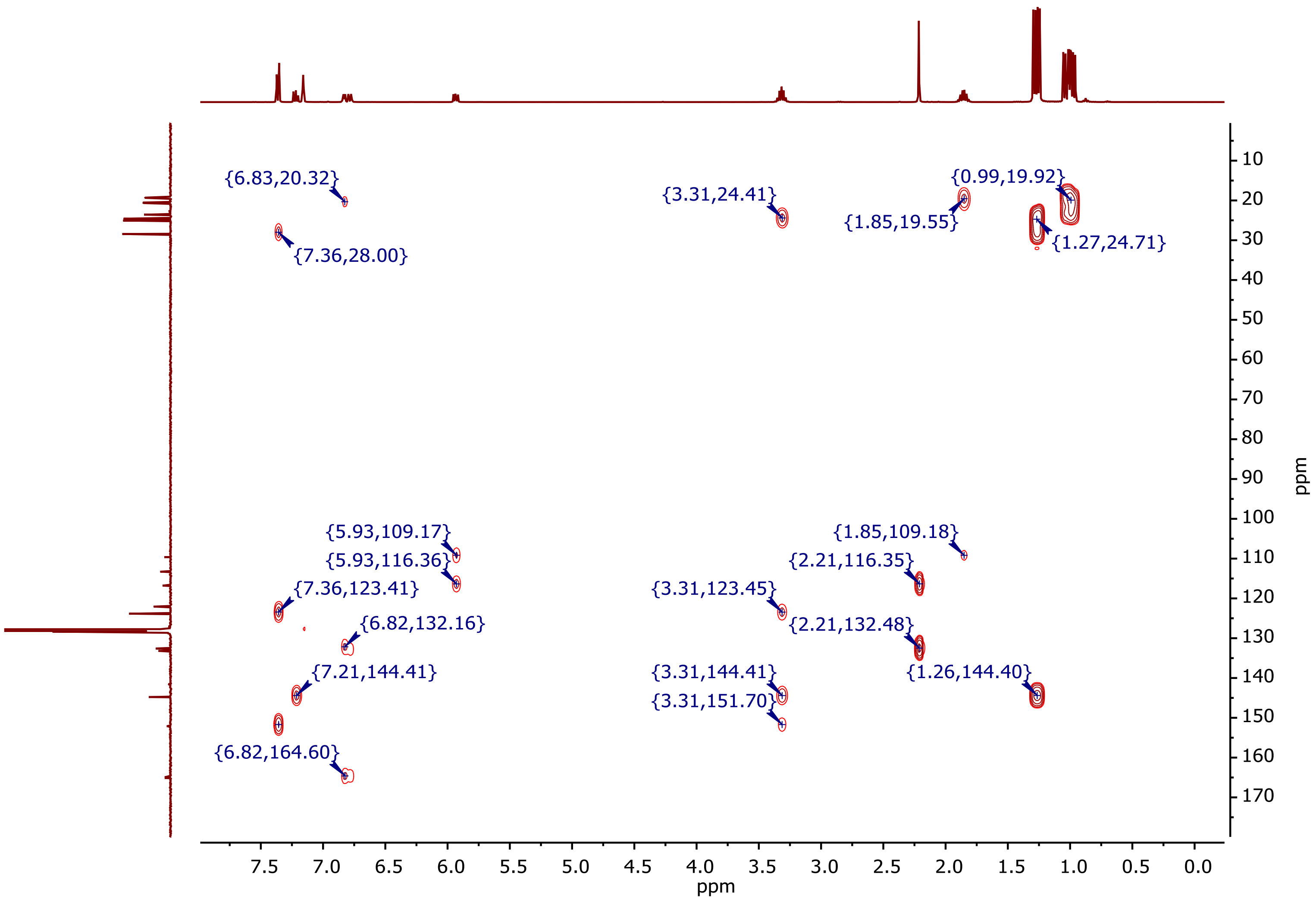

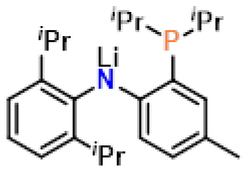

*Figure S 137: ^{1}H-^{13}C HMBC NMR spectrum of **LiPN^{Dipp}** in C_6D_6 at 298 K.*

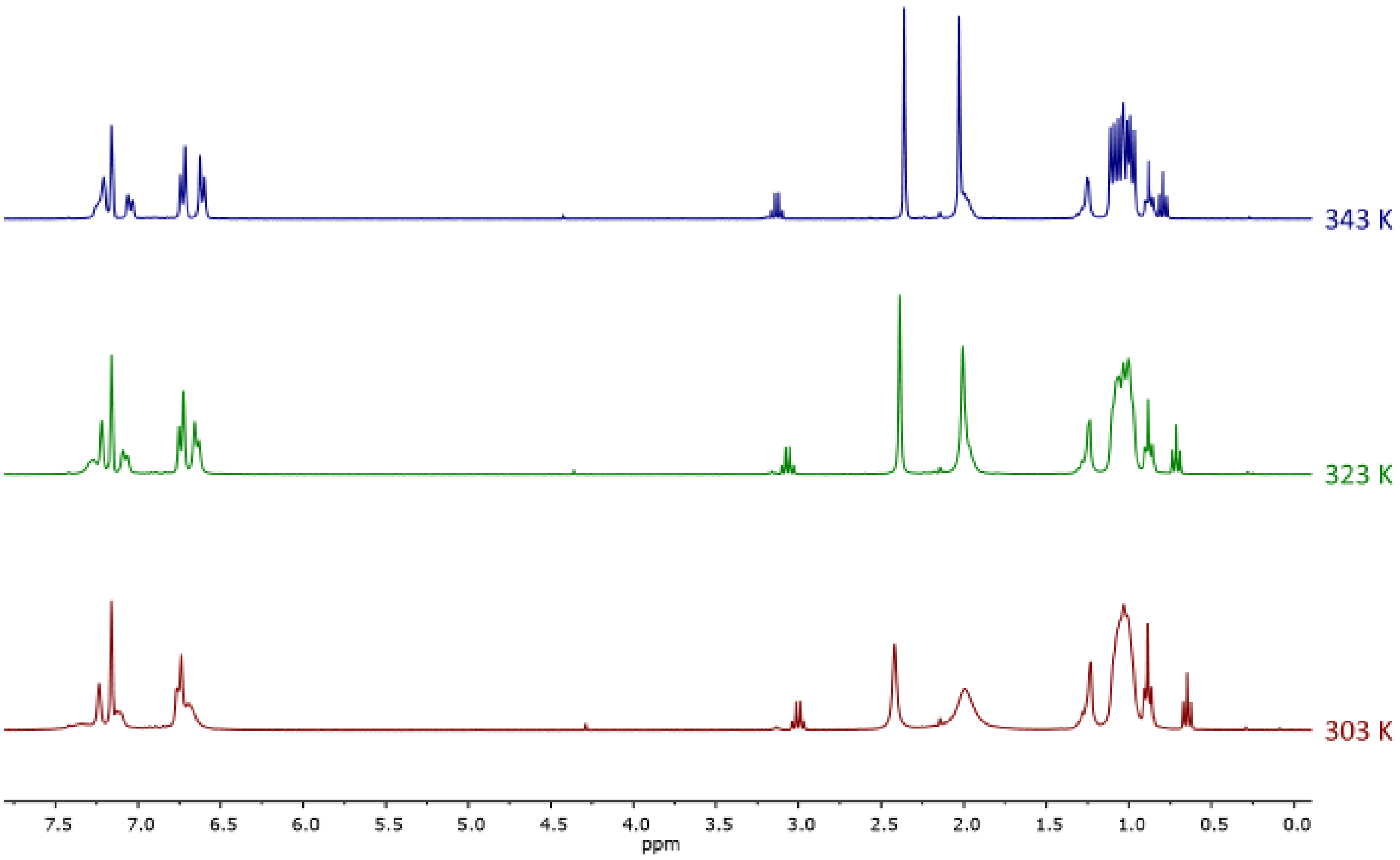

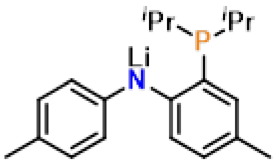

Figure S 138: VT ^{1}H NMR spectrum of ***LiPN^{Tol}*** *in C_6D_6 at 303 K (red), 323 K (green) and 343 K (blue).*

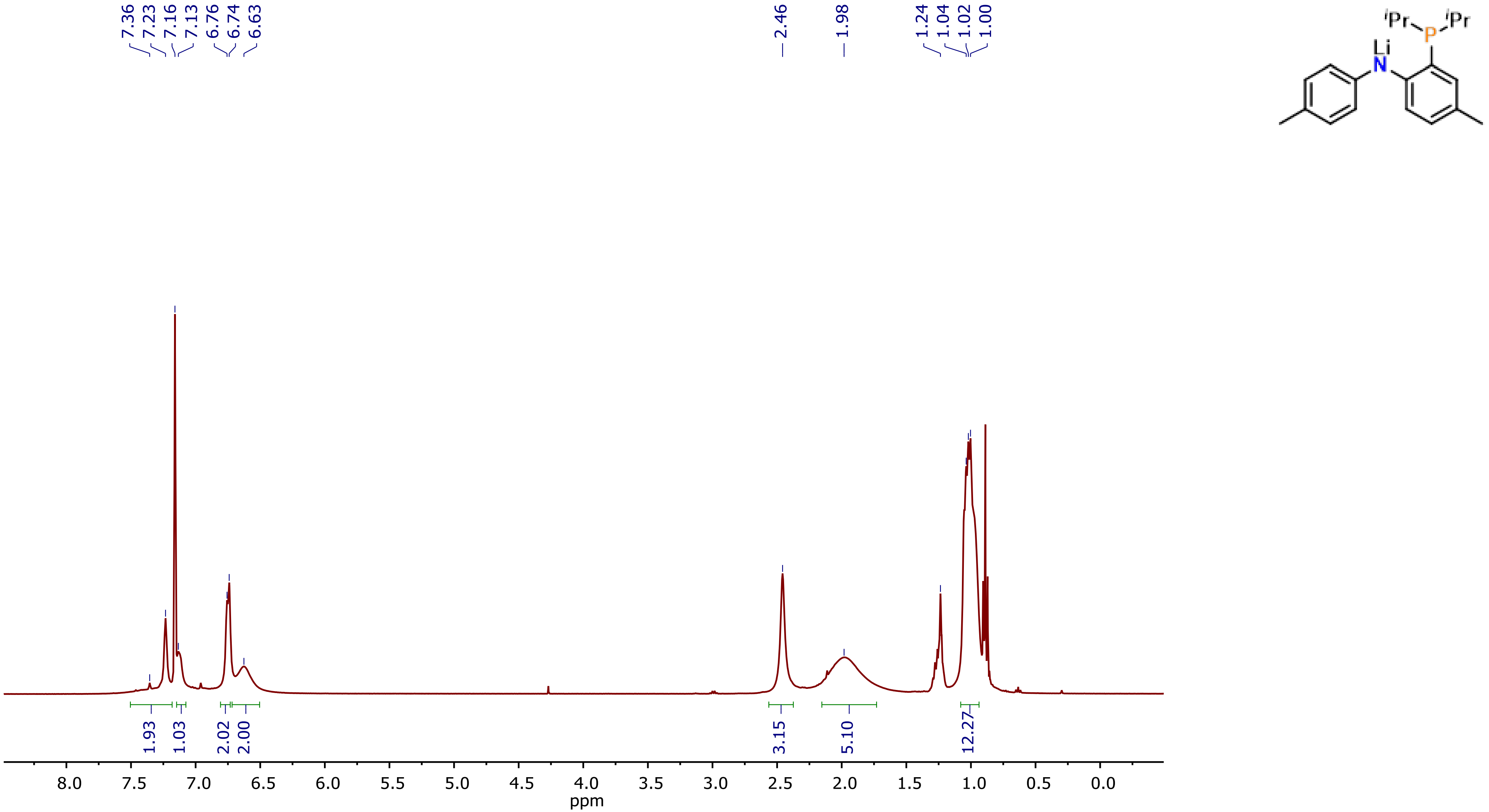

*Figure S 139: ^{1}H NMR spectrum of **LiPN^{Tol}** in C_6D_6 at 298 K.*

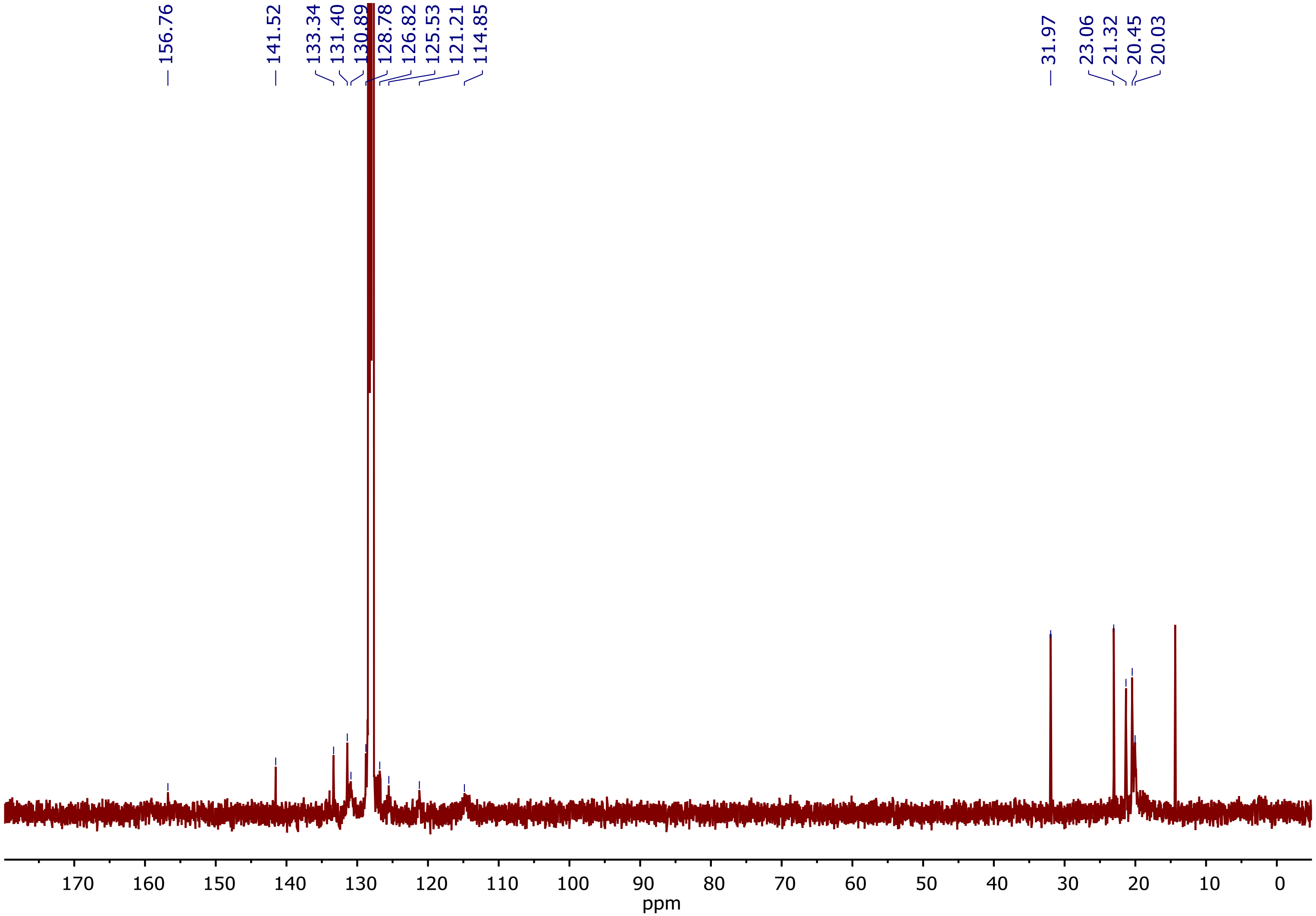

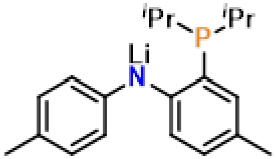

*Figure S 140: $^{13}C\{^{1}H\}$ NMR spectrum of **LiPN^{Tol}** in C_6D_6 at 298 K.*

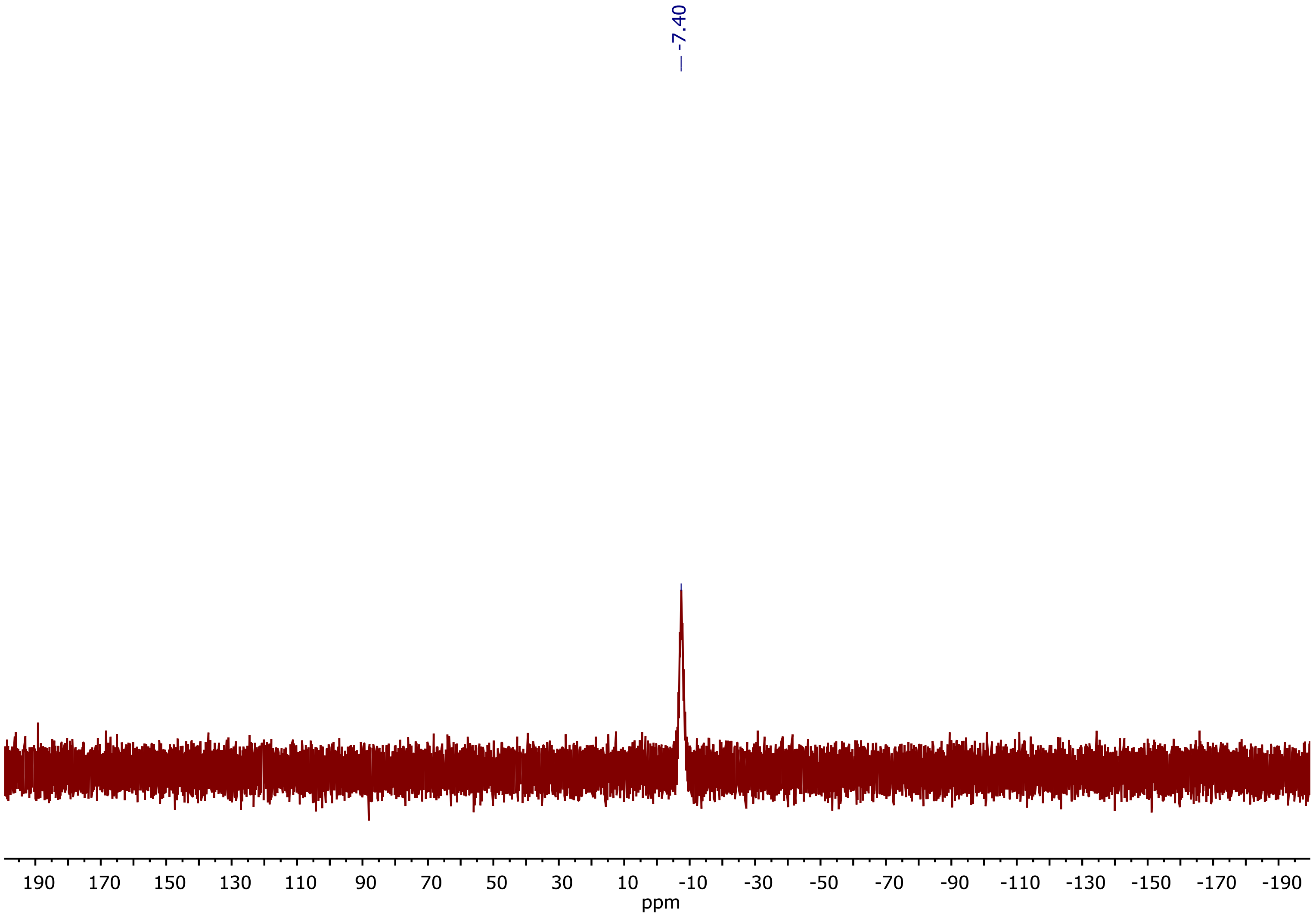

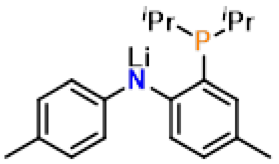

*Figure S 141: $^{31}P\{^{1}H\}$ NMR spectrum of **$LiPN^{Tol}$** in C_6D_6 at 298 K.*

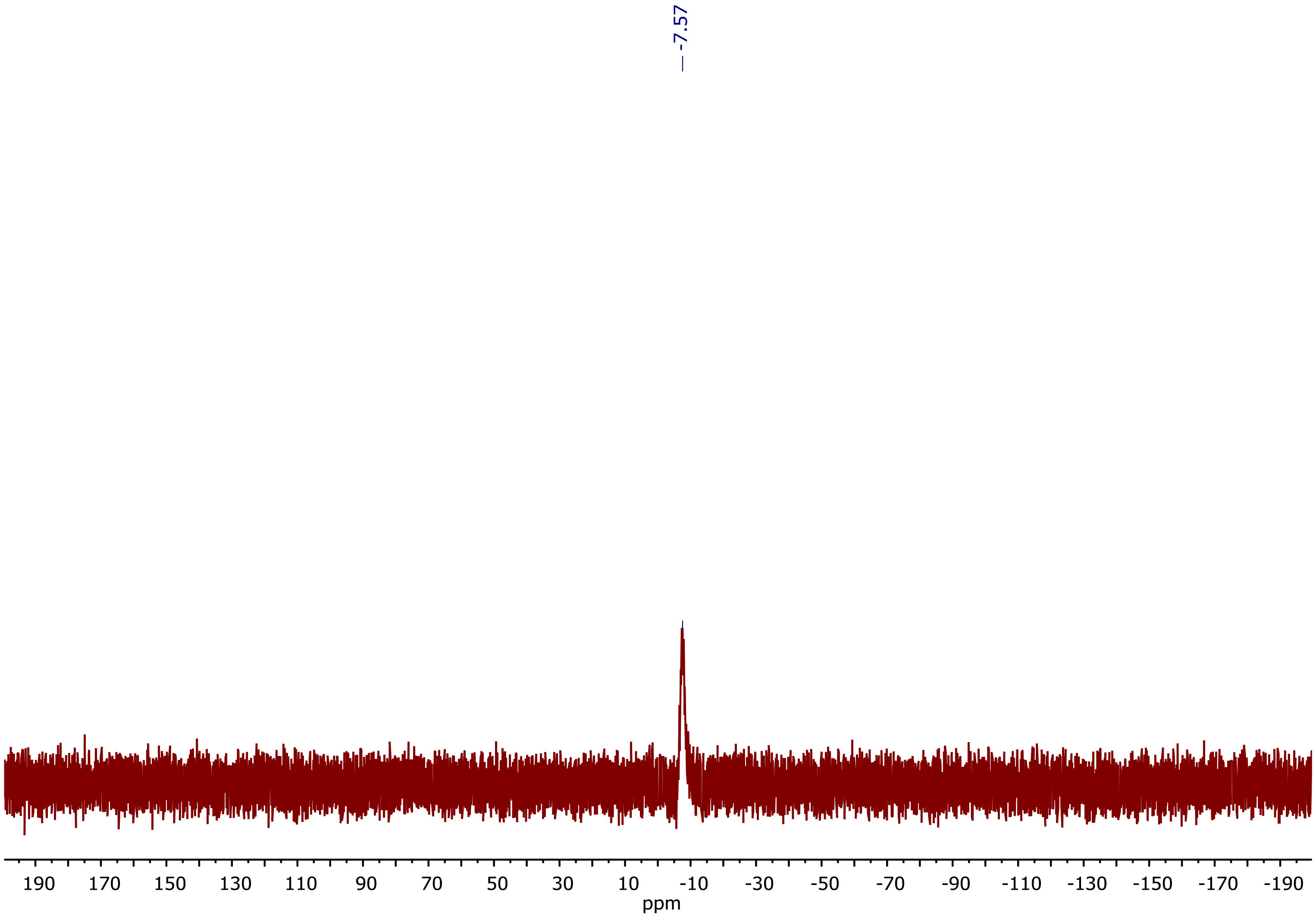

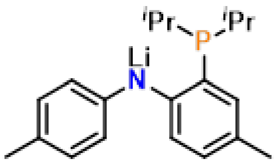

*Figure S 142: ^{31}P NMR spectrum of **LiPN^{Tol}** in C_6D_6 at 298 K.*

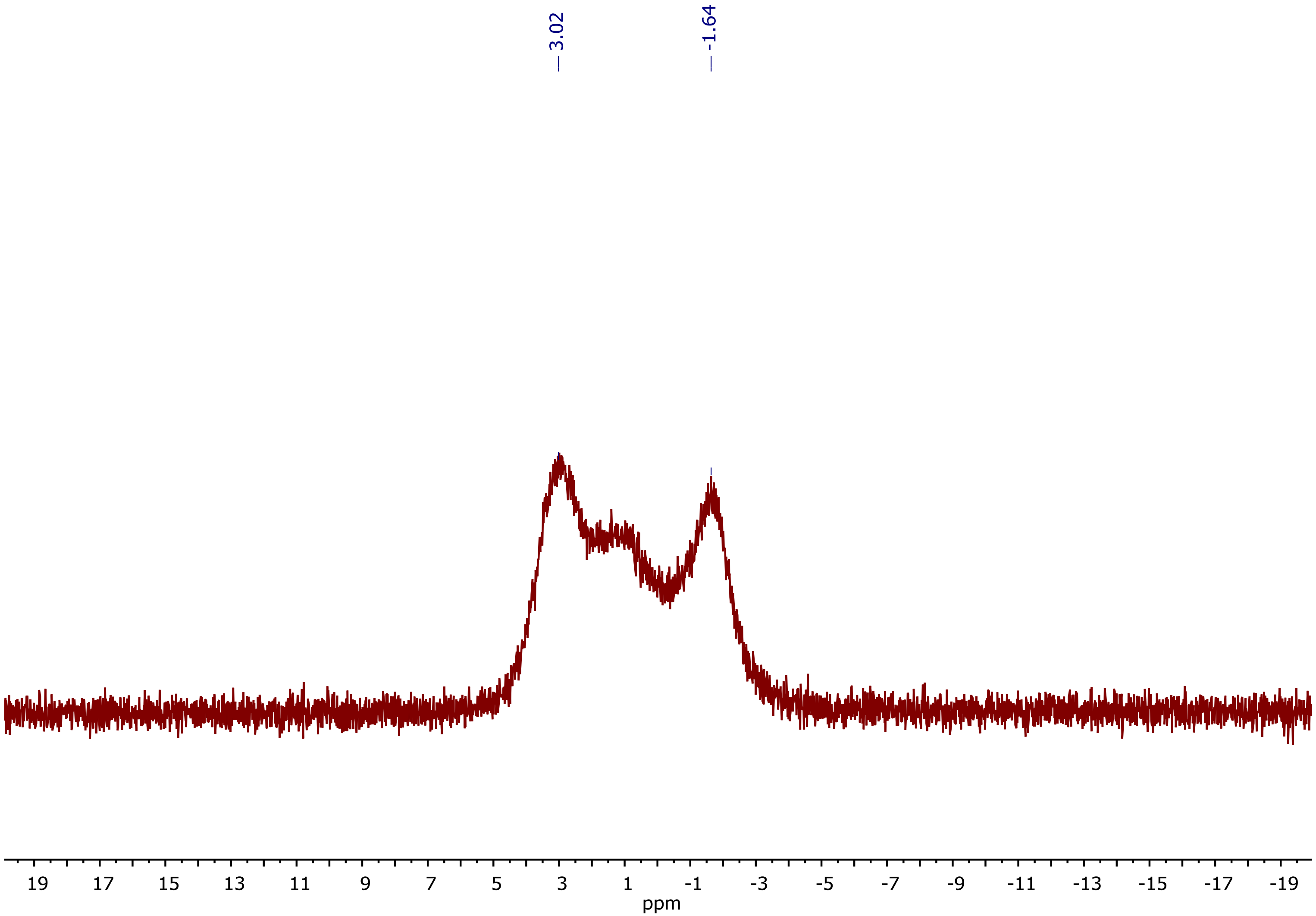

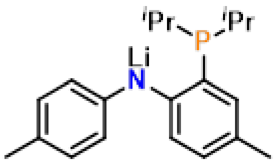

*Figure S 143: $^{7}Li\{^{1}H\}$ NMR spectrum of **LiPN^{Tol}** in C_6D_6 at 298 K.*

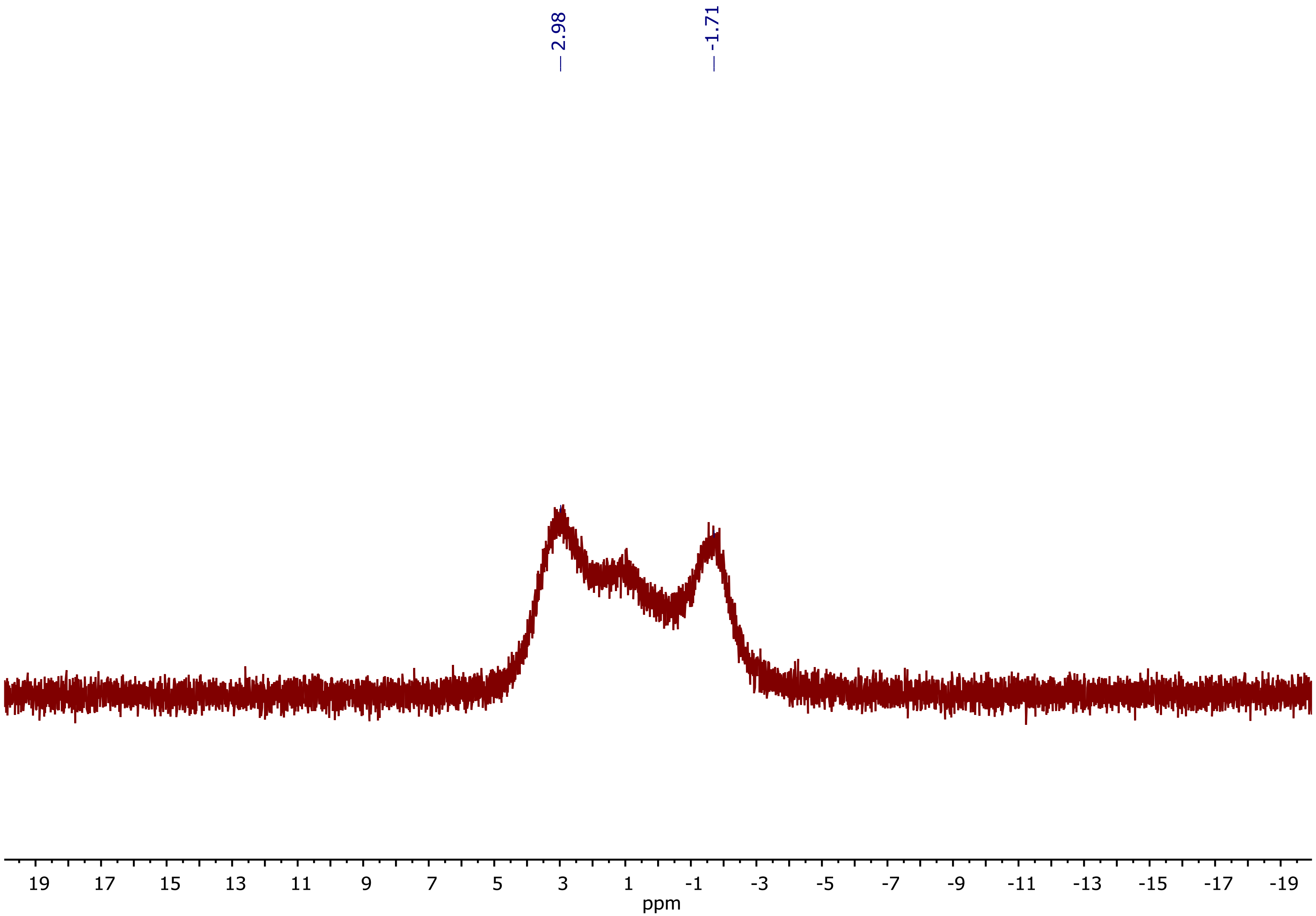

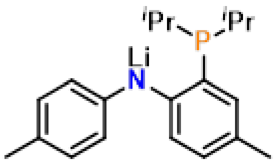

*Figure S 144: ^{7}Li NMR spectrum of **LiPN^{Tol}** in C_6D_6 at 298 K.*

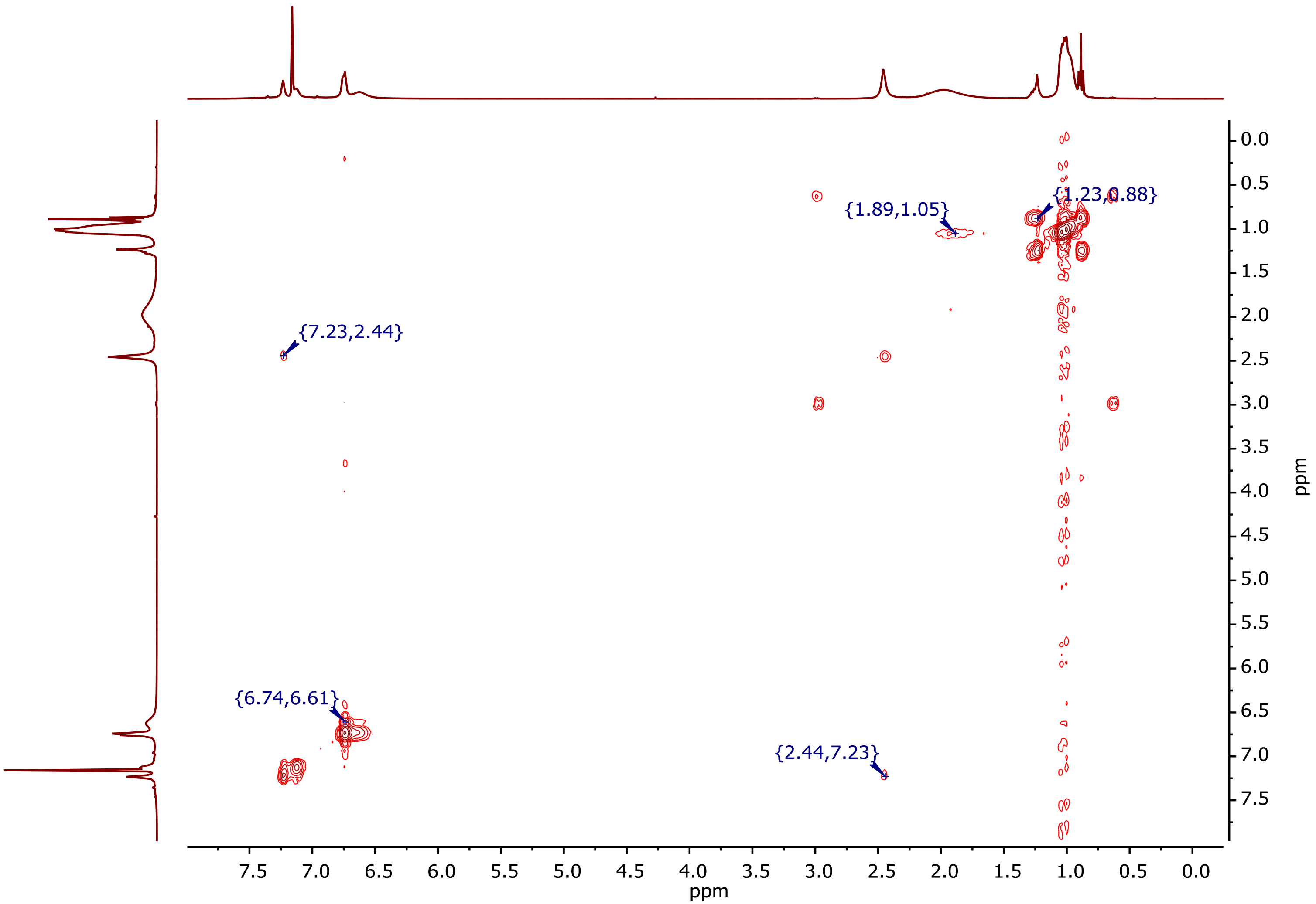

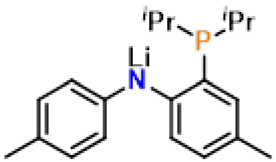

*Figure S 145: ^{1}H-^{1}H COSY NMR spectrum of **LiPN^{Tol}** in C_6D_6 at 298 K.*

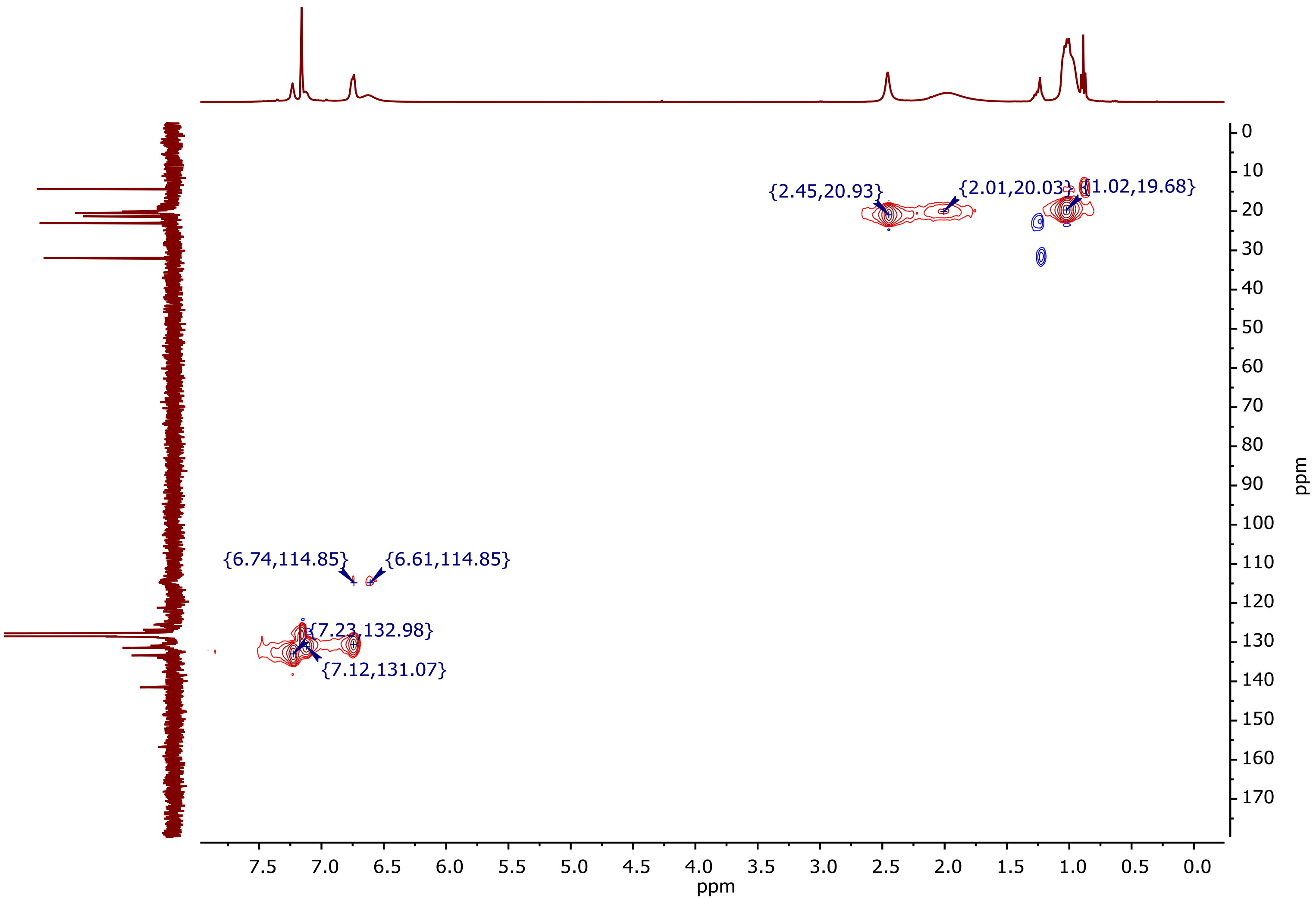

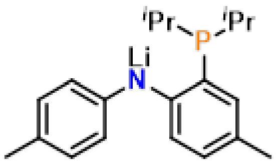

Figure S 146: ^{1}H-^{13}C HSQC NMR spectrum of **LiPN^{Tol}** in C_6D_6 at 298 K.

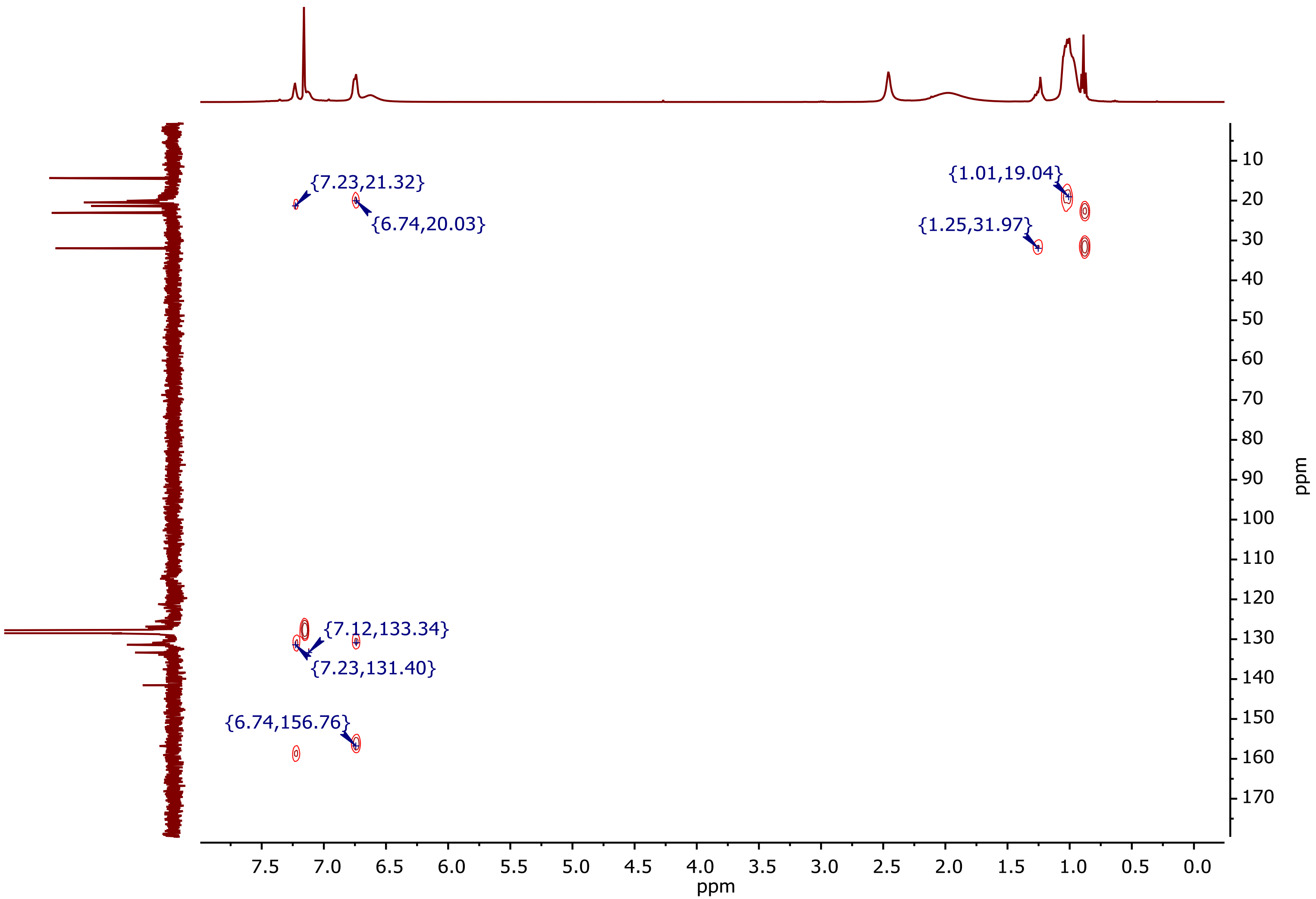

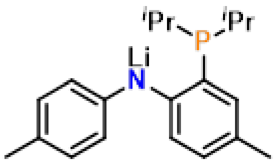

*Figure S 147: ^{1}H-^{13}C HMBC NMR spectrum of **LiPN^{Tol}** in C_6D_6 at 298 K.*

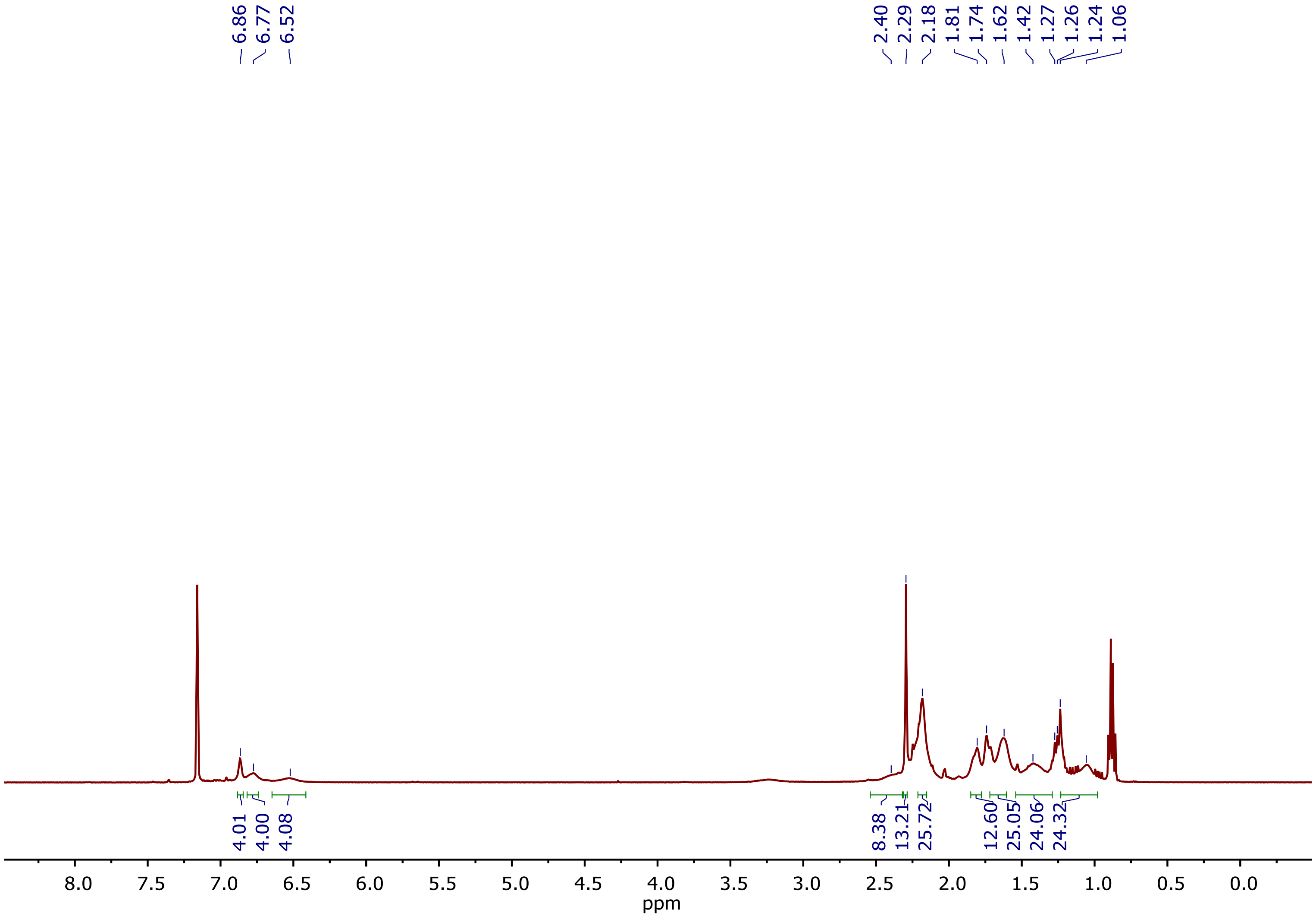

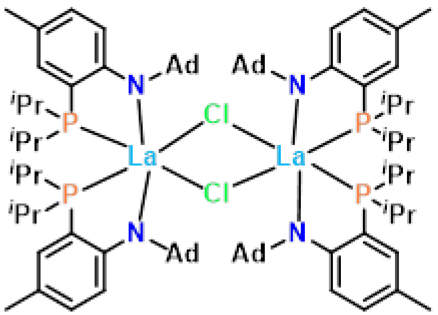

*Figure S 148: ^{1}H NMR spectrum of **3a** in C_6D_6 at 298 K.*

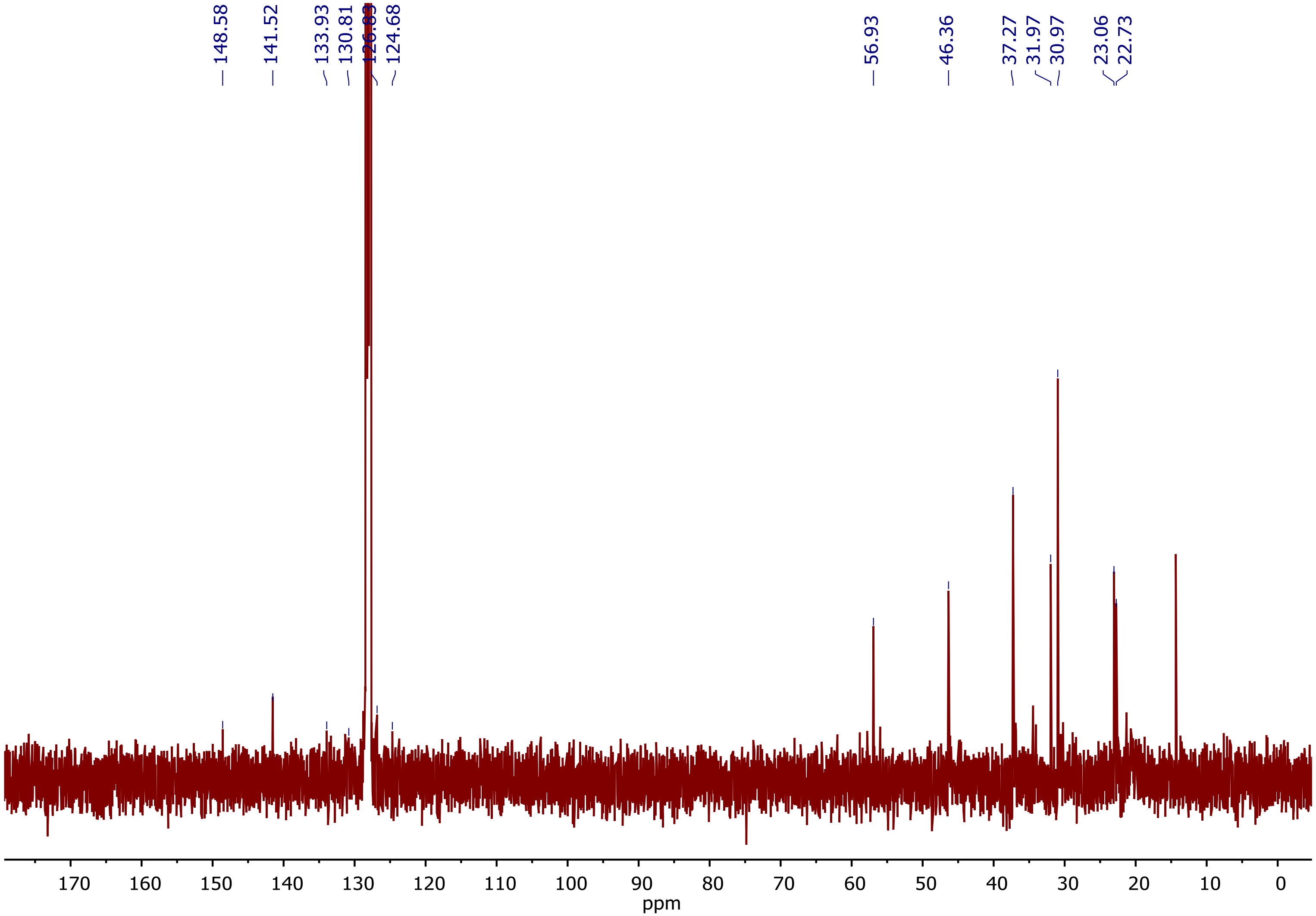

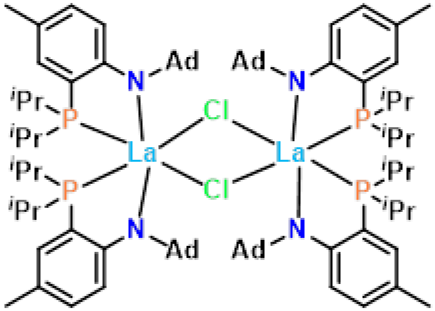

*Figure S 149: $^{13}C\{^{1}H\}$ NMR spectrum of **3a** in C_6D_6 at 298 K.*

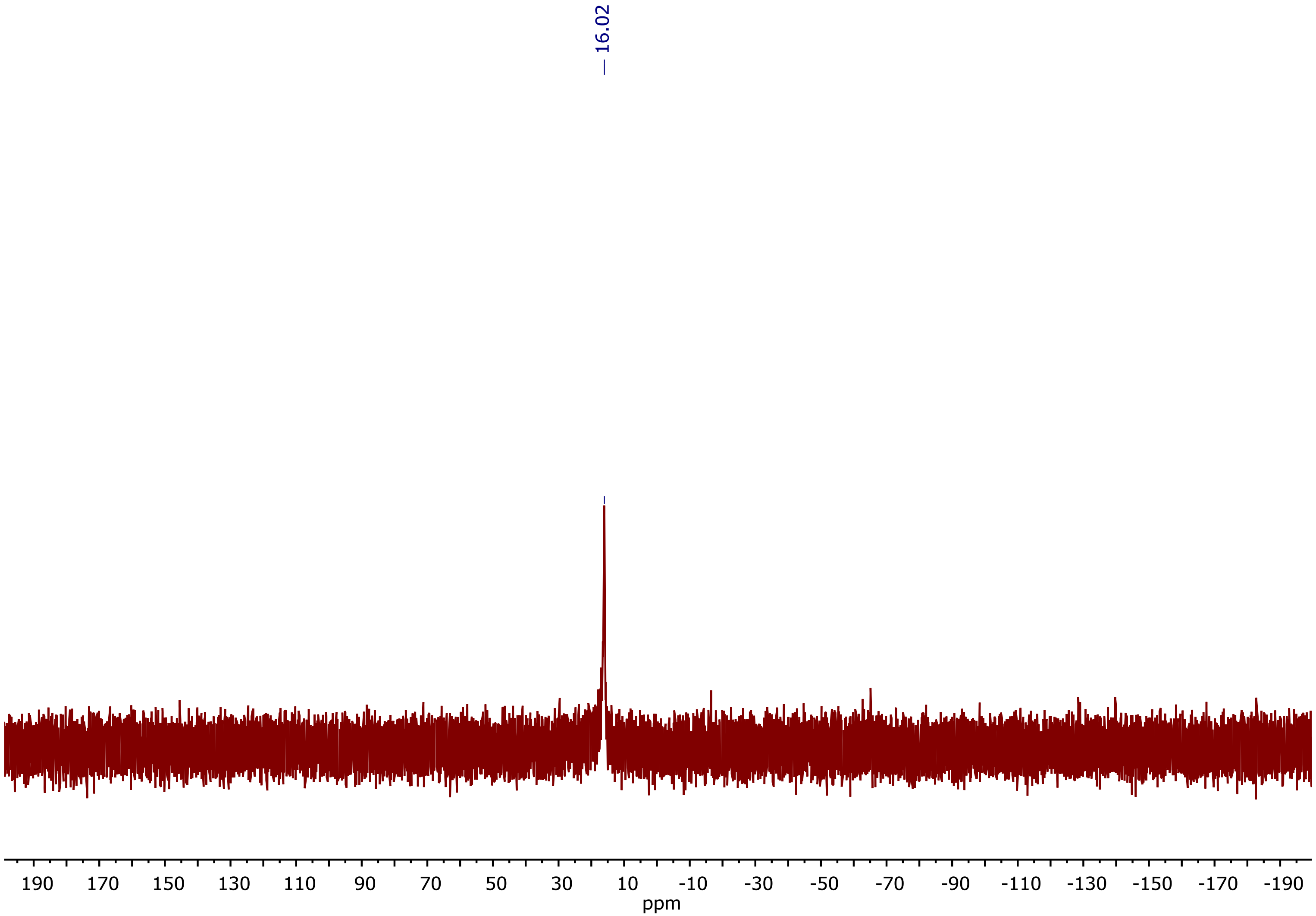

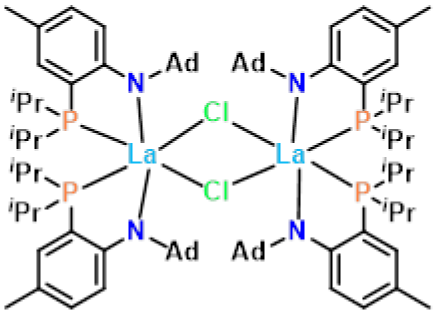

*Figure S 150: $^{31}P\{^{1}H\}$ NMR spectrum of **3a** in C_6D_6 at 298 K.*

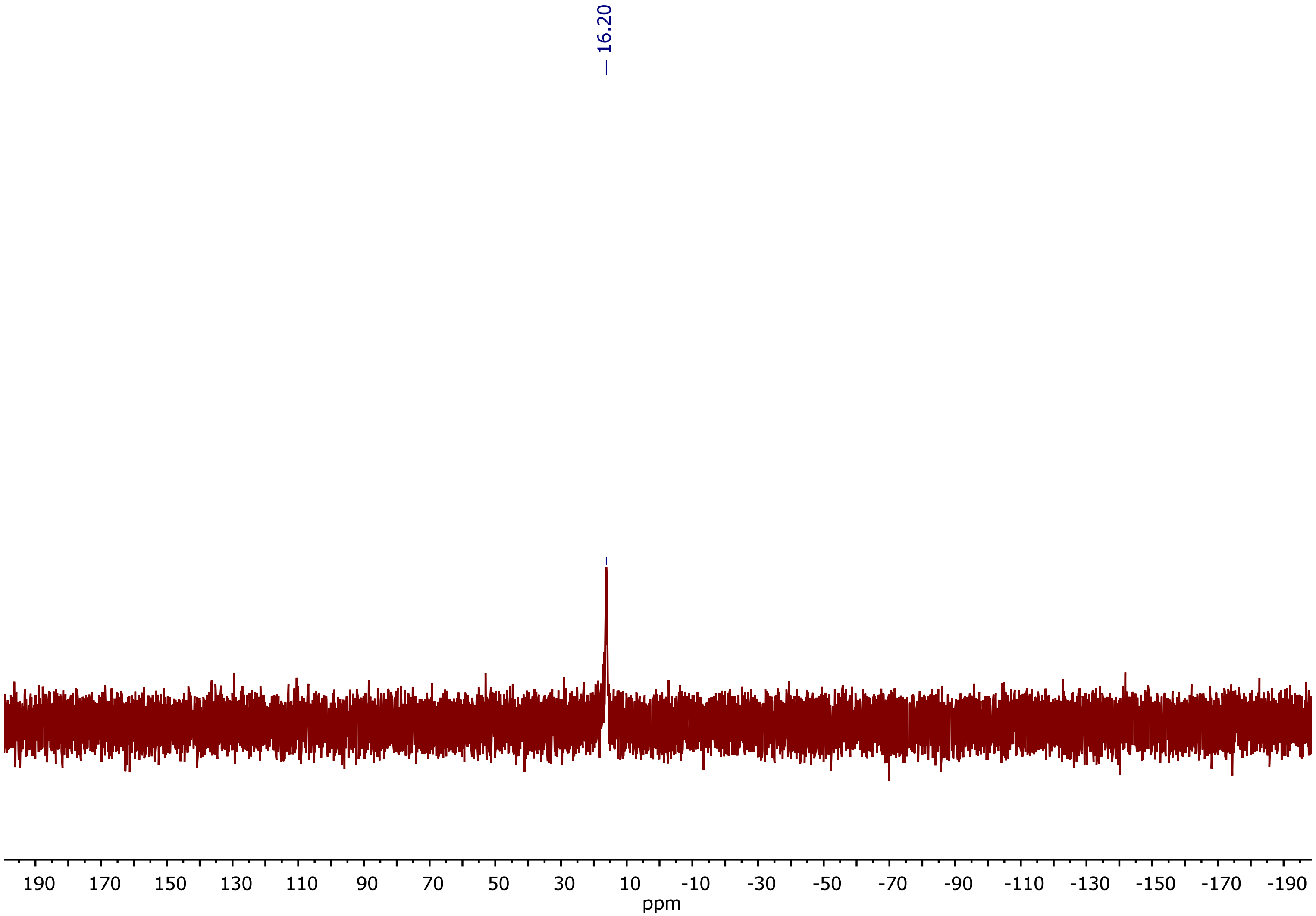

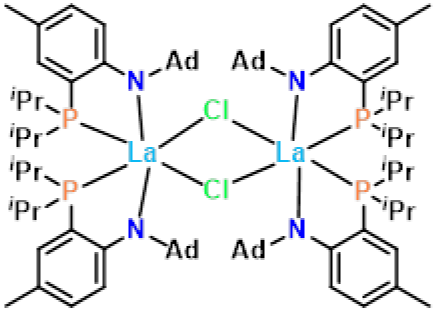

*Figure S 151: ^{31}P NMR spectrum of **3a** in C_6D_6 at 298 K.*

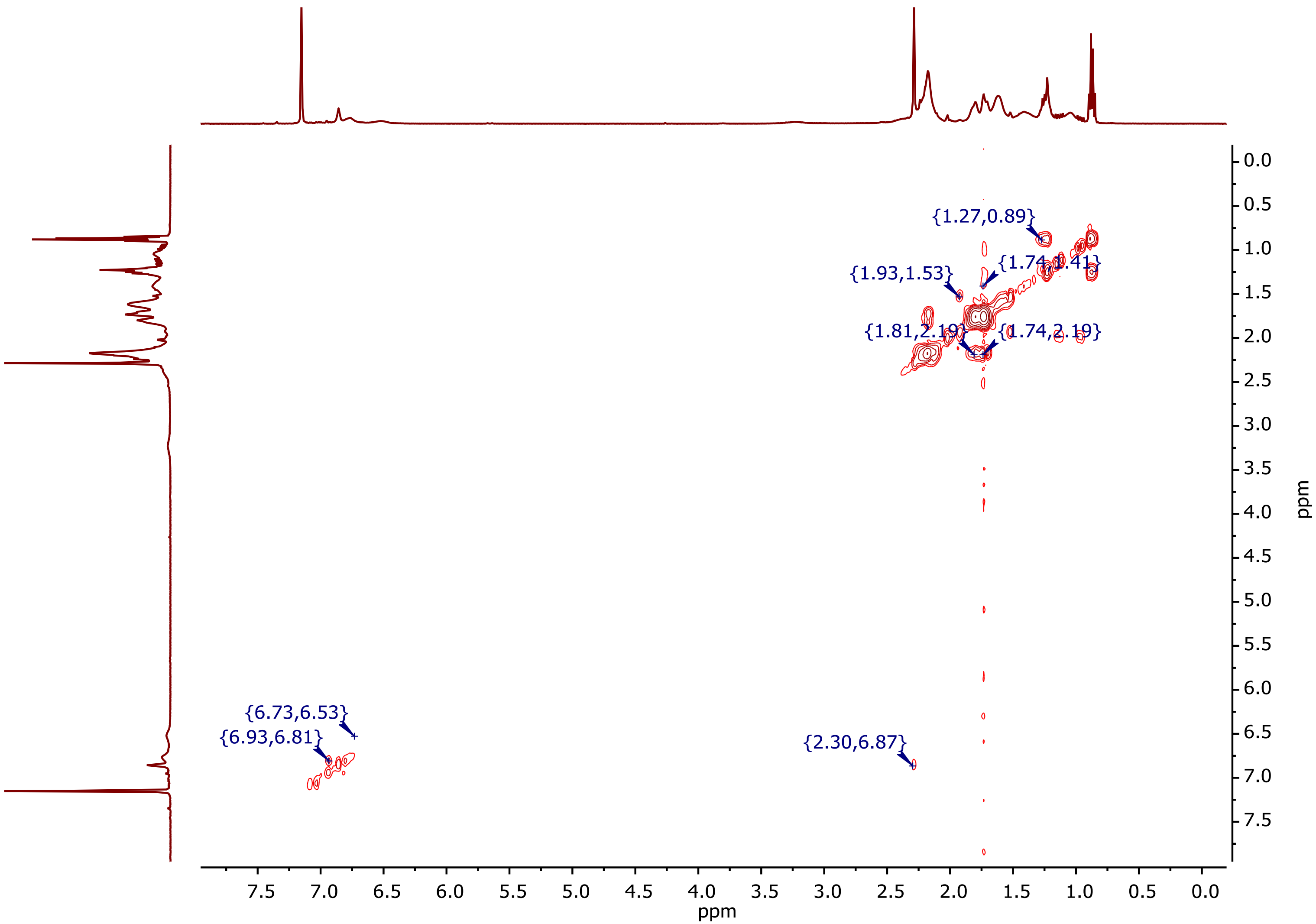

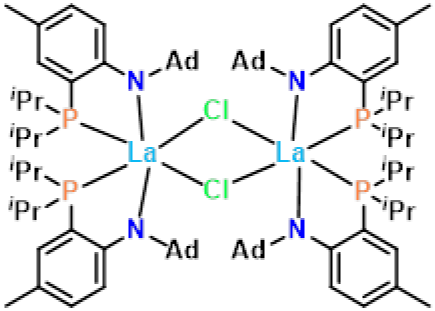

Figure S 152: ^1H-^1H COSY NMR spectrum of **3a** in C_6D_6 at 298 K.

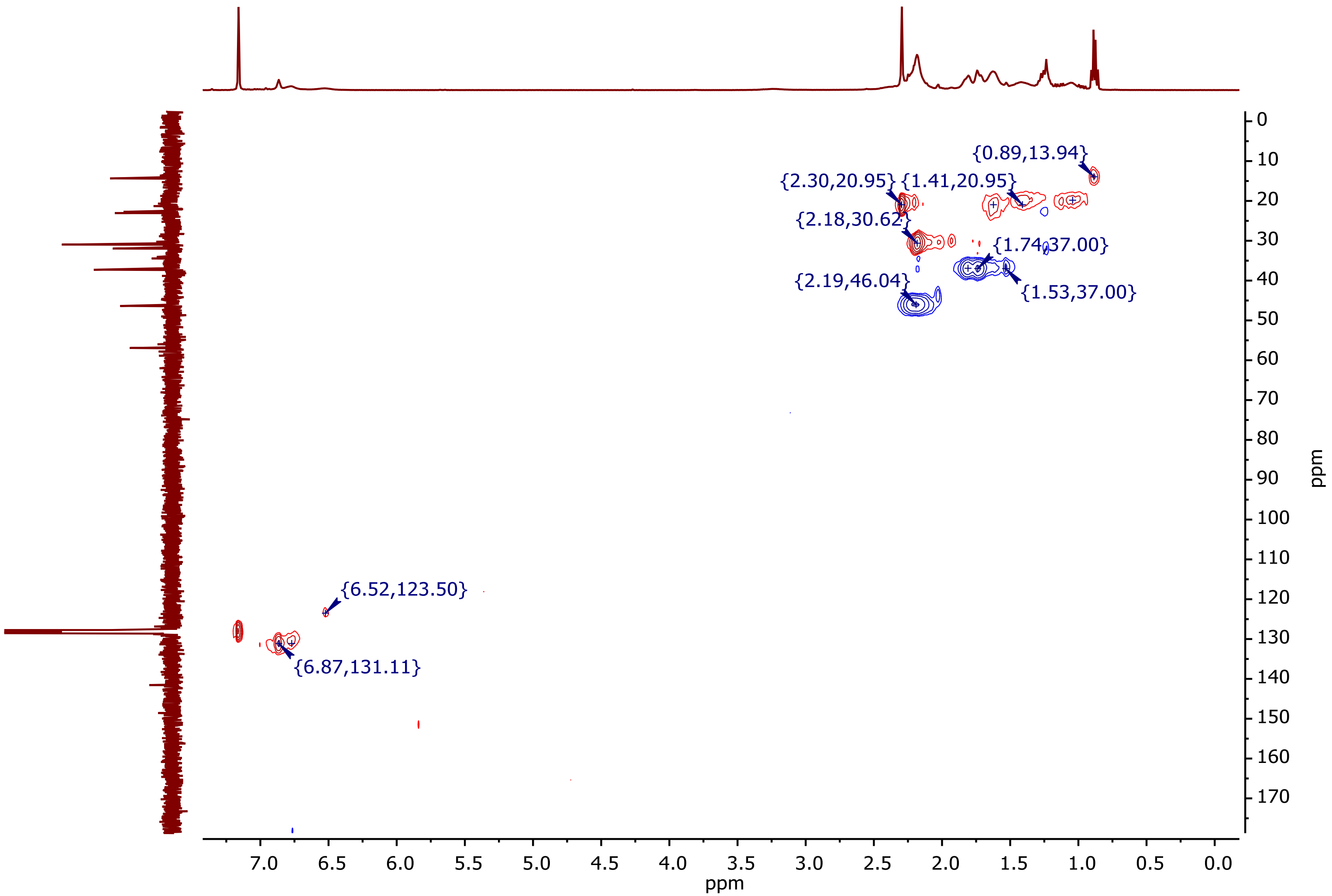

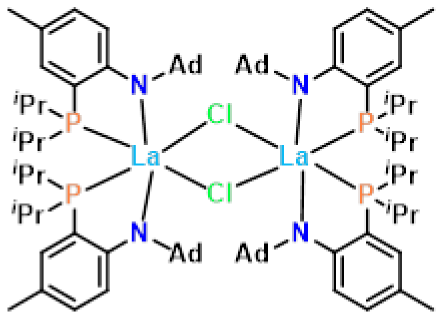

*Figure S 153: ^{1}H-^{13}C HSQC NMR spectrum of **3a** in C_6D_6 at 298 K.*

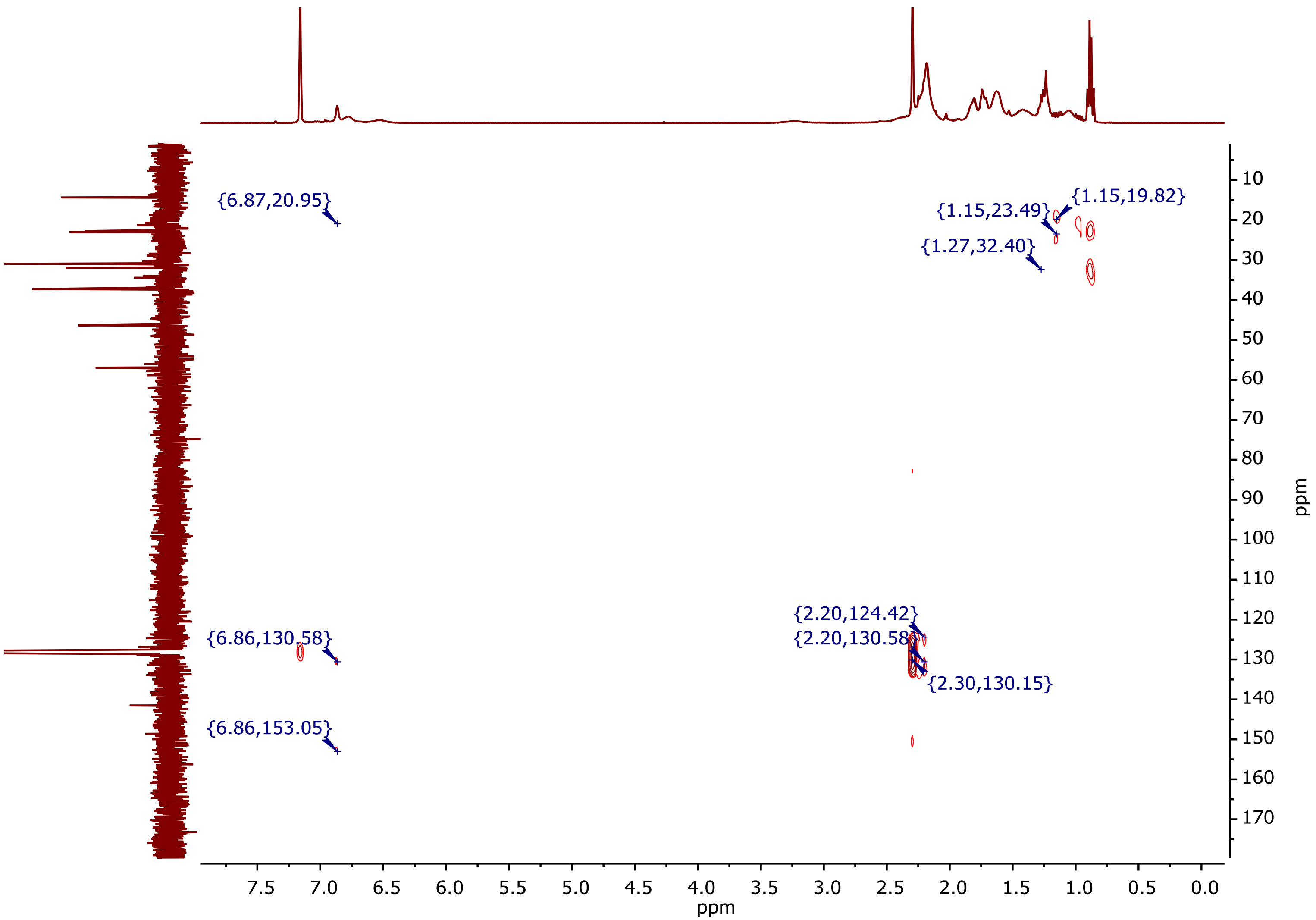

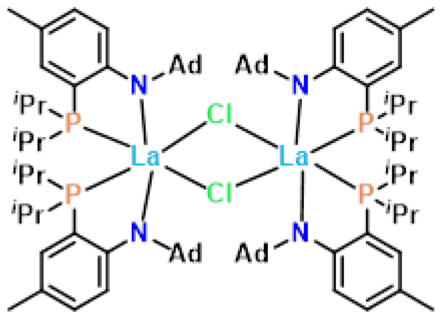

*Figure S 154: ¹H-¹³C HMBC NMR spectrum of **3a** in C_6D_6 at 298 K.*

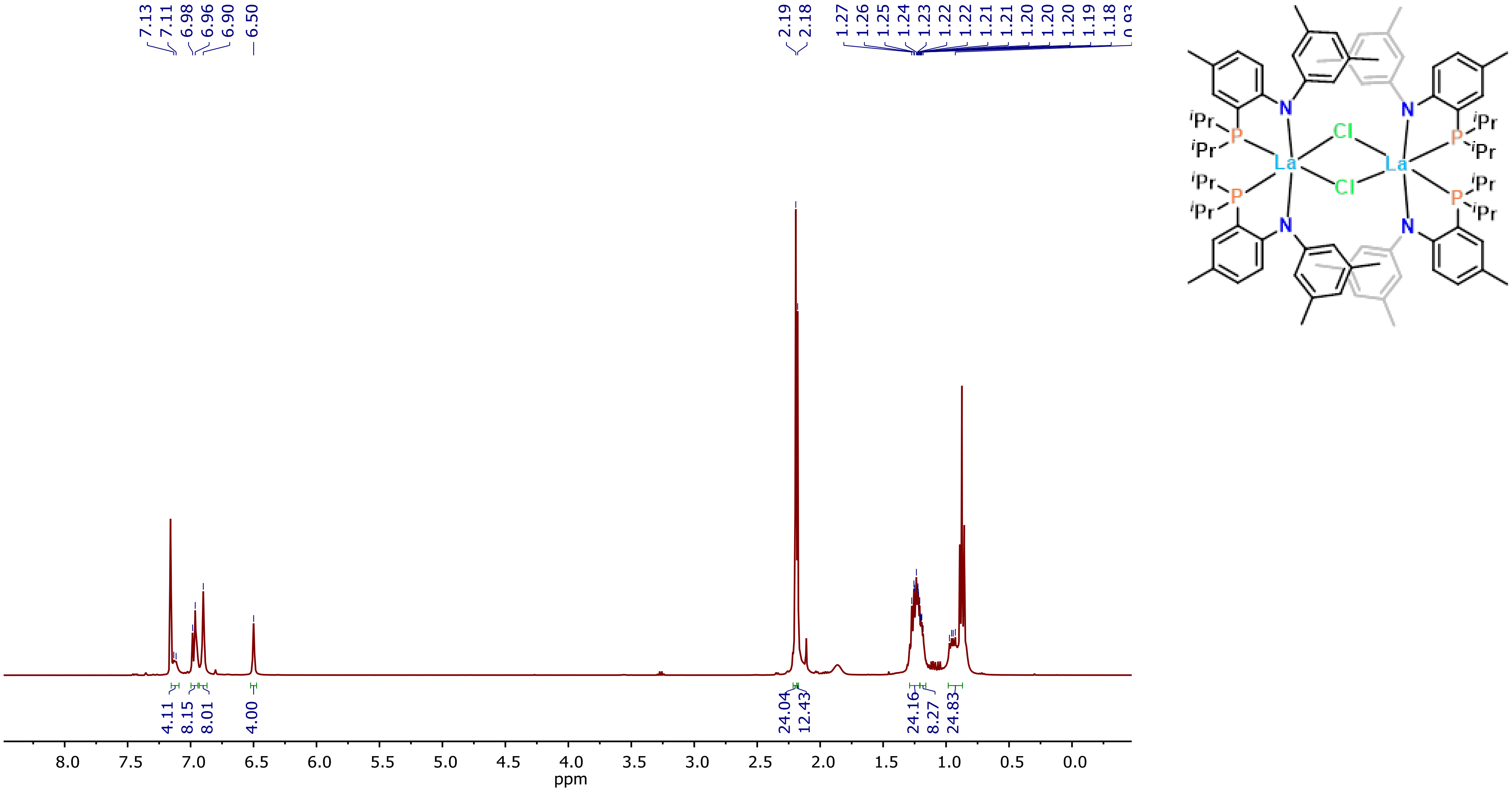

*Figure S 155: ^{1}H NMR spectrum of **3d** in C_6D_6 at 298 K.*

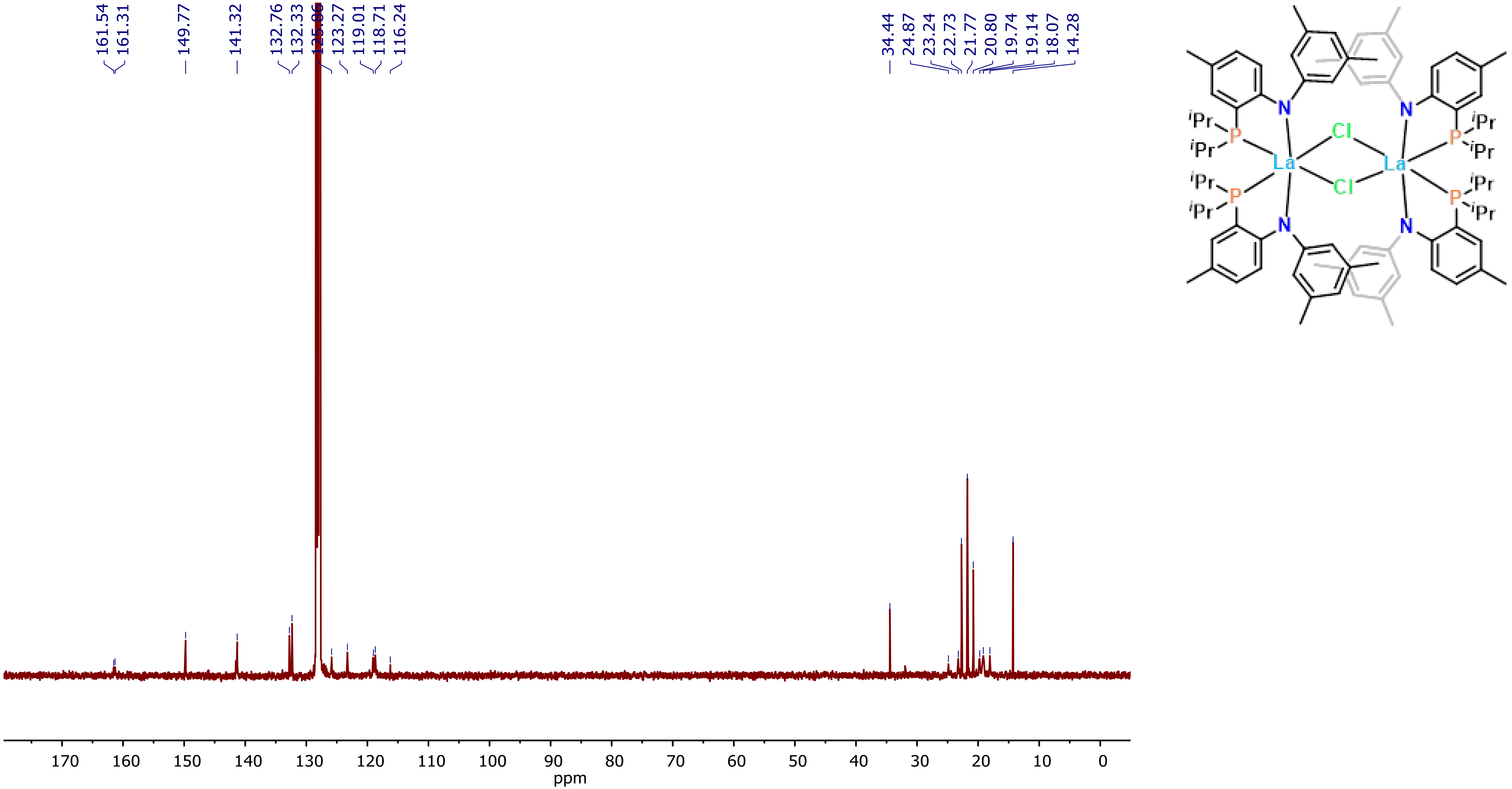

*Figure S 156: $^{13}C\{^{1}H\}$ NMR spectrum of **3d** in C_6D_6 at 298 K.*

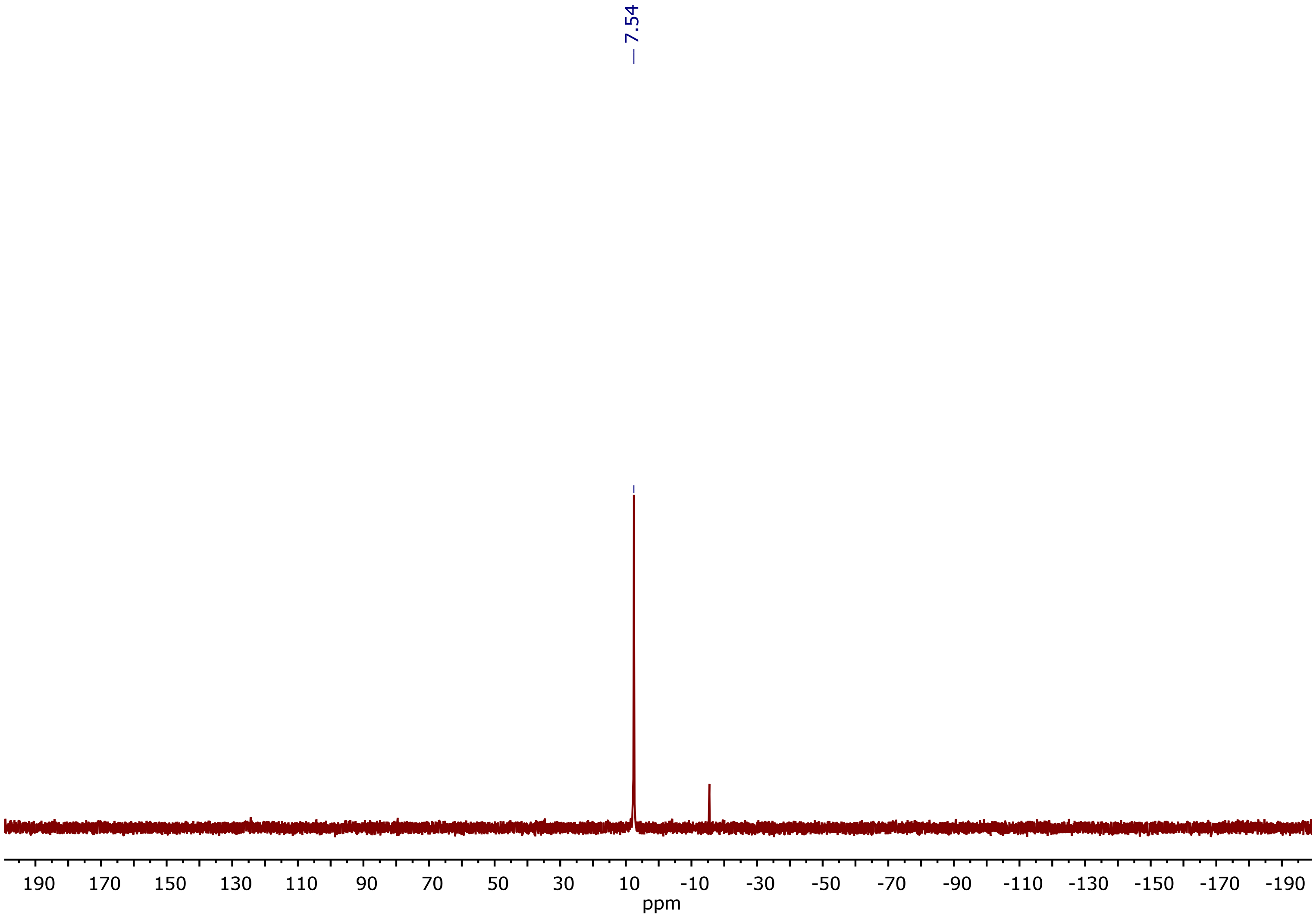

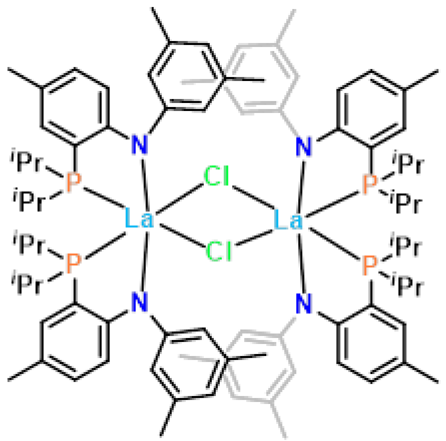

*Figure S 157: $^{31}P\{^1H\}$ NMR spectrum of **3d** in C_6D_6 at 298 K.*

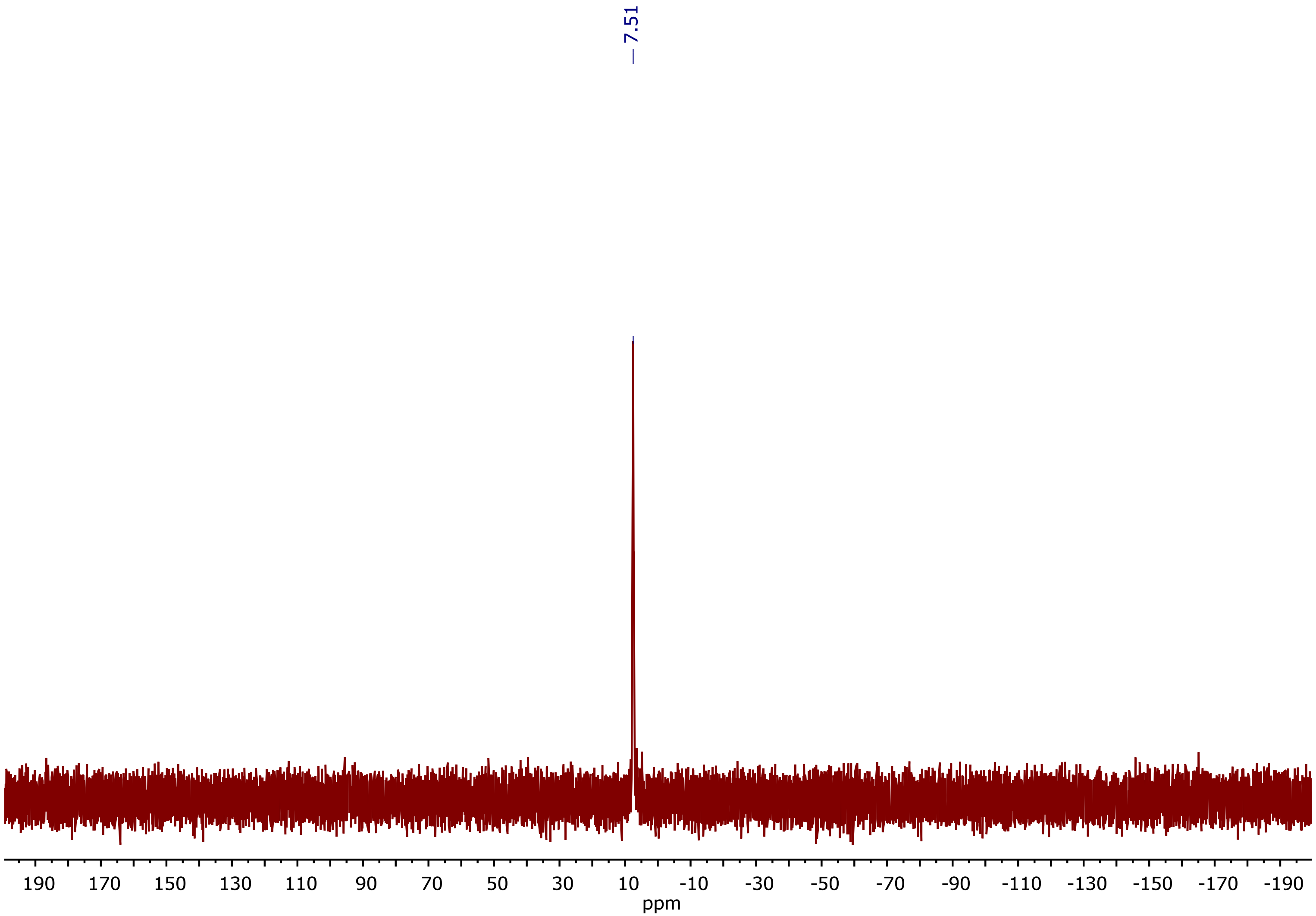

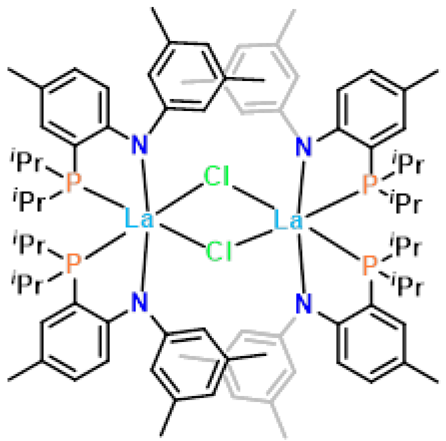

*Figure S 158: ^{31}P NMR spectrum of **3d** in C_6D_6 at 298 K.*

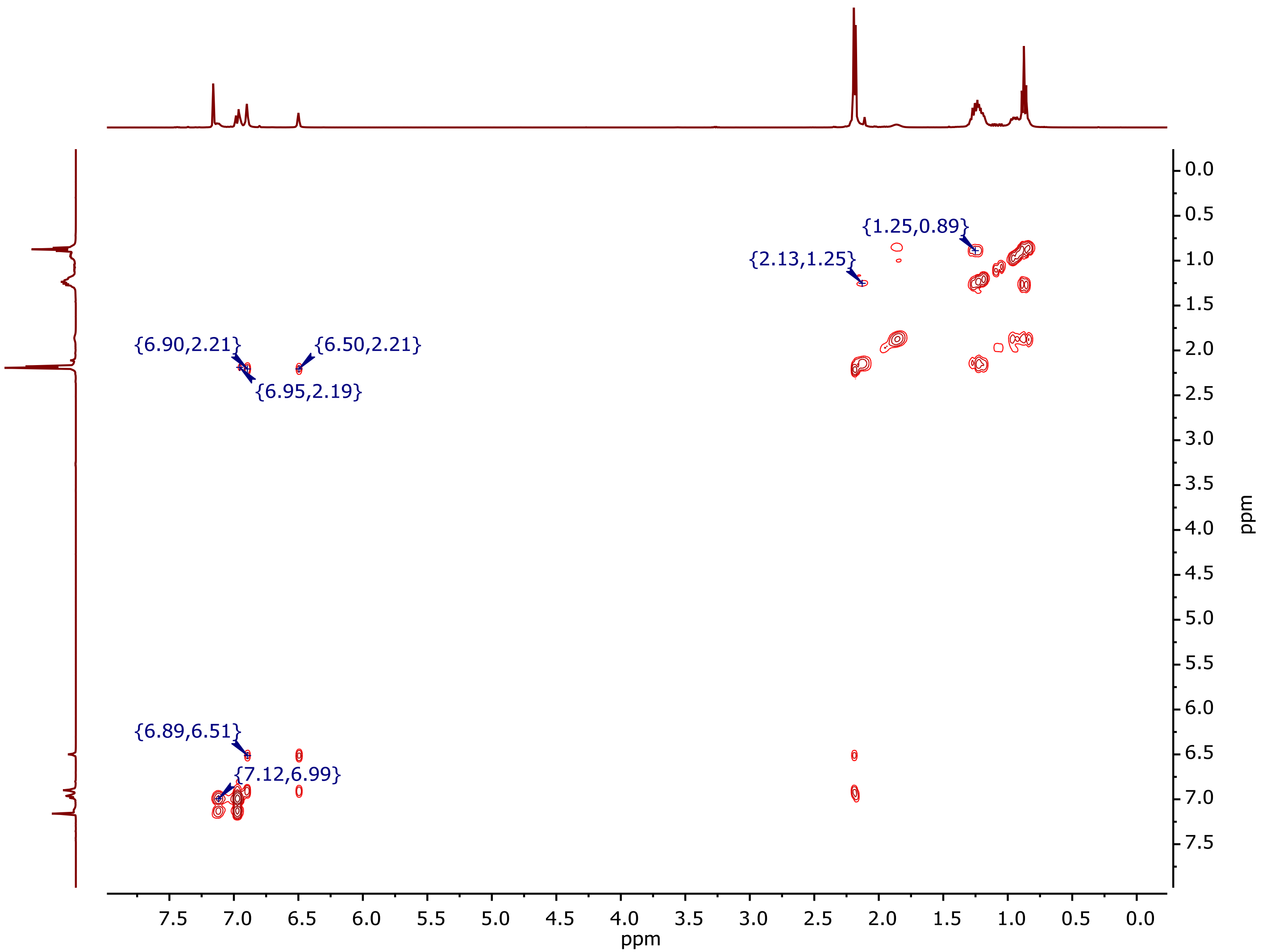

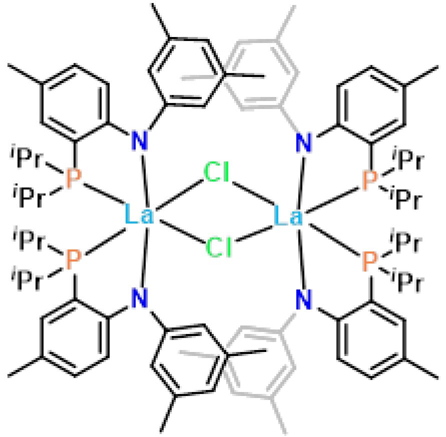

*Figure S 159: ¹H-¹H COSY NMR spectrum of **3d** in C_6D_6 at 298 K.*

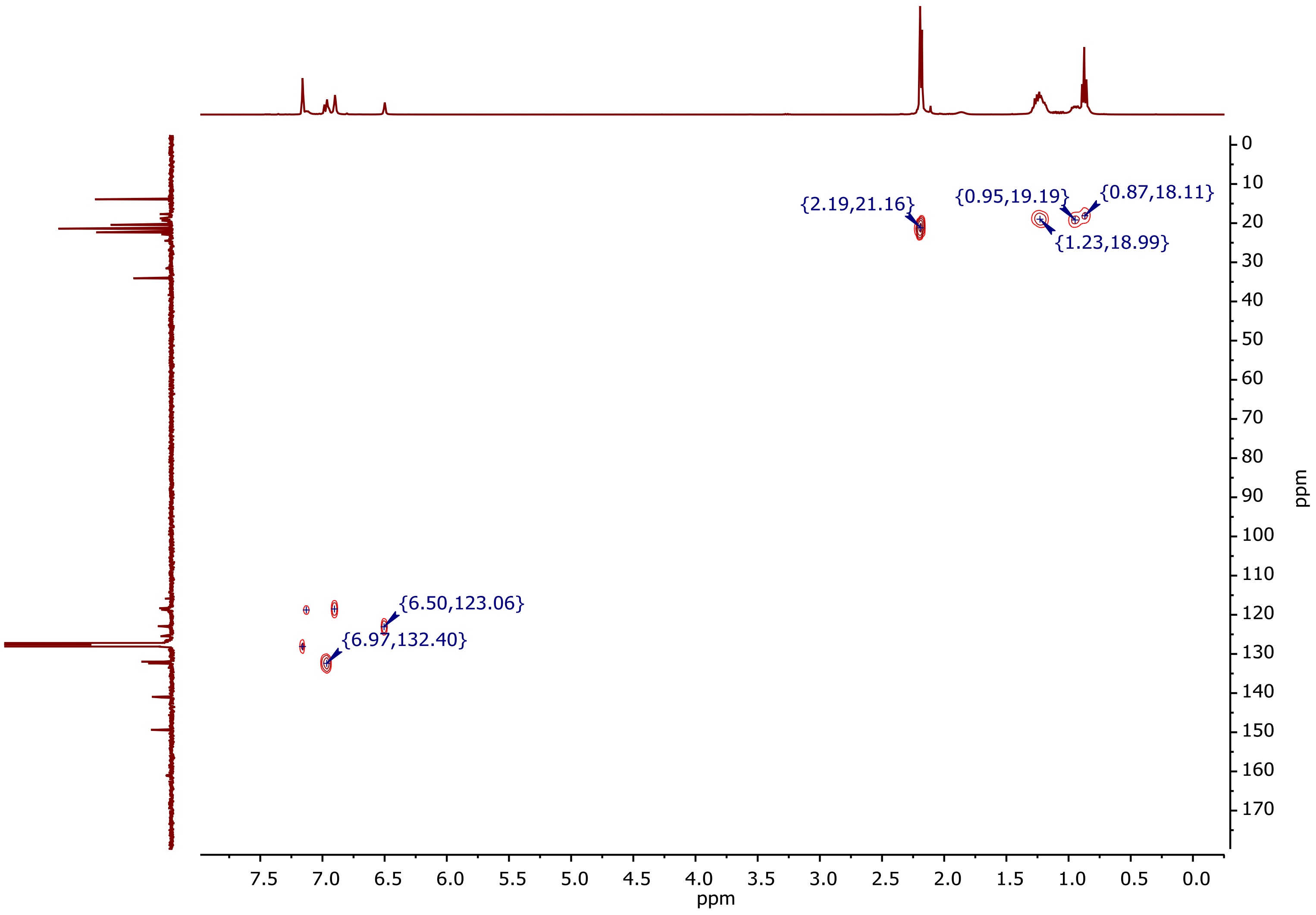

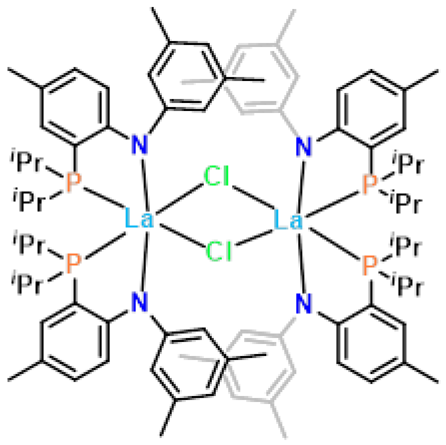

*Figure S 160: ^{1}H-^{13}C HSQC NMR spectrum of **3d** in C_6D_6 at 298 K.*

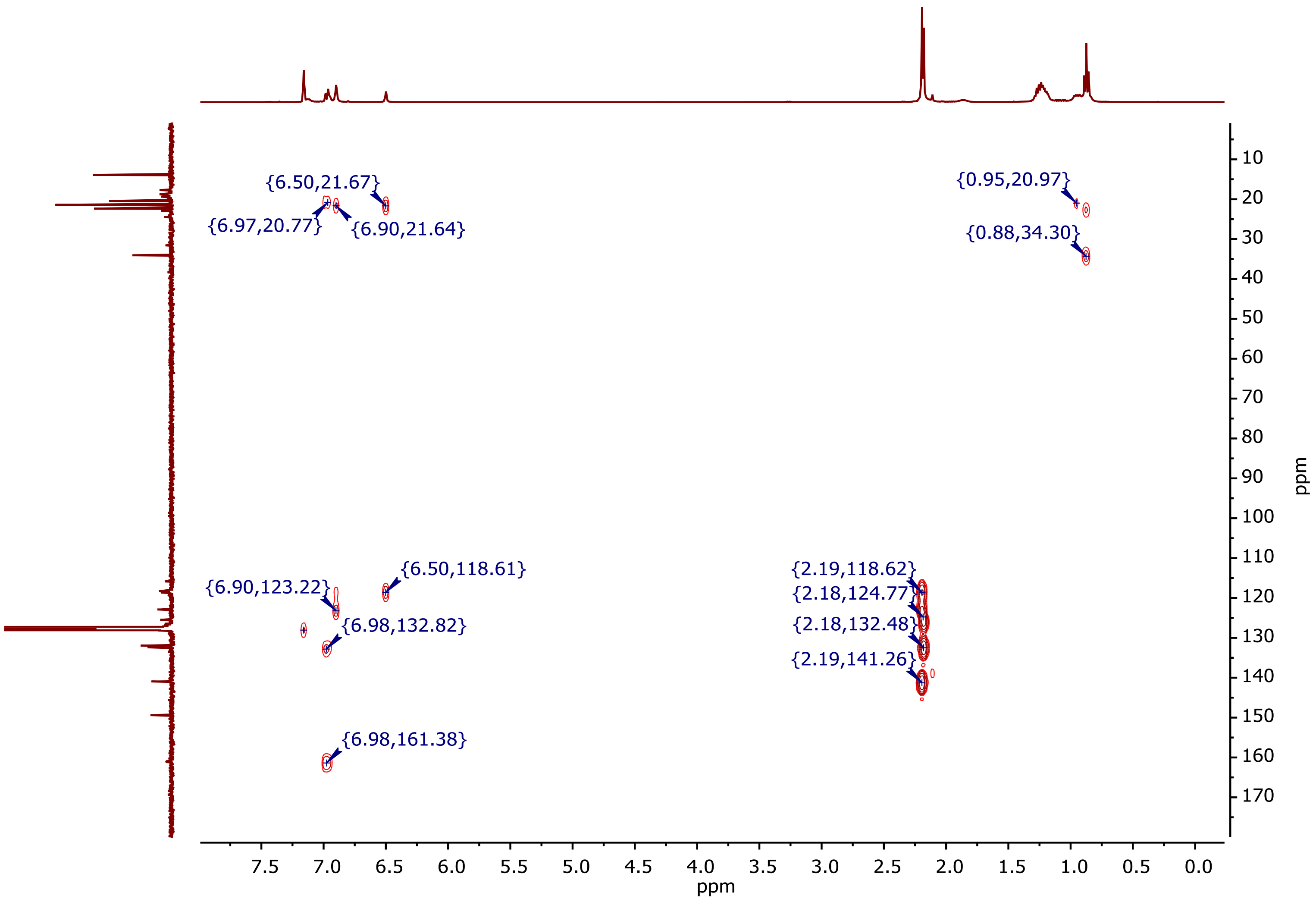

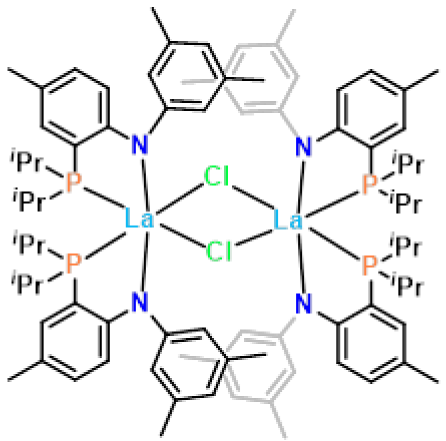

*Figure S 161: ^{1}H-^{13}C HMBC NMR spectrum of **3d** in C_6D_6 at 298 K.*

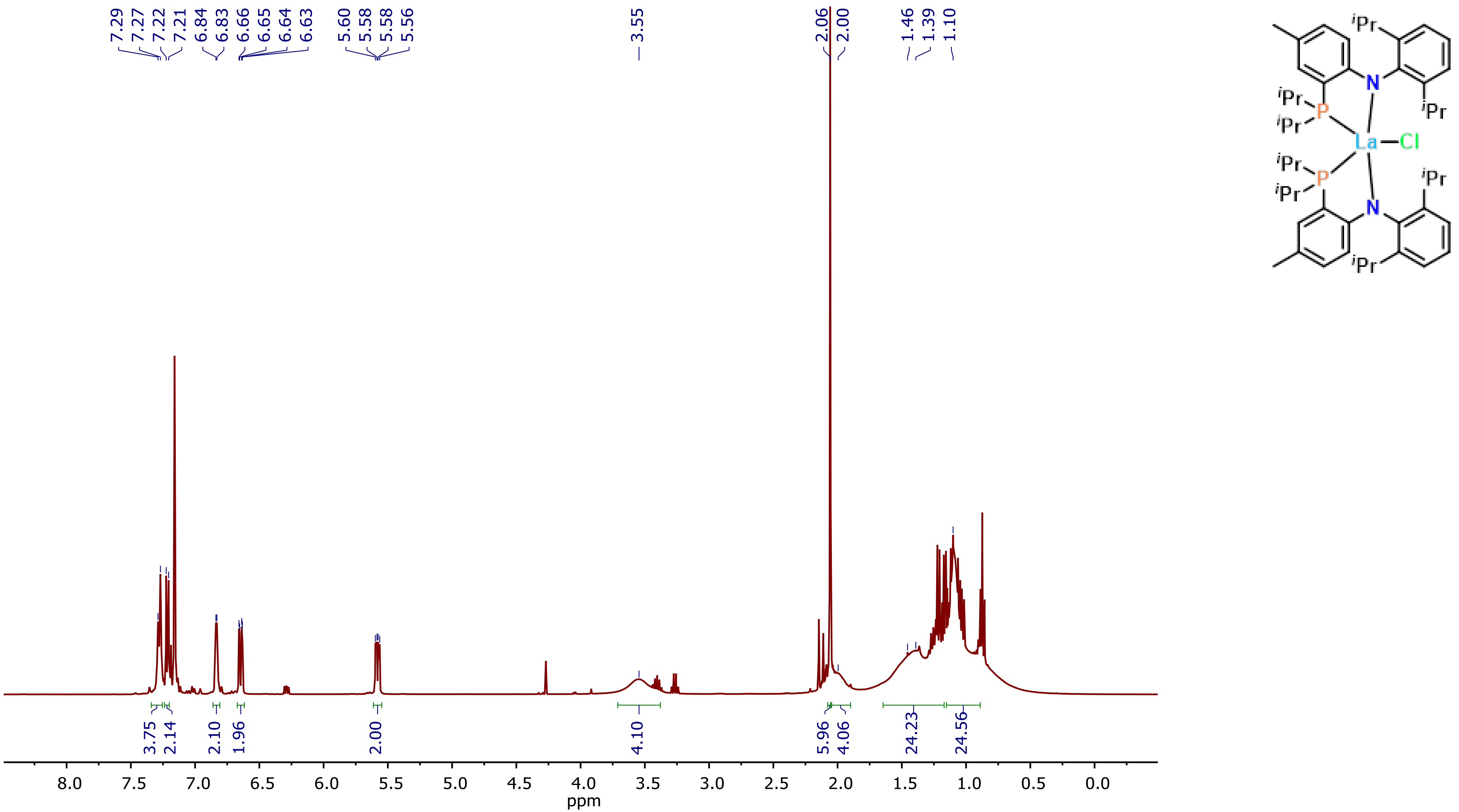

Figure S 162: ^{1}H NMR spectrum of **3e** in C_6D_6 at 298 K.

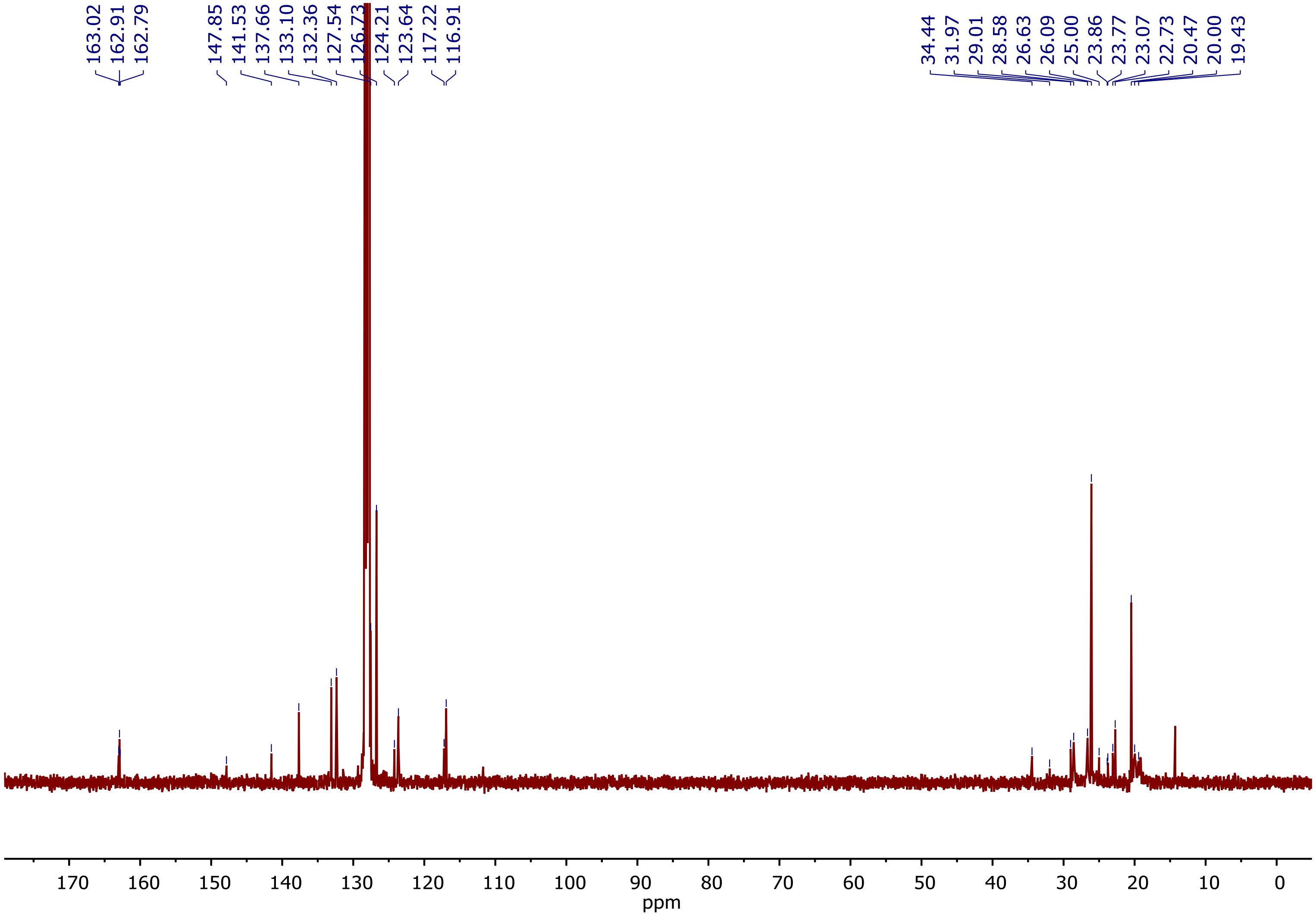

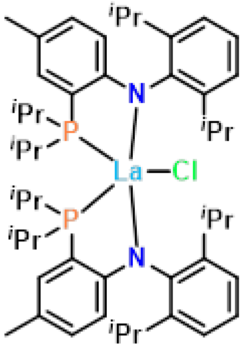

*Figure S 163: $^{13}C\{^1H\}$ NMR spectrum of **3e** in C_6D_6 at 298 K.*

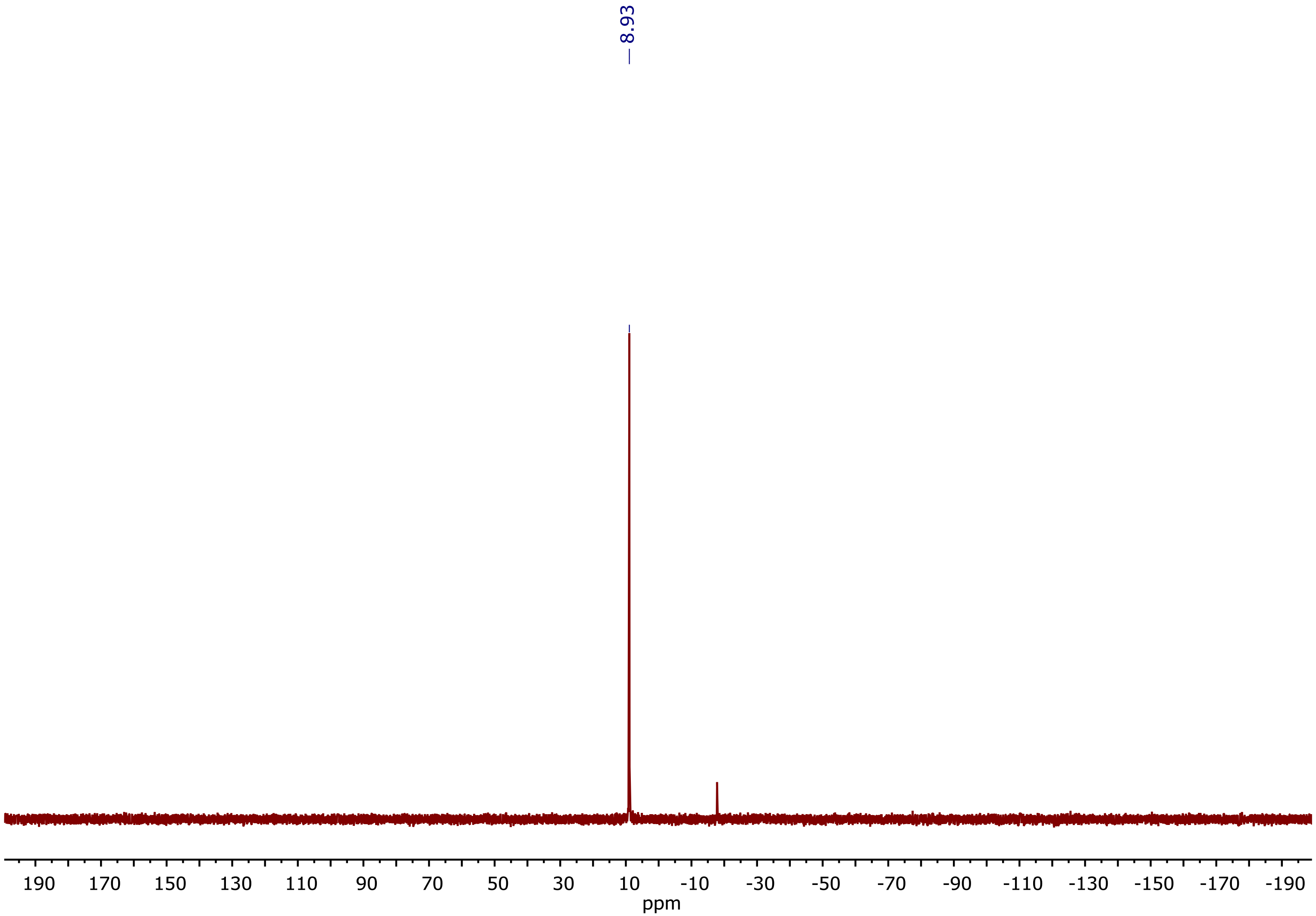

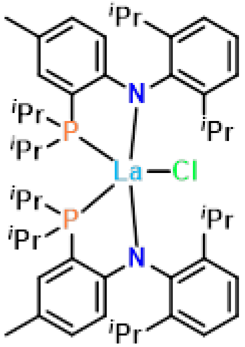

*Figure S 164: $^{31}P\{^1H\}$ NMR spectrum of **3e** in C_6D_6 at 298 K.*

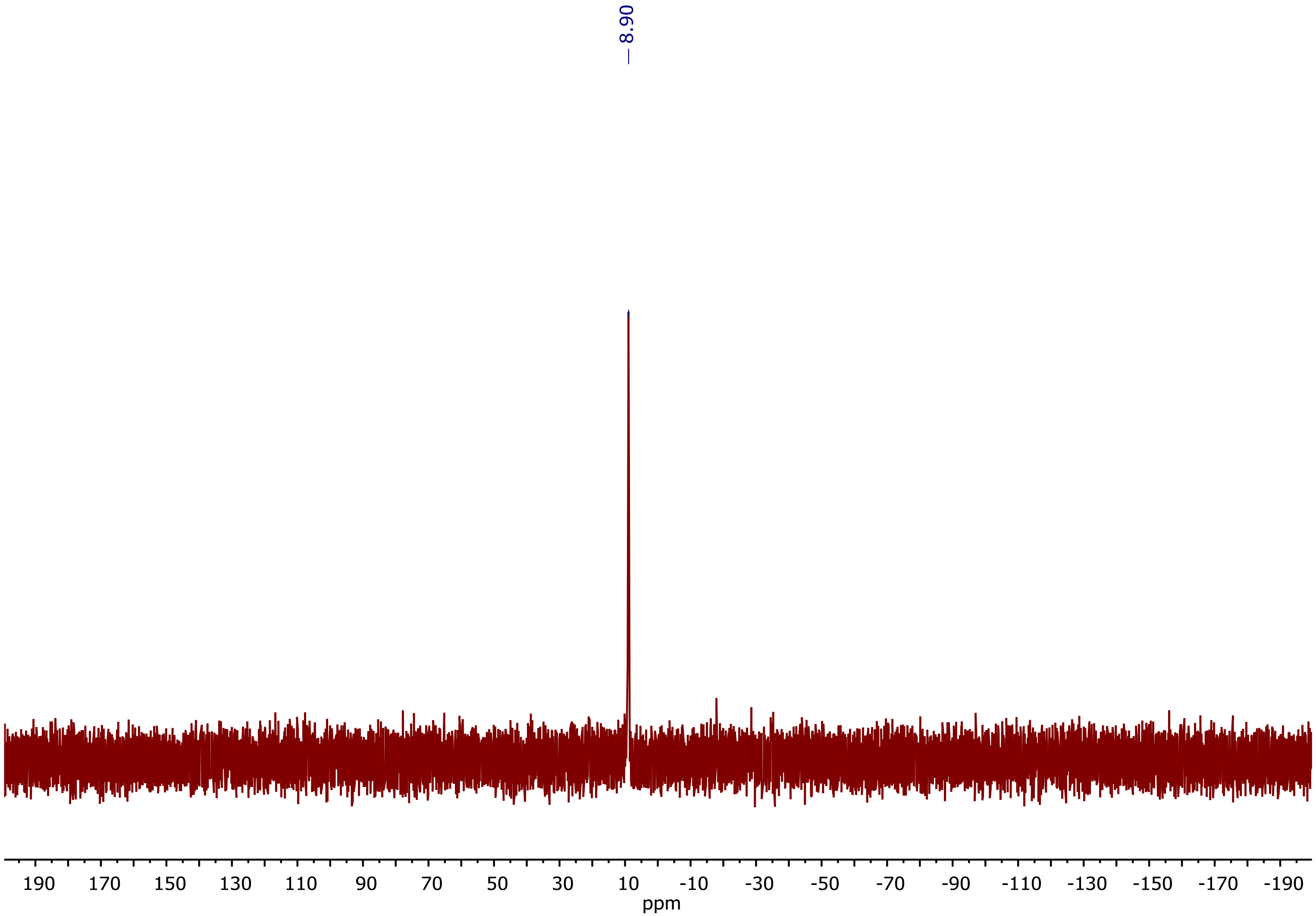

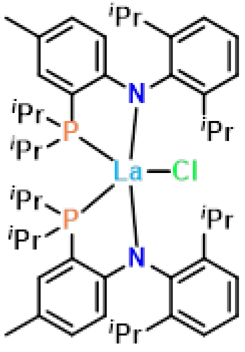

*Figure S 165: ^{31}P NMR spectrum of **3e** in C_6D_6 at 298 K.*

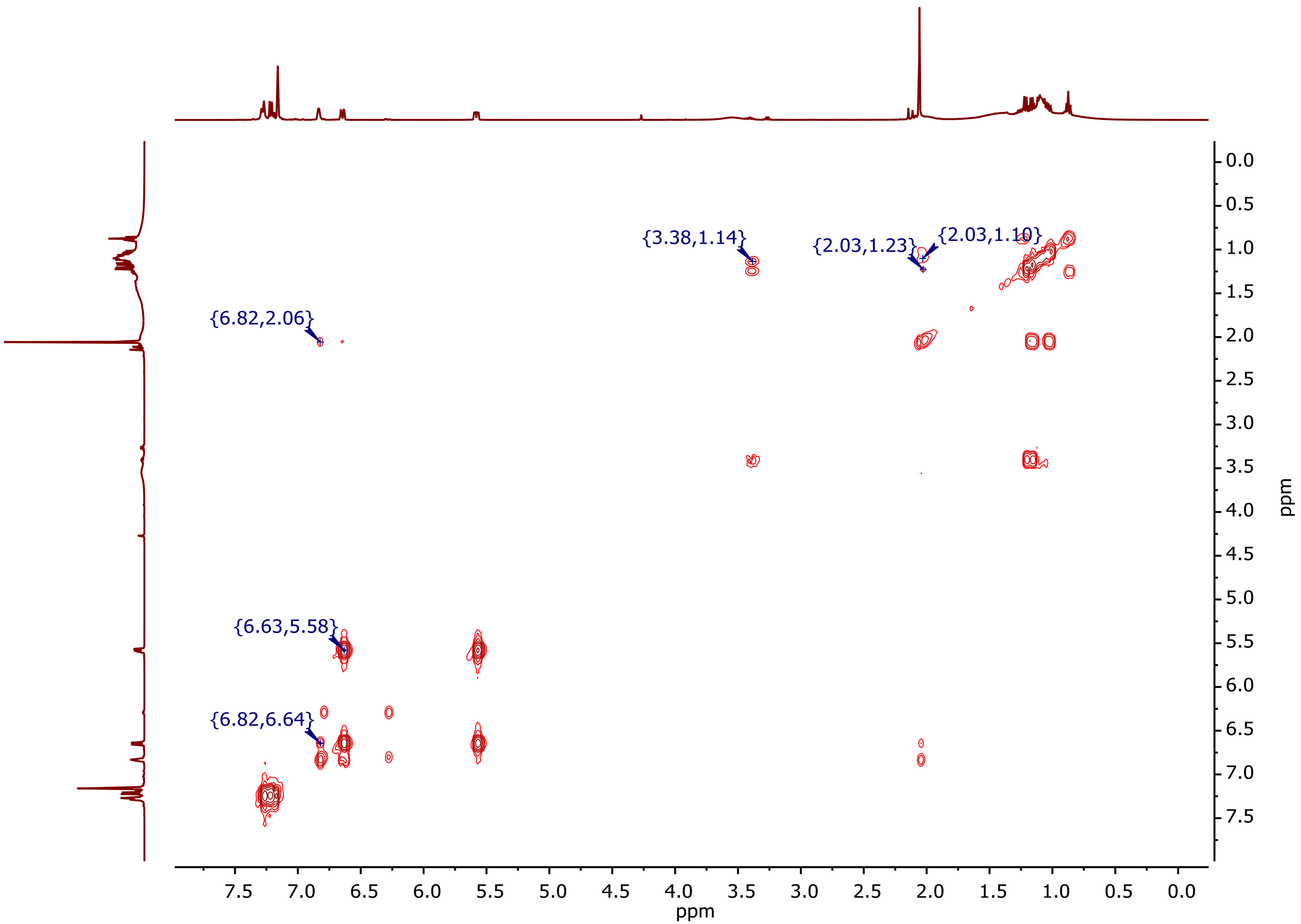

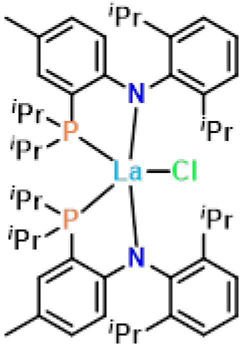

*Figure S 166: ^{1}H-^{1}H COSY NMR spectrum of **3e** in C_6D_6 at 298 K.*

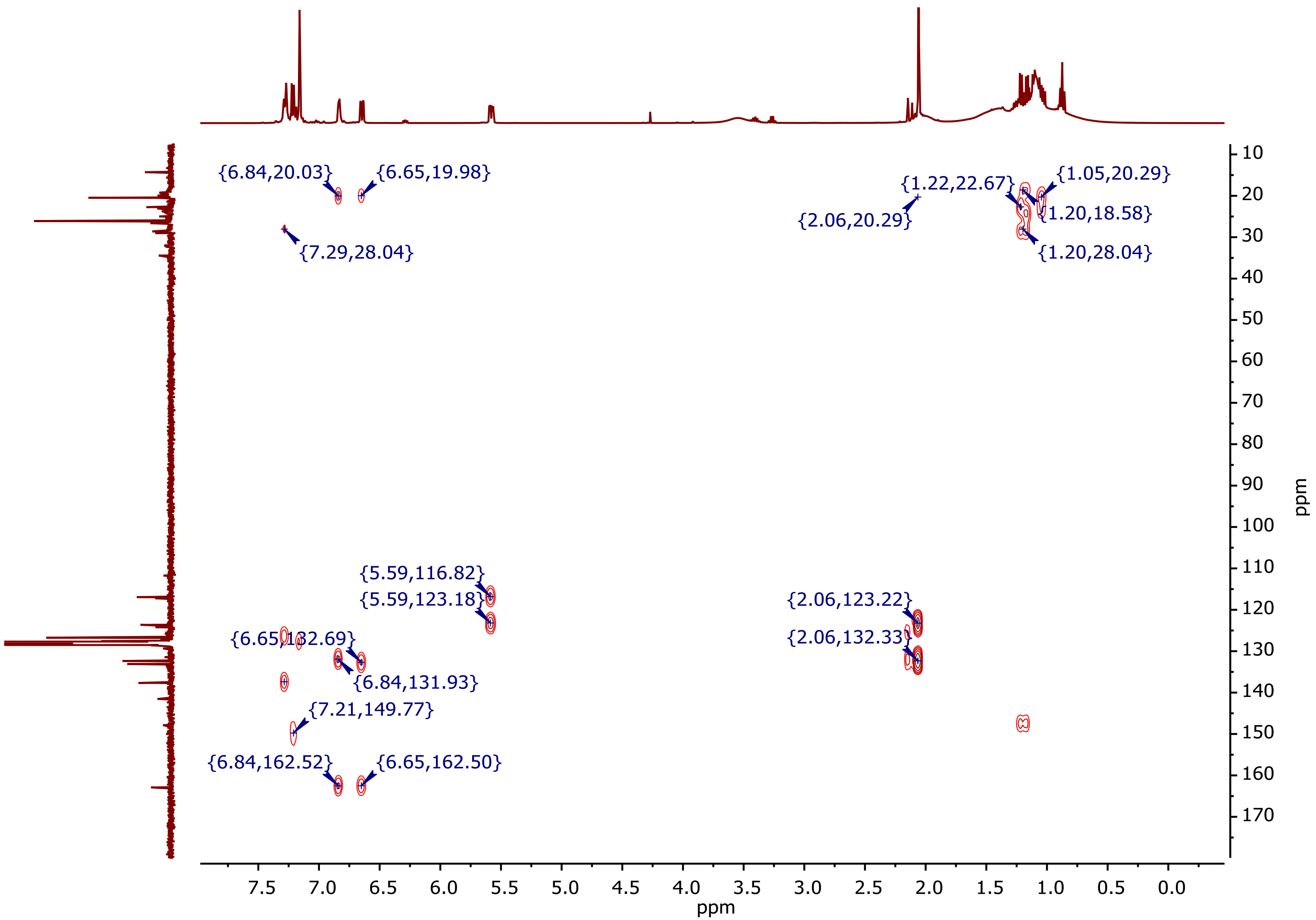

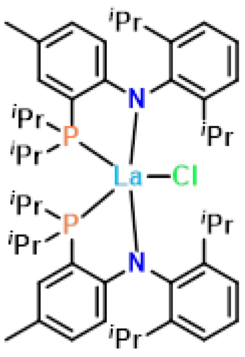

*Figure S 167: ^{1}H-^{13}C HSQC NMR spectrum of **3e** in C_6D_6 at 298 K.*

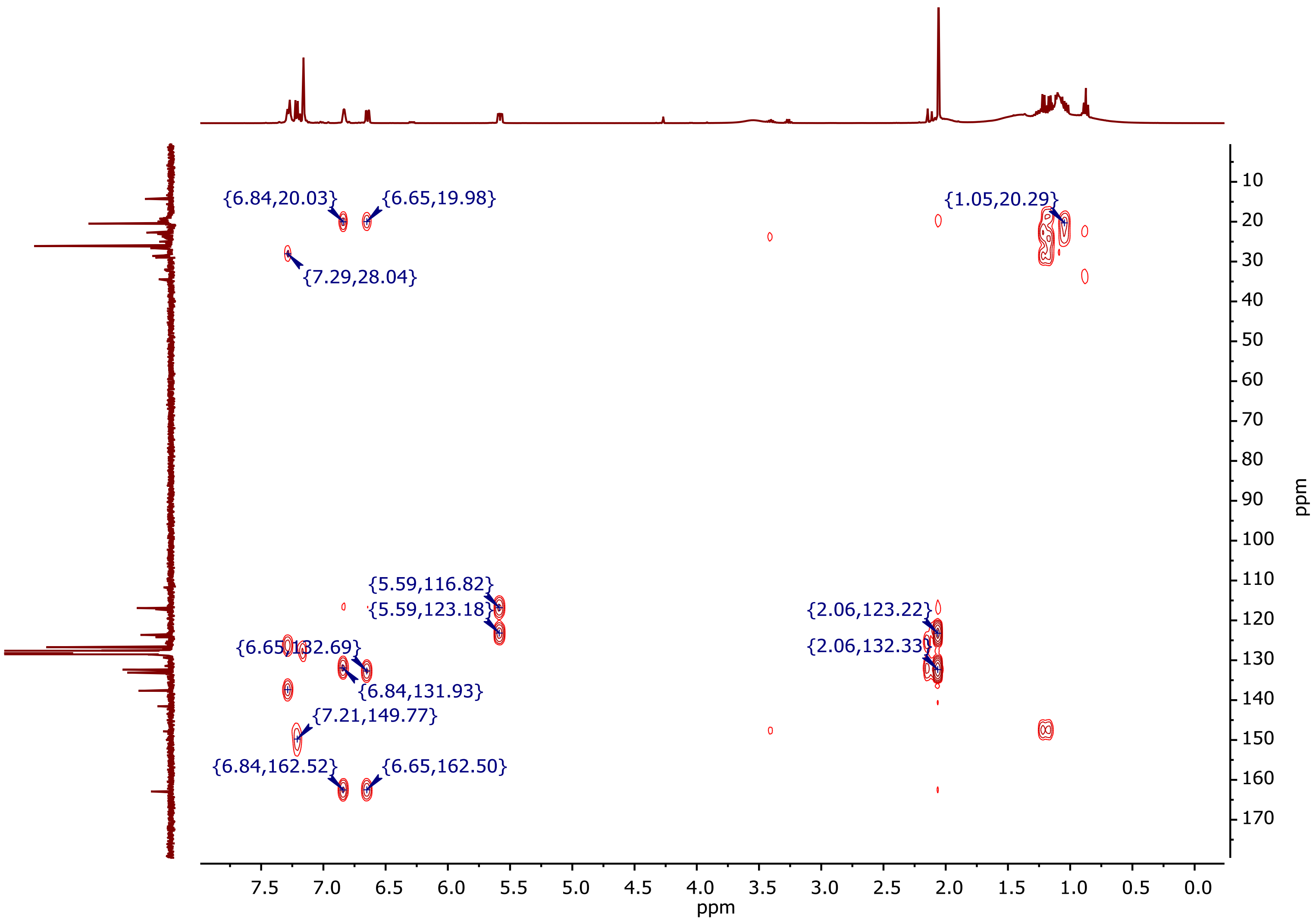

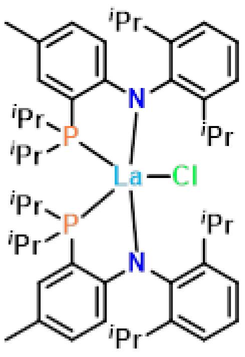

*Figure S 168: ¹H-¹³C HMBC NMR spectrum of **3e** in C_6D_6 at 298 K.*

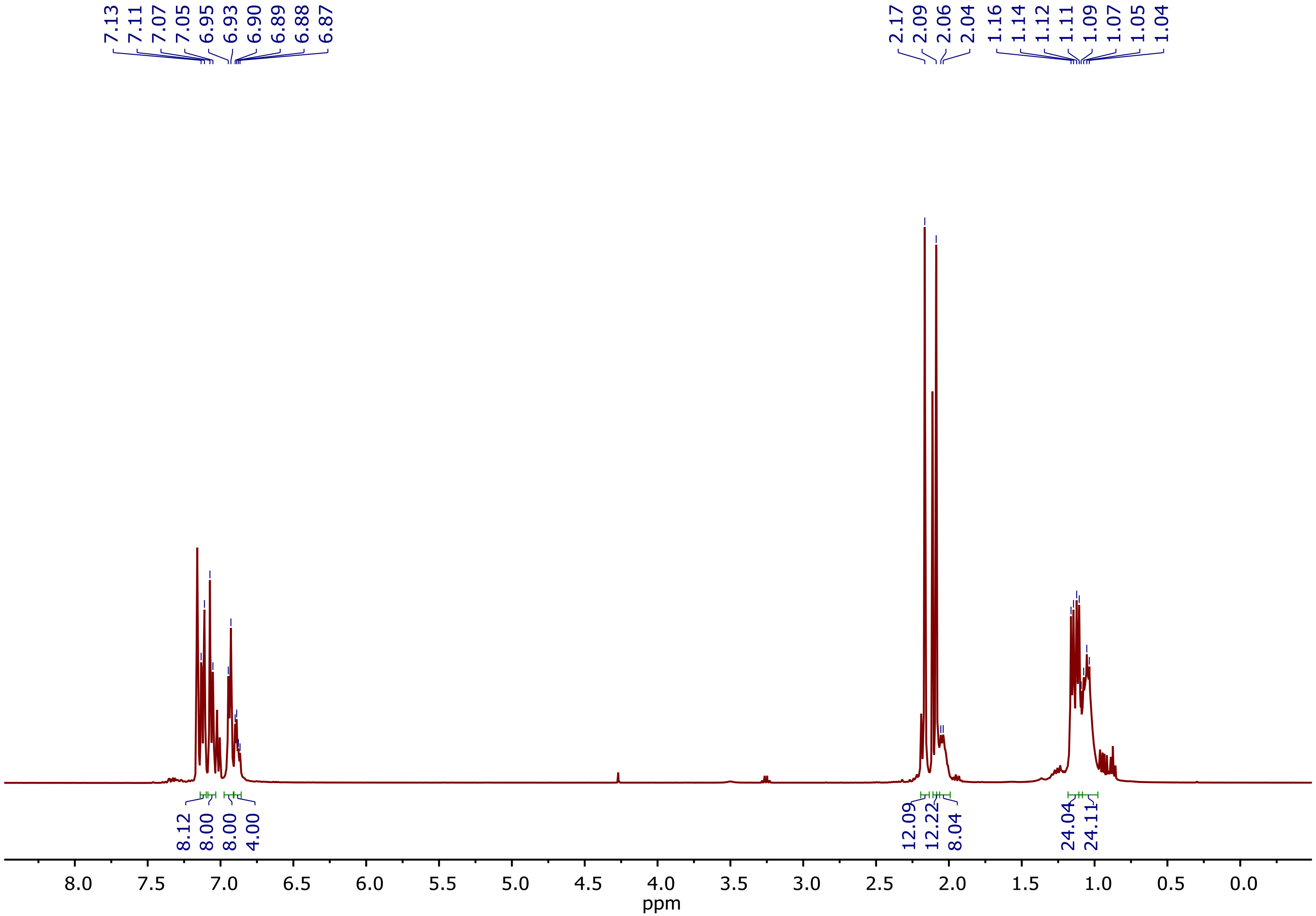

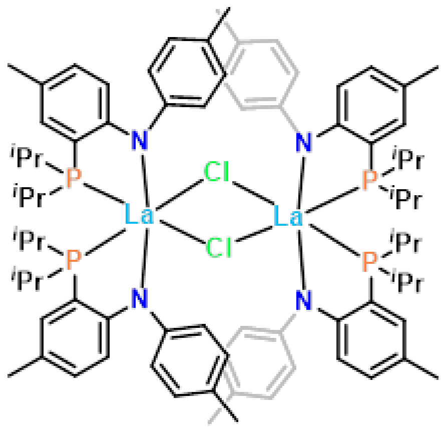

*Figure S 169: ^{1}H NMR spectrum of **3f** in C_6D_6 at 298 K.*

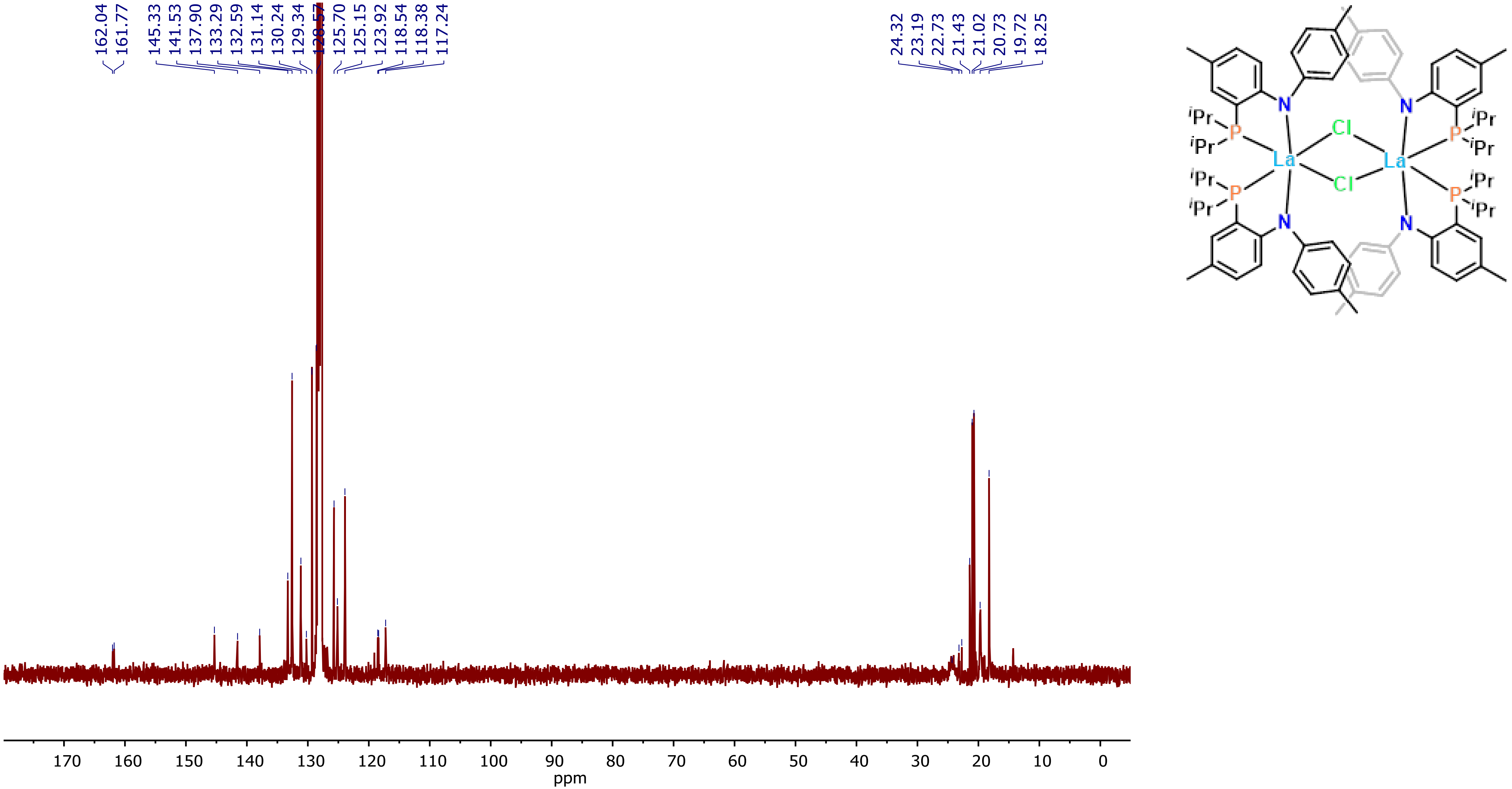

*Figure S 170: $^{13}C\{^{1}H\}$ NMR spectrum of **3f** in C_6D_6 at 298 K.*

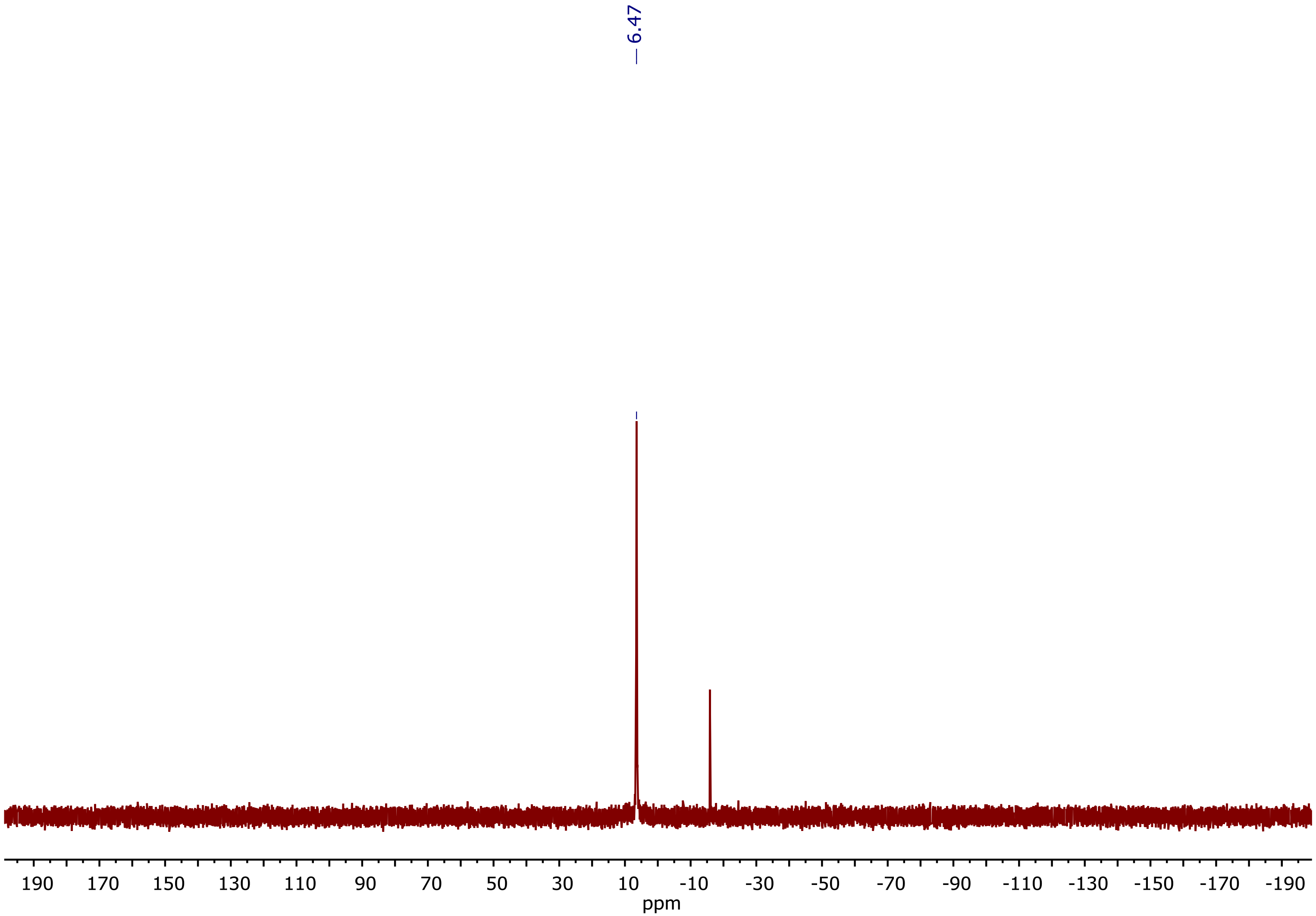

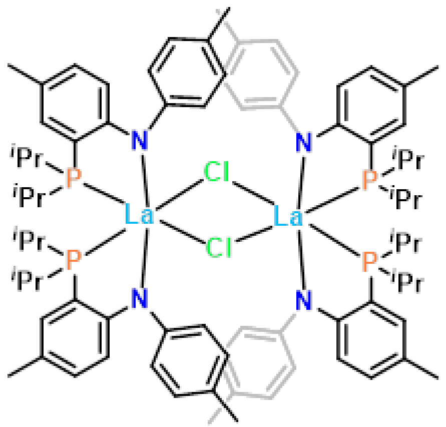

*Figure S 171: $^{31}P\{^{1}H\}$ NMR spectrum of **3f** in C_6D_6 at 298 K.*

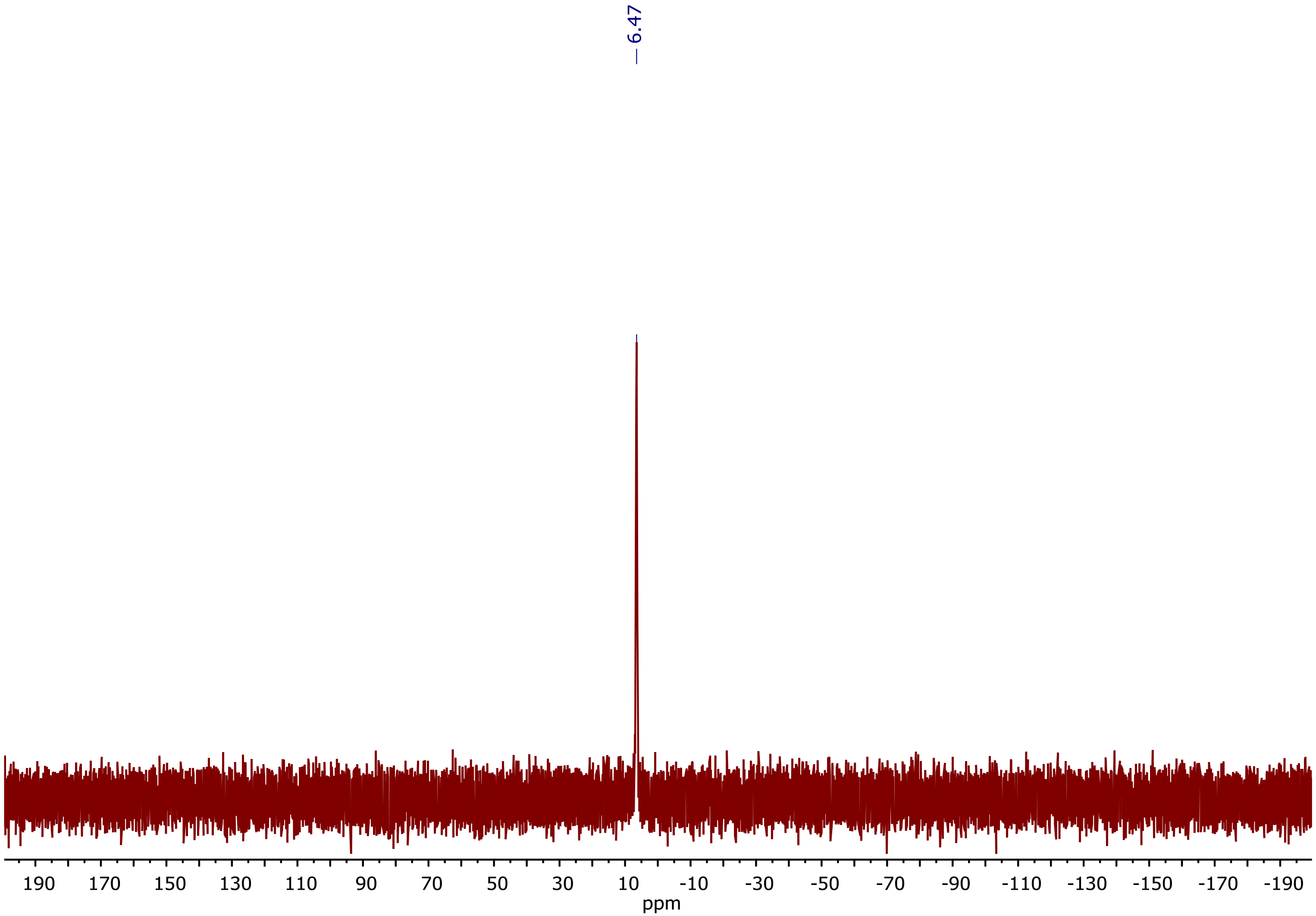

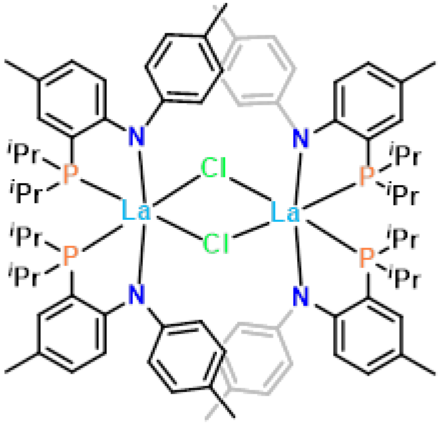

Figure S 172: ^{31}P NMR spectrum of ***3f*** in C_6D_6 at 298 K.

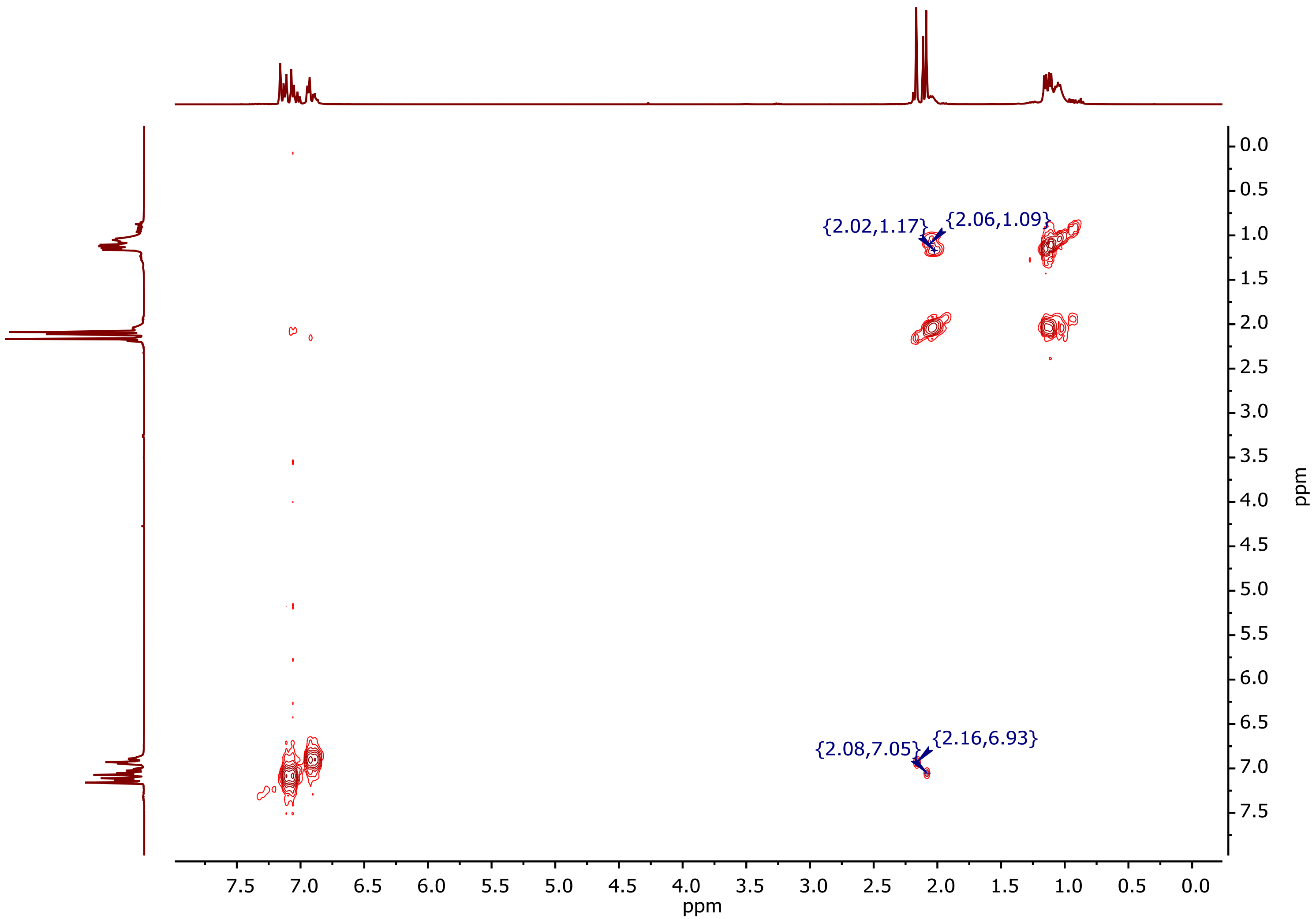

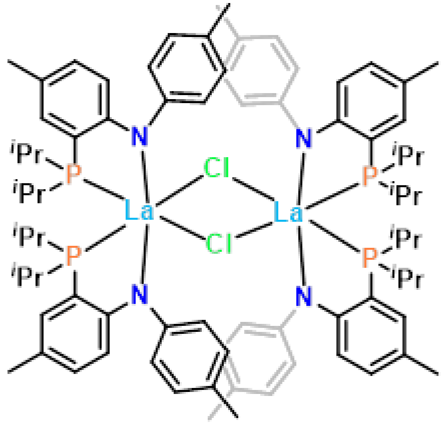

*Figure S 173: ^{1}H-^{1}H COSY NMR spectrum of **3f** in C_6D_6 at 298 K.*

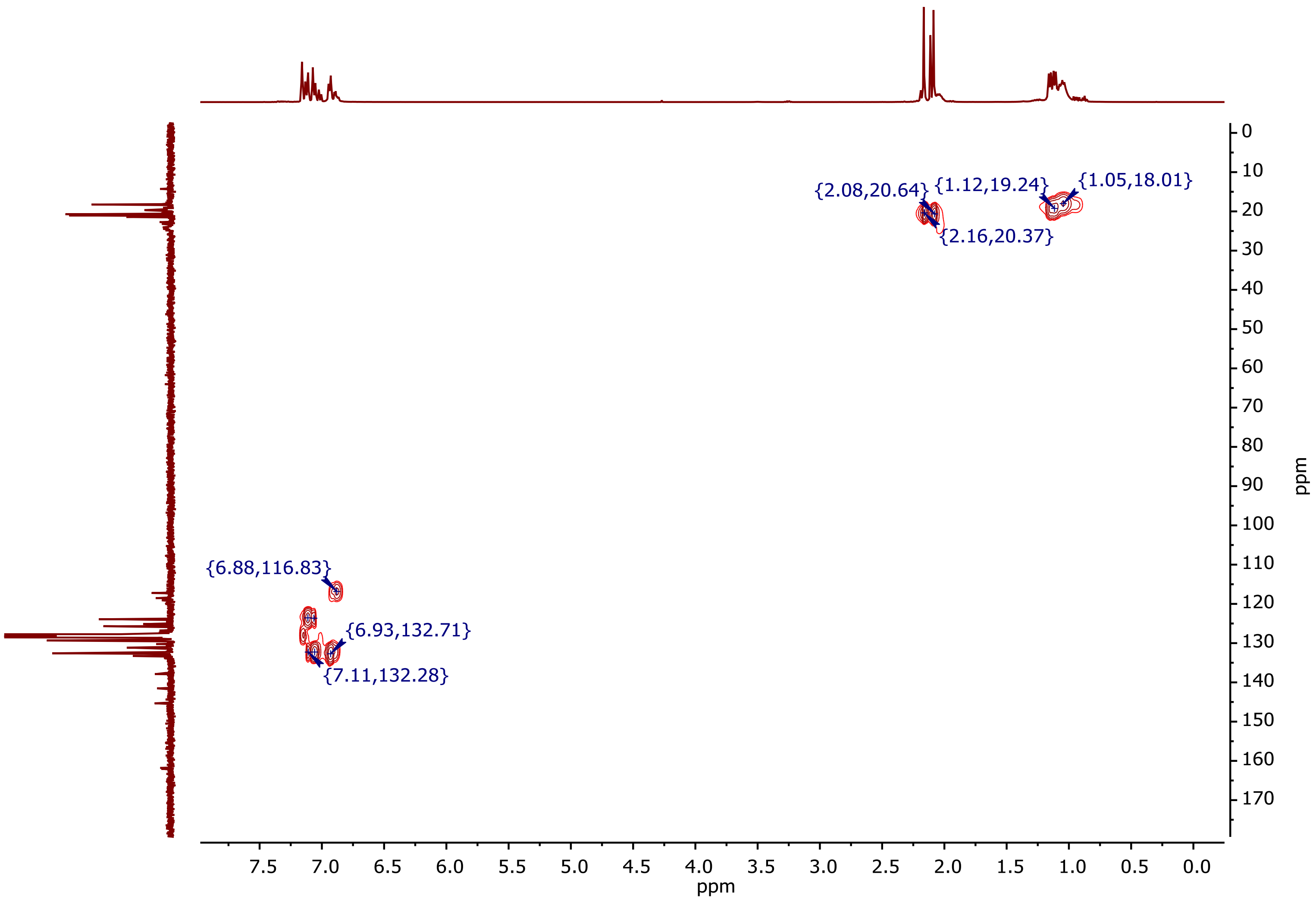

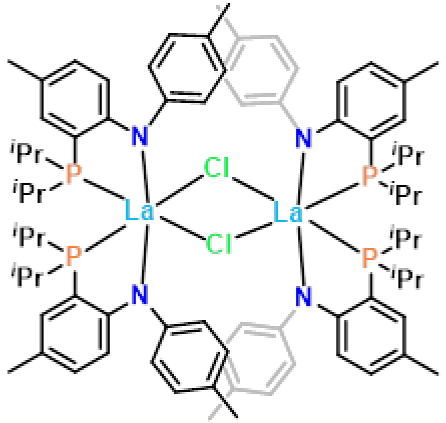

*Figure S 174: ^{1}H-^{13}C HSQC NMR spectrum of **3f** in C_6D_6 at 298 K.*

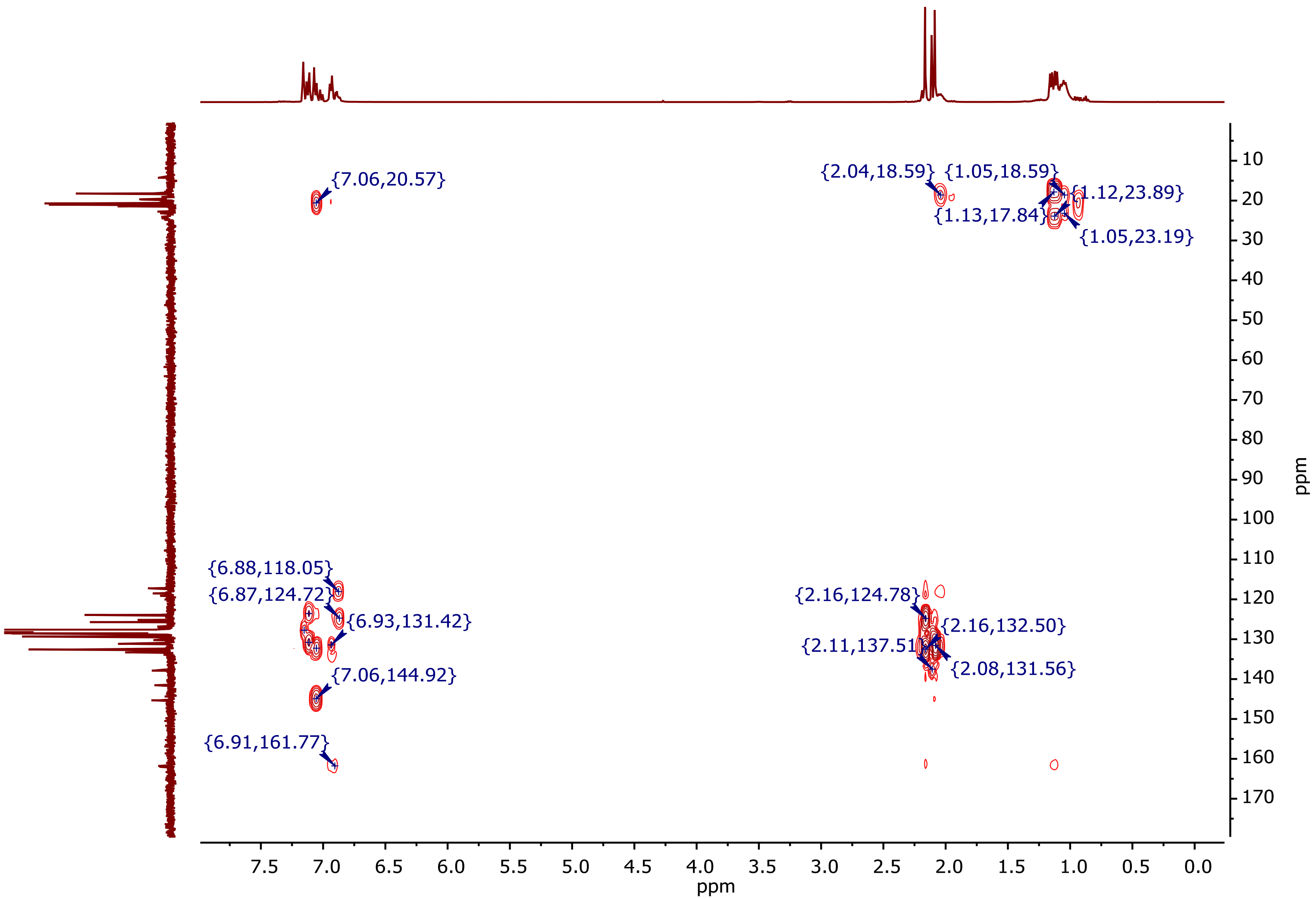

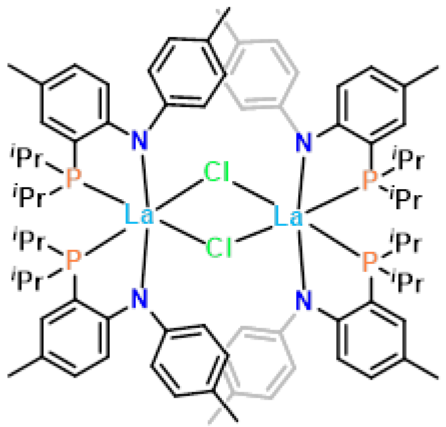

Figure S 175: ^{1}H-^{13}C HMBC NMR spectrum of ***3f*** in C_6D_6 at 298 K.

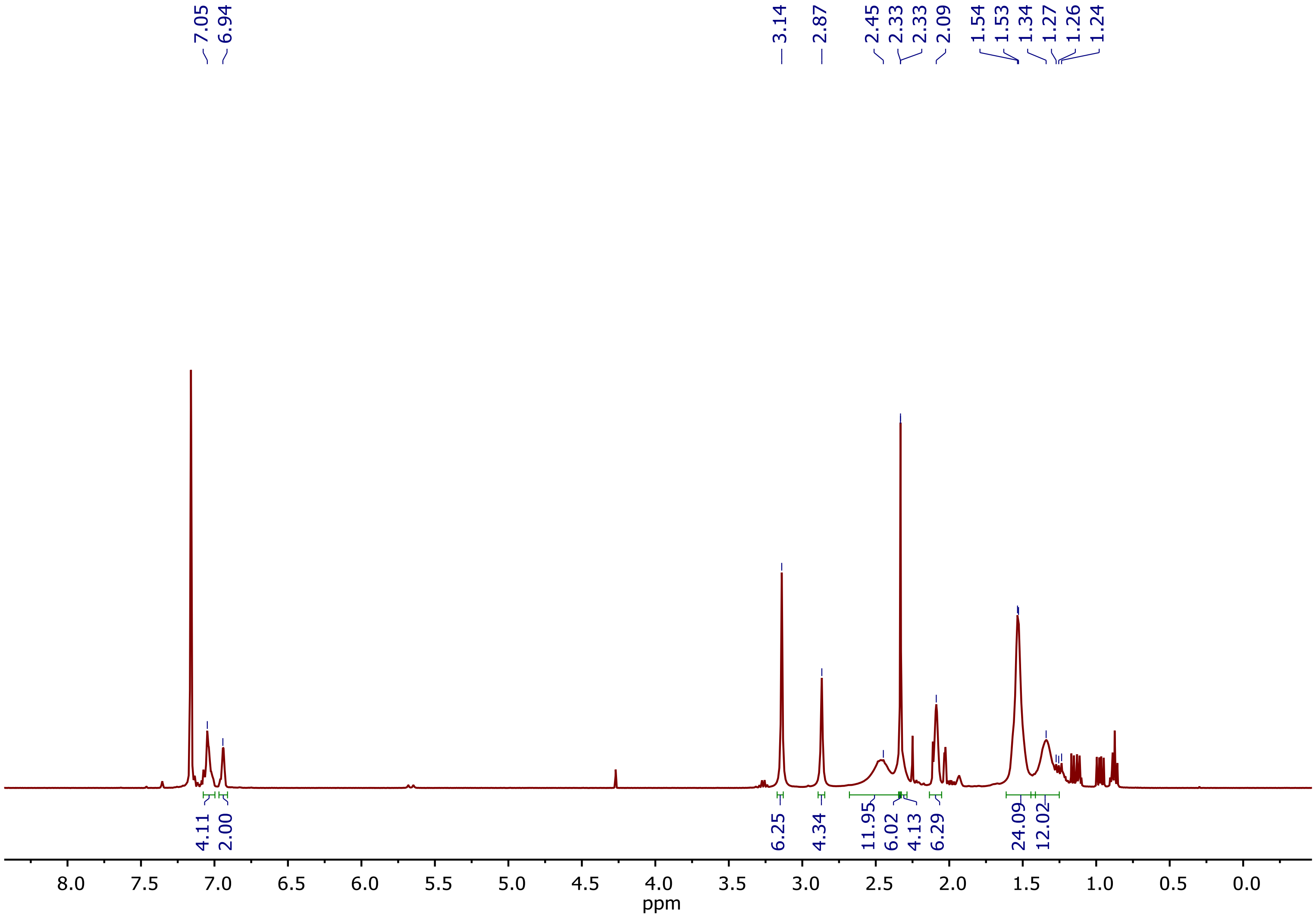

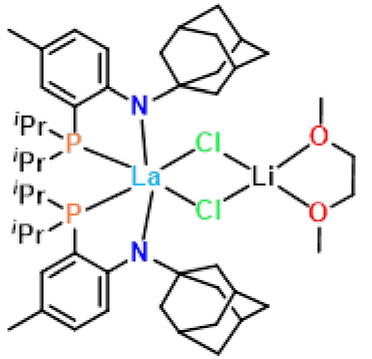

Figure S 176: ^{1}H NMR spectrum of **4a** in C_6D_6 at 298 K.

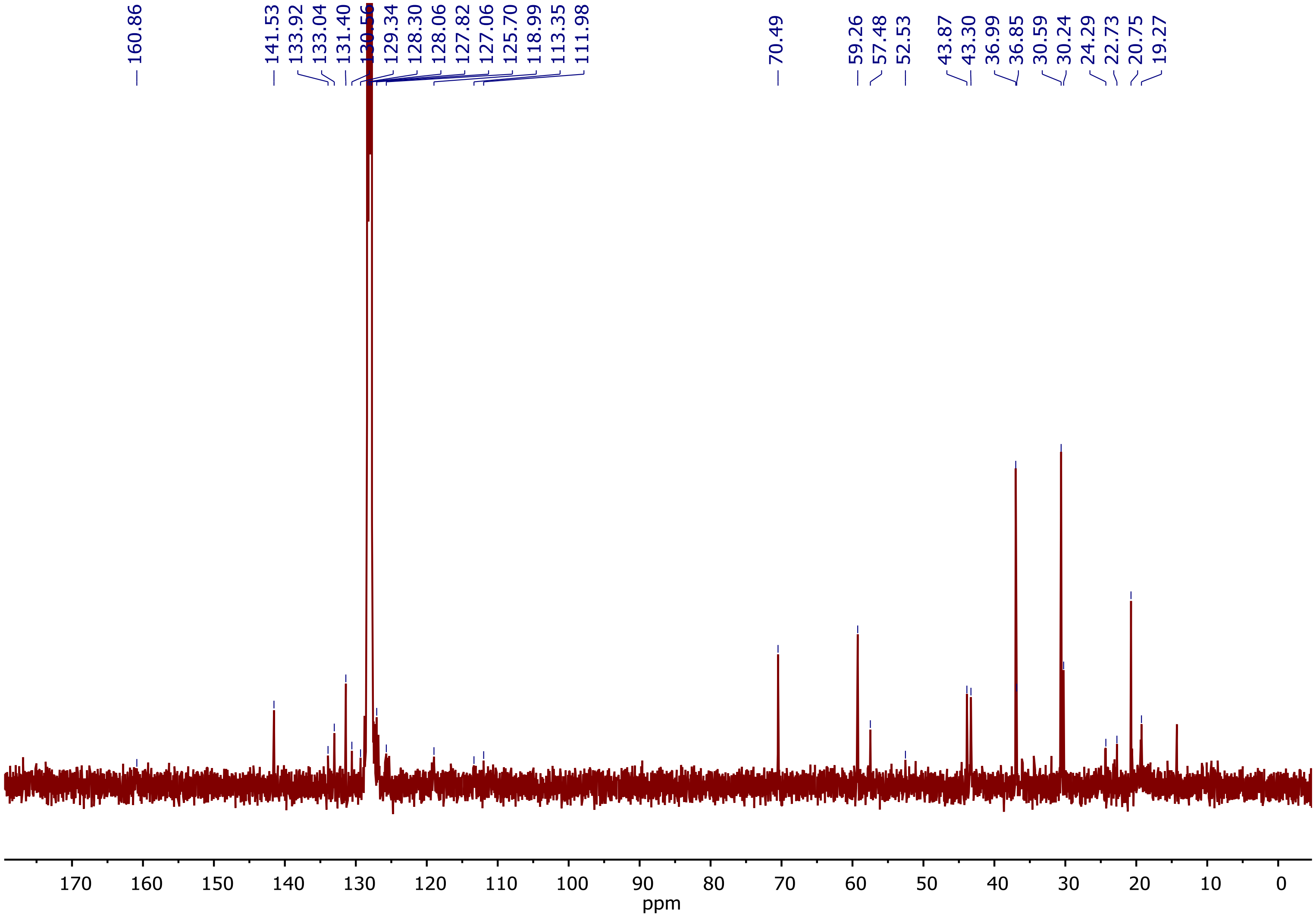

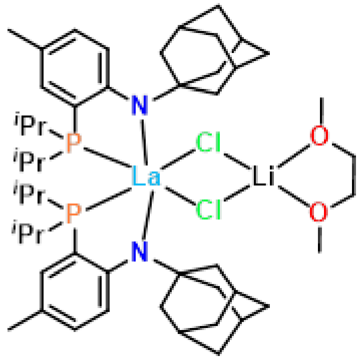

*Figure S 177: $^{13}C\{^{1}H\}$ NMR spectrum of **4a** in C_6D_6 at 298 K.*

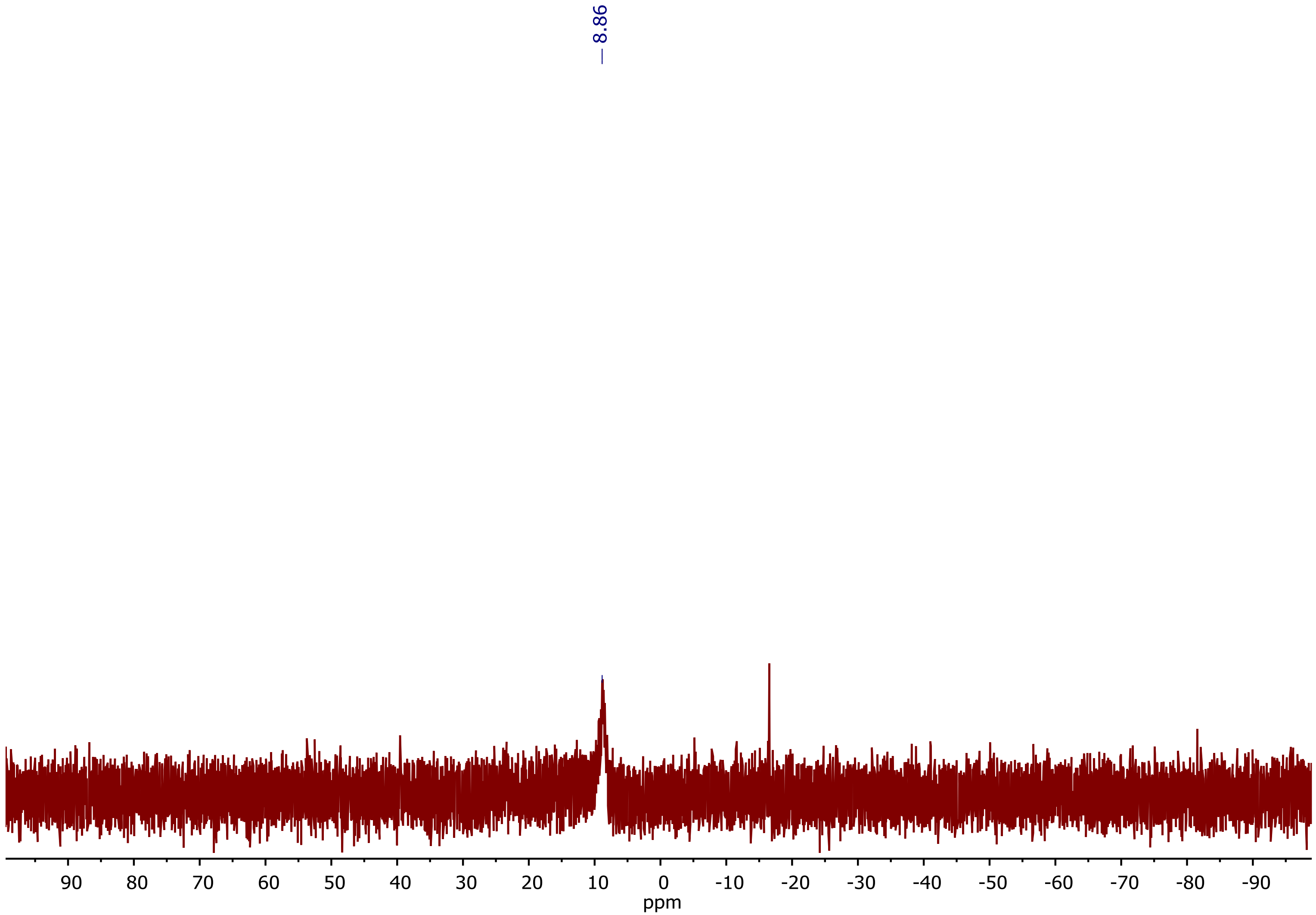

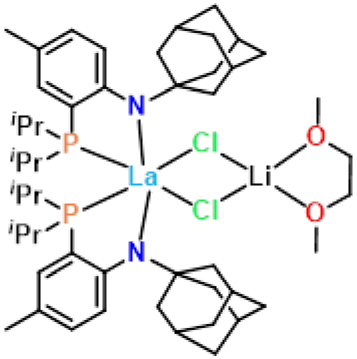

*Figure S 178: $^{31}P\{^{1}H\}$ NMR spectrum of **4a** in C_6D_6 at 298 K.*

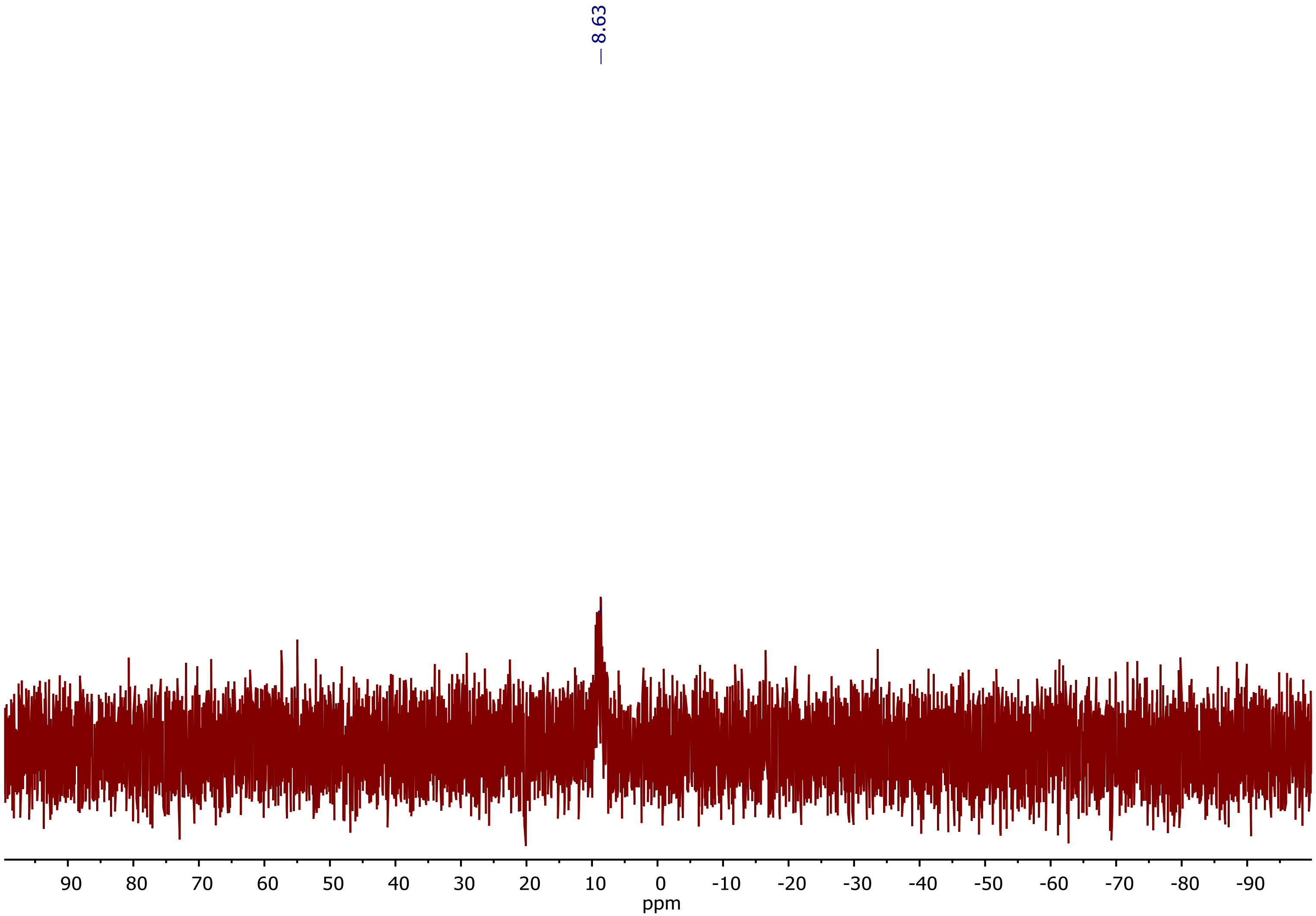

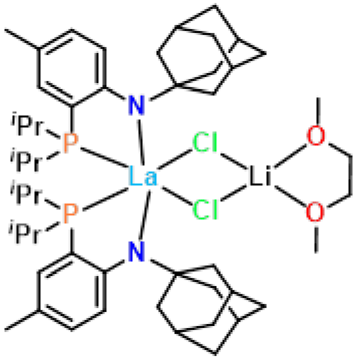

*Figure S 179: ^{31}P NMR spectrum of **4a** in C_6D_6 at 298 K.*

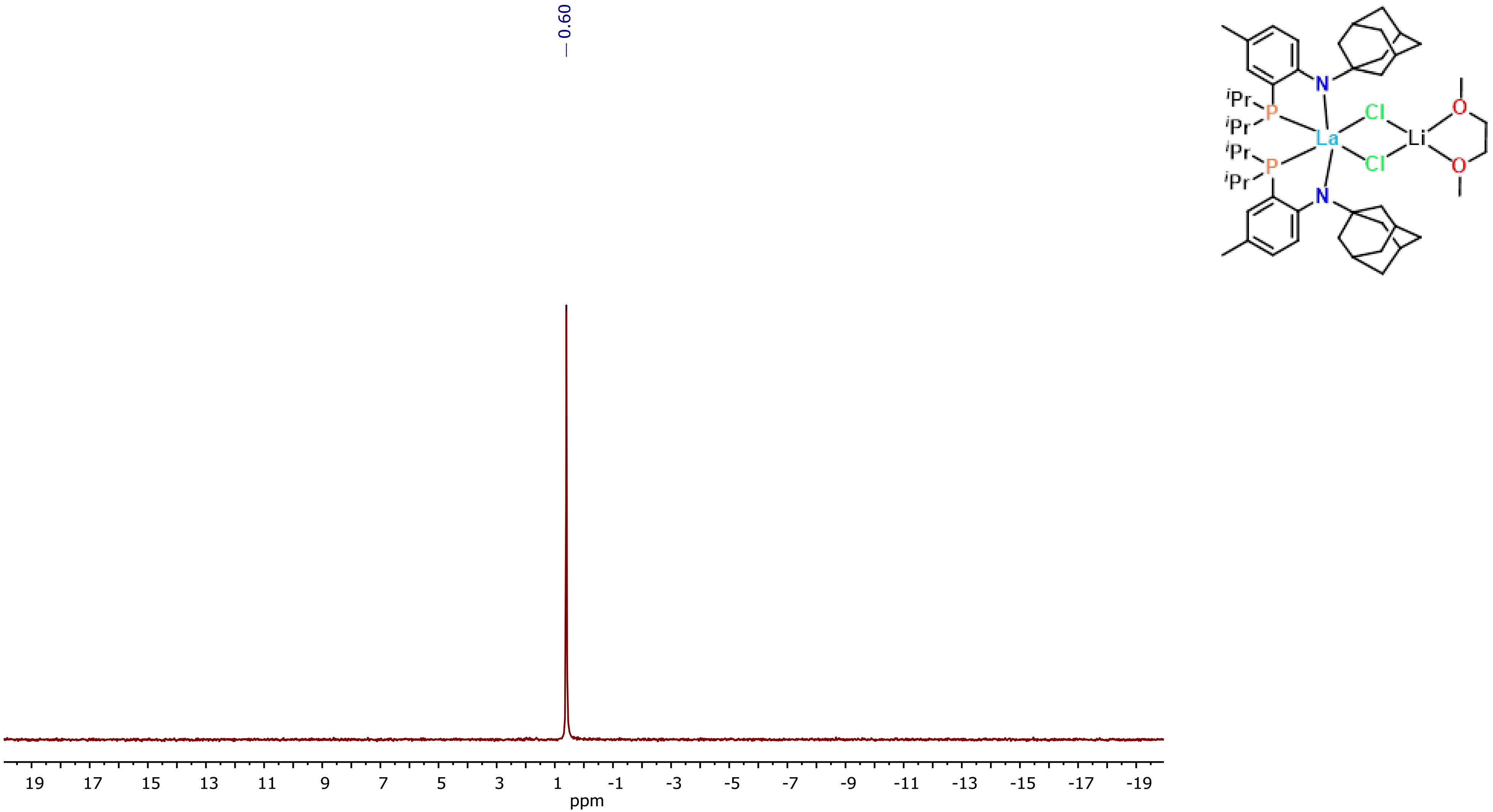

*Figure S 180: ^{7}Li{^{1}H} NMR spectrum of **4a** in C_6D_6 at 298 K.*

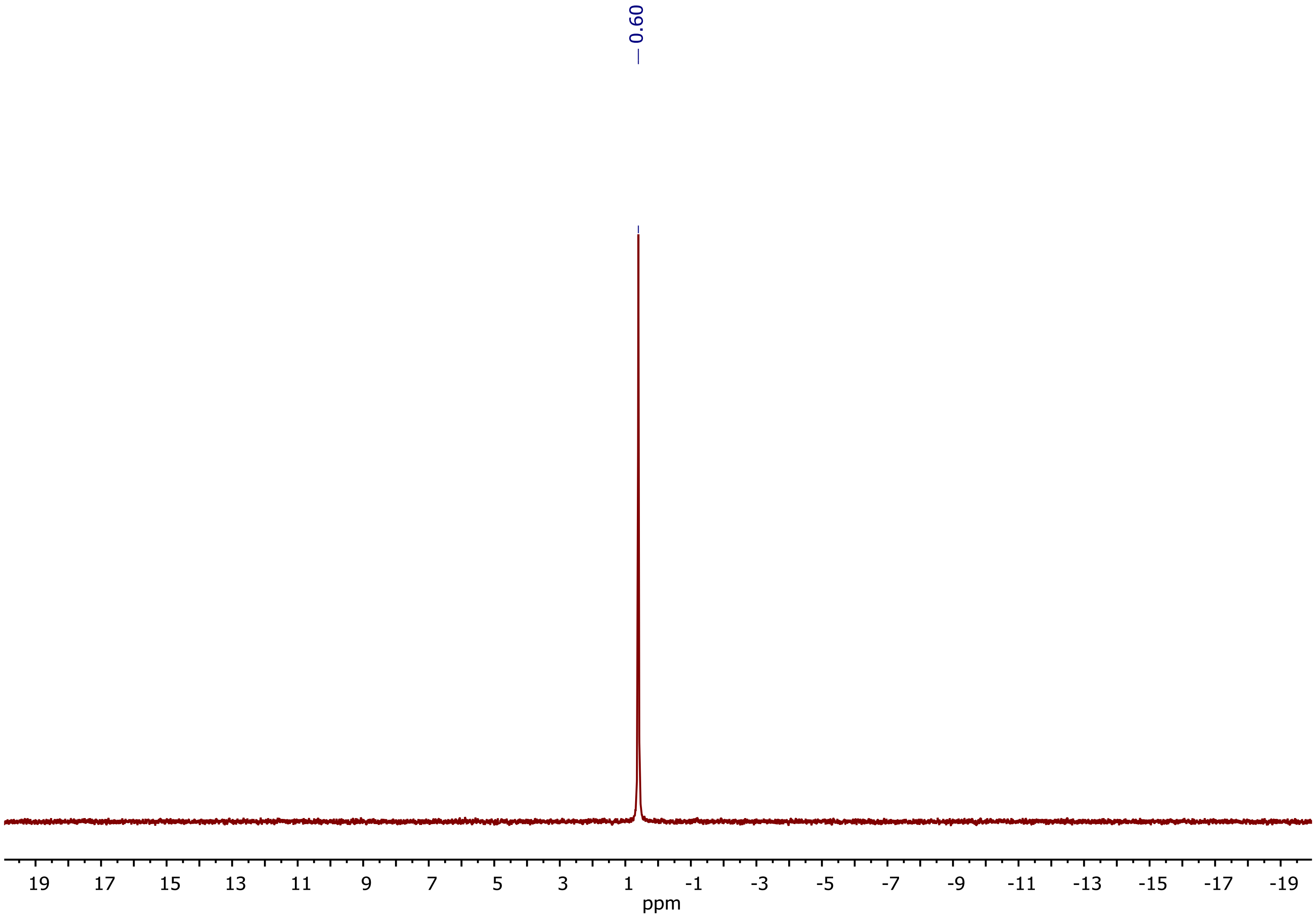

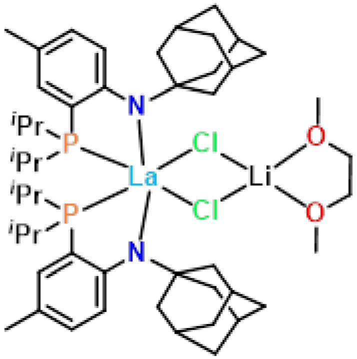

*Figure S 181: ^{7}Li NMR spectrum of **4a** in C_6D_6 at 298 K.*

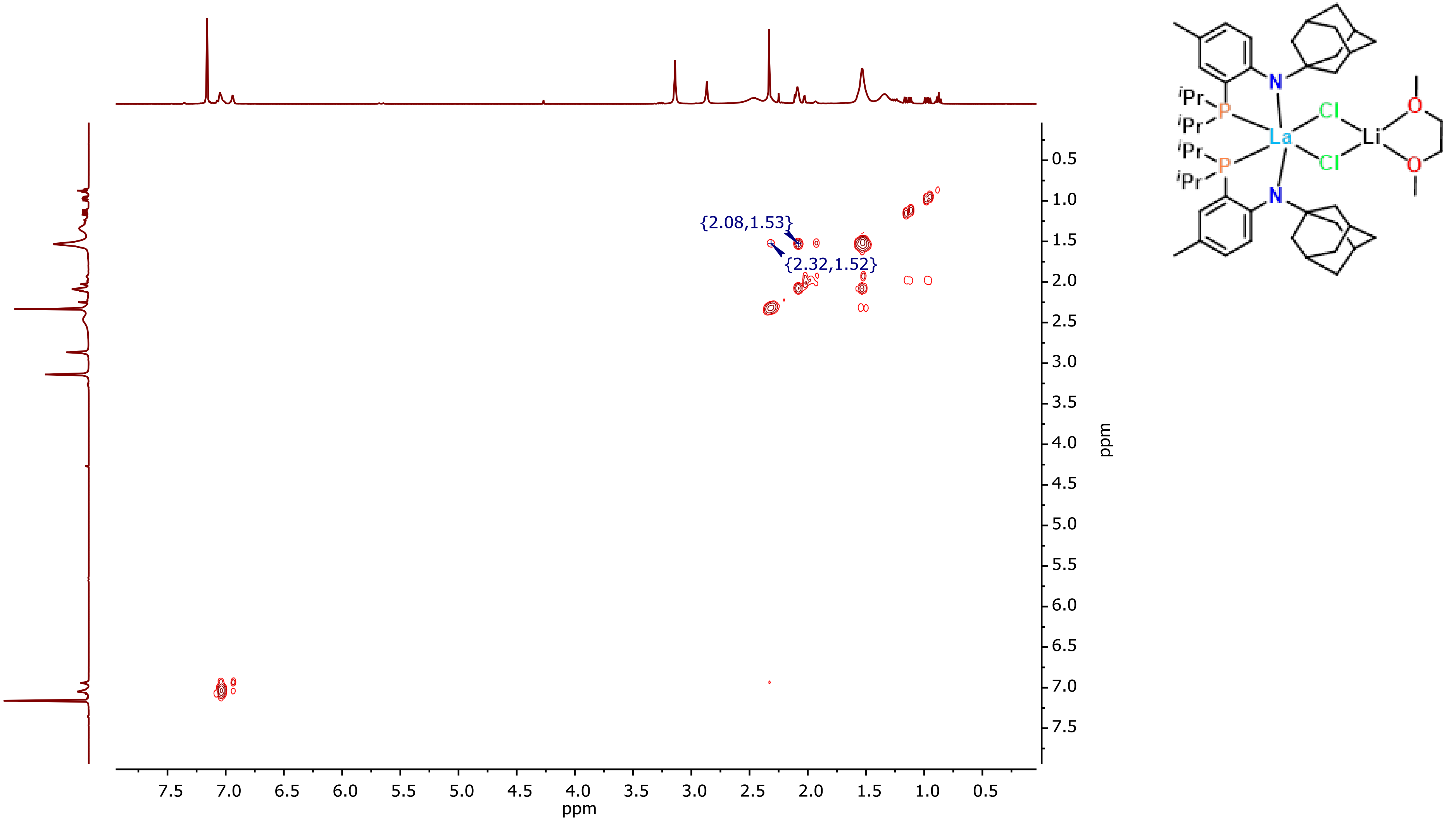

*Figure S 182: ^{1}H-^{1}H COSY NMR spectrum of **4a** in C_6D_6 at 298 K.*

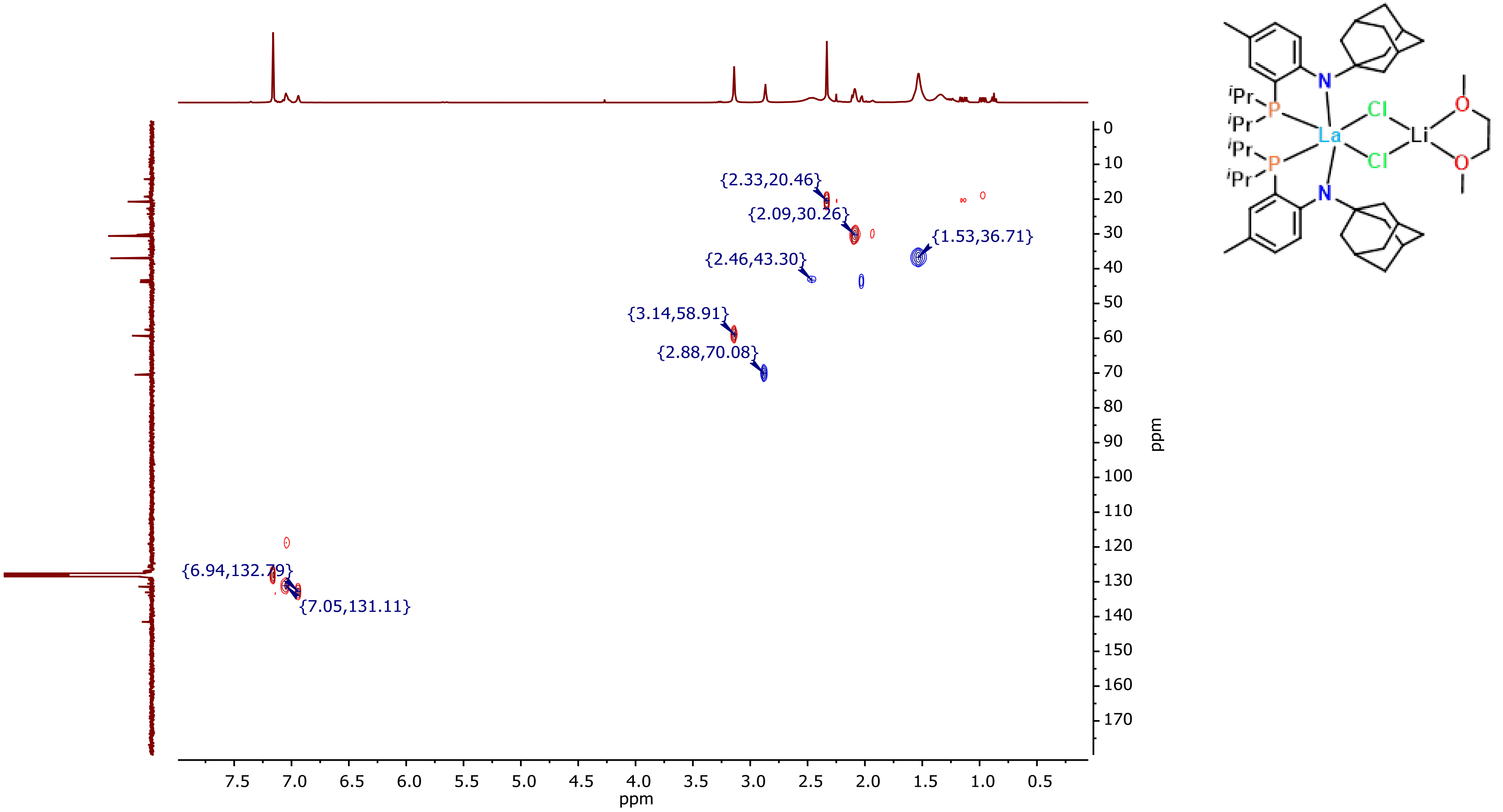

*Figure S 183: ^{1}H-^{13}C HSQC NMR spectrum of **4a** in C_6D_6 at 298 K.*

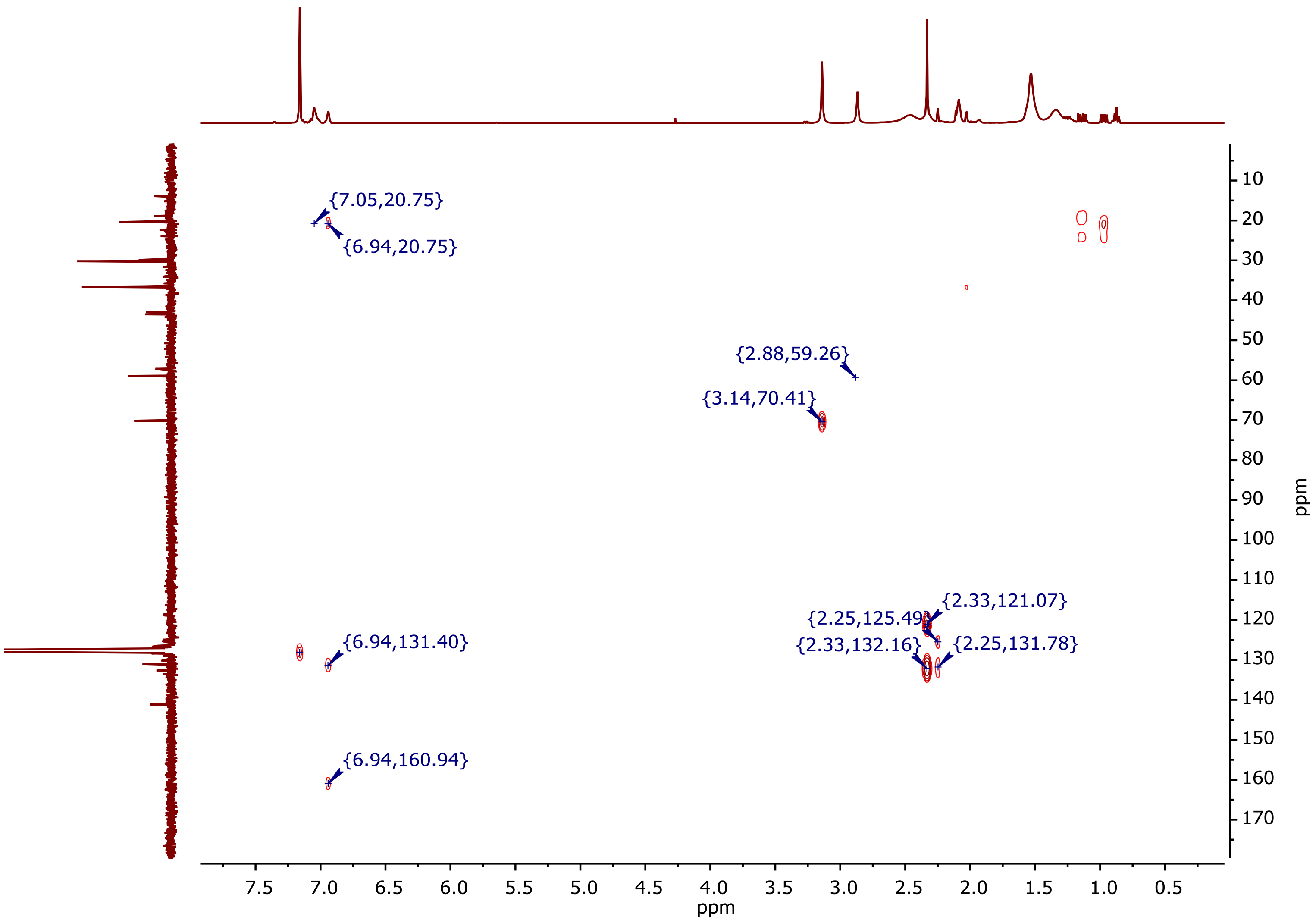

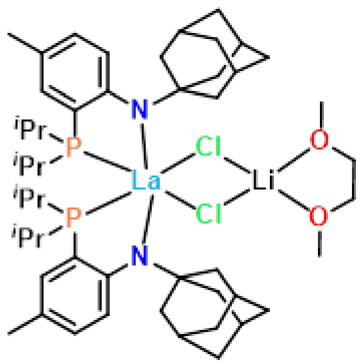

*Figure S 184: ^{1}H-^{13}C HMBC NMR spectrum of **4a** in C_6D_6 at 298 K.*

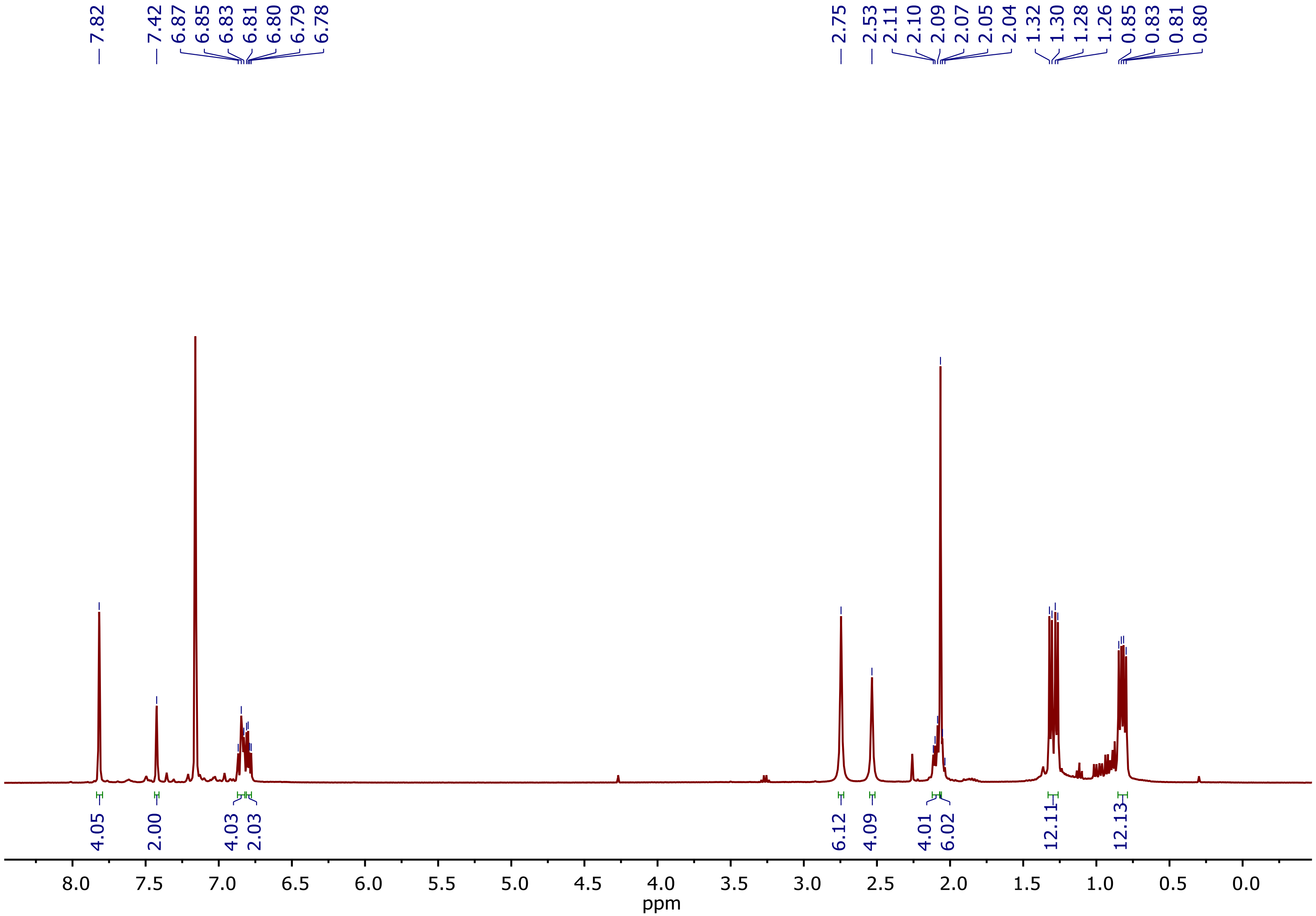

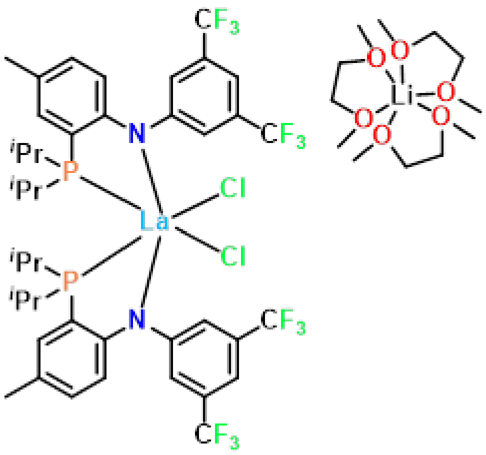

*Figure S 185: ^{1}H NMR spectrum of **4b** in C_6D_6 at 298 K.*

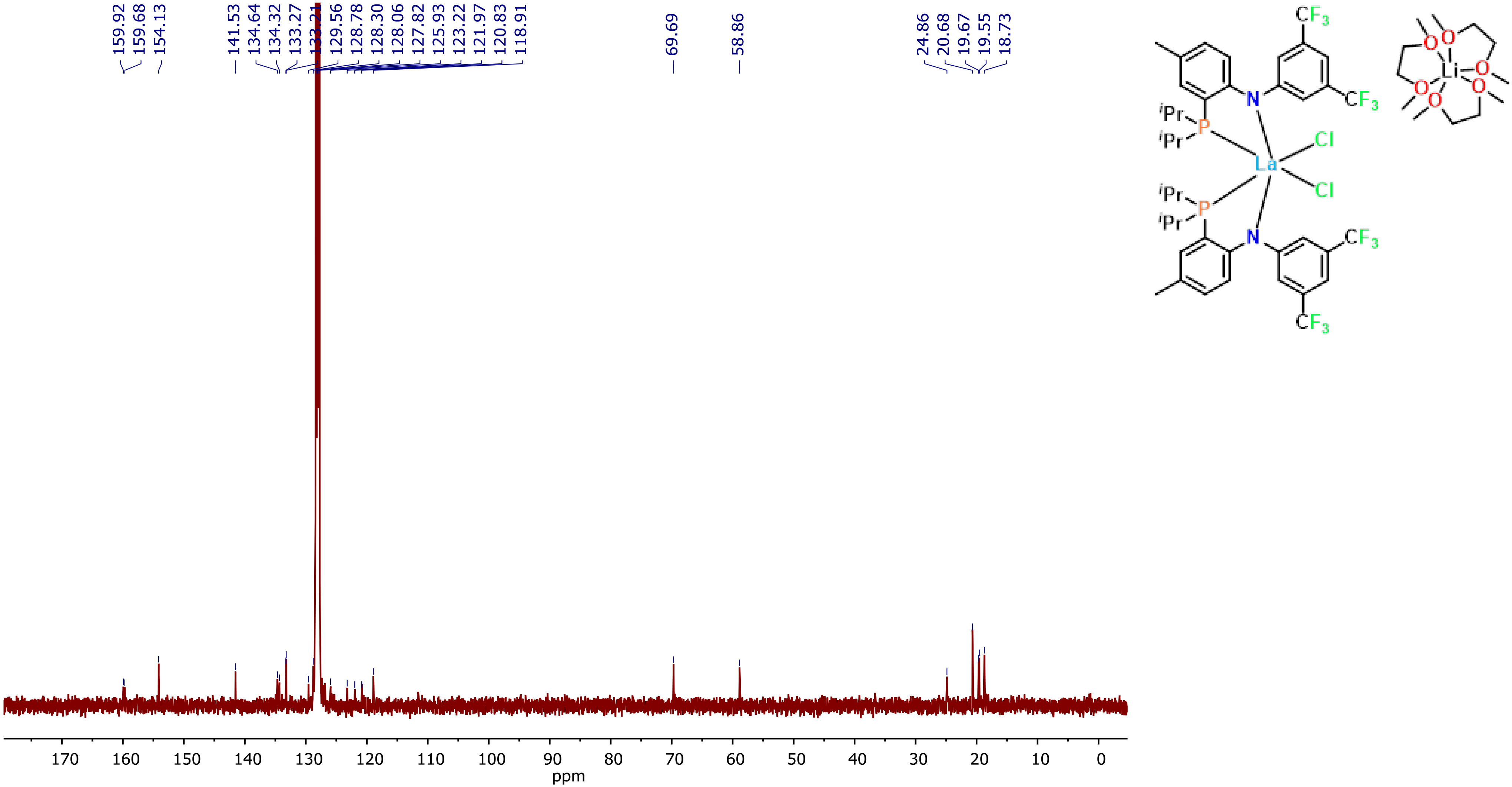

*Figure S 186: $^{13}C\{^{1}H\}$ NMR spectrum of **4b** in C_6D_6 at 298 K.*

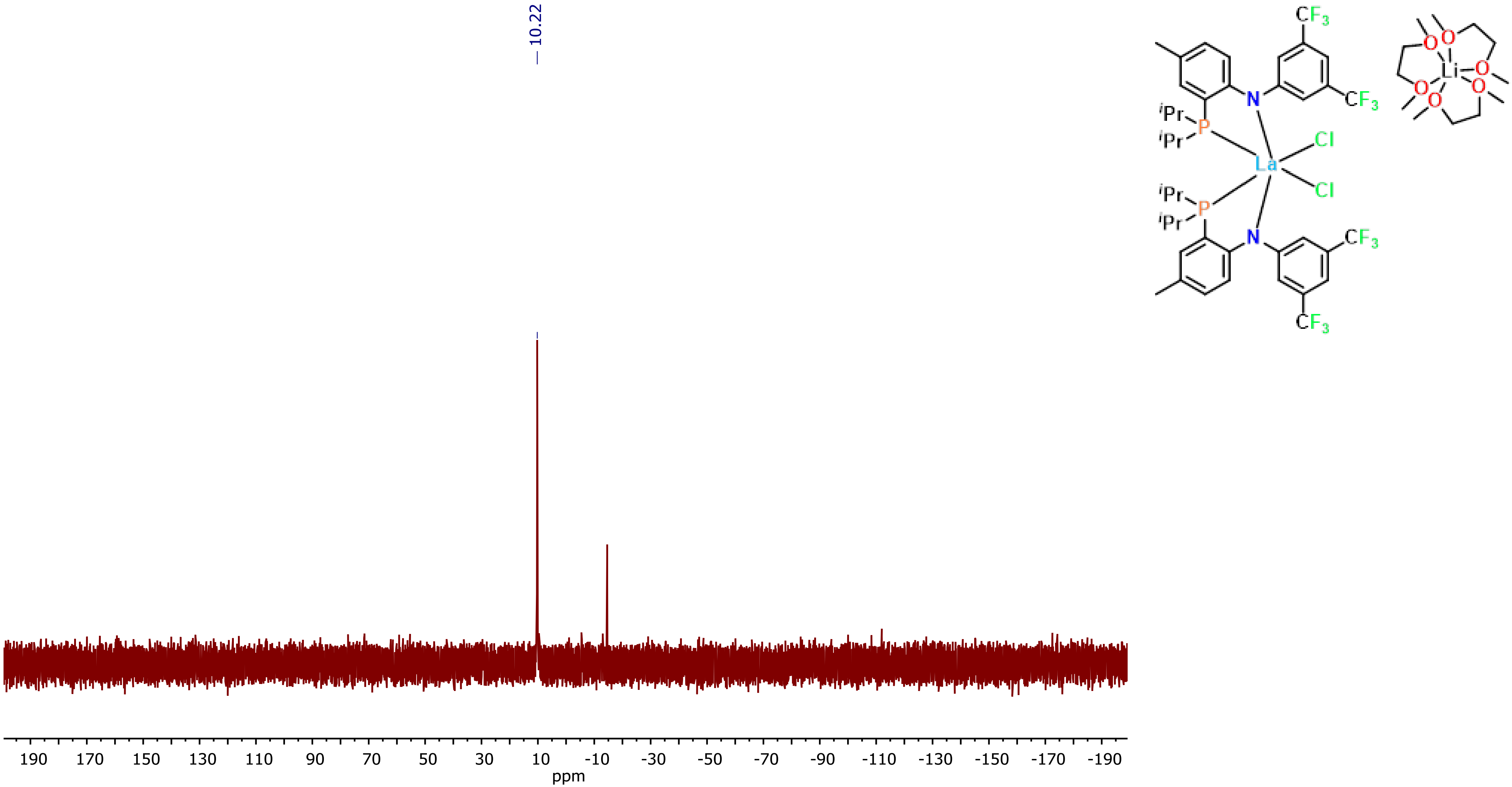

*Figure S 187: $^{31}P\{^{1}H\}$ NMR spectrum of **4b** in C_6D_6 at 298 K.*

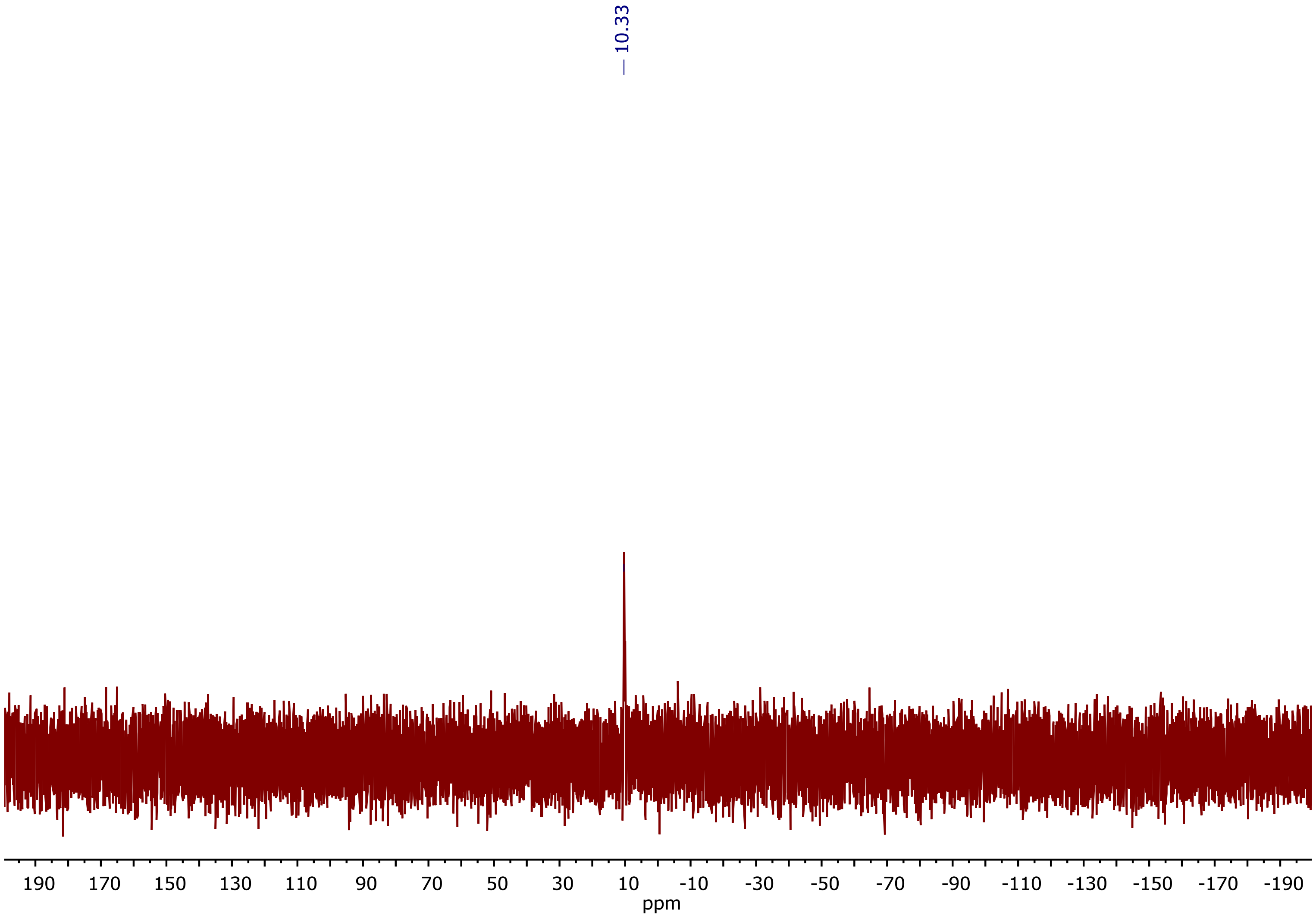

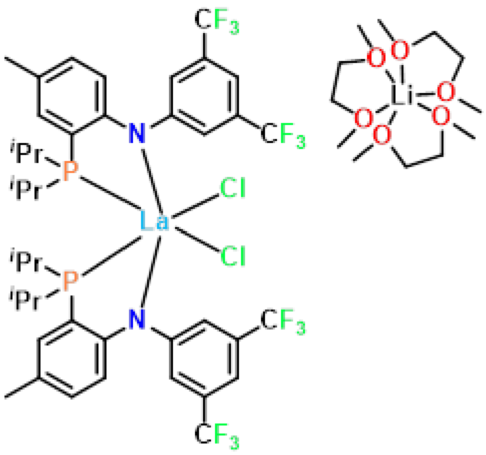

*Figure S 188: ^{31}P NMR spectrum of **4b** in C_6D_6 at 298 K.*

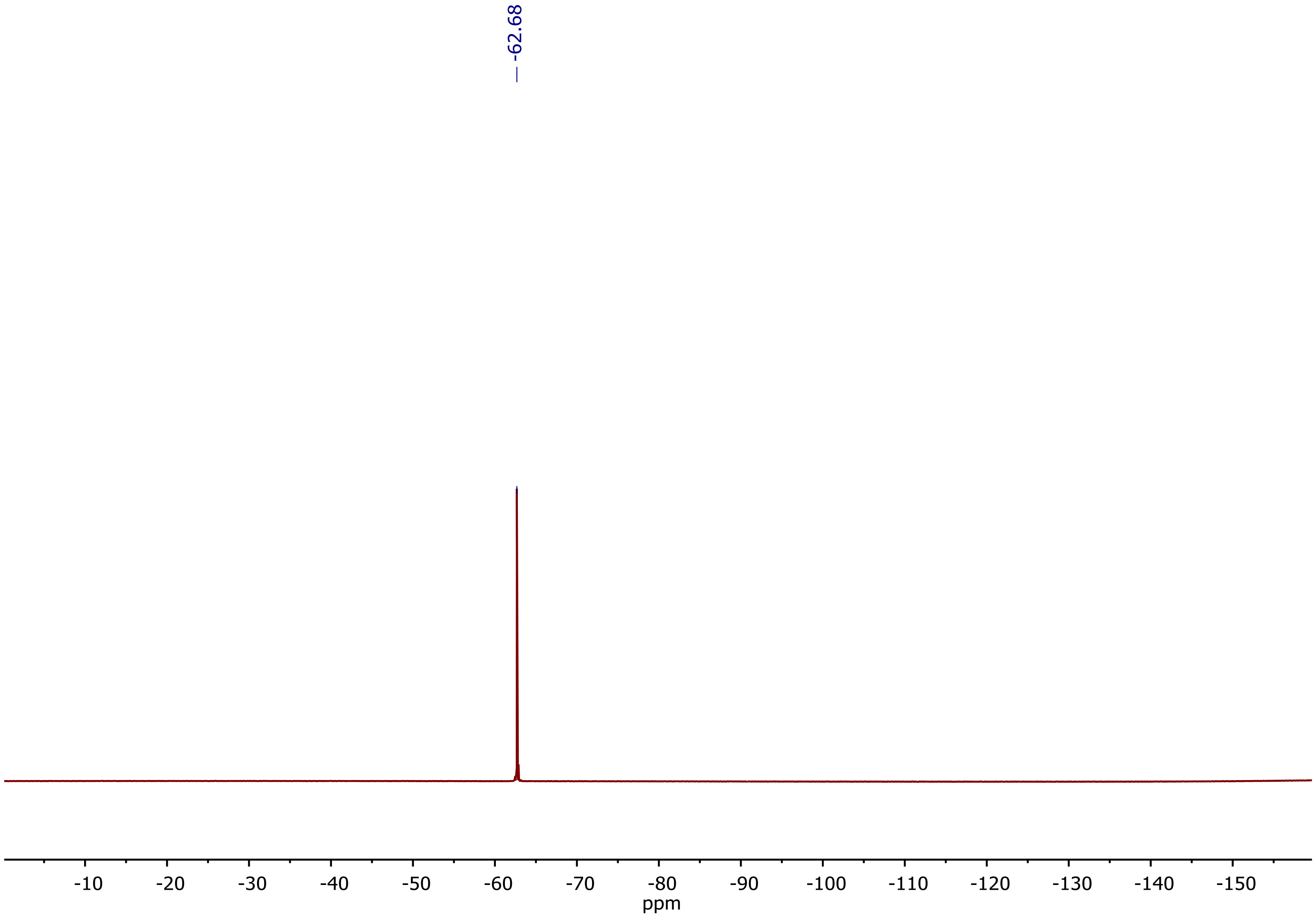

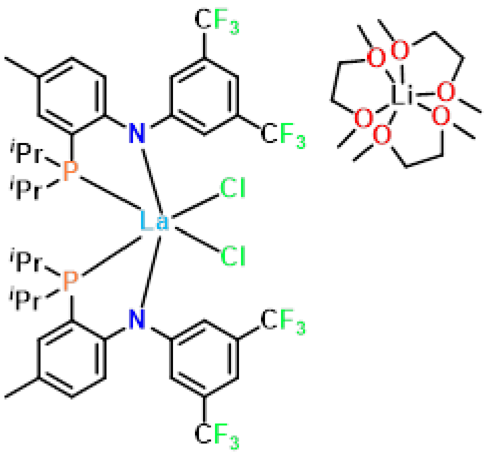

Figure S 189: ^{19}F NMR spectrum of **4b** in C_6D_6 at 298 K.

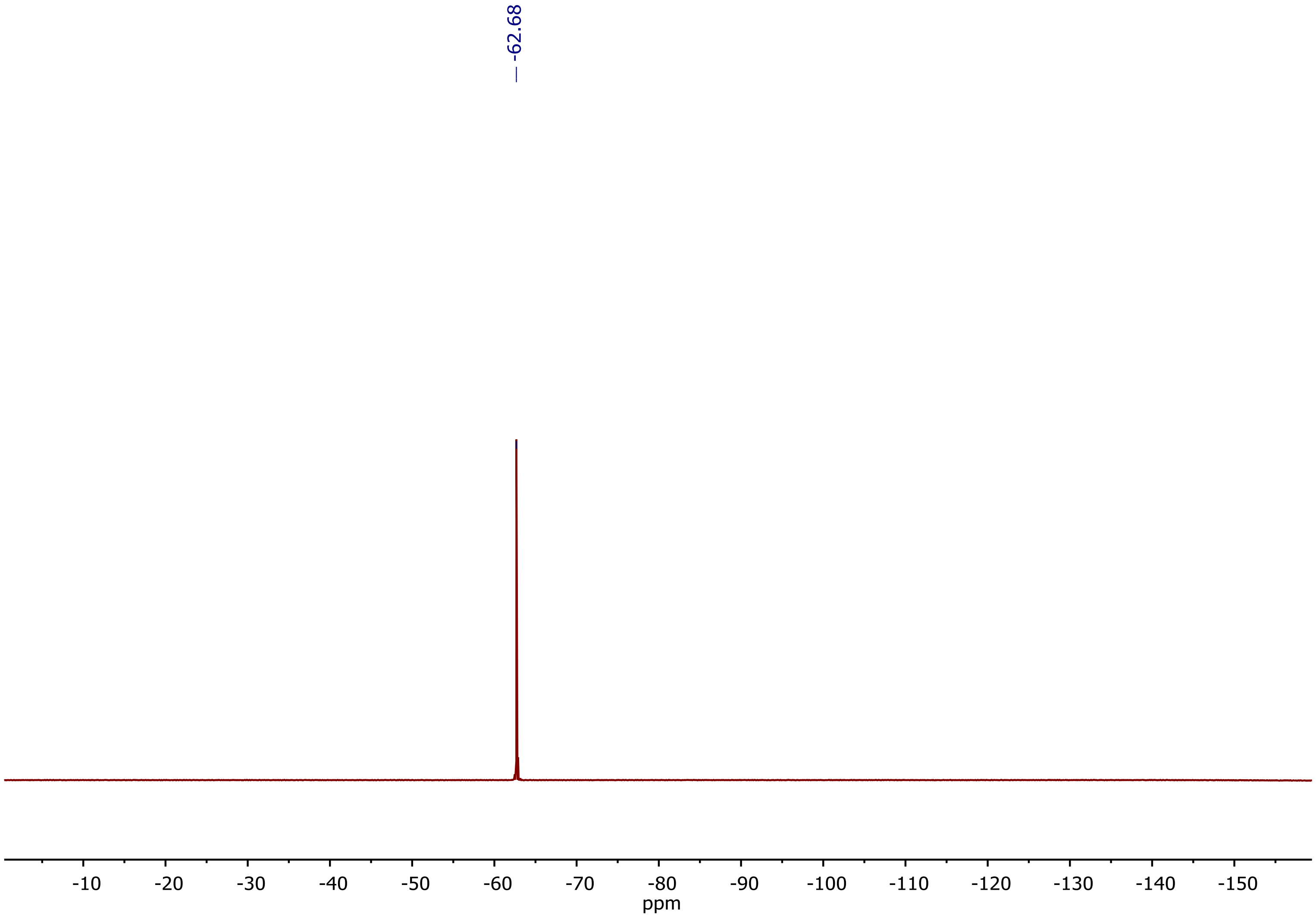

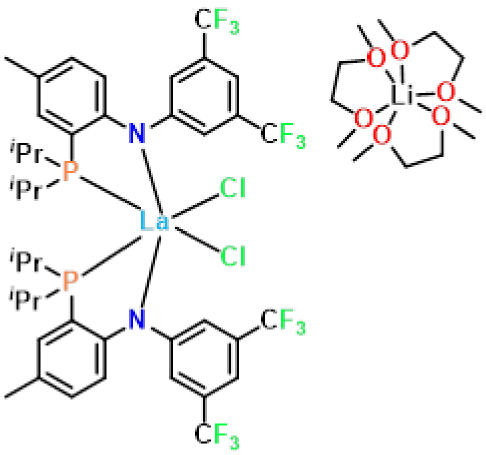

*Figure S 190: $^{19}F\{^{1}H\}$ NMR spectrum of **4b** in C_6D_6 at 298 K.*

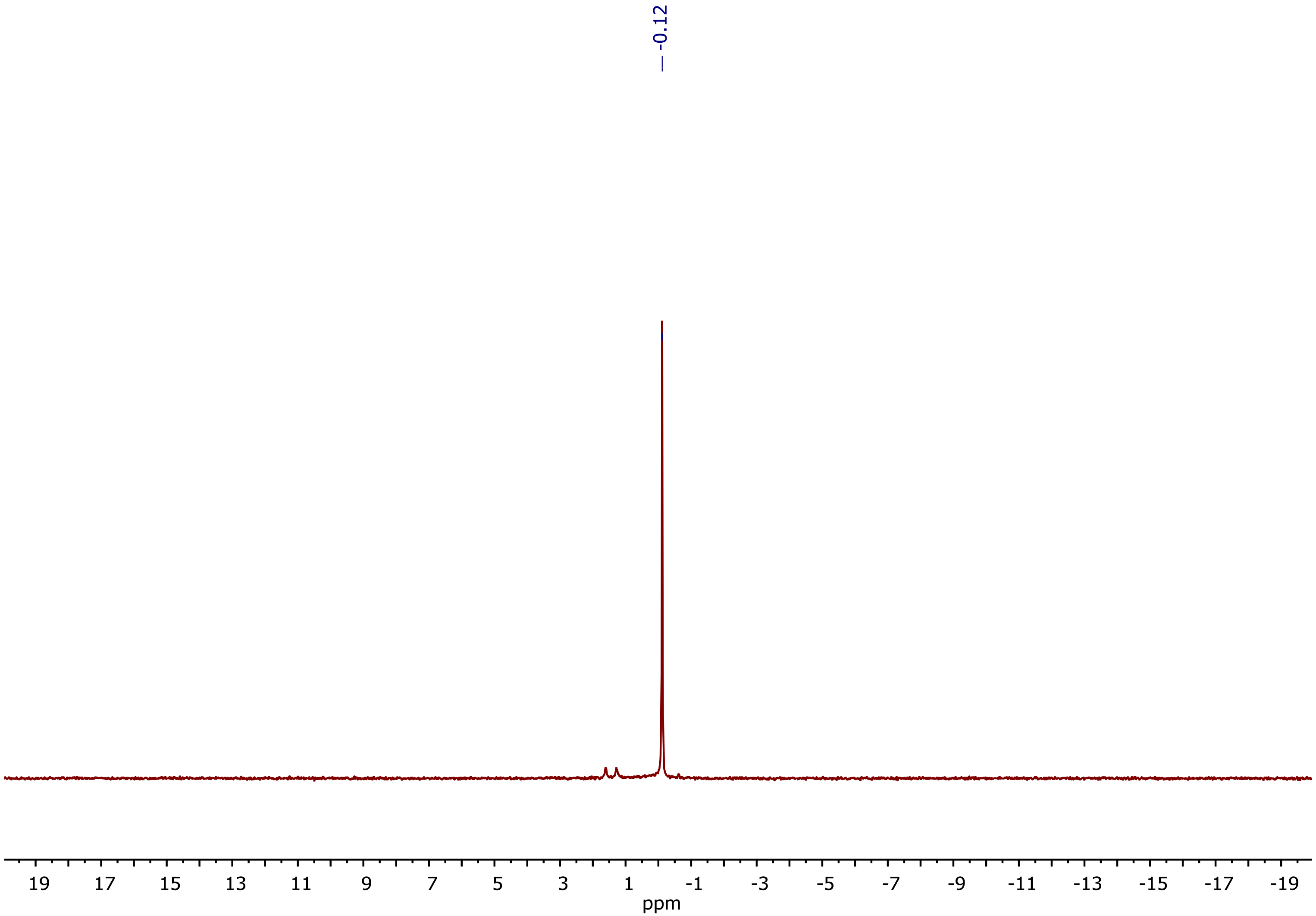

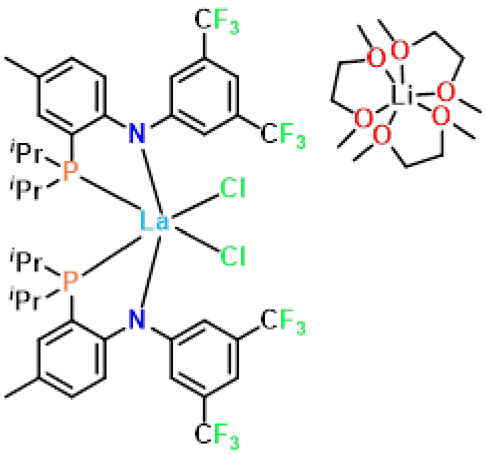

*Figure S 191: $^{7}Li\{^{1}H\}$ NMR spectrum of **4b** in C_6D_6 at 298 K.*

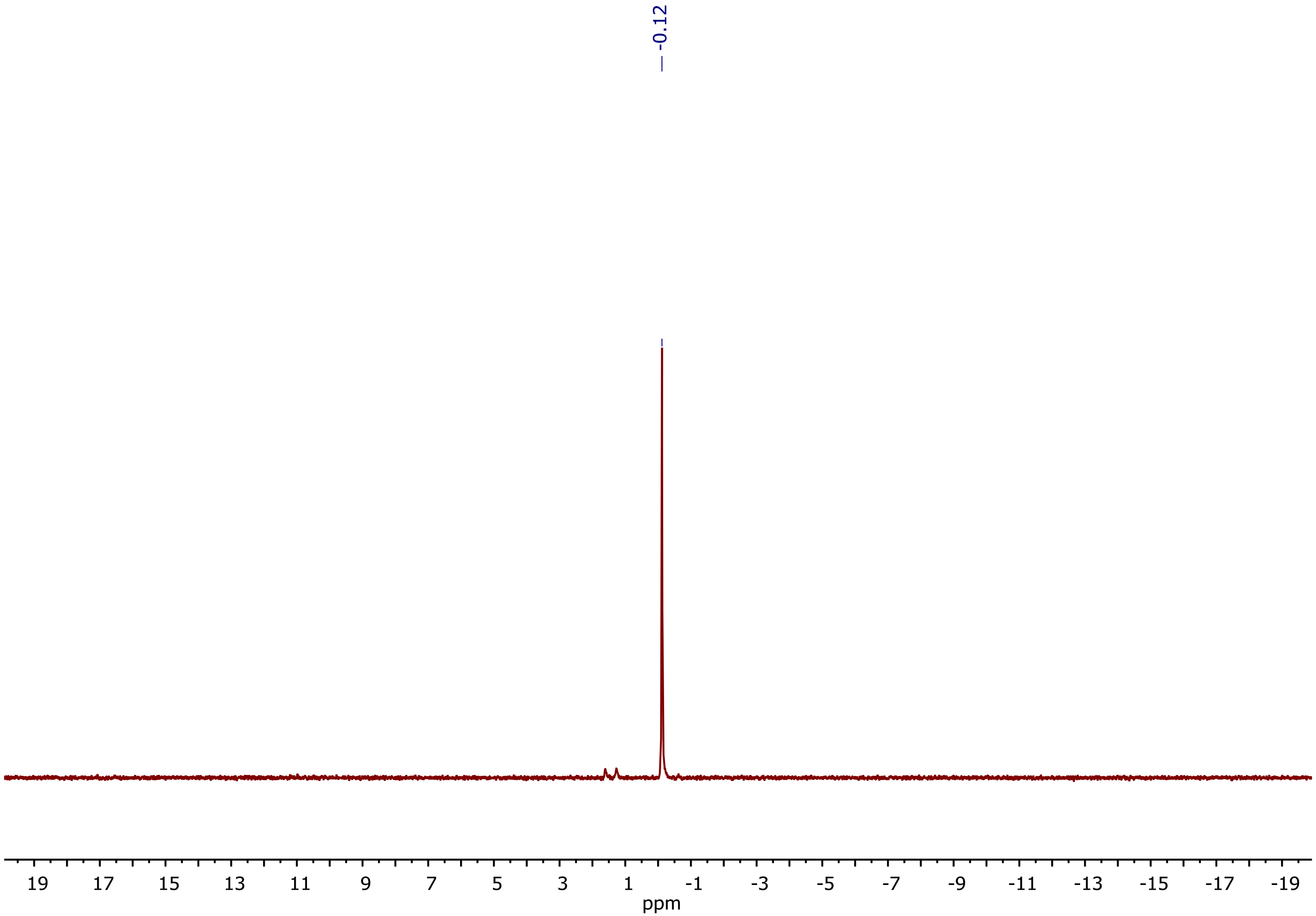

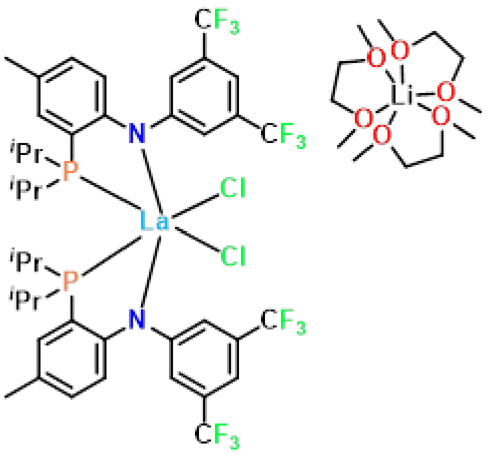

Figure S 192: ^{7}Li NMR spectrum of **4b** in C_6D_6 at 298 K.

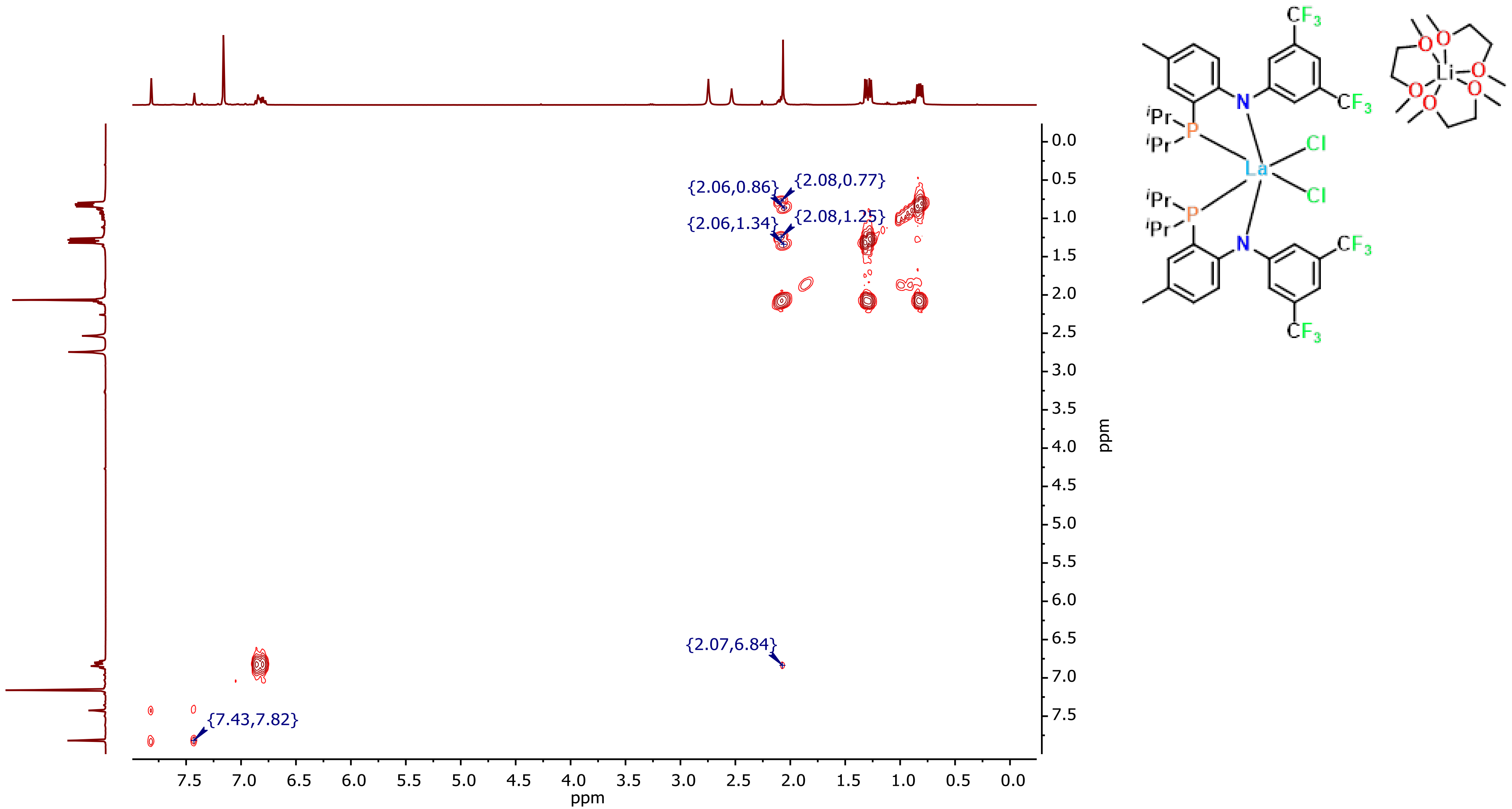

*Figure S 193: ^{1}H-^{1}H COSY NMR spectrum of **4b** in C_6D_6 at 298 K.*

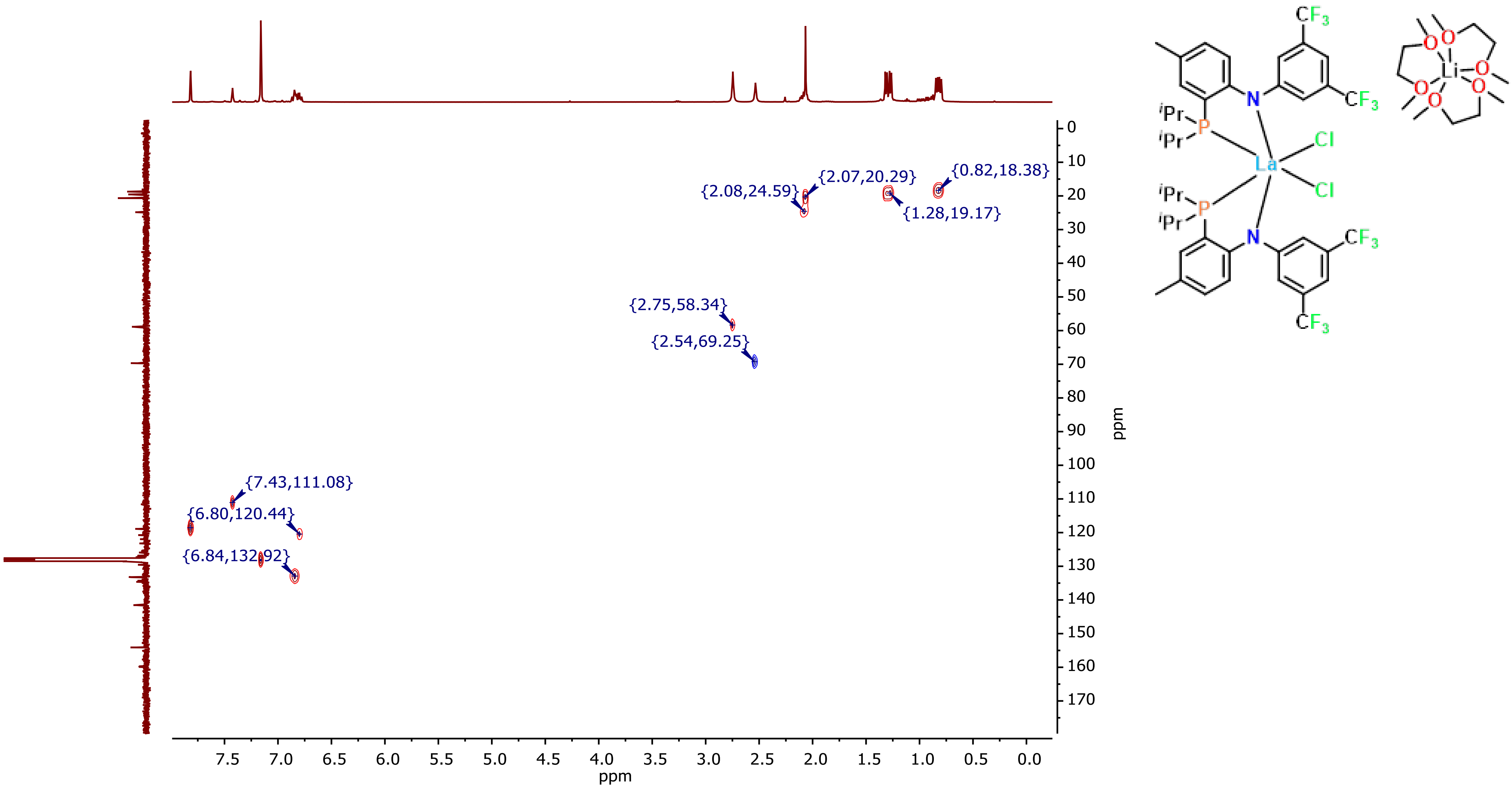

Figure S 194: ^{1}H-^{13}C HSQC NMR spectrum of **4b** in C_6D_6 at 298 K.

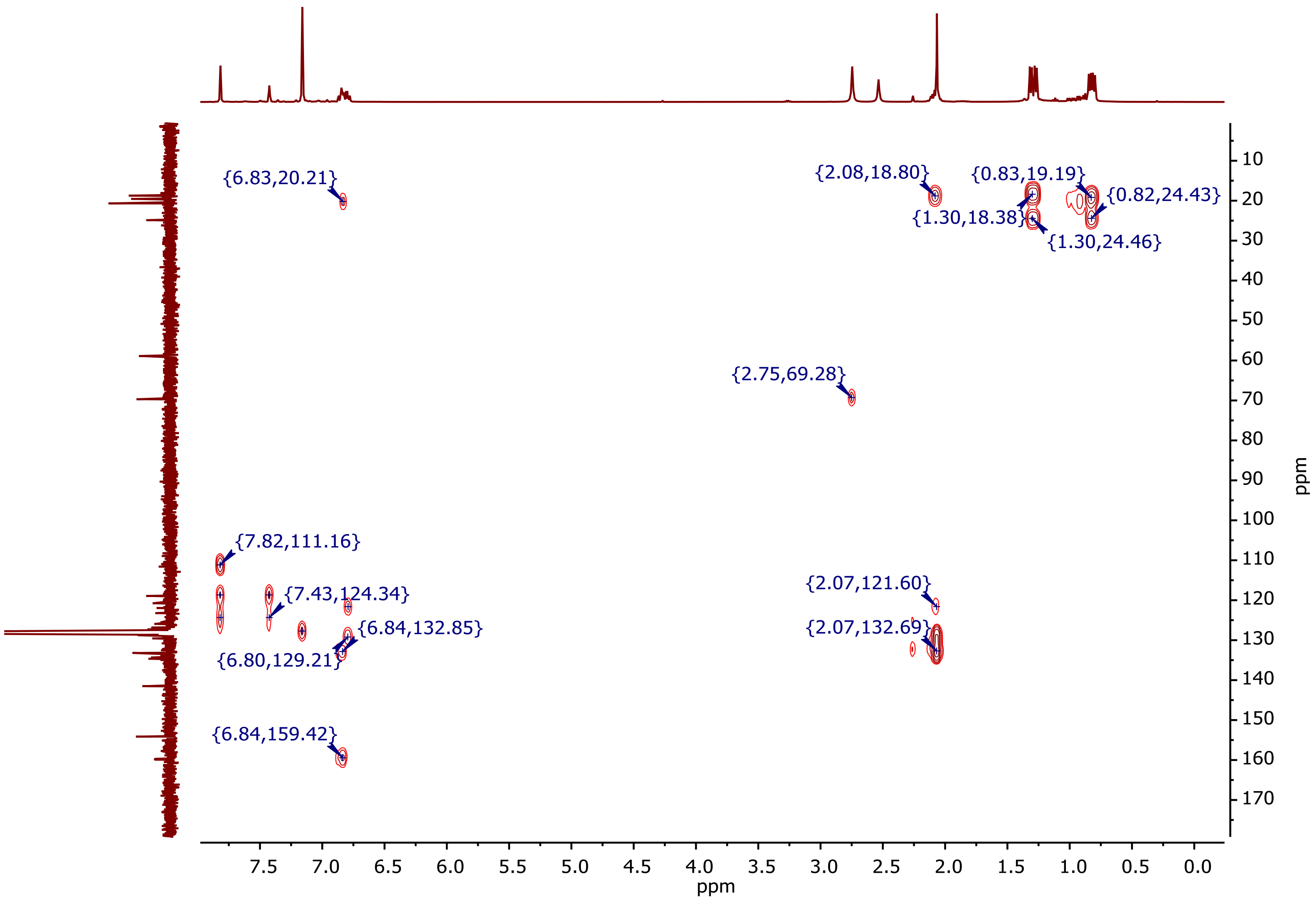

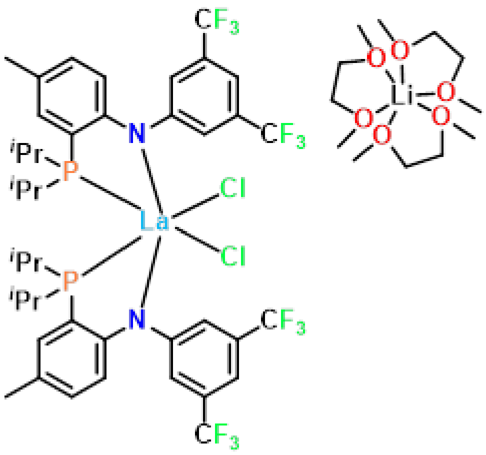

*Figure S 195: ^{1}H-^{13}C HMBC NMR spectrum of **4b** in C_6D_6 at 298 K.*

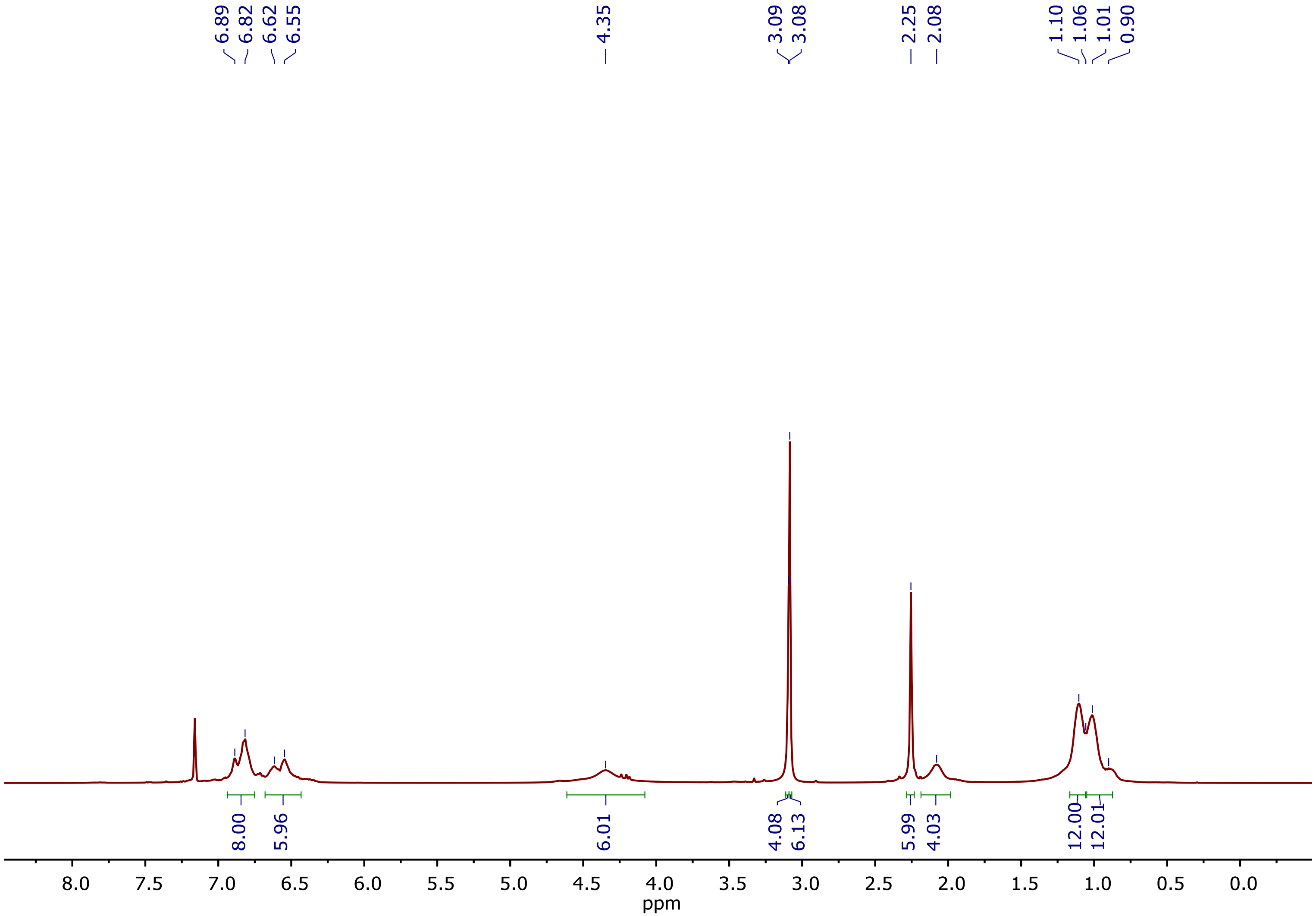

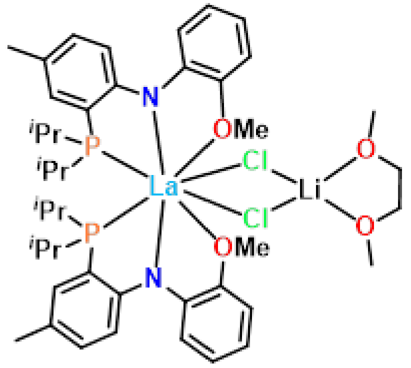

Figure S 196: ^{1}H NMR spectrum of **4c** in C_6D_6 at 298 K.

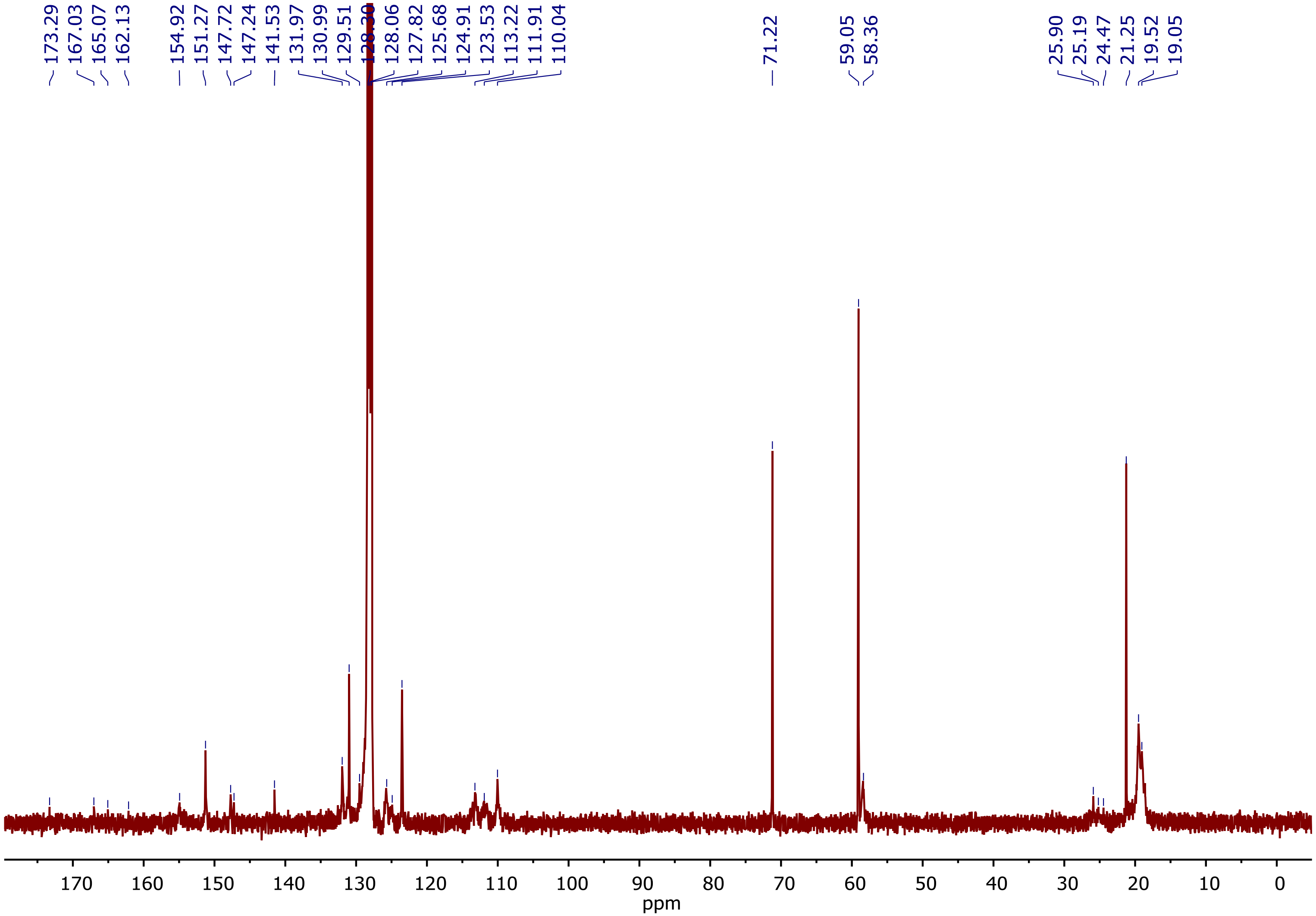

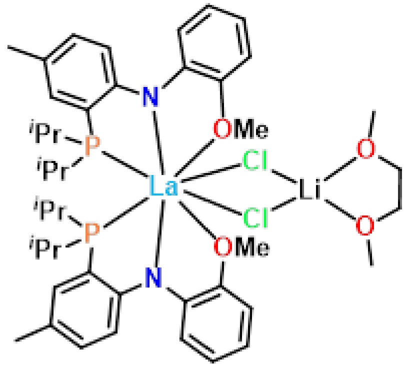

*Figure S 197: $^{13}C\{^{1}H\}$ NMR spectrum of **4c** in C_6D_6 at 298 K.*

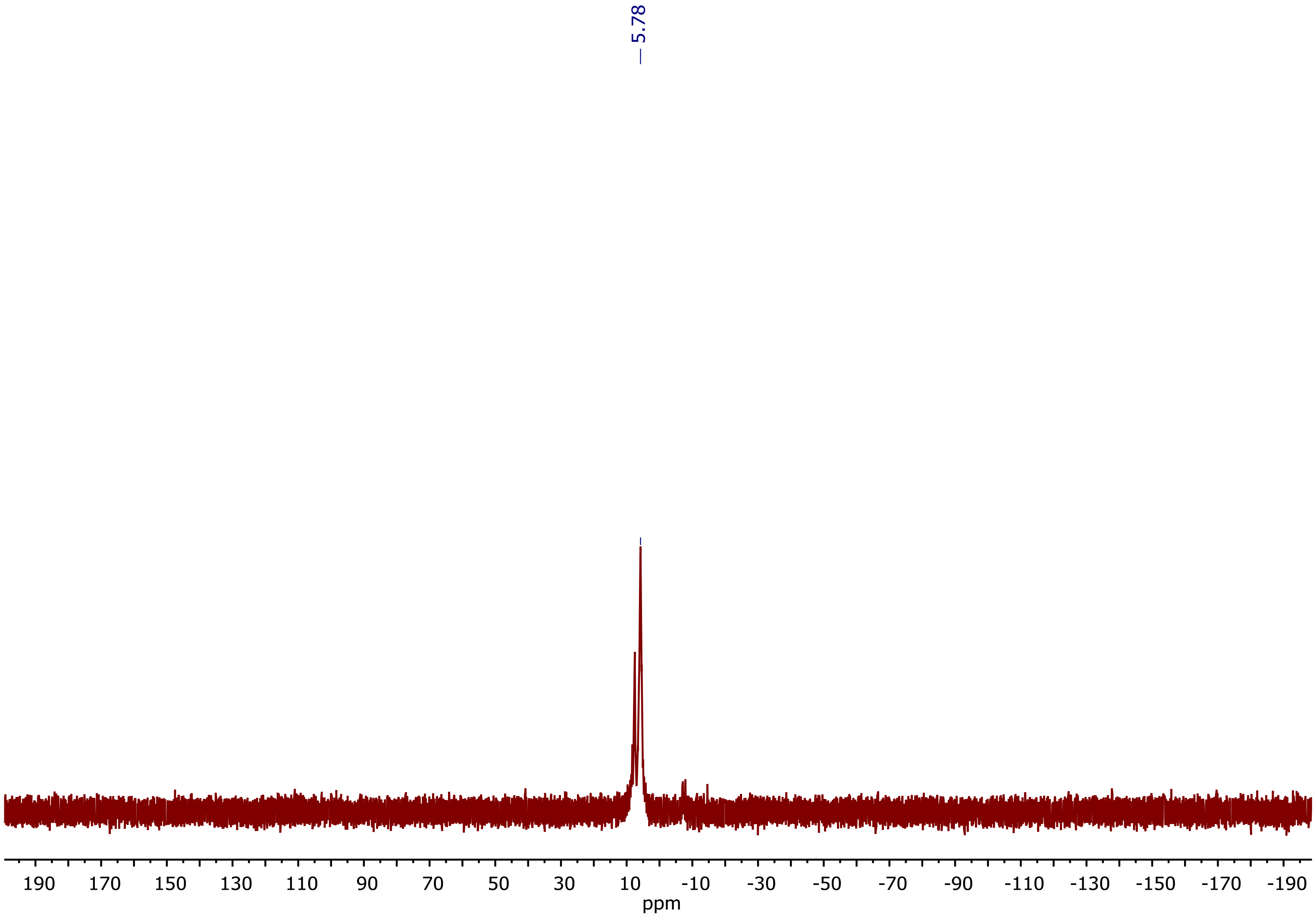

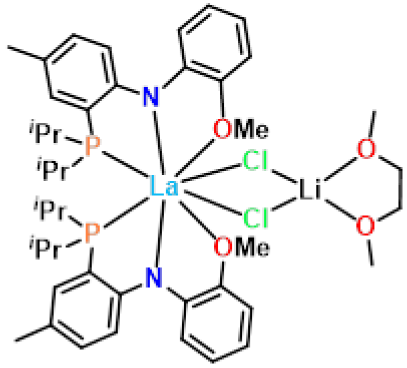

*Figure S 198: $^{31}P\{^1H\}$ NMR spectrum of **4c** in C_6D_6 at 298 K.*

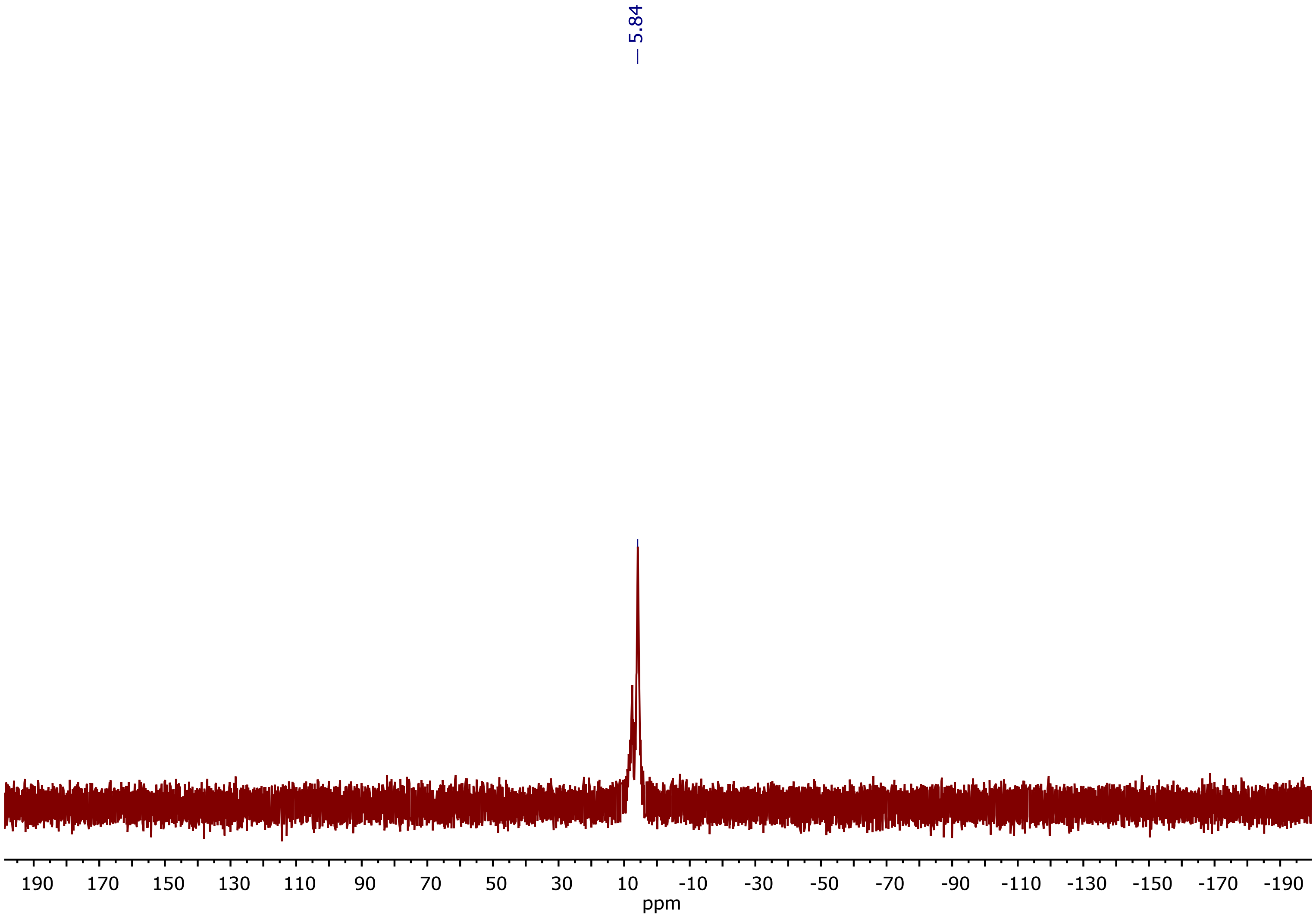

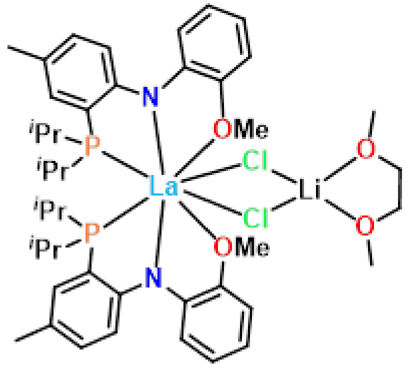

*Figure S 199: ^{31}P NMR spectrum of **4c** in C_6D_6 at 298 K.*

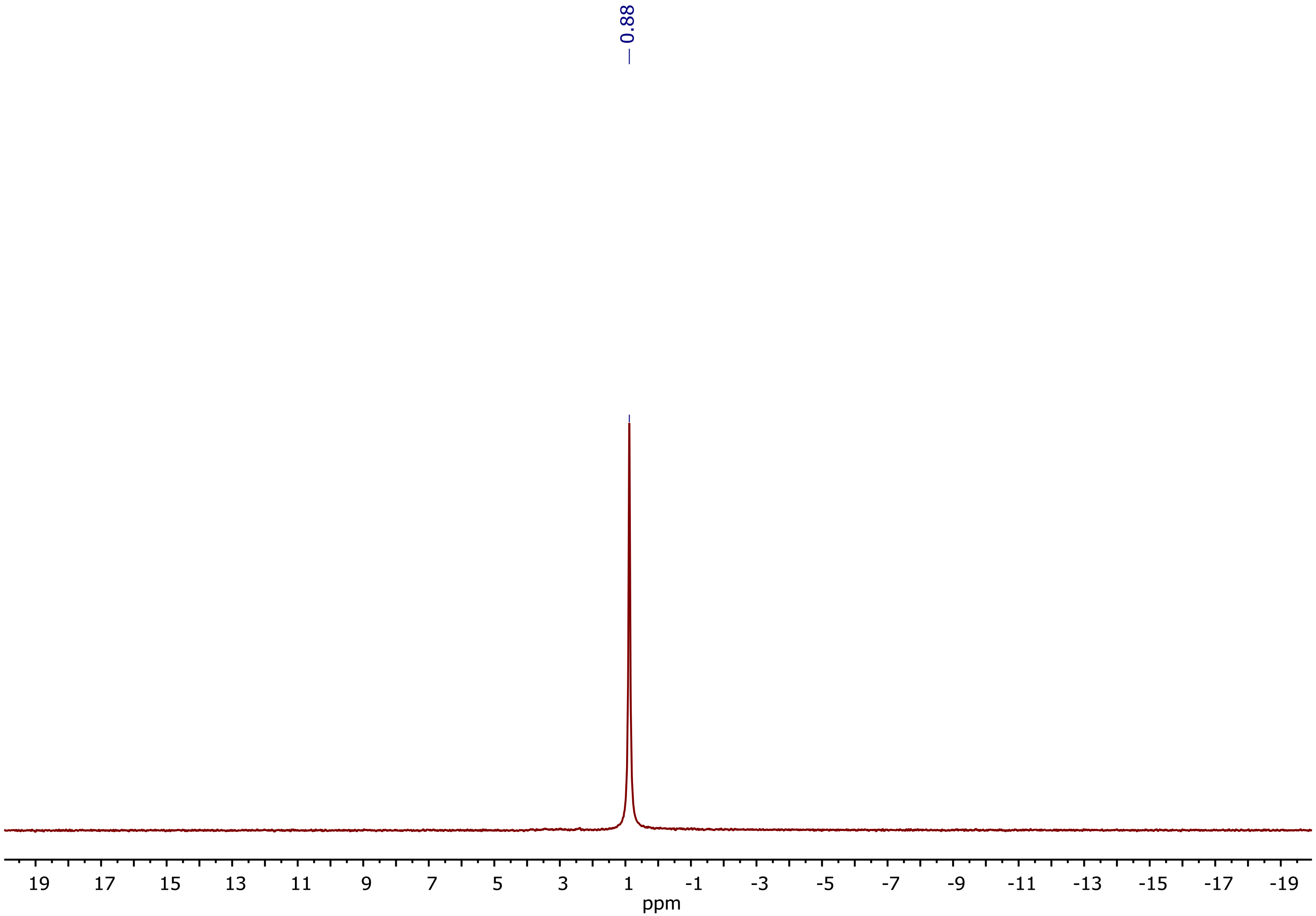

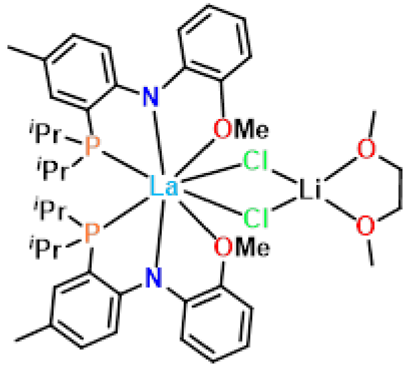

*Figure S 200: ^{7}Li{^{1}H} NMR spectrum of **4c** in C_6D_6 at 298 K.*

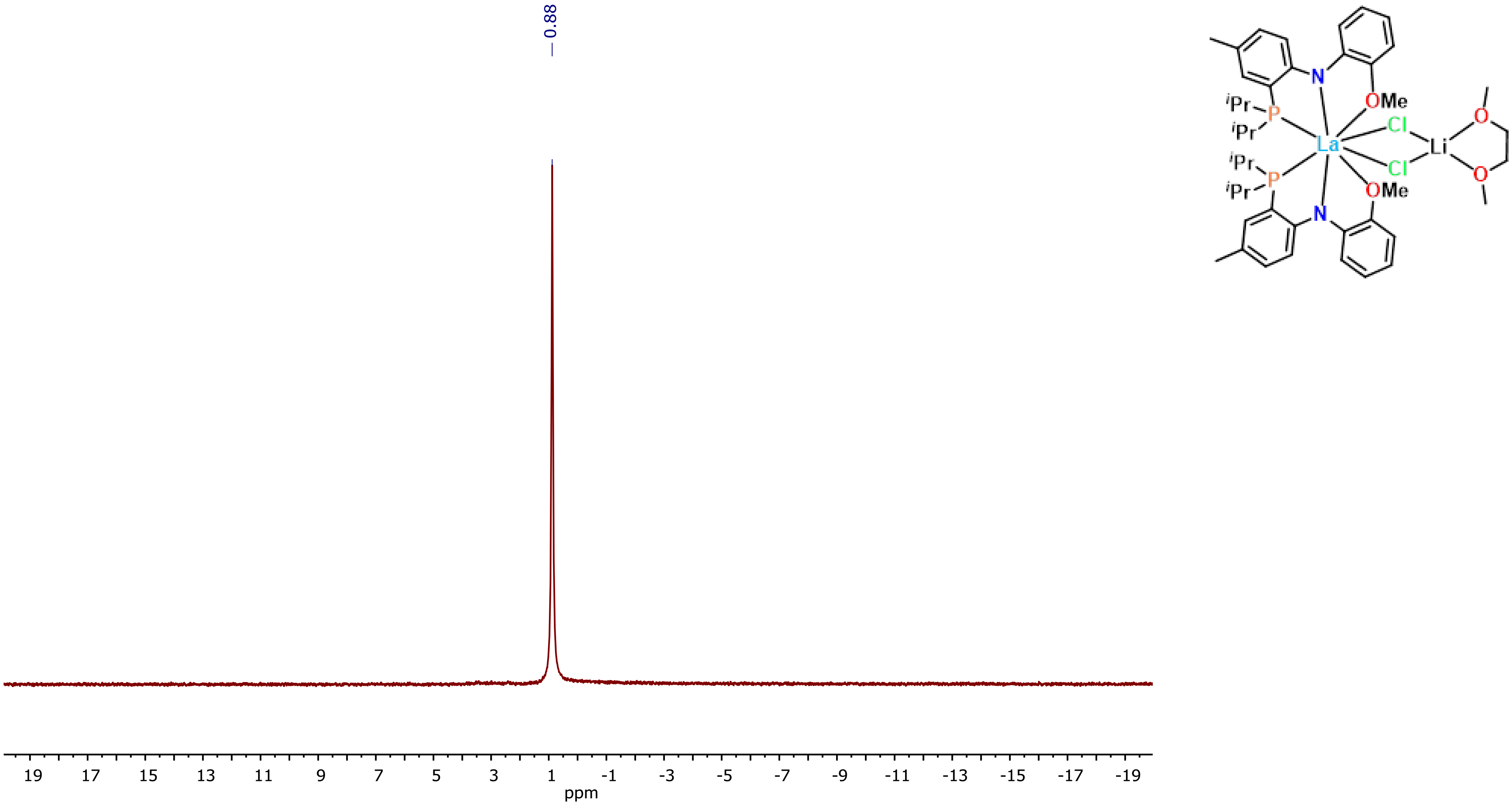

*Figure S 201: ⁷Li NMR spectrum of **4c** in C_6D_6 at 298 K.*

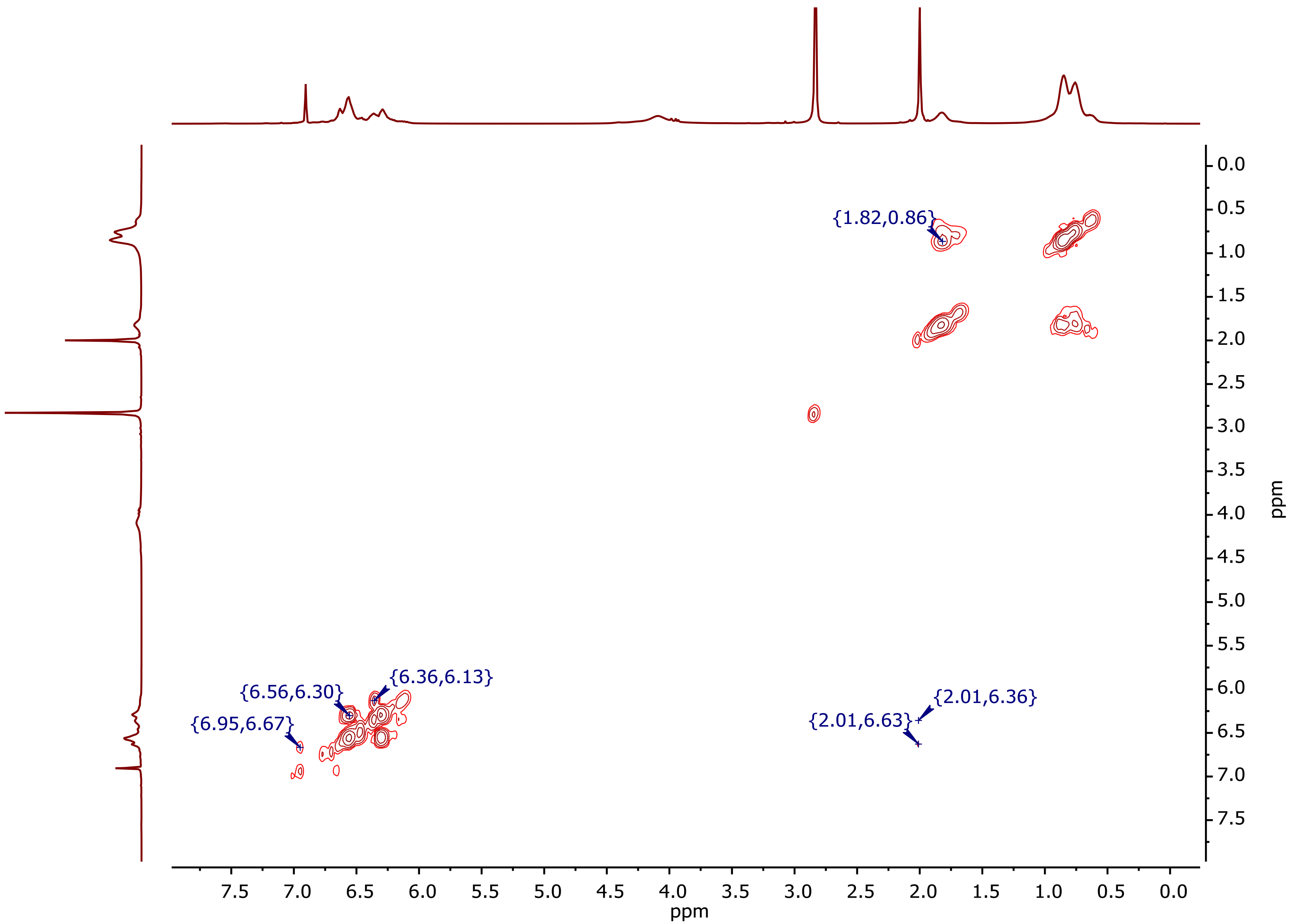

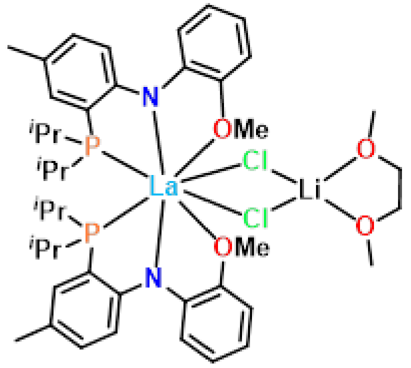

*Figure S 202: ^{1}H-^{1}H COSY NMR spectrum of **4c** in C_6D_6 at 298 K.*

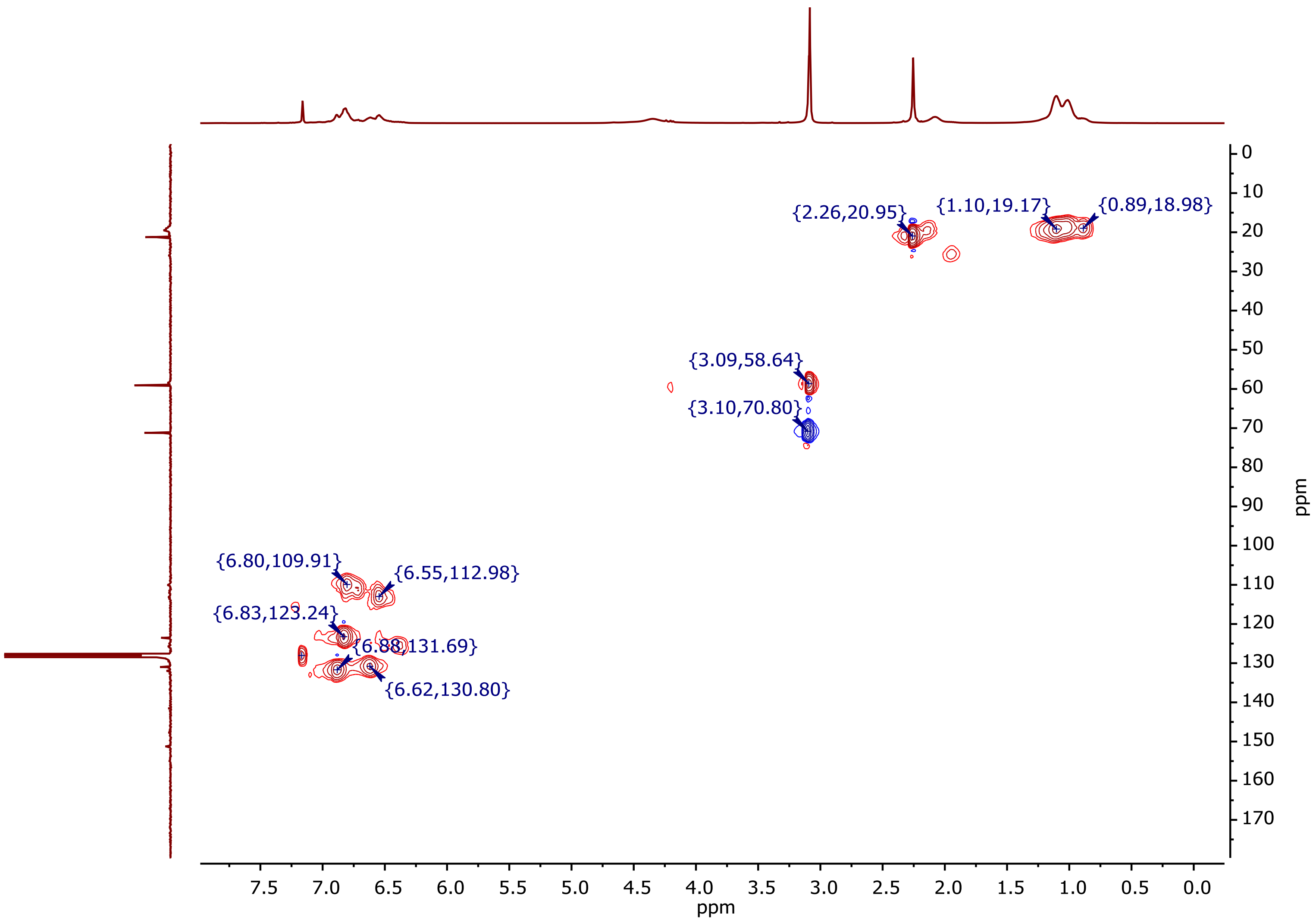

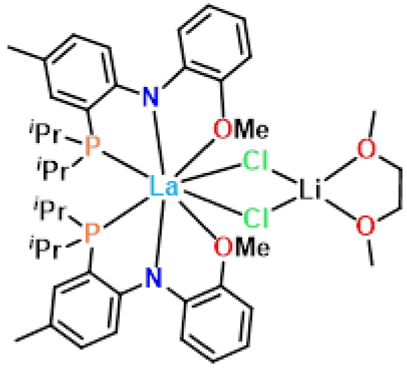

*Figure S 203: ^{1}H-^{13}C HSQC NMR spectrum of **4c** in C_6D_6 at 298 K.*

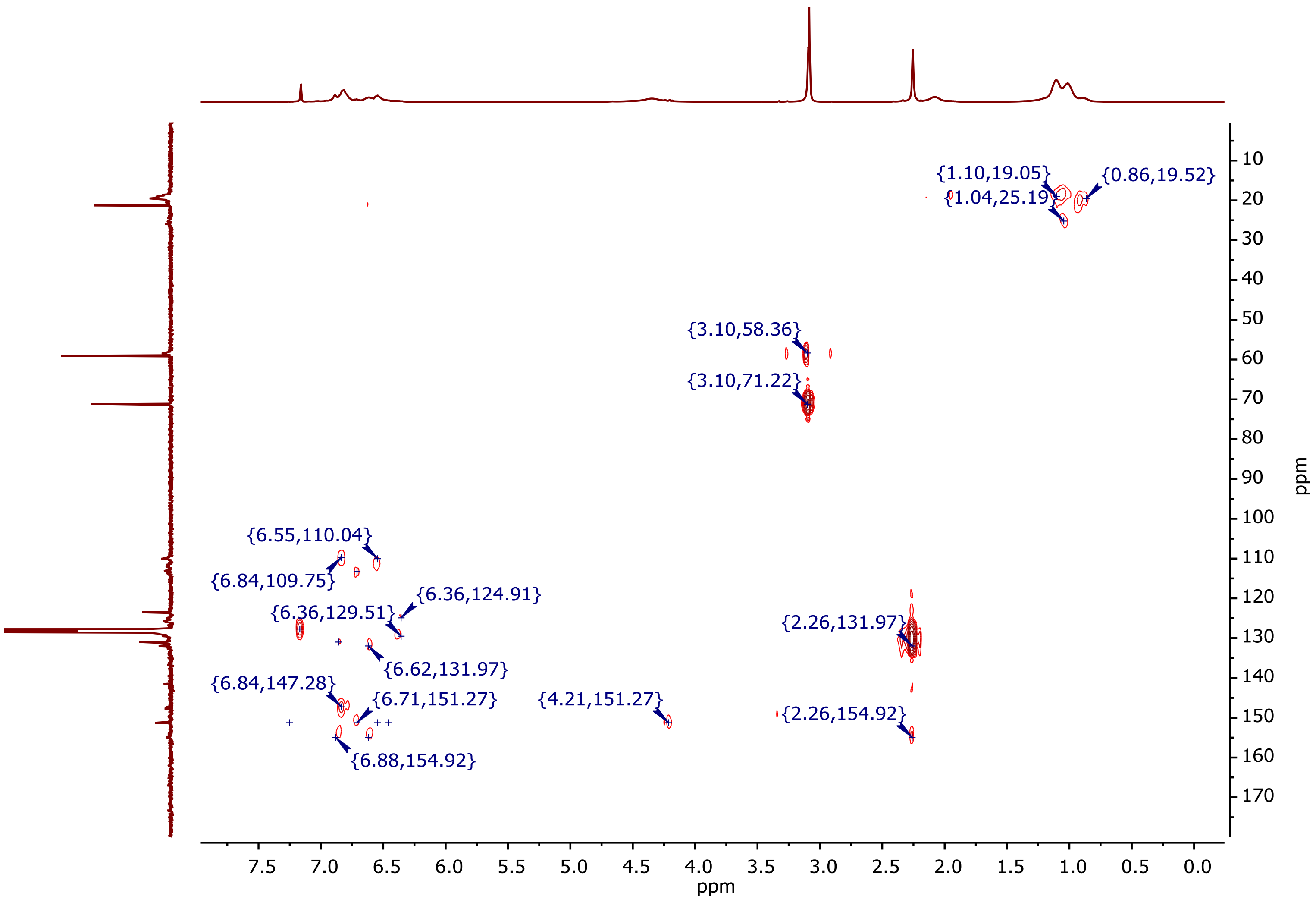

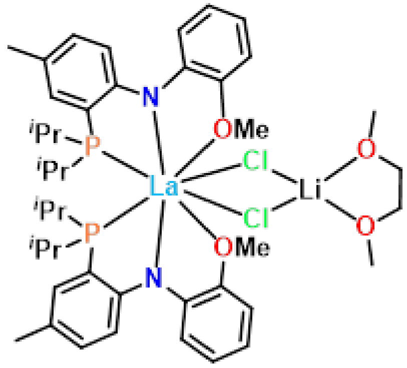

*Figure S 204: ^{1}H-^{13}C HMBC NMR spectrum of **4c** in C_6D_6 at 298 K.*

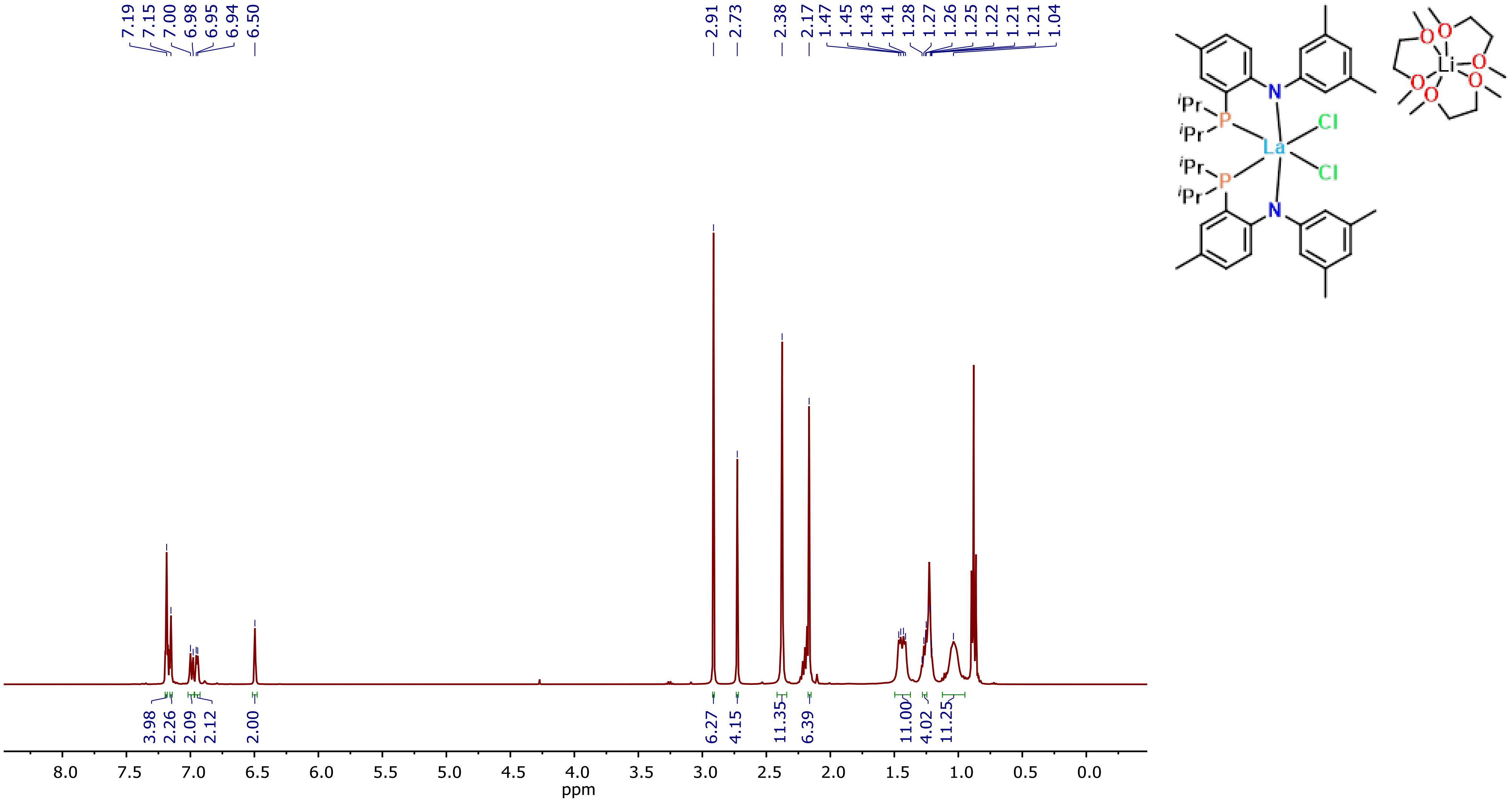

*Figure S 205: ^{1}H NMR spectrum of **4d** in C_6D_6 at 298 K.*

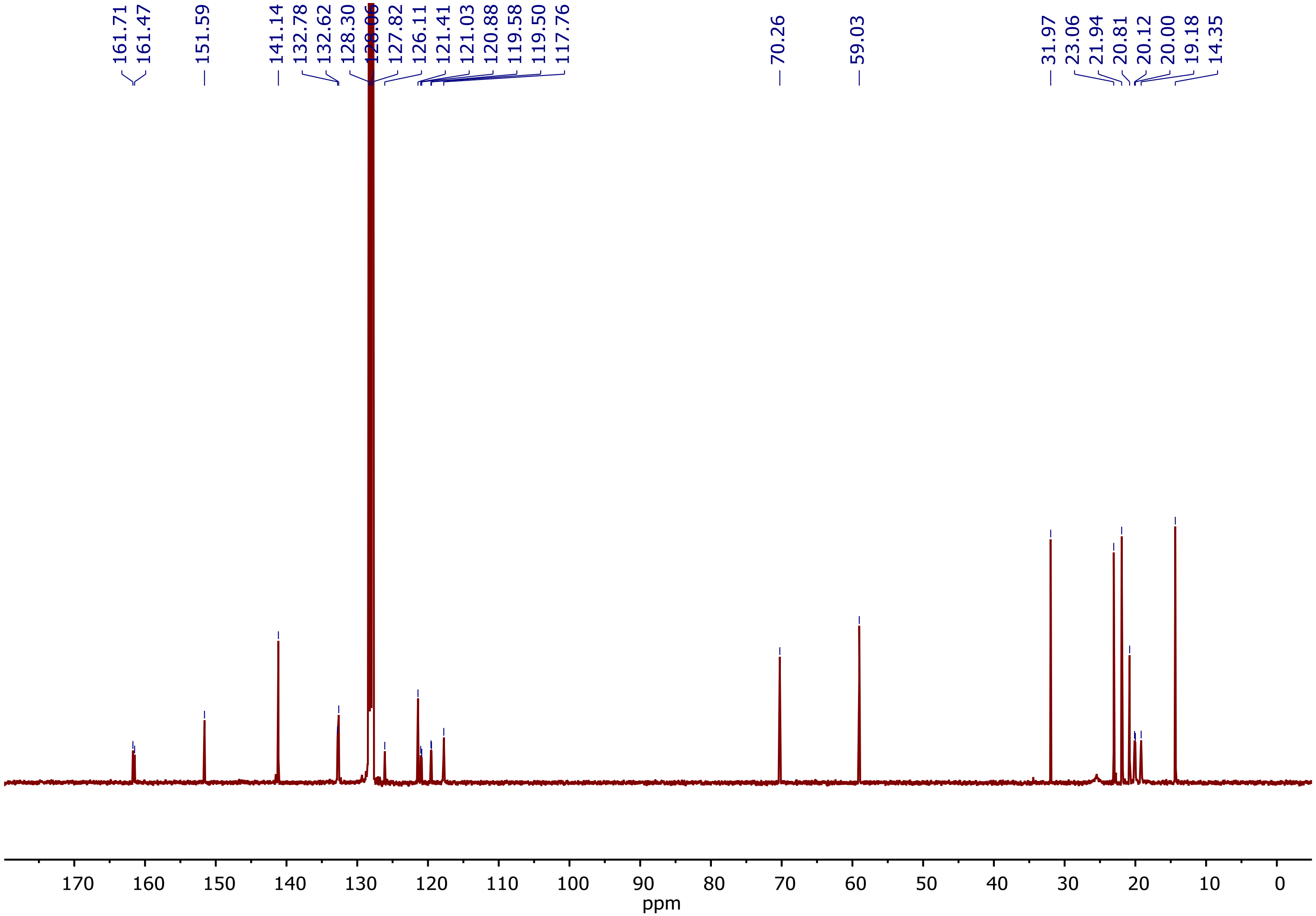

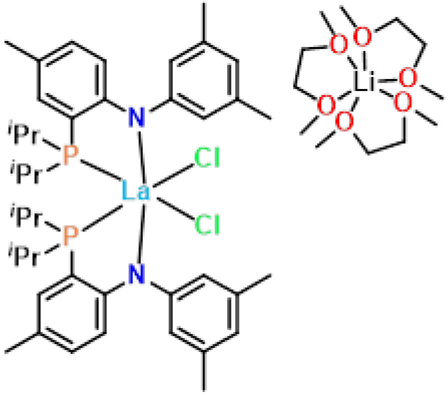

*Figure S 206: ^{13}C{^{1}H} NMR spectrum of **4d** in C_6D_6 at 298 K.*

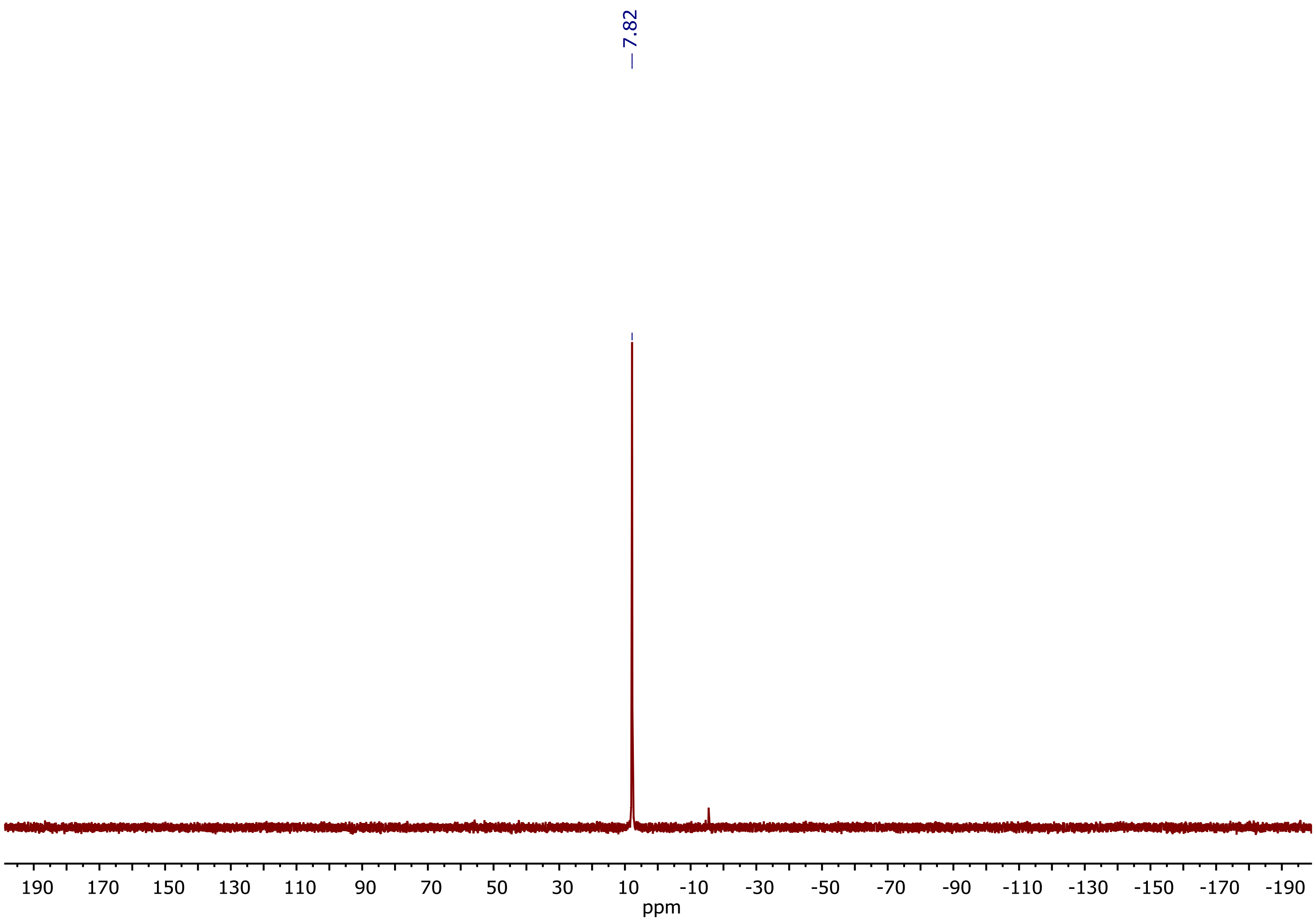

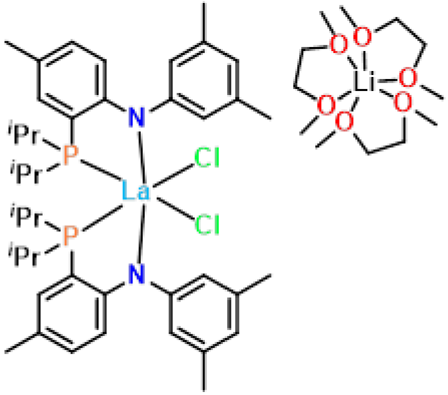

*Figure S 207: $^{31}P\{^{1}H\}$ NMR spectrum of **4d** in C_6D_6 at 298 K.*

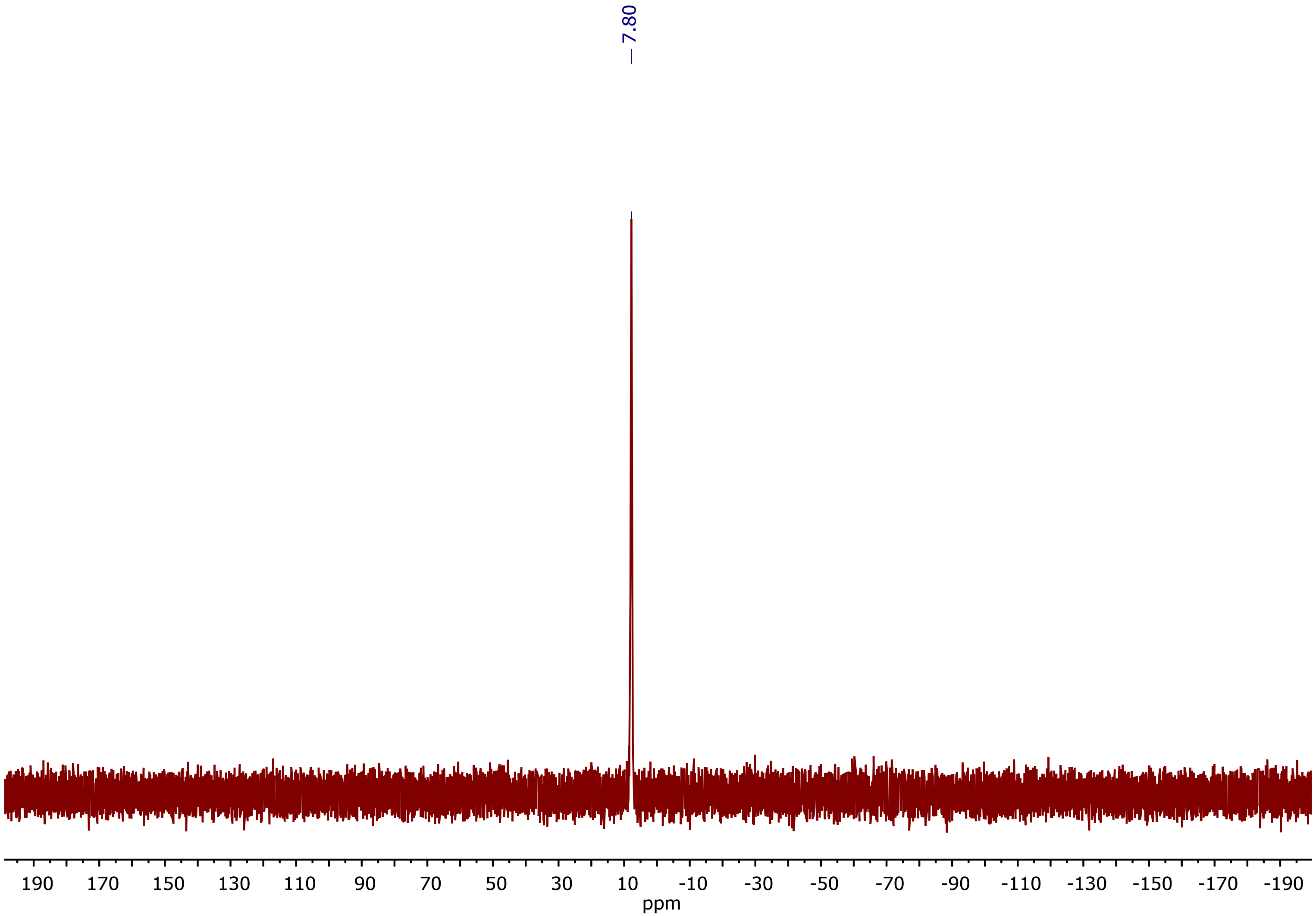

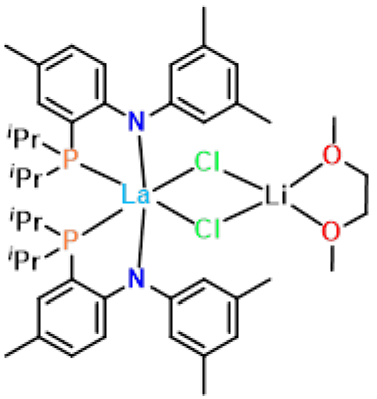

*Figure S 208: ^{31}P NMR spectrum of **4d** in C_6D_6 at 298 K.*

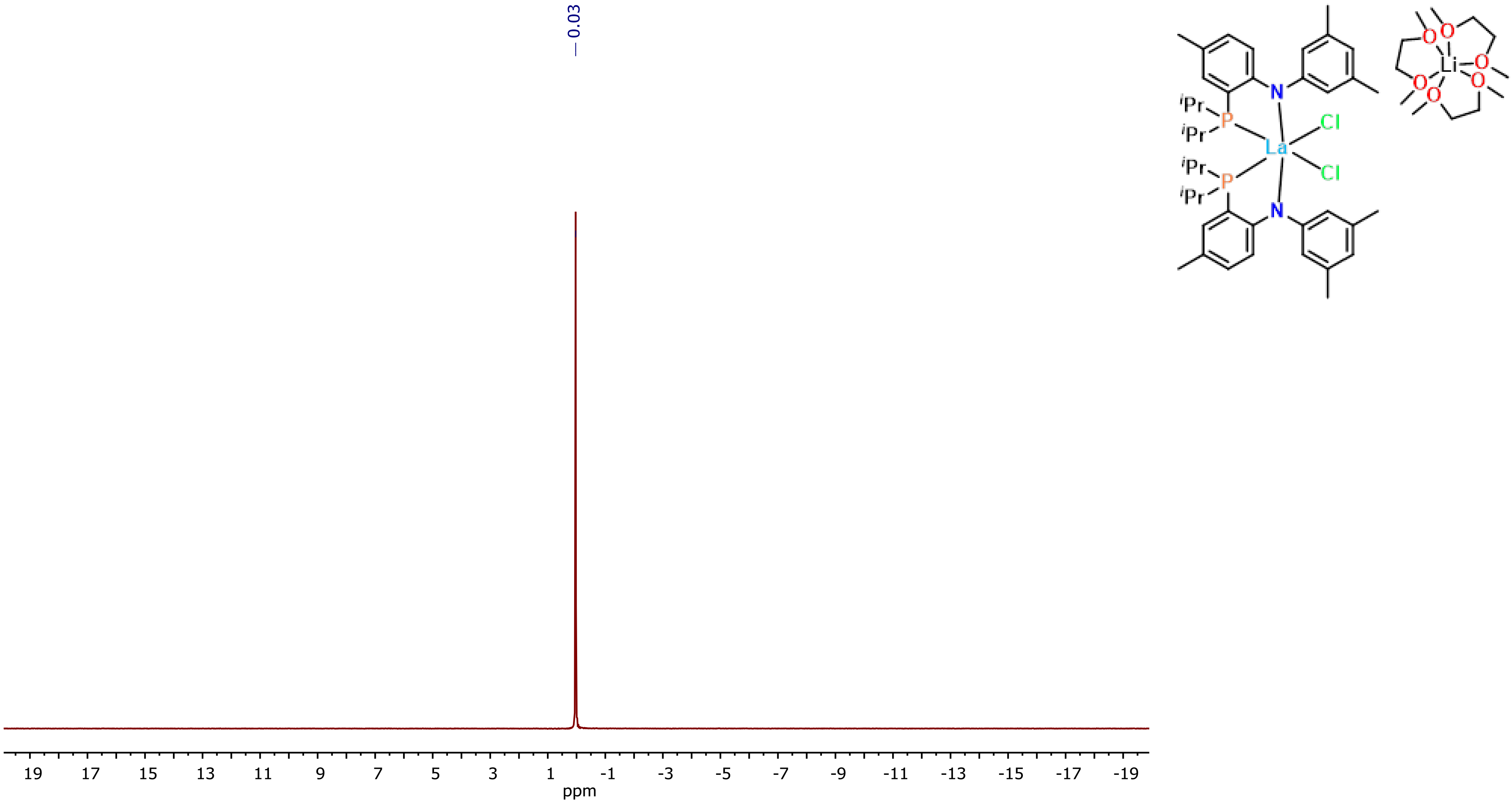

*Figure S 209: $^{7}Li\{^{1}H\}$ NMR spectrum of **4d** in C_6D_6 at 298 K.*

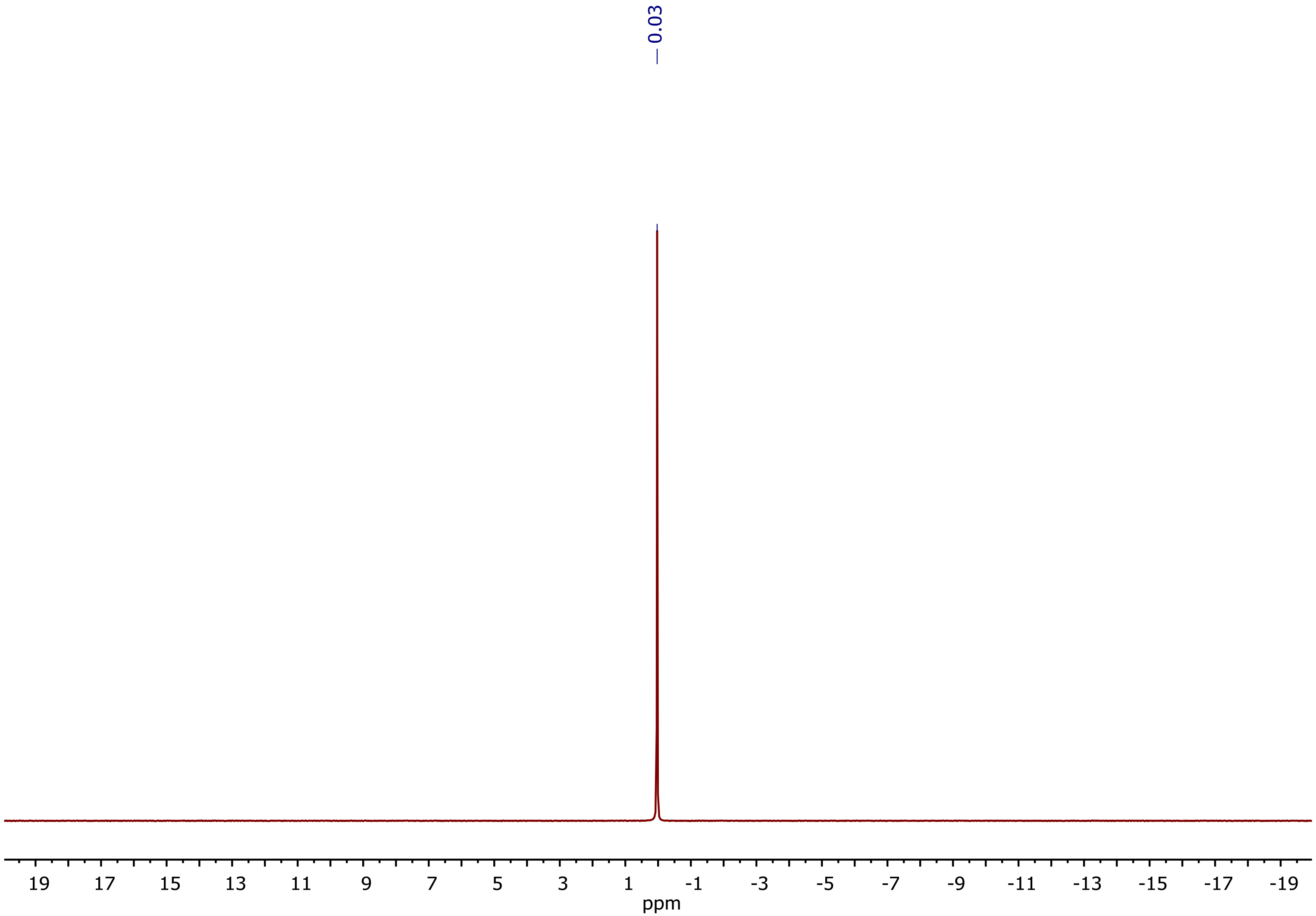

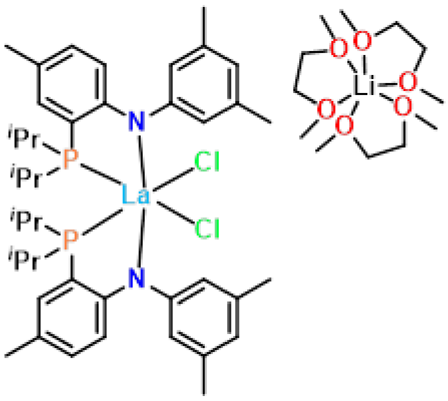

*Figure S 210: ^{7}Li NMR spectrum of **4d** in C_6D_6 at 298 K.*

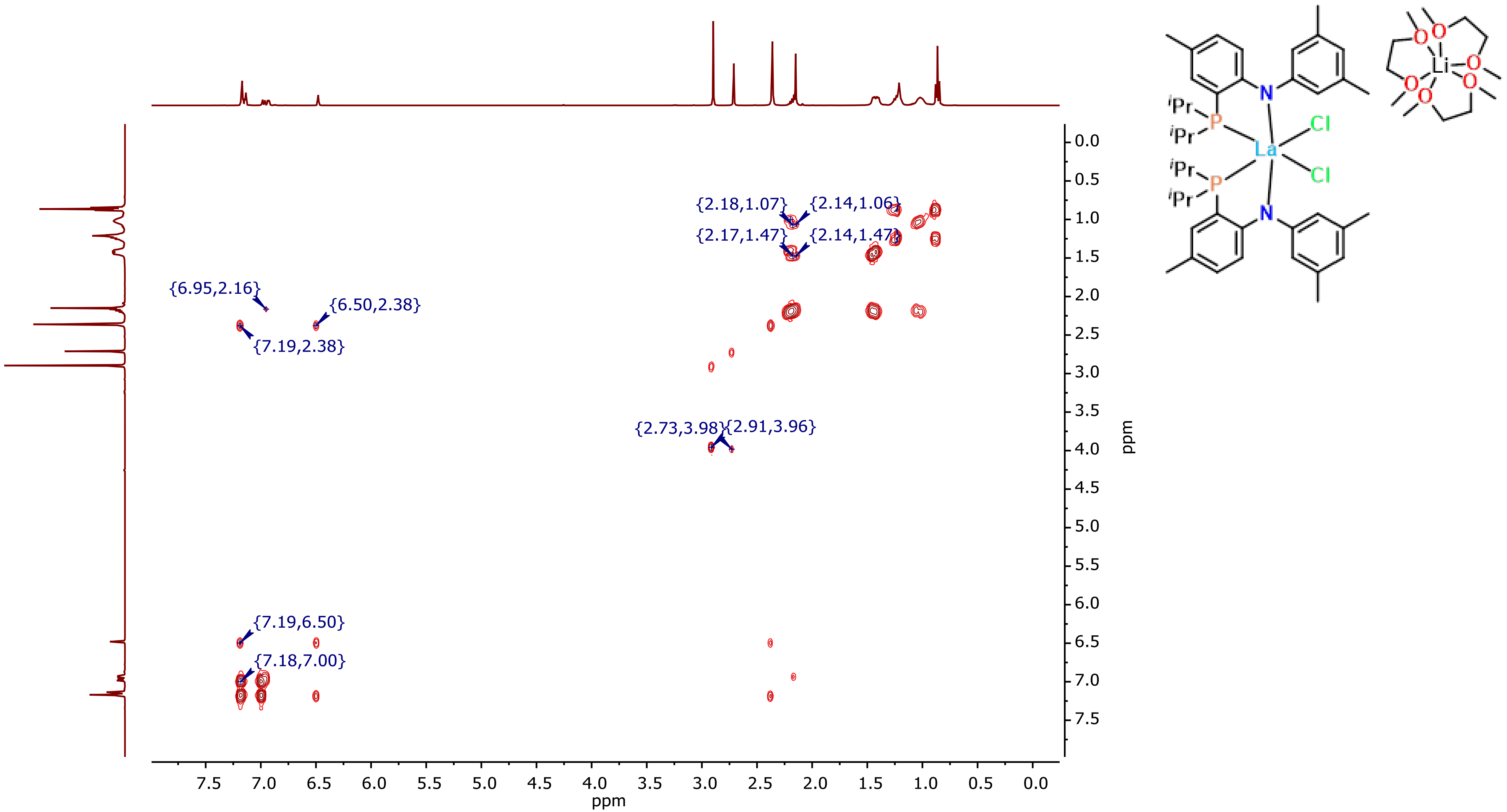

*Figure S 211: ^{1}H-^{1}H COSY NMR spectrum of **4d** in C_6D_6 at 298 K.*

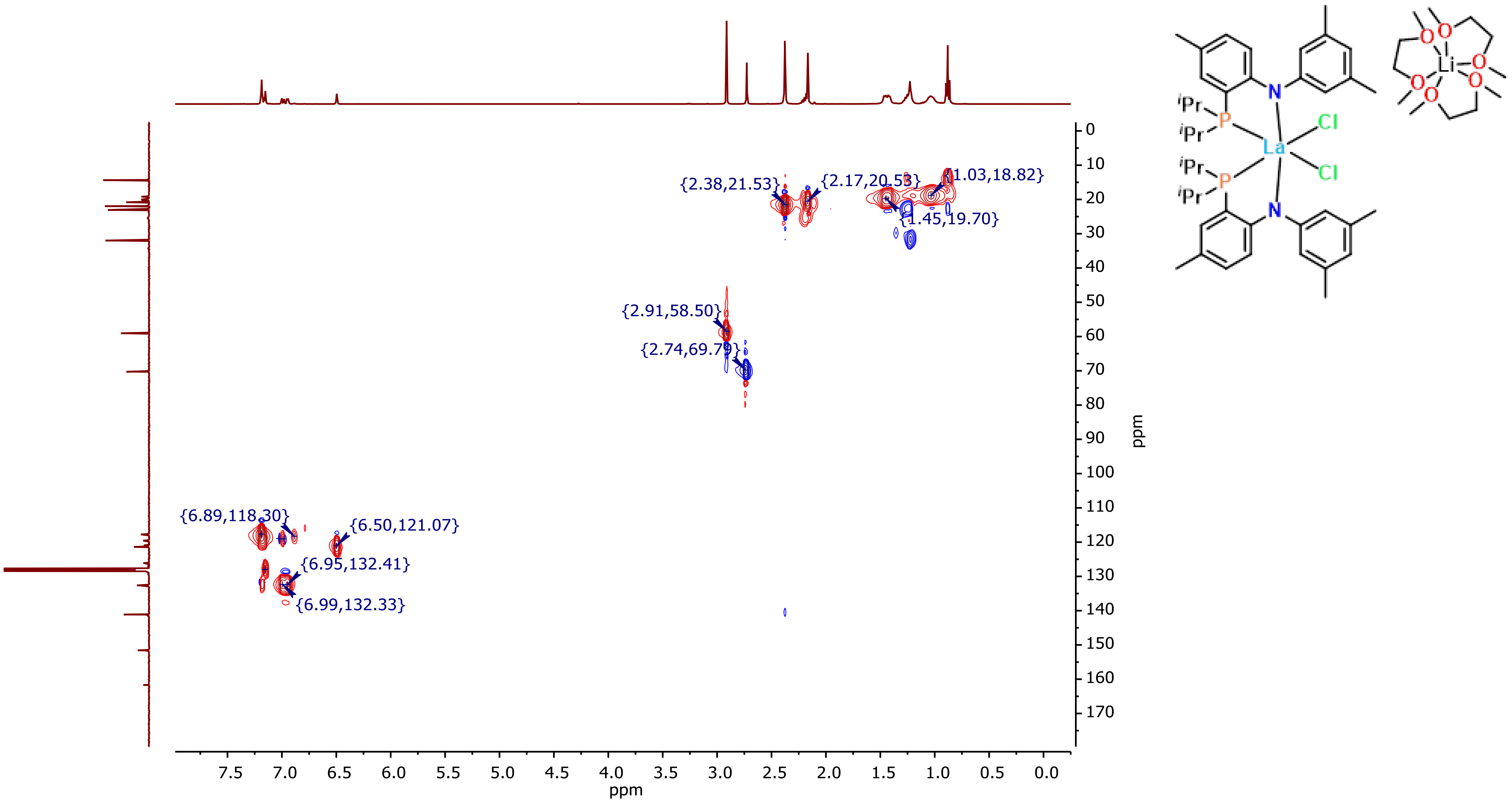

Figure S 212: ^{1}H-^{13}C HSQC NMR spectrum of **4d** in C_6D_6 at 298 K.

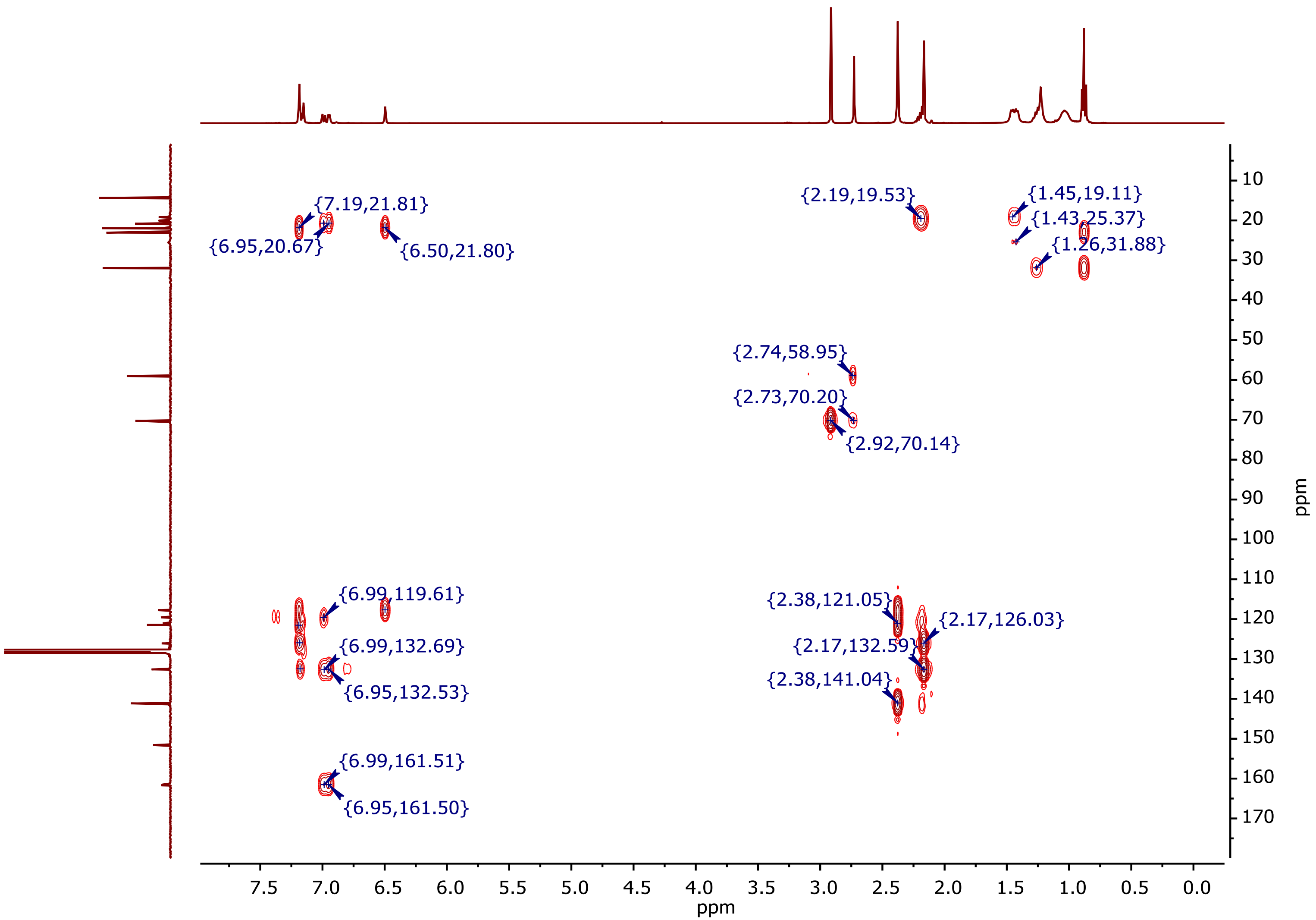

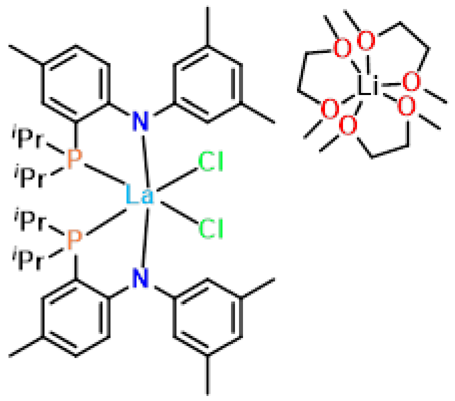

Figure S 213: ^{1}H-^{13}C HMBC NMR spectrum of **4d** in C_6D_6 at 298 K.

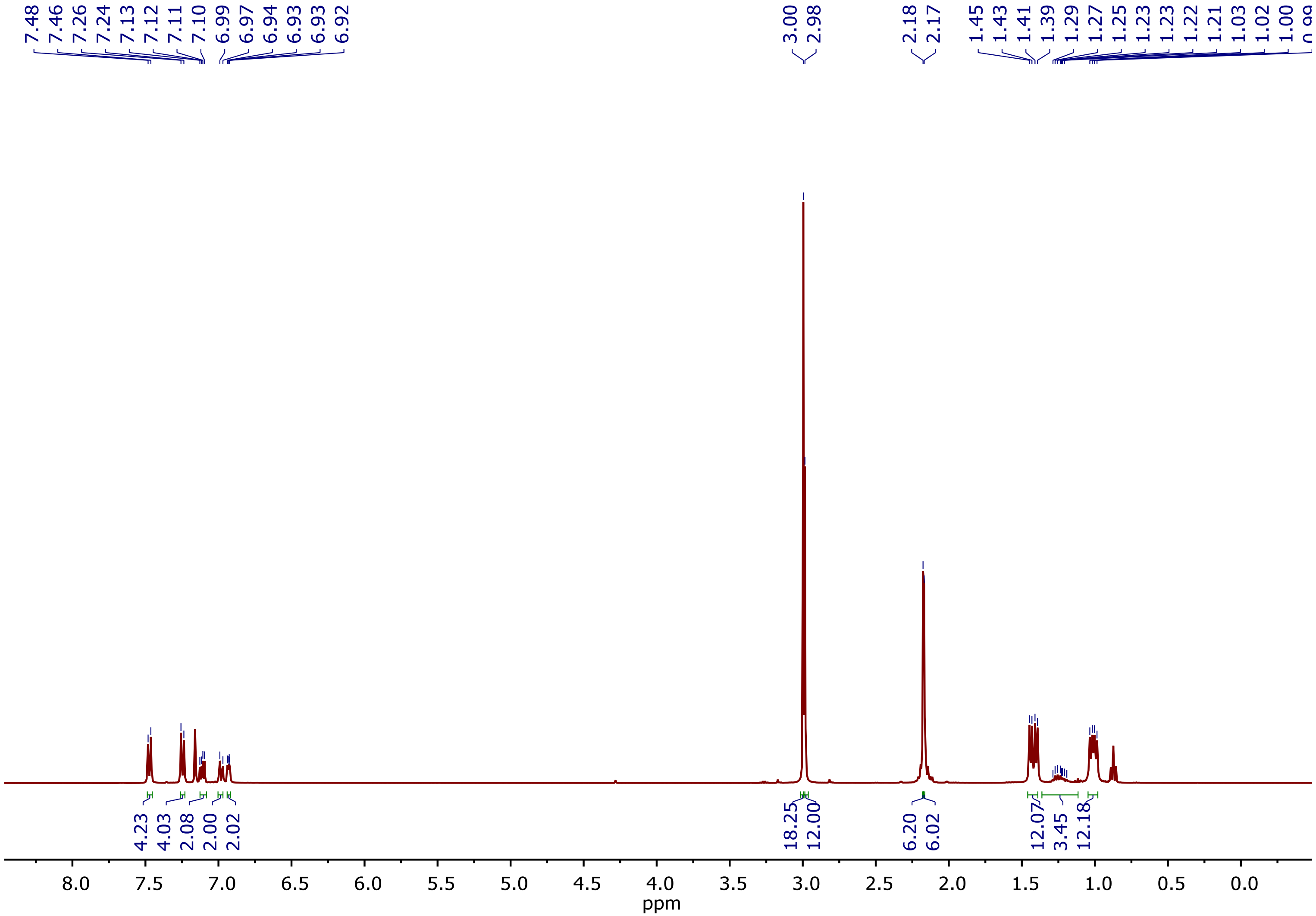

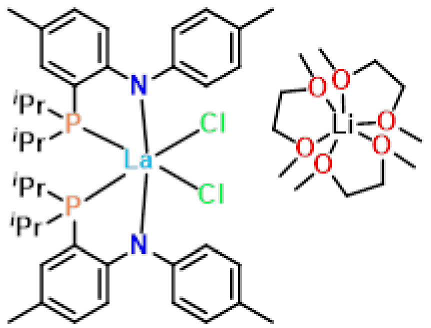

*Figure S 214: ^{1}H NMR spectrum of **4f** in C_6D_6 at 298 K.*

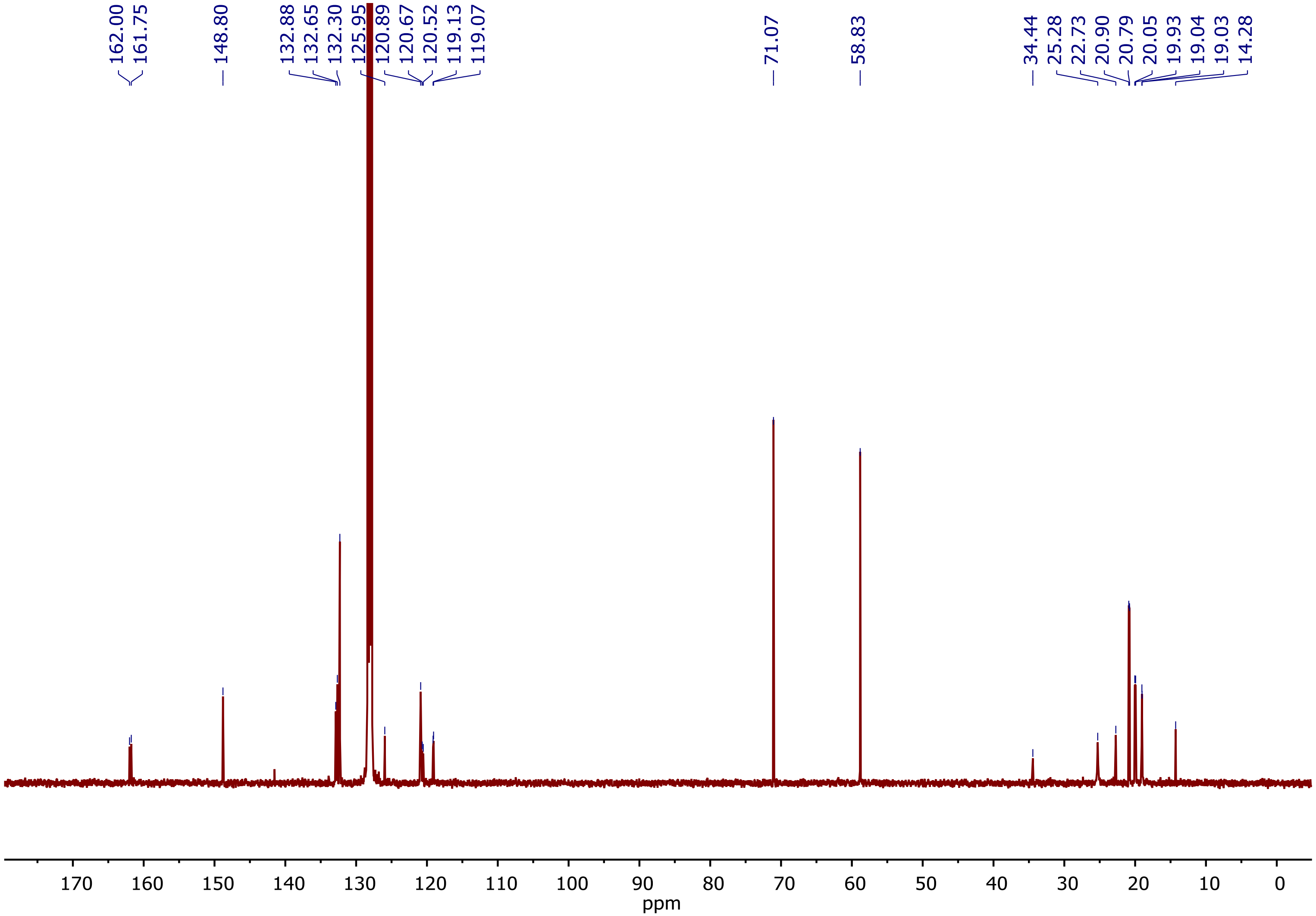

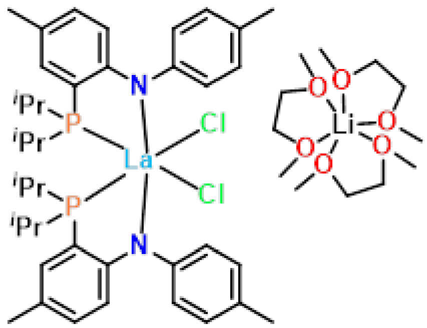

*Figure S 215: $^{13}C\{^1H\}$ NMR spectrum of **4f** in C_6D_6 at 298 K.*

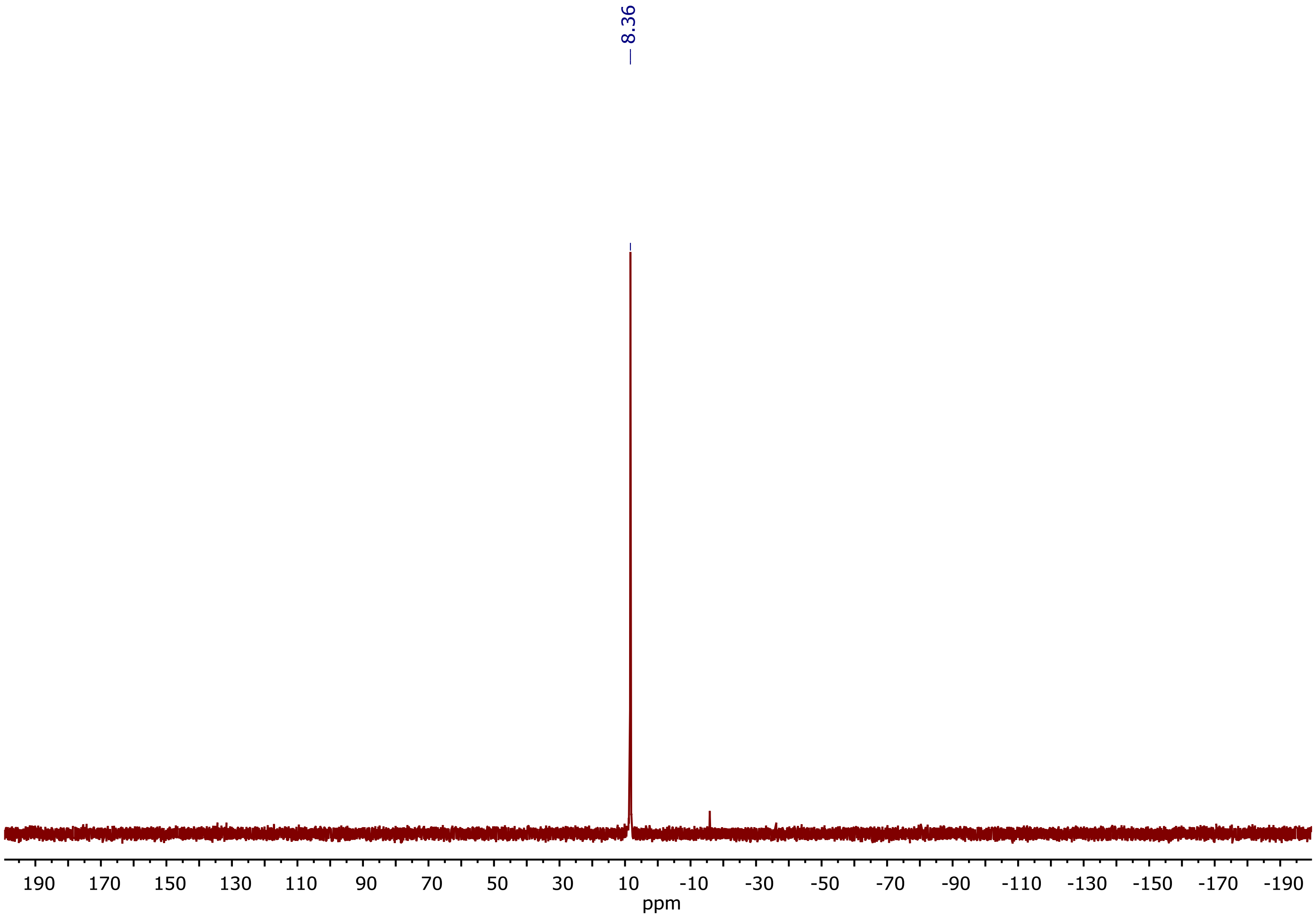

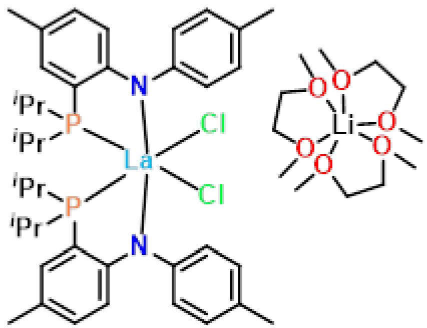

*Figure S 216: $^{31}P\{^1H\}$ NMR spectrum of **4f** in C_6D_6 at 298 K.*

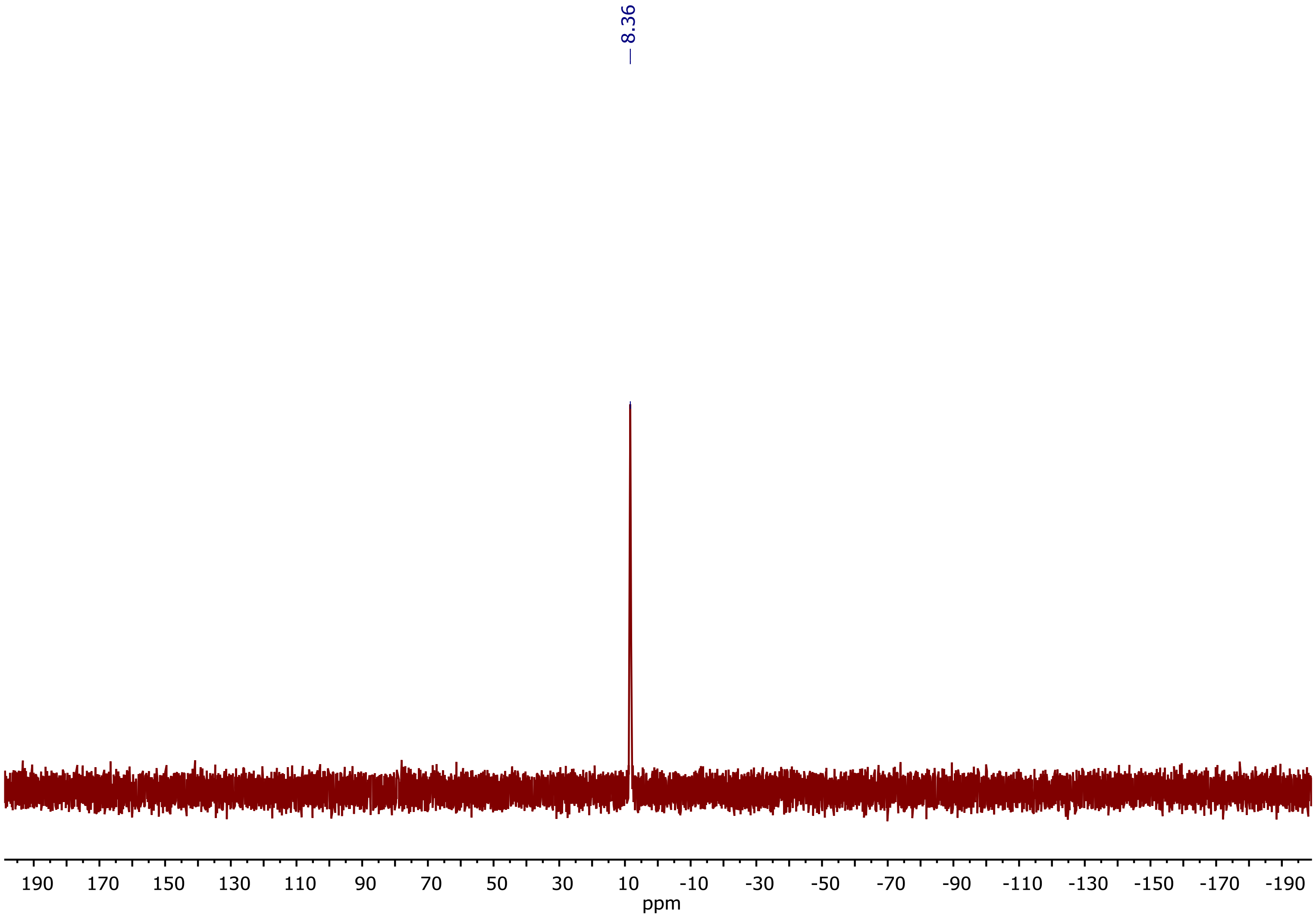

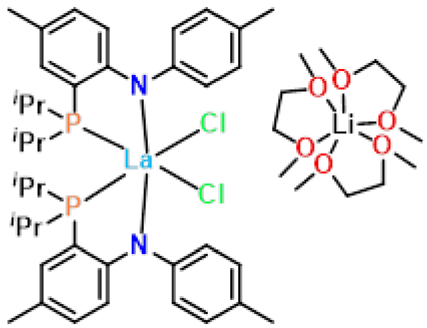

*Figure S 217: ^{31}P NMR spectrum of **4f** in C_6D_6 at 298 K.*

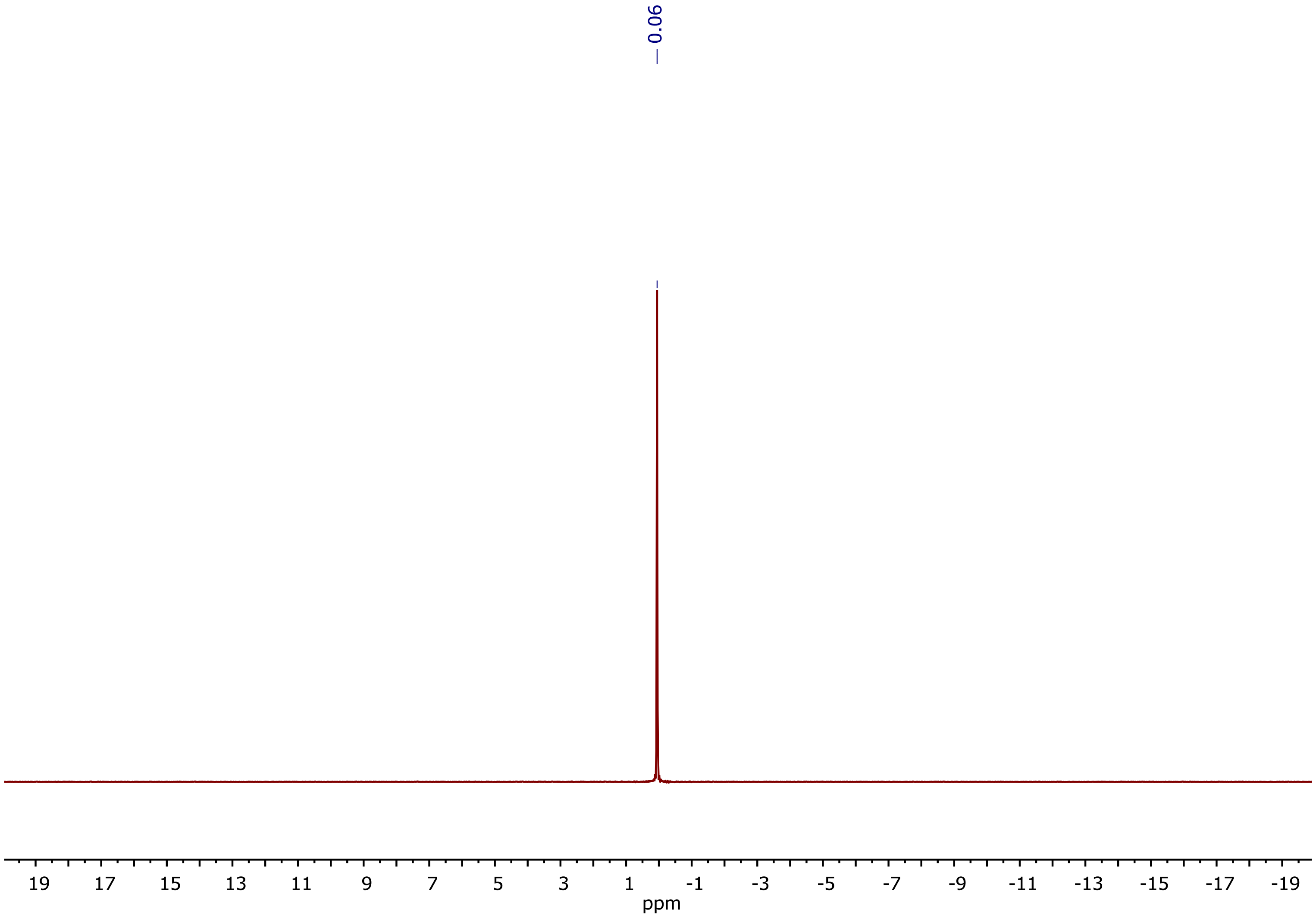

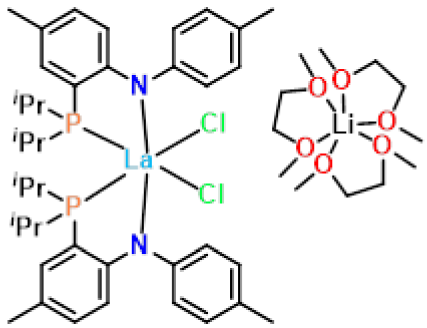

*Figure S 218: ^{7}Li{^{1}H} NMR spectrum of **4f** in C_6D_6 at 298 K.*

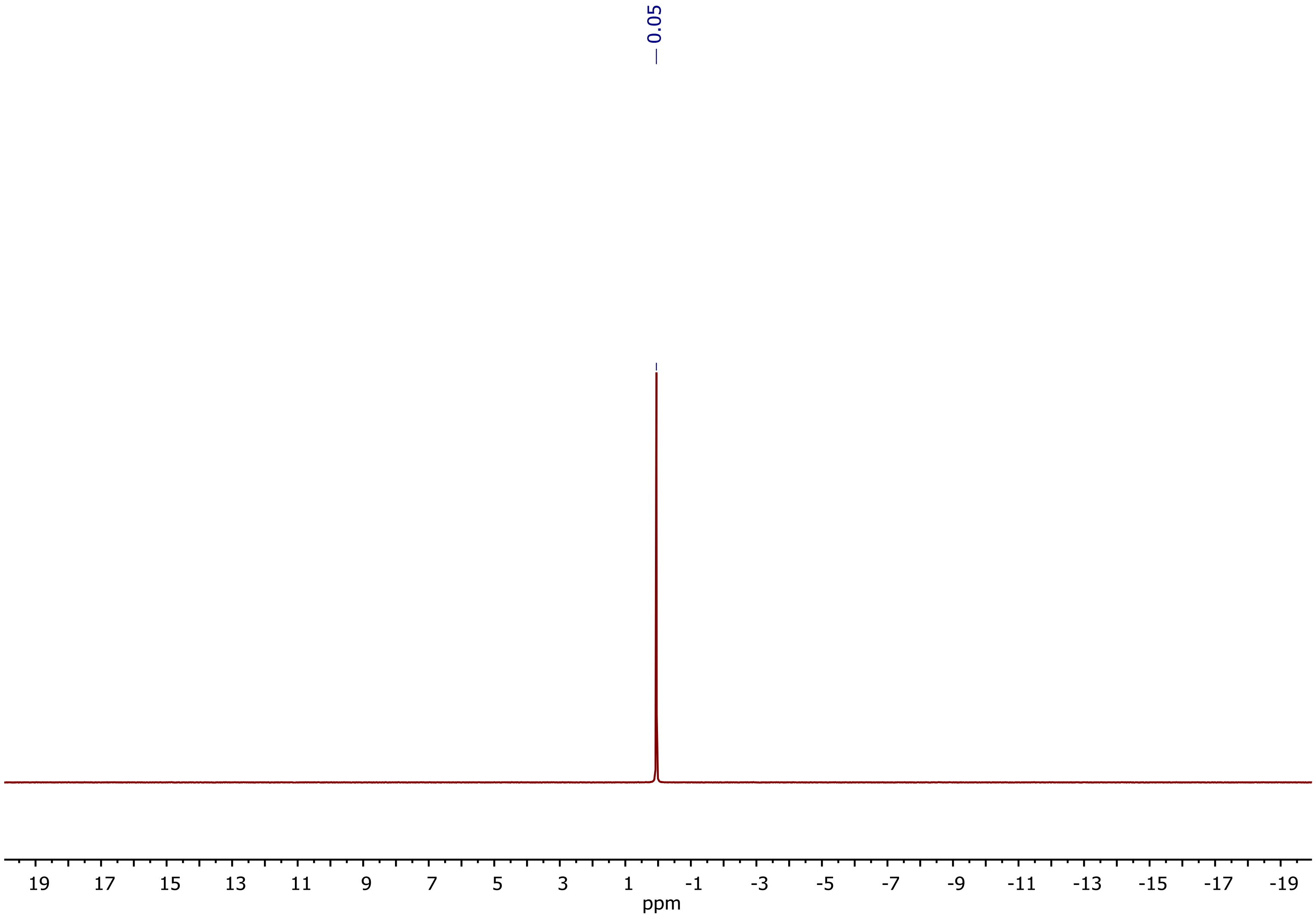

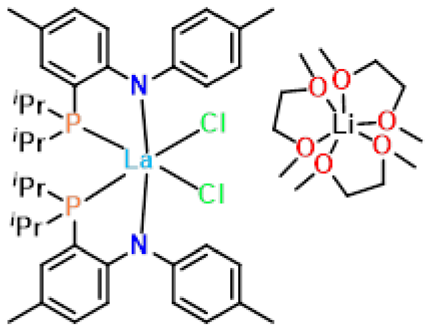

*Figure S 219: ^{7}Li NMR spectrum of **4f** in C_6D_6 at 298 K.*

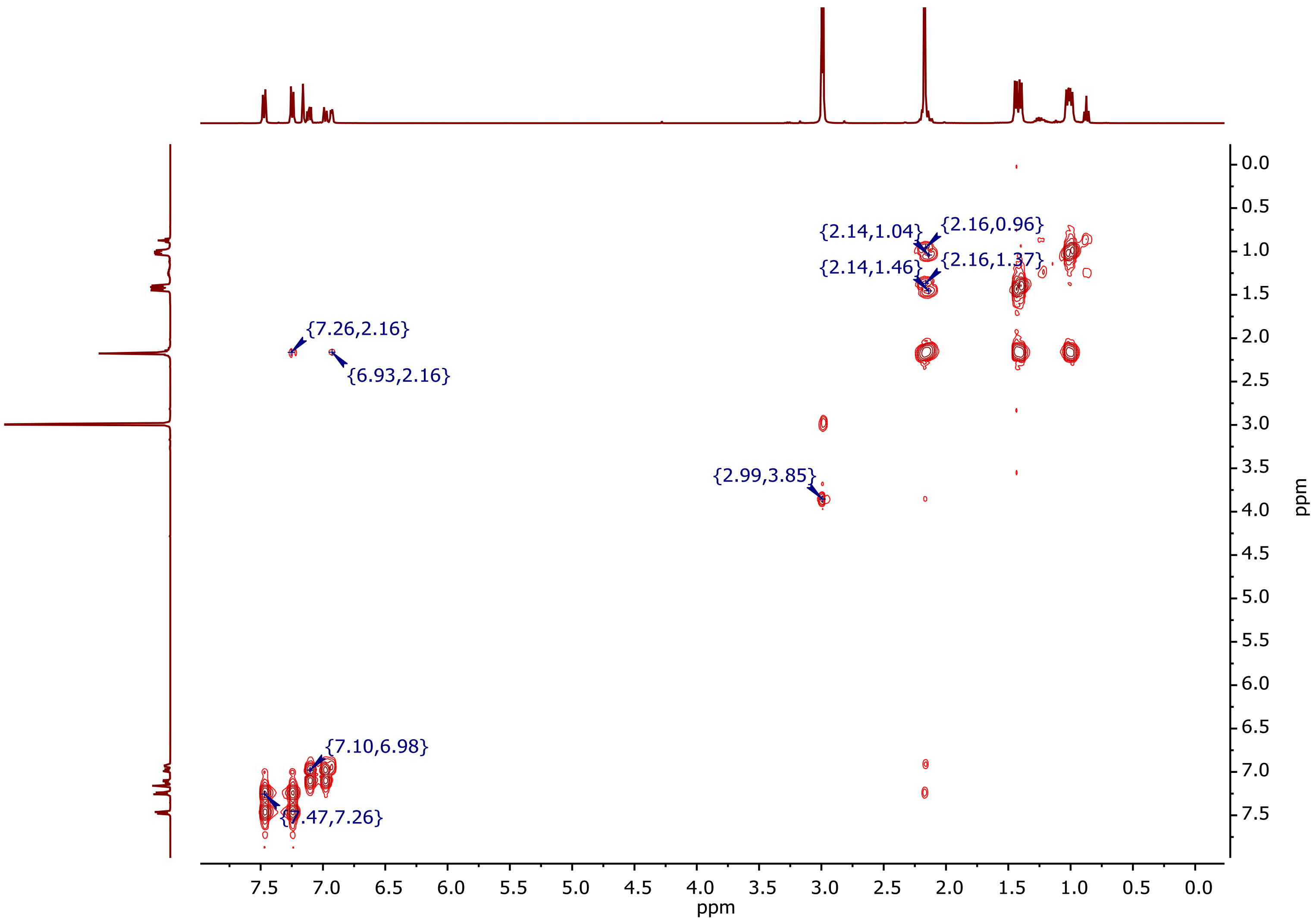

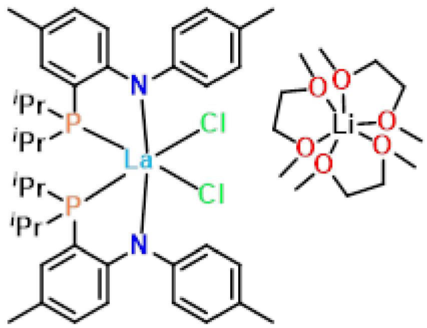

Figure S 220: ¹H-¹H COSY NMR spectrum of **4f** in C_6D_6 at 298 K.

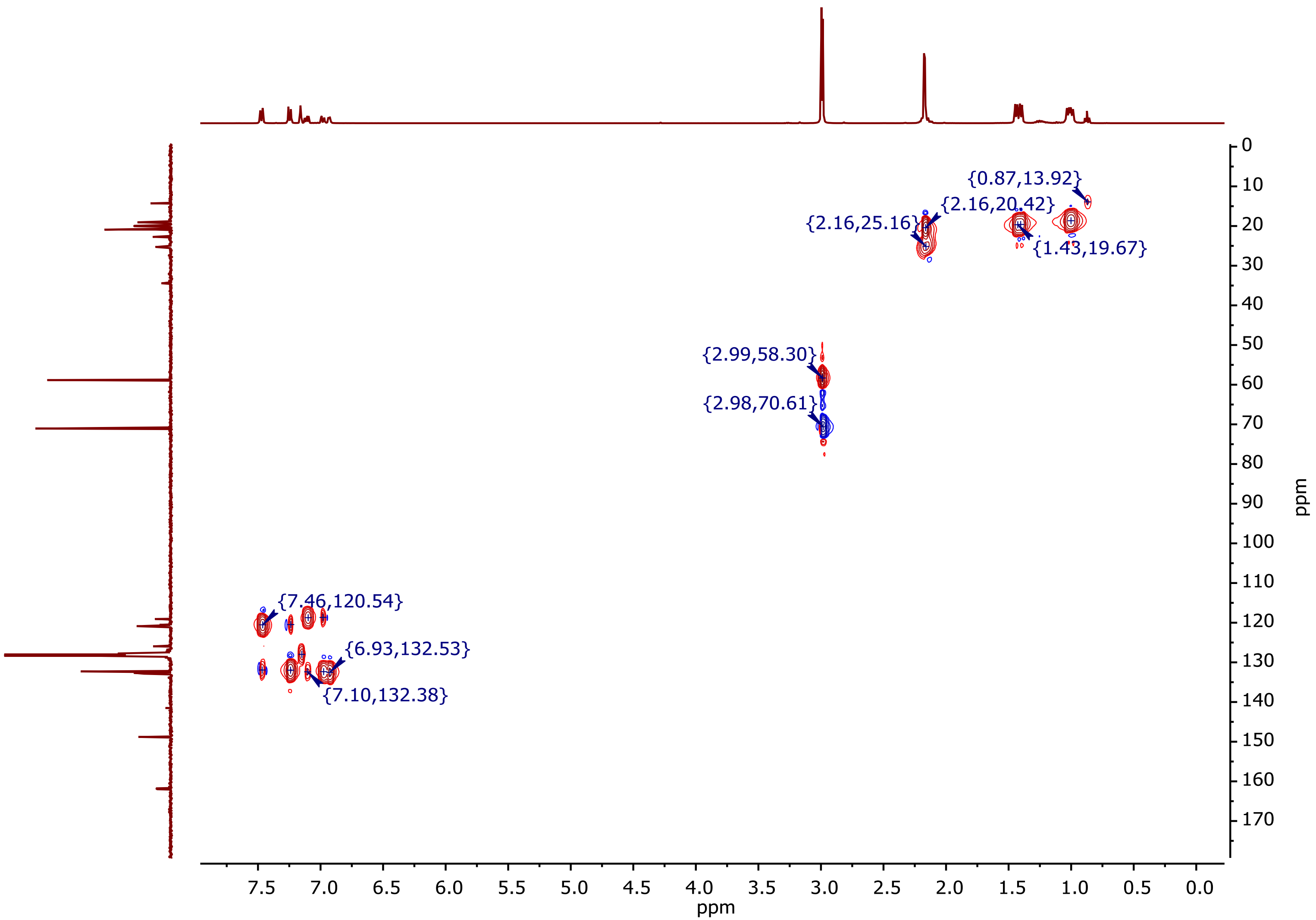

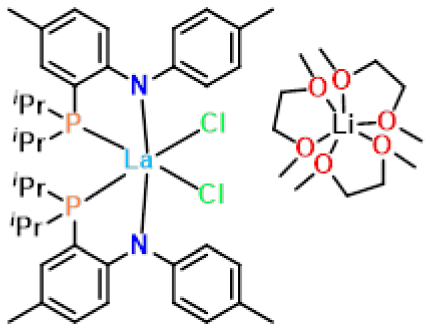

Figure S 221: ^{1}H-^{13}C HSQC NMR spectrum of ***4f*** in C_6D_6 at 298 K.

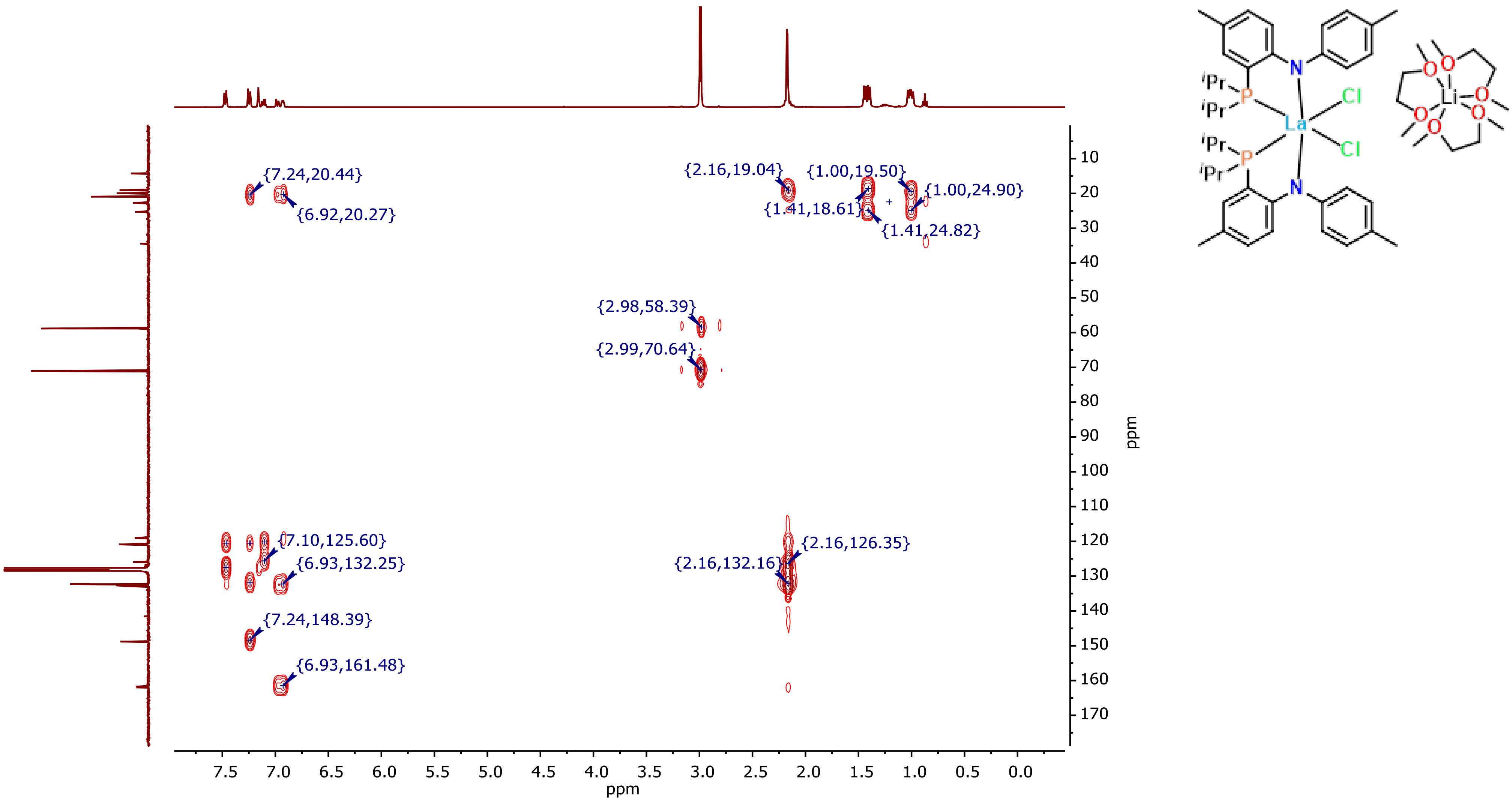

*Figure S 222: ^{1}H-^{13}C HMBC NMR spectrum of **4f** in C_6D_6 at 298 K.*

Mass spectra

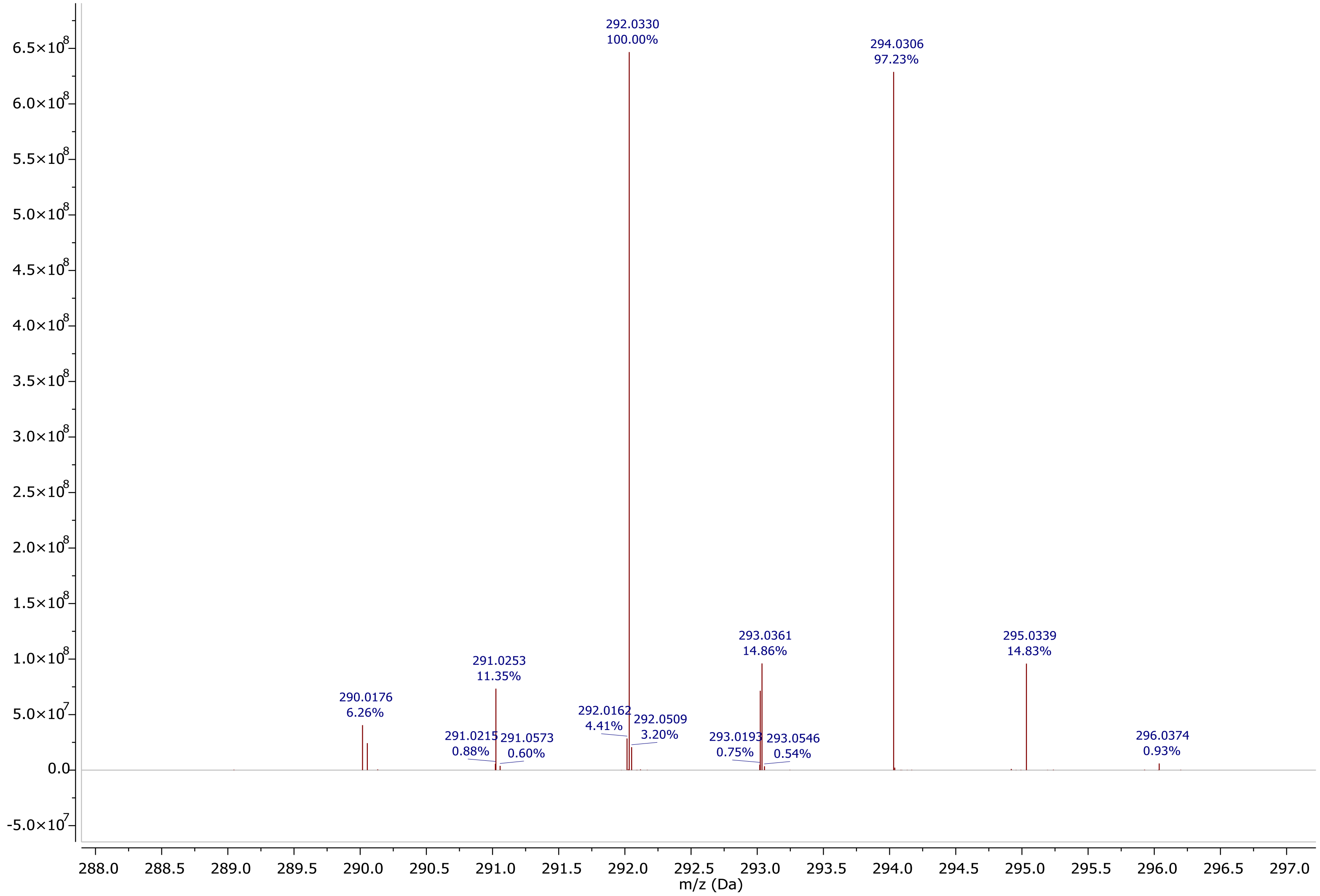

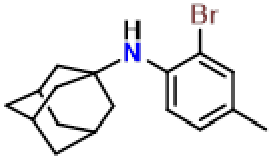

*Figure S 223: Hi – Res Mass (ESI$^+$) of **2a**.*

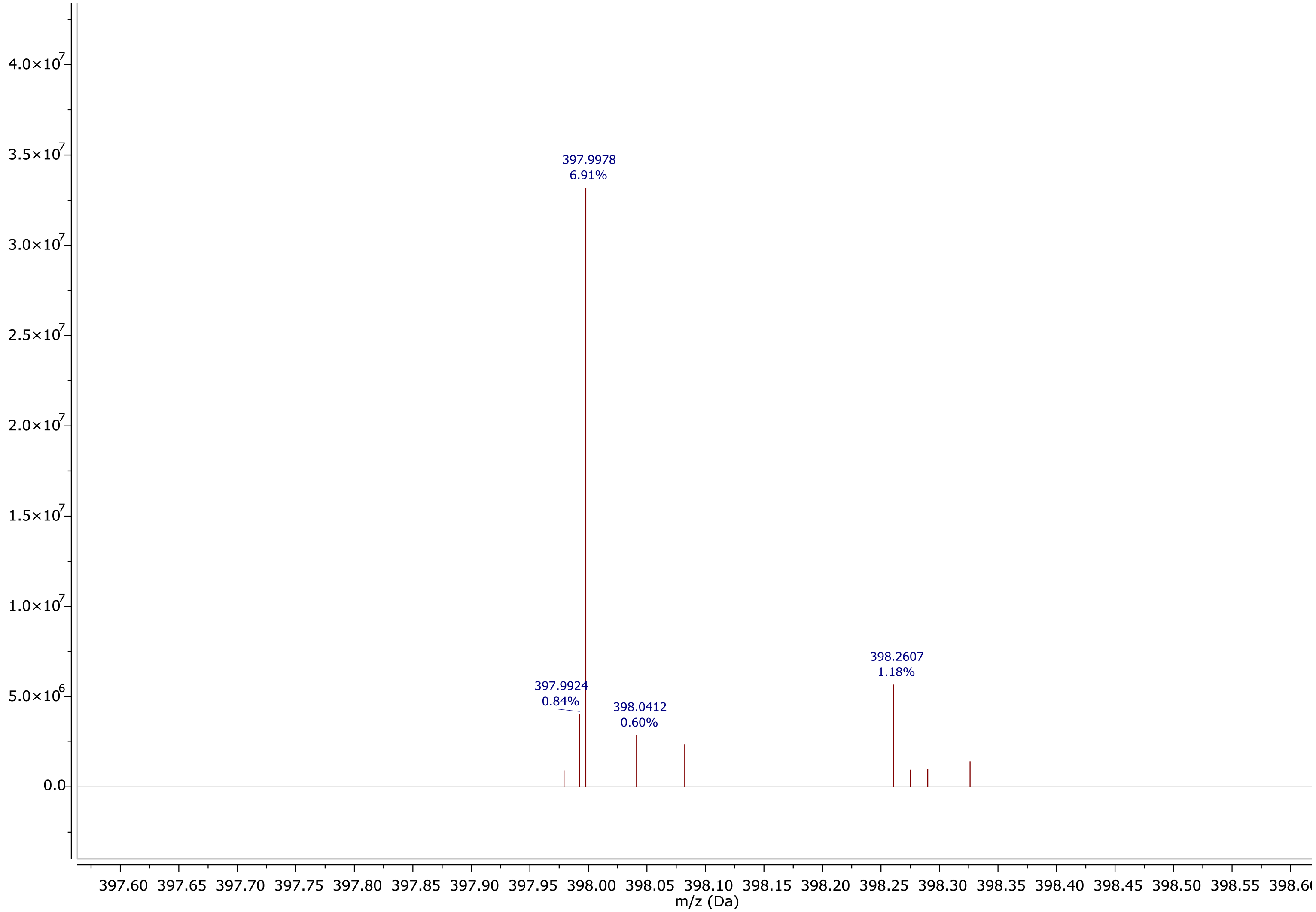

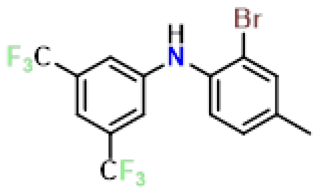

*Figure S 224: Hi – Res Mass (ESI$^+$) of **2b**.*

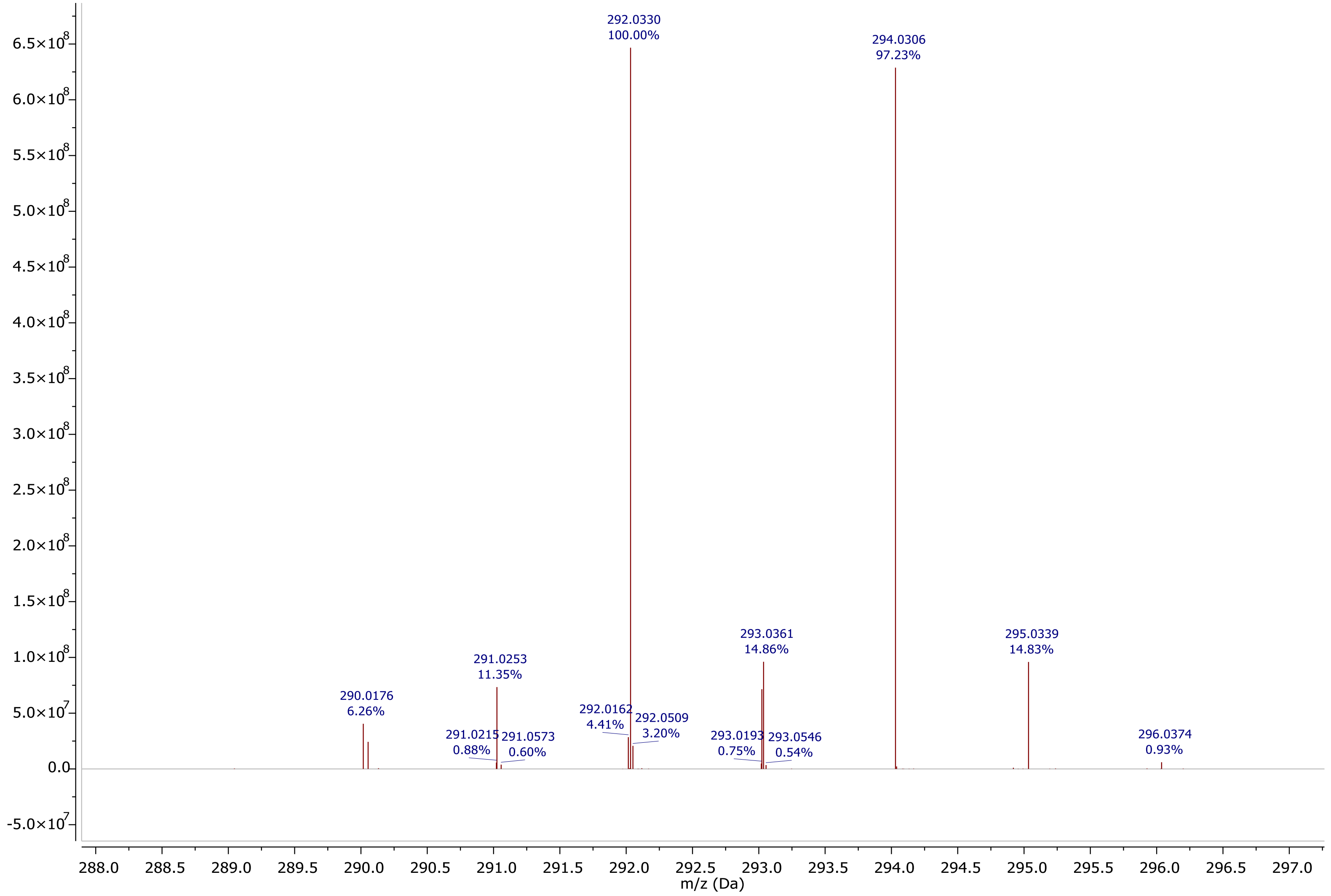

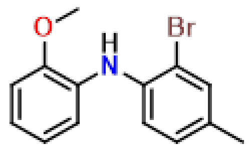

Figure S 225: Hi – Res Mass (ESI$^+$) of ***2c.***

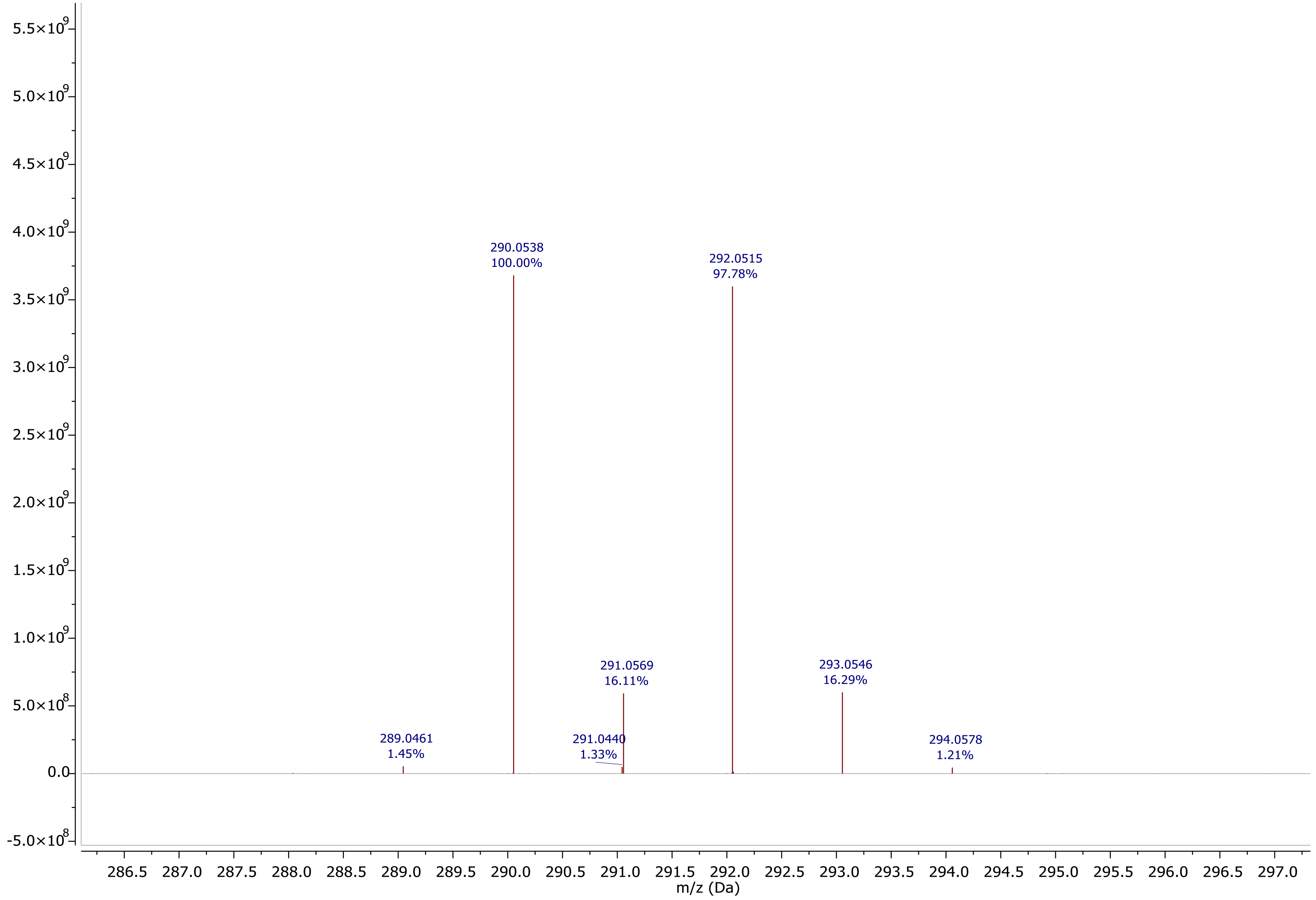

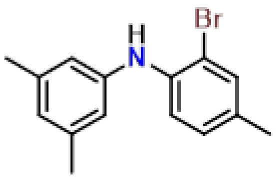

*Figure S 226: Hi – Res Mass (ESI⁺) of **2d**.*

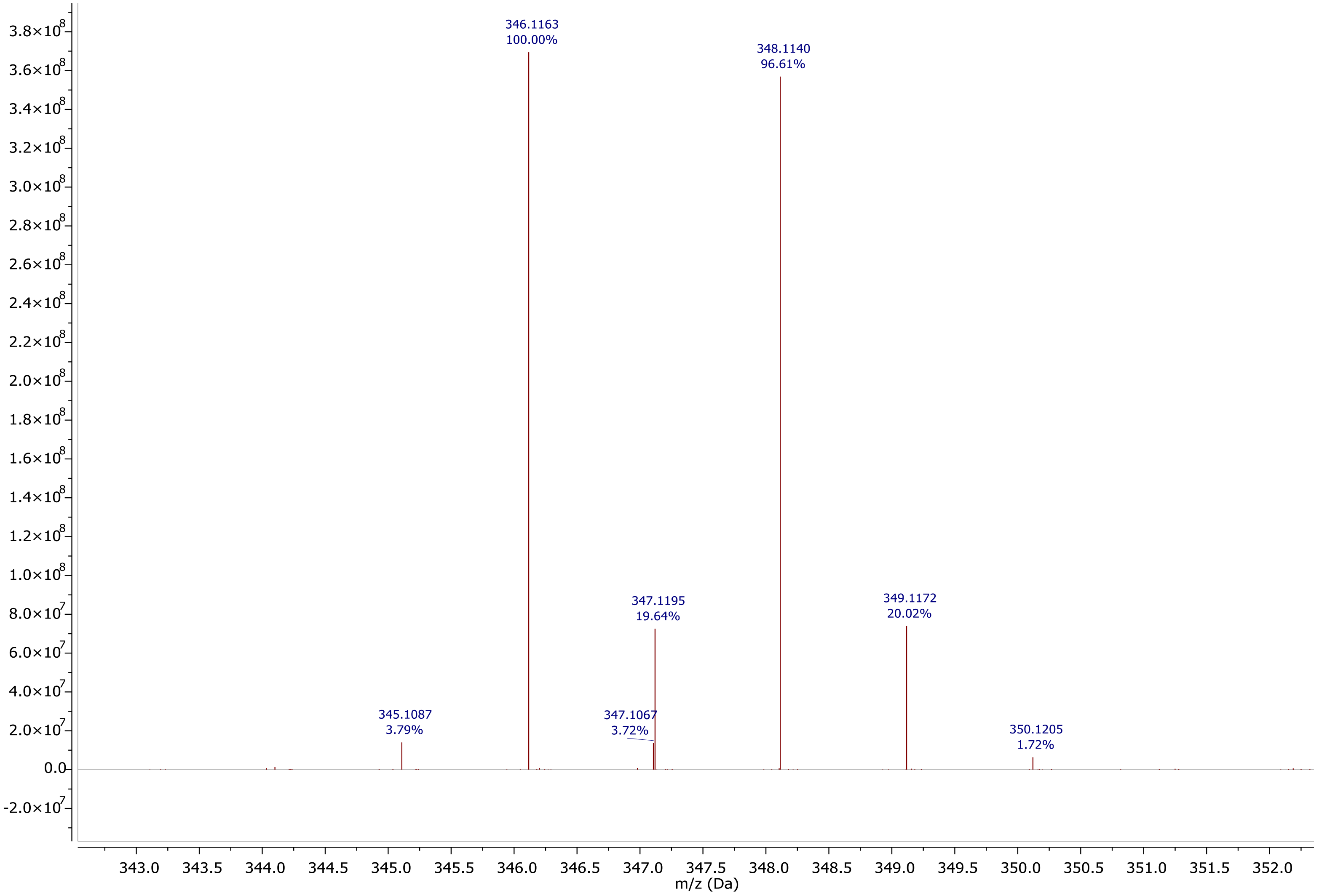

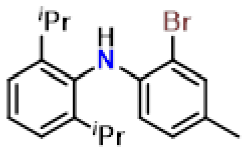

*Figure S 227: Hi – Res Mass (ESI+) of **2e**.*

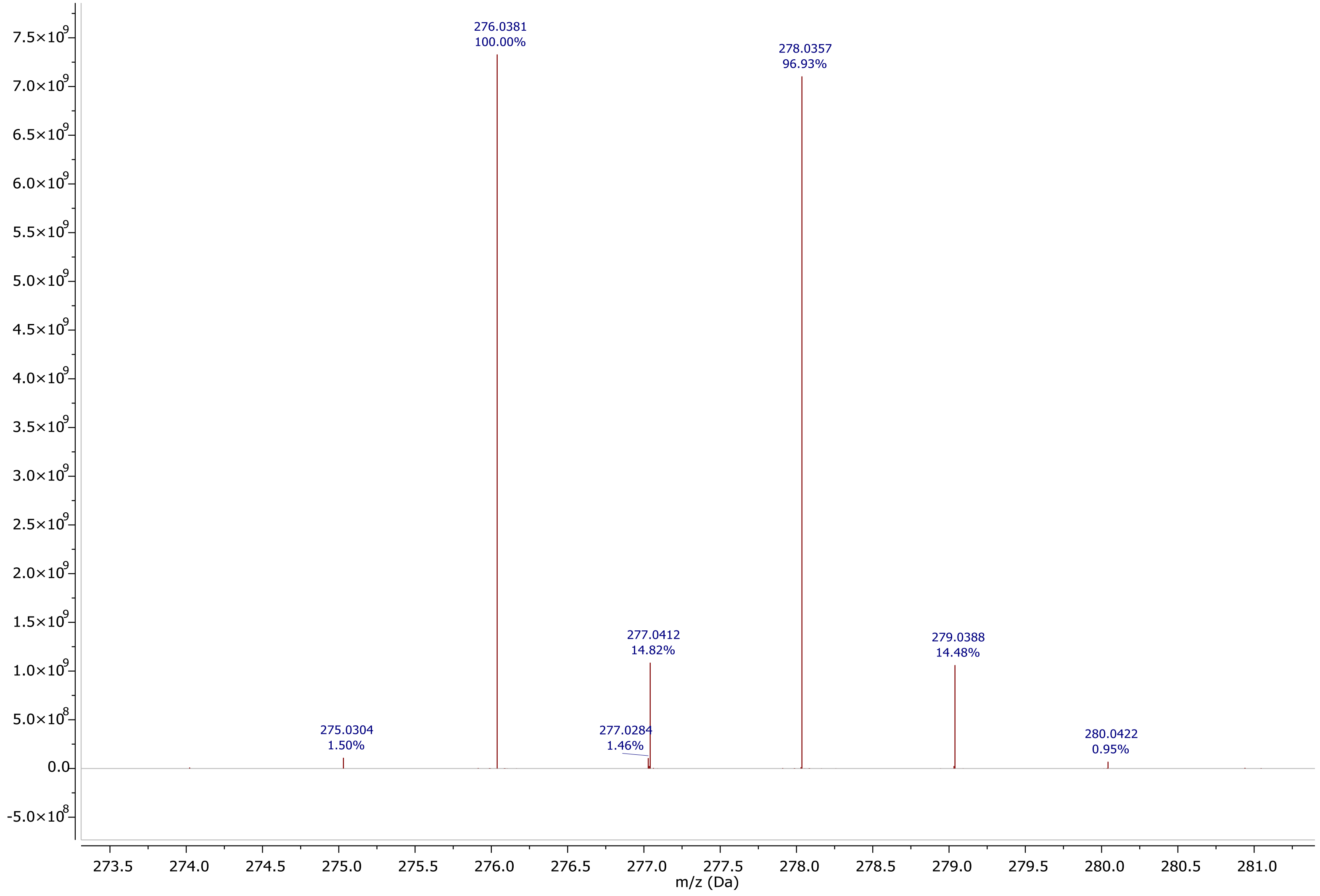

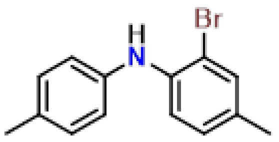

*Figure S 228: Hi – Res Mass (ESI+) of **2f**.*

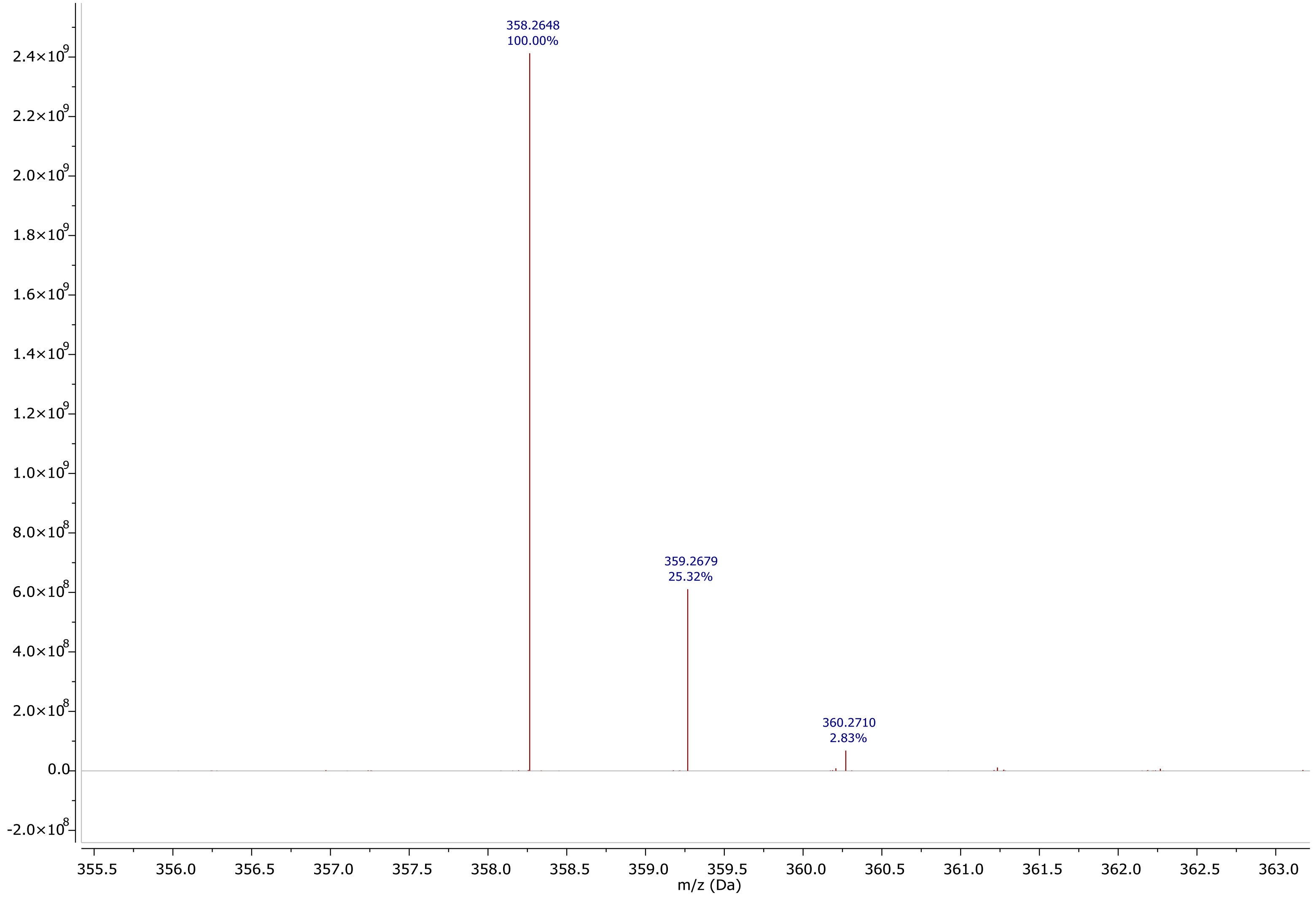

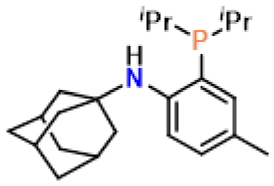

Figure S 229: Hi – Res Mass (ESI+) of **HPN^{Ad}**.

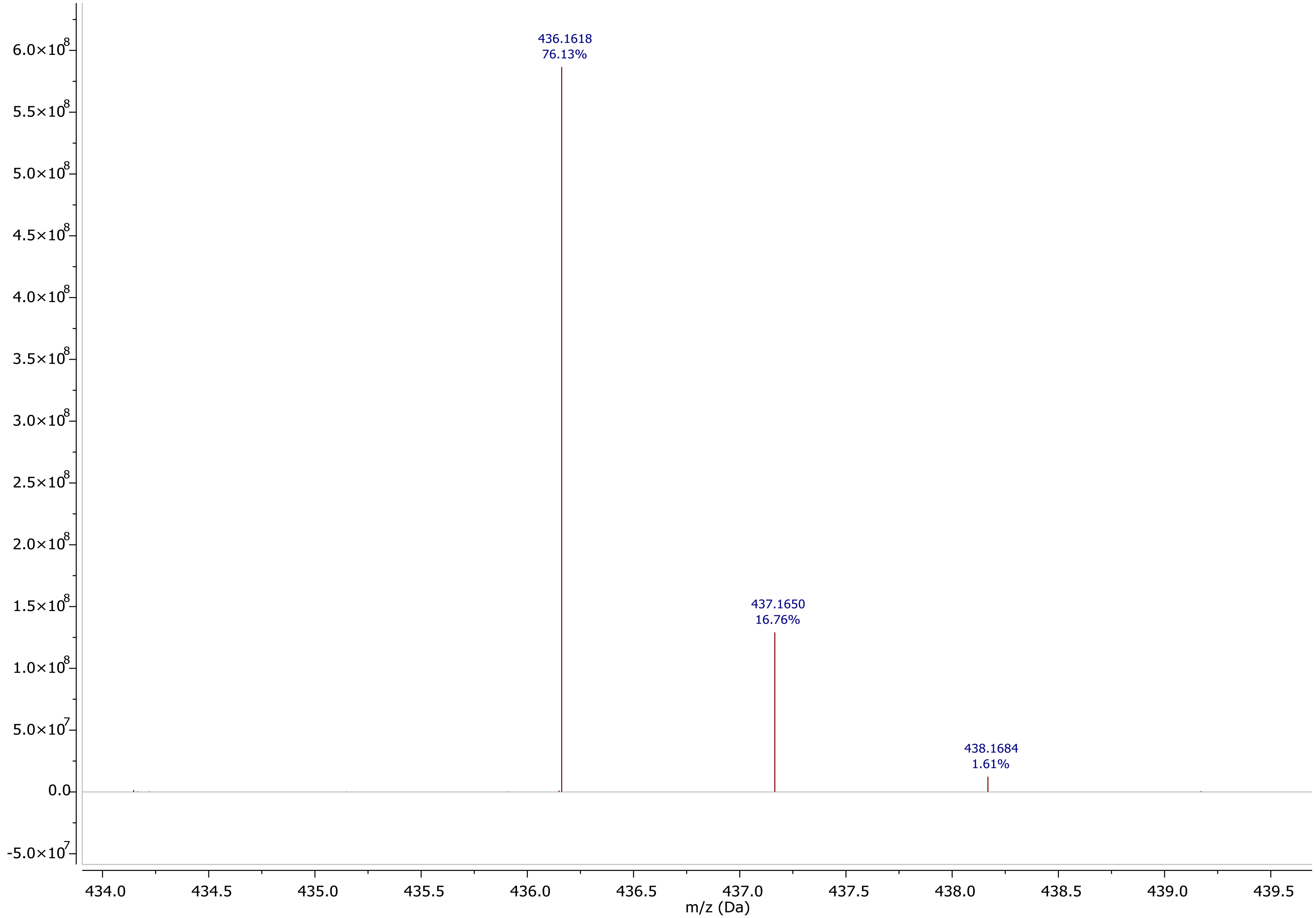

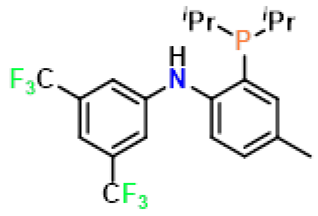

Figure S 230: Hi – Res Mass (ESI+) of ***HPN3,5CF3****.*

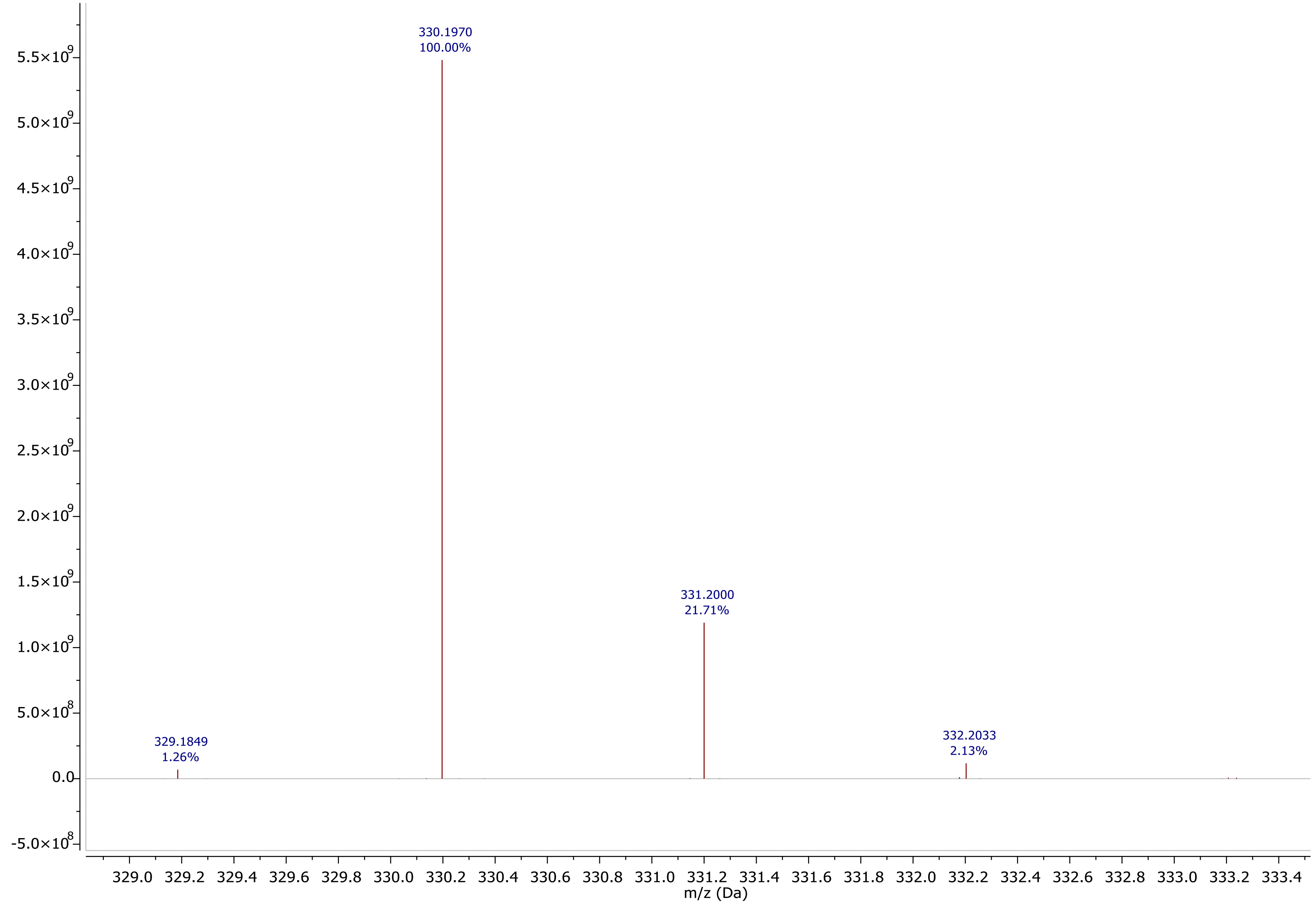

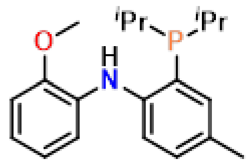

*Figure S 231: Hi – Res Mass (ESI$^+$) of **HPN^{OMe}**.*

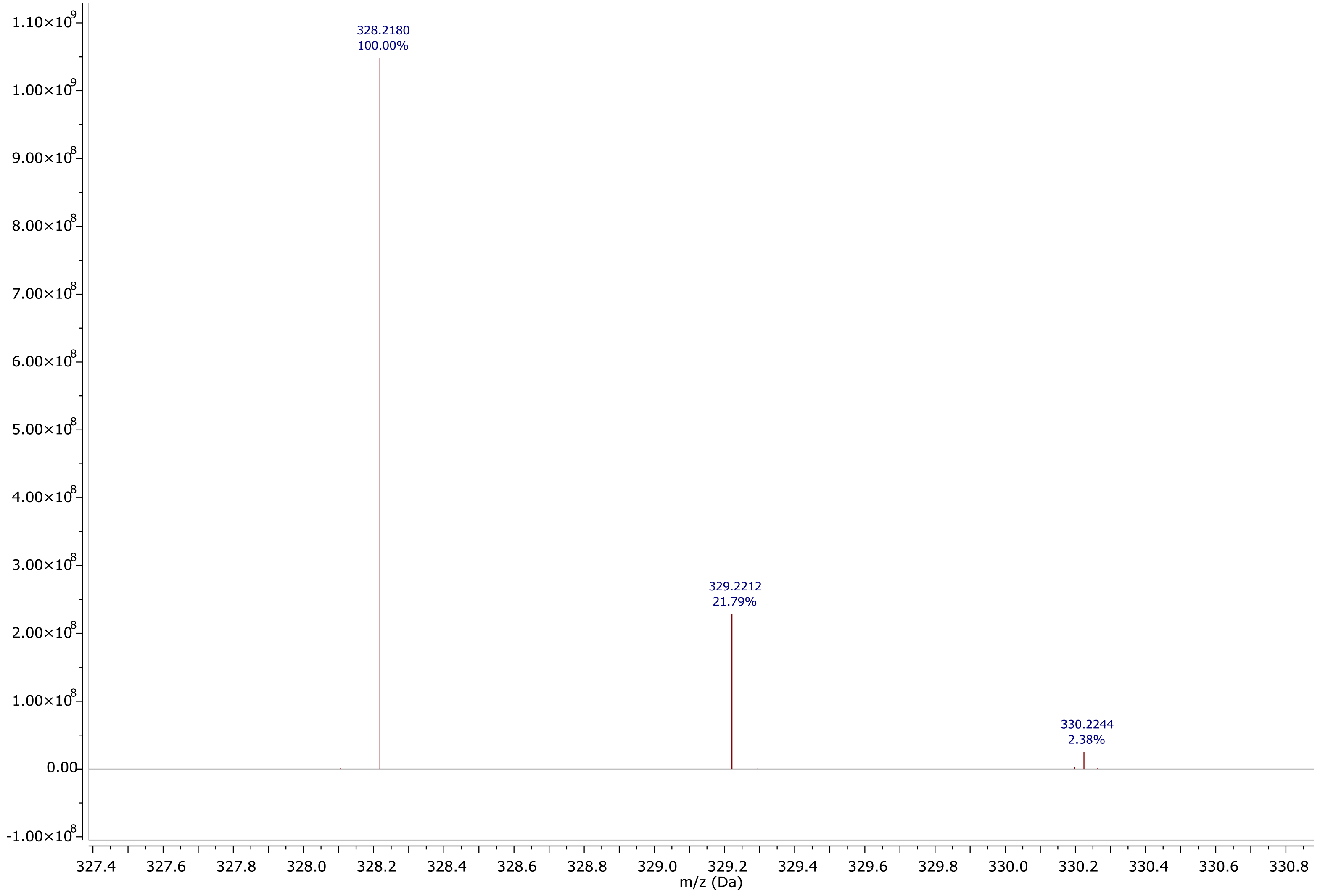

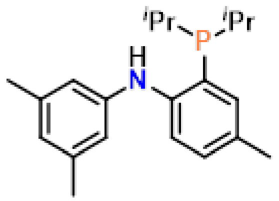

*Figure S 232: Hi – Res Mass (ESI$^+$) of **HPN^{3,5Me}**.*

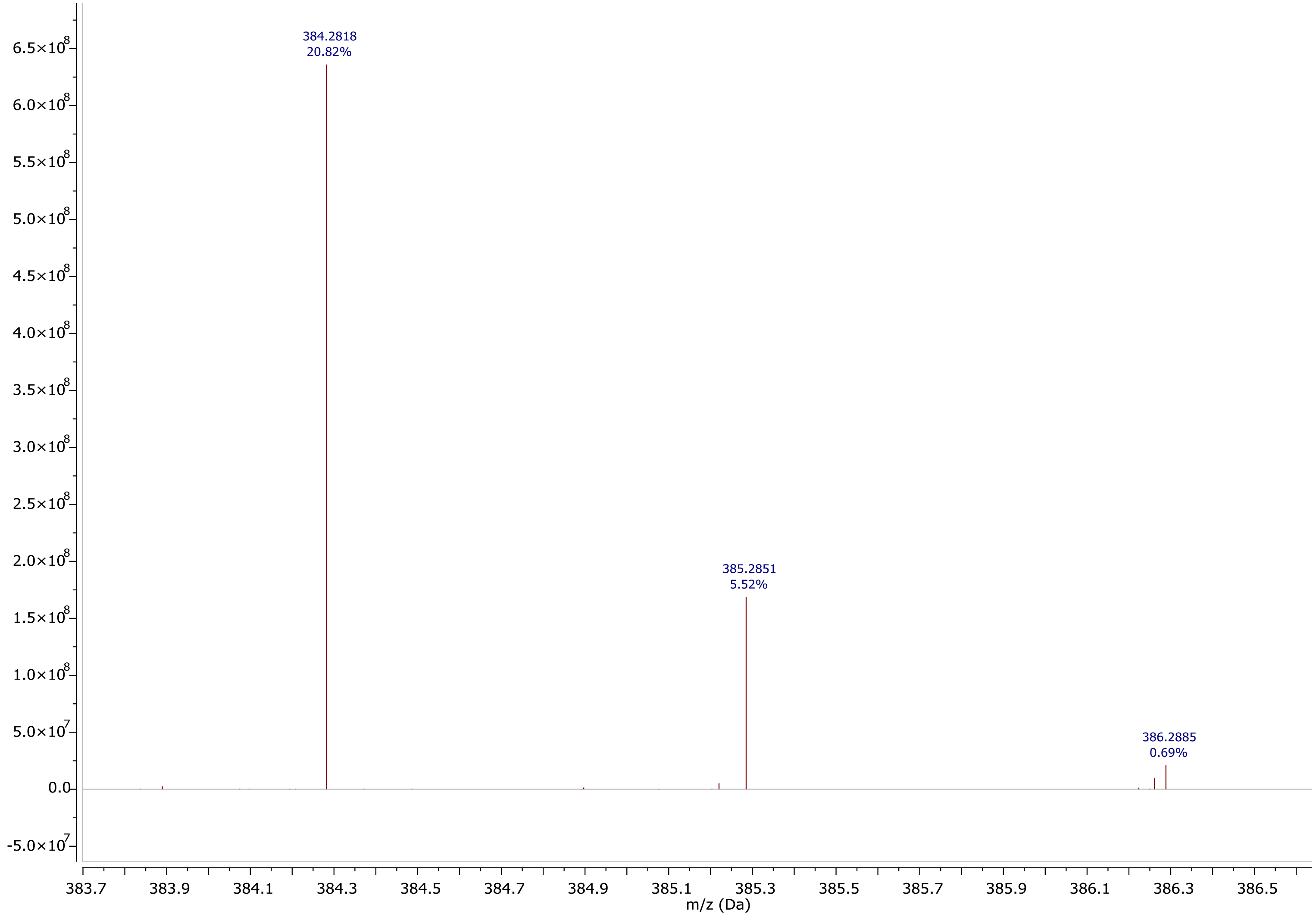

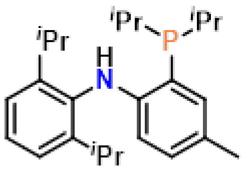

*Figure S 233: Hi – Res Mass (ESI+) of **HPN^{Dipp}**.*

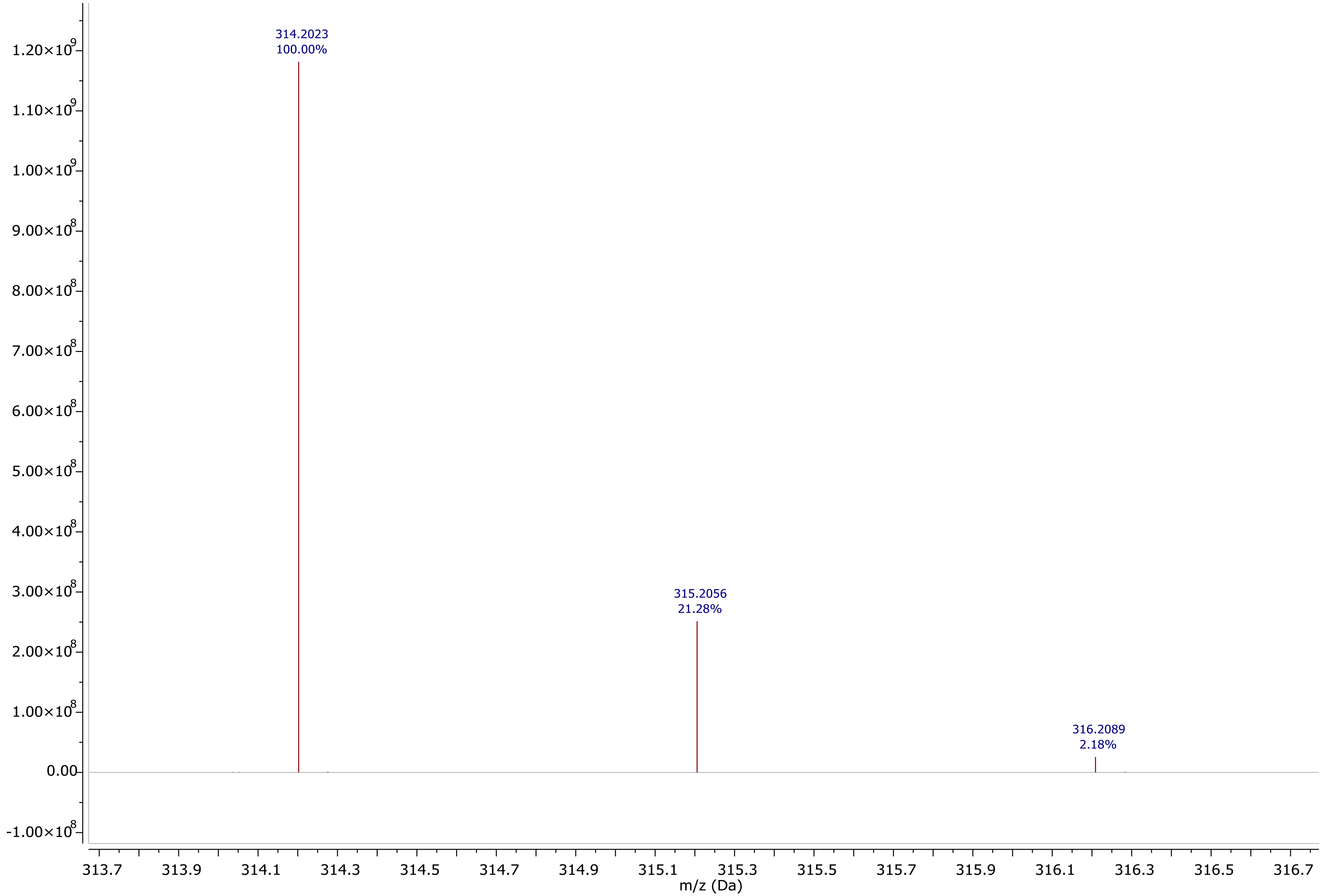

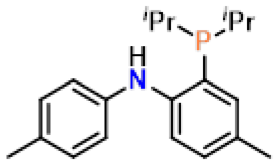

*Figure S 234: Hi – Res Mass (ESI$^+$) of **HPN^{Tol}**.*

UV-Vis-NIR spectra

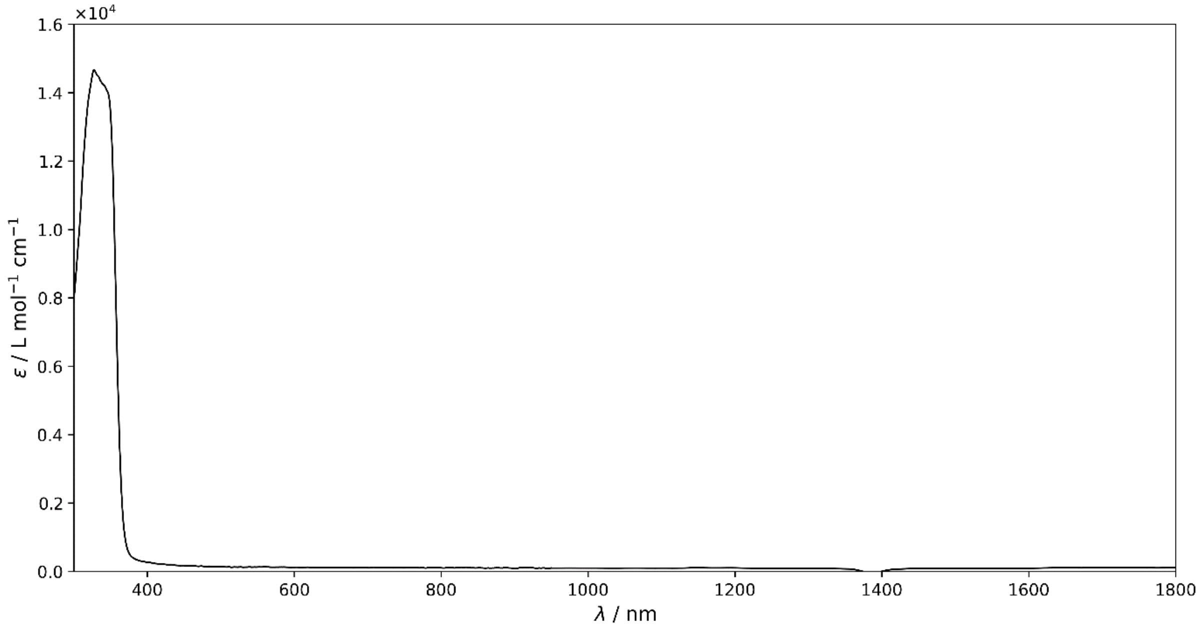

Figure S 235: UV-Vis-NIR spectrum of ***HPN**^{Ad}* *in toluene at 298 K.*

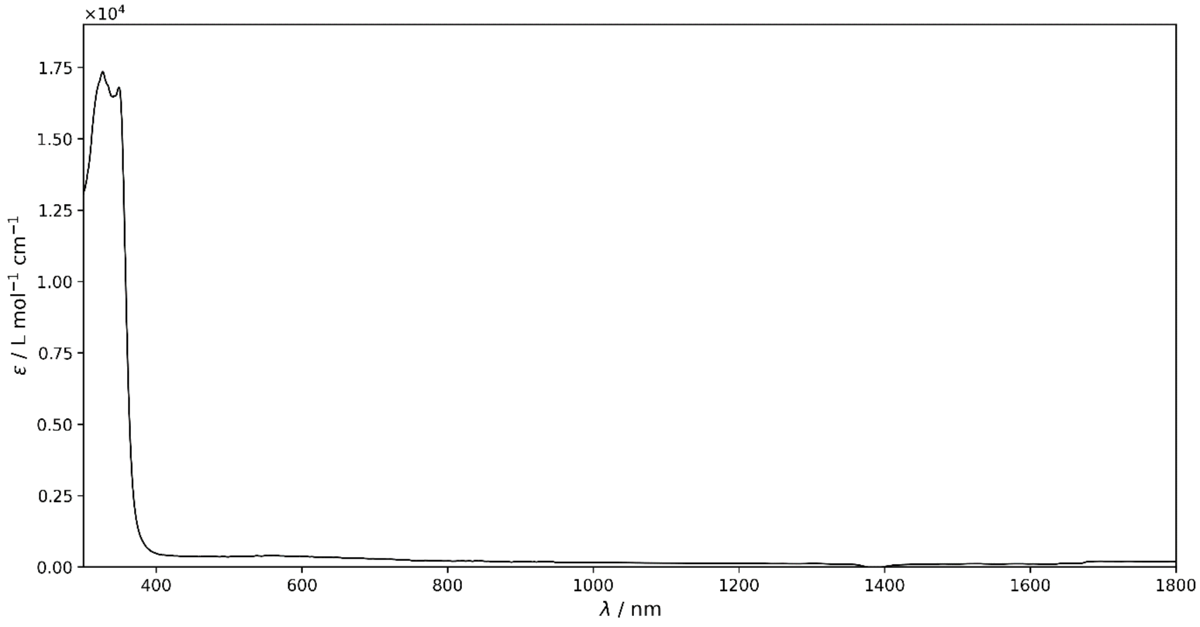

Figure S 236: UV-Vis-NIR spectrum of ***HPN**^{3,5CF3}* *in toluene at 298 K.*

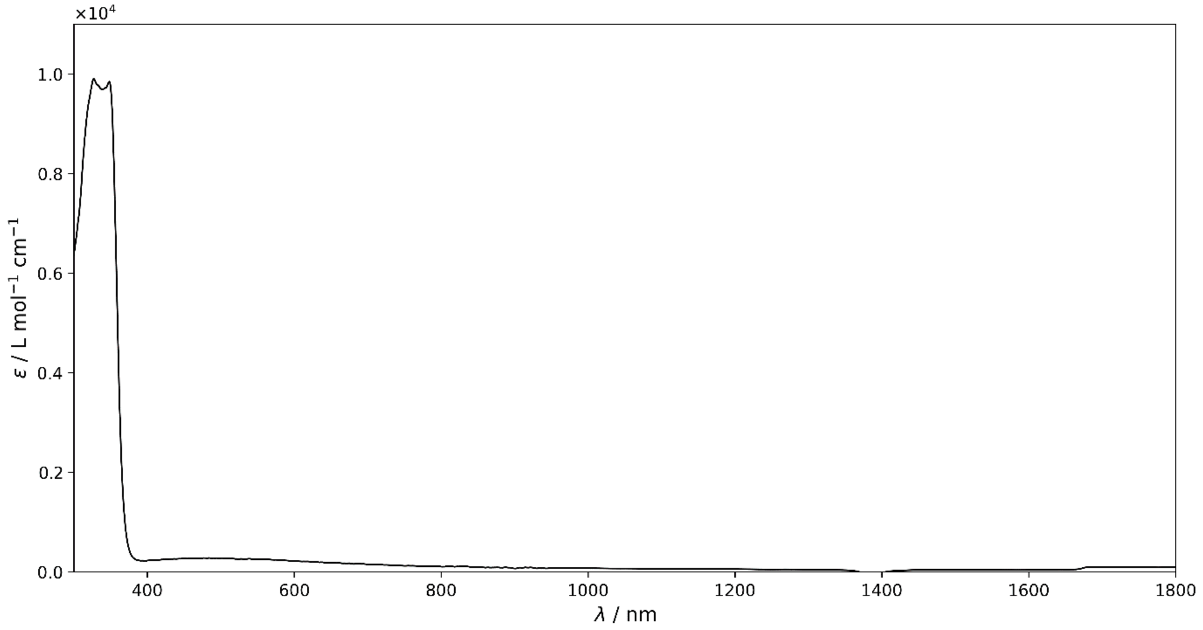

Figure S 237: UV-Vis-NIR spectrum of ***HPN***$^{\textbf{\textit{OMe}}}$ *in toluene at 298 K.*

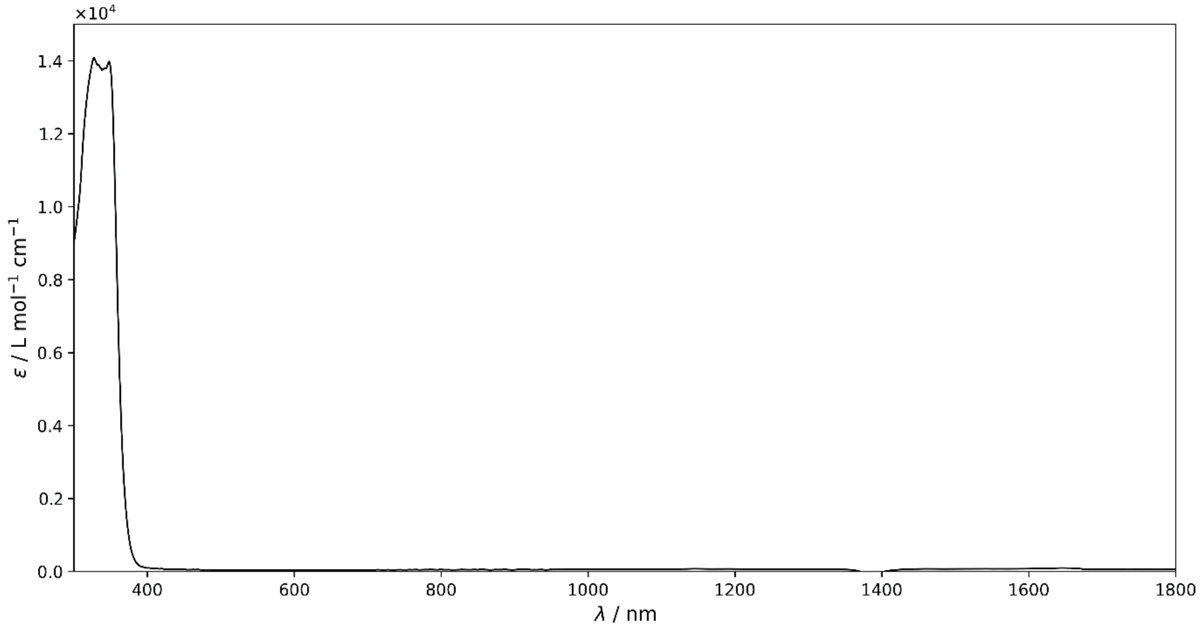

Figure S 238: UV-Vis-NIR spectrum of ***HPN***$^{\textbf{\textit{3,5Me}}}$ *in toluene at 298 K.*

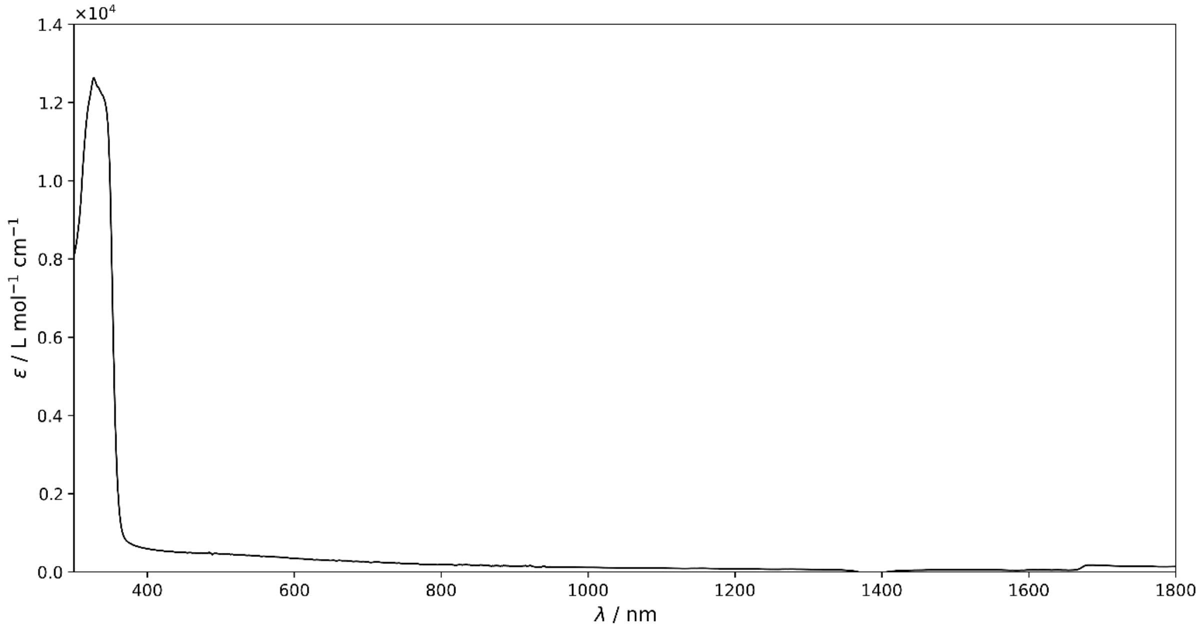

*Figure S 239: UV-Vis-NIR spectrum of **HPN^{Dipp}** in toluene at 298 K.*

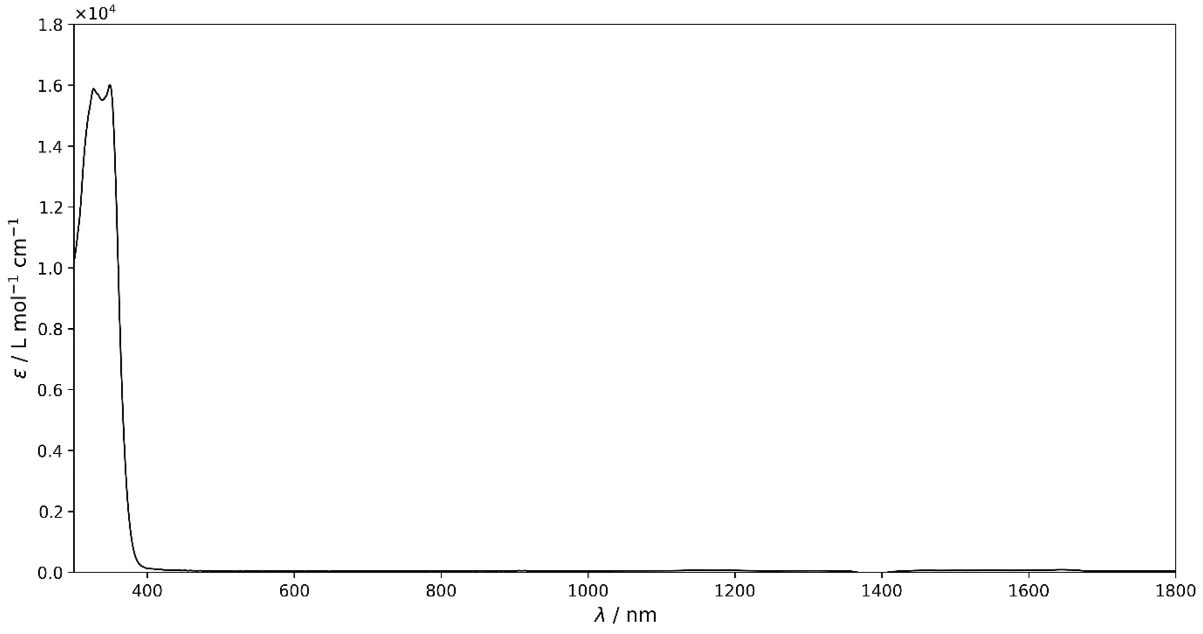

*Figure S 240: UV-Vis-NIR spectrum of **HPN^{Tol}** in toluene at 298 K.*

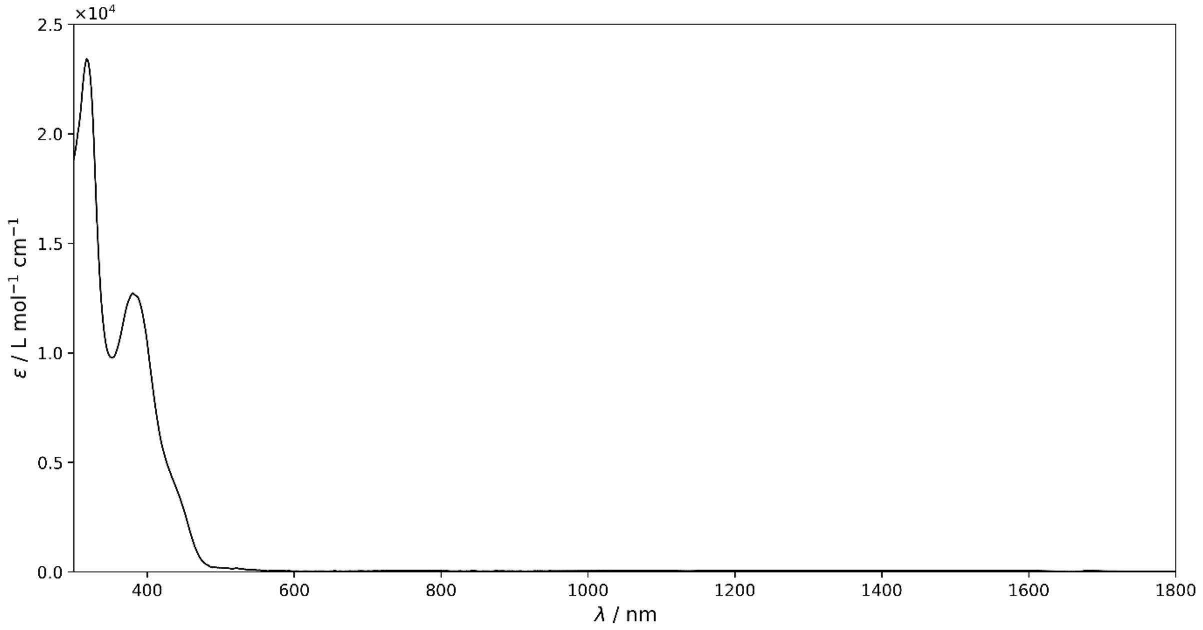

*Figure S 241: UV-Vis-NIR spectrum of **LiPN^{Ad}** in toluene at 298 K.*

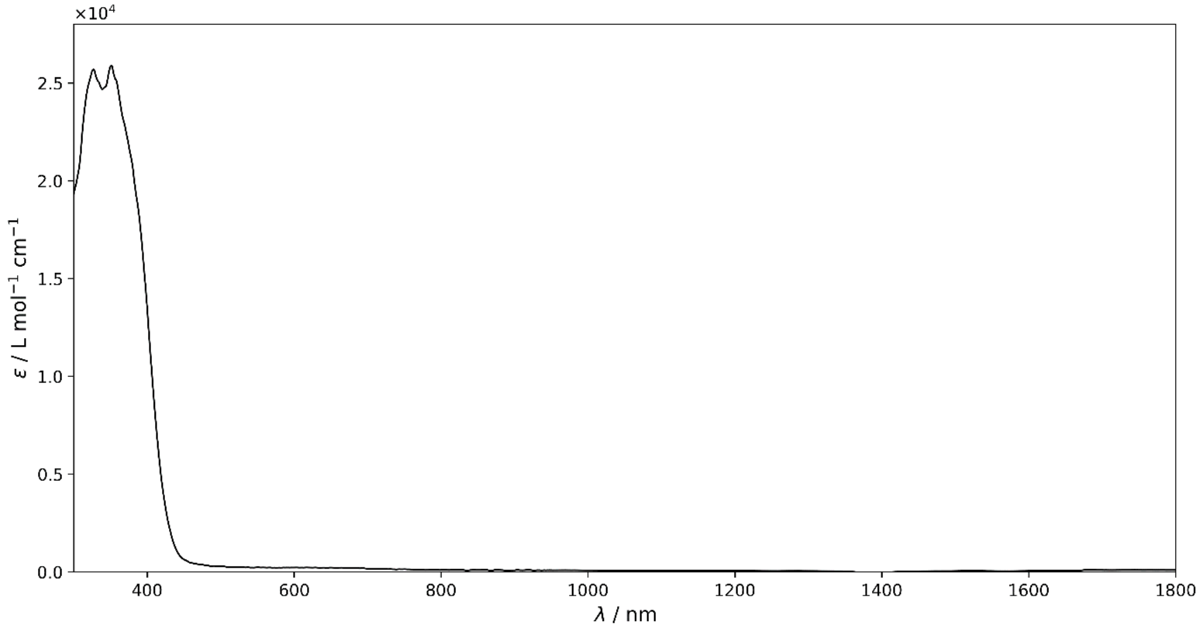

*Figure S 242: UV-Vis-NIR spectrum of **LiPN^{3,5CF3}** in toluene at 298 K.*

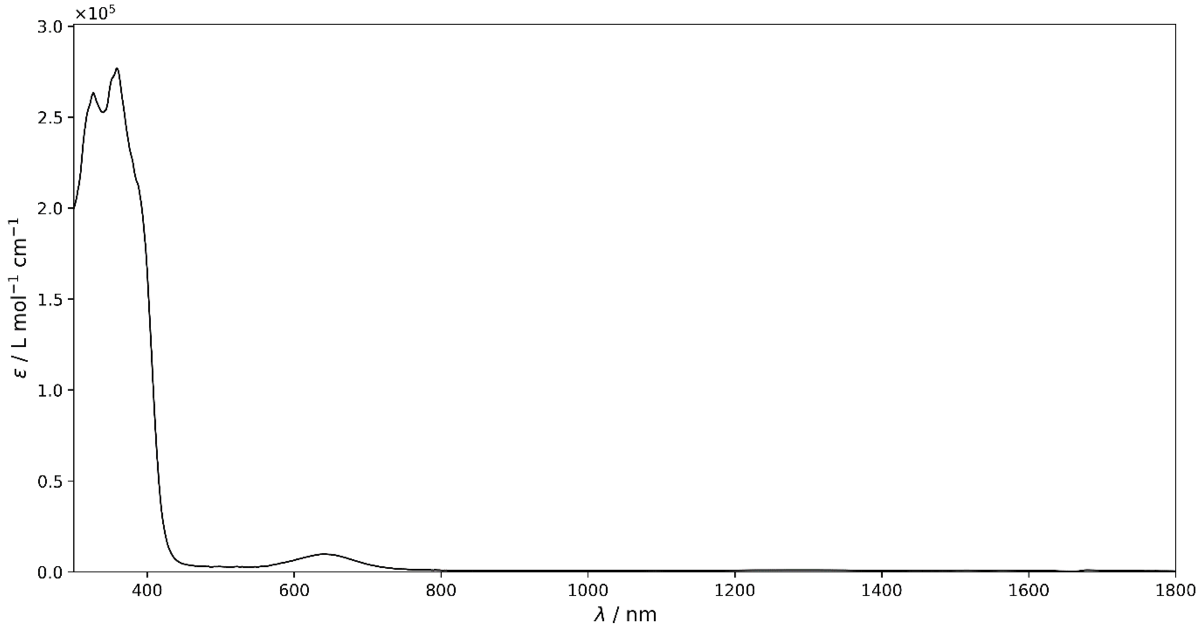

Figure S 243: UV-Vis-NIR spectrum of ***LiPN***$^{\textit{\textbf{OMe}}}$ *in toluene at 298 K.*

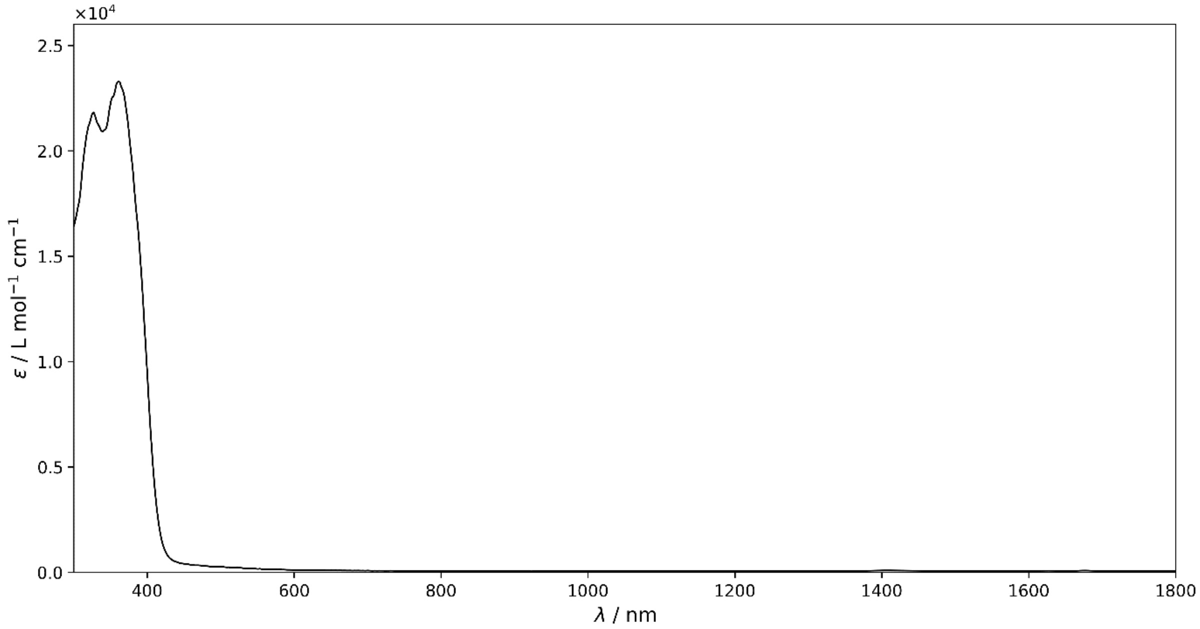

Figure S 244: UV-Vis-NIR spectrum of ***LiPN***$^{\textit{\textbf{3,5Me}}}$ *in toluene at 298 K.*

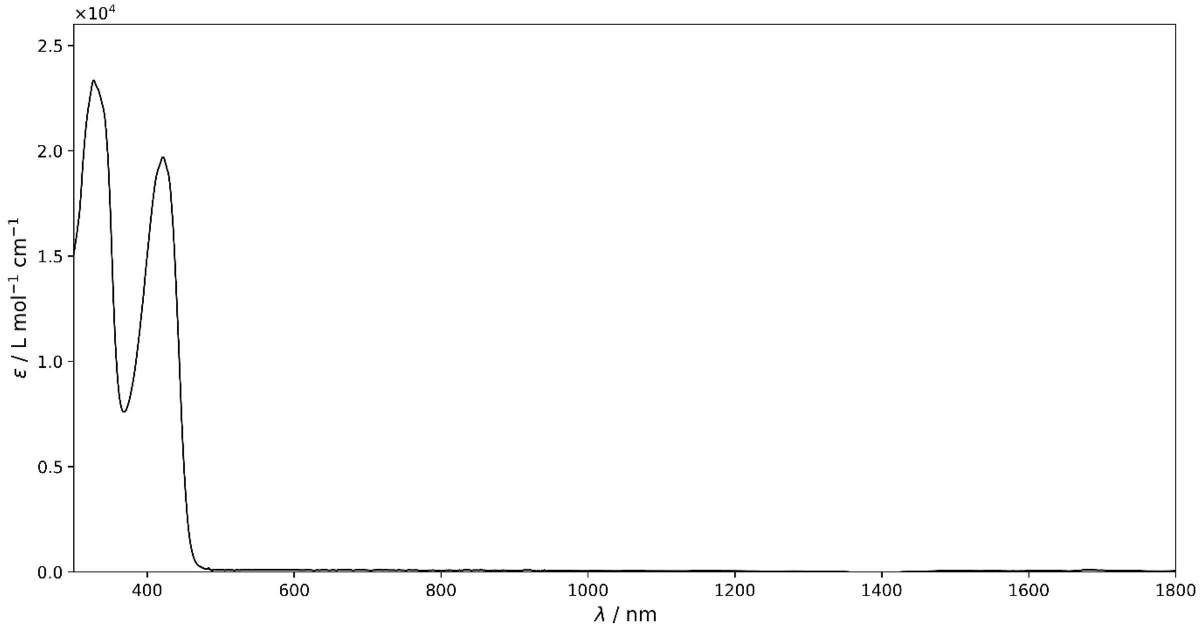

Figure S 245: UV-Vis-NIR spectrum of ***LiPN^{Dipp}*** *in toluene at 298 K.*

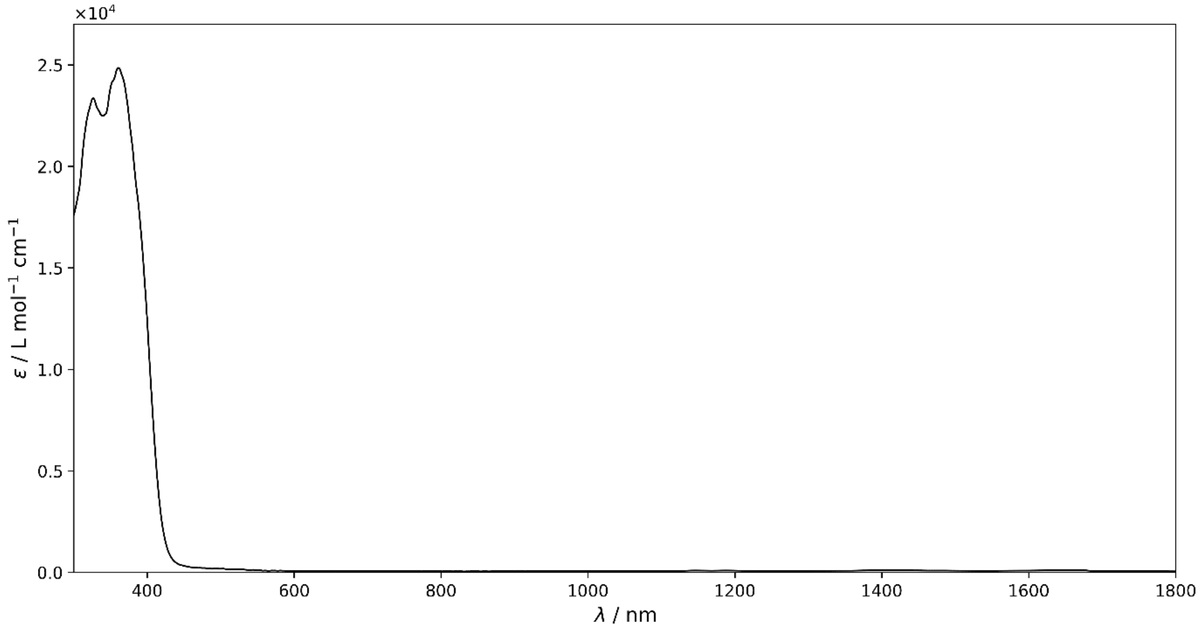

Figure S 246: UV-Vis-NIR spectrum of ***LiPN^{Tol}*** *in toluene at 298 K.*

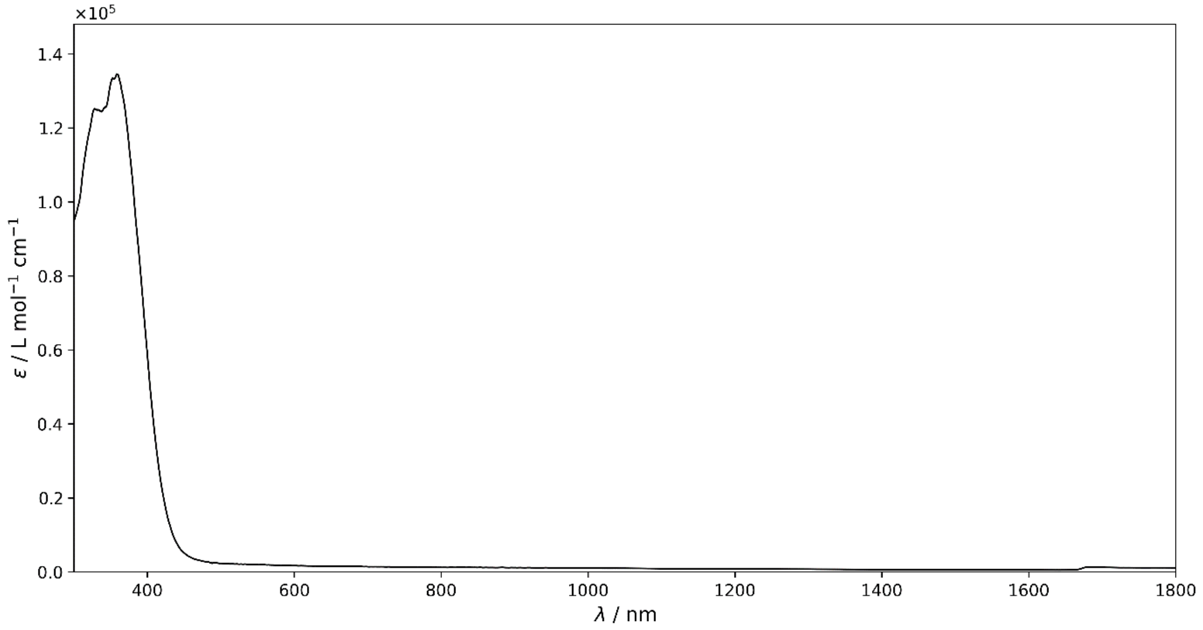

Figure S 247: UV-Vis-NIR spectrum of ***3a*** *in toluene at 298 K.*

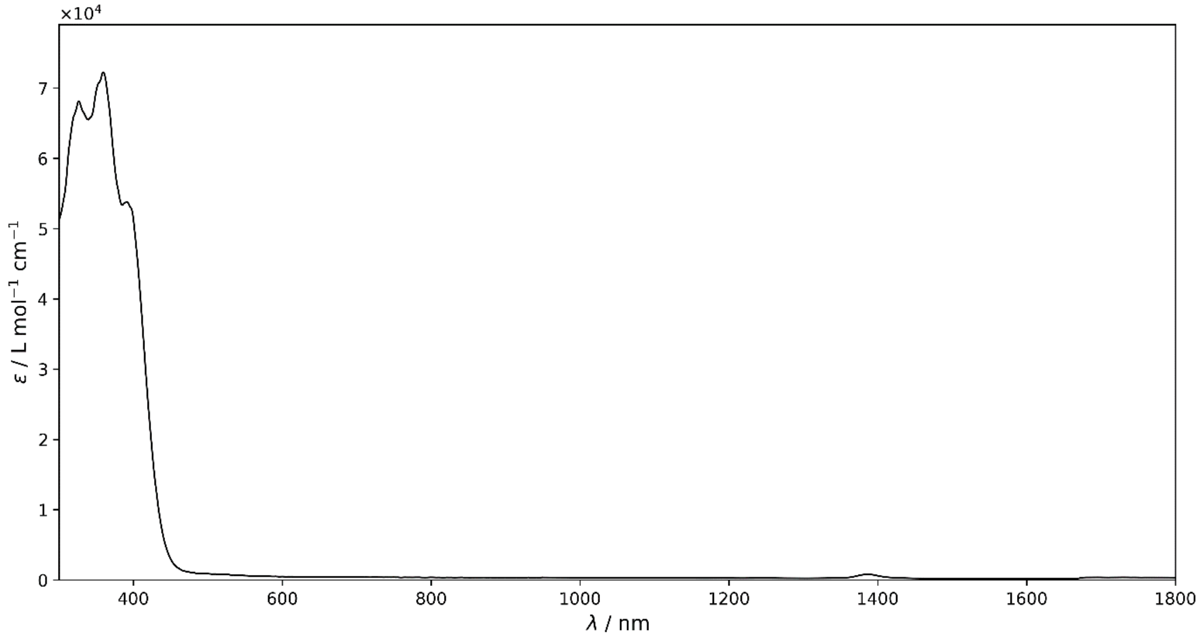

Figure S 248: UV-Vis-NIR spectrum of ***3d*** *in toluene at 298 K.*

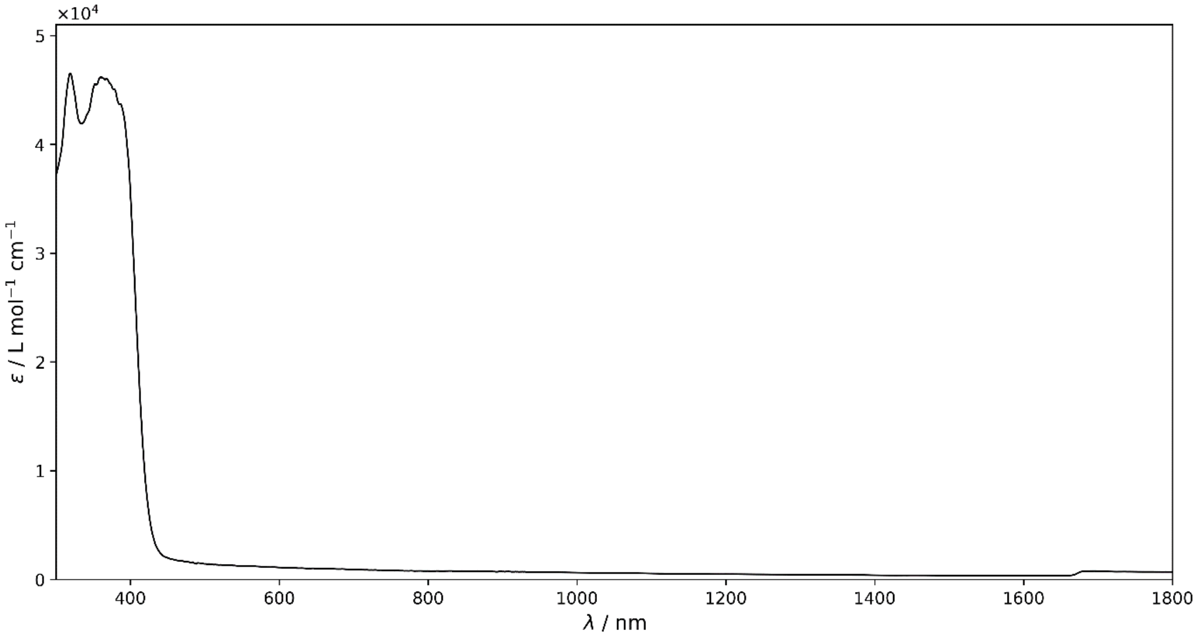

*Figure S 249: UV-Vis-NIR spectrum of **3e** in toluene at 298 K.*

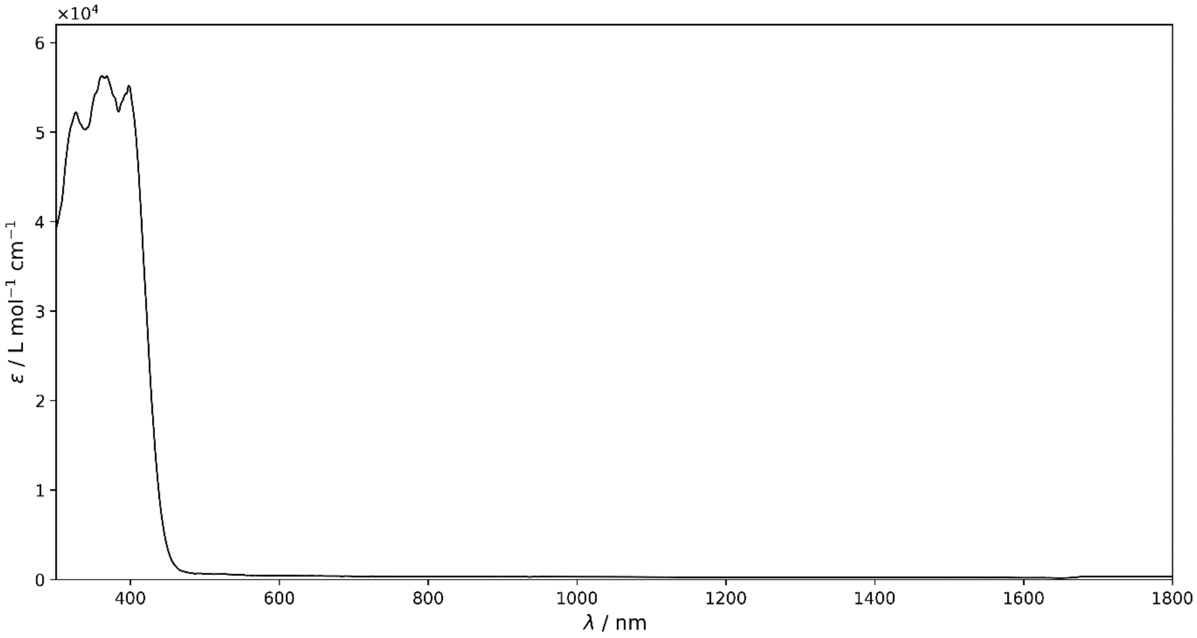

*Figure S 250: UV-Vis-NIR spectrum of **3f** in toluene at 298 K.*

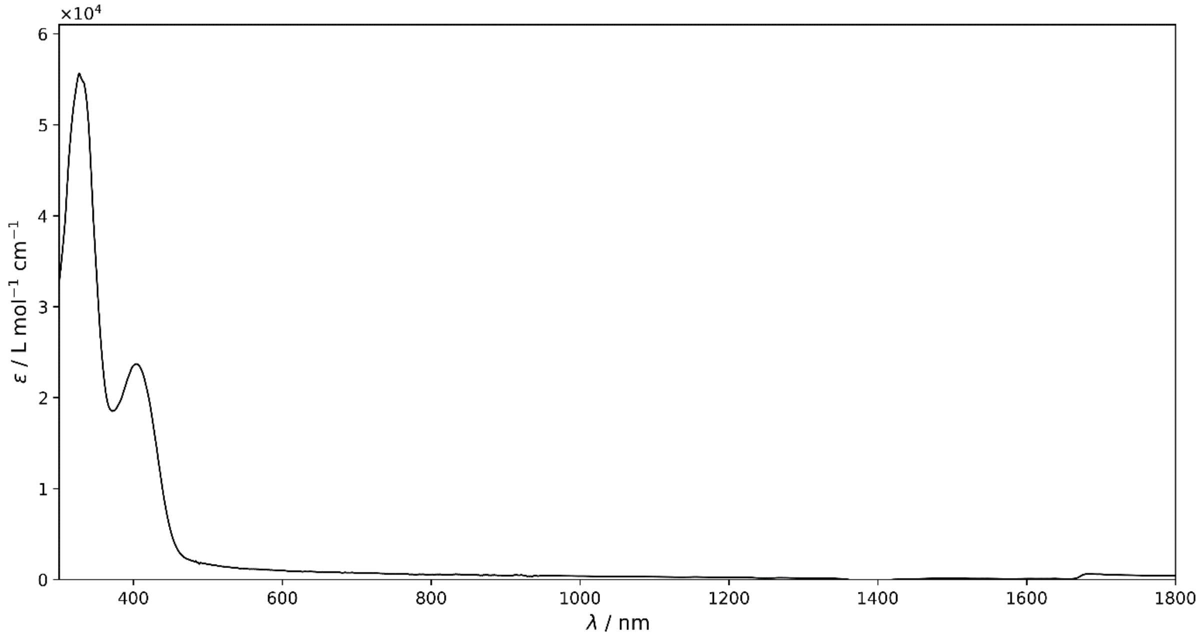

*Figure S 251: UV-Vis-NIR spectrum of **4a** in toluene at 298 K.*

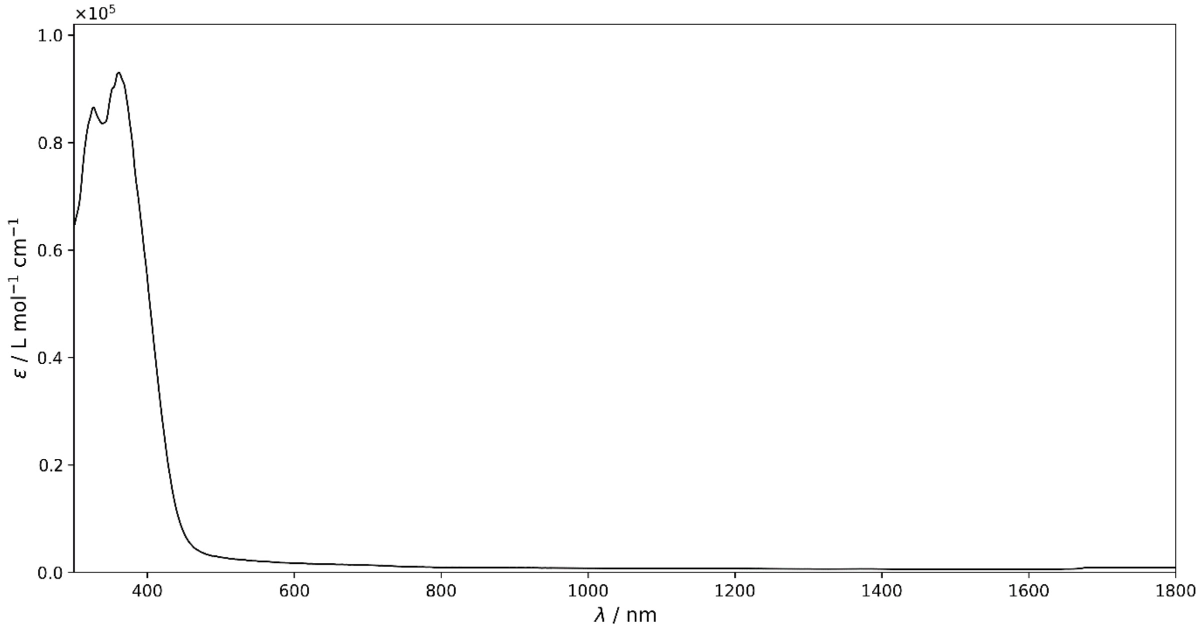

*Figure S 252: UV-Vis-NIR spectrum of **4b** in toluene at 298 K.*

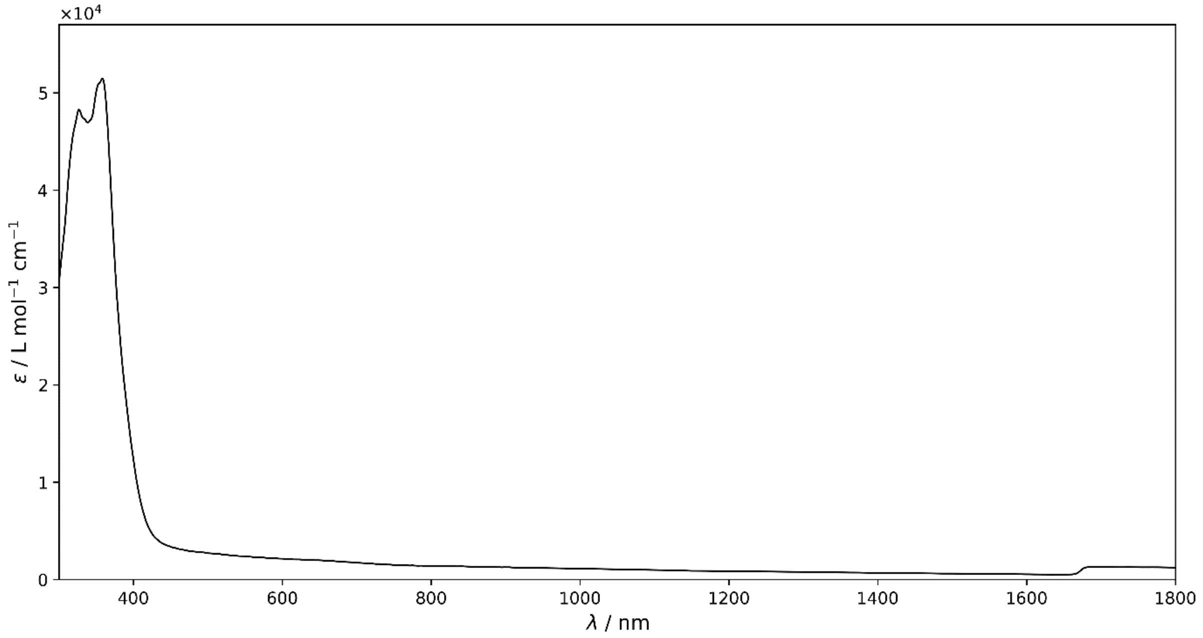

Figure S 253: UV-Vis-NIR spectrum of ***4c*** *in toluene at 298 K.*

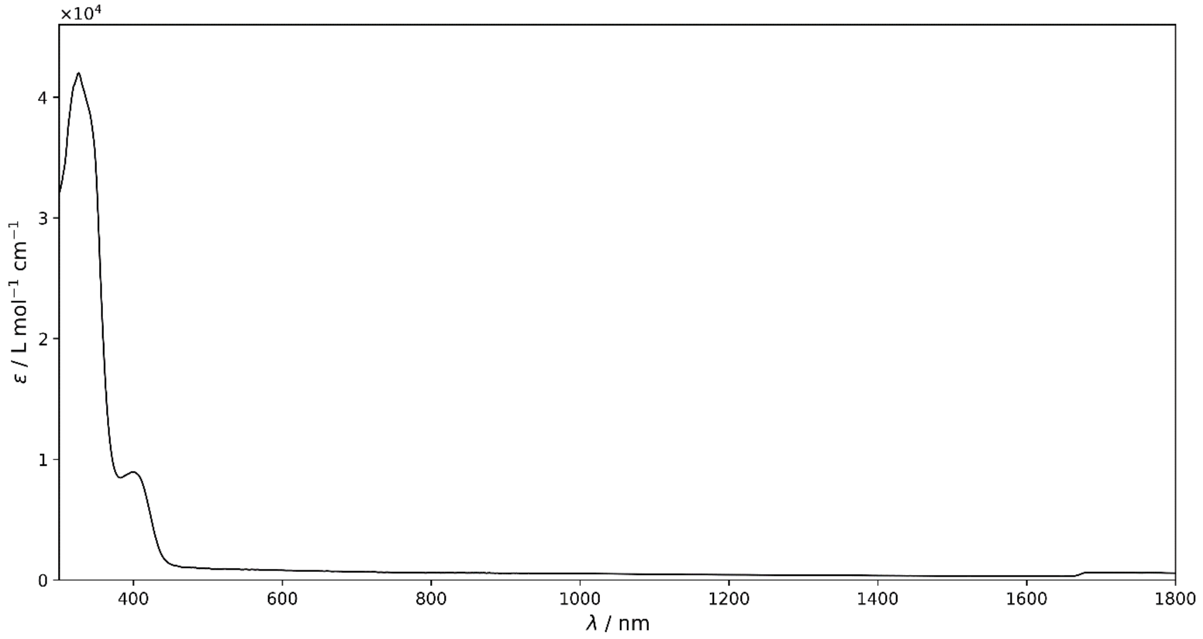

Figure S 254: UV-Vis-NIR spectrum of ***4d*** *in toluene at 298 K.*

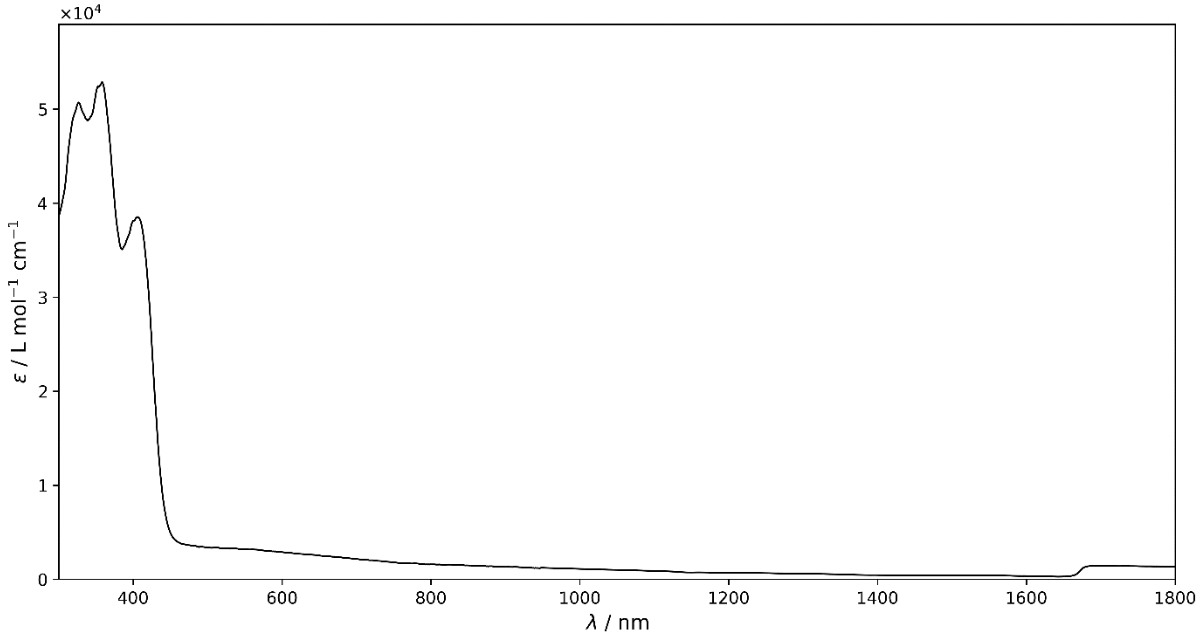

Figure S 255: UV-Vis-NIR spectrum of ***4f*** *in toluene at 298 K.*

IR spectra

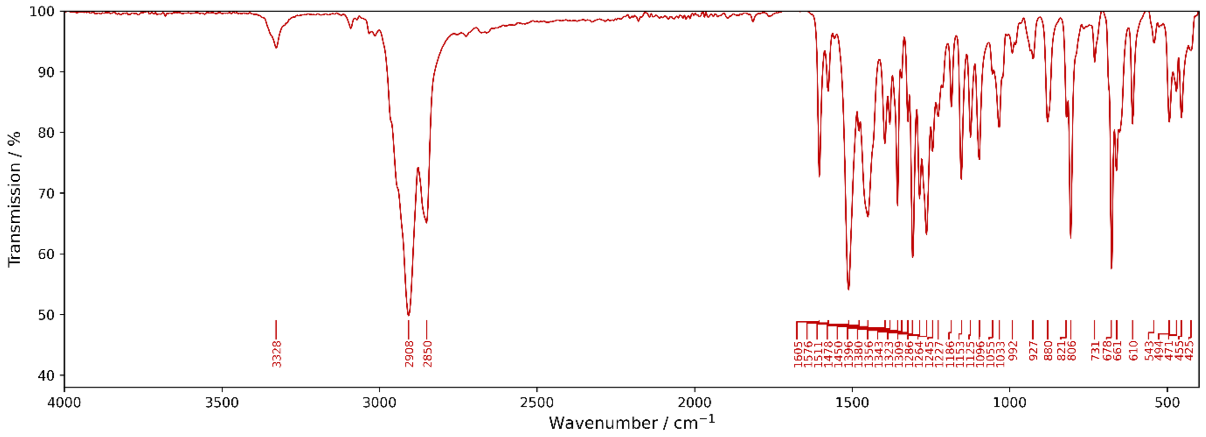

*Figure S 256: ATR-IR spectrum of **HPN^{Ad}**.*

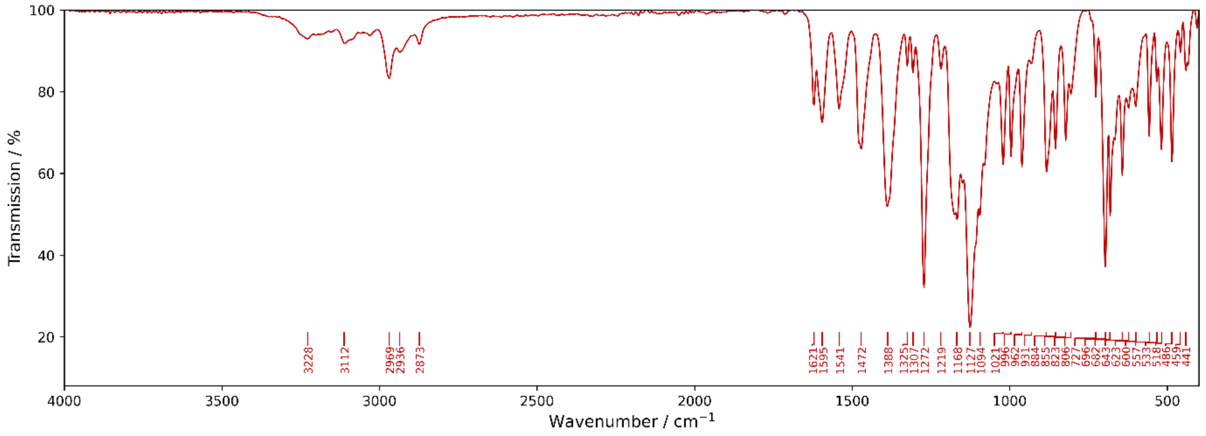

*Figure S 257: ATR-IR spectrum of **HPN^{3,5CF3}**.*

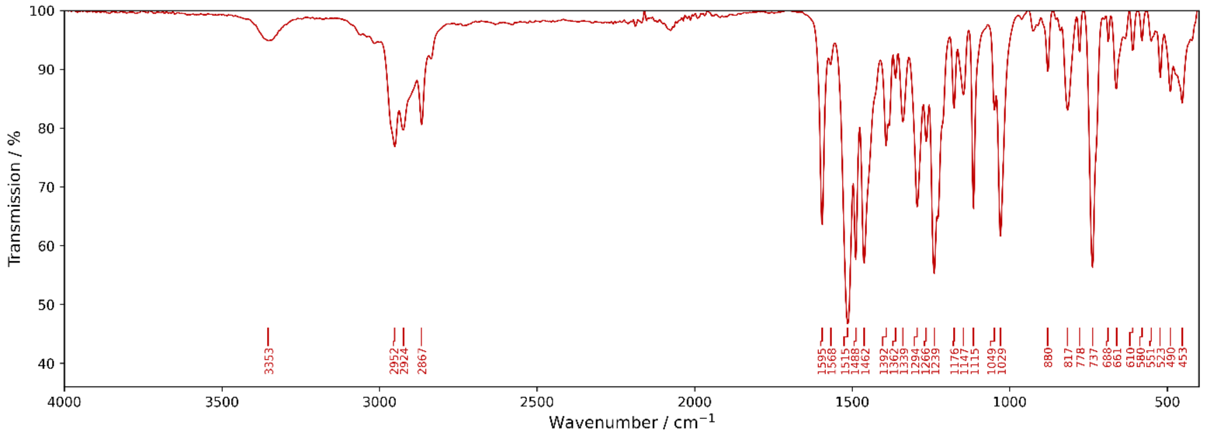

*Figure S 258: ATR-IR spectrum of **HPN^{OMe}**.*

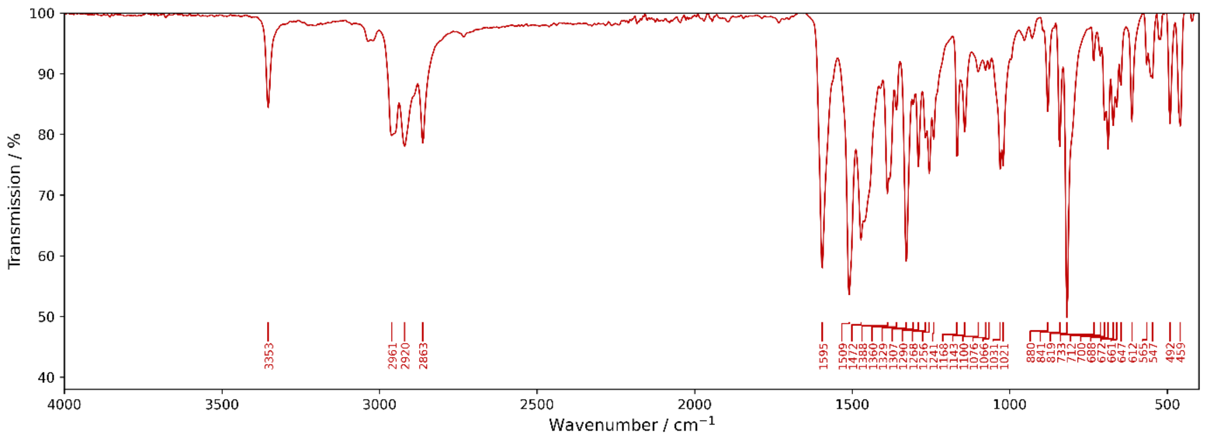

Figure S 259: ATR-IR spectrum of ***HPN***$^{\mathit{3,5Me}}$.

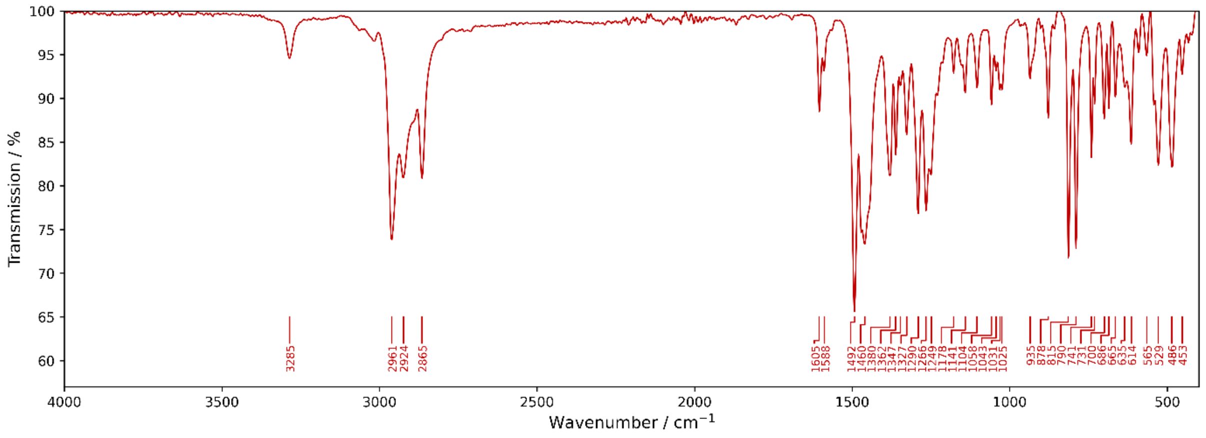

Figure S 260: ATR-IR spectrum of ***HPN***^{Dipp}.

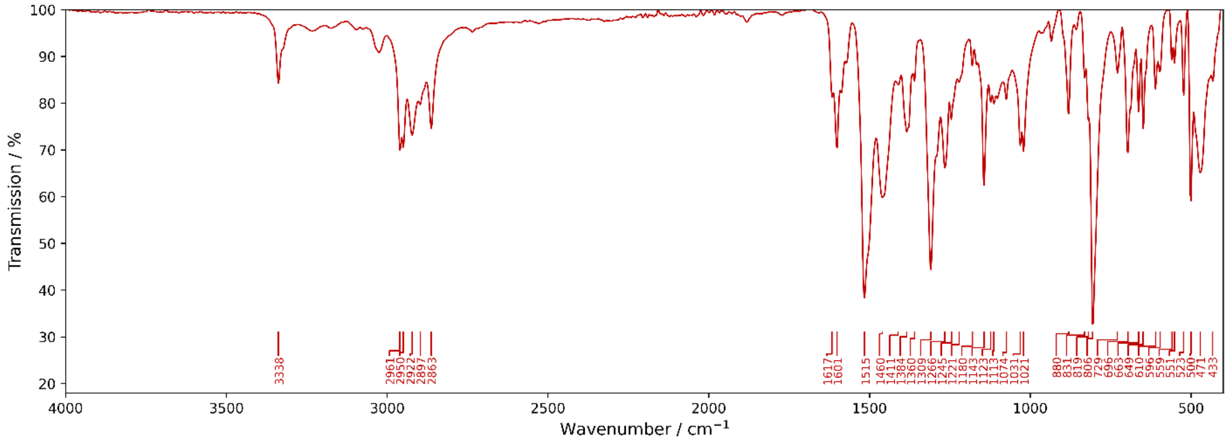

Figure S 261: ATR-IR spectrum of ***HPN***^{Tol}.

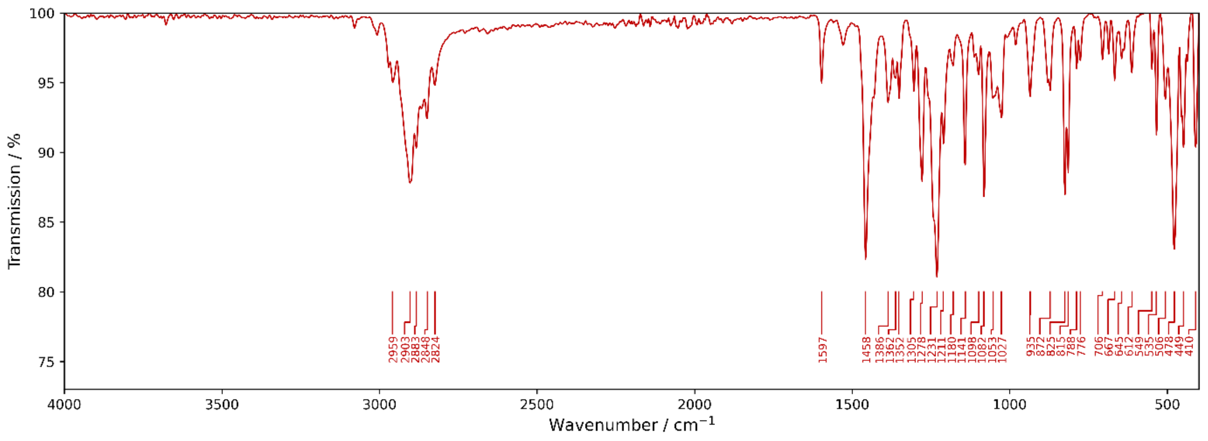

Figure S 262: ATR-IR spectrum of **LiPN**[Ad].

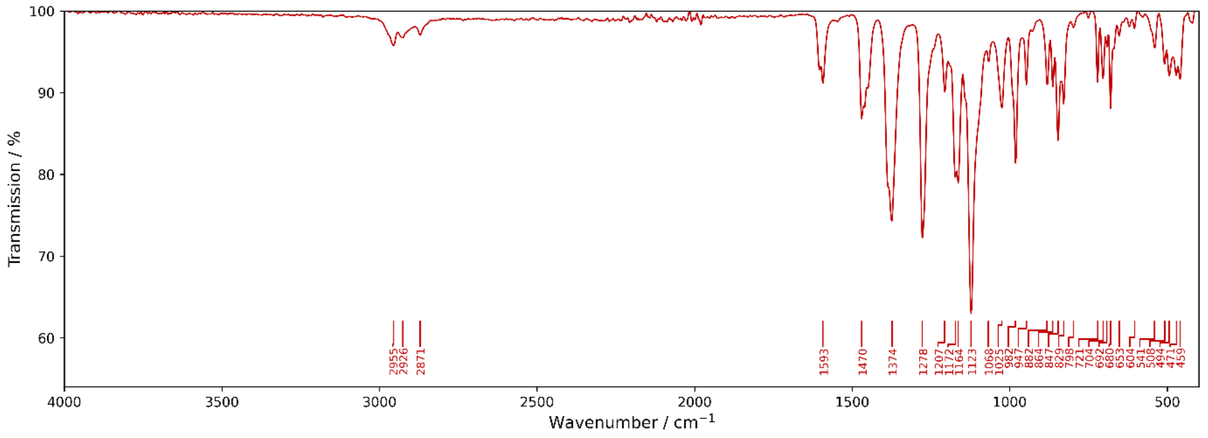

Figure S 263: ATR-IR spectrum of **LiPN**[3,5CF3].

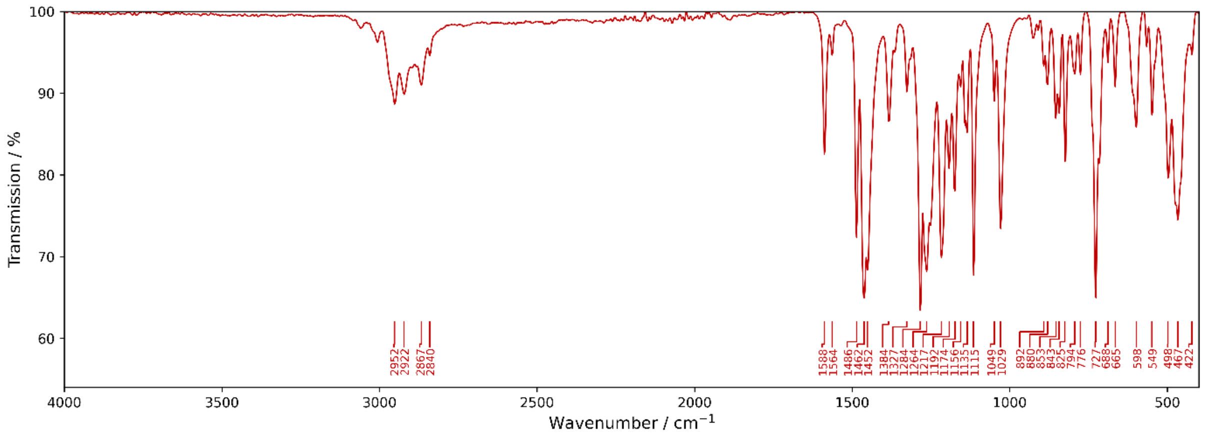

Figure S 264: ATR-IR spectrum of **LiPN**[OMe].

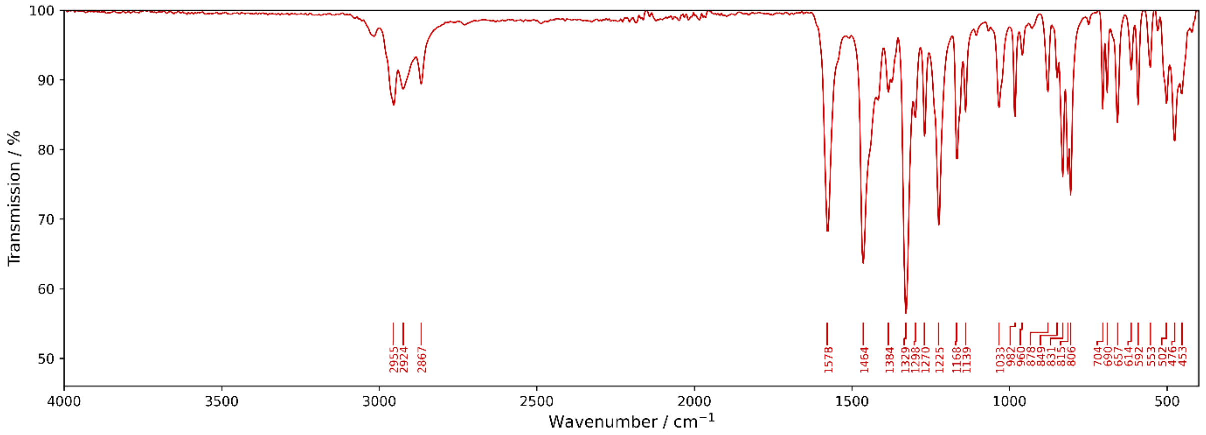

Figure S 265: ATR-IR spectrum of ***LiPN***$^{\boldsymbol{3,5Me}}$.

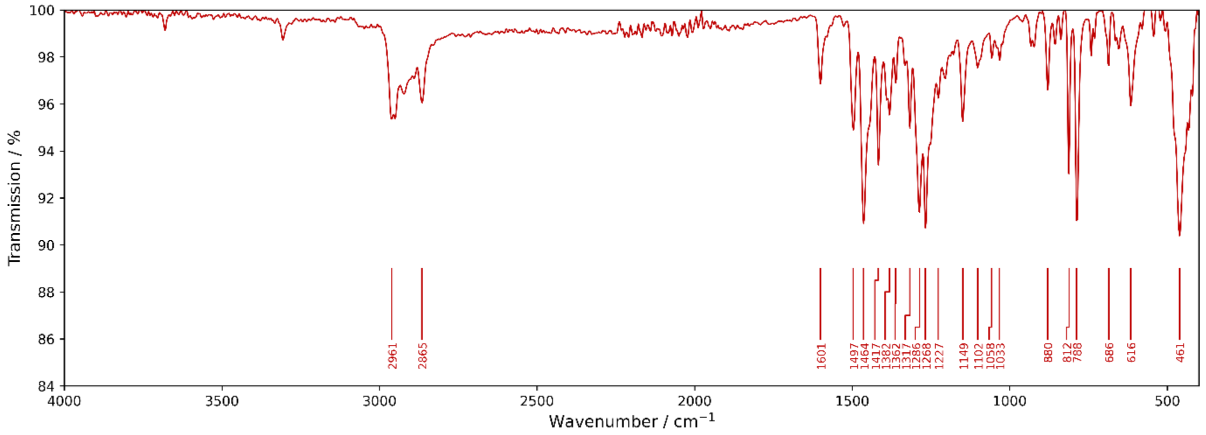

Figure S 266: ATR-IR spectrum of ***LiPN***$^{\boldsymbol{Dipp}}$.

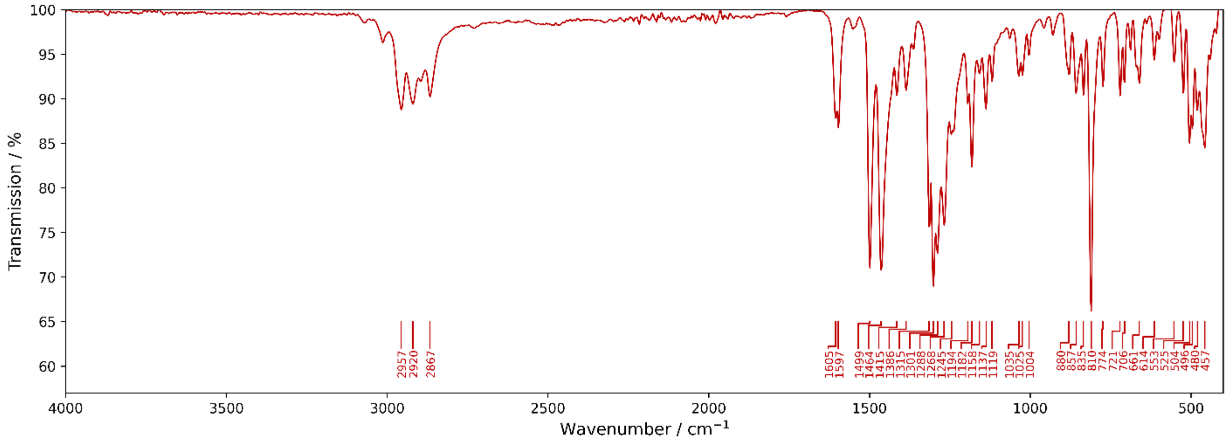

Figure S 267: ATR-IR spectrum of ***LiPN***$^{\boldsymbol{Tol}}$.

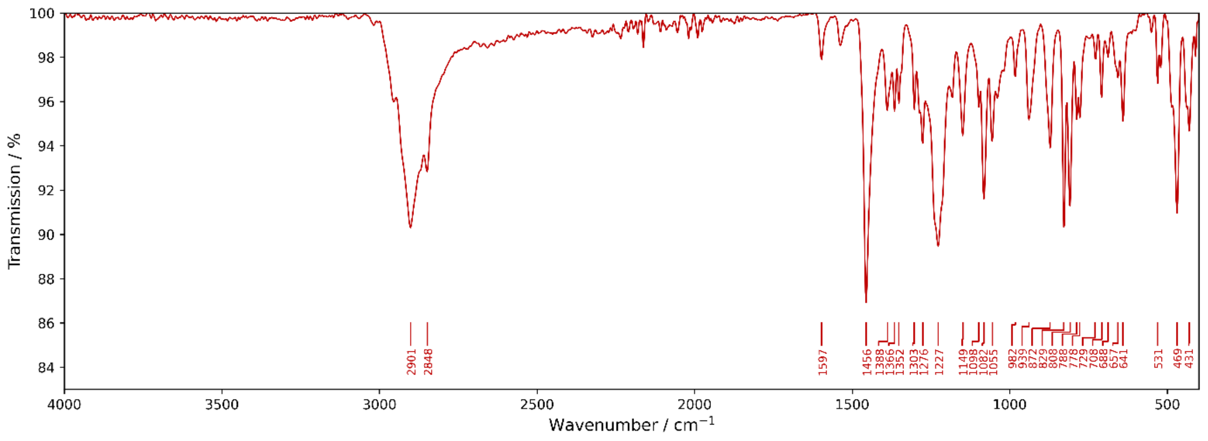

Figure S 268: ATR-IR spectrum of ***3a****.*

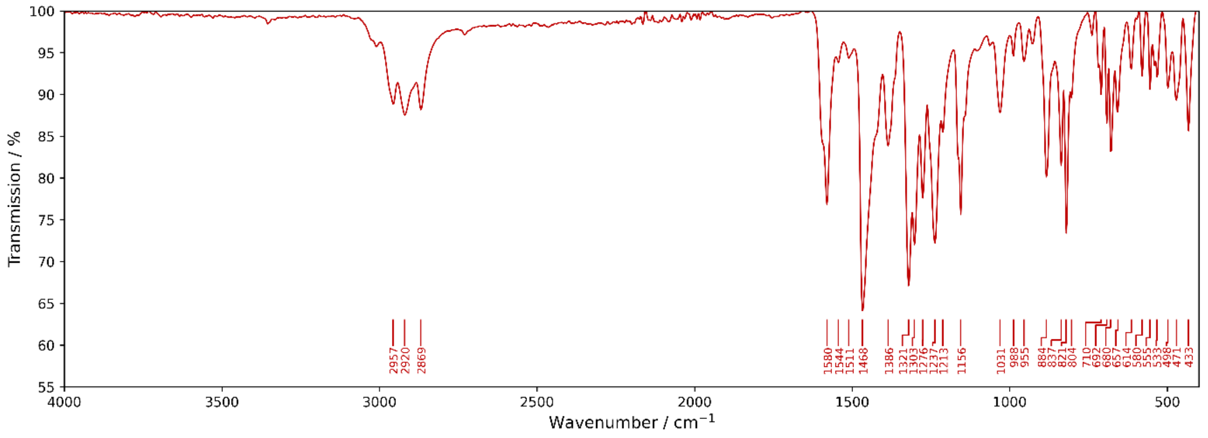

Figure S 269: ATR-IR spectrum of ***3d****.*

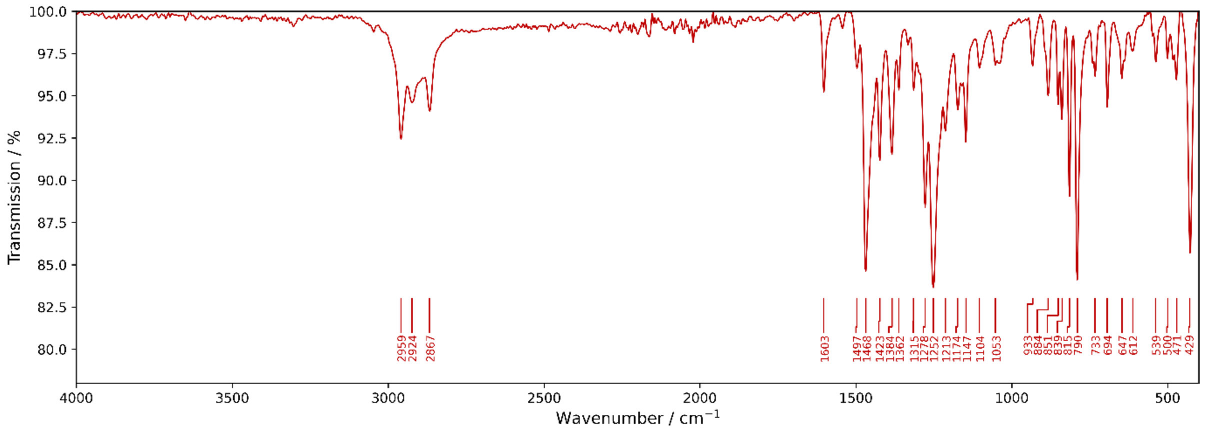

Figure S 270: ATR-IR spectrum of ***3e****.*

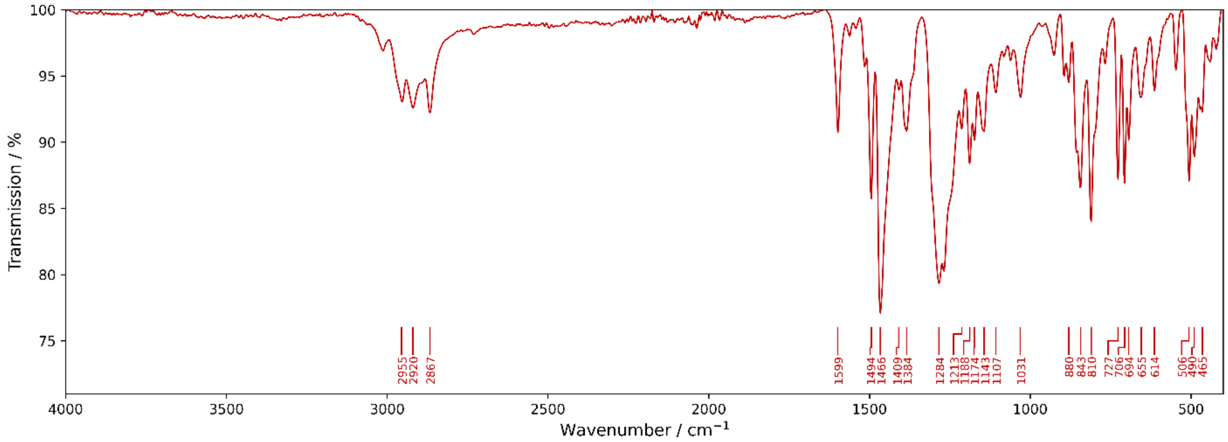

*Figure S 271: ATR-IR spectrum of **3f**.*

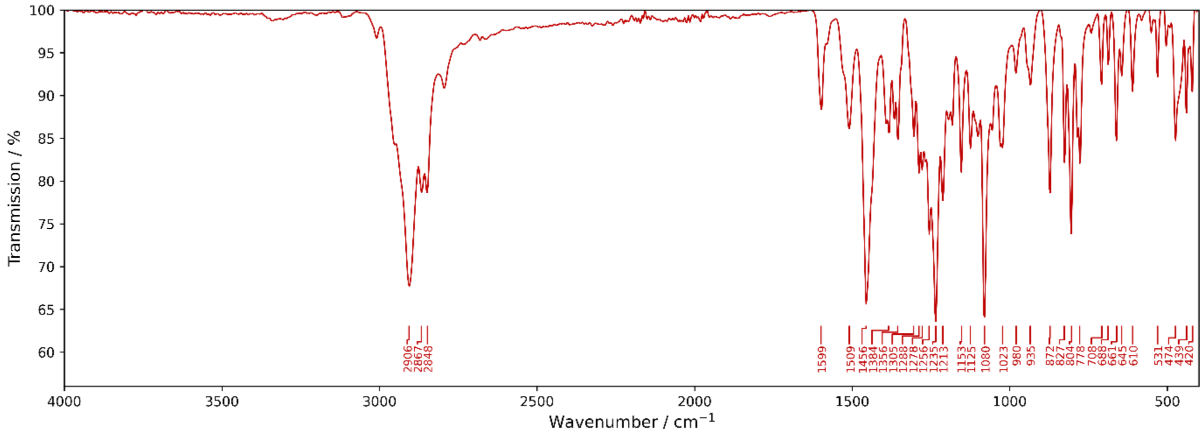

*Figure S 272: ATR-IR spectrum of **4a**.*

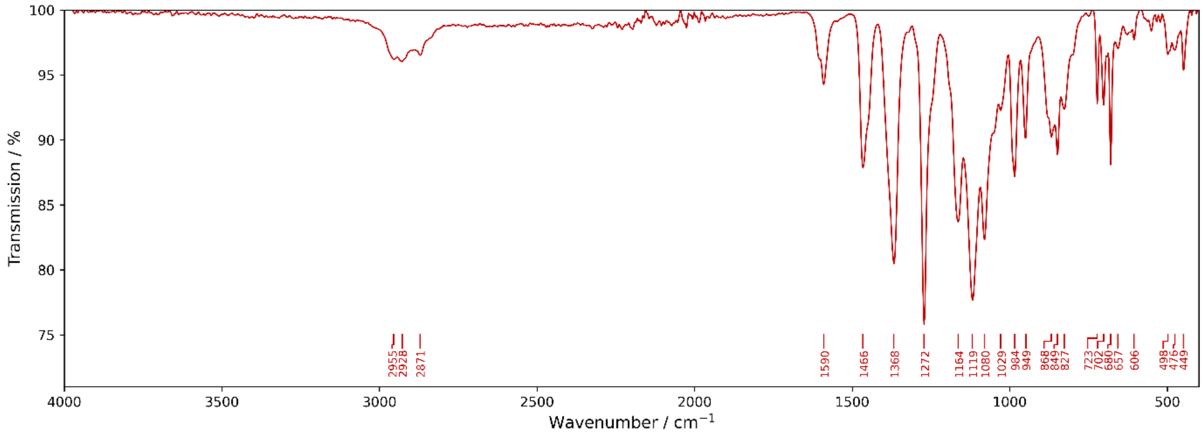

*Figure S 273: ATR-IR spectrum of **4b**.*

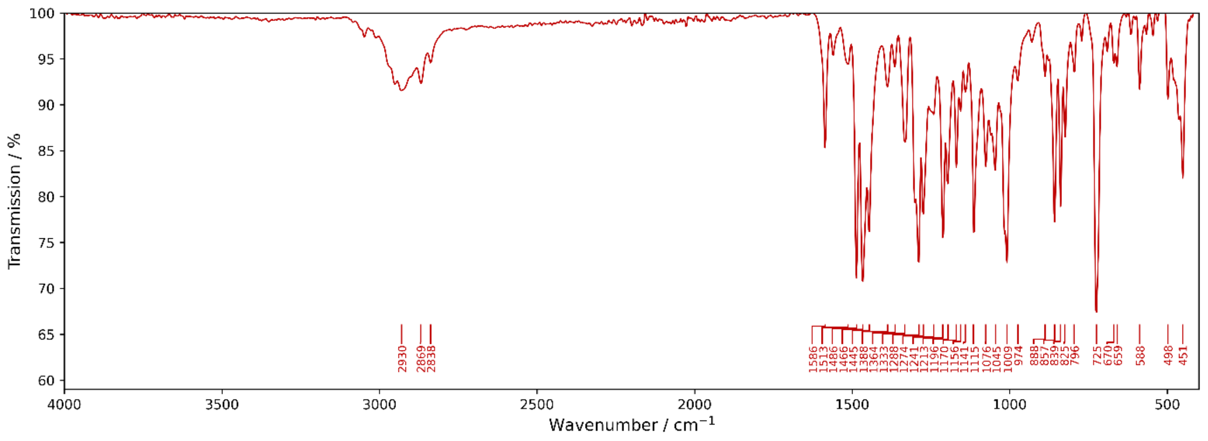

*Figure S 274: ATR-IR spectrum of **4c**.*

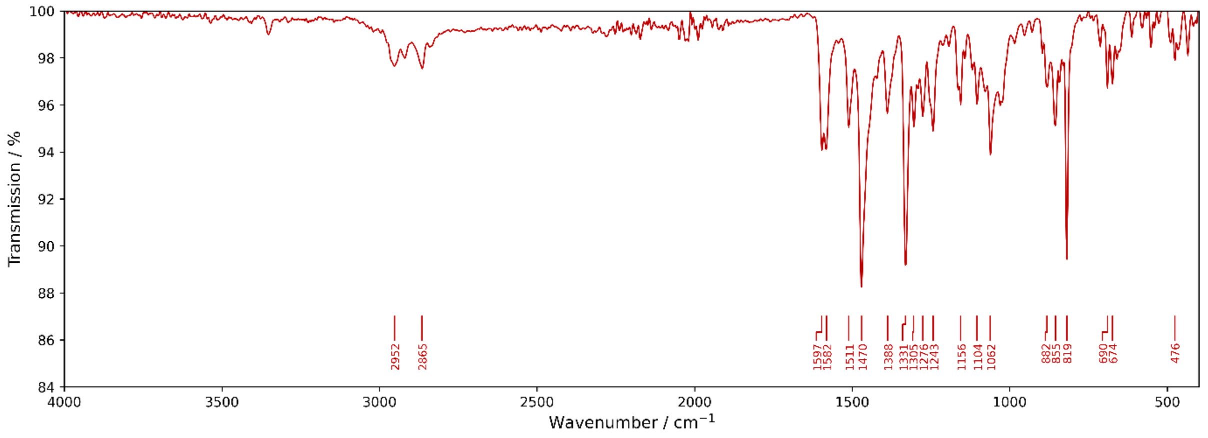

*Figure S 275: ATR-IR spectrum of **4d**.*

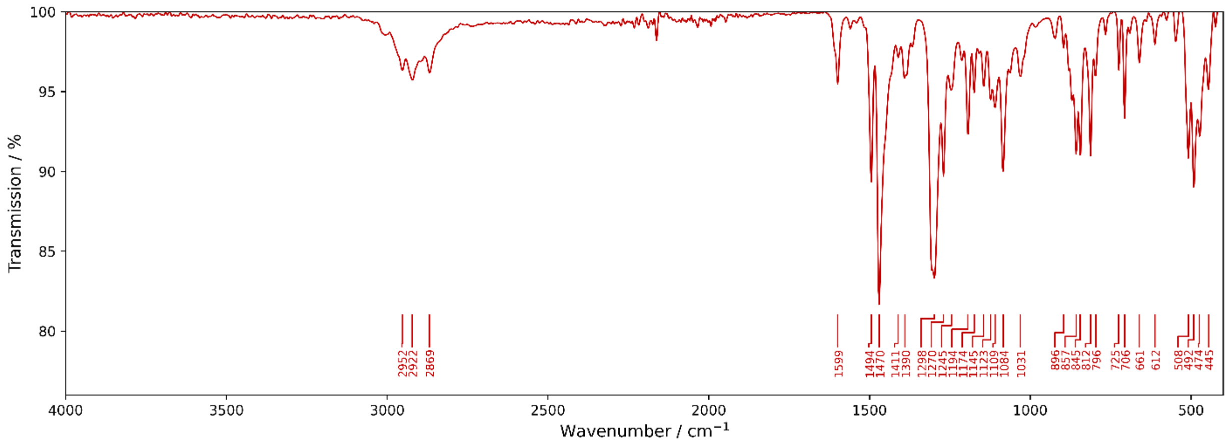

*Figure S 276: ATR-IR spectrum of **4f**.*

Crystallographic details

Table S 1: Crystallographic information on the protonated and lithated ***PN***$^{\textbf{\textit{R}}}$ *ligands.*

	$\mathbf{LiPN^{Ad}}$	$\mathbf{LiPN^{3,5\text{-}CF3}}$	$\mathbf{LiPN^{OMe}}$	$\mathbf{LiPN^{OMe}\ Et_2O}$	$\mathbf{LiPN^{3,5Me}}$	$\mathbf{HPN^{Dipp}}$	$\mathbf{LiPN^{Dipp}}$	$\mathbf{LiPN^{Tol}}$	**X**
Chemical formula	$C_{27}H_{45}N_1O_1P_1Li_1$	$C_{29}H_{43}N_1O_2F_6P_1Li_1$	$C_{40}H_{54}N_2O_2P_2Li_2$ 0.25 (C_6H_{14})	$C_{44}H_{64}N_2O_3P_2Li_1$	$C_{46}H_{68}N_2O_1P_2Li_2$ $C_4H_{10}O_1$	$C_{25}H_{38}N_1P_1$	4 ($C_{50}H_{74}N_2P_2Li_2$)	$C_{44}H_{64}N_2O_1P_2Li_2$	$C_{21}H_{30}N_1O_1P_1$
M_r	437.55	589.55	692.21	744.79	814.96	383.53	3115.71	712.79	343.43
Crystal system	Monoclinic	Monoclinic	Monoclinic	Triclinic	Monoclinic	Triclinic	Triclinic	Monoclinic	Orthorhombic
Space group	$P2_1/n$	$P2_1/n$	C_2/c	P-1	$P2_1/c$	P-1	P-1	C_2/c	$Pna2_1$
a (Å)	10.6161(5)	13.2059(7)	36.3871(15)	10.5493(5)	18.5215(15)	9.2262(4)	10.5033(6)	19.348(7)	16.9081(13)
b (Å)	15.2912(7)	13.76014(7)	15.3993(6)	11.2321(5)	16.4577(16)	9.5170(4)	19.9167(11)	12.321(4)	11.0334(8)
c (Å)	16.2405(6)	18.4307(12)	15.4682(6)	21.0934(8)	16.9763(13)	14.4559(6)	23.1906(14)	18.267(7)	10.5224(9)
α (°)	90	90	90	75.095(2)	90	74.655(2)	82.056(2)	90	90
β (°)	97.143(2)	106.547(2)	105.6970(10)	76.858(2)	90.088(3)	76.514(2)	85.946(2)	97.896(17)	90
γ (°)	90	90	90	65.534(2)	90	71.711(2)	81.401(2)	90	90
V ($Å^3$)	2615.9(2)	3210.4(3)	8344.1(6)	2177.56(17)	5174.7(8)	1146.49(9)	4744.5(5)	4313(3)	1963.0(3)
Z	4	4	8	2	4	2	1	4	4
Densitiy (g cm^{-3})	1.111	1.220	1.102	1.136	1.046	1.111	1.090	1.098	1.162
F(000)	960	1248	2980	804	1776	420	1696	1544	744
Radiation Type	MoKα	MoKα	MoKα	MoKα	MoKα	MoKα	MoKα	MoKα	MoKα
μ (mm^{-1})	0.123	0.145	0.138	0.138	0.120	0.129	0.125	0.134	0.147
Crystal size (mm)	0.25x0.23x0.18	0.20x0.19x0.18	0.15x0.14x0.13	0.25x0.19x0.18	0.25x0.21x0.18	0.28x0.24x0.08	0.10x0.08x0.07	0.15x0.13x0.09	0.19x0.18x0.06
Meas. Refl.	33748	99426	94947	120565	57685	46339	143447	49114	30700
Indep. Refl.	5793	7360	7677	10051	9184	4467	16855	4952	4335
Obsvd. [$I > 2\sigma(I)$]	4384	5275	6523	8845	7795	3875	10616	4323	4164
R_{int}	0.0733	0.1151	0.0565	0.0458	0.0702	0.0953	0.1378	0.0449	0.0345
R_1 [$I > 2\sigma(I)$]	0.0427	0.0837	0.0396	0.0365	0.0565	0.0473	0.0596	0.0401	0.0287
$wR_2(F^2)$	0.1086	0.2480	0.1050	0.1009	0.1383	0.0997	0.1739	0.1077	0.0817
GooF	1.046	1.140	1.036	1.061	1.040	1.053	1.017	1.048	1.055
$\Delta\rho_{max}$ (e $Å^{-3}$)	0.296	1.092	0.215	0.316	0.318	0.260	0.333	0.306	0.155
$\Delta\rho_{min}$ (e $Å^{-3}$)	-0.237	-0.553	-0.210	-0.235	-0.283	-0.212	-0.340	-0.179	-0.231
CCDC	2363267	2363280	2363278	2363277	2363272	2363269	2363275	2363270	2383112

*Table S 2: Crystallographic information on the neutral and anionic lanthanum complexes **2** and **3**.*

	2a	2d	2d‘	2e	3a	3c	3d	3f
Chemical formula	$C_{92}H_{140}N_4P_4Cl_2La_2$ 0.25 C_5H_{12}	$C_{84}H_{116}N_4P_4Cl_2La_2$ 2($C_4H_{10}O_1$)	$C_{46}H_{68}N_2O_2P_2Cl_1La_1$ $C_4H_{10}O_1$	$C_{50}H_{74}N_2P_2Cl_1La_1$	$C_{50}H_{80}N_2O_2P_2La_1Cl_2Li_1$	$C_{44}H_{64}N_2O_4P_2La_1Cl_2Li_1$	$C_{12}H_{30}O_6Li_1$ $C_{42}H_{58}N_2P_2Cl_2La_1$	$C_{12}H_{30}O_6Li_1$ $C_{40}H_{54}N_2P_2Cl_2La_1$
M_r	1792.71	1802.64	991.44	939.41	1019.85	963.66	1139.95	1111.90
Crystal system	Triclinic	Orthorhombic	Monoclinic	Orthorhombic	Orthorhombic	Orthorhombic	Monoclinic	Triclinic
Space group	*P*-1	*Pbcn*	$P2_1/c$	*Aea*2	$P2_12_12_1$	*Pbca*	C_2/c	*P*-1
a (Å)	15.3042(7)	21.1241(7)	14.7160(6)	18.6242(7)	12.822(2)	13.1990(4)	18.0485(8)	15.4560(6)
b (Å)	18.9893(8)	24.4696(8)	12.7924(5)	18.5620(6)	19.436(4)	20.5879(5)	15.9956(9)	19.7155(7)
c (Å)	19.9923(9)	18.5659(5)	28.1582(10)	14.3609(6)	20.464(4)	35.3489(10)	22.9458(13)	20.5964(8)
α (°)	81.019(2)	90	90	90	90	90	90	97.6330(10)
β (°)	88.128(2)	90	104.0680(10)	90	90	90	104.162(2)	94.926(2)
γ (°)	71.314(2)	90	90	90	90	90	90	110.7780(10)
V ($Å^3$)	5435.4(4)	9596.7(5)	5141.9(3)	4964.6(3)	5099.8(17)	9605.7(5)	6423.0(6)	5755.8(4)
Z	2	4	4	4	4	8	4	4
Densitiy (g cm^{-3})	1.095	1.248	1.281	1.257	1.328	1.333	1.179	1.283
F(000)	1877	3760	2080	1968	2136	3984	2392	2328
Radiation Type	MoKα	MoKα	MoKα	MoKα	MoKα	MoKα	MoKα	MoKα
μ (mm^{-1})	0.921	1.045	0.985	1.012	1.044	1.107	0.840	0.936
Crystal size (mm)	0.20x0.18x0.16	0.25x0.23x0.17	0.x15x0.14x0.09	0.26x0.24x0.15	0.15x0.14x0.13	0.15x0.14x0.13	0.10x0.08x0.03	0.18x0.16x0.11
Meas. Refl.	168251	118304	132875	67779	35302	98201	61953	29112
Indep. Refl.	20000	8455	11811	5940	8386	11046	6303	29112
Obsvd. [$I > 2\sigma(I)$]	17117	6118	7791	5349	5229	7907	5375	23675
R_{int}	0.0561	0.1218	0.1440	0.0396	0.1764	0.0860	0.0525	-
R_1 [$I > 2\sigma(I)$]	0.0260	0.0384	0.0483	0.0202	0.0748	0.0405	0.0388	0.0565
$wR_2(F^2)$	0.0715	0.0815	0.1234	0.0515	0.1941	0.0728	0.0959	0.0954
GooF	1.041	1.047	1.011	1.027	1.080	1.070	1.050	1.057
$\Delta\rho_{max}$ (e $Å^{-3}$)	0.434	0.504	1.250	0.381	1.243	0.403	0.357	0.721
$\Delta\rho_{min}$ (e $Å^{-3}$)	-0.318	-0.508	-0.875	-0.264	-0.750	-0.486	-0.368	-0.822
CCDC	2363279	2363281	2363276	2363271	2363266	2363274	2363273	2363268

Table S 3: Selected bond lengths and angles for the protonated and lithated PNR ligands.

	**LiPN^{Ad}**	**LiPN$^{3,5\text{-}CF3}$**	**LiPN^{OMe}**	**LiPN^{OMe} Et$_2$O**	**LiPN^{3,5Me}**	**HPN^{Dipp}**	**LiPN^{Dipp}**	**LiPN^{Tol}**	**X**
Li1 – N1	1.933(3)	2.005(6)	2.065(3)	2.134(2)	2.041(6)	-	1.959(5)	2.026(3)	-
Li1 – N2	-	-	2.047(3)	2.145(2)	2.055(6)	-	-	-	-
Li2 – N1	-	-	2.056(3)	2.068(2)	2.045(6)	-	-	2.047(2)	-
Li2 – N2	-	-	2.061(3)	2.053(2)	2.051(6)	-	-	-	-
Li1 – P1	2.480(3)	2.660(5)	2.487(3)	-	-	-	2.466(5)	-	-
Li1 – P2	-	-	-	-	-	-	-	-	-
Li2 – P1	-	-	-	2.508(2)	2.503(6)	-	-	2.5357(17)	-
Li2 – P2	-	-	2.495(3)	2.512(2)	2.502(6)	-	-	-	-
Li1 – O1/O100	1.943(3)	1.947(6)	-	2.120(2) / 2.038(3)	1.874(5)	-	-	1.903(4)	-
Li1 – O2/O200	-	1.966(5)	1.904(3)	2.104(2)	-	-	-	-	-
Li2 – O1/O100	-	-	1.931(3)	-	-	-	-	-	-
Li2 – O2/O200	-	-	-	-	-	-	-	-	-
N1 – C10	1.3588(19)	1.409(3)	1.411(2)	1.4088(14)	1.425(4)	1.3651(18)	1.375(3)	1.4128(16)	1.346(3)
C10 – C11	1.441(2)	1.412(4)	1.413(2)	1.4116(16)	1.420(4)	1.411(2)	1.428(4)	1.4122(18)	1.429(3)
C11 – P1	1.8206(14)	1.832(3)	1.8303(17)	1.8298(11)	1.815(3)	1.8395(15)	1.805(3)	1.8269(13)	1.776(2)
N1 – Li1/2 – P1	81.98(10)	77.33(18)	83.85(10)	82.32(7)	80.1(2)	-	85.28(18)	77.05(4)	-

*Table S 4: Selected bond lengths and angles for the neutral and anionic lanthanum complexes **2** and **3.***

	**2a**	**2d**	**2d'**	**2e**	**3a**	**3c**	**3d**	**3f**
La1 – La2	4.642(1)	4.643(1)	-	-	-	-	-	-
La1 – Li1	-	-	-	-	3.59(4)	3.741(7)	-	-
La1 – P1	3.0696(6)	3.1898(10)	3.1914(11)	3.2132(6)	3.151(5)	3.1890(8)	3.1300(8)	3.1218(10)
La1 – P2	3.0657(6)	3.2085(11)	3.2064(11)	-	3.129(5)	3.1947(9)	-	3.1663(10)
La1 – N1	2.3908(19)	2.414(3)	2.460(3)	2.399(2)	2.474(15)	2.468(2)	2.473(2)	2.482(3)
La1 – N2	2.371(2)	2.429(3)	2.468(3)	-	2.469(14)	2.452(2)	-	2.476(3)
La1 – O1	-	-	2.602(3)	-	-	2.658(2)	-	-
La1 – O2	-	-	2.669(3)	-	-	2.632(2)	-	-
La1 – Cl1	2.8970(6)	2.9205(8)	2.7596(12)	2.6745(12)	2.854(5)	2.8560(9)	2.7476(9)	2.7265(10)
La1 – Cl2 / Cl1A	2.8797(6)	2.8628(8)	-		2.935(4)	2.9192(9)	-	2.7449(10)
Li1 – Cl1	-	-	-	-	2.31(4)	2.317(9)	-	-
Li1 – Cl2	-	-	-	-	2.30(4)	2.300(8)	-	-
N1 – C10	1.410(3)	1.395(5)	1.397(5)	1.387(3)	1.39(2)	1.415(4)	1.384(4)	1.397(5)
C10 – C11	1.415(4)	1.419(5)	1.419(6)	1.415(4)	1.41(2)	1.411(4)	1.424(4)	1.420(5)
C11 – P1	1.837(3)	1.822(4)	1.817(4)	1.818(3)	1.85(2)	1.830(3)	1.814(3)	1.815(4)
P1 – La1 – N1	62.95(5)	62.05(7)	61.51(8)	63.20(5)	62.5(3)	60.14(6)	62.37(6)	
P2 – La1 – N2	63.17(5)	61.85(8)	62.55(8)	-	63.8(4)	62.48(6)	-	
N1 – La1 – N2	112.23(7)	125.95(11)	119.75(11)	128.71(12)	91.7(5)	101.50(8)	98.44(11)	95.27(10)
P1 – La1 – P2	141.021(17)	117.59(3)	93.92(3)	171.14(13)	163.12(15)	129.20(2)	165.98(3)	167.05(3)
N1/2 – La1 – Cl1	116.09(5)	109.76(8)	90.27(9)	115.64(6)	105.0(3)	146.93(6)	140.30(6)/95.55(6)	137.83(7)
N1/2 – La1 – Cl1A/Cl2	116.35(5)	93.93(8)	-	-	143.8(3)	100.53(6)	-	100.24(8)
P1/2 – La1 – Cl1	144.101(18)	88.50(3)	89.04(4)	85.570(16)	83.79(14)	148.26(3)	90.76(2)/79.91(2)	81.01(3)
P1/2 – La1 – Cl1A/Cl2	141.694(17)	153.89(3)	78.21(3)	-	85.66(14)	143.45(3)	-	91.48(3)
Cl1 – La1 – Cl1A/Cl2	72.991(16)	73.18(3)	-	-	80.11(12)	76.15(3)	96.94(4)	98.76(4)
τ_5 / τ_4'	-	-	-	0.71	-	-	-	-

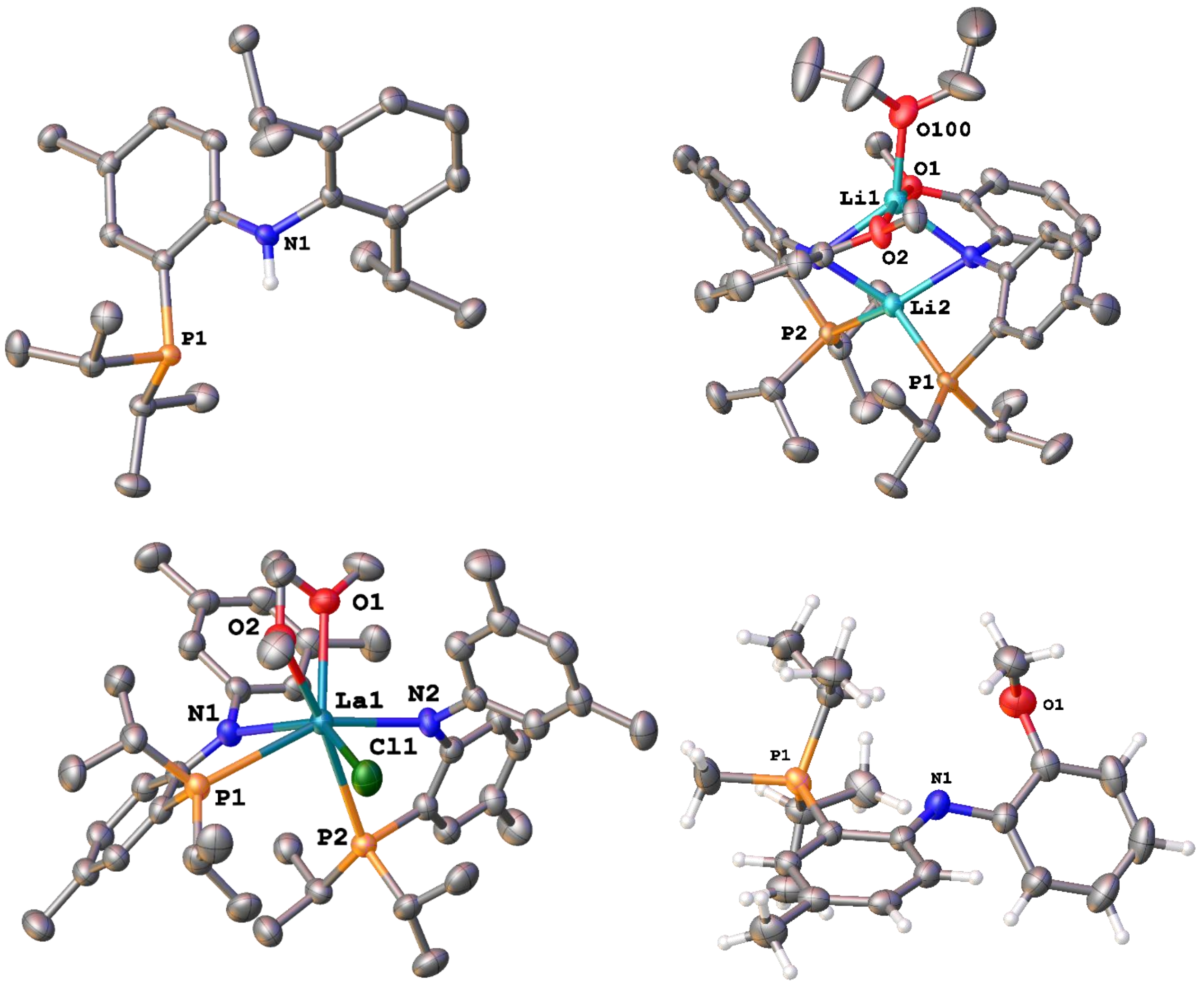

Figure S 277: Molecular structures of ***HPN^{Dipp}*** *(top. left)* ***$LiPN^{OMe}$ Et_2O*** *(top, right), the DME coordinated complex* ***2d'*** *(bottom, left) and the decomposition product* ***X*** *from the reaction between* ***$LiPN^{OMe}$*** *and $LaCl_3(THF)_{1.2}$ in boiling toluene (bottom, right). Hydrogen atoms and solvent lattice molecules have been omitted for clarity. Ellipsoids are shown at a probability level of 50%.*

Buried Volume

*Table S 5: %V_{buried} for complex **LiPN^{Ad}** (left) and complex **LiPN^{3,5CF3}** (right).*[1]

%V Free	%V Buried	% V Tot/V Ex
52.9	**47.1**	99.9

%V Free	%V Buried	% V Tot/V Ex
60.1	**39.9**	99.9

Quadrant	V f	V b	V t	%V f	%V b
SW	11.2	33.7	44.9	**25.0**	75.0
NW	38.3	6.5	44.9	**85.4**	14.6
NE	21.4	23.5	44.9	**47.7**	52.3
SE	23.9	20.9	44.9	**53.4**	46.6

Quadrant	V f	V b	V t	%V f	%V b
SW	41.0	3.9	44.9	**91.3**	8.7
NW	23.7	21.1	44.9	**52.9**	47.1
NE	20.3	24.6	44.9	**45.2**	54.8
SE	22.9	22.0	44.9	**51.0**	49.0

*Table S 6: %V_{buried} for complex **LiPN^{OMe}** (left) and complex **LiPN^{3,5Me}** (right).*[1]

%V Free	%V Buried	% V Tot/V Ex
55.5	**44.5**	99.9

%V Free	%V Buried	% V Tot/V Ex
56.7	**43.3**	99.9

Quadrant	V f	V b	V t	%V f	%V b
SW	37.3	7.5	44.9	**83.2**	16.8
NW	16.5	28.3	44.9	**36.9**	63.1
NE	23.9	21.0	44.9	**53.2**	46.8
SE	22.0	22.9	44.9	**48.9**	51.1

Quadrant	V f	V b	V t	%V f	%V b
SW	19.2	25.6	44.9	**42.9**	57.1
NW	39.9	5.0	44.9	**88.8**	11.2
NE	22.0	22.9	44.9	**48.9**	51.1
SE	20.8	24.1	44.9	**46.3**	53.7

*Table S 7: %V_{buried} for complex **LiPN^{Dipp}** (left) and complex **LiPN^{Tol}** (right).*[1]

%V Free	%V Buried	% V Tot/V Ex
53.6	**46.4**	99.9

%V Free	%V Buried	% V Tot/V Ex
57.9	**42.1**	99.9

Quadrant	V f	V b	V t	%V f	%V b
SW	29.2	15.6	44.9	**65.1**	34.9
NW	19.8	25.0	44.9	**44.2**	55.8
NE	26.5	18.4	44.9	**59.0**	41.0
SE	20.7	24.1	44.9	**46.2**	53.8

Quadrant	V f	V b	V t	%V f	%V b
SW	40.5	4.4	44.9	**90.3**	9.7
NW	22.7	22.2	44.9	**50.6**	49.4
NE	18.3	26.6	44.9	**40.8**	59.2
SE	22.3	22.5	44.9	**49.8**	50.2

*Table S 8: %V_{buried} for complex **3a** (left) and complex **3d** (right).*[1]

%V Free	%V Buried	% V Tot/V Ex
59.8	**40.2**	99.9

%V Free	%V Buried	% V Tot/V Ex
57.5	**42.5**	99.9

Quadrant	V f	V b	V t	%V f	%V b
SW	28.2	16.6	44.9	**63.0**	37.0
NW	25.3	19.6	44.9	**56.3**	43.7
NE	28.6	16.3	44.9	**63.6**	36.4
SE	25.3	19.6	44.9	**56.4**	43.6

Quadrant	V f	V b	V t	%V f	%V b
SW	22.6	22.2	44.9	**50.5**	49.5
NW	27.6	17.3	44.9	**61.4**	38.6
NE	25.2	19.7	44.9	**56.1**	43.9
SE	27.8	17.0	44.9	**62.0**	38.0

*Table S 9: %V_{buried} for complex **3e**.*[1]

%V Free	%V Buried	% V Tot/V Ex
39.5	**60.5**	99.9

Quadrant	V f	V b	V t	%V f	%V b
SW	19.4	25.4	44.9	**43.4**	56.6
NW	16.0	28.8	44.9	**35.7**	64.3
NE	19.4	25.4	44.9	**43.4**	56.6
SE	16.0	28.8	44.9	**35.7**	64.3

*Table S 10: %V_{buried} for complex **4a** (left) and complex **4c** (right).*[1]

%V Free	%V Buried	% V Tot/V Ex
51.1	**48.9**	99.9

%V Free	%V Buried	% V Tot/V Ex
52.0	**48.0**	99.9

Quadrant	V f	V b	V t	%V f	%V b
SW	25.6	19.2	44.9	**57.1**	42.9
NW	20.1	24.7	44.9	**44.9**	55.1
NE	25.3	19.5	44.9	**56.5**	43.5
SE	20.6	24.3	44.9	**45.9**	54.1

Quadrant	V f	V b	V t	%V f	%V b
SW	20.9	24.0	44.9	**46.5**	53.5
NW	27.2	17.7	44.9	**60.6**	39.4
NE	18.9	26.0	44.9	**42.0**	58.0
SE	26.5	18.4	44.9	**59.0**	41.0

*Table S 11: %V_{buried} for complex **4d** (left) and complex **4f** (right).*[1]

%V Free	%V Buried	% V Tot/V Ex
44.1	**55.9**	99.9

%V Free	%V Buried	% V Tot/V Ex
43.2	**56.8**	99.9

Quadrant	V f	V b	V t	%V f	%V b
SW	21.3	23.6	44.9	**47.5**	52.5
NW	18.3	26.6	44.9	**40.7**	59.3
NE	21.3	23.6	44.9	**47.5**	52.5
SE	18.3	26.6	44.9	**40.7**	59.3

Quadrant	V f	V b	V t	%V f	%V b
SW	16.2	28.7	44.9	**36.0**	64.0
NW	21.1	23.7	44.9	**47.1**	52.9
NE	19.0	25.9	44.9	**42.3**	57.7
SE	21.2	23.7	44.9	**47.2**	52.8

Literature

[1] Falivene, L.; Cao, Z.; Petta, A.; Serra, L.; Poater, A.; Oliva, R.; Scarano, V.; Cavallo, L. *Nat. Chem.* **2019**, *11*, 872.
